# Identification of diagnostic biomarks and immune cell infiltration in ulcerative colitis

**DOI:** 10.1038/s41598-023-33388-5

**Published:** 2023-04-13

**Authors:** Qin Chen, Shaosheng Bei, Zhiyun Zhang, Xiaofeng Wang, Yunying Zhu

**Affiliations:** 1 Department of Anorectal, Kunming Municipal Hospital of Traditional Chinese Medicine, The Third Affiliated Hospital of Yunnan University of Chinese Medicine, No. 25 Dongfeng East Road, Panlong District, Kunming, 650011 Yunnan Province China; 2 Department of Anorectal, Xiyuan Hospital, China Academy of Chinese Medical Sciences, Beijing, China; 3 Department of Colorectal Surgery, Guang’an Men Hospital, China Academy of Chinese Medical Sciences, Beijing, China

**Keywords:** Biomarkers, Gastroenterology

## Abstract

We aimed to explore diagnostic biomarks and immune cell infiltration characteristics in ulcerative colitis (UC). We used the dataset GSE38713 as the training set and dataset GSE94648 as the test set. A total of 402 differentially expressed genes (DEGs) were obtained from GSE38713. Annotating, visualizing, and integrating discovery of these differential genes was performed using Gene Ontology (GO), Kyoto Gene and Genome Encyclopedia Pathway (KEGG), and Gene Set Enrichment Analysis (GSEA). Protein–protein interaction networks were constructed from the STRING database, and protein functional modules were identified using the CytoHubba plugin of Cytoscape. Random forest and LASSO regression were used to screen for UC-related diagnostic markers, and ROC curves were generated to validate their diagnostic value. The composition of 22 immune cells was analyzed, and the immune cell infiltration in UC was analyzed using CIBERSORT. Results: Seven diagnostic markers associated with UC were identified: TLCD3A, KLF9, EFNA1, NAAA,WDR4, CKAP4, and CHRNA1. Immune cell infiltration assessment revealed that macrophages M1, activated dendritic cells, and neutrophil cells infiltrated relatively more compared to normal control samples. Our results suggest a new functional feature of UC and suggest potential biomarkers for UC through comprehensive analysis of integrated gene expression data.

## Introduction

Ulcerative colitis (UC) is a chronic nonspecific inflammatory disease of the intestine, that is characterized by persistent or recurrent abdominal pain, diarrhea, and mucopurulent stools. The incidence and prevalence of UC are increasing worldwide^1^, thus increasing the medical and economic burden on society. Therefore, the exploration of diagnostic biomarkers and therapeutic targets has become a focal issue for improving the prognosis of UC.

The etiology and pathogenesis of UC are still unclear, and current research suggests that it is mainly caused by the interaction of genetic susceptibility, epithelial barrier defects, immune system dysfunction, and environmental factors^2,3^. Among all the factors, an impaired immune response plays an important role in the development and progression of UC^4^. Both innate and adaptive immunity have been shown to play important roles in intestinal inflammation^5^. When the tolerance mechanisms of the intestinal barrier fail, local immune cells are stimulated, resulting in production of chemokines and subsequent infiltration of immune cells. Thus, the inflammatory process is further exacerbated^4^. Studies have shown that the cytokines interleukin (IL)-13, TNF, IL-23, IL-9, and IL-36 promote inflammatory immune cell infiltration and are important in the pathogenesis of UC^6^. Different types of immune cells, whether in an activated or inactivated state, can modulate the immune response by inhibiting, maintaining, or promoting the development of UC^7^.

With the completion of the Human Genome Project, histological technologies, mainly high-throughput microarray analysis and bioinformatics analysis, have provided reliable technical support for studying the pathological mechanisms of complex diseases^8^. Several relevant studies have used microarray analysis to show the involvement of differentially expressed genes (DEGs) in biological functions and pathways contributing to the development of UC, as well as potential biomarkers that are immunologically relevant to patients with UC^9,10^. However, the biomarkers that have been identified are still less accurate in the diagnosis and prognosis of UC, mainly due to the complexity of UC pathogenesis. Different microarray platforms and small sample sizes may have led to inconsistent results in these studies. Further comprehensive analyses are necessary to identify new, more reliable diagnostic biomarkers and therapeutic targets to overcome these inconsistencies.

Therefore, in the current study, we screened DEGs in UG samples using microarray sequencing of UC from the Gene Expression Omnibus (GEO) database, which included 30 UC patients and 13 normal controls and performed functional enrichment analysis of Gene Ontology (GO), Kyoto Gene and Genome Encyclopedia Pathway (KEGG), and Gene Set Enrichment Analysis (GSEA), and constructed a protein–protein interaction (PPI) network to explore important protein action modules, while random forest and LASSO regression were used to screen for the diagnostic markers of UC. We also used another dataset for ROC validation, and used CIBERSORT^11^ to calculate its immune cell composition and analyze its correlation with UC. We assessed the immune cell infiltration in UC, which provides new ideas for further research on the molecular mechanism underlying UC pathogenesis.

## Materials and methods

### Data download and pre-processing

UC expression profiles with reliable sample sources were downloaded from the GEO (https://www.ncbi.nlm.nih.gov/geo/) database using the GEOquery package^12^of the R software (version 3.6.5, http://r-project.org/). The dataset GSE38713^13^ and GSE94648^14^ with samples from *Homo sapiens* and platforms based on GPL570 and GPL19109 [HG-U133_Plus_2] Affymetrix Human Genome U133 Plus 2.0 Array were used. The GSE38713 dataset included 30 UC patient samples and 13 normal samples and GSE94648 dataset included 25 UC patient samples and 22 normal samples, both of which were included in this study (Table 1). The raw data of the GSE38713 and GSE94648 datasets were read using the affy package^15^; RMA background correction, and data normalization were performed to obtain the gene expression matrices of the two datasets. HUGO Gene Nomenclature Committee (HGNC)^16^ is responsible for providing a unique, standardized and widely disseminated symbol for all genes on the human genome including protein-coding genes, non-coding RNA genes, methyl genes and other genes; for each human gene, mRNA expression profiles were obtained using the HGNC mRNA gene annotation file.

**Table1:** Related information of dataset Platform (Affymetrix Human Genome U133 Plus 2.0 Array).

Dataset	Patient	Control
GSE38713	30	13
GSE94648	25	22

### Identification of DEGs

We used GSE38713 as the training set and GSE94648 as the test set. The GSE38713 dataset was screened for differentially expressed genes (DEGs) using the limma package^17^,and the volcano plot of DEGs was plotted using the ggplot2^18^ package. Criterion for selection was adj. *p* value < 0.05, and | log2FC|> 1. Around 402 genes were found to be differentially expressed.

### PPI Network Analysis and Identification of Key Genes

The STRING^19^ database searches were used to identify interactions between known proteins and predicted proteins. We used the DEGs obtained from differential expression analysis and put them into the STRING database to obtain their protein interaction networks, and then put the networks into Cytoscape^20^ software to identify the genes that interact more strongly with other genes and visualize them. Using the MCODE^21^ plugin to identify its sub-networks and based on the score, the three highest-rated sub-networks were obtained, which we believed may serve a specific function.

### Functional enrichment analysis

GO^22^ is a database established by the Gene Ontology Consortium to create a semantic vocabulary standard for qualifying and describing gene and protein functions for a wide range of species that can be updated as research progresses. GO annotations are divided into three broad categories: molecular function (MF), biological process (BP), and cellular components (CC). KEGG^23–25^ is a comprehensive database that integrates genomic, chemical, and systemic functional information. KEGG database specifically stores information about gene pathways in different species. Metascape^26^ is a web tool that provides a variety of functions such as gene enrichment analysis and protein interaction network analysis. The website integrates more than 40 gene function annotation databases and provides diverse visualizations. We used Metascape to perform GO/KEGG functional enrichment analysis of differentially expressed genes, selecting functions with p < 0.01, minimum count of 3, and enrichment factor > 1.5. We also used the R package Pathview^27^ to visualize the more important pathways in KEGG and R package ggplot2 to visualize the more important functions in GO.

### GSEA functional enrichment analysis

GSEA^28^ is based on the idea of using predefined gene sets (usually from functional annotations or results of previous experiments) to rank genes according to their differential expression in two types of samples, and then testing whether the predefined set of genes is enriched at the top or bottom of the ranking table. We used the clusterProfiler package^29^ to analyze the gene expression profile of GSE38713 using the GSEA method, selecting "c2.cp.kegg.v7.4.symbols.gmt" and "c5.go.bp.v7.4.symbols.gmt" as the reference gene set^30^ , and *p* < 0.05 was considered significantly enriched.

### Random Forest identification for signature genes

For the 402 differentially expressed genes obtained, the RandomForest package was used to filter the feature genes. RandomForest^31^ package in R was used to construct a random forest for the 402 differentially expressed genes. The larger the Gini coefficient, the better the classification, and the larger the decrease in the Gini value when selecting a certain point, the better the classification. The parameter MeanDecreaseGini^30^ is the average decreasing GINI value, the more it decreases, the better the classification effect of this node. This node is chosen as the classification node, that is, the node with the largest GINI value as the classification node. The first 2/3 nodes with the best classification effect (keeping the largest MeanDecreaseGini and removing the first 1/3 nodes with small ones) were selected, and the nodes with poor classification effect were removed. Finally, a total of six rounds were screened to obtain 54 feature genes that contributed more to the classification.

### Identification and validation of diagnostic markers

LASSO is a shrinkage estimation method that allows variable selection by constructing a penalty function that can compress the coefficients of variables and make regression coefficients zero. We used the LASSO regression algorithm for feature selection to screen for diagnostic markers of UC based on feature genes obtained from random forest. The GSE94648 dataset was used as a test set to validate the diagnostic efficacy of the obtained diagnostic markers, and use GEPIA2^32^ to analyze the prognosis of the obtained diagnostic markers and UC-related Colorectal Cancer (CRC) in the TCGA database.

### Immune cell infiltration analysis

CIBERSORT^11^ is based on the principle of linear support vector regression to deconvolute the transcriptome expression matrix and to estimate the composition and abundance of immune cells in a mixture of cells^33^. We downloaded the original code and the corresponding immune cell files from the CIBERSORT official website and derived the immune cell infiltration matrix in R based on the gene expression profile of GSE38713 and the immune cell files. We used the corrplot package^34^ to plot correlation heat maps and visualize the correlation of the 22 immune cell infiltrates. The ggplot2 package was used to plot box line plots for visualizing the differences between the infiltration of 22 immune cells; igraph package^35^ was used to plot correlation network plots of immune cell infiltrates for visualizing the interactions of the 22 immune cell infiltrates, and *p* < 0.05, |correlation coefficient > 0.4) were used as the criteria for interactions. We correlated the obtained diagnostic markers with immune cell infiltrates and then visualized the results using the pheatmap^36^ package.

## Results

### Data download and pre-processing

**Figure 1:**
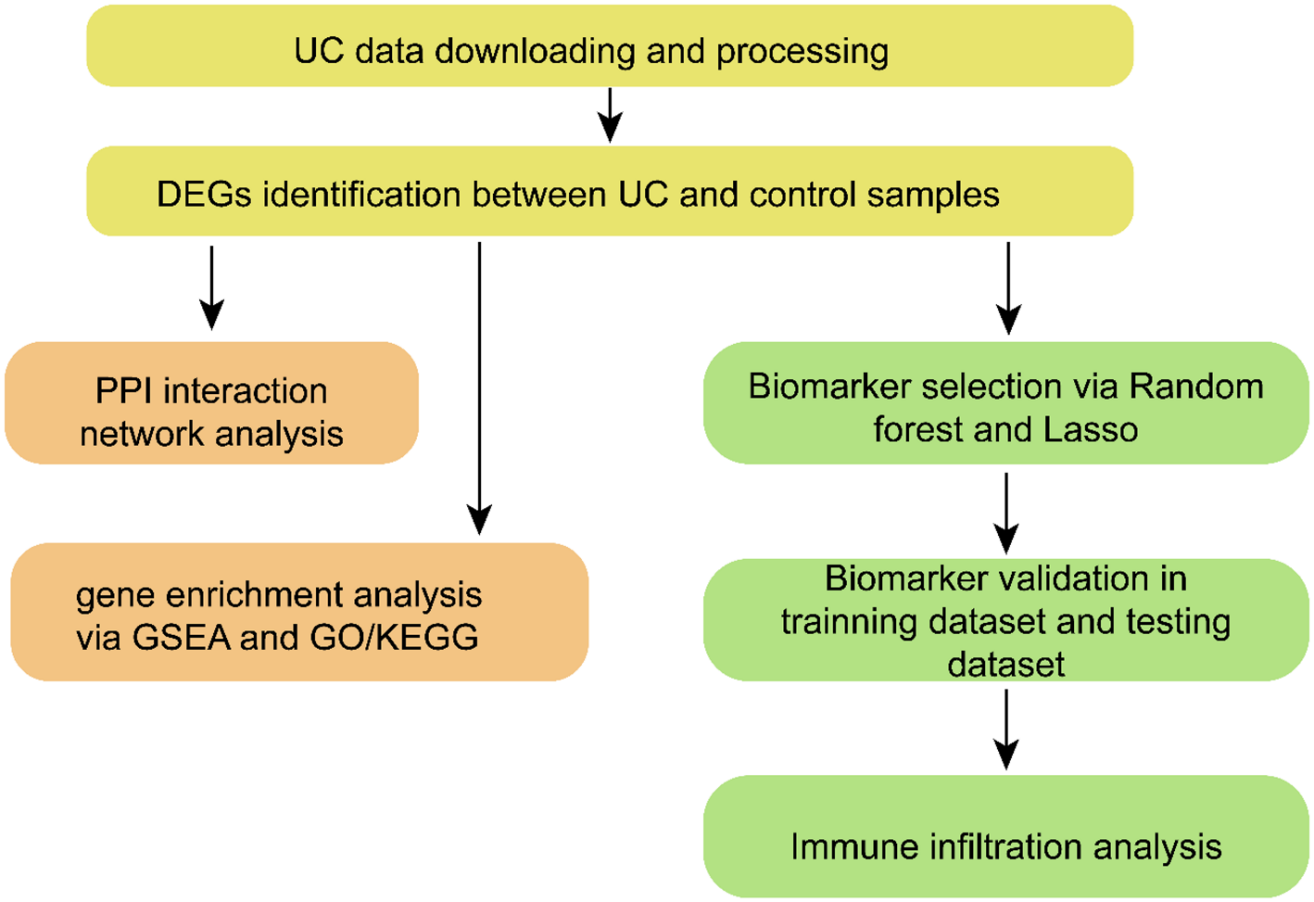
Flow chart of data analysis.

**Figure 2:**
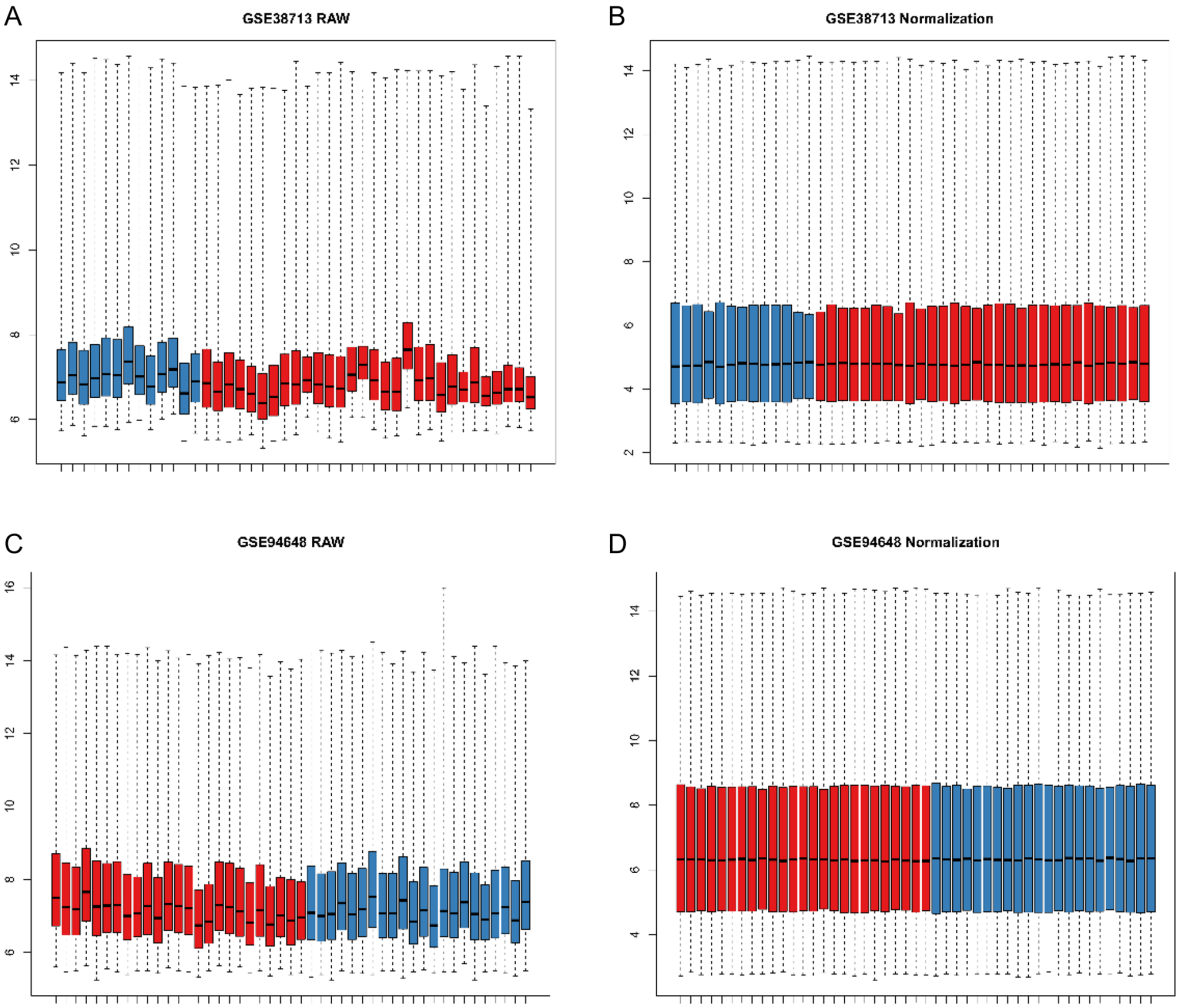
Data box plots. (**A**) GSE38713 uncorrected box plot; (**B**) GSE38713 corrected box plot; (**C**) GSE94648 uncorrected box plot; (**D**) GSE94648 corrected box plot. Blue represents normal samples, red represents disease samples.

The data analysis process is illustrated in Fig. 1. First, the gene expression matrices from the GEO official website GSE38713 and GSE94648 datasets (Table 1) were normalized and processed based on the RMA method using the affy package. The two datasets were found to be more suitable for analysis since they had more positive data (Fig. 2). The protein gene annotation files were downloaded from HGNC, and 16,930 mRNAs were obtained after matching.

### DEG Analysis

**Figure 3:**
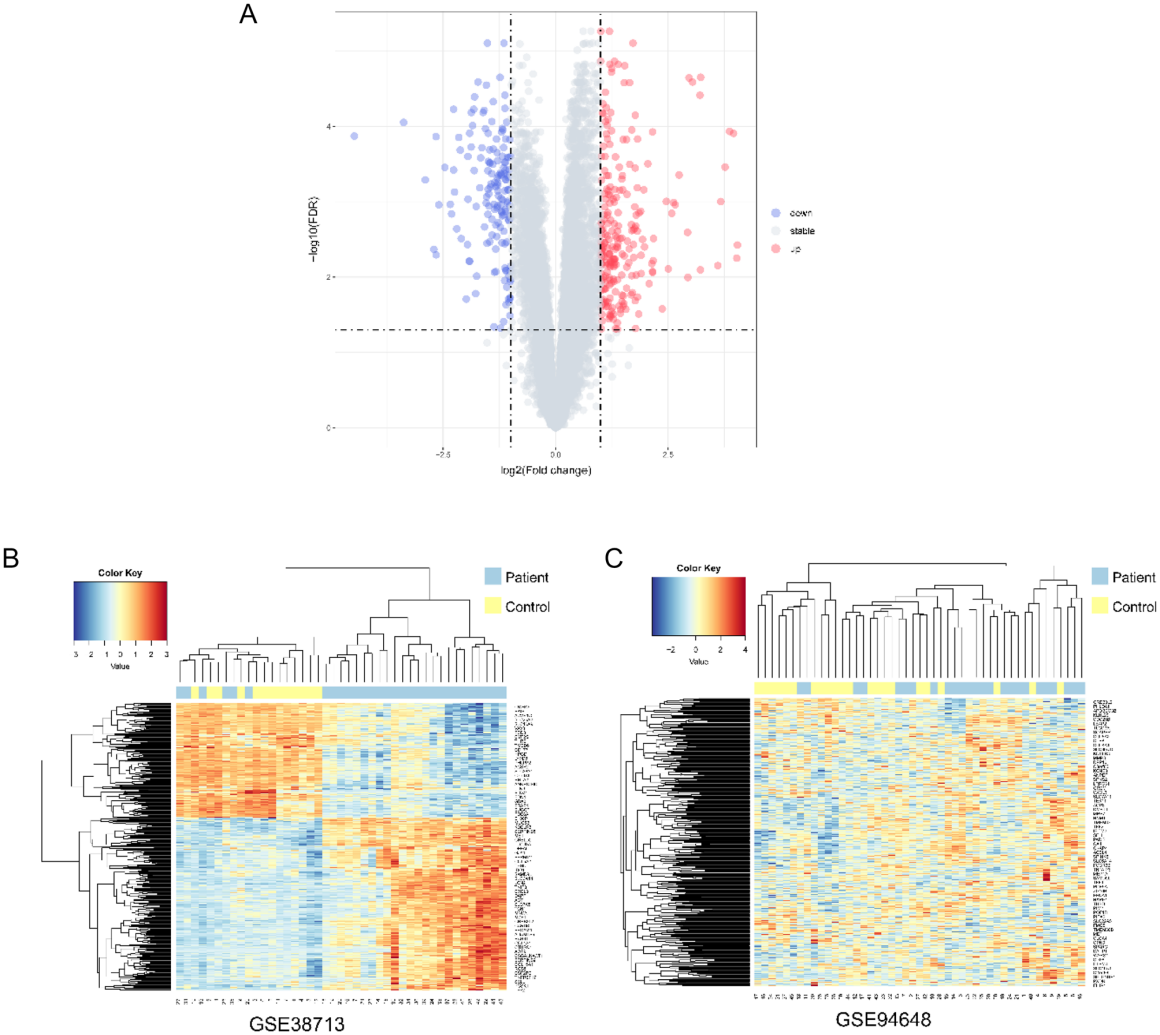
Volcano and Heat map. (**A**) Volcano map of GSE38713 DEGs, red represents up-regulated differential genes, blue represents down-regulated differential genes, and gray represents no differential genes; (**B**) heat map of GSE38713 clusters; (**C**) heat map of GSE94648 clusters. Yellow represents the control group; blue represents the UC group.

After data preprocessing, we performed differential expression analysis on the GSE38713 expression matrix using the R package limma, with |logFC| > 1 and adj. *p* value < 0.05 as the threshold screening, and a total of 402 DEGs, 242 upregulated genes, and 160 downregulated genes were extracted from the gene expression matrix. The distribution of DEGs is shown in the volcano plot (Fig. 3A). We then performed a hierarchical clustering analysis of the 402 DEGs in GSE38713 and GSE94648, and found that the majority of disease samples were clustered into one category and normal samples were clustered into a different category (Fig. 3B,C).

### PPI Network Analysis and Identification of Key Genes

**Figure 4:**
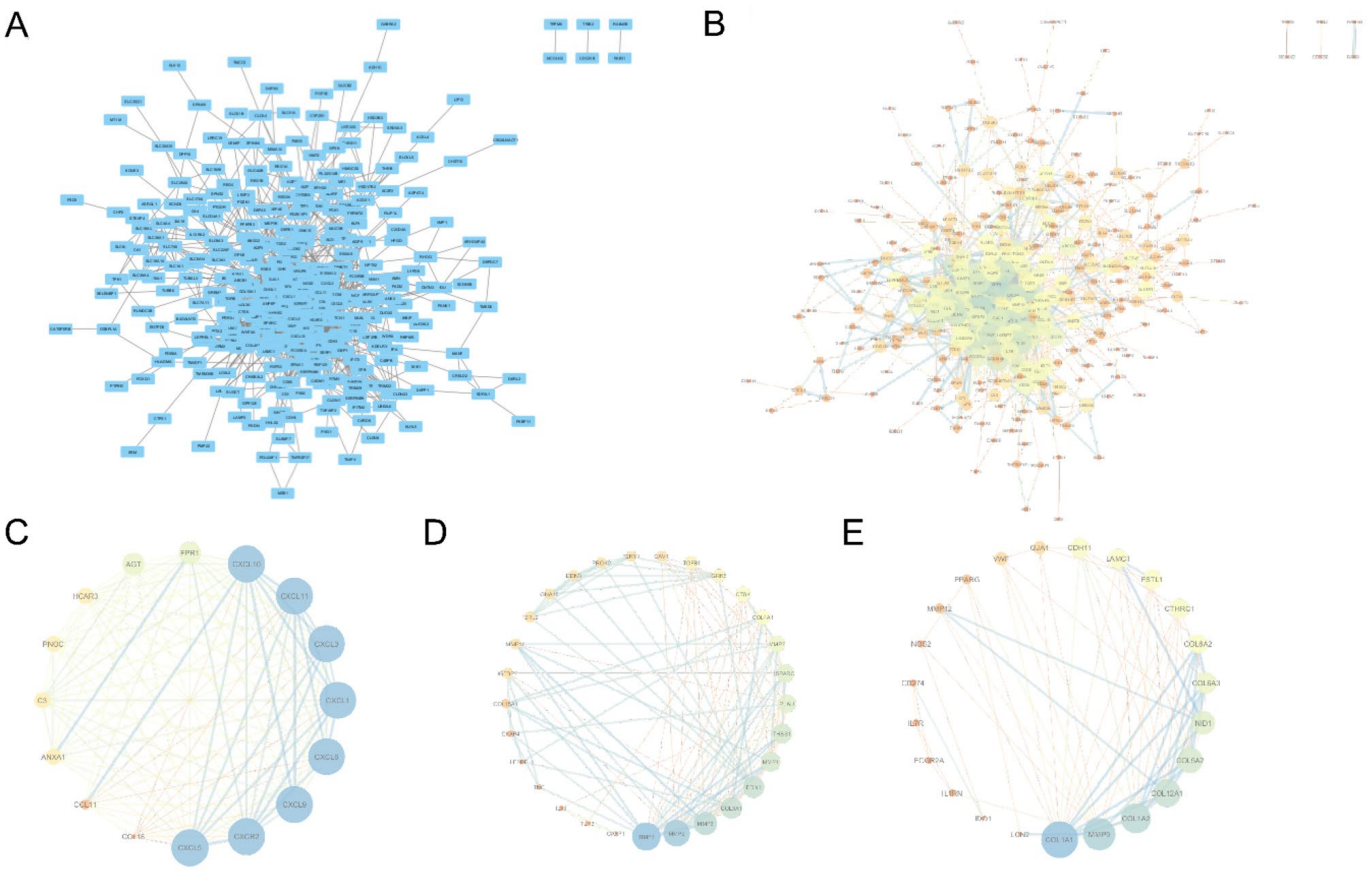
Protein–protein interaction (PPI) network analysis. (**A**) PPI network obtained from STRING database; (**B**) network analysis and visualization using Network Analyzer in Cytoscape; (**C**–**E**) the three highest rated word networks identified with the MCODE plugin, which were considered as the three more important functional modules.

We placed the 402 DEGs into the STRING database to obtain their PPI networks (Fig. 4A), and the PPI networks were placed into Cytoscape to identify and visualize important genes with strong interactions with other genes (Fig. 4B). The MCODE plug-in was used to identify the three sub-networks with the highest scores (Fig. 4C–E) (tableS2-Supplement 1).

### Functional enrichment analysis

**Figure 5:**
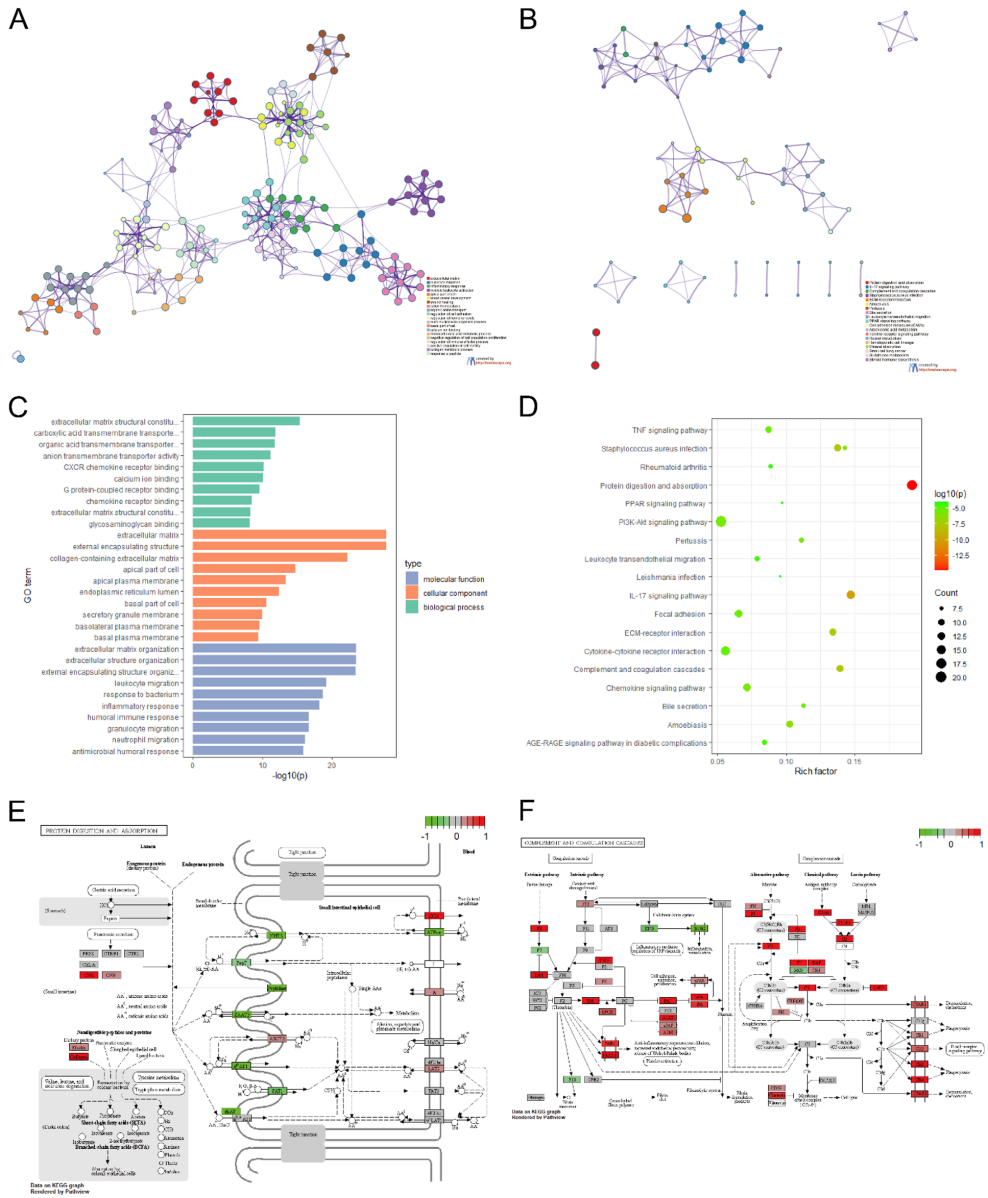
Functional enrichment analysis of differentially expressed genes. (**A**) Network diagram of top 20 GO enrichment functions, with cluster IDs to indicate color, each node is an enriched term; (**B**) network diagram of top 20 KEGG enrichment functions, with cluster IDs to indicate color; (**C**) barplot of GO enrichment functions, the length of the function bar is shown by *p* value; (**D**) dotplot of top 20 KEGG enrichment results; (**E**) pathway map of Protein digestion and absorption; (**F**) pathway map of Complement and coagulation cascades.

We first performed a functional enrichment analysis of DEGs using Metascape to screen for function at p < 0.01, a minimum count of 3, and an enrichment factor > 1. 5. The DEGs were mainly associated with extracellular matrix organization, inflammatory response, humoral immune response, apical part of cell, external encapsulating structure, carboxylic acid transmembrane transporter activity, protein digestion and absorption, ECM-receptor interaction, complement and coagulation cascades, and PI3K-Akt signaling pathway (Fig. 5) (tableS3-Supplement 2). The detailed enrichment results are shown in Supplement 3.

### GSEA functional enrichment analysis

**Figure 6:**
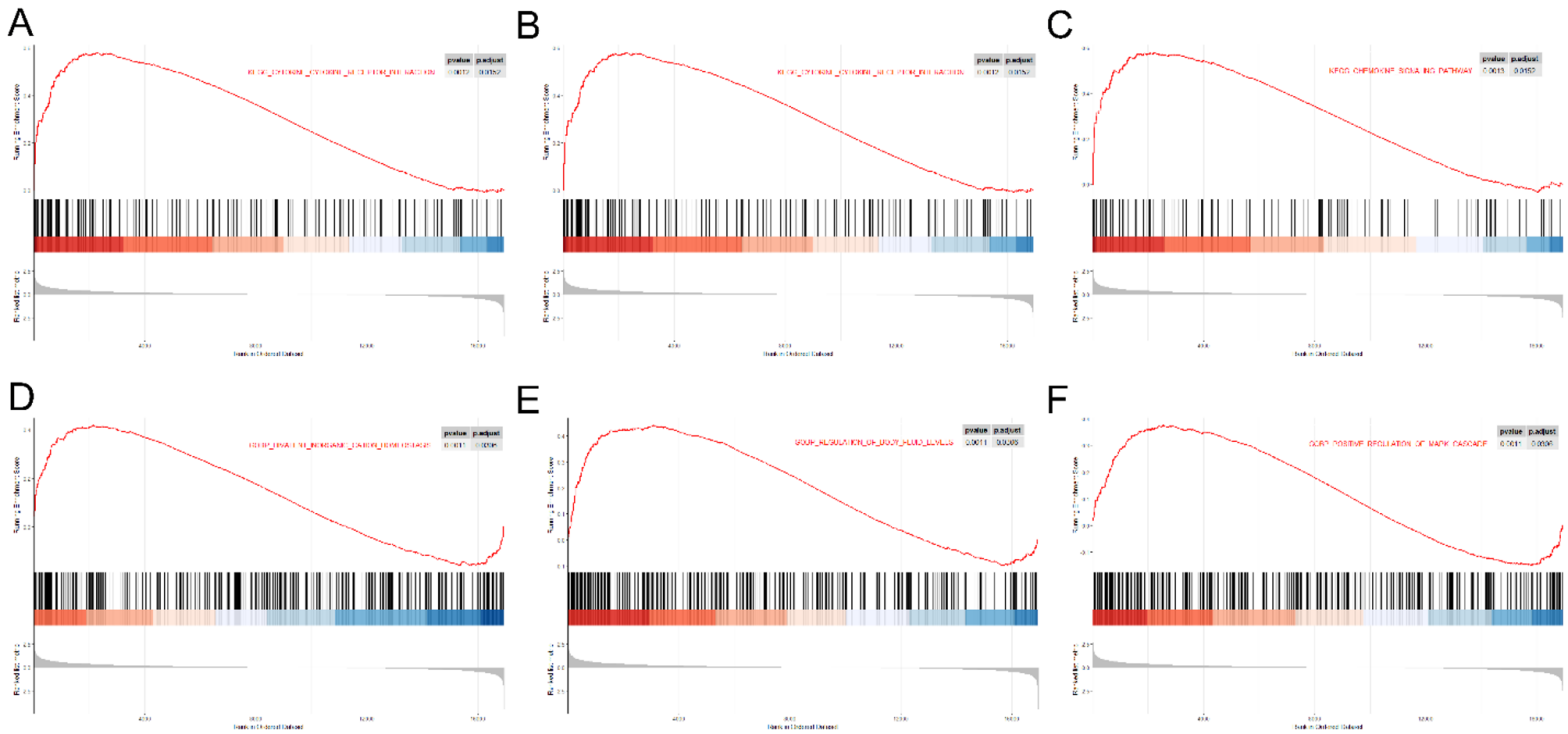
GSEA function enrichment analysis, (**A**–**C**) the top 3 GSEA enrich plots of enriched KEGG.D-F. The top 3 GSEA enrich plots of enriched GO. (**A**) KEGG_cytokine cytokine receptor interaction; (**B**) KEGG_focal adhesion; (**C**) KEGG_ chemokine signaling pathway. (**D**) GOBP_divalent inorganic cation homeostasis; (**E**) GOBP_regulation of body fluid levels; F. GOBP_ positive regulation of MAPK cascade.

We first downloaded the gene sets "c2.cp.kegg.v7.4.symbols.gmt" and "c5.go.bp.v7.4.symbols.gmt." The GSEA function in the clusterProfiler package was used to enrich the GSE38713 expression profile with "c2.cp.kegg.v7.4.symbols.gmt" and "c5.go.bp.v7.4.symbols.gmt" as reference gene sets^30^ (these two are more commonly used in functional enrichment). We used *p* value < 0.05 as the threshold to screen for differential functions. The results of KEGG and GO enrichments are shown in Fig. 6 (Table 4-Supplement 4) The main enrichments in GO BP were: Divalent inorganic cation homeostasis, regulation of body fluid levels, positive regulation of MAPK cascade. The main enrichments in KEGG were: cytokine receptor interaction, focal adhesion, chemokine signaling pathway, among others. The detailed enrichment results are shown in Supplement 5.

### Random Forest and LASSO Identification for Diagnostic Markers

**Figure 7:**
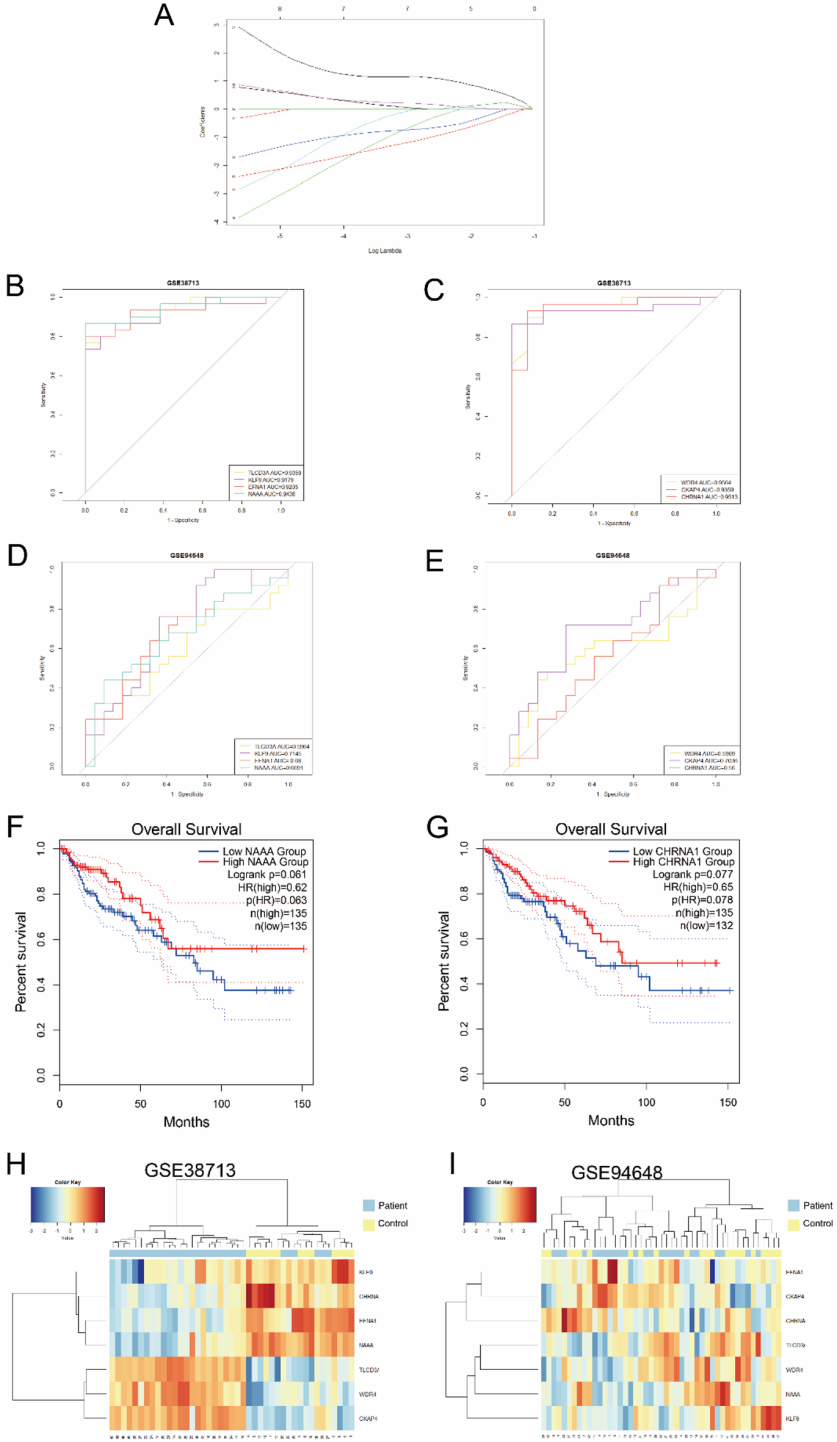
Identification, validation and prognostic analysis of diagnostic markers. (**A**) LASSO logistic regression algorithm for screening diagnostic markers; (**B**–**E**) ROC curves of diagnostic markers in GSE38713 and GSE94648; (**F**,**G**) GEPIA2 database showing the impact of NAAA and CHRNA1 on survival prognosis of UC-associated colorectal cancer; (**H**,**I**) Hierarchical clustering of diagnostic markers for GSE38713 and GSE94648. Normal samples are in yellow and disease samples are in blue.

A total of 402 DEGs were obtained from the gene expression matrix, and these 402 DEGs were used to construct a random forest. The top 2/3 nodes with the best classification effect were selected (keeping the largest MeanDecreaseGini and removing the top 1/3 nodes with small ones), and the poorly classified nodes were removed. We screened a total of six rounds and obtained 54 important genes. We then used the LASSO regression algorithm to identify seven diagnostic markers associated with UC, namely TLCD3A, KLF9, EFNA1, NAAA,WDR4, CKAP4, and CHRNA1, from the important genes obtained (Fig. 7A). The single gene ROC analysis using the expression values of the seven diagnostic markers in GSE38713 and GSE94648 revealed that the AUC values of all diagnostic markers in GSE38713 were greater than 0.9 (Fig. 7B,C). Good diagnostic values were also demonstrated in GSE94648 (Fig. 7D,E), with most genes having AUC values above 0.65. We then performed a hierarchical clustering analysis using these seven genes in GSE38713 and GSE94648 (Fig. 7H,I); the samples in both datasets were clustered into two categories, with one category clustering most of the normal samples and one category clustering most of the disease samples. We then inserted these seven genes into GEPIA2 and found that NAAA and CHRNA1 had a significant effect on the survival prognosis of UC-associated CRC (Fig. 7F,G).

### Immune Cell Infiltration Analysis and its Correlation with Diagnostic Markers

**Figure 8:**
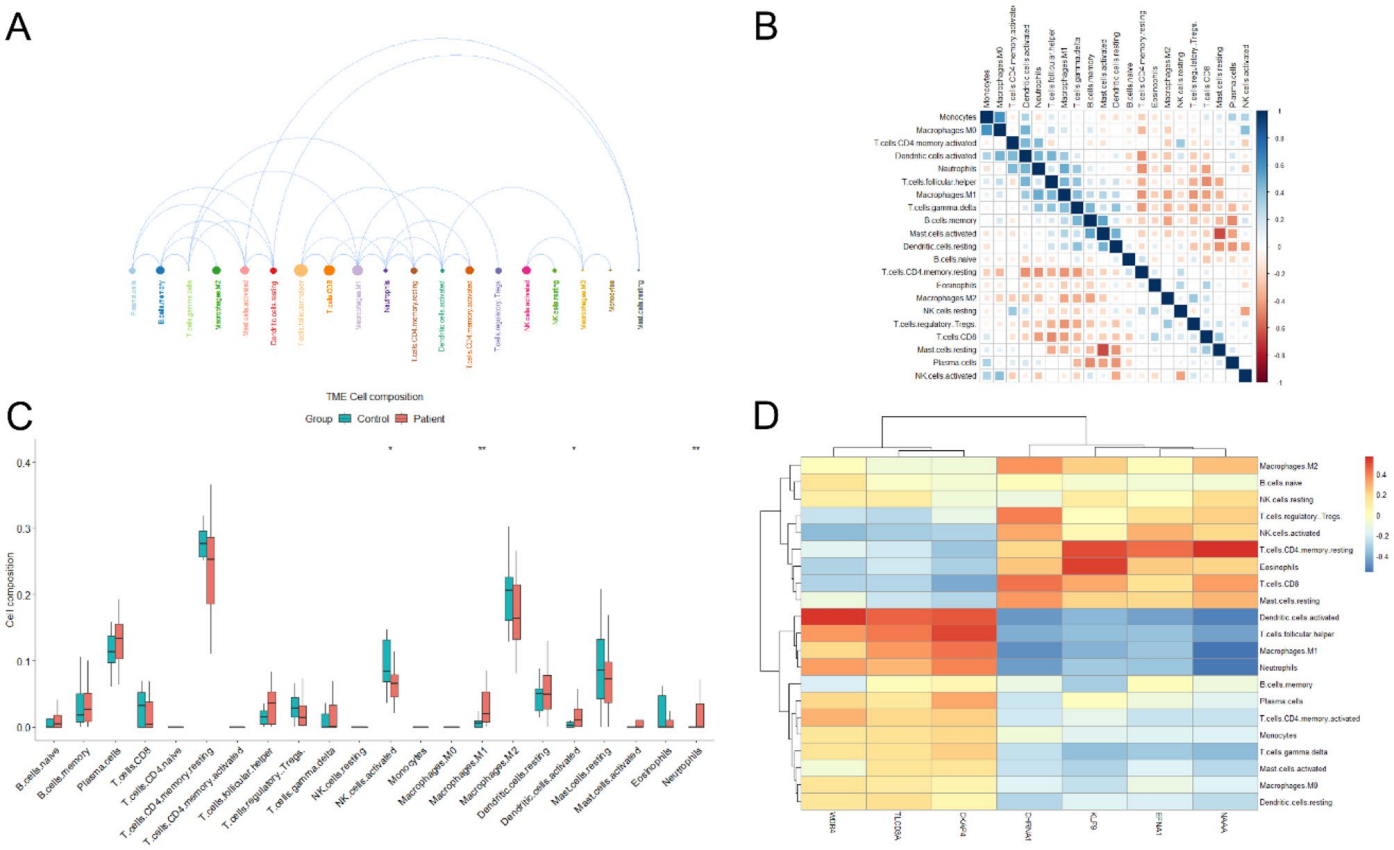
Visualization of immune cell infiltration and its correlation analysis with diagnostic markers. (**A**) Interaction plot of 22 immune cell infiltrations; circle size represents the strength of interactions with other immune cells, the larger the circle, the stronger the interactions with other immune cells. (**B**) Correlation heat map of 22 immune cell infiltrations; blue indicates positive correlation, red indicates negative correlation, the darker the color, the stronger the correlation. (**C**) Box line plot of 22 immune cell infiltrations, red represents UC group, blue represents control group. Box line plot of the proportion of 22 immune cell infiltrates; red represents UC group, blue represents Control group. (**D**) Correlation analysis of 22 immune cell infiltrates and diagnostic markers; red represents positive correlation, blue represents negative correlation. *p < 0.05; **p < 0.01.

The results of interactions of 22 immune cells (Fig. 8A) showed that follicular helper T cells had the strongest interactions with other immune cells, while resting mast cells, monocytes, macrophages, M0, and T cells had weaker interactions with other immune cells. The results of the correlation heat map of 22 immune cells showed (Fig. 8B) that T cells CD4 memory resting, dendritic cells activated, neutrophil, M1 macrophages, T cells gamma delta, and mast cells resting showed a significant negative correlation with follicular helper T cells, activated mast cell, dendritic cells resting; monocytes showed a significant negative correlation with monocytes and showed a significant positive correlation with macrophages M0; activated dendritic cells showed a significant positive correlation with macrophages M0, neutrophils, and T cells follicular helper cells, whereas showed a significant negative correlation with T cells CD4 memory resting. The box line plot of immune cell infiltration differences (Fig. 8C) showed that macrophages M1, activated dendritic cells, and neutrophil cells infiltrated relatively more, while NK cells activated cells infiltrated relatively less, compared with normal control samples. The results of the correlation analysis (Fig. 8D) showed that immune cells were clustered into two categories: macrophages M2, B cells naïve, NK cells resting, T cells regulatory, NK cells activated, T cells CD4 memory resting, eosinophils, T cells CD8, and resting mast cells showed a significant positive correlation with CKAP4, TLCD3A, WDR4 and a negative correlation with KLF9, EFNA1, NAA, and CHRNA1, while the rest of the immune cells showed the opposite trend.

## Discussion

Ulcerative colitis (UC) is a refractory disease characterized by a long duration, recurrence, and difficulty in healing^37^. The exact pathogenesis of this disease remains unknown. However, understanding the pathology of UC and underlying molecular mechanisms is essential for its clinical diagnosis and treatment. The use of efficient genome-wide gene expression microarray data and bioinformatics analysis can help us understand the molecular mechanisms of disease onset and progression, and is necessary for the identification of potential diagnostic biomarkers. To date, relevant reports have been published in terms of immune infiltration. Xiu et al.^9^ by raw letter analysis has predicted central genes, namely CDC42, POLR2A, RAC1, PIK3R1, MAPK1, and SRC, that have important roles in the pathological differences between children and adults with UC as well as immune cells, namely B cells, T cells, monocytes, macrophages, and mast cells, which may be potential biomarkers for the diagnosis and treatment of UC. Xue et al.^10^ showed that DPP10, S100P, AMPD1, and ASS1 may serve as diagnostic biomarkers for UC and that differentially infiltrating immune cells may help indicate the progression of UC. Zhu et al.^38^ revealed that immunity and infection are the two most important factors in the pathogenesis of UC; in this study, we used microarray-based bioinformatics analysis to explore the gene expression profile and pathogenesis of UC. To avoid a high false positive rate and one-sided results, we selected two gene microarray datasets (GSE38713 and GSE94648) for comprehensive analysis. We screened differential genes by performing GO, KEGG, GESA, and PPI analysis; used random forest and LASSO regression to screen for diagnostic markers; and used CIBERSORT to screen for immune cells associated with UC.

We used the dataset GSE38713 as the training set and GSE94648 as the validation set, and identified 402 DEGs, 242 upregulated genes and 160 downregulated genes in the dataset GSE38713. PPI network analysis of the obtained differential genes yielded three important sub-networks, and we suggest that these three modules may play a special role in the pathogenesis of UC. The largest of these subnetworks is dominated by the chemokine family, including CXCL1, CXCL3, CXCL5, CXCL6, CXCL9, CXCL10, CXCL11, and the chemokine receptor CXCR2, a large class of peptides that play a key role in the regulation of inflammation^39^. Chemokines are classified into the four families C, CC, CXC, and CX3C, according to the number and arrangement of their N-terminal cysteine residues; the CC subfamily mainly recruits lymphocytes and dendritic cells, and the CXC subfamily mainly recruits neutrophils and monocytes. Chemokines can chemotacticize leukocytes to participate in immune and inflammatory responses^40,41^. Blocking chemokines or their receptors significantly reduced intestinal inflammation and mucosal damage in animals with UC, suggesting that chemokines play a key role in the pathogenesis of UC^42^. CXCL1 upregulates and recruits circulating white blood cells, allowing the inflammation cycle to continue. Relevant studies have shown that CXCL1 is significantly upregulated in the colon tissues of UC patients and rats, and may be a potential biomarker of UC tissue biopsy^39,43,44^. CXCL9 is a small cytokine called MIG, which serves as a chemoattractant for T cells induced by IFN-γ. Serum CXCL9 level is related to UC disease activity, and its expression increases in patients with UC and UC mouse models. Thus, it may become a marker of patient response to treatment^45,46^.Animal studies have reported that CXCL10 inhibits the proliferation of intestinal epithelial cells and regulates the proliferation of crypt cells during acute colitis in mice, making it a new therapeutic target for inflammatory bowel disease^47^. CXCR2 plays a key role in the pathogenesis of UC by regulating the immune response of neutrophils. Blocking CXCR2 can improve DSS-induced intestinal mucosal inflammation in mice, and CXCR2 can be used as a new target for UC drug therapy^48,49^.

The second subnetwork is dominated by the matrix metalloproteinase family, including MMP1, MMP2, MMP3, MMP7, MMP10, and MMP9( in the third subnetwork), and its associated matrix metalloprotein inhibitor TIMP1. UC lesions are strongly associated with excessive ECM degradation. Matrix metalloproteinases (MMPs) can degrade proteins in the (ECM) and have an important role in ulceration and tissue remodeling^50,51^. Increased levels of MMP1 and MMP2 have been shown to play a major role in degradation of the intestinal matrix^52^. Both protein and mRNA levels of MMP-2 and MMP9 were significantly increased in inflammatory bowel disease tissues, with the highest expression levels in severely inflamed tissues^53^. MMP9 activates myosin light chain kinase (MLCK) to impair colonic epithelial permeability and plays an important role in enhancing the degree of inflammation^54^. Upregulation of intestinal mucosal MMP9 expression in patients with UC correlates with severity, and its increase suggests severe mucosal damage in active UC^55^. MMP3 is produced by mesenchymal cells and immune cells in the lamina propria. Some studies have reported serum MMP3 as a potential biomarker for endoscopic and histological activity of UC^56^.The primary cellular source of MMP7 in patients with active UC is most likely leukocytes^57^. In UC, MMP7 was found to be expressed in epithelial cells at the ulcer margin, developmental abnormalities, and transformed cells, and its expression was correlated with the degree of endoscopic inflammation^58^. Tissue inhibitors of metalloproteinases (TIMPs) are natural inhibitors of MMPs, which in turn are a group of secreted glycoproteins that are widely found in tissues and body^59^. TIMP-1 is one of the four isoforms of TIMPs and mainly inhibits MMP1, MMP3, and MMP9 activities^60^. MMP-1 mRNA, TIMP-1 mRNA, and MMP-1 mRNA/TIMP-1 mRNA ratio in the diseased colonic mucosa of patients with UC can be used as biomarkers to determine the severity of the patients’ clinical symptoms^61^. As shown by GO and KEGG analysis of DEGs, in the BP annotation of GO, extracellular matrix organization, inflammatory response, humoral immune response; in the CC annotation of GO, extracellular matrix; and in the MF annotation of GO, extracellular matrix structural components and CXCR chemokine receptor binding were significantly associated with the occurrence and development of UC.

Inflammation is an important pathological response in UC pathogenesis. Pathologically, inflammation occurs in the lining of the colon and rectum, and is manifested by infiltration of neutrophils, macrophages, lymphocytes, and mast cells. Intestinal inflammation further destroys the mucosa and submucosa, eventually leading to intestinal ulceration^62^. ECM constitutes the framework structure for cell survival and affects the basic life activities of cells, and its components are in dynamic balance; imbalance will cause various pathological changes, such as ulcer formation^63^. Degradation of ECM is involved in the pathological development of UC, and quantitative changes in its component composition and structure play an important role in the pathogenesis of inflammatory bowel disease^64,65^.The pathogenesis of UC is related to immunological abnormalities, and various factors involved in the immune system may be directly or indirectly associated with UC^66^. Generally, immune responses are divided into cellular and humoral immune responses according to different effectors^67^. Chemokine receptors are a class of G protein-coupled receptors that play an important role in inflammatory cells of injured or infected organs. Chemokine receptor expression is upregulated during the active phase of UC^68^.In KEGG, among the important pathways of enrichment, complement and coagulation cascades, chemokine signaling pathway, IL-17 signaling pathway, and ECM-receptor interaction were significantly enriched, which is consistent with previous studies^44,69,70^. In our study, the PI3K-Akt signaling pathway was enriched with the highest number of genes. The PI3K/AKT signaling pathway is closely related to the regulation of cytokines and plays an important role in the process of intestinal inflammation, which can lead to dysregulation of the inflammatory response^71^. In UC, UC-associated colon carcinogenesis can be induced by upregulating the PI3K/Akt signaling pathway. When this pathway is blocked, the activation of nuclear factor kappa B (NF-κB) is inhibited, and cytokine release is reduced^72,73^.

To investigate the biological functions of the DEGs associated with UC, GSEA was performed. The top3 enriched GO-BPs were divalent inorganic cation homeostasis, regulation of body fluid levels and positive regulation of MAPK cascade, respectively. Cytokine cytokine receptor interaction, focal adhesion, and chemokine signaling pathways were the first three significantly enriched KEGG pathways. The enrichment of cytokine-cytokine receptor interaction and chemokine signaling pathway were consistent with the results of a previous study^44^.

The seven diagnostic markers screened using random forest screen and LASSO regression were TLCD3A, KLF9, EFNA1, NAAA, WDR4, CKAP4, and CHRNA1. Among them, only NAAA has a small number of reports in the literature on its association with UC. A decreased number of NAAA-positive immune cells detected in active UC has been reported^74^. NAAA-targeted drugs have potential value in the treatment of human inflammatory diseases^75^.

From the analysis of immune cell infiltration assessment and its correlation with diagnostic markers, it is clear that among 22 immune cells, follicular helper T cells have the strongest interactions with other immune cells. The immune balance between follicular helper T (TFH) cells and follicular regulatory T (TFR) cells is important for regulating B-cell responses, and changes in the ratio between the two, shifts the balance from immune tolerance to an immune response state, leading to B-cell immune dysregulation and the pathogenesis of UC^76^. It has been shown that increased inducible co-stimulation positive (ICOS) + programmed cell death 1 positive (PD-1) + TFH cells are associated with B-cell activation in UC pathogenesis and may act as potential biomarkers for UC disease monitoring^77^. In the differential analysis of immune cell infiltration, macrophages M1 (*p* < 0.01), activated dendritic cells (*p* < 0.05), and neutrophils (*p* < 0.01) were highly expressed in UC tissues compared to normal tissues. Neutrophils are predominantly present in areas of colonic mucosal injury in patients with UC, forming their characteristic crypt abscesses, producing reactive oxygen species and releasing serine proteases, matrix metalloproteinases, and myeloperoxidase (MPO)^78,79^. Macrophages are the main effector cells of the innate immune system and play various roles, such as phagocytosis of pathogens, secretion of cytokines and chemokines, and antigen presentation. They are divided into M1 macrophages (classically called activated macrophages) and M2 macrophages (alternatively called activated macrophages). M1 macrophages are more frequently present in the lamina propria of the colonic mucosa of UC and produce large amounts of pro-inflammatory cytokines. Their abnormal activation is an important part of UC development^80–83^. Activated and mature dendritic cells may play a role in inducing an immune response that is exacerbated in UC, and their increased function may be related to the inflammatory mucosal environment found in patients with UC^84^.

Our study has certain limitations. First, the sample size was increased to further clarify the diagnostic accuracy of the core genes associated with UC. Second, the results of the two microarrays as training and validation sets, respectively, may be more one-sided, and external validation is needed to avoid false-positive rates; third, further ex vivo experiments are needed to validate the potential mechanisms by which the obtained important gene modules act on UC.

## Conclusions

In conclusion, the aim of this study was to explore the molecular mechanisms underlying UC pathogenesis through bioinformatics analysis. We aimed to identify the relevant biological functions and signaling pathways involved in the development of UC. We identified three functional modules that play an important role in the development of UC occurrence through PPI network analysis. Seven genes were identified by LASSO regression as potential diagnostic markers for UC, and the area under the curve for most genes was greater than 0.65 was estimated by ROC curve analysis and further by GEPIA2 analysis. NAAA and CHRNA1 were predicted to also serve as prognostic markers for survival in UC-associated CRC. The relationship between immune cell infiltration and seven diagnostic markers was also analyzed by CIBERSORT, and positive relationships were obtained between Macrophages M2, NK cells resting, T cell regulatory and CKAP4, TLCD3A, WDR4. In contrast, these immune cells were inversely correlated with KLF9, EFNA1, NAA, and CHRNA1; however, further experiments are required to validate the current findings.

## Supplementary Information

Supplementary Information 1.

Supplementary Information 2.

Supplementary Information 3.

Supplementary Information 4.

Supplementary Information 5.

## Data Availability

Our data can be found in the Gene Expression Omnibus (GEO, https://www.ncbi.nlm.nih.gov/geo/, GSE38713 and GSE94648) database.
