# Supplementary material for: Identification of diagnostic biomarks and immune cell infiltration in ulcerative colitis
Source: Sci Rep. 2023 Apr 13;13:6081. doi: 10.1038/s41598-023-33388-5 (PMC10102327; doi:10.1038/s41598-023-33388-5)
Supplement: Supplementary file 1 — Supplementary Information 1. [file 41598_2023_33388_MOESM1_ESM.pdf]

Table 2. Top3 Modules identified by MCODE plugin

	MCODE scores	Information	Upregulated DEGS	Downregulated DEGS
Module 1	14.667	16 nodes; 110 edges	CXCR2, C3, CCL18, CXCL11, CXCL6, CXCL10, AGT, CCL11, PNOC, ANXA1, CXCL5, FPR1, CXCL3, HCAR3, CXCL1, CXCL9	
Module 2	7.857	29 nodes; 110 edges	CTSK, GRK5, COL3A1, MMP3, CASP1, PLAU, MMP10, SPARC, MMP1, CAV1, MMP2, TIMP1, IGFBP7, F2RL2, COL4A1, COL15A1, MMP7, TNC, PROK2, CKAP4, IL33, TGFBI, LEPREL1, GNA15	EDN1, THBS1, EDN3, TLR3, P2RY1
Module 3	6.818	23 nodes; 75 edges	FCGR2A, IL1RN, CD274, COL6A2, NID1, MMP12, IDO1, COL1A2, COL5A2, LAMC1, CDH11, COL6A3, COL1A1, CTHRC1, LCN2, COL12A1, NOS2, FSTL1, VWF, MMP9, IL7R, GJA1	PPARG
