# Supplementary material for: Identification of diagnostic biomarks and immune cell infiltration in ulcerative colitis
Source: Sci Rep. 2023 Apr 13;13:6081. doi: 10.1038/s41598-023-33388-5 (PMC10102327; doi:10.1038/s41598-023-33388-5)
Supplement: Supplementary file 2 — Supplementary Information 2. [file 41598_2023_33388_MOESM2_ESM.pdf]

Table 3. Top enrichment function of GO/KEGG

| **Category** | **GO** | **Description** | **count** | **LogP** |
|---|---|---|---|---|
| GO Biological Processes | GO:0030198 | extracellular matrix organization | 42 | -23.46980197 |
| GO Biological Processes | GO:0043062 | extracellular structure organization | 42 | -23.42662795 |
| GO Biological Processes | GO:0045229 | external encapsulating structure organization | 42 | -23.34066897 |
| GO Biological Processes | GO:0050900 | leukocyte migration | 42 | -19.18499513 |
| GO Biological Processes | GO:0009617 | response to bacterium | 49 | -18.67851633 |
| GO Biological Processes | GO:0006954 | inflammatory response | 50 | -18.21499759 |
| GO Biological Processes | GO:0006959 | humoral immune response | 34 | -16.63810234 |
| GO Biological Processes | GO:0097530 | granulocyte migration | 23 | -16.63794885 |
| GO Biological Processes | GO:1990266 | neutrophil migration | 21 | -16.09005675 |
| GO Biological Processes | GO:0019730 | antimicrobial humoral response | 22 | -15.90671083 |
| GO Cellular Components | GO:0031012 | extracellular matrix | 54 | -27.83764385 |
| GO Cellular Components | GO:0030312 | external encapsulating structure | 54 | -27.79957381 |

| | | | | |
|---|---|---|---|---|
| GO Cellular Components | GO:0062023 | collagen-containing extracellular matrix | 42 | -22.26810209 |
| GO Cellular Components | GO:0045177 | apical part of cell | 33 | -14.69303631 |
| GO Cellular Components | GO:0016324 | apical plasma membrane | 29 | -13.355918 |
| GO Cellular Components | GO:0005788 | endoplasmic reticulum lumen | 26 | -12.36489181 |
| GO Cellular Components | GO:0045178 | basal part of cell | 22 | -10.51340097 |
| GO Cellular Components | GO:0030667 | secretory granule membrane | 23 | -9.955872464 |
| GO Cellular Components | GO:0016323 | basolateral plasma membrane | 19 | -9.549588736 |
| GO Cellular Components | GO:0009925 | basal plasma membrane | 20 | -9.429519716 |
| GO Molecular Functions | GO:0005201 | extracellular matrix structural constituent | 23 | -15.33881909 |
| GO Molecular Functions | GO:0046943 | carboxylic acid transmembrane transporter activity | 19 | -11.83394444 |
| GO Molecular Functions | GO:0005342 | organic acid transmembrane transporter activity | 19 | -11.78432514 |
| GO Molecular Functions | GO:0008509 | anion transmembrane transporter activity | 30 | -11.16699963 |
| GO Molecular Functions | GO:0045236 | CXCR chemokine receptor binding | 8 | -10.20678133 |
| GO Molecular Functions | GO:0005509 | calcium ion binding | 36 | -10.1536751 |

| | | | | |
|---|---|---|---|---|
| GO Molecular Functions | GO:0001664 | G protein-coupled receptor binding | 22 | -9.626149143 |
| GO Molecular Functions | GO:0042379 | chemokine receptor binding | 11 | -8.420130124 |
| GO Molecular Functions | GO:0030020 | extracellular matrix structural constituent conferring tensile strength | 9 | -8.278491748 |
| GO Molecular Functions | GO:0005539 | glycosaminoglycan binding | 18 | -8.172074945 |
| KEGG Pathway | hsa04974 | Protein digestion and absorption | 18 | -14.85800725 |
| KEGG Pathway | hsa04657 | IL-17 signaling pathway | 14 | -10.07868049 |
| KEGG Pathway | hsa04610 | Complement and coagulation cascades | 11 | -7.780515126 |
| KEGG Pathway | hsa05150 | Staphylococcus aureus infection | 11 | -7.721815054 |
| KEGG Pathway | hsa04512 | ECM-receptor interaction | 11 | -7.606953049 |
| KEGG Pathway | hsa05146 | Amoebiasis | 11 | -6.40038318 |
| KEGG Pathway | hsa04151 | PI3K-Akt signaling pathway | 20 | -6.159939434 |
| KEGG Pathway | ko05150 | Staphylococcus aureus infection | 8 | -5.901202089 |
| KEGG Pathway | hsa05133 | Pertussis | 9 | -5.624986197 |
| KEGG Pathway | hsa04062 | Chemokine signaling pathway | 13 | -5.623230368 |
