# Supplementary material for: Identification of diagnostic biomarks and immune cell infiltration in ulcerative colitis
Source: Sci Rep. 2023 Apr 13;13:6081. doi: 10.1038/s41598-023-33388-5 (PMC10102327; doi:10.1038/s41598-023-33388-5)
Supplement: Supplementary file 3 — Supplementary Information 3. [file 41598_2023_33388_MOESM3_ESM.pdf]

_MEMBER_MyList _LogP_MyList GO _PATTERN_ _RANK_ GiniIndex Category CategoryID Description PARENT_GO LogP Enrichment Z-score #TotalGeneInLibrary #GeneInGO #GeneInHitList #GeneInGOAndHitList %InGO STDV %InGO GeneID Hits Log(q-value) EvidenceCutoff GROUP_ID FirstInGroupByEnrichment FirstInGroupByLogP BestLogPInGroup BestEnrichmentInGroup

1 -27.83764385 GO:0031012 M1 1 0 GO Cellular Components 20 extracellular matrix -27.83764385 6.683666473 16.43770054 28162 566 402 54 13.43283582 1.700777188 12|183|301|308|1116|1277|1278|1281|1282|1290|1292|1293|1303|1306|1755|2263|3371|3490|3915|4017|4256|4312|4313|4314|4316|4318|4319|4321|4811|5266|5270|5271|5272|6279|6280|6678|7045|7052|7057|7076|7098|7450|7474|7837|7980|9510|23452|26585|55214|56999|83716|115908|284217|727897 SERPINA3|AGT|ANXA1|ANXA5|CHI3L1|COL1A1|COL1A2|COL3A1|COL4A1|COL5A2|COL6A2|COL6A3|COL12A1|COL15A1|DMBT1|FGFR2|TNC|IGFBP7|LAMC1|LOXL2|MGP|MMP1|MMP2|MMP3|MMP7|MMP9|MMP10|MMP12|NID1|PI3|SERPINE2|SERPINB8|SERPINB9|S100A8|S100A9|SPARC|TGFBI|TGM2|THBS1|TIMP1|TLR3|VWF|WNT5A|PXDN|TFPI2|ADAMTS1|ANGPTL2|GREM1|P3H2|ADAMTS9|CRISPLD2|CTHRC1|LAMA1|MUC5B -23.75259117 0 1 1 1 -27.83764385 9.477992391

1 -27.79957381 GO:0030312 M1 1 0 GO Cellular Components 20 external encapsulating structure -27.79957381 6.671878702 16.4183911 28162 567 402 54 13.43283582 1.700777188 12|183|301|308|1116|1277|1278|1281|1282|1290|1292|1293|1303|1306|1755|2263|3371|3490|3915|4017|4256|4312|4313|4314|4316|4318|4319|4321|4811|5266|5270|5271|5272|6279|6280|6678|7045|7052|7057|7076|7098|7450|7474|7837|7980|9510|23452|26585|55214|56999|83716|115908|284217|727897 SERPINA3|AGT|ANXA1|ANXA5|CHI3L1|COL1A1|COL1A2|COL3A1|COL4A1|COL5A2|COL6A2|COL6A3|COL12A1|COL15A1|DMBT1|FGFR2|TNC|IGFBP7|LAMC1|LOXL2|MGP|MMP1|MMP2|MMP3|MMP7|MMP9|MMP10|MMP12|NID1|PI3|SERPINE2|SERPINB8|SERPINB9|S100A8|S100A9|SPARC|TGFBI|TGM2|THBS1|TIMP1|TLR3|VWF|WNT5A|PXDN|TFPI2|ADAMTS1|ANGPTL2|GREM1|P3H2|ADAMTS9|CRISPLD2|CTHRC1|LAMA1|MUC5B -23.75259117 0 1 0 0 -27.83764385 9.477992391

1 -23.46980197 GO:0030198 M1 1 0 GO Biological Processes 19 extracellular matrix organization -23.46980197 7.448856981 15.53259953 28162 395 402 42 10.44776119 1.525586426 183|682|857|1277|1278|1281|1282|1290|1292|1293|1303|1306|1513|3371|3915|4017|4312|4313|4314|4316|4318|4319|4321|4811|5175|5268|6678|6696|7045|7057|7076|7450|7837|9510|11005|26585|28984|55151|55790|56999|83716|284217 AGT|BSG|CAV1|COL1A1|COL1A2|COL3A1|COL4A1|COL5A2|COL6A2|COL6A3|COL12A1|COL15A1|CTSK|TNC|LAMC1|LOXL2|MMP1|MMP2|MMP3|MMP7|MMP9|MMP10|MMP12|NID1|PECAM1|SERPINB5|SPARC|SPP1|TGFBI|THBS1|TIMP1|VWF|PXDN|ADAMTS1|SPINK5|GREM1|RGCC|TMEM38B|CSGALNACT1|ADAMTS9|CRISPLD2|LAMA1 -19.69162633 0 1 0 0 -27.83764385 9.477992391

1 -23.42662795 GO:0043062 M1 1 0 GO Biological Processes 19 extracellular structure organization -23.42662795 7.430046736 15.50716453 28162 396 402 42

10.44776119 1.525586426
183|682|857|1277|1278|1281|1282|1290|1292|1293|1303|1306|1513|3371|3915|4017|4312|4313|4314|4316|4318|4319|4321|4811|5175|5268|6678|6696|7045|7057|7076|7450|7837|9510|11005|26585|28984|55151|55790|56999|83716|284217
AGT|BSG|CAV1|COL1A1|COL1A2|COL3A1|COL4A1|COL5A2|COL6A2|COL6A3|COL12A1|COL15A1|CTSK|TNC|LAMC1|LOXL2|MMP1|MMP2|MMP3|MMP7|MMP9|MMP10|MMP12|NID1|PECAM1|SERPINB5|SPARC|SPP1|TGFBI|THBS1|TIMP1|VWF|PXDN|ADAMTS1|SPINK5|GREM1|RGCC|TMEM38B|CSGALNACT1|ADAMTS9|CRISPLD2|LAMA1-19.69162633 0 1 0 0
-27.83764385 9.477992391
1 -23.34066897 GO:0045229 M1 1 0 GO Biological Processes 19 external encapsulating structure organization -23.34066897 7.392709818 15.4565599 28162
398 402 42 10.44776119 1.525586426
183|682|857|1277|1278|1281|1282|1290|1292|1293|1303|1306|1513|3371|3915|4017|4312|4313|4314|4316|4318|4319|4321|4811|5175|5268|6678|6696|7045|7057|7076|7450|7837|9510|11005|26585|28984|55151|55790|56999|83716|284217
AGT|BSG|CAV1|COL1A1|COL1A2|COL3A1|COL4A1|COL5A2|COL6A2|COL6A3|COL12A1|COL15A1|CTSK|TNC|LAMC1|LOXL2|MMP1|MMP2|MMP3|MMP7|MMP9|MMP10|MMP12|NID1|PECAM1|SERPINB5|SPARC|SPP1|TGFBI|THBS1|TIMP1|VWF|PXDN|ADAMTS1|SPINK5|GREM1|RGCC|TMEM38B|CSGALNACT1|ADAMTS9|CRISPLD2|LAMA1-19.69162633 0 1 0 0
-27.83764385 9.477992391
1 -22.26810209 GO:0062023 M1 1 0 GO Cellular Components20
collagen-containing extracellular matrix -22.26810209 6.939383272 14.82907425
28162 424 402 42 10.44776119 1.525586426
12|183|301|308|1277|1278|1281|1282|1290|1292|1293|1303|1306|2263|3371|3490|3915|4017|4256|4313|4318|4811|5270|5271|5272|6279|6280|6678|7045|7052|7057|7076|7450|7474|7837|9510|23452|26585|55214|56999|115908|284217
SERPINA3|AGT|ANXA1|ANXA5|COL1A1|COL1A2|COL3A1|COL4A1|COL5A2|COL6A2|COL6A3|COL12A1|COL15A1|FGFR2|TNC|IGFBP7|LAMC1|LOXL2|MGP|MMP2|MMP9|NID1|SERPINE2|SERPINB8|SERPINB9|S100A8|S100A9|SPARC|TGFBI|TGM2|THBS1|TIMP1|VWF|WNT5A|PXDN|ADAMTS1|ANGPTL2|GREM1|P3H2|ADAMTS9|CTHRC1|LAMA1 -18.6982407 0 1 0
0 -27.83764385 9.477992391
1 -15.33881909 GO:0005201 M1 1 0 GO Molecular Functions 21 extracellular matrix structural constituent -15.33881909 9.477992391 13.34221241 28162 170 402
23 5.721393035 1.158362377
1116|1277|1278|1281|1282|1290|1292|1293|1303|1306|3371|3490|3915|4256|4811|6678|7045|7057|7450|7837|7980|115908|284217
CHI3L1|COL1A1|COL1A2|COL3A1|COL4A1|COL5A2|COL6A2|COL6A3|COL12A1|COL15A1|TNC|IGFBP7|LAMC1|MGP|NID1|SPARC|TGFBI|THBS1|VWF|PXDN|TFPI2|CTHRC1|LAMA1
-12.19492643 0 1 0 0 -27.83764385 9.477992391
1 -12.36489181 GO:0005788 M1 1 0 GO Cellular Components20 endoplasmic reticulum lumen -12.36489181 5.952362371 10.48192586 28162 306 402 26
6.467661692 1.226708902
718|1277|1278|1281|1282|1290|1292|1293|1303|1306|1906|3371|3488|3490|3915|6696|7057|7076|7474|7873|10970|11167|23753|51237|55214|493869

C3|COL1A1|COL1A2|COL3A1|COL4A1|COL5A2|COL6A2|COL6A3|COL12A1|COL15A1|EDN1|TNC|IGFBP5|IGFBP7|LAMC1|SPP1|THBS1|TIMP1|WNT5A|MANF|CKAP4|FSTL1|SDF2L1|MZB1|P3H2|GPX8 -9.640128462 0 1 0 0 -27.83764385 9.477992391

1 -7.994684113 GO:0005604 M1 1 0 GO Cellular Components20 basement membrane -7.994684113 8.943156558 9.282792598 28162 94 402 12 2.985074627 0.848757934

1282|1306|3371|3915|4017|4811|6678|7045|7076|9510|55214|284217

COL4A1|COL15A1|TNC|LAMC1|LOXL2|NID1|SPARC|TGFBI|TIMP1|ADAMTS1|P3H2|LAMA1 -5.703576326 0 1 0 0 -27.83764385 9.477992391

1 -7.97708802 GO:0005198 M1 1 0 GO Molecular Functions 21 structural molecule activity -7.97708802 3.184305744 7.063055827 28162 704 402 32 7.960199005 1.350009335

288|1116|1277|1278|1281|1282|1290|1292|1293|1303|1306|3371|3490|3915|4256|4478|4811|5266|6678|7045|7057|7450|7837|7980|9073|9076|10562|79861|84617|115908|137075|284217

ANK3|CHI3L1|COL1A1|COL1A2|COL3A1|COL4A1|COL5A2|COL6A2|COL6A3|COL12A1|COL15A1|TNC|IGFBP7|LAMC1|MGP|MSN|NID1|PI3|SPARC|TGFBI|THBS1|VWF|PXDN|TFPI2|CLDN8|CLDN1|OLFM4|TUBAL3|TUBB6|CTHRC1|CLDN23|LAMA1 -5.689773222 0 1 0 0 -27.83764385 9.477992391

1 -19.18499513 GO:0050900 M1 1 0 GO Biological Processes 19 leukocyte migration -19.18499513 5.757922715 13.06170119 28162 511 402 42 10.44776119 1.525586426

240|301|682|857|1277|1278|1906|1908|2919|2921|3579|3627|4067|4283|4312|4318|4478|5175|6279|6280|6283|6356|6362|6372|6373|6374|6402|6566|7056|7057|7474|8140|10451|10562|11240|23657|26585|57126|83483|90865|124976|255231

ALOX5|ANXA1|BSG|CAV1|COL1A1|COL1A2|EDN1|EDN3|CXCL1|CXCL3|CXCR2|CXCL10|LYN|CXCL9|MMP1|MMP9|MSN|PECAM1|S100A8|S100A9|S100A12|CCL11|CCL18|CXCL6|CXCL11|CXCL5|SELL|SLC16A1|THBD|THBS1|WNT5A|SLC7A5|VAV3|OLFM4|PADI2|SLC7A11|GREM1|CD177|PLVAP|IL33|SPNS2|MCOLN2 -15.68208053 0 2 1 1 -19.18499513 31.13543394

1 -18.67851633 GO:0009617 M1 1 0 GO Biological Processes 19 response to bacterium -18.67851633 4.715221967 12.22174506 28162 728 402 49 12.18905473 1.631722541

718|722|725|834|857|1439|1604|1670|1671|1672|1755|1906|2263|2697|2919|2921|3248|3553|3575|3620|3627|3934|4067|4283|4843|5105|5266|5272|5320|6279|6280|6283|6372|6373|6374|6584|6678|7056|7098|7474|9076|10891|11005|11126|29126|79931|83998|116842|646627

C3|C4BPA|C4BPB|CASP1|CAV1|CSF2RB|CD55|DEFA5|DEFA6|DEFB1|DMBT1|EDN1|FGFR2|GJA1|CXCL1|CXCL3|HPGD|IL1B|IL7R|IDO1|CXCL10|LCN2|LYN|CXCL9|NOS2|PCK1|PI3|SERPINB9|PLA2G2A|S100A8|S100A9|S100A12|CXCL6|CXCL11|CXCL5|SLC22A5|SPARC|THBD|TLR3|WNT5A|CLDN1|PPARGC1A|SPINK5|CD160|CD274|TNIP3|REG4|LEAP2|LYPD8 -15.23359368 0 2 0 0 -19.18499513 31.13543394

1 -16.63810234 GO:0006959 M1 1 0 GO Biological Processes 19 humoral immune response -16.63810234 6.235237425 12.39736178 28162 382 402 34 8.457711443

1.387791708
240|629|716|718|722|725|1604|1670|1671|1672|1755|2919|2921|3426|3553|3627|3934|4283|5068|5266|5320|5450|5967|5968|6279|6280|6283|6372|6373|6374|11005|28984|116842|124976
ALOX5|CFB|C1S|C3|C4BPA|C4BPB|CD55|DEFA5|DEFA6|DEFB1|DMBT1|CXCL1|CXCL3|CFI|IL1B|CXCL10|LCN2|CXCL9|REG3A|PI3|PLA2G2A|POU2AF1|REG1A|REG1B|S100A8|S100A9|S100A12|CXCL6|CXCL11|CXCL5|SPINK5|RGCC|LEAP2|SPNS2 -13.3313289 0 2 0 0
-19.18499513 31.13543394
1 -16.63794885 GO:0097530 M1 1 0 GO Biological Processes 19 granulocyte migration -16.63794885 10.81381682 14.45368558 28162 149 402 23 5.721393035
1.158362377
301|682|1906|1908|2919|2921|3579|3627|4283|5175|6279|6280|6283|6356|6362|6372|6373|6374|7057|10451|10562|57126|255231
ANXA1|BSG|EDN1|EDN3|CXCL1|CXCL3|CXCR2|CXCL10|CXCL9|PECAM1|S100A8|S100A9|S100A12|CCL11|CCL18|CXCL6|CXCL11|CXCL5|THBS1|VAV3|OLFM4|CD177|MCOLN2
-13.3313289 0 2 0 0 -19.18499513 31.13543394
1 -16.09005675 GO:1990266 M1 1 0 GO Biological Processes 19 neutrophil migration -16.09005675 11.86410688 14.59011411 28162 124 402 21 5.223880597
1.109770192
682|1906|1908|2919|2921|3579|3627|4283|5175|6279|6280|6283|6356|6362|6372|6373|6374|10451|10562|57126|255231
BSG|EDN1|EDN3|CXCL1|CXCL3|CXCR2|CXCL10|CXCL9|PECAM1|S100A8|S100A9|S100A12|CCL11|CCL18|CXCL6|CXCL11|CXCL5|VAV3|OLFM4|CD177|MCOLN2 -12.82122536 0 2
0 0 -19.18499513 31.13543394
1 -15.90671083 GO:0019730 M1 1 0 GO Biological Processes 19 antimicrobial humoral response -15.90671083 10.77765021 14.1059887 28162 143 402 22
5.472636816 1.134394361
1670|1671|1672|1755|2919|2921|3627|3934|4283|5068|5266|5320|5967|5968|6279|6280|6283|6372|6373|6374|11005|116842
DEFA5|DEFA6|DEFB1|DMBT1|CXCL1|CXCL3|CXCL10|LCN2|CXCL9|REG3A|PI3|PLA2G2A|REG1A|REG1B|S100A8|S100A9|S100A12|CXCL6|CXCL11|CXCL5|SPINK5|LEAP2
-12.67264155 0 2 0 0 -19.18499513 31.13543394
1 -15.28705279 GO:0002237 M1 1 0 GO Biological Processes 19 response to molecule of bacterial origin -15.28705279 6.27657953 11.88421584 28162 346 402
31 7.711442786 1.330542411
834|1439|1604|1670|1671|1755|1906|2263|2697|2919|2921|3248|3553|3620|3627|4067|4283|4843|5105|6279|6280|6372|6373|6374|6678|7056|7474|9076|10891|29126|79931
CASP1|CSF2RB|CD55|DEFA5|DEFA6|DMBT1|EDN1|FGFR2|GJA1|CXCL1|CXCL3|HPGD|IL1B|IDO1|CXCL10|LYN|CXCL9|NOS2|PCK1|S100A8|S100A9|CXCL6|CXCL11|CXCL5|SPARC|THBD|WNT5A|CLDN1|PPARGC1A|CD274|TNIP3 -12.16948908 0 2 0 0 -19.18499513
31.13543394
1 -15.12376518 GO:0032496 M1 1 0 GO Biological Processes 19 response to lipopolysaccharide -15.12376518 6.44675396 11.90338487 28162 326 402 30

7.462686567 1.31066897
834|1439|1604|1670|1671|1906|2263|2697|2919|2921|3248|3553|3620|3627|4067|4283|4843|5105|6279|6280|6372|6373|6374|6678|7056|7474|9076|10891|29126|79931
CASP1|CSF2RB|CD55|DEFA5|DEFA6|EDN1|FGFR2|GJA1|CXCL1|CXCL3|HPGD|IL1B|IDO1|CXCL10|LYN|CXCL9|NOS2|PCK1|S100A8|S100A9|CXCL6|CXCL11|CXCL5|SPARC|THBD|WNT5A|CLDN1|PPARGC1A|CD274|TNIP3 -12.03102505 0 2 0 0 -19.18499513 31.13543394
1 -14.80740033 GO:0097529 M1 1 0 GO Biological Processes 19 myeloid leukocyte migration -14.80740033 7.96076436 12.47288334 28162 220 402 25 6.218905473
1.204485566
301|682|1906|1908|2919|2921|3579|3627|4067|4283|5175|6279|6280|6283|6356|6362|6372|6373|6374|7057|10451|10562|26585|57126|255231
ANXA1|BSG|EDN1|EDN3|CXCL1|CXCL3|CXCR2|CXCL10|LYN|CXCL9|PECAM1|S100A8|S100A9|S100A12|CCL11|CCL18|CXCL6|CXCL11|CXCL5|THBS1|VAV3|OLFM4|GREM1|CD177|MCOLN2 -11.76041769 0 2 0 0 -19.18499513 31.13543394
1 -13.78291159 GO:0071621 M1 1 0 GO Biological Processes 19 granulocyte chemotaxis -13.78291159 10.73419194 13.07267801 28162 124 402 19
4.726368159 1.058368993
301|682|1906|1908|2919|2921|3579|3627|4283|6279|6280|6283|6356|6362|6372|6373|6374|7057|10451
ANXA1|BSG|EDN1|EDN3|CXCL1|CXCL3|CXCR2|CXCL10|CXCL9|S100A8|S100A9|S100A12|CCL11|CCL18|CXCL6|CXCL11|CXCL5|THBS1|VAV3 -10.94116489 0 2 0 0
-19.18499513 31.13543394
1 -13.77066359 GO:0061844 M1 1 0 GO Biological Processes 19 antimicrobial humoral immune response mediated by antimicrobial peptide -13.77066359 14.37020028
14.22928454 28162 78 402 16 3.980099502 0.975022334
1670|1671|1672|2919|2921|3627|4283|5068|5967|5968|6280|6283|6372|6373|6374|11005
DEFA5|DEFA6|DEFB1|CXCL1|CXCL3|CXCL10|CXCL9|REG3A|REG1A|REG1B|S100A9|S100A12|CXCL6|CXCL11|CXCL5|SPINK5 -10.94116489 0 2 0 0 -19.18499513 31.13543394
1 -13.53147934 GO:0030595 M1 1 0 GO Biological Processes 19 leukocyte chemotaxis -13.53147934 7.406667105 11.66267252 28162 227 402 24
5.970149254 1.181714164
240|301|682|1906|1908|2919|2921|3579|3627|4067|4283|6279|6280|6283|6356|6362|6372|6373|6374|7057|7474|10451|11240|26585
ALOX5|ANXA1|BSG|EDN1|EDN3|CXCL1|CXCL3|CXCR2|CXCL10|LYN|CXCL9|S100A8|S100A9|S100A12|CCL11|CCL18|CXCL6|CXCL11|CXCL5|THBS1|WNT5A|VAV3|PADI2|GREM1
-10.71752714 0 2 0 0 -19.18499513 31.13543394
1 -12.94441532 GO:0030593 M1 1 0 GO Biological Processes 19 neutrophil chemotaxis -12.94441532 11.56243057 12.92330865 28162 103 402 17
4.228855721 1.003727321
682|1906|1908|2919|2921|3579|3627|4283|6279|6280|6283|6356|6362|6372|6373|6374|10451
BSG|EDN1|EDN3|CXCL1|CXCL3|CXCR2|CXCL10|CXCL9|S100A8|S100A9|S100A12|CCL11|CCL18|CXCL6|CXCL11|CXCL5|VAV3 -10.20918654 0 2 0 0 -19.18499513 31.13543394

1 -11.86160646 GO:0071396 M1 1 0 GO Biological Processes 19 cellular response to lipid -11.86160646 4.283922116 9.411434725 28162 556 402 34 8.457711443 1.387791708
301|687|834|1277|1604|1670|1671|1906|2263|2919|2921|3371|3553|3627|4067|4283|4478|4843|5105|5166|5468|6338|6372|6373|6374|6696|7474|9076|10891|11240|29126|54762|79931|339479
ANXA1|KLF9|CASP1|COL1A1|CD55|DEFA5|DEFA6|EDN1|FGFR2|CXCL1|CXCL3|TNC|IL1B|CXCL10|LYN|CXCL9|MSN|NOS2|PCK1|PDK4|PPARG|SCNN1B|CXCL6|CXCL11|CXCL5|SPP1|WNT5A|CLDN1|PPARGC1A|PADI2|CD274|GRAMD1C|TNIP3|BRINP3 -9.176351654 0 2 0 0 -19.18499513 31.13543394

1 -11.06679647 GO:1990868 M1 1 0 GO Biological Processes 19 response to chemokine -11.06679647 10.83320511 11.67416357 28162 97 402 15 3.731343284 0.945283402
1906|2919|2921|3579|3627|4283|5967|6091|6356|6362|6372|6373|6374|7032|11240
EDN1|CXCL1|CXCL3|CXCR2|CXCL10|CXCL9|REG1A|ROBO1|CCL11|CCL18|CXCL6|CXCL11|CXCL5|TFF2|PADI2 -8.474658689 0 2 0 0 -19.18499513 31.13543394

1 -11.06679647 GO:1990869 M1 1 0 GO Biological Processes 19 cellular response to chemokine -11.06679647 10.83320511 11.67416357 28162 97 402 15 3.731343284 0.945283402
1906|2919|2921|3579|3627|4283|5967|6091|6356|6362|6372|6373|6374|7032|11240
EDN1|CXCL1|CXCL3|CXCR2|CXCL10|CXCL9|REG1A|ROBO1|CCL11|CCL18|CXCL6|CXCL11|CXCL5|TFF2|PADI2 -8.474658689 0 2 0 0 -19.18499513 31.13543394

1 -10.69042577 GO:0060326 M1 1 0 GO Biological Processes 19 cell chemotaxis -10.69042577 5.476590986 9.490571255 28162 307 402 24 5.970149254 1.181714164
240|301|682|1906|1908|2919|2921|3579|3627|4067|4283|6279|6280|6283|6356|6362|6372|6373|6374|7057|7474|10451|11240|26585
ALOX5|ANXA1|BSG|EDN1|EDN3|CXCL1|CXCL3|CXCR2|CXCL10|LYN|CXCL9|S100A8|S100A9|S100A12|CCL11|CCL18|CXCL6|CXCL11|CXCL5|THBS1|WNT5A|VAV3|PADI2|GREM1 -8.120564384 0 2 0 0 -19.18499513 31.13543394

1 -10.54153634 GO:0070098 M1 1 0 GO Biological Processes 19 chemokine-mediated signaling pathway -10.54153634 11.1450701 11.47021537 28162 88 402 14 3.482587065 0.914409651
1906|2919|2921|3579|3627|4283|6091|6356|6362|6372|6373|6374|7032|11240
EDN1|CXCL1|CXCL3|CXCR2|CXCL10|CXCL9|ROBO1|CCL11|CCL18|CXCL6|CXCL11|CXCL5|TFF2|PADI2 -7.978853542 0 2 0 0 -19.18499513 31.13543394

1 -10.20678133 GO:0045236 M1 1 0 GO Molecular Functions 21 CXCR chemokine receptor binding -10.20678133 31.13543394 15.39033677 28162 18 402 8 1.990049751 0.696552772 2919|2921|3627|4283|6372|6373|6374|7032
CXCL1|CXCL3|CXCL10|CXCL9|CXCL6|CXCL11|CXCL5|TFF2 -7.658109241 0 2 0 0 -19.18499513 31.13543394

1 -9.626149143 GO:0001664 M1 1 0 GO Molecular Functions 21 G protein-coupled receptor binding -9.626149143 5.332885744 8.909670292 28162 289 402 22 5.472636816 1.134394361

183|718|1672|1906|1908|2357|2769|2919|2921|3627|4283|5028|5368|6356|6362|6372|6373|6374|7032|7474|60675|115908
AGT|C3|DEFB1|EDN1|EDN3|FPR1|GNA15|CXCL1|CXCL3|CXCL10|CXCL9|P2RY1|PNOC|CCL11|CCL18|CXCL6|CXCL11|CXCL5|TFF2|WNT5A|PROK2|CTHRC1 -7.141459364 0 2 0 0 -19.18499513 31.13543394

1 -9.24784326 GO:0071222 M1 1 0 GO Biological Processes 19 cellular response to lipopolysaccharide -9.24784326 6.400939465 9.154241023 28162 197 402 18 4.47761194 1.031484687
834|1604|1670|1671|2919|2921|3553|3627|4067|4283|4843|6372|6373|6374|7474|10891|29126|79931
CASP1|CD55|DEFA5|DEFA6|CXCL1|CXCL3|IL1B|CXCL10|LYN|CXCL9|NOS2|CXCL6|CXCL11|CXCL5|WNT5A|PPARGC1A|CD274|TNIP3 -6.818908714 0 2 0 0 -19.18499513 31.13543394

1 -8.905937109 GO:0006935 M1 1 0 GO Biological Processes 19 chemotaxis -8.905937109 3.480980192 7.664522958 28162 644 402 32 7.960199005 1.350009335
240|301|682|1672|1906|1908|1942|2357|2919|2921|3579|3627|4067|4283|5328|6091|6279|6280|6283|6356|6362|6372|6373|6374|7057|7436|7474|10451|11240|26585|60675|284217
ALOX5|ANXA1|BSG|DEFB1|EDN1|EDN3|EFNA1|FPR1|CXCL1|CXCL3|CXCR2|CXCL10|LYN|CXCL9|PLAU|ROBO1|S100A8|S100A9|S100A12|CCL11|CCL18|CXCL6|CXCL11|CXCL5|THBS1|VLDLR|WNT5A|VAV3|PADI2|GREM1|PROK2|LAMA1 -6.497443724 0 2 0 0 -19.18499513 31.13543394

1 -8.873054486 GO:0042330 M1 1 0 GO Biological Processes 19 taxis -8.873054486 3.470203164 7.643347662 28162 646 402 32 7.960199005 1.350009335
240|301|682|1672|1906|1908|1942|2357|2919|2921|3579|3627|4067|4283|5328|6091|6279|6280|6283|6356|6362|6372|6373|6374|7057|7436|7474|10451|11240|26585|60675|284217
ALOX5|ANXA1|BSG|DEFB1|EDN1|EDN3|EFNA1|FPR1|CXCL1|CXCL3|CXCR2|CXCL10|LYN|CXCL9|PLAU|ROBO1|S100A8|S100A9|S100A12|CCL11|CCL18|CXCL6|CXCL11|CXCL5|THBS1|VLDLR|WNT5A|VAV3|PADI2|GREM1|PROK2|LAMA1 -6.46952452 0 2 0 0 -19.18499513 31.13543394

1 -8.832753247 GO:0071219 M1 1 0 GO Biological Processes 19 cellular response to molecule of bacterial origin -8.832753247 6.03342141 8.789204205 28162 209 402 18 4.47761194 1.031484687
834|1604|1670|1671|2919|2921|3553|3627|4067|4283|4843|6372|6373|6374|7474|10891|29126|79931
CASP1|CD55|DEFA5|DEFA6|CXCL1|CXCL3|IL1B|CXCL10|LYN|CXCL9|NOS2|CXCL6|CXCL11|CXCL5|WNT5A|PPARGC1A|CD274|TNIP3 -6.434130615 0 2 0 0 -19.18499513 31.13543394

1 -8.420130124 GO:0042379 M1 1 0 GO Molecular Functions 21 chemokine receptor binding -8.420130124 11.16814478 10.17640867 28162 69 402 11 2.736318408 0.813665012

1672|2919|2921|3627|4283|6356|6362|6372|6373|6374|7032
DEFB1|CXCL1|CXCL3|CXCL10|CXCL9|CCL11|CCL18|CXCL6|CXCL11|CXCL5|TFF2
-6.063343561 0 2 0 0 -19.18499513 31.13543394
1 -8.083771269 GO:0071216 M1 1 0 GO Biological Processes 19 cellular response to biotic stimulus -8.083771269 5.41195311 8.137827647 28162 233 402 18 4.47761194 1.031484687
834|1604|1670|1671|2919|2921|3553|3627|4067|4283|4843|6372|6373|6374|7474|10891|29126|79931
CASP1|CD55|DEFA5|DEFA6|CXCL1|CXCL3|IL1B|CXCL10|LYN|CXCL9|NOS2|CXCL6|CXCL11|CXCL5|WNT5A|PPARGC1A|CD274|TNIP3 -5.780207917 0 2 0 0 -19.18499513 31.13543394
1 -8.025680856 GO:0005125 M1 1 0 GO Molecular Functions 21 cytokine activity -8.025680856 5.365893935 8.08764912 28162 235 402 18 4.47761194 1.031484687
1906|2919|2921|3553|3557|3627|4283|6356|6362|6372|6373|6374|6398|6696|7076|7474|26585|90865
EDN1|CXCL1|CXCL3|IL1B|IL1RN|CXCL10|CXCL9|CCL11|CCL18|CXCL6|CXCL11|CXCL5|SECTM1|SPP1|TIMP1|WNT5A|GREM1|IL33 -5.730746661 0 2 0 0 -19.18499513 31.13543394
1 -7.554057277 GO:0008009 M1 1 0 GO Molecular Functions 21 chemokine activity -7.554057277 12.86719464 10.00506331 28162 49 402 9 2.23880597 0.737867616 2919|2921|3627|4283|6356|6362|6372|6373|6374
CXCL1|CXCL3|CXCL10|CXCL9|CCL11|CCL18|CXCL6|CXCL11|CXCL5 -5.323315934 0 2 0 0 -19.18499513 31.13543394
1 -6.764575395 GO:0048018 M1 1 0 GO Molecular Functions 21 receptor ligand activity -6.764575395 3.45949266 6.581718998 28162 486 402 24 5.970149254 1.181714164
183|1906|1908|2919|2921|3553|3557|3627|4283|5368|5967|6356|6362|6372|6373|6374|6398|6696|7031|7076|7474|7873|26585|90865
AGT|EDN1|EDN3|CXCL1|CXCL3|IL1B|IL1RN|CXCL10|CXCL9|PNOC|REG1A|CCL11|CCL18|CXCL6|CXCL11|CXCL5|SECTM1|SPP1|TFF1|TIMP1|WNT5A|MANF|GREM1|IL33
-4.620225197 0 2 0 0 -19.18499513 31.13543394
1 -6.667507566 GO:0030546 M1 1 0 GO Molecular Functions 21 signaling receptor activator activity -6.667507566 3.417303725 6.509333534 28162 492 402 24 5.970149254 1.181714164
183|1906|1908|2919|2921|3553|3557|3627|4283|5368|5967|6356|6362|6372|6373|6374|6398|6696|7031|7076|7474|7873|26585|90865
AGT|EDN1|EDN3|CXCL1|CXCL3|IL1B|IL1RN|CXCL10|CXCL9|PNOC|REG1A|CCL11|CCL18|CXCL6|CXCL11|CXCL5|SECTM1|SPP1|TFF1|TIMP1|WNT5A|MANF|GREM1|IL33
-4.534338775 0 2 0 0 -19.18499513 31.13543394
1 -6.526334418 GO:0030545 M1 1 0 GO Molecular Functions 21 receptor regulator activity -6.526334418 3.255331151 6.356023666 28162 538 402 25 6.218905473 1.204485566
183|1906|1908|2919|2921|3553|3557|3627|4283|5368|5967|6356|6362|6372|6373|6

374|6398|6696|7031|7076|7474|7837|7873|26585|90865
AGT|EDN1|EDN3|CXCL1|CXCL3|IL1B|IL1RN|CXCL10|CXCL9|PNOC|REG1A|CCL11|CCL18|CXCL6|CXCL11|CXCL5|SECTM1|SPP1|TFF1|TIMP1|WNT5A|PXDN|MANF|GREM1|IL33
-4.403631061 0 2 0 0 -19.18499513 31.13543394
1 -6.387126435 GO:0005126 M1 1 0 GO Molecular Functions 21 cytokine receptor binding -6.387126435 4.410853142 6.776920743 28162 270 402 17 4.228855721 1.003727321
1672|2919|2921|3553|3557|3627|4283|6356|6362|6372|6373|6374|7032|7837|10979|26585|90865
DEFB1|CXCL1|CXCL3|IL1B|IL1RN|CXCL10|CXCL9|CCL11|CCL18|CXCL6|CXCL11|CXCL5|TFF2|PXDN|FERMT2|GREM1|IL33 -4.297520768 0 2 0 0 -19.18499513 31.13543394
1 -5.497300798 GO:0042742 M1 1 0 GO Biological Processes 19 defense response to bacterium -5.497300798 3.623520329 5.926109373 28162 348 402 18 4.47761194 1.031484687
1670|1671|1672|1755|3575|3934|4843|5266|5320|6279|6280|6283|6372|7098|11005|11126|116842|646627
DEFA5|DEFA6|DEFB1|DMBT1|IL7R|LCN2|NOS2|PI3|PLA2G2A|S100A8|S100A9|S100A12|CXCL6|TLR3|SPINK5|CD160|LEAP2|LYPD8 -3.51664408 0 2 0 0 -19.18499513 31.13543394
1 -18.21499759 GO:0006954 M1 1 0 GO Biological Processes 19 inflammatory response -18.21499759 4.502231772 11.92102424 28162 778 402 50 12.43781095 1.645952335
12|183|240|301|718|834|1116|2357|2697|2919|2921|3373|3553|3557|3579|3620|3627|4067|4225|4283|4314|4318|4843|5068|5320|5468|5729|6279|6280|6283|6356|6362|6372|6373|6374|6696|7052|7057|7076|7098|7130|7474|8876|55198|60675|64922|79931|90865|145741|405753
SERPINA3|AGT|ALOX5|ANXA1|C3|CASP1|CHI3L1|FPR1|GJA1|CXCL1|CXCL3|HYAL1|IL1B|IL1RN|CXCR2|IDO1|CXCL10|LYN|MEP1B|CXCL9|MMP3|MMP9|NOS2|REG3A|PLA2G2A|PPARG|PTGDR|S100A8|S100A9|S100A12|CCL11|CCL18|CXCL6|CXCL11|CXCL5|SPP1|TGM2|THBS1|TIMP1|TLR3|TNFAIP6|WNT5A|VNN1|APPL2|PROK2|LRRC19|TNIP3|IL33|C2CD4A|DUOXA2
-14.82122746 0 3 1 1 -18.21499759 6.847454457
1 -9.22542851 GO:0031347 M1 1 0 GO Biological Processes 19 regulation of defense response -9.22542851 3.417303725 7.776517267 28162 697 402 34 8.457711443 1.387791708
183|240|301|718|834|2697|3553|3620|4067|4314|4318|4321|4332|5272|5320|5468|6279|6280|6283|6372|7052|7098|7130|7474|11005|11126|23705|54941|55198|64922|90865|145741|405753|727897
AGT|ALOX5|ANXA1|C3|CASP1|GJA1|IL1B|IDO1|LYN|MMP3|MMP9|MMP12|MNDA|SERPINB9|PLA2G2A|PPARG|S100A8|S100A9|S100A12|CXCL6|TGM2|TLR3|TNFAIP6|WNT5A|SPINK5|CD160|CADM1|RNF125|APPL2|LRRC19|IL33|C2CD4A|DUOXA2|MUC5B -6.801695158 0 3 0 0 -18.21499759 6.847454457
1 -8.349607247 GO:0050727 M1 1 0 GO Biological Processes 19 regulation of inflammatory response -8.349607247 4.36655476 7.833547557 28162 369 402 23 5.721393035 1.158362377

183|240|301|718|834|3553|3620|4067|4314|4318|5320|5468|6279|6280|6283|7052|7098|7130|7474|64922|90865|145741|405753

AGT|ALOX5|ANXA1|C3|CASP1|IL1B|IDO1|LYN|MMP3|MMP9|PLA2G2A|PPARG|S100A8|S100A9|S100A12|TGM2|TLR3|TNFAIP6|WNT5A|LRRC19|IL33|C2CD4A|DUOXA2

-6.005915982 0 3 0 0 -18.21499759 6.847454457

1 -7.193467106 GO:0050729 M1 1 0 GO Biological Processes 19 positive regulation of inflammatory response -7.193467106 6.847454457 8.134225326 28162 133 402 13 3.233830846 0.882281956

183|718|3553|3620|5320|6279|6280|6283|7052|7098|7474|90865|145741

AGT|C3|IL1B|IDO1|PLA2G2A|S100A8|S100A9|S100A12|TGM2|TLR3|WNT5A|IL33|C2CD4A -5.009807323 0 3 0 0 -18.21499759 6.847454457

1 -6.510079142 GO:0031349 M1 1 0 GO Biological Processes 19 positive regulation of defense response -6.510079142 3.881148275 6.630042821 28162 361 402 20 4.975124378 1.084445172

183|718|2697|3553|3620|4067|4321|4332|5320|6279|6280|6283|7052|7098|7474|11126|23705|90865|145741|727897

AGT|C3|GJA1|IL1B|IDO1|LYN|MMP12|MNDA|PLA2G2A|S100A8|S100A9|S100A12|TGM2|TLR3|WNT5A|CD160|CADM1|IL33|C2CD4A|MUC5B -4.395062614 0 3 0 0 -18.21499759 6.847454457

1 -6.427580109 GO:0001817 M1 1 0 GO Biological Processes 19 regulation of cytokine production -6.427580109 2.777105521 6.06494969 28162 782 402 31 7.711442786 1.330542411

183|240|301|718|834|1001|2633|3553|3620|4067|4321|4332|4843|5450|6372|7057|7098|7474|8140|9246|11126|11148|23705|28951|28984|29126|54941|55198|64922|90865|255231

AGT|ALOX5|ANXA1|C3|CASP1|CDH3|GBP1|IL1B|IDO1|LYN|MMP12|MNDA|NOS2|POU2AF1|CXCL6|THBS1|TLR3|WNT5A|SLC7A5|UBE2L6|CD160|HHLA2|CADM1|TRIB2|RGCC|CD274|RNF125|APPL2|LRRC19|IL33|MCOLN2 -4.322605519 0 3 0 0 -18.21499759 6.847454457

1 -6.401180855 GO:0032103 M1 1 0 GO Biological Processes 19 positive regulation of response to external stimulus -6.401180855 3.303169809 6.310169938 28162 509 402 24 5.970149254 1.181714164

183|718|1906|1908|3553|3620|3627|4067|4321|4332|5320|6279|6280|6283|7052|7056|7057|7098|7474|11126|23705|90865|145741|727897

AGT|C3|EDN1|EDN3|IL1B|IDO1|CXCL10|LYN|MMP12|MNDA|PLA2G2A|S100A8|S100A9|S100A12|TGM2|THBD|THBS1|TLR3|WNT5A|CD160|CADM1|IL33|C2CD4A|MUC5B

-4.307643047 0 3 0 0 -18.21499759 6.847454457

1 -5.524118885 GO:0010942 M1 1 0 GO Biological Processes 19 positive regulation of cell death -5.524118885 2.793593383 5.576090678 28162 652 402 26 6.467661692 1.226708902

183|301|857|3248|3620|4067|4314|4318|4332|4843|5468|6279|6280|6533|7052|7057|7098|7474|10451|10891|11126|22822|28984|29126|85477|130399

AGT|ANXA1|CAV1|HPGD|IDO1|LYN|MMP3|MMP9|MNDA|NOS2|PPARG|S100A8|S100A9|SLC6A6|TGM2|THBS1|TLR3|WNT5A|VAV3|PPARGC1A|CD160|PHLDA1|RGCC|CD274|SCIN|AC

VR1C -3.536421546 0 3 0 0 -18.21499759 6.847454457

1 -5.286669896 GO:0043068 M1 1 0 GO Biological Processes 19 positive regulation of programmed cell death -5.286669896 2.849683784 5.464075507 28162 590 402 24 5.970149254 1.181714164 183|301|857|3248|3620|4067|4318|4332|4843|5468|6279|6280|7052|7057|7098|7474|10451|10891|11126|22822|28984|29126|85477|130399 AGT|ANXA1|CAV1|HPGD|IDO1|LYN|MMP9|MNDA|NOS2|PPARG|S100A8|S100A9|TGM2|THBS1|TLR3|WNT5A|VAV3|PPARGC1A|CD160|PHLDA1|RGCC|CD274|SCIN|ACVR1C -3.350276964 0 3 0 0 -18.21499759 6.847454457

1 -5.238245485 GO:0001819 M1 1 0 GO Biological Processes 19 positive regulation of cytokine production -5.238245485 3.206165967 5.593330138 28162 437 402 20 4.975124378 1.084445172 183|301|718|834|3553|3620|4321|4332|5450|7057|7098|7474|8140|11126|11148|23705|28984|29126|90865|255231 AGT|ANXA1|C3|CASP1|IL1B|IDO1|MMP12|MNDA|POU2AF1|THBS1|TLR3|WNT5A|SLC7A5|CD160|HHLA2|CADM1|RGCC|CD274|IL33|MCOLN2 -3.306078401 0 3 0 0 -18.21499759 6.847454457

1 -4.985063296 GO:0043065 M1 1 0 GO Biological Processes 19 positive regulation of apoptotic process -4.985063296 2.807070917 5.263787713 28162 574 402 23 5.721393035 1.158362377 183|301|857|3248|3620|4067|4318|4332|5468|6279|6280|7052|7057|7098|7474|10451|10891|11126|22822|28984|29126|85477|130399 AGT|ANXA1|CAV1|HPGD|IDO1|LYN|MMP9|MNDA|PPARG|S100A8|S100A9|TGM2|THBS1|TLR3|WNT5A|VAV3|PPARGC1A|CD160|PHLDA1|RGCC|CD274|SCIN|ACVR1C -3.093416691 0 3 0 0 -18.21499759 6.847454457

1 -2.143602428 GO:0001818 M1 1 0 GO Biological Processes 19 negative regulation of cytokine production -2.143602428 2.278202484 2.974215676 28162 369 402 12 2.985074627 0.848757934 301|1001|2633|3620|7057|9246|28951|28984|29126|54941|55198|90865 ANXA1|CDH3|GBP1|IDO1|THBS1|UBE2L6|TRIB2|RGCC|CD274|RNF125|APPL2|IL33 -0.811787143 0 3 0 0 -18.21499759 6.847454457

1 -15.86933699 GO:0002274 M1 1 0 GO Biological Processes 19 myeloid leukocyte activation -15.86933699 4.557266617 11.13705169 28162 661 402 43 10.69651741 1.541495895 12|240|290|718|1116|1604|2040|2212|2215|2357|2919|3579|3598|3934|4067|4318|4332|5175|5320|5328|5729|6279|6280|6282|6283|6286|6372|6402|6947|7057|7098|7130|7474|8876|10562|10970|11031|11240|22918|57126|79888|83716|90865 SERPINA3|ALOX5|ANPEP|C3|CHI3L1|CD55|STOM|FCGR2A|FCGR3B|FPR1|CXCL1|CXCR2|IL13RA2|LCN2|LYN|MMP9|MNDA|PECAM1|PLA2G2A|PLAU|PTGDR|S100A8|S100A9|S100A11|S100A12|S100P|CXCL6|SELL|TCN1|THBS1|TLR3|TNFAIP6|WNT5A|VNN1|OLFM4|CKAP4|RAB31|PADI2|CD93|CD177|LPCAT1|CRISPLD2|IL33 -12.66745239 0 4 1 1 -15.86933699 5.282815431

1 -15.57326378 GO:0002444 M1 1 0 GO Biological Processes 19 myeloid leukocyte mediated immunity -15.57326378 4.940568406 11.26220505 28162 553 402 39

9.701492537 1.476204539

12|240|290|718|1116|1604|2040|2212|2215|2357|2919|3579|3598|3934|4067|4318|4332|5175|5272|5328|5729|6279|6280|6282|6283|6286|6372|6402|6947|7130|8876|10562|10970|11031|11240|22918|57126|79888|83716

SERPINA3|ALOX5|ANPEP|C3|CHI3L1|CD55|STOM|FCGR2A|FCGR3B|FPR1|CXCL1|CXCR2|IL13RA2|LCN2|LYN|MMP9|MNDA|PECAM1|SERPINB9|PLAU|PTGDR|S100A8|S100A9|S100A11|S100A12|S100P|CXCL6|SELL|TCN1|TNFAIP6|VNN1|OLFM4|CKAP4|RAB31|PADI2|CD93|CD177|LPCAT1|CRISPLD2 -12.4013424 0 4 0 0 -15.86933699 5.282815431

1 -14.89329178 GO:0002275 M1 1 0 GO Biological Processes 19 myeloid cell activation involved in immune response -14.89329178 4.840144731 10.94478758

28162 550 402 38 9.452736318 1.459161662

12|240|290|718|1116|1604|2040|2212|2215|2357|2919|3579|3598|3934|4067|4318|4332|5175|5328|5729|6279|6280|6282|6283|6286|6402|6947|7130|8876|10562|10970|11031|11240|22918|57126|79888|83716|90865

SERPINA3|ALOX5|ANPEP|C3|CHI3L1|CD55|STOM|FCGR2A|FCGR3B|FPR1|CXCL1|CXCR2|IL13RA2|LCN2|LYN|MMP9|MNDA|PECAM1|PLAU|PTGDR|S100A8|S100A9|S100A11|S100A12|S100P|SELL|TCN1|TNFAIP6|VNN1|OLFM4|CKAP4|RAB31|PADI2|CD93|CD177|LPCAT1|CRISPLD2|IL33 -11.82403274 0 4 0 0 -15.86933699 5.282815431

1 -14.66755782 GO:0045055 M1 1 0 GO Biological Processes 19 regulated exocytosis -14.66755782 4.041618829 10.36685056 28162 780 402 45

11.19402985 1.572538494

12|240|290|308|718|1116|1604|2040|2212|2215|2357|2919|3579|3598|3934|4067|4318|4332|5028|5175|5328|5729|6279|6280|6282|6283|6286|6402|6678|6947|7057|7076|7130|7450|7873|8876|10562|10970|11031|11240|22918|57126|79888|83716|85477

SERPINA3|ALOX5|ANPEP|ANXA5|C3|CHI3L1|CD55|STOM|FCGR2A|FCGR3B|FPR1|CXCL1|CXCR2|IL13RA2|LCN2|LYN|MMP9|MNDA|P2RY1|PECAM1|PLAU|PTGDR|S100A8|S100A9|S100A11|S100A12|S100P|SELL|SPARC|TCN1|THBS1|TIMP1|TNFAIP6|VWF|MANF|VNN1|OLFM4|CKAP4|RAB31|PADI2|CD93|CD177|LPCAT1|CRISPLD2|SCIN -11.66495802 0 4 0 0

-15.86933699 5.282815431

1 -14.65124282 GO:0042119 M1 1 0 GO Biological Processes 19 neutrophil activation -14.65124282 5.033872553 10.96312661 28162 501 402 36 8.955223881

1.424140094

12|240|290|718|1116|1604|2040|2212|2215|2357|2919|3579|3934|4318|4332|5175|5320|5328|6279|6280|6282|6283|6286|6372|6402|6947|7130|8876|10562|10970|11031|11240|22918|57126|79888|83716

SERPINA3|ALOX5|ANPEP|C3|CHI3L1|CD55|STOM|FCGR2A|FCGR3B|FPR1|CXCL1|CXCR2|LCN2|MMP9|MNDA|PECAM1|PLA2G2A|PLAU|S100A8|S100A9|S100A11|S100A12|S100P|CXCL6|SELL|TCN1|TNFAIP6|VNN1|OLFM4|CKAP4|RAB31|PADI2|CD93|CD177|LPCAT1|CRISPLD2

-11.66495802 0 4 0 0 -15.86933699 5.282815431

1 -14.51829403 GO:0036230 M1 1 0 GO Biological Processes 19 granulocyte activation -14.51829403 4.98413073 10.88282104 28162 506 402 36 8.955223881

1.424140094

12|240|290|718|1116|1604|2040|2212|2215|2357|2919|3579|3934|4318|4332|5175|5320|5328|6279|6280|6282|6283|6286|6372|6402|6947|7130|8876|10562|10970|11031|1

1240|22918|57126|79888|83716
SERPINA3|ALOX5|ANPEP|C3|CHI3L1|CD55|STOM|FCGR2A|FCGR3B|FPR1|CXCL1|CXCR2|LCN2|MMP9|MNDA|PECAM1|PLA2G2A|PLAU|S100A8|S100A9|S100A11|S100A12|S100P|CXCL6|SELL|TCN1|TNFAIP6|VNN1|OLFM4|CKAP4|RAB31|PADI2|CD93|CD177|LPCAT1|CRISPLD2
-11.55214601 0 4 0 0 -15.86933699 5.282815431
1 -14.47277259 GO:0043299 M1 1 0 GO Biological Processes 19 leukocyte degranulation -14.47277259 4.826861966 10.77472337 28162 537 402 37
9.2039801 1.441810602
12|240|290|718|1116|1604|2040|2212|2215|2357|2919|3579|3598|3934|4067|4318|4332|5175|5328|5729|6279|6280|6282|6283|6286|6402|6947|7130|8876|10562|10970|11031|11240|22918|57126|79888|83716
SERPINA3|ALOX5|ANPEP|C3|CHI3L1|CD55|STOM|FCGR2A|FCGR3B|FPR1|CXCL1|CXCR2|IL13RA2|LCN2|LYN|MMP9|MNDA|PECAM1|PLAU|PTGDR|S100A8|S100A9|S100A11|S100A12|S100P|SELL|TCN1|TNFAIP6|VNN1|OLFM4|CKAP4|RAB31|PADI2|CD93|CD177|LPCAT1|CRISPLD2 -11.53973329 0 4 0 0 -15.86933699 5.282815431
1 -13.91491304 GO:0002446 M1 1 0 GO Biological Processes 19 neutrophil mediated immunity -13.91491304 4.903830846 10.59891812 28162 500 402 35
8.706467662 1.406138096
12|240|290|718|1116|1604|2040|2212|2215|2357|2919|3579|3934|4318|4332|5175|5328|6279|6280|6282|6283|6286|6372|6402|6947|7130|8876|10562|10970|11031|11240|22918|57126|79888|83716
SERPINA3|ALOX5|ANPEP|C3|CHI3L1|CD55|STOM|FCGR2A|FCGR3B|FPR1|CXCL1|CXCR2|LCN2|MMP9|MNDA|PECAM1|PLAU|S100A8|S100A9|S100A11|S100A12|S100P|CXCL6|SELL|TCN1|TNFAIP6|VNN1|OLFM4|CKAP4|RAB31|PADI2|CD93|CD177|LPCAT1|CRISPLD2 -11.0292984
0 4 0 0 -15.86933699 5.282815431
1 -13.52147173 GO:0043312 M1 1 0 GO Biological Processes 19 neutrophil degranulation -13.52147173 4.900947935 10.43912167 28162 486 402 34
8.457711443 1.387791708
12|240|290|718|1116|1604|2040|2212|2215|2357|2919|3579|3934|4318|4332|5175|5328|6279|6280|6282|6283|6286|6402|6947|7130|8876|10562|10970|11031|11240|22918|57126|79888|83716
SERPINA3|ALOX5|ANPEP|C3|CHI3L1|CD55|STOM|FCGR2A|FCGR3B|FPR1|CXCL1|CXCR2|LCN2|MMP9|MNDA|PECAM1|PLAU|S100A8|S100A9|S100A11|S100A12|S100P|SELL|TCN1|TNFAIP6|VNN1|OLFM4|CKAP4|RAB31|PADI2|CD93|CD177|LPCAT1|CRISPLD2 -10.71752714 0
4 0 0 -15.86933699 5.282815431
1 -13.44421222 GO:0002283 M1 1 0 GO Biological Processes 19 neutrophil activation involved in immune response -13.44421222 4.87088077 10.3911457
28162 489 402 34 8.457711443 1.387791708
12|240|290|718|1116|1604|2040|2212|2215|2357|2919|3579|3934|4318|4332|5175|5328|6279|6280|6282|6283|6286|6402|6947|7130|8876|10562|10970|11031|11240|22918|57126|79888|83716
SERPINA3|ALOX5|ANPEP|C3|CHI3L1|CD55|STOM|FCGR2A|FCGR3B|FPR1|CXCL1|CXCR2|LCN2|MMP9|MNDA|PECAM1|PLAU|S100A8|S100A9|S100A11|S100A12|S100P|SELL|TCN1|TNFAIP6|VNN1|OLFM4|CKAP4|RAB31|PADI2|CD93|CD177|LPCAT1|CRISPLD2 -10.65250208 0

4 0 0 -15.86933699 5.282815431
1 -13.19798363 GO:0002366 M1 1 0 GO Biological Processes 19 leukocyte activation involved in immune response -13.19798363 3.989227474 9.777879151 28162 720 402 41 10.19900498 1.509407313
12|240|290|301|718|1116|1604|2040|2212|2215|2357|2919|3579|3598|3934|4067|4318|4332|5105|5175|5328|5450|5729|6279|6280|6282|6283|6286|6402|6947|7130|8876|10562|10970|11031|11240|22918|57126|79888|83716|90865
SERPINA3|ALOX5|ANPEP|ANXA1|C3|CHI3L1|CD55|STOM|FCGR2A|FCGR3B|FPR1|CXCL1|CXCR2|IL13RA2|LCN2|LYN|MMP9|MNDA|PCK1|PECAM1|PLAU|POU2AF1|PTGDR|S100A8|S100A9|S100A11|S100A12|S100P|SELL|TCN1|TNFAIP6|VNN1|OLFM4|CKAP4|RAB31|PADI2|CD93|CD177|LPCAT1|CRISPLD2|IL33 -10.44103559 0 4 0 0 -15.86933699 5.282815431
1 -13.11879279 GO:0002263 M1 1 0 GO Biological Processes 19 cell activation involved in immune response -13.11879279 3.967187543 9.733418263 28162 724 402 41 10.19900498 1.509407313
12|240|290|301|718|1116|1604|2040|2212|2215|2357|2919|3579|3598|3934|4067|4318|4332|5105|5175|5328|5450|5729|6279|6280|6282|6283|6286|6402|6947|7130|8876|10562|10970|11031|11240|22918|57126|79888|83716|90865
SERPINA3|ALOX5|ANPEP|ANXA1|C3|CHI3L1|CD55|STOM|FCGR2A|FCGR3B|FPR1|CXCL1|CXCR2|IL13RA2|LCN2|LYN|MMP9|MNDA|PCK1|PECAM1|PLAU|POU2AF1|PTGDR|S100A8|S100A9|S100A11|S100A12|S100P|SELL|TCN1|TNFAIP6|VNN1|OLFM4|CKAP4|RAB31|PADI2|CD93|CD177|LPCAT1|CRISPLD2|IL33 -10.37284014 0 4 0 0 -15.86933699 5.282815431
1 -9.955872464 GO:0030667 M1 1 0 GO Cellular Components20 secretory granule membrane -9.955872464 5.282815431 9.04980952 28162 305 402 23 5.721393035 1.158362377
290|682|762|857|952|1604|1755|2040|2212|2215|2357|3579|5175|5328|6402|6678|8876|10970|11031|22918|27074|57126|79888
ANPEP|BSG|CA4|CAV1|CD38|CD55|DMBT1|STOM|FCGR2A|FCGR3B|FPR1|CXCR2|PECAM1|PLAU|SELL|SPARC|VNN1|CKAP4|RAB31|CD93|LAMP3|CD177|LPCAT1-7.446708916 0 4 0 0 -15.86933699 5.282815431
1 -8.74230528 GO:0034774 M1 1 0 GO Cellular Components20 secretory granule lumen -8.74230528 4.786347764 8.223205668 28162 322 402 22 5.472636816 1.134394361
12|240|718|1116|1670|2919|3934|4332|5122|6279|6280|6282|6283|6286|6678|6947|7057|7076|7450|10562|11240|83716
SERPINA3|ALOX5|C3|CHI3L1|DEFA5|CXCL1|LCN2|MNDA|PCSK1|S100A8|S100A9|S100A11|S100A12|S100P|SPARC|TCN1|THBS1|TIMP1|VWF|OLFM4|PADI2|CRISPLD2
-6.348535151 0 4 0 0 -15.86933699 5.282815431
1 -8.642961439 GO:0060205 M1 1 0 GO Cellular Components20 cytoplasmic vesicle lumen -8.642961439 4.727619571 8.146373186 28162 326 402 22 5.472636816 1.134394361
12|240|718|1116|1670|2919|3934|4332|5122|6279|6280|6282|6283|6286|6678|6947|7057|7076|7450|10562|11240|83716
SERPINA3|ALOX5|C3|CHI3L1|DEFA5|CXCL1|LCN2|MNDA|PCSK1|S100A8|S100A9|S100A11|S100A12|S100P|SPARC|TCN1|THBS1|TIMP1|VWF|OLFM4|PADI2|CRISPLD2

-6.258736628 0 4 0 0 -15.86933699 5.282815431
1 -8.593868561 GO:0031983 M1 1 0 GO Cellular Components20 vesicle lumen -8.593868561 4.698792622 8.108423526 28162 328 402 22 5.472636816 1.134394361
12|240|718|1116|1670|2919|3934|4332|5122|6279|6280|6282|6283|6286|6678|6947|7057|7076|7450|10562|11240|83716
SERPINA3|ALOX5|C3|CHI3L1|DEFA5|CXCL1|LCN2|MNDA|PCSK1|S100A8|S100A9|S100A11|S100A12|S100P|SPARC|TCN1|THBS1|TIMP1|VWF|OLFM4|PADI2|CRISPLD2
-6.214338871 0 4 0 0 -15.86933699 5.282815431
1 -6.976021703 GO:0030659 M1 1 0 GO Cellular Components20 cytoplasmic vesicle membrane -6.976021703 2.881428334 6.404074382 28162 778 402 32 7.960199005 1.350009335
290|301|682|762|857|952|1604|1755|2040|2212|2215|2357|2555|3575|3579|5175|5328|6338|6402|6678|7474|8876|10970|11015|11031|22918|27074|55198|57126|57214|79888|282679
ANPEP|ANXA1|BSG|CA4|CAV1|CD38|CD55|DMBT1|STOM|FCGR2A|FCGR3B|FPR1|GABRA2|IL7R|CXCR2|PECAM1|PLAU|SCNN1B|SELL|SPARC|WNT5A|VNN1|CKAP4|KDELR3|RAB31|CD93|LAMP3|APPL2|CD177|CEMIP|LPCAT1|AQP11 -4.804100323 0 4 0 0
-15.86933699 5.282815431
1 -14.69303631 GO:0045177 M1 1 0 GO Cellular Components20 apical part of cell -14.69303631 5.55722589 11.26871415 28162 416 402 33 8.208955224 1.369087084
301|343|762|1513|1836|2151|2697|4478|5028|5174|5243|6338|6505|6533|6550|6566|6584|8140|9076|9429|9963|10050|22802|23255|23657|50506|53841|81693|140803|154043|340024|387755|405753
ANXA1|AQP8|CA4|CTSK|SLC26A2|F2RL2|GJA1|MSN|P2RY1|PDZK1|ABCB1|SCNN1B|SLC1A1|SLC6A6|SLC9A3|SLC16A1|SLC22A5|SLC7A5|CLDN1|ABCG2|SLC23A1|SLC17A4|CLCA4|MTCL1|SLC7A11|DUOX2|CDHR5|AMN|TRPM6|CNKSR3|SLC6A19|INSC|DUOXA2 -11.66724297 0 5 1 1 -14.69303631 13.06105068
1 -13.355918 GO:0016324 M1 1 0 GO Cellular Components20 apical plasma membrane -13.355918 5.755204149 10.81910076 28162 353 402 29 7.213930348 1.290370231
301|762|1513|1836|2151|2697|4478|5028|5174|5243|6338|6505|6533|6550|6566|6584|8140|9076|9429|9963|10050|22802|23255|50506|53841|81693|140803|154043|340024
ANXA1|CA4|CTSK|SLC26A2|F2RL2|GJA1|MSN|P2RY1|PDZK1|ABCB1|SCNN1B|SLC1A1|SLC6A6|SLC9A3|SLC16A1|SLC22A5|SLC7A5|CLDN1|ABCG2|SLC23A1|SLC17A4|CLCA4|MTCL1|DUOX2|CDHR5|AMN|TRPM6|CNKSR3|SLC6A19 -10.57610709 0 5 0 0 -14.69303631 13.06105068
1 -9.178683515 GO:0031526 M1 1 0 GO Cellular Components20 brush border membrane -9.178683515 13.06105068 11.15994716 28162 59 402 11 2.736318408 0.813665012
762|5174|6519|6550|6584|9429|23657|53841|81693|140803|340024
CA4|PDZK1|SLC3A1|SLC9A3|SLC22A5|ABCG2|SLC7A11|CDHR5|AMN|TRPM6|SLC6A19
-6.760089803 0 5 0 0 -14.69303631 13.06105068

1	-8.35400382	GO:0031253	M1	1	0	GO Cellular Components20	cell projection membrane	-8.35400382	4.559775089	7.923160606	28162	338	402	22	5.472636816	1.134394361	762|1836|2555|4478|5145|5174|5967|6286|6519|6533|6550|6584|8140|9429|10979|23362|23657|53841|55198|81693|140803|340024	CA4|SLC26A2|GABRA2|MSN|PDE6A|PDZK1|REG1A|S100P|SLC3A1|SLC6A6|SLC9A3|SLC22A5|SLC7A5|ABCG2|FERMT2|PSD3|SLC7A11|CDHR5|APPL2|AMN|TRPM6|SLC6A19	-6.005991182	0	5	0	0	-14.69303631	13.06105068

1	-6.657112204	GO:0005903	M1	1	0	GO Cellular Components20	brush border	-6.657112204	7.629722674	8.032149974	28162	101	402	11	2.736318408	0.813665012	762|5174|6519|6550|6584|9429|23657|53841|81693|140803|340024	CA4|PDZK1|SLC3A1|SLC9A3|SLC22A5|ABCG2|SLC7A11|CDHR5|AMN|TRPM6|SLC6A19	-4.526583509	0	5	0	0	-14.69303631	13.06105068

1	-5.569477596	GO:0098862	M1	1	0	GO Cellular Components20	cluster of actin-based cell projections	-5.569477596	5.388825105	6.614733679	28162	156	402	12	2.985074627	0.848757934	762|3620|5174|6519|6550|6584|9429|23657|53841|81693|140803|340024	CA4|IDO1|PDZK1|SLC3A1|SLC9A3|SLC22A5|ABCG2|SLC7A11|CDHR5|AMN|TRPM6|SLC6A19	-3.573647476	0	5	0	0	-14.69303631	13.06105068

1	-14.50221864	GO:0001568	M1	1	0	GO Biological Processes	19	blood vessel development	-14.50221864	4.08265955	10.33226703	28162	755	402	44	10.94527363	1.557143983	240|290|301|682|718|857|1116|1277|1278|1282|1306|1906|1942|2263|3248|3373|3553|3627|4017|4162|4313|5175|5396|5468|6091|6356|6505|6678|7045|7057|7098|7127|7453|7474|9510|10451|11005|11126|23452|26585|28984|56999|60675|284217	ALOX5|ANPEP|ANXA1|BSG|C3|CAV1|CHI3L1|COL1A1|COL1A2|COL4A1|COL15A1|EDN1|EFNA1|FGFR2|HPGD|HYAL1|IL1B|CXCL10|LOXL2|MCAM|MMP2|PECAM1|PRRX1|PPARG|ROBO1|CCL11|SLC1A1|SPARC|TGFBI|THBS1|TLR3|TNFAIP2|WARS1|WNT5A|ADAMTS1|VAV3|SPINK5|CD160|ANGPTL2|GREM1|RGCC|ADAMTS9|PROK2|LAMA1	-11.55214601	0	6	1	1	-14.50221864	9.137573005

1	-14.06608207	GO:0048514	M1	1	0	GO Biological Processes	19	blood vessel morphogenesis	-14.06608207	4.23634776	10.26496307	28162	678	402	41	10.19900498	1.509407313	240|290|301|682|718|857|1116|1282|1306|1906|1942|2263|3248|3373|3553|3627|4017|4162|4313|5396|5468|6091|6356|6505|6678|7045|7057|7098|7127|7453|7474|9510|10451|11005|11126|23452|26585|28984|56999|60675|284217	ALOX5|ANPEP|ANXA1|BSG|C3|CAV1|CHI3L1|COL4A1|COL15A1|EDN1|EFNA1|FGFR2|HPGD|HYAL1|IL1B|CXCL10|LOXL2|MCAM|MMP2|PRRX1|PPARG|ROBO1|CCL11|SLC1A1|SPARC|TGFBI|THBS1|TLR3|TNFAIP2|WARS1|WNT5A|ADAMTS1|VAV3|SPINK5|CD160|ANGPTL2|GREM1|RGCC|ADAMTS9|PROK2|LAMA1	-11.16522746	0	6	0	0	-14.50221864	9.137573005

1	-13.86358075	GO:0001944	M1	1	0	GO Biological Processes	19	vasculature development	-13.86358075	3.916655604	9.986717967	28162	787	402	44	10.94527363	1.557143983	240|290|301|682|718|857|1116|1277|1278|1282|1306|1906|1942|2263|3248|3373|3

553|3627|4017|4162|4313|5175|5396|5468|6091|6356|6505|6678|7045|7057|7098|7127|7453|7474|9510|10451|11005|11126|23452|26585|28984|56999|60675|284217

ALOX5|ANPEP|ANXA1|BSG|C3|CAV1|CHI3L1|COL1A1|COL1A2|COL4A1|COL15A1|EDN1|EFNA1|FGFR2|HPGD|HYAL1|IL1B|CXCL10|LOXL2|MCAM|MMP2|PECAM1|PRRX1|PPARG|ROBO1|CCL11|SLC1A1|SPARC|TGFBI|THBS1|TLR3|TNFAIP2|WARS1|WNT5A|ADAMTS1|VAV3|SPINK5|CD160|ANGPTL2|GREM1|RGCC|ADAMTS9|PROK2|LAMA1 -10.99268936 0 6 0 0 -14.50221864 9.137573005

1 -13.22868061 GO:0001525 M1 1 0 GO Biological Processes 19 angiogenesis -13.22868061 4.400721351 10.03723326 28162 589 402 37 9.2039801 1.441810602

240|290|301|682|718|857|1116|1282|1306|1906|1942|2263|3373|3553|3627|4017|4162|4313|5468|6091|6356|6678|7045|7057|7098|7127|7453|7474|9510|10451|11005|11126|23452|26585|28984|56999|60675

ALOX5|ANPEP|ANXA1|BSG|C3|CAV1|CHI3L1|COL4A1|COL15A1|EDN1|EFNA1|FGFR2|HYAL1|IL1B|CXCL10|LOXL2|MCAM|MMP2|PPARG|ROBO1|CCL11|SPARC|TGFBI|THBS1|TLR3|TNFAIP2|WARS1|WNT5A|ADAMTS1|VAV3|SPINK5|CD160|ANGPTL2|GREM1|RGCC|ADAMTS9|PROK2 -10.46045157 0 6 0 0 -14.50221864 9.137573005

1 -7.672216324 GO:0045765 M1 1 0 GO Biological Processes 19 regulation of angiogenesis -7.672216324 4.365428053 7.479369799 28162 337 402 21 5.223880597 1.109770192

240|718|1116|1942|3373|3553|3627|5468|6356|6678|7057|7098|7453|7474|9510|11005|11126|26585|28984|56999|60675

ALOX5|C3|CHI3L1|EFNA1|HYAL1|IL1B|CXCL10|PPARG|CCL11|SPARC|THBS1|TLR3|WARS1|WNT5A|ADAMTS1|SPINK5|CD160|GREM1|RGCC|ADAMTS9|PROK2 -5.421113699 0 6 0 0 -14.50221864 9.137573005

1 -7.540239551 GO:1901342 M1 1 0 GO Biological Processes 19 regulation of vasculature development -7.540239551 4.28906488 7.375238603 28162 343 402 21 5.223880597 1.109770192

240|718|1116|1942|3373|3553|3627|5468|6356|6678|7057|7098|7453|7474|9510|11005|11126|26585|28984|56999|60675

ALOX5|C3|CHI3L1|EFNA1|HYAL1|IL1B|CXCL10|PPARG|CCL11|SPARC|THBS1|TLR3|WARS1|WNT5A|ADAMTS1|SPINK5|CD160|GREM1|RGCC|ADAMTS9|PROK2 -5.316737215 0 6 0 0 -14.50221864 9.137573005

1 -4.303906386 GO:0016525 M1 1 0 GO Biological Processes 19 negative regulation of angiogenesis -4.303906386 4.798268929 5.537151501 28162 146 402 10 2.487562189 0.776790586

240|3627|5468|6678|7057|9510|11005|11126|28984|56999

ALOX5|CXCL10|PPARG|SPARC|THBS1|ADAMTS1|SPINK5|CD160|RGCC|ADAMTS9 -2.53795711 0 6 0 0 -14.50221864 9.137573005

1 -4.253855673 GO:2000181 M1 1 0 GO Biological Processes 19 negative regulation of blood vessel morphogenesis -4.253855673 4.733427457 5.479972065 28162 148 402 10 2.487562189 0.776790586

240|3627|5468|6678|7057|9510|11005|11126|28984|56999

ALOX5|CXCL10|PPARG|SPARC|THBS1|ADAMTS1|SPINK5|CD160|RGCC|ADAMTS9

-2.49467476 0 6 0 0 -14.50221864 9.137573005
1 -4.22914765 GO:1901343 M1 1 0 GO Biological Processes 19 negative regulation of vasculature development -4.22914765 4.701659488 5.451764928 28162 149 402 10 2.487562189 0.776790586 240|3627|5468|6678|7057|9510|11005|11126|28984|56999 ALOX5|CXCL10|PPARG|SPARC|THBS1|ADAMTS1|SPINK5|CD160|RGCC|ADAMTS9 -2.474421078 0 6 0 0 -14.50221864 9.137573005
1 -2.603613649 GO:1904037 M1 1 0 GO Biological Processes 19 positive regulation of epithelial cell apoptotic process -2.603613649 7.005472637 4.573844738 28162 40 402 4 0.995024876 0.495031064 7057|10891|11126|28984 THBS1|PPARGC1A|CD160|RGCC -1.168884912 0 6 0 0 -14.50221864 9.137573005
1 -2.383476348 GO:2000353 M1 1 0 GO Biological Processes 19 positive regulation of endothelial cell apoptotic process -2.383476348 9.137573005 4.698203434 28162 23 402 3 0.746268657 0.429247719 7057|11126|28984 THBS1|CD160|RGCC -1.001605443 0 6 0 0 -14.50221864 9.137573005
1 -2.139188431 GO:1903671 M1 1 0 GO Biological Processes 19 negative regulation of sprouting angiogenesis -2.139188431 7.505863539 4.144723426 28162 28 402 3 0.746268657 0.429247719 240|7057|56999 ALOX5|THBS1|ADAMTS9 -0.810292083 0 6 0 0 -14.50221864 9.137573005
1 -14.44741806 GO:0042060 M1 1 0 GO Biological Processes 19 wound healing -14.44741806 4.817890103 10.75966154 28162 538 402 37 9.2039801 1.441810602 240|301|308|725|857|1001|1277|1278|1281|1906|2151|2263|2697|2769|3371|4067|4162|4321|5028|5068|5270|5328|5967|6279|6678|7056|7057|7076|7450|7474|7980|9076|10451|10979|23657|50506|57126 ALOX5|ANXA1|ANXA5|C4BPB|CAV1|CDH3|COL1A1|COL1A2|COL3A1|EDN1|F2RL2|FGFR2|GJA1|GNA15|TNC|LYN|MCAM|MMP12|P2RY1|REG3A|SERPINE2|PLAU|REG1A|S100A8|SPARC|THBD|THBS1|TIMP1|VWF|WNT5A|TFPI2|CLDN1|VAV3|FERMT2|SLC7A11|DUOX2|CD177 -11.53076918 0 7 1 1 -14.44741806 5.320612129
1 -13.80479342 GO:0009611 M1 1 0 GO Biological Processes 19 response to wounding -13.80479342 4.25864598 10.17840038 28162 658 402 40 9.950248756 1.492949787 240|301|308|725|857|1001|1277|1278|1281|1906|2151|2263|2697|2769|3371|4067|4162|4321|5028|5068|5270|5328|5967|6279|6505|6678|6696|7056|7057|7076|7450|7474|7980|9076|10451|10979|23657|50506|57126|64764 ALOX5|ANXA1|ANXA5|C4BPB|CAV1|CDH3|COL1A1|COL1A2|COL3A1|EDN1|F2RL2|FGFR2|GJA1|GNA15|TNC|LYN|MCAM|MMP12|P2RY1|REG3A|SERPINE2|PLAU|REG1A|S100A8|SLC1A1|SPARC|SPP1|THBD|THBS1|TIMP1|VWF|WNT5A|TFPI2|CLDN1|VAV3|FERMT2|SLC7A11|DUOX2|CD177|CREB3L2 -10.94814247 0 7 0 0 -14.44741806 5.320612129
1 -6.865184278 GO:0007596 M1 1 0 GO Biological Processes 19 blood coagulation -6.865184278 4.084823695 6.917258136 28162 343 402 20 4.975124378 1.084445172 308|725|857|1277|1278|1281|1906|2151|2769|4067|5028|5270|5328|7056|7057|745

0|7980|10451|23657|57126
ANXA5|C4BPB|CAV1|COL1A1|COL1A2|COL3A1|EDN1|F2RL2|GNA15|LYN|P2RY1|SERPINE2|PLAU|THBD|THBS1|VWF|TFPI2|VAV3|SLC7A11|CD177 -4.707503337 0 7 0 0
-14.44741806 5.320612129
1 -6.78417771 GO:0007599 M1 1 0 GO Biological Processes 19 hemostasis
-6.78417771 4.03773639 6.851767552 28162 347 402 20 4.975124378
1.084445172
308|725|857|1277|1278|1281|1906|2151|2769|4067|5028|5270|5328|7056|7057|7450|7980|10451|23657|57126
ANXA5|C4BPB|CAV1|COL1A1|COL1A2|COL3A1|EDN1|F2RL2|GNA15|LYN|P2RY1|SERPINE2|PLAU|THBD|THBS1|VWF|TFPI2|VAV3|SLC7A11|CD177 -4.634822159 0 7 0 0
-14.44741806 5.320612129
1 -6.764117853 GO:0050817 M1 1 0 GO Biological Processes 19 coagulation
-6.764117853 4.026133699 6.835548053 28162 348 402 20 4.975124378
1.084445172
308|725|857|1277|1278|1281|1906|2151|2769|4067|5028|5270|5328|7056|7057|7450|7980|10451|23657|57126
ANXA5|C4BPB|CAV1|COL1A1|COL1A2|COL3A1|EDN1|F2RL2|GNA15|LYN|P2RY1|SERPINE2|PLAU|THBD|THBS1|VWF|TFPI2|VAV3|SLC7A11|CD177 -4.620225197 0 7 0 0
-14.44741806 5.320612129
1 -6.447270673 GO:0050878 M1 1 0 GO Biological Processes 19 regulation of body fluid levels -6.447270673 3.32275382 6.344699973 28162 506 402 24
5.970149254 1.181714164
308|360|725|857|1277|1278|1281|1906|2151|2697|2769|4067|5028|5270|5328|6338|7056|7057|7450|7980|9076|10451|23657|57126
ANXA5|AQP3|C4BPB|CAV1|COL1A1|COL1A2|COL3A1|EDN1|F2RL2|GJA1|GNA15|LYN|P2RY1|SERPINE2|PLAU|SCNN1B|THBD|THBS1|VWF|TFPI2|CLDN1|VAV3|SLC7A11|CD177
-4.337304137 0 7 0 0 -14.44741806 5.320612129
1 -5.511732263 GO:0030168 M1 1 0 GO Biological Processes 19 platelet activation
-5.511732263 5.320612129 6.553768874 28162 158 402 12 2.985074627
0.848757934 1277|1278|1281|2151|2769|4067|5028|5270|7056|7450|10451|23657
COL1A1|COL1A2|COL3A1|F2RL2|GNA15|LYN|P2RY1|SERPINE2|THBD|VWF|VAV3|SLC7A11 -3.527331604 0 7 0 0 -14.44741806 5.320612129
1 -12.34993116 GO:0055080 M1 1 0 GO Biological Processes 19 cation homeostasis -12.34993116 3.838615143 9.351182681 28162 730 402 40
9.950248756 1.492949787
183|288|857|952|1604|1906|1908|2151|2357|2697|2769|3579|3627|3934|4067|4283|4499|5028|5166|5729|5967|6279|6280|6338|6356|6373|6505|6550|7052|7474|8671|9429|55151|55532|57214|60675|79689|159371|255231|282679
AGT|ANK3|CAV1|CD38|CD55|EDN1|EDN3|F2RL2|FPR1|GJA1|GNA15|CXCR2|CXCL10|LCN2|LYN|CXCL9|MT1M|P2RY1|PDK4|PTGDR|REG1A|S100A8|S100A9|SCNN1B|CCL11|CXCL11|SLC1A1|SLC9A3|TGM2|WNT5A|SLC4A4|ABCG2|TMEM38B|SLC30A10|CEMIP|PROK2|STEAP4|SLC35G1|MCOLN2|AQP11-9.635386976 0 8 1 1 -12.34993116 42.03283582
1 -12.1629314 GO:0098771 M1 1 0 GO Biological Processes 19 inorganic ion

homeostasis -12.1629314 3.786741966 9.244648143 28162 740 402 40 9.950248756 1.492949787 183|288|857|952|1604|1906|1908|2151|2357|2697|2769|3579|3627|3934|4067|4283|4499|5028|5166|5729|5967|6279|6280|6338|6356|6373|6505|6550|7052|7474|8671|9429|55151|55532|57214|60675|79689|159371|255231|282679 AGT|ANK3|CAV1|CD38|CD55|EDN1|EDN3|F2RL2|FPR1|GJA1|GNA15|CXCR2|CXCL10|LCN2|LYN|CXCL9|MT1M|P2RY1|PDK4|PTGDR|REG1A|S100A8|S100A9|SCNN1B|CCL11|CXCL11|SLC1A1|SLC9A3|TGM2|WNT5A|SLC4A4|ABCG2|TMEM38B|SLC30A10|CEMIP|PROK2|STEAP4|SLC35G1|MCOLN2|AQP11-9.458371439 0 8 0 0 -12.34993116 42.03283582

1 -11.94372481 GO:0050801 M1 1 0 GO Biological Processes 19 ion homeostasis -11.94372481 3.649610904 9.072348748 28162 787 402 41 10.19900498 1.509407313 183|288|857|952|1604|1906|1908|2151|2357|2697|2769|3579|3627|3934|4067|4283|4499|5028|5166|5729|5967|6279|6280|6338|6356|6373|6505|6550|7052|7474|8671|9429|23657|55151|55532|57214|60675|79689|159371|255231|282679 AGT|ANK3|CAV1|CD38|CD55|EDN1|EDN3|F2RL2|FPR1|GJA1|GNA15|CXCR2|CXCL10|LCN2|LYN|CXCL9|MT1M|P2RY1|PDK4|PTGDR|REG1A|S100A8|S100A9|SCNN1B|CCL11|CXCL11|SLC1A1|SLC9A3|TGM2|WNT5A|SLC4A4|ABCG2|SLC7A11|TMEM38B|SLC30A10|CEMIP|PROK2|STEAP4|SLC35G1|MCOLN2|AQP11 -9.24892468 0 8 0 0 -12.34993116 42.03283582

1 -11.32308266 GO:0055065 M1 1 0 GO Biological Processes 19 metal ion homeostasis -11.32308266 3.891929243 8.962426998 28162 648 402 36 8.955223881 1.424140094 183|288|857|952|1604|1906|1908|2151|2357|2697|2769|3579|3627|3934|4067|4283|4499|5028|5729|5967|6279|6280|6338|6356|6373|6505|7052|7474|9429|55151|55532|57214|60675|79689|159371|255231 AGT|ANK3|CAV1|CD38|CD55|EDN1|EDN3|F2RL2|FPR1|GJA1|GNA15|CXCR2|CXCL10|LCN2|LYN|CXCL9|MT1M|P2RY1|PTGDR|REG1A|S100A8|S100A9|SCNN1B|CCL11|CXCL11|SLC1A1|TGM2|WNT5A|ABCG2|TMEM38B|SLC30A10|CEMIP|PROK2|STEAP4|SLC35G1|MCOLN2 -8.707463779 0 8 0 0 -12.34993116 42.03283582

1 -11.0077023 GO:0072507 M1 1 0 GO Biological Processes 19 divalent inorganic cation homeostasis -11.0077023 4.343393035 9.077326965 28162 500 402 31 7.711442786 1.330542411 288|857|952|1604|1906|1908|2151|2357|2697|2769|3579|3627|4067|4283|4499|5028|5729|5967|6279|6280|6356|6373|6505|7052|7474|55151|55532|57214|60675|159371|255231 ANK3|CAV1|CD38|CD55|EDN1|EDN3|F2RL2|FPR1|GJA1|GNA15|CXCR2|CXCL10|LYN|CXCL9|MT1M|P2RY1|PTGDR|REG1A|S100A8|S100A9|CCL11|CXCL11|SLC1A1|TGM2|WNT5A|TMEM38B|SLC30A10|CEMIP|PROK2|SLC35G1|MCOLN2 -8.423117655 0 8 0 0 -12.34993116 42.03283582

1 -10.04156164 GO:0072503 M1 1 0 GO Biological Processes 19 cellular divalent inorganic cation homeostasis -10.04156164 4.223673731 8.581437875 28162 481 402 29 7.213930348 1.290370231 857|952|1604|1906|1908|2151|2357|2697|2769|3579|3627|4067|4283|4499|5028|5729|6279|6280|6356|6373|6505|7052|7474|55151|55532|57214|60675|159371|255231

CAV1|CD38|CD55|EDN1|EDN3|F2RL2|FPR1|GJA1|GNA15|CXCR2|CXCL10|LYN|CXCL9|MT1M|P2RY1|PTGDR|S100A8|S100A9|CCL11|CXCL11|SLC1A1|TGM2|WNT5A|TMEM38B|SLC30A10|CEMIP|PROK2|SLC35G1|MCOLN2 -7.519623804 0 8 0 0 -12.34993116 42.03283582

1 -9.929853849 GO:0030003 M1 1 0 GO Biological Processes 19 cellular cation homeostasis -9.929853849 3.636428544 8.215658398 28162 655 402 34 8.457711443 1.387791708

857|952|1604|1906|1908|2151|2357|2697|2769|3579|3627|3934|4067|4283|4499|5028|5729|6279|6280|6356|6373|6505|6550|7052|7474|8671|9429|55151|55532|57214|60675|159371|255231|282679

CAV1|CD38|CD55|EDN1|EDN3|F2RL2|FPR1|GJA1|GNA15|CXCR2|CXCL10|LCN2|LYN|CXCL9|MT1M|P2RY1|PTGDR|S100A8|S100A9|CCL11|CXCL11|SLC1A1|SLC9A3|TGM2|WNT5A|SLC4A4|ABCG2|TMEM38B|SLC30A10|CEMIP|PROK2|SLC35G1|MCOLN2|AQP11 -7.426939251 0 8 0 0 -12.34993116 42.03283582

1 -9.705340293 GO:0006873 M1 1 0 GO Biological Processes 19 cellular ion homeostasis -9.705340293 3.565659725 8.075987831 28162 668 402 34 8.457711443 1.387791708

857|952|1604|1906|1908|2151|2357|2697|2769|3579|3627|3934|4067|4283|4499|5028|5729|6279|6280|6356|6373|6505|6550|7052|7474|8671|9429|55151|55532|57214|60675|159371|255231|282679

CAV1|CD38|CD55|EDN1|EDN3|F2RL2|FPR1|GJA1|GNA15|CXCR2|CXCL10|LCN2|LYN|CXCL9|MT1M|P2RY1|PTGDR|S100A8|S100A9|CCL11|CXCL11|SLC1A1|SLC9A3|TGM2|WNT5A|SLC4A4|ABCG2|TMEM38B|SLC30A10|CEMIP|PROK2|SLC35G1|MCOLN2|AQP11 -7.208586004 0 8 0 0 -12.34993116 42.03283582

1 -9.475725266 GO:0006875 M1 1 0 GO Biological Processes 19 cellular metal ion homeostasis -9.475725266 3.770306454 8.08392027 28162 576 402 31 7.711442786 1.330542411

857|952|1604|1906|1908|2151|2357|2697|2769|3579|3627|3934|4067|4283|4499|5028|5729|6279|6280|6356|6373|6505|7052|7474|9429|55151|55532|57214|60675|159371|255231

CAV1|CD38|CD55|EDN1|EDN3|F2RL2|FPR1|GJA1|GNA15|CXCR2|CXCL10|LCN2|LYN|CXCL9|MT1M|P2RY1|PTGDR|S100A8|S100A9|CCL11|CXCL11|SLC1A1|TGM2|WNT5A|ABCG2|TMEM38B|SLC30A10|CEMIP|PROK2|SLC35G1|MCOLN2 -7.014203353 0 8 0 0 -12.34993116 42.03283582

1 -8.191438553 GO:0051480 M1 1 0 GO Biological Processes 19 regulation of cytosolic calcium ion concentration -8.191438553 4.467257913 7.797724412 28162 345 402 22 5.472636816 1.134394361

857|952|1604|1906|2151|2357|2697|2769|3579|3627|4067|4283|5028|5729|6373|7052|7474|55151|57214|60675|159371|255231

CAV1|CD38|CD55|EDN1|F2RL2|FPR1|GJA1|GNA15|CXCR2|CXCL10|LYN|CXCL9|P2RY1|PTGDR|CXCL11|TGM2|WNT5A|TMEM38B|CEMIP|PROK2|SLC35G1|MCOLN2 -5.868731779 0 8 0 0 -12.34993116 42.03283582

1 -7.921862181 GO:0055074 M1 1 0 GO Biological Processes 19 calcium ion homeostasis -7.921862181 3.849160789 7.373186798 28162 455 402 25

6.218905473 1.204485566
857|952|1604|1906|1908|2151|2357|2697|2769|3579|3627|4067|4283|5028|5729|5967|6356|6373|7052|7474|55151|57214|60675|159371|255231
CAV1|CD38|CD55|EDN1|EDN3|F2RL2|FPR1|GJA1|GNA15|CXCR2|CXCL10|LYN|CXCL9|P2RY1|PTGDR|REG1A|CCL11|CXCL11|TGM2|WNT5A|TMEM38B|CEMIP|PROK2|SLC35G1|MCOLN2
-5.638307532 0 8 0 0 -12.34993116 42.03283582
1 -7.584167357 GO:0007204 M1 1 0 GO Biological Processes 19 positive regulation of cytosolic calcium ion concentration -7.584167357 4.519659766 7.498608318 28162
310 402 20 4.975124378 1.084445172
857|952|1604|1906|2151|2357|2697|2769|3579|3627|4067|4283|5028|5729|6373|7052|55151|57214|60675|255231
CAV1|CD38|CD55|EDN1|F2RL2|FPR1|GJA1|GNA15|CXCR2|CXCL10|LYN|CXCL9|P2RY1|PTGDR|CXCL11|TGM2|TMEM38B|CEMIP|PROK2|MCOLN2 -5.346744429 0 8 0 0
-12.34993116 42.03283582
1 -7.511754875 GO:0006874 M1 1 0 GO Biological Processes 19 cellular calcium ion homeostasis -7.511754875 3.795289916 7.136204281 28162 443 402 24
5.970149254 1.181714164
857|952|1604|1906|1908|2151|2357|2697|2769|3579|3627|4067|4283|5028|5729|6356|6373|7052|7474|55151|57214|60675|159371|255231
CAV1|CD38|CD55|EDN1|EDN3|F2RL2|FPR1|GJA1|GNA15|CXCR2|CXCL10|LYN|CXCL9|P2RY1|PTGDR|CCL11|CXCL11|TGM2|WNT5A|TMEM38B|CEMIP|PROK2|SLC35G1|MCOLN2
-5.294076005 0 8 0 0 -12.34993116 42.03283582
1 -6.693314204 GO:0051235 M1 1 0 GO Biological Processes 19 maintenance of location -6.693314204 4.172538561 6.85759084 28162 319 402 19 4.726368159
1.058368993
288|718|857|2697|3553|3627|3934|4067|4283|5468|6279|6280|6373|11015|55151|57214|85477|130399|255231
ANK3|C3|CAV1|GJA1|IL1B|CXCL10|LCN2|LYN|CXCL9|PPARG|S100A8|S100A9|CXCL11|KDELR3|TMEM38B|CEMIP|SCIN|ACVR1C|MCOLN2 -4.55481658 0 8 0 0
-12.34993116 42.03283582
1 -6.683448526 GO:0034762 M1 1 0 GO Biological Processes 19 regulation of transmembrane transport -6.683448526 3.22375732 6.425516005 28162 565 402
26 6.467661692 1.226708902
288|718|857|1906|1908|2040|2697|3553|3627|3752|4067|4283|4318|5174|5243|6373|7057|10008|10891|23433|23596|55151|55198|57214|57628|140738
ANK3|C3|CAV1|EDN1|EDN3|STOM|GJA1|IL1B|CXCL10|KCND3|LYN|CXCL9|MMP9|PDZK1|ABCB1|CXCL11|THBS1|KCNE3|PPARGC1A|RHOQ|OPN3|TMEM38B|APPL2|CEMIP|DPP10|TMEM37 -4.547623492 0 8 0 0 -12.34993116 42.03283582
1 -6.423097763 GO:0051651 M1 1 0 GO Biological Processes 19 maintenance of location in cell -6.423097763 5.027851175 7.033308678 28162 209 402 15
3.731343284 0.945283402
288|857|2697|3627|3934|4067|4283|6279|6280|6373|11015|55151|57214|85477|255231
ANK3|CAV1|GJA1|CXCL10|LCN2|LYN|CXCL9|S100A8|S100A9|CXCL11|KDELR3|TMEM38B|

CEMIP|SCIN|MCOLN2 -4.320597792 0 8 0 0 -12.34993116 42.03283582
1 -6.20582016 GO:0034765 M1 1 0 GO Biological Processes 19 regulation of ion transmembrane transport -6.20582016 3.127443141 6.119402668 28162 560 402 25 6.218905473 1.204485566
288|718|857|1906|1908|2040|3553|3627|3752|4067|4283|4318|5174|5243|6373|7057|10008|10891|23433|23596|55151|55198|57214|57628|140738
ANK3|C3|CAV1|EDN1|EDN3|STOM|IL1B|CXCL10|KCND3|LYN|CXCL9|MMP9|PDZK1|ABCB1|CXCL11|THBS1|KCNE3|PPARGC1A|RHOQ|OPN3|TMEM38B|APPL2|CEMIP|DPP10|TMEM37 -4.136561123 0 8 0 0 -12.34993116 42.03283582
1 -4.548790617 GO:0048248 M1 1 0 GO Molecular Functions 21 CXCR3 chemokine receptor binding -4.548790617 42.03283582 11.04207458 28162 5 402 3 0.746268657 0.429247719 3627|4283|6373 CXCL10|CXCL9|CXCL11 -2.738597074 0 8 0 0 -12.34993116 42.03283582
1 -4.33032624 GO:0034764 M1 1 0 GO Biological Processes 19 positive regulation of transmembrane transport -4.33032624 4.061143558 5.319446599 28162 207 402 12 2.985074627 0.848757934
288|718|1908|3627|4283|5174|5243|6373|10008|23433|23596|57214
ANK3|C3|EDN3|CXCL10|CXCL9|PDZK1|ABCB1|CXCL11|KCNE3|RHOQ|OPN3|CEMIP -2.551687511 0 8 0 0 -12.34993116 42.03283582
1 -4.33032624 GO:0034767 M1 1 0 GO Biological Processes 19 positive regulation of ion transmembrane transport -4.33032624 4.061143558 5.319446599 28162 207 402 12 2.985074627 0.848757934
288|718|1908|3627|4283|5174|5243|6373|10008|23433|23596|57214
ANK3|C3|EDN3|CXCL10|CXCL9|PDZK1|ABCB1|CXCL11|KCNE3|RHOQ|OPN3|CEMIP -2.551687511 0 8 0 0 -12.34993116 42.03283582
1 -3.898106537 GO:1904062 M1 1 0 GO Biological Processes 19 regulation of cation transmembrane transport -3.898106537 3.090649693 4.667172859 28162 340 402 15 3.731343284 0.945283402
288|857|1908|2040|3627|4067|4283|4318|5174|6373|10008|10891|55151|57214|57628
ANK3|CAV1|EDN3|STOM|CXCL10|LYN|CXCL9|MMP9|PDZK1|CXCL11|KCNE3|PPARGC1A|TMEM38B|CEMIP|DPP10-2.206192101 0 8 0 0 -12.34993116 42.03283582
1 -3.365535713 GO:0010522 M1 1 0 GO Biological Processes 19 regulation of calcium ion transport into cytosol -3.365535713 5.161927206 4.889738311 28162 95 402 7 1.741293532 0.652391792 857|3627|4067|4283|6373|55151|57214
CAV1|CXCL10|LYN|CXCL9|CXCL11|TMEM38B|CEMIP -1.77187141 0 8 0 0 -12.34993116 42.03283582
1 -3.114658621 GO:0051279 M1 1 0 GO Biological Processes 19 regulation of release of sequestered calcium ion into cytosol -3.114658621 5.530636292 4.759358089 28162 76 402 6 1.492537313 0.604761504 3627|4067|4283|6373|55151|57214
CXCL10|LYN|CXCL9|CXCL11|TMEM38B|CEMIP -1.563220316 0 8 0 0 -12.34993116 42.03283582
1 -3.049021401 GO:0060401 M1 1 0 GO Biological Processes 19 cytosolic calcium ion transport -3.049021401 3.644465534 4.198504311 28162 173 402 9

2.23880597 0.737867616 857|3627|4067|4283|6373|55151|57214|159371|255231
CAV1|CXCL10|LYN|CXCL9|CXCL11|TMEM38B|CEMIP|SLC35G1|MCOLN2 -1.514984205 0
8 0 0 -12.34993116 42.03283582
1 -3.024546631 GO:0010524 M1 1 0 GO Biological Processes 19 positive regulation of calcium ion transport into cytosol -3.024546631 6.60893645 4.918391422 28162
53 402 5 1.243781095 0.552765814 857|3627|4283|6373|57214
CAV1|CXCL10|CXCL9|CXCL11|CEMIP -1.499355638 0 8 0 0 -12.34993116
42.03283582
1 -3.00931632 GO:1904064 M1 1 0 GO Biological Processes 19 positive regulation of cation transmembrane transport -3.00931632 3.974736248 4.261314018 28162
141 402 8 1.990049751 0.696552772
288|1908|3627|4283|5174|6373|10008|57214
ANK3|EDN3|CXCL10|CXCL9|PDZK1|CXCL11|KCNE3|CEMIP -1.488026201 0 8 0
0 -12.34993116 42.03283582
1 -2.939387393 GO:0051209 M1 1 0 GO Biological Processes 19 release of sequestered calcium ion into cytosol -2.939387393 4.378420398 4.311055372 28162
112 402 7 1.741293532 0.652391792 3627|4067|4283|6373|55151|57214|255231
CXCL10|LYN|CXCL9|CXCL11|TMEM38B|CEMIP|MCOLN2 -1.429689558 0 8 0 0
-12.34993116 42.03283582
1 -2.916870755 GO:0051283 M1 1 0 GO Biological Processes 19 negative regulation of sequestering of calcium ion -2.916870755 4.339673315 4.280670984
28162 113 402 7 1.741293532 0.652391792
3627|4067|4283|6373|55151|57214|255231
CXCL10|LYN|CXCL9|CXCL11|TMEM38B|CEMIP|MCOLN2 -1.413956157 0 8 0 0
-12.34993116 42.03283582
1 -2.872583926 GO:0051282 M1 1 0 GO Biological Processes 19 regulation of sequestering of calcium ion -2.872583926 4.264200735 4.22094704 28162 115 402
7 1.741293532 0.652391792 3627|4067|4283|6373|55151|57214|255231
CXCL10|LYN|CXCL9|CXCL11|TMEM38B|CEMIP|MCOLN2 -1.378269499 0 8 0 0
-12.34993116 42.03283582
1 -2.800398308 GO:0060402 M1 1 0 GO Biological Processes 19 calcium ion transport into cytosol -2.800398308 3.687090861 3.997375289 28162 152 402 8
1.990049751 0.696552772 857|3627|4067|4283|6373|55151|57214|255231
CAV1|CXCL10|LYN|CXCL9|CXCL11|TMEM38B|CEMIP|MCOLN2 -1.319853157 0 8 0
0 -12.34993116 42.03283582
1 -2.786873647 GO:0051208 M1 1 0 GO Biological Processes 19 sequestering of calcium ion -2.786873647 4.120866257 4.105478048 28162 119 402 7
1.741293532 0.652391792 3627|4067|4283|6373|55151|57214|255231
CXCL10|LYN|CXCL9|CXCL11|TMEM38B|CEMIP|MCOLN2 -1.307505447 0 8 0 0
-12.34993116 42.03283582
1 -2.735633805 GO:0030001 M1 1 0 GO Biological Processes 19 metal ion transport -2.735633805 2.30632844 3.495796857 28162 486 402 16 3.980099502
0.975022334
857|2697|3627|3752|4067|4283|6373|8671|10008|55151|55532|57214|140738|14080

3|159371|255231
CAV1|GJA1|CXCL10|KCND3|LYN|CXCL9|CXCL11|SLC4A4|KCNE3|TMEM38B|SLC30A10|CEMIP|TMEM37|TRPM6|SLC35G1|MCOLN2 -1.274675545 0 8 0 0 -12.34993116 42.03283582
1 -2.603613649 GO:0051281 M1 1 0 GO Biological Processes 19 positive regulation of release of sequestered calcium ion into cytosol -2.603613649 7.005472637 4.573844738 28162 40 402 4 0.995024876 0.495031064
3627|4283|6373|57214CXCL10|CXCL9|CXCL11|CEMIP -1.168884912 0 8 0 0 -12.34993116 42.03283582
1 -2.512403527 GO:0010959 M1 1 0 GO Biological Processes 19 regulation of metal ion transport -2.512403527 2.83622374 3.488049105 28162 247 402 10 2.487562189 0.776790586
857|2697|3627|4067|4283|6373|10008|55151|55532|57214
CAV1|GJA1|CXCL10|LYN|CXCL9|CXCL11|KCNE3|TMEM38B|SLC30A10|CEMIP -1.0987556 0 8 0 0 -12.34993116 42.03283582
1 -2.495668792 GO:0097553 M1 1 0 GO Biological Processes 19 calcium ion transmembrane import into cytosol -2.495668792 3.659575258 3.713607163 28162 134 402 7 1.741293532 0.652391792 3627|4067|4283|6373|55151|57214|255231
CXCL10|LYN|CXCL9|CXCL11|TMEM38B|CEMIP|MCOLN2 -1.086175878 0 8 0 0 -12.34993116 42.03283582
1 -2.485106577 GO:1904427 M1 1 0 GO Biological Processes 19 positive regulation of calcium ion transmembrane transport -2.485106577 5.003909026 4.036167893 28162 70 402 5 1.243781095 0.552765814 3627|4283|6373|10008|57214
CXCL10|CXCL9|CXCL11|KCNE3|CEMIP -1.079101992 0 8 0 0 -12.34993116 42.03283582
1 -2.340166283 GO:0070588 M1 1 0 GO Biological Processes 19 calcium ion transmembrane transport -2.340166283 2.534874967 3.237890517 28162 304 402 11 2.736318408 0.813665012
3627|4067|4283|6373|10008|55151|57214|140738|140803|159371|255231
CXCL10|LYN|CXCL9|CXCL11|KCNE3|TMEM38B|CEMIP|TMEM37|TRPM6|SLC35G1|MCOLN2 -0.965281498 0 8 0 0 -12.34993116 42.03283582
1 -2.307948214 GO:1903169 M1 1 0 GO Biological Processes 19 regulation of calcium ion transmembrane transport -2.307948214 3.381952307 3.460457977 28162 145 402 7 1.741293532 0.652391792 3627|4067|4283|6373|10008|55151|57214
CXCL10|LYN|CXCL9|CXCL11|KCNE3|TMEM38B|CEMIP -0.941754182 0 8 0 0 -12.34993116 42.03283582
1 -2.198623359 GO:0006816 M1 1 0 GO Biological Processes 19 calcium ion transport -2.198623359 2.226678344 3.007216644 28162 409 402 13 3.233830846 0.882281956
857|2697|3627|4067|4283|6373|10008|55151|57214|140738|140803|159371|255231
CAV1|GJA1|CXCL10|LYN|CXCL9|CXCL11|KCNE3|TMEM38B|CEMIP|TMEM37|TRPM6|SLC35G1|MCOLN2 -0.855791233 0 8 0 0 -12.34993116 42.03283582
1 -2.152601885 GO:0051928 M1 1 0 GO Biological Processes 19 positive regulation of calcium ion transport -2.152601885 3.562104731 3.35617992 28162 118 402 6

1.492537313	0.604761504	857|3627|4283|6373|10008|57214
CAV1|CXCL10|CXCL9|CXCL11|KCNE3|CEMIP	-0.819949002 0	8	0	0
-12.34993116 42.03283582
1	-2.124838493 GO:0051924	M1	1	0	GO Biological Processes 19	regulation of calcium ion transport	-2.124838493 2.649128308	3.074549166	28162	238	402	9
2.23880597	0.737867616	857|2697|3627|4067|4283|6373|10008|55151|57214
CAV1|GJA1|CXCL10|LYN|CXCL9|CXCL11|KCNE3|TMEM38B|CEMIP	-0.799666466 0	8
0	0	-12.34993116 42.03283582
1	-11.84131436 GO:0015711	M1	1	0	GO Biological Processes 19	organic anion transport	-11.84131436 5.20300355	9.886727479	28162	377	402	28	6.965174129
1.269625794
301|366|759|762|1836|1906|2697|3553|4843|5320|6505|6519|6533|6566|8140|8671|9429|9963|10050|11254|23657|28231|84647|123264|151473|200931|206358|220963
ANXA1|AQP9|CA1|CA4|SLC26A2|EDN1|GJA1|IL1B|NOS2|PLA2G2A|SLC1A1|SLC3A1|SLC6A6|SLC16A1|SLC7A5|SLC4A4|ABCG2|SLC23A1|SLC17A4|SLC6A14|SLC7A11|SLCO4A1|PLA2G12B|SLC51B|SLC16A14|SLC51A|SLC36A1|SLC16A9 -9.167173039 0	9	1	1
-11.84131436 30.02345416
1	-11.83394444 GO:0046943	M1	1	0	GO Molecular Functions 21	carboxylic acid transmembrane transporter activity	-11.83394444 8.424302538	11.26163641	28162
158	402	19	4.726368159	1.058368993
366|1836|5243|6505|6519|6533|6566|8140|9429|9963|11254|23657|55089|123264|151473|200931|206358|220963|340024
AQP9|SLC26A2|ABCB1|SLC1A1|SLC3A1|SLC6A6|SLC16A1|SLC7A5|ABCG2|SLC23A1|SLC6A14|SLC7A11|SLC38A4|SLC51B|SLC16A14|SLC51A|SLC36A1|SLC16A9|SLC6A19
-9.167173039 0	9	0	0	-11.84131436 30.02345416
1	-11.78432514 GO:0005342	M1	1	0	GO Molecular Functions 21	organic acid transmembrane transporter activity	-11.78432514 8.371319503	11.21679673	28162
159	402	19	4.726368159	1.058368993
366|1836|5243|6505|6519|6533|6566|8140|9429|9963|11254|23657|55089|123264|151473|200931|206358|220963|340024
AQP9|SLC26A2|ABCB1|SLC1A1|SLC3A1|SLC6A6|SLC16A1|SLC7A5|ABCG2|SLC23A1|SLC6A14|SLC7A11|SLC38A4|SLC51B|SLC16A14|SLC51A|SLC36A1|SLC16A9|SLC6A19
-9.126508582 0	9	0	0	-11.84131436 30.02345416
1	-11.76346474 GO:0015849	M1	1	0	GO Biological Processes 19	organic acid transport	-11.76346474 5.604378109	10.04682363	28162	325	402	26	6.467661692
1.226708902
301|366|1836|1906|2182|2697|3553|5243|5320|5468|6505|6519|6533|6566|7057|8140|9429|9963|11254|23657|55089|84647|123264|200931|206358|340024
ANXA1|AQP9|SLC26A2|EDN1|ACSL4|GJA1|IL1B|ABCB1|PLA2G2A|PPARG|SLC1A1|SLC3A1|SLC6A6|SLC16A1|THBS1|SLC7A5|ABCG2|SLC23A1|SLC6A14|SLC7A11|SLC38A4|PLA2G12B|SLC51B|SLC51A|SLC36A1|SLC6A19	-9.114422107 0	9	0	0	-11.84131436 30.02345416
1	-11.37295311 GO:0046942	M1	1	0	GO Biological Processes 19	carboxylic acid transport	-11.37295311 5.899345378	10.00379539	28162	285	402	24	5.970149254
1.181714164

301|366|1836|1906|2697|3553|4843|5320|6505|6519|6533|6566|8140|9429|9963|10050|11254|23657|84647|123264|151473|200931|206358|220963

ANXA1|AQP9|SLC26A2|EDN1|GJA1|IL1B|NOS2|PLA2G2A|SLC1A1|SLC3A1|SLC6A6|SLC16A1|SLC7A5|ABCG2|SLC23A1|SLC17A4|SLC6A14|SLC7A11|PLA2G12B|SLC51B|SLC16A14|SLC51A|SLC36A1|SLC16A9 -8.749216337 0 9 0 0 -11.84131436 30.02345416

1 -11.16699963 GO:0008509 M1 1 0 GO Molecular Functions 21 anion transmembrane transporter activity -11.16699963 4.549008206 9.255831476 28162 462 402 30 7.462686567 1.31066897

360|366|1836|2555|2697|5243|6505|6519|6533|6550|6566|6584|8140|8671|9429|9963|10050|11254|22802|23657|28231|55089|80704|123264|151473|200931|206358|220963|282679|340024

AQP3|AQP9|SLC26A2|GABRA2|GJA1|ABCB1|SLC1A1|SLC3A1|SLC6A6|SLC9A3|SLC16A1|SLC22A5|SLC7A5|SLC4A4|ABCG2|SLC23A1|SLC17A4|SLC6A14|CLCA4|SLC7A11|SLCO4A1|SLC38A4|SLC19A3|SLC51B|SLC16A14|SLC51A|SLC36A1|SLC16A9|AQP11|SLC6A19 -8.559349681 0 9 0 0 -11.84131436 30.02345416

1 -9.342379551 GO:0098656 M1 1 0 GO Biological Processes 19 anion transmembrane transport -9.342379551 3.828127124 8.052961722 28162 549 402 30 7.462686567 1.31066897

360|366|718|1836|1906|2555|2697|3553|5243|6505|6519|6533|6550|6566|6584|7057|8140|8671|9963|11254|22802|23433|23596|23657|55089|55198|55670|80704|206358|340024

AQP3|AQP9|C3|SLC26A2|EDN1|GABRA2|GJA1|IL1B|ABCB1|SLC1A1|SLC3A1|SLC6A6|SLC9A3|SLC16A1|SLC22A5|THBS1|SLC7A5|SLC4A4|SLC23A1|SLC6A14|CLCA4|RHOQ|OPN3|SLC7A11|SLC38A4|APPL2|PEX26|SLC19A3|SLC36A1|SLC6A19 -6.891994004 0 9 0 0 -11.84131436 30.02345416

1 -8.184928449 GO:0015718 M1 1 0 GO Biological Processes 19 monocarboxylic acid transport -8.184928449 6.297054056 8.531331978 28162 178 402 16 3.980099502 0.975022334

301|366|1906|2697|3553|4843|5320|6505|6566|9429|23657|84647|123264|151473|200931|220963

ANXA1|AQP9|EDN1|GJA1|IL1B|NOS2|PLA2G2A|SLC1A1|SLC16A1|ABCG2|SLC7A11|PLA2G12B|SLC51B|SLC16A14|SLC51A|SLC16A9 -5.866299588 0 9 0 0 -11.84131436 30.02345416

1 -7.883100979 GO:0015291 M1 1 0 GO Molecular Functions 21 secondary active transmembrane transporter activity -7.883100979 5.254104478 7.964671039 28162 240 402 18 4.47761194 1.031484687

1836|6505|6533|6550|6566|6584|8140|8671|9963|10050|11254|23657|28231|55089|151473|206358|220963|340024

SLC26A2|SLC1A1|SLC6A6|SLC9A3|SLC16A1|SLC22A5|SLC7A5|SLC4A4|SLC23A1|SLC17A4|SLC6A14|SLC7A11|SLCO4A1|SLC38A4|SLC16A14|SLC36A1|SLC16A9|SLC6A19 -5.603274202 0 9 0 0 -11.84131436 30.02345416

1 -7.77344456 GO:0022804 M1 1 0 GO Molecular Functions 21 active transmembrane transporter activity -7.77344456 4.639385852 7.65188351 28162 302 402 20 4.975124378 1.084445172

1836|5243|6505|6533|6550|6566|6584|8140|8671|9429|9963|10050|11254|23657|28231|55089|151473|206358|220963|340024

SLC26A2|ABCB1|SLC1A1|SLC6A6|SLC9A3|SLC16A1|SLC22A5|SLC7A5|SLC4A4|ABCG2|SLC23A1|SLC17A4|SLC6A14|SLC7A11|SLCO4A1|SLC38A4|SLC16A14|SLC36A1|SLC16A9|SLC6A19

-5.504613168 0 9 0 0 -11.84131436 30.02345416

1 -7.636724399 GO:1901682 M1 1 0 GO Molecular Functions 21 sulfur compound transmembrane transporter activity -7.636724399 13.13526119 10.12594954 28162 48 402 9 2.23880597 0.737867616

1836|2697|6505|6519|6533|9429|23657|80704|206358

SLC26A2|GJA1|SLC1A1|SLC3A1|SLC6A6|ABCG2|SLC7A11|SLC19A3|SLC36A1

-5.392515481 0 9 0 0 -11.84131436 30.02345416

1 -7.554057277 GO:0072348 M1 1 0 GO Biological Processes 19 sulfur compound transport -7.554057277 12.86719464 10.00506331 28162 49 402 9 2.23880597 0.737867616 1836|2697|6505|6519|6533|9429|23657|80704|206358

SLC26A2|GJA1|SLC1A1|SLC3A1|SLC6A6|ABCG2|SLC7A11|SLC19A3|SLC36A1

-5.323315934 0 9 0 0 -11.84131436 30.02345416

1 -7.299425397 GO:1905039 M1 1 0 GO Biological Processes 19 carboxylic acid transmembrane transport -7.299425397 6.368611488 8.039122007 28162 154 402 14 3.482587065 0.914409651

366|5243|6505|6519|6533|6566|7057|8140|9963|11254|23657|55089|206358|340024

AQP9|ABCB1|SLC1A1|SLC3A1|SLC6A6|SLC16A1|THBS1|SLC7A5|SLC23A1|SLC6A14|SLC7A11|SLC38A4|SLC36A1|SLC6A19 -5.103701103 0 9 0 0 -11.84131436 30.02345416

1 -7.263911127 GO:1903825 M1 1 0 GO Biological Processes 19 organic acid transmembrane transport -7.263911127 6.327523672 8.003598118 28162 155 402 14 3.482587065 0.914409651

366|5243|6505|6519|6533|6566|7057|8140|9963|11254|23657|55089|206358|340024

AQP9|ABCB1|SLC1A1|SLC3A1|SLC6A6|SLC16A1|THBS1|SLC7A5|SLC23A1|SLC6A14|SLC7A11|SLC38A4|SLC36A1|SLC6A19 -5.074260981 0 9 0 0 -11.84131436 30.02345416

1 -6.821005364 GO:0015293 M1 1 0 GO Molecular Functions 21 symporter activity -6.821005364 6.368611488 7.745171014 28162 143 402 13 3.233830846 0.882281956

6505|6533|6566|6584|8671|9963|10050|11254|55089|151473|206358|220963|340024

SLC1A1|SLC6A6|SLC16A1|SLC22A5|SLC4A4|SLC23A1|SLC17A4|SLC6A14|SLC38A4|SLC16A14|SLC36A1|SLC16A9|SLC6A19 -4.666117324 0 9 0 0 -11.84131436 30.02345416

1 -6.516520352 GO:0008514 M1 1 0 GO Molecular Functions 21 organic anion transmembrane transporter activity -6.516520352 5.509922299 7.263592918 28162 178 402 14 3.482587065 0.914409651

1836|2697|6505|6519|6533|6566|8140|8671|9429|9963|11254|23657|28231|206358

SLC26A2|GJA1|SLC1A1|SLC3A1|SLC6A6|SLC16A1|SLC7A5|SLC4A4|ABCG2|SLC23A1|SLC6A14|SLC7A11|SLCO4A1|SLC36A1 -4.396394418 0 9 0 0 -11.84131436 30.02345416

1 -6.35431216 GO:0015175 M1 1 0 GO Molecular Functions 21 neutral amino acid transmembrane transporter activity -6.35431216 14.4230319 9.424275775 28162 34 402 7 1.741293532 0.652391792

6505|6533|8140|11254|23657|206358|340024

SLC1A1|SLC6A6|SLC7A5|SLC6A14|SLC7A11|SLC36A1|SLC6A19 -4.271117345 0 9 0 0 -11.84131436 30.02345416
1 -6.222637983 GO:0008028 M1 1 0 GO Molecular Functions 21 monocarboxylic acid transmembrane transporter activity -6.222637983 9.137573005 8.144186596 28162 69 402 9 2.23880597 0.737867616 6505|6533|6566|9429|23657|123264|151473|200931|220963 SLC1A1|SLC6A6|SLC16A1|ABCG2|SLC7A11|SLC51B|SLC16A14|SLC51A|SLC16A9 -4.151087149 0 9 0 0 -11.84131436 30.02345416
1 -5.579943804 GO:0015171 M1 1 0 GO Molecular Functions 21 amino acid transmembrane transporter activity -5.579943804 7.688933382 7.299490993 28162 82 402 9 2.23880597 0.737867616 6505|6519|6533|8140|11254|23657|55089|206358|340024 SLC1A1|SLC3A1|SLC6A6|SLC7A5|SLC6A14|SLC7A11|SLC38A4|SLC36A1|SLC6A19 -3.582179184 0 9 0 0 -11.84131436 30.02345416
1 -5.554980625 GO:0015804 M1 1 0 GO Biological Processes 19 neutral amino acid transport -5.554980625 11.1450701 8.104318665 28162 44 402 7 1.741293532 0.652391792 6505|6519|6533|8140|11254|206358|340024 SLC1A1|SLC3A1|SLC6A6|SLC7A5|SLC6A14|SLC36A1|SLC6A19 -3.562993844 0 9 0 0 -11.84131436 30.02345416
1 -5.010313434 GO:0003333 M1 1 0 GO Biological Processes 19 amino acid transmembrane transport -5.010313434 6.567630597 6.575716799 28162 96 402 9 2.23880597 0.737867616 6505|6519|6533|8140|11254|23657|55089|206358|340024 SLC1A1|SLC3A1|SLC6A6|SLC7A5|SLC6A14|SLC7A11|SLC38A4|SLC36A1|SLC6A19 -3.114087232 0 9 0 0 -11.84131436 30.02345416
1 -4.557718203 GO:0089718 M1 1 0 GO Biological Processes 19 amino acid import across plasma membrane -4.557718203 10.00781805 7.0301392 28162 42 402 6 1.492537313 0.604761504 6505|6533|8140|11254|23657|206358 SLC1A1|SLC6A6|SLC7A5|SLC6A14|SLC7A11|SLC36A1 -2.738597074 0 9 0 0 -11.84131436 30.02345416
1 -4.168434275 GO:0043090 M1 1 0 GO Biological Processes 19 amino acid import -4.168434275 8.578129759 6.389013606 28162 49 402 6 1.492537313 0.604761504 6505|6533|8140|11254|23657|206358 SLC1A1|SLC6A6|SLC7A5|SLC6A14|SLC7A11|SLC36A1 -2.42356601 0 9 0 0 -11.84131436 30.02345416
1 -4.132363721 GO:0006865 M1 1 0 GO Biological Processes 19 amino acid transport -4.132363721 4.578740286 5.341391706 28162 153 402 10 2.487562189 0.776790586 2697|6505|6519|6533|8140|11254|23657|55089|206358|340024 GJA1|SLC1A1|SLC3A1|SLC6A6|SLC7A5|SLC6A14|SLC7A11|SLC38A4|SLC36A1|SLC6A19 -2.393945492 0 9 0 0 -11.84131436 30.02345416
1 -4.013986523 GO:0000099 M1 1 0 GO Molecular Functions 21 sulfur amino acid transmembrane transporter activity -4.013986523 30.02345416 9.241610993 28162 7 402 3 0.746268657 0.429247719 6505|6519|23657 SLC1A1|SLC3A1|SLC7A11 -2.305460374 0 9 0 0 -11.84131436 30.02345416

1 -3.23873503 GO:0022858 M1 1 0 GO Molecular Functions 21 alanine transmembrane transporter activity -3.23873503 17.51368159 6.885296737 28162 12 402 3 0.746268657 0.429247719 6533|11254|206358 SLC6A6|SLC6A14|SLC36A1 -1.672477766 0 9 0 0 -11.84131436 30.02345416
1 -2.936968961 GO:0032328 M1 1 0 GO Biological Processes 19 alanine transport -2.936968961 14.01094527 6.065488101 28162 15 402 3 0.746268657 0.429247719 6533|11254|206358 SLC6A6|SLC6A14|SLC36A1 -1.429689558 0 9 0 0 -11.84131436 30.02345416
1 -2.813136226 GO:0015179 M1 1 0 GO Molecular Functions 21 L-amino acid transmembrane transporter activity -2.813136226 5.936841218 4.568000633 28162 59 402 5 1.243781095 0.552765814 6505|6519|8140|23657|206358 SLC1A1|SLC3A1|SLC7A5|SLC7A11|SLC36A1 -1.330819648 0 9 0 0 -11.84131436 30.02345416
1 -2.643726612 GO:0098739 M1 1 0 GO Biological Processes 19 import across plasma membrane -2.643726612 3.480980192 3.799065629 28162 161 402 8 1.990049751 0.696552772 6505|6533|6550|7057|8140|11254|23657|206358 SLC1A1|SLC6A6|SLC9A3|THBS1|SLC7A5|SLC6A14|SLC7A11|SLC36A1 -1.200970022 0 9 0 0 -11.84131436 30.02345416
1 -2.596714616 GO:0015807 M1 1 0 GO Biological Processes 19 L-amino acid transport -2.596714616 5.30717624 4.215699974 28162 66 402 5 1.243781095 0.552765814 6505|6519|8140|23657|206358 SLC1A1|SLC3A1|SLC7A5|SLC7A11|SLC36A1 -1.163045135 0 9 0 0 -11.84131436 30.02345416
1 -2.218198882 GO:0098657 M1 1 0 GO Biological Processes 19 import into cell -2.218198882 2.741271901 3.190853207 28162 230 402 9 2.23880597 0.737867616 6505|6533|6550|7057|8140|11254|23657|54492|206358 SLC1A1|SLC6A6|SLC9A3|THBS1|SLC7A5|SLC6A14|SLC7A11|NEURL1B|SLC36A1 -0.873646775 0 9 0 0 -11.84131436 30.02345416
1 -11.63848096 GO:0030155 M1 1 0 GO Biological Processes 19 regulation of cell adhesion -11.63848096 3.722253853 8.993048027 28162 734 402 39 9.701492537 1.476204539 240|288|301|857|1277|1604|1942|2633|3371|3373|3553|3557|3575|3620|4067|4321|4811|5105|5270|5328|5801|7045|7052|7057|7474|8876|10451|10562|10979|11005|11126|11148|26585|28984|29126|55824|124872|145741|284217 ALOX5|ANK3|ANXA1|CAV1|COL1A1|CD55|EFNA1|GBP1|TNC|HYAL1|IL1B|IL1RN|IL7R|IDO1|LYN|MMP12|NID1|PCK1|SERPINE2|PLAU|PTPRR|TGFBI|TGM2|THBS1|WNT5A|VNN1|VAV3|OLFM4|FERMT2|SPINK5|CD160|HHLA2|GREM1|RGCC|CD274|PAG1|B4GALNT2|C2CD4A|LAMA1-8.998038502 0 10 1 1 -11.63848096 6.145151436
1 -6.415584725 GO:0007159 M1 1 0 GO Biological Processes 19 leukocyte cell-cell adhesion -6.415584725 3.828127124 6.553535407 28162 366 402 20 4.975124378 1.084445172 240|301|857|1604|3553|3575|3620|4067|4478|5105|5175|6279|6280|6402|8876|11126|11148|29126|55824|57126 ALOX5|ANXA1|CAV1|CD55|IL1B|IL7R|IDO1|LYN|MSN|PCK1|PECAM1|S100A8|S100A9|SE

LL|VNN1|CD160|HHLA2|CD274|PAG1|CD177 -4.315545353 0 10 0 0
-11.63848096 6.145151436
1 -5.771624424 GO:0022407 M1 1 0 GO Biological Processes 19 regulation of cell-cell adhesion -5.771624424 3.351137252 5.974718693 28162 439 402 21
5.223880597 1.109770192
240|288|301|857|1604|3553|3557|3575|3620|4067|5105|5270|5801|7474|8876|11126|11148|28984|29126|55824|124872
ALOX5|ANK3|ANXA1|CAV1|CD55|IL1B|IL1RN|IL7R|IDO1|LYN|PCK1|SERPINE2|PTPRR|WNT5A|VNN1|CD160|HHLA2|RGCC|CD274|PAG1|B4GALNT2-3.754025559 0 10 0 0
-11.63848096 6.145151436
1 -5.757253122 GO:0050865 M1 1 0 GO Biological Processes 19 regulation of cell activation -5.757253122 2.877445317 5.749118568 28162 633 402 26 6.467661692
1.226708902
301|857|952|1604|3553|3575|3598|3620|4067|4332|5105|5270|5320|7056|7057|7474|8876|10451|11005|11126|11148|29126|51237|55824|57126|90865
ANXA1|CAV1|CD38|CD55|IL1B|IL7R|IL13RA2|IDO1|LYN|MNDA|PCK1|SERPINE2|PLA2G2A|THBD|THBS1|WNT5A|VNN1|VAV3|SPINK5|CD160|HHLA2|CD274|MZB1|PAG1|CD177|IL33
-3.743694235 0 10 0 0 -11.63848096 6.145151436
1 -5.506580061 GO:0070661 M1 1 0 GO Biological Processes 19 leukocyte proliferation -5.506580061 3.804889292 6.004964199 28162 313 402 17
4.228855721 1.003727321
301|952|1503|1604|3553|3575|3620|4067|4332|4478|10451|11148|23705|26585|29126|51237|90865
ANXA1|CD38|CTPS1|CD55|IL1B|IL7R|IDO1|LYN|MNDA|MSN|VAV3|HHLA2|CADM1|GREM1|CD274|MZB1|IL33 -3.524055408 0 10 0 0 -11.63848096 6.145151436
1 -5.417928405 GO:0045785 M1 1 0 GO Biological Processes 19 positive regulation of cell adhesion -5.417928405 3.296693006 5.741098545 28162 425 402 20
4.975124378 1.084445172
240|288|301|857|1604|3373|3553|3575|4067|4811|5105|7052|7474|8876|10451|10562|10979|11126|11148|29126
ALOX5|ANK3|ANXA1|CAV1|CD55|HYAL1|IL1B|IL7R|LYN|NID1|PCK1|TGM2|WNT5A|VNN1|VAV3|OLFM4|FERMT2|CD160|HHLA2|CD274 -3.459081851 0 10 0 0
-11.63848096 6.145151436
1 -5.237450532 GO:0002694 M1 1 0 GO Biological Processes 19 regulation of leukocyte activation -5.237450532 2.830493995 5.426080503 28162 594 402 24
5.970149254 1.181714164
301|857|952|1604|3553|3575|3598|3620|4067|4332|5105|5320|7057|7474|8876|10451|11005|11126|11148|29126|51237|55824|57126|90865
ANXA1|CAV1|CD38|CD55|IL1B|IL7R|IL13RA2|IDO1|LYN|MNDA|PCK1|PLA2G2A|THBS1|WNT5A|VNN1|VAV3|SPINK5|CD160|HHLA2|CD274|MZB1|PAG1|CD177|IL33
-3.306078401 0 10 0 0 -11.63848096 6.145151436
1 -4.803257647 GO:0046651 M1 1 0 GO Biological Processes 19 lymphocyte proliferation -4.803257647 3.713148041 5.520208038 28162 283 402 15
3.731343284 0.945283402

301|952|1503|1604|3553|3575|3620|4067|4332|4478|10451|11148|23705|29126|51237

ANXA1|CD38|CTPS1|CD55|IL1B|IL7R|IDO1|LYN|MNDA|MSN|VAV3|HHLA2|CADM1|CD274|MZB1 -2.939544848 0 10 0 0 -11.63848096 6.145151436

1 -4.749579006 GO:0032943 M1 1 0 GO Biological Processes 19 mononuclear cell proliferation -4.749579006 3.674198935 5.470018966 28162 286 402 15 3.731343284 0.945283402

301|952|1503|1604|3553|3575|3620|4067|4332|4478|10451|11148|23705|29126|51237

ANXA1|CD38|CTPS1|CD55|IL1B|IL7R|IDO1|LYN|MNDA|MSN|VAV3|HHLA2|CADM1|CD274|MZB1 -2.888704743 0 10 0 0 -11.63848096 6.145151436

1 -4.318341614 GO:0070663 M1 1 0 GO Biological Processes 19 regulation of leukocyte proliferation -4.318341614 3.778885655 5.213640069 28162 241 402 13 3.233830846 0.882281956

301|952|1604|3553|3620|4067|4332|10451|11148|26585|29126|51237|90865

ANXA1|CD38|CD55|IL1B|IDO1|LYN|MNDA|VAV3|HHLA2|GREM1|CD274|MZB1|IL33

-2.545266838 0 10 0 0 -11.63848096 6.145151436

1 -4.314219191 GO:0022409 M1 1 0 GO Biological Processes 19 positive regulation of cell-cell adhesion -4.314219191 3.553500613 5.130086972 28162 276 402 14 3.482587065 0.914409651

240|288|301|857|1604|3553|3575|4067|5105|7474|8876|11126|11148|29126

ALOX5|ANK3|ANXA1|CAV1|CD55|IL1B|IL7R|LYN|PCK1|WNT5A|VNN1|CD160|HHLA2|CD274 -2.543696332 0 10 0 0 -11.63848096 6.145151436

1 -4.128416113 GO:0002696 M1 1 0 GO Biological Processes 19 positive regulation of leukocyte activation -4.128416113 2.969901118 4.781084197 28162 401 402 17 4.228855721 1.003727321

301|857|952|1604|3553|3575|4067|5105|7057|7474|8876|10451|11126|11148|29126|57126|90865

ANXA1|CAV1|CD38|CD55|IL1B|IL7R|LYN|PCK1|THBS1|WNT5A|VNN1|VAV3|CD160|HHLA2|CD274|CD177|IL33 -2.391063638 0 10 0 0 -11.63848096 6.145151436

1 -3.986190811 GO:0050867 M1 1 0 GO Biological Processes 19 positive regulation of cell activation -3.986190811 2.890607641 4.652065867 28162 412 402 17 4.228855721 1.003727321

301|857|952|1604|3553|3575|4067|5105|7057|7474|8876|10451|11126|11148|29126|57126|90865

ANXA1|CAV1|CD38|CD55|IL1B|IL7R|LYN|PCK1|THBS1|WNT5A|VNN1|VAV3|CD160|HHLA2|CD274|CD177|IL33 -2.282616762 0 10 0 0 -11.63848096 6.145151436

1 -3.82384146 GO:1903039 M1 1 0 GO Biological Processes 19 positive regulation of leukocyte cell-cell adhesion -3.82384146 3.59255007 4.792283428 28162 234 402 12 2.985074627 0.848757934

240|301|857|1604|3553|3575|4067|5105|8876|11126|11148|29126

ALOX5|ANXA1|CAV1|CD55|IL1B|IL7R|LYN|PCK1|VNN1|CD160|HHLA2|CD274

-2.143576956 0 10 0 0 -11.63848096 6.145151436

1 -3.595646346 GO:0050870 M1 1 0 GO Biological Processes 19 positive regulation

of T cell activation -3.595646346 3.634915047 4.634159463 28162 212 402 11 2.736318408 0.813665012 301|857|1604|3553|3575|4067|5105|8876|11126|11148|29126 ANXA1|CAV1|CD55|IL1B|IL7R|LYN|PCK1|VNN1|CD160|HHLA2|CD274 -1.961124251 0 10 0 0 -11.63848096 6.145151436

1 -3.54365849 GO:0050863 M1 1 0 GO Biological Processes 19 regulation of T cell activation -3.54365849 2.999284921 4.376029952 28162 327 402 14 3.482587065 0.914409651 301|857|1604|3553|3575|3620|4067|5105|8876|11005|11126|11148|29126|55824 ANXA1|CAV1|CD55|IL1B|IL7R|IDO1|LYN|PCK1|VNN1|SPINK5|CD160|HHLA2|CD274|PAG1 -1.918279774 0 10 0 0 -11.63848096 6.145151436

1 -3.503644746 GO:1903037 M1 1 0 GO Biological Processes 19 regulation of leukocyte cell-cell adhesion -3.503644746 2.972018694 4.336337953 28162 330 402 14 3.482587065 0.914409651 240|301|857|1604|3553|3575|3620|4067|5105|8876|11126|11148|29126|55824 ALOX5|ANXA1|CAV1|CD55|IL1B|IL7R|IDO1|LYN|PCK1|VNN1|CD160|HHLA2|CD274|PAG1 -1.884796897 0 10 0 0 -11.63848096 6.145151436

1 -3.441570819 GO:0050670 M1 1 0 GO Biological Processes 19 regulation of lymphocyte proliferation -3.441570819 3.486886833 4.466409299 28162 221 402 11 2.736318408 0.813665012 301|952|1604|3553|3620|4067|4332|10451|11148|29126|51237 ANXA1|CD38|CD55|IL1B|IDO1|LYN|MNDA|VAV3|HHLA2|CD274|MZB1 -1.840192379 0 10 0 0 -11.63848096 6.145151436

1 -3.408477865 GO:0032944 M1 1 0 GO Biological Processes 19 regulation of mononuclear cell proliferation -3.408477865 3.455614305 4.430313809 28162 223 402 11 2.736318408 0.813665012 301|952|1604|3553|3620|4067|4332|10451|11148|29126|51237 ANXA1|CD38|CD55|IL1B|IDO1|LYN|MNDA|VAV3|HHLA2|CD274|MZB1 -1.810201542 0 10 0 0 -11.63848096 6.145151436

1 -3.376544548 GO:0051249 M1 1 0 GO Biological Processes 19 regulation of lymphocyte activation -3.376544548 2.482254084 4.056979978 28162 508 402 18 4.47761194 1.031484687 301|857|952|1604|3553|3575|3620|4067|4332|5105|8876|10451|11005|11126|11148|29126|51237|55824 ANXA1|CAV1|CD38|CD55|IL1B|IL7R|IDO1|LYN|MNDA|PCK1|VNN1|VAV3|SPINK5|CD160|HHLA2|CD274|MZB1|PAG1 -1.780580357 0 10 0 0 -11.63848096 6.145151436

1 -3.268492546 GO:0042110 M1 1 0 GO Biological Processes 19 T cell activation -3.268492546 2.507221786 3.986698101 28162 475 402 17 4.228855721 1.003727321 301|857|1503|1604|3553|3575|3620|4067|4478|5105|8876|11005|11126|11148|23705|29126|55824 ANXA1|CAV1|CTPS1|CD55|IL1B|IL7R|IDO1|LYN|MSN|PCK1|VNN1|SPINK5|CD160|HHLA2|CADM1|CD274|PAG1 -1.692801614 0 10 0 0 -11.63848096 6.145151436

1 -3.229317514 GO:0046649 M1 1 0 GO Biological Processes 19 lymphocyte

activation -3.229317514 2.136947887 3.808157403 28162 754 402 23 5.721393035 1.158362377 301|857|952|1503|1604|3553|3575|3620|4067|4332|4478|5105|5450|8876|10451|11005|11126|11148|23705|29126|51237|55824|57823 ANXA1|CAV1|CD38|CTPS1|CD55|IL1B|IL7R|IDO1|LYN|MNDA|MSN|PCK1|POU2AF1|VNN1|VAV3|SPINK5|CD160|HHLA2|CADM1|CD274|MZB1|PAG1|SLAMF7 -1.6637775 0 10 0 0 -11.63848096 6.145151436

1 -2.880660695 GO:0031295 M1 1 0 GO Biological Processes 19 T cell costimulation -2.880660695 6.145151436 4.679194811 28162 57 402 5 1.243781095 0.552765814 857|4067|11126|11148|29126 CAV1|LYN|CD160|HHLA2|CD274 -1.383294291 0 10 0 0 -11.63848096 6.145151436

1 -2.874016637 GO:0070665 M1 1 0 GO Biological Processes 19 positive regulation of leukocyte proliferation -2.874016637 3.786741966 4.090415121 28162 148 402 8 1.990049751 0.696552772 301|952|1604|3553|4067|10451|11148|29126 ANXA1|CD38|CD55|IL1B|LYN|VAV3|HHLA2|CD274 -1.378483992 0 10 0 0 -11.63848096 6.145151436

1 -2.813136226 GO:0031294 M1 1 0 GO Biological Processes 19 lymphocyte costimulation -2.813136226 5.936841218 4.568000633 28162 59 402 5 1.243781095 0.552765814 857|4067|11126|11148|29126 CAV1|LYN|CD160|HHLA2|CD274 -1.330819648 0 10 0 0 -11.63848096 6.145151436

1 -2.70415097 GO:0051251 M1 1 0 GO Biological Processes 19 positive regulation of lymphocyte activation -2.70415097 2.55817821 3.560411528 28162 356 402 13 3.233830846 0.882281956 301|857|952|1604|3553|3575|4067|5105|8876|10451|11126|11148|29126 ANXA1|CAV1|CD38|CD55|IL1B|IL7R|LYN|PCK1|VNN1|VAV3|CD160|HHLA2|CD274 -1.249155064 0 10 0 0 -11.63848096 6.145151436

1 -2.673751649 GO:0042098 M1 1 0 GO Biological Processes 19 T cell proliferation -2.673751649 3.21679866 3.747714001 28162 196 402 9 2.23880597 0.737867616 301|1503|1604|3553|3620|4478|11148|23705|29126 ANXA1|CTPS1|CD55|IL1B|IDO1|MSN|HHLA2|CADM1|CD274 -1.226652078 0 10 0 0 -11.63848096 6.145151436

1 -2.477774174 GO:0050671 M1 1 0 GO Biological Processes 19 positive regulation of lymphocyte proliferation -2.477774174 3.632467293 3.689511713 28162 135 402 7 1.741293532 0.652391792 301|952|1604|3553|10451|11148|29126 ANXA1|CD38|CD55|IL1B|VAV3|HHLA2|CD274 -1.075230121 0 10 0 0 -11.63848096 6.145151436

1 -2.460052904 GO:0032946 M1 1 0 GO Biological Processes 19 positive regulation of mononuclear cell proliferation -2.460052904 3.605757975 3.665644152 28162 136 402 7 1.741293532 0.652391792 301|952|1604|3553|10451|11148|29126 ANXA1|CD38|CD55|IL1B|VAV3|HHLA2|CD274 -1.060908724 0 10 0 0 -11.63848096 6.145151436

1 -11.41707142 GO:0010817 M1 1 0 GO Biological Processes 19 regulation of

hormone levels	-11.41707142 4.369885465 9.269609536 28162 513 402 32
7.960199005 1.350009335
126|183|240|301|952|1513|1906|1908|2182|2690|2697|3284|3294|3553|3557|4067|4843|5028|5122|6566|6696|8140|10891|25825|28231|29785|50506|79154|79644|130399|195814|405753
ADH1C|AGT|ALOX5|ANXA1|CD38|CTSK|EDN1|EDN3|ACSL4|GHR|GJA1|HSD3B2|HSD17B2|IL1B|IL1RN|LYN|NOS2|P2RY1|PCSK1|SLC16A1|SPP1|SLC7A5|PPARGC1A|BACE2|SLCO4A1|CYP2S1|DUOX2|DHRS11|SRD5A3|ACVR1C|SDR16C5|DUOXA2 -8.78506213 0 11 1 1
-11.41707142 4.369885465
1 -6.111102421 GO:0009914 M1 1 0 GO Biological Processes 19 hormone transport -6.111102421 4.003127221 6.450110161 28162 315 402 18 4.47761194
1.031484687
183|240|301|952|1906|1908|2182|2697|3553|3557|4067|4843|5028|6566|6696|8140|28231|130399
AGT|ALOX5|ANXA1|CD38|EDN1|EDN3|ACSL4|GJA1|IL1B|IL1RN|LYN|NOS2|P2RY1|SLC16A1|SPP1|SLC7A5|SLCO4A1|ACVR1C -4.061942859 0 11 0 0 -11.41707142
4.369885465
1 -5.028229137 GO:0046879 M1 1 0 GO Biological Processes 19 hormone secretion -5.028229137 3.675002039 5.652417039 28162 305 402 16 3.980099502
0.975022334
183|240|301|952|1906|1908|2182|2697|3553|3557|4067|4843|5028|6566|6696|130399
AGT|ALOX5|ANXA1|CD38|EDN1|EDN3|ACSL4|GJA1|IL1B|IL1RN|LYN|NOS2|P2RY1|SLC16A1|SPP1|ACVR1C -3.125820702 0 11 0 0 -11.41707142 4.369885465
1 -4.542510752 GO:0046883 M1 1 0 GO Biological Processes 19 regulation of hormone secretion -4.542510752 3.729148932 5.351039094 28162 263 402 14
3.482587065 0.914409651
183|240|301|952|1906|1908|2182|2697|3553|4843|5028|6566|6696|130399
AGT|ALOX5|ANXA1|CD38|EDN1|EDN3|ACSL4|GJA1|IL1B|NOS2|P2RY1|SLC16A1|SPP1|ACVR1C -2.733574212 0 11 0 0 -11.41707142 4.369885465
1 -3.618724124 GO:0051046 M1 1 0 GO Biological Processes 19 regulation of secretion -3.618724124 2.388229308 4.192273669 28162 616 402 21 5.223880597
1.109770192
183|240|301|952|1906|1908|2182|2697|3553|3598|4067|4843|5028|6279|6566|6696|7032|28984|57126|85477|130399
AGT|ALOX5|ANXA1|CD38|EDN1|EDN3|ACSL4|GJA1|IL1B|IL13RA2|LYN|NOS2|P2RY1|S100A8|SLC16A1|SPP1|TFF2|RGCC|CD177|SCIN|ACVR1C -1.974862003 0 11 0 0
-11.41707142 4.369885465
1 -3.607039447 GO:0030072 M1 1 0 GO Biological Processes 19 peptide hormone secretion -3.607039447 3.403468488 4.565574405 28162 247 402 12 2.985074627
0.848757934 240|301|952|1906|1908|2182|2697|3553|3557|4843|6566|130399
ALOX5|ANXA1|CD38|EDN1|EDN3|ACSL4|GJA1|IL1B|IL1RN|NOS2|SLC16A1|ACVR1C
-1.96829677 0 11 0 0 -11.41707142 4.369885465
1 -3.248106822 GO:0023061 M1 1 0 GO Biological Processes 19 signal release

-3.248106822 2.496709325 3.967361021 28162 477 402 17 4.228855721 1.003727321
183|240|301|952|1906|1908|2182|2697|3553|3557|4067|4843|5028|6566|6696|27163|130399
AGT|ALOX5|ANXA1|CD38|EDN1|EDN3|ACSL4|GJA1|IL1B|IL1RN|LYN|NOS2|P2RY1|SLC16A1|SPP1|NAAA|ACVR1C -1.678245434 0 11 0 0 -11.41707142 4.369885465
1 -3.078955437 GO:0030073 M1 1 0 GO Biological Processes 19 insulin secretion -3.078955437 3.384286298 4.143250194 28162 207 402 10 2.487562189 0.776790586 240|301|952|2182|2697|3553|3557|4843|6566|130399
ALOX5|ANXA1|CD38|ACSL4|GJA1|IL1B|IL1RN|NOS2|SLC16A1|ACVR1C -1.537522191 0 11 0 0 -11.41707142 4.369885465
1 -2.925865338 GO:0051047 M1 1 0 GO Biological Processes 19 positive regulation of secretion -2.925865338 2.849683784 3.843179954 28162 295 402 12 2.985074627 0.848757934
952|1906|1908|2182|2697|3553|5028|6279|6696|28984|57126|85477
CD38|EDN1|EDN3|ACSL4|GJA1|IL1B|P2RY1|S100A8|SPP1|RGCC|CD177|SCIN -1.421085478 0 11 0 0 -11.41707142 4.369885465
1 -2.858924491 GO:1903530 M1 1 0 GO Biological Processes 19 regulation of secretion by cell -2.858924491 2.235789139 3.567569587 28162 564 402 18 4.47761194 1.031484687
183|240|301|952|1906|1908|2182|2697|3553|3598|4067|4843|5028|6566|6696|28984|57126|130399
AGT|ALOX5|ANXA1|CD38|EDN1|EDN3|ACSL4|GJA1|IL1B|IL13RA2|LYN|NOS2|P2RY1|SLC16A1|SPP1|RGCC|CD177|ACVR1C -1.365824874 0 11 0 0 -11.41707142 4.369885465
1 -2.689918161 GO:0002790 M1 1 0 GO Biological Processes 19 peptide secretion -2.689918161 2.445800921 3.509057318 28162 401 402 14 3.482587065 0.914409651
240|301|952|1906|1908|2182|2697|3553|3557|4843|6279|6566|26286|130399
ALOX5|ANXA1|CD38|EDN1|EDN3|ACSL4|GJA1|IL1B|IL1RN|NOS2|S100A8|SLC16A1|ARFGAP3|ACVR1C -1.237911023 0 11 0 0 -11.41707142 4.369885465
1 -2.684771263 GO:0046887 M1 1 0 GO Biological Processes 19 positive regulation of hormone secretion -2.684771263 3.954702295 3.968061443 28162 124 402 7 1.741293532 0.652391792 952|1906|1908|2182|2697|5028|6696
CD38|EDN1|EDN3|ACSL4|GJA1|P2RY1|SPP1 -1.237125753 0 11 0 0 -11.41707142 4.369885465
1 -2.660553226 GO:0051048 M1 1 0 GO Biological Processes 19 negative regulation of secretion -2.660553226 3.502736318 3.820391706 28162 160 402 8 1.990049751 0.696552772 301|1906|2697|3553|3598|5028|7032|130399
ANXA1|EDN1|GJA1|IL1B|IL13RA2|P2RY1|TFF2|ACVR1C -1.214543479 0 11 0 0 -11.41707142 4.369885465
1 -2.407903264 GO:1903531 M1 1 0 GO Biological Processes 19 negative regulation of secretion by cell -2.407903264 3.52793586 3.595368998 28162 139 402 7 1.741293532 0.652391792 301|1906|2697|3553|3598|5028|130399

ANXA1|EDN1|GJA1|IL1B|IL13RA2|P2RY1|ACVR1C -1.021446766 0 11 0 0 -11.41707142 4.369885465
1 -2.37761358 GO:0050796 M1 1 0 GO Biological Processes 19 regulation of insulin secretion -2.37761358 3.148527028 3.460375738 28162 178 402 8 1.990049751 0.696552772 240|952|2182|2697|3553|4843|6566|130399 ALOX5|CD38|ACSL4|GJA1|IL1B|NOS2|SLC16A1|ACVR1C -0.996680676 0 11 0 0 -11.41707142 4.369885465
1 -2.23178057 GO:1903532 M1 1 0 GO Biological Processes 19 positive regulation of secretion by cell -2.23178057 2.585045253 3.155161548 28162 271 402 10 2.487562189 0.776790586 952|1906|1908|2182|2697|3553|5028|6696|28984|57126 CD38|EDN1|EDN3|ACSL4|GJA1|IL1B|P2RY1|SPP1|RGCC|CD177 -0.882189587 0 11 0 0 -11.41707142 4.369885465
1 -10.76599264 GO:0044706 M1 1 0 GO Biological Processes 19 multi-multicellular organism process -10.76599264 6.868110428 10.12249669 28162 204 402 20 4.975124378 1.084445172 682|952|1906|2182|2697|3248|3488|3490|3553|3620|4313|4316|4318|5270|5368|6696|7056|7076|81671|130399 BSG|CD38|EDN1|ACSL4|GJA1|HPGD|IGFBP5|IGFBP7|IL1B|IDO1|MMP2|MMP7|MMP9|SERPINE2|PNOC|SPP1|THBD|TIMP1|VMP1|ACVR1C -8.188832017 0 12 1 1 -10.76599264 14.01094527
1 -10.01252469 GO:0007565 M1 1 0 GO Biological Processes 19 female pregnancy -10.01252469 7.124209461 9.835611036 28162 177 402 18 4.47761194 1.031484687 682|952|2182|2697|3248|3488|3490|3553|3620|4313|4316|4318|5368|6696|7056|7076|81671|130399 BSG|CD38|ACSL4|GJA1|HPGD|IGFBP5|IGFBP7|IL1B|IDO1|MMP2|MMP7|MMP9|PNOC|SPP1|THBD|TIMP1|VMP1|ACVR1C-7.497020968 0 12 0 0 -10.76599264 14.01094527
1 -8.711987104 GO:0007566 M1 1 0 GO Biological Processes 19 embryo implantation -8.711987104 14.01094527 11.08090706 28162 50 402 10 2.487562189 0.776790586 682|2697|3490|3553|4313|4318|6696|7076|81671|130399 BSG|GJA1|IGFBP7|IL1B|MMP2|MMP9|SPP1|TIMP1|VMP1|ACVR1C -6.323015857 0 12 0 0 -10.76599264 14.01094527
1 -2.561047129 GO:0046697 M1 1 0 GO Biological Processes 19 decidualization -2.561047129 10.50820896 5.118745859 28162 20 402 3 0.746268657 0.429247719 682|2697|6696 BSG|GJA1|SPP1 -1.137313776 0 12 0 0 -10.76599264 14.01094527
1 -2.139188431 GO:0001893 M1 1 0 GO Biological Processes 19 maternal placenta development -2.139188431 7.505863539 4.144723426 28162 28 402 3 0.746268657 0.429247719 682|2697|6696 BSG|GJA1|SPP1 -0.810292083 0 12 0 0 -10.76599264 14.01094527
1 -2.07688532 GO:0060135 M1 1 0 GO Biological Processes 19 maternal process involved in female pregnancy -2.07688532 5.003909026 3.609159088 28162 56 402 4 0.995024876 0.495031064 682|2697|4316|6696 BSG|GJA1|MMP7|SPP1 -0.762296437 0 12 0 0 -10.76599264 14.01094527

1 -10.51340097 GO:0045178 M1 1 0 GO Cellular Components20 basal part of cell -10.51340097 5.927707616 9.605970972 28162 260 402 22 5.472636816 1.134394361 288|301|360|366|682|762|952|1906|3248|4478|5028|5967|6533|6566|8140|8671|9073|9076|9963|23705|123264|200931 ANK3|ANXA1|AQP3|AQP9|BSG|CA4|CD38|EDN1|HPGD|MSN|P2RY1|REG1A|SLC6A6|SLC16A1|SLC7A5|SLC4A4|CLDN8|CLDN1|SLC23A1|CADM1|SLC51B|SLC51A -7.957780023 0 13 1 1 -10.51340097 6.24901315

1 -9.549588736 GO:0016323 M1 1 0 GO Cellular Components20 basolateral plasma membrane -9.549588736 6.24901315 9.253619923 28162 213 402 19 4.726368159 1.058368993 288|301|360|366|682|762|952|3248|4478|5028|6533|6566|8140|8671|9073|9076|23705|123264|200931 ANK3|ANXA1|AQP3|AQP9|BSG|CA4|CD38|HPGD|MSN|P2RY1|SLC6A6|SLC16A1|SLC7A5|SLC4A4|CLDN8|CLDN1|CADM1|SLC51B|SLC51A -7.076637361 0 13 0 0 -10.51340097 6.24901315

1 -9.429519716 GO:0009925 M1 1 0 GO Cellular Components20 basal plasma membrane -9.429519716 5.789646807 9.004939458 28162 242 402 20 4.975124378 1.084445172 288|301|360|366|682|762|952|3248|4478|5028|6533|6566|8140|8671|9073|9076|9963|23705|123264|200931 ANK3|ANXA1|AQP3|AQP9|BSG|CA4|CD38|HPGD|MSN|P2RY1|SLC6A6|SLC16A1|SLC7A5|SLC4A4|CLDN8|CLDN1|SLC23A1|CADM1|SLC51B|SLC51A -6.97360168 0 13 0 0 -10.51340097 6.24901315

1 -10.1536751 GO:0005509 M1 1 0 GO Molecular Functions 21 calcium ion binding -10.1536751 3.537125034 8.257540337 28162 713 402 36 8.955223881 1.424140094 301|308|716|1001|1009|4017|4038|4256|4321|4811|4925|5320|6279|6280|6282|6283|6286|6402|6678|7056|7057|7436|11167|11240|22918|50506|53841|57758|63928|64123|79174|79888|83998|84647|85477|388743 ANXA1|ANXA5|C1S|CDH3|CDH11|LOXL2|LRP4|MGP|MMP12|NID1|NUCB2|PLA2G2A|S100A8|S100A9|S100A11|S100A12|S100P|SELL|SPARC|THBD|THBS1|VLDLR|FSTL1|PADI2|CD93|DUOX2|CDHR5|SCUBE2|CHP2|ADGRL4|CRELD2|LPCAT1|REG4|PLA2G12B|SCIN|CAPN8 -7.611842438 0 14 1 1 -10.1536751 3.537125034

1 -10.10672347 GO:0032787 M1 1 0 GO Biological Processes 19 monocarboxylic acid metabolic process -10.10672347 3.692807281 8.32553058 28162 645 402 34 8.457711443 1.387791708 18|51|126|240|301|682|718|857|957|1555|1906|2053|2182|3248|3553|3620|4199|5105|5166|5209|5468|6566|8671|8876|8942|9388|10891|27163|29785|55198|60481|79799|80221|114876 ABAT|ACOX1|ADH1C|ALOX5|ANXA1|BSG|C3|CAV1|ENTPD5|CYP2B6|EDN1|EPHX2|ACSL4|HPGD|IL1B|IDO1|ME1|PCK1|PDK4|PFKFB3|PPARG|SLC16A1|SLC4A4|VNN1|KYNU|LIPG|PPARGC1A|NAAA|CYP2S1|APPL2|ELOVL5|UGT2A3|ACSF2|OSBPL1A -7.57162419 0 15 1 1 -10.10672347 15.01172708

1 -6.564713155 GO:0006631 M1 1 0 GO Biological Processes 19 fatty acid metabolic process -6.564713155 3.75293177 6.605076824 28162 392 402 21 5.223880597 1.109770192 51|240|301|718|857|1555|1906|2053|2182|3248|3553|5105|5166|5468|9388|10891|27163|29785|55198|60481|80221 ACOX1|ALOX5|ANXA1|C3|CAV1|CYP2B6|EDN1|EPHX2|ACSL4|HPGD|IL1B|PCK1|PDK4|PPARG|LIPG|PPARGC1A|NAAA|CYP2S1|APPL2|ELOVL5|ACSF2 -4.439416988 0 15 0 0 -10.10672347 15.01172708

1 -6.385462018 GO:0019216 M1 1 0 GO Biological Processes 19 regulation of lipid metabolic process -6.385462018 3.659575258 6.463130301 28162 402 402 21 5.223880597 1.109770192 12|51|183|301|718|857|2053|2330|3158|3490|3553|4067|4199|5105|5166|5468|10451|10891|55198|60481|79888 SERPINA3|ACOX1|AGT|ANXA1|C3|CAV1|EPHX2|FMO5|HMGCS2|IGFBP7|IL1B|LYN|ME1|PCK1|PDK4|PPARG|VAV3|PPARGC1A|APPL2|ELOVL5|LPCAT1 -4.297520768 0 15 0 0 -10.10672347 15.01172708

1 -5.212811599 GO:0062013 M1 1 0 GO Biological Processes 19 positive regulation of small molecule metabolic process -5.212811599 5.465262341 6.396494822 28162 141 402 11 2.736318408 0.813665012 301|957|2981|3553|4843|5028|5209|5468|8671|10891|60481 ANXA1|ENTPD5|GUCA2B|IL1B|NOS2|P2RY1|PFKFB3|PPARG|SLC4A4|PPARGC1A|ELOVL5 -3.284754709 0 15 0 0 -10.10672347 15.01172708

1 -5.208729545 GO:0033559 M1 1 0 GO Biological Processes 19 unsaturated fatty acid metabolic process -5.208729545 6.091715336 6.584111249 28162 115 402 10 2.487562189 0.776790586 51|240|301|1555|1906|2053|3248|3553|29785|60481 ACOX1|ALOX5|ANXA1|CYP2B6|EDN1|EPHX2|HPGD|IL1B|CYP2S1|ELOVL5 -3.282320833 0 15 0 0 -10.10672347 15.01172708

1 -4.675734339 GO:0008610 M1 1 0 GO Biological Processes 19 lipid biosynthetic process -4.675734339 2.498522477 4.933074858 28162 729 402 26 6.467661692 1.226708902 240|301|718|1906|2053|2182|3158|3248|3284|3294|3490|3553|5105|5166|5320|9388|10891|55151|56895|60481|79154|79644|79888|80157|80235|114876 ALOX5|ANXA1|C3|EDN1|EPHX2|ACSL4|HMGCS2|HPGD|HSD3B2|HSD17B2|IGFBP7|IL1B|PCK1|PDK4|PLA2G2A|LIPG|PPARGC1A|TMEM38B|AGPAT4|ELOVL5|DHRS11|SRD5A3|LPCAT1|CWH43|PIGZ|OSBPL1A -2.832871679 0 15 0 0 -10.10672347 15.01172708

1 -4.497674147 GO:0032309 M1 1 0 GO Biological Processes 19 icosanoid secretion -4.497674147 9.775078098 6.92967097 28162 43 402 6 1.492537313 0.604761504 301|1906|3553|4843|5320|84647 ANXA1|EDN1|IL1B|NOS2|PLA2G2A|PLA2G12B -2.693729553 0 15 0 0 -10.10672347 15.01172708

1 -4.219947106 GO:0006692 M1 1 0 GO Biological Processes 19 prostanoid metabolic process -4.219947106 8.756840796 6.472491791 28162 48 402 6 1.492537313 0.604761504 51|301|1906|3248|3553|29785 ACOX1|ANXA1|EDN1|HPGD|IL1B|CYP2S1 -2.467430689 0 15 0 0

-10.10672347 15.01172708
1 -4.219947106 GO:0006693 M1 1 0 GO Biological Processes 19 prostaglandin metabolic process -4.219947106 8.756840796 6.472491791 28162 48 402 6 1.492537313 0.604761504 51|301|1906|3248|3553|29785 ACOX1|ANXA1|EDN1|HPGD|IL1B|CYP2S1 -2.467430689 0 15 0 0 -10.10672347 15.01172708
1 -4.150466084 GO:0006690 M1 1 0 GO Biological Processes 19 icosanoid metabolic process -4.150466084 5.125955588 5.518527057 28162 123 402 9 2.23880597 0.737867616 51|240|301|1555|1906|2053|3248|3553|29785 ACOX1|ALOX5|ANXA1|CYP2B6|EDN1|EPHX2|HPGD|IL1B|CYP2S1 -2.409908469 0 15 0 0 -10.10672347 15.01172708
1 -4.086359644 GO:0042180 M1 1 0 GO Biological Processes 19 cellular ketone metabolic process -4.086359644 3.585478121 4.981047694 28162 254 402 13 3.233830846 0.882281956 301|857|1555|3553|3620|5166|5468|6999|8942|10891|23657|55198|60481 ANXA1|CAV1|CYP2B6|IL1B|IDO1|PDK4|PPARG|TDO2|KYNU|PPARGC1A|SLC7A11|APPL2|ELOVL5 -2.355347347 0 15 0 0 -10.10672347 15.01172708
1 -4.082880801 GO:0019217 M1 1 0 GO Biological Processes 19 regulation of fatty acid metabolic process -4.082880801 5.718753173 5.63109488 28162 98 402 8 1.990049751 0.696552772 301|857|3553|5166|5468|10891|55198|60481 ANXA1|CAV1|IL1B|PDK4|PPARG|PPARGC1A|APPL2|ELOVL5 -2.353961493 0 15 0 0 -10.10672347 15.01172708
1 -4.068984893 GO:0071715 M1 1 0 GO Biological Processes 19 icosanoid transport -4.068984893 8.241732514 6.228977351 28162 51 402 6 1.492537313 0.604761504 301|1906|3553|4843|5320|84647 ANXA1|EDN1|IL1B|NOS2|PLA2G2A|PLA2G12B -2.341108309 0 15 0 0 -10.10672347 15.01172708
1 -3.705744461 GO:0045923 M1 1 0 GO Biological Processes 19 positive regulation of fatty acid metabolic process -3.705744461 9.217727154 6.100034669 28162 38 402 5 1.243781095 0.552765814 301|3553|5468|10891|60481 ANXA1|IL1B|PPARG|PPARGC1A|ELOVL5 -2.042577184 0 15 0 0 -10.10672347 15.01172708
1 -3.671174343 GO:0062012 M1 1 0 GO Biological Processes 19 regulation of small molecule metabolic process -3.671174343 2.71901906 4.363320697 28162 438 402 17 4.228855721 1.003727321 301|857|957|2053|2330|2981|3553|4843|5028|5166|5209|5468|8671|10891|23657|55198|60481 ANXA1|CAV1|ENTPD5|EPHX2|FMO5|GUCA2B|IL1B|NOS2|P2RY1|PDK4|PFKFB3|PPARG|SLC4A4|PPARGC1A|SLC7A11|APPL2|ELOVL5 -2.014243196 0 15 0 0 -10.10672347 15.01172708
1 -3.39208188 GO:0072330 M1 1 0 GO Biological Processes 19 monocarboxylic acid biosynthetic process -3.39208188 3.440187456 4.412420308 28162 224 402 11 2.736318408 0.813665012 240|301|1906|2053|3248|3553|3620|5166|9388|60481|114876

ALOX5|ANXA1|EDN1|EPHX2|HPGD|IL1B|IDO1|PDK4|LIPG|ELOVL5|OSBPL1A
-1.794577636 0 15 0 0 -10.10672347 15.01172708
1 -3.139515502 GO:0006633 M1 1 0 GO Biological Processes 19 fatty acid biosynthetic process -3.139515502 3.75293177 4.306703184 28162 168 402 9
2.23880597 0.737867616 240|301|1906|2053|3248|3553|5166|9388|60481
ALOX5|ANXA1|EDN1|EPHX2|HPGD|IL1B|PDK4|LIPG|ELOVL5 -1.584594464 0 15 0
0 -10.10672347 15.01172708
1 -3.029274715 GO:0032310 M1 1 0 GO Biological Processes 19 prostaglandin secretion -3.029274715 15.01172708 6.31043428 28162 14 402 3 0.746268657
0.429247719 1906|3553|4843 EDN1|IL1B|NOS2 -1.500806012 0 15 0 0
-10.10672347 15.01172708
1 -3.020847866 GO:0046394 M1 1 0 GO Biological Processes 19 carboxylic acid biosynthetic process -3.020847866 2.776559277 3.894539788 28162 328 402 13
3.233830846 0.882281956
240|301|1906|2053|3248|3553|3620|5166|8942|9388|29968|60481|114876
ALOX5|ANXA1|EDN1|EPHX2|HPGD|IL1B|IDO1|PDK4|KYNU|LIPG|PSAT1|ELOVL5|OSBPL1A
-1.49761169 0 15 0 0 -10.10672347 15.01172708
1 -2.960565937 GO:0044283 M1 1 0 GO Biological Processes 19 small molecule biosynthetic process -2.960565937 2.122870496 3.601655709 28162 693 402 21
5.223880597 1.109770192
240|301|957|1906|2053|3158|3248|3553|3620|5028|5105|5166|8942|9388|10891|10924|29968|51363|60481|79644|114876
ALOX5|ANXA1|ENTPD5|EDN1|EPHX2|HMGCS2|HPGD|IL1B|IDO1|P2RY1|PCK1|PDK4|KYNU|LIPG|PPARGC1A|SMPDL3A|PSAT1|CHST15|ELOVL5|SRD5A3|OSBPL1A -1.446337673 0 15
0 0 -10.10672347 15.01172708
1 -2.926347301 GO:0016053 M1 1 0 GO Biological Processes 19 organic acid biosynthetic process -2.926347301 2.710450723 3.795614937 28162 336 402 13
3.233830846 0.882281956
240|301|1906|2053|3248|3553|3620|5166|8942|9388|29968|60481|114876
ALOX5|ANXA1|EDN1|EPHX2|HPGD|IL1B|IDO1|PDK4|KYNU|LIPG|PSAT1|ELOVL5|OSBPL1A
-1.421085478 0 15 0 0 -10.10672347 15.01172708
1 -2.845359753 GO:0010565 M1 1 0 GO Biological Processes 19 regulation of cellular ketone metabolic process -2.845359753 3.408067769 3.954406667 28162 185
402 9 2.23880597 0.737867616
301|857|3553|5166|5468|10891|23657|55198|60481
ANXA1|CAV1|IL1B|PDK4|PPARG|PPARGC1A|SLC7A11|APPL2|ELOVL5 -1.359478494 0
15 0 0 -10.10672347 15.01172708
1 -2.836885433 GO:0045834 M1 1 0 GO Biological Processes 19 positive regulation of lipid metabolic process -2.836885433 3.736252073 4.043495892 28162 150 402
8 1.990049751 0.696552772 183|301|3553|4067|5468|10451|10891|60481
AGT|ANXA1|IL1B|LYN|PPARG|VAV3|PPARGC1A|ELOVL5 -1.351600323 0 15 0 0
-10.10672347 15.01172708
1 -2.771670055 GO:0015732 M1 1 0 GO Biological Processes 19 prostaglandin transport -2.771670055 12.36259877 5.639347249 28162 17 402 3 0.746268657

0.429247719 1906|3553|4843 EDN1|IL1B|NOS2 -1.303326622 0 15 0 0 -10.10672347 15.01172708

1 -2.571277049 GO:0046890 M1 1 0 GO Biological Processes 19 regulation of lipid biosynthetic process -2.571277049 3.105874568 3.623656593 28162 203 402 9 2.23880597 0.737867616 301|718|3490|3553|5105|5166|10891|60481|79888 ANXA1|C3|IGFBP7|IL1B|PCK1|PDK4|PPARGC1A|ELOVL5|LPCAT1 -1.141064218 0 15 0 0 -10.10672347 15.01172708

1 -2.383476348 GO:0045723 M1 1 0 GO Biological Processes 19 positive regulation of fatty acid biosynthetic process -2.383476348 9.137573005 4.698203434 28162 23 402 3 0.746268657 0.429247719 301|3553|60481 ANXA1|IL1B|ELOVL5 -1.001605443 0 15 0 0 -10.10672347 15.01172708

1 -2.220014968 GO:0006636 M1 1 0 GO Biological Processes 19 unsaturated fatty acid biosynthetic process -2.220014968 5.494488343 3.865934849 28162 51 402 4 0.995024876 0.495031064 301|1906|3553|60481 ANXA1|EDN1|IL1B|ELOVL5 -0.8750318 0 15 0 0 -10.10672347 15.01172708

1 -2.132224823 GO:0042304 M1 1 0 GO Biological Processes 19 regulation of fatty acid biosynthetic process -2.132224823 5.18923899 3.70803971 28162 54 402 4 0.995024876 0.495031064 301|3553|5166|60481 ANXA1|IL1B|PDK4|ELOVL5 -0.805814901 0 15 0 0 -10.10672347 15.01172708

1 -2.07688532 GO:0046456 M1 1 0 GO Biological Processes 19 icosanoid biosynthetic process -2.07688532 5.003909026 3.609159088 28162 56 402 4 0.995024876 0.495031064 240|301|1906|3553 ALOX5|ANXA1|EDN1|IL1B -0.762296437 0 15 0 0 -10.10672347 15.01172708

1 -2.015464226 GO:0001516 M1 1 0 GO Biological Processes 19 prostaglandin biosynthetic process -2.015464226 6.779489649 3.8744056 28162 31 402 3 0.746268657 0.429247719 301|1906|3553 ANXA1|EDN1|IL1B -0.713946752 0 15 0 0 -10.10672347 15.01172708

1 -2.015464226 GO:0046457 M1 1 0 GO Biological Processes 19 prostanoid biosynthetic process -2.015464226 6.779489649 3.8744056 28162 31 402 3 0.746268657 0.429247719 301|1906|3553 ANXA1|EDN1|IL1B -0.713946752 0 15 0 0 -10.10672347 15.01172708

1 -10.08983319 GO:0008285 M1 1 0 GO Biological Processes 19 negative regulation of cell population proliferation -10.08983319 3.442264111 8.174681715 28162 753 402 37 9.2039801 1.441810602 240|687|857|1030|2263|2697|2919|3437|3488|3490|3553|3620|4067|4332|5270|5468|5967|6091|6282|6678|7031|7057|7453|7474|9510|10891|11040|26585|28984|29126|55198|55214|64081|85477|90865|130399|282679 ALOX5|KLF9|CAV1|CDKN2B|FGFR2|GJA1|CXCL1|IFIT3|IGFBP5|IGFBP7|IL1B|IDO1|LYN|MNDA|SERPINE2|PPARG|REG1A|ROBO1|S100A11|SPARC|TFF1|THBS1|WARS1|WNT5A|ADAMTS1|PPARGC1A|PIM2|GREM1|RGCC|CD274|APPL2|P3H2|PBLD|SCIN|IL33|ACVR1C|AQP11 -7.561364482 0 16 1 1 -10.08983319 6.452409008

1 -9.576024263 GO:0050673 M1 1 0 GO Biological Processes 19 epithelial cell proliferation -9.576024263 4.308605039 8.407839377 28162 439 402 27 6.71641791 1.248413442

240|687|857|1001|1030|2263|2697|3373|3488|3915|4017|4321|5068|5268|5468|5967|6091|6356|6678|7057|7474|9076|28984|60675|64081|195814|282679

ALOX5|KLF9|CAV1|CDH3|CDKN2B|FGFR2|GJA1|HYAL1|IGFBP5|LAMC1|LOXL2|MMP12|REG3A|SERPINB5|PPARG|REG1A|ROBO1|CCL11|SPARC|THBS1|WNT5A|CLDN1|RGCC|PROK2|PBLD|SDR16C5|AQP11-7.097243344 0 16 0 0 -10.08983319 6.452409008

1 -8.045467671 GO:0050678 M1 1 0 GO Biological Processes 19 regulation of epithelial cell proliferation -8.045467671 4.206941792 7.604304383 28162 383 402 23 5.721393035 1.158362377

240|687|857|1001|1030|2263|2697|3373|3915|4321|5068|5268|5468|5967|6091|6356|6678|7057|7474|9076|28984|64081|282679

ALOX5|KLF9|CAV1|CDH3|CDKN2B|FGFR2|GJA1|HYAL1|LAMC1|MMP12|REG3A|SERPINB5|PPARG|REG1A|ROBO1|CCL11|SPARC|THBS1|WNT5A|CLDN1|RGCC|PBLD|AQP11

-5.746673055 0 16 0 0 -10.08983319 6.452409008

1 -7.537645055 GO:0001667 M1 1 0 GO Biological Processes 19 ameboidal-type cell migration -7.537645055 3.679344872 7.094622935 28162 476 402 25 6.218905473 1.204485566

183|301|1906|1908|1942|2697|3373|4017|4318|5308|5468|5801|6091|6286|6678|7057|7076|7474|11167|26585|28984|55198|56999|64081|130399

AGT|ANXA1|EDN1|EDN3|EFNA1|GJA1|HYAL1|LOXL2|MMP9|PITX2|PPARG|PTPRR|ROBO1|S100P|SPARC|THBS1|TIMP1|WNT5A|FSTL1|GREM1|RGCC|APPL2|ADAMTS9|PBLD|ACVR1C

-5.316737215 0 16 0 0 -10.08983319 6.452409008

1 -7.056497024 GO:0050680 M1 1 0 GO Biological Processes 19 negative regulation of epithelial cell proliferation -7.056497024 6.091715336 7.796821913 28162 161 402 14 3.482587065 0.914409651

240|687|857|1030|2263|2697|5468|6091|6678|7057|7474|28984|64081|282679

ALOX5|KLF9|CAV1|CDKN2B|FGFR2|GJA1|PPARG|ROBO1|SPARC|THBS1|WNT5A|RGCC|PBLD|AQP11 -4.878746101 0 16 0 0 -10.08983319 6.452409008

1 -5.91716603 GO:0010631 M1 1 0 GO Biological Processes 19 epithelial cell migration -5.91716603 3.707631758 6.213242806 28162 359 402 19 4.726368159 1.058368993

183|301|1906|1942|3373|4017|4318|5468|5801|6091|6286|6678|7057|7474|11167|26585|28984|56999|64081

AGT|ANXA1|EDN1|EFNA1|HYAL1|LOXL2|MMP9|PPARG|PTPRR|ROBO1|S100P|SPARC|THBS1|WNT5A|FSTL1|GREM1|RGCC|ADAMTS9|PBLD -3.883730575 0 16 0 0

-10.08983319 6.452409008

1 -5.863632329 GO:0090132 M1 1 0 GO Biological Processes 19 epithelium migration -5.863632329 3.676905528 6.168680311 28162 362 402 19 4.726368159 1.058368993

183|301|1906|1942|3373|4017|4318|5468|5801|6091|6286|6678|7057|7474|11167|26585|28984|56999|64081

AGT|ANXA1|EDN1|EFNA1|HYAL1|LOXL2|MMP9|PPARG|PTPRR|ROBO1|S100P|SPARC|THBS1|WNT5A|FSTL1|GREM1|RGCC|ADAMTS9|PBLD -3.837838985 0 16 0 0

-10.08983319 6.452409008

1 -5.775727852 GO:0090130 M1 1 0 GO Biological Processes 19 tissue migration

-5.775727852 3.626811447 6.095452131 28162 367 402 19 4.726368159
1.058368993
183|301|1906|1942|3373|4017|4318|5468|5801|6091|6286|6678|7057|7474|11167|26585|28984|56999|64081
AGT|ANXA1|EDN1|EFNA1|HYAL1|LOXL2|MMP9|PPARG|PTPRR|ROBO1|S100P|SPARC|THBS1|WNT5A|FSTL1|GREM1|RGCC|ADAMTS9|PBLD -3.756094817 0 16 0 0
-10.08983319 6.452409008
1 -4.857704277 GO:0043542 M1 1 0 GO Biological Processes 19 endothelial cell migration -4.857704277 3.75293177 5.571085631 28162 280 402 15 3.731343284
0.945283402
183|301|1906|1942|4017|5468|6091|6286|6678|7057|7474|11167|26585|28984|56999
AGT|ANXA1|EDN1|EFNA1|LOXL2|PPARG|ROBO1|S100P|SPARC|THBS1|WNT5A|FSTL1|GREM1|RGCC|ADAMTS9 -2.989698582 0 16 0 0 -10.08983319 6.452409008
1 -4.607457951 GO:0030336 M1 1 0 GO Biological Processes 19 negative regulation of cell migration -4.607457951 3.396592794 5.26994816 28162 330 402
16 3.980099502 0.975022334
1281|2697|3488|5468|5801|6091|7057|7076|10891|11240|26585|28984|56999|64081|90865|130399
COL3A1|GJA1|IGFBP5|PPARG|PTPRR|ROBO1|THBS1|TIMP1|PPARGC1A|PADI2|GREM1|RGCC|ADAMTS9|PBLD|IL33|ACVR1C -2.771081098 0 16 0 0 -10.08983319
6.452409008
1 -4.459195557 GO:0040013 M1 1 0 GO Biological Processes 19 negative regulation of locomotion -4.459195557 3.158966441 5.078537904 28162 377 402
17 4.228855721 1.003727321
1281|2697|3488|5468|5801|6091|7057|7076|7474|10891|11240|26585|28984|56999|64081|90865|130399
COL3A1|GJA1|IGFBP5|PPARG|PTPRR|ROBO1|THBS1|TIMP1|WNT5A|PPARGC1A|PADI2|GREM1|RGCC|ADAMTS9|PBLD|IL33|ACVR1C -2.661411271 0 16 0 0
-10.08983319 6.452409008
1 -4.375446614 GO:2000146 M1 1 0 GO Biological Processes 19 negative regulation of cell motility -4.375446614 3.248914846 5.057719568 28162 345 402
16 3.980099502 0.975022334
1281|2697|3488|5468|5801|6091|7057|7076|10891|11240|26585|28984|56999|64081|90865|130399
COL3A1|GJA1|IGFBP5|PPARG|PTPRR|ROBO1|THBS1|TIMP1|PPARGC1A|PADI2|GREM1|RGCC|ADAMTS9|PBLD|IL33|ACVR1C -2.590915061 0 16 0 0 -10.08983319
6.452409008
1 -4.271934482 GO:0051271 M1 1 0 GO Biological Processes 19 negative regulation of cellular component movement -4.271934482 3.184305744 4.962626577
28162 352 402 16 3.980099502 0.975022334
1281|2697|3488|5468|5801|6091|7057|7076|10891|11240|26585|28984|56999|64081|90865|130399
COL3A1|GJA1|IGFBP5|PPARG|PTPRR|ROBO1|THBS1|TIMP1|PPARGC1A|PADI2|GREM1|RG

CC|ADAMTS9|PBLD|IL33|ACVR1C -2.510509148 0 16 0 0 -10.08983319 6.452409008
1 -4.021156897 GO:0010632 M1 1 0 GO Biological Processes 19 regulation of epithelial cell migration -4.021156897 3.335939351 4.845177134 28162 294 402 14 3.482587065 0.914409651 183|301|1906|1942|3373|4318|5468|5801|6678|7057|7474|28984|56999|64081 AGT|ANXA1|EDN1|EFNA1|HYAL1|MMP9|PPARG|PTPRR|SPARC|THBS1|WNT5A|RGCC|ADAMTS9|PBLD -2.305460374 0 16 0 0 -10.08983319 6.452409008
1 -3.968766049 GO:0001937 M1 1 0 GO Biological Processes 19 negative regulation of endothelial cell proliferation -3.968766049 6.452409008 5.727664999 28162 76 402 7 1.741293532 0.652391792 240|857|2697|5468|6678|7057|28984 ALOX5|CAV1|GJA1|PPARG|SPARC|THBS1|RGCC -2.269113421 0 16 0 0 -10.08983319 6.452409008
1 -3.853515461 GO:0001935 M1 1 0 GO Biological Processes 19 endothelial cell proliferation -3.853515461 3.891929243 4.914144731 28162 198 402 11 2.736318408 0.813665012 240|857|2697|4017|5468|6356|6678|7057|7474|28984|60675 ALOX5|CAV1|GJA1|LOXL2|PPARG|CCL11|SPARC|THBS1|WNT5A|RGCC|PROK2 -2.167315508 0 16 0 0 -10.08983319 6.452409008
1 -2.878052978 GO:0001936 M1 1 0 GO Biological Processes 19 regulation of endothelial cell proliferation -2.878052978 3.445314412 3.993663693 28162 183 402 9 2.23880597 0.737867616 240|857|2697|5468|6356|6678|7057|7474|28984 ALOX5|CAV1|GJA1|PPARG|CCL11|SPARC|THBS1|WNT5A|RGCC -1.38190994 0 16 0 0 -10.08983319 6.452409008
1 -2.708718666 GO:0010594 M1 1 0 GO Biological Processes 19 regulation of endothelial cell migration -2.708718666 3.019600274 3.717074603 28162 232 402 10 2.487562189 0.776790586 183|301|1906|1942|5468|6678|7057|7474|28984|56999 AGT|ANXA1|EDN1|EFNA1|PPARG|SPARC|THBS1|WNT5A|RGCC|ADAMTS9 -1.252243485 0 16 0 0 -10.08983319 6.452409008
1 -2.407090069 GO:0010634 M1 1 0 GO Biological Processes 19 positive regulation of epithelial cell migration -2.407090069 3.184305744 3.498055364 28162 176 402 8 1.990049751 0.696552772 183|301|1906|3373|4318|6678|7057|7474 AGT|ANXA1|EDN1|HYAL1|MMP9|SPARC|THBS1|WNT5A -1.021446766 0 16 0 0 -10.08983319 6.452409008
1 -2.223997773 GO:0010633 M1 1 0 GO Biological Processes 19 negative regulation of epithelial cell migration -2.223997773 3.687090861 3.459482676 28162 114 402 6 1.492537313 0.604761504 5468|5801|7057|28984|56999|64081 PPARG|PTPRR|THBS1|RGCC|ADAMTS9|PBLD -0.876598754 0 16 0 0 -10.08983319 6.452409008
1 -9.658258659 GO:0002697 M1 1 0 GO Biological Processes 19 regulation of immune effector process -9.658258659 4.20028338 8.392155522 28162 467 402 28 6.965174129 1.269625794

301|629|716|718|722|725|1604|3426|3553|3575|3598|4067|4321|4843|5105|5272|6372|7098|7474|8140|11005|11126|23705|51237|54941|55198|57126|90865

ANXA1|CFB|C1S|C3|C4BPA|C4BPB|CD55|CFI|IL1B|IL7R|IL13RA2|LYN|MMP12|NOS2|PCK1|SERPINB9|CXCL6|TLR3|WNT5A|SLC7A5|SPINK5|CD160|CADM1|MZB1|RNF125|APPL2|CD177|IL33 -7.167578517 0 17 1 1 -9.658258659 6.538441128

1 -8.299809005 GO:0002683 M1 1 0 GO Biological Processes 19 negative regulation of immune system process -8.299809005 4.17199363 7.718097367 28162 403 402 24 5.970149254 1.181714164

301|722|725|1281|1604|2633|3575|3598|3620|4067|4321|4332|5272|5468|7057|7098|11005|11126|11240|26585|29126|54941|55824|90865

ANXA1|C4BPA|C4BPB|COL3A1|CD55|GBP1|IL7R|IL13RA2|IDO1|LYN|MMP12|MNDA|SERPINB9|PPARG|THBS1|TLR3|SPINK5|CD160|PADI2|GREM1|CD274|RNF125|PAG1|IL33

-5.964633591 0 17 0 0 -9.658258659 6.538441128

1 -7.4443201 GO:0050777 M1 1 0 GO Biological Processes 19 negative regulation of immune response -7.4443201 6.538441128 8.184430144 28162 150 402 14 3.482587065 0.914409651

301|722|725|1281|1604|3575|3598|4067|4321|5272|5468|11005|11126|90865

ANXA1|C4BPA|C4BPB|COL3A1|CD55|IL7R|IL13RA2|LYN|MMP12|SERPINB9|PPARG|SPINK5|CD160|IL33 -5.233028028 0 17 0 0 -9.658258659 6.538441128

1 -6.187015763 GO:0050778 M1 1 0 GO Biological Processes 19 positive regulation of immune response -6.187015763 2.761684351 5.928769287 28162 761 402 30 7.462686567 1.31066897

301|629|716|718|722|725|952|1604|2212|2357|2633|3426|3553|3620|4067|4321|4332|5105|7474|10451|11126|11148|23705|28984|29126|55198|55824|57126|90865|727897

ANXA1|CFB|C1S|C3|C4BPA|C4BPB|CD38|CD55|FCGR2A|FPR1|GBP1|CFI|IL1B|IDO1|LYN|MMP12|MNDA|PCK1|WNT5A|VAV3|CD160|HHLA2|CADM1|RGCC|CD274|APPL2|PAG1|CD177|IL33|MUC5B -4.122304353 0 17 0 0 -9.658258659 6.538441128

1 -6.139173964 GO:0002699 M1 1 0 GO Biological Processes 19 positive regulation of immune effector process -6.139173964 4.776458616 6.766976341 28162 220 402 15 3.731343284 0.945283402

301|718|3553|3598|4067|4843|5105|7474|8140|11126|23705|51237|55198|57126|90865

ANXA1|C3|IL1B|IL13RA2|LYN|NOS2|PCK1|WNT5A|SLC7A5|CD160|CADM1|MZB1|APPL2|CD177|IL33 -4.081195937 0 17 0 0 -9.658258659 6.538441128

1 -5.679051425 GO:0002703 M1 1 0 GO Biological Processes 19 regulation of leukocyte mediated immunity -5.679051425 4.692661096 6.448010169 28162 209 402 14 3.482587065 0.914409651

718|722|725|3553|3575|3598|4067|4843|5272|6372|7098|11126|23705|57126

C3|C4BPA|C4BPB|IL1B|IL7R|IL13RA2|LYN|NOS2|SERPINB9|CXCL6|TLR3|CD160|CADM1|CD177 -3.673461468 0 17 0 0 -9.658258659 6.538441128

1 -4.887047087 GO:0002698 M1 1 0 GO Biological Processes 19 negative regulation of immune effector process -4.887047087 5.604378109 6.208512332 28162 125 402 10 2.487562189 0.776790586

301|722|725|1604|3575|3598|5272|11005|54941|90865

ANXA1|C4BPA|C4BPB|CD55|IL7R|IL13RA2|SERPINB9|SPINK5|RNF125|IL33
-3.013250712 0 17 0 0 -9.658258659 6.538441128
1 -4.486074512 GO:0002920 M1 1 0 GO Biological Processes 19 regulation of humoral immune response -4.486074512 5.039908372 5.745779213 28162 139 402 10 2.487562189 0.776790586 629|716|718|722|725|1604|3426|3553|11005|124976
CFB|C1S|C3|C4BPA|C4BPB|CD55|CFI|IL1B|SPINK5|SPNS2 -2.684604537 0 17 0 0 -9.658258659 6.538441128
1 -4.047939833 GO:0002253 M1 1 0 GO Biological Processes 19 activation of immune response -4.047939833 2.567450705 4.561740274 28162 573 402 21 5.223880597 1.109770192
629|716|718|722|725|952|1604|2212|2357|2633|3426|3553|4067|4332|10451|11126|11148|28984|55198|55824|727897
CFB|C1S|C3|C4BPA|C4BPB|CD38|CD55|FCGR2A|FPR1|GBP1|CFI|IL1B|LYN|MNDA|VAV3|CD160|HHLA2|RGCC|APPL2|PAG1|MUC5B -2.322141218 0 17 0 0 -9.658258659 6.538441128
1 -3.57327558 GO:0030449 M1 1 0 GO Biological Processes 19 regulation of complement activation -3.57327558 4.831360439 4.975925533 28162 116 402 8 1.990049751 0.696552772 629|716|718|722|725|1604|3426|3553
CFB|C1S|C3|C4BPA|C4BPB|CD55|CFI|IL1B -1.942933445 0 17 0 0 -9.658258659 6.538441128
1 -3.177614184 GO:0002250 M1 1 0 GO Biological Processes 19 adaptive immune response -3.177614184 2.16157641 3.780564193 28162 713 402 22 5.472636816 1.134394361
301|608|716|718|722|725|1604|3108|3426|3553|3575|3598|4067|11126|23705|27074|29126|54941|55824|57823|90865|255231
ANXA1|TNFRSF17|C1S|C3|C4BPA|C4BPB|CD55|HLA-DMA|CFI|IL1B|IL7R|IL13RA2|LYN|CD160|CADM1|LAMP3|CD274|RNF125|PAG1|SLAMF7|IL33|MCOLN2 -1.617769917 0 17 0 0 -9.658258659 6.538441128
1 -3.102896117 GO:0002819 M1 1 0 GO Biological Processes 19 regulation of adaptive immune response -3.102896117 3.708779631 4.262932961 28162 170 402 9 2.23880597 0.737867616 301|718|722|725|3553|3575|11126|29126|90865
ANXA1|C3|C4BPA|C4BPB|IL1B|IL7R|CD160|CD274|IL33 -1.554224028 0 17 0 0 -9.658258659 6.538441128
1 -2.94490535 GO:0006956 M1 1 0 GO Biological Processes 19 complement activation -2.94490535 3.522304678 4.073842705 28162 179 402 9 2.23880597 0.737867616 629|716|718|722|725|1604|3426|3553|28984
CFB|C1S|C3|C4BPA|C4BPB|CD55|CFI|IL1B|RGCC -1.432583283 0 17 0 0 -9.658258659 6.538441128
1 -2.940147641 GO:0002831 M1 1 0 GO Biological Processes 19 regulation of response to biotic stimulus -2.940147641 2.501954513 3.731984864 28162 420 402 15 3.731343284 0.945283402
1604|4067|4321|4332|5272|5468|6372|7474|11005|11126|23705|29126|54941|55198|727897
CD55|LYN|MMP12|MNDA|SERPINB9|PPARG|CXCL6|WNT5A|SPINK5|CD160|CADM1|CD2

74|RNF125|APPL2|MUC5B -1.429689558 0 17 0 0 -9.658258659 6.538441128
1 -2.746837451 GO:0002822 M1 1 0 GO Biological Processes 19 regulation of adaptive immune response based on somatic recombination of immune receptors built from immunoglobulin superfamily domains -2.746837451 3.615727813 3.929637011 28162 155 402 8 1.990049751 0.696552772 301|718|722|725|3553|3575|29126|90865 ANXA1|C3|C4BPA|C4BPB|IL1B|IL7R|CD274|IL33 -1.284186033 0 17 0 0 -9.658258659 6.538441128
1 -2.711886008 GO:0002706 M1 1 0 GO Biological Processes 19 regulation of lymphocyte mediated immunity -2.711886008 3.569667586 3.88540519 28162 157 402 8 1.990049751 0.696552772 718|722|725|3553|3575|5272|11126|23705 C3|C4BPA|C4BPB|IL1B|IL7R|SERPINB9|CD160|CADM1 -1.254852966 0 17 0 0 -9.658258659 6.538441128
1 -2.651538975 GO:0002449 M1 1 0 GO Biological Processes 19 lymphocyte mediated immunity -2.651538975 2.522746379 3.504114587 28162 361 402 13 3.233830846 0.882281956 716|718|722|725|1604|3426|3553|3575|3598|5272|11126|23705|57823 C1S|C3|C4BPA|C4BPB|CD55|CFI|IL1B|IL7R|IL13RA2|SERPINB9|CD160|CADM1|SLAMF7 -1.207158852 0 17 0 0 -9.658258659 6.538441128
1 -2.569651101 GO:0002460 M1 1 0 GO Biological Processes 19 adaptive immune response based on somatic recombination of immune receptors built from immunoglobulin superfamily domains -2.569651101 2.46805269 3.41597312 28162 369 402 13 3.233830846 0.882281956 301|716|718|722|725|1604|3426|3553|3575|3598|23705|29126|90865 ANXA1|C1S|C3|C4BPA|C4BPB|CD55|CFI|IL1B|IL7R|IL13RA2|CADM1|CD274|IL33 -1.141064218 0 17 0 0 -9.658258659 6.538441128
1 -2.035002585 GO:0002705 M1 1 0 GO Biological Processes 19 positive regulation of leukocyte mediated immunity -2.035002585 3.362626866 3.185750102 28162 125 402 6 1.492537313 0.604761504 718|3553|4843|11126|23705|57126 C3|IL1B|NOS2|CD160|CADM1|CD177 -0.72963256 0 17 0 0 -9.658258659 6.538441128
1 -9.512538998 GO:2000147 M1 1 0 GO Biological Processes 19 positive regulation of cell motility -9.512538998 3.78344341 8.107847391 28162 574 402 31 7.711442786 1.330542411 183|301|857|1277|1672|1906|1908|3373|3488|3553|3627|4067|4162|4316|4318|5175|5328|6282|6356|6678|7057|7130|7474|9076|9510|10979|29126|50506|57214|83483|405753 AGT|ANXA1|CAV1|COL1A1|DEFB1|EDN1|EDN3|HYAL1|IGFBP5|IL1B|CXCL10|LYN|MCAM|MMP7|MMP9|PECAM1|PLAU|S100A11|CCL11|SPARC|THBS1|TNFAIP6|WNT5A|CLDN1|ADAMTS1|FERMT2|CD274|DUOX2|CEMIP|PLVAP|DUOXA2 -7.045339952 0 18 1 1 -9.512538998 3.78344341
1 -9.294205833 GO:0051272 M1 1 0 GO Biological Processes 19 positive regulation of cellular component movement -9.294205833 3.705966753 7.96586535 28162 586 402 31 7.711442786 1.330542411 183|301|857|1277|1672|1906|1908|3373|3488|3553|3627|4067|4162|4316|4318|517

5|5328|6282|6356|6678|7057|7130|7474|9076|9510|10979|29126|50506|57214|83483|405753
AGT|ANXA1|CAV1|COL1A1|DEFB1|EDN1|EDN3|HYAL1|IGFBP5|IL1B|CXCL10|LYN|MCAM|MMP7|MMP9|PECAM1|PLAU|S100A11|CCL11|SPARC|THBS1|TNFAIP6|WNT5A|CLDN1|ADAMTS1|FERMT2|CD274|DUOX2|CEMIP|PLVAP|DUOXA2 -6.849283181 0 18 0 0 -9.512538998 3.78344341
1 -9.276284016 GO:0040017 M1 1 0 GO Biological Processes 19 positive regulation of locomotion -9.276284016 3.699653352 7.954202154 28162 587 402 31 7.711442786 1.330542411
183|301|857|1277|1672|1906|1908|3373|3488|3553|3627|4067|4162|4316|4318|5175|5328|6282|6356|6678|7057|7130|7474|9076|9510|10979|29126|50506|57214|83483|405753
AGT|ANXA1|CAV1|COL1A1|DEFB1|EDN1|EDN3|HYAL1|IGFBP5|IL1B|CXCL10|LYN|MCAM|MMP7|MMP9|PECAM1|PLAU|S100A11|CCL11|SPARC|THBS1|TNFAIP6|WNT5A|CLDN1|ADAMTS1|FERMT2|CD274|DUOX2|CEMIP|PLVAP|DUOXA2 -6.836756396 0 18 0 0 -9.512538998 3.78344341
1 -8.082897576 GO:0030335 M1 1 0 GO Biological Processes 19 positive regulation of cell migration -8.082897576 3.566422433 7.314554618 28162 550 402 28 6.965174129 1.269625794
183|301|857|1277|1906|1908|3373|3488|3553|3627|4067|4162|4316|4318|5175|5328|6282|6356|6678|7057|7130|7474|9076|9510|10979|29126|57214|83483
AGT|ANXA1|CAV1|COL1A1|EDN1|EDN3|HYAL1|IGFBP5|IL1B|CXCL10|LYN|MCAM|MMP7|MMP9|PECAM1|PLAU|S100A11|CCL11|SPARC|THBS1|TNFAIP6|WNT5A|CLDN1|ADAMTS1|FERMT2|CD274|CEMIP|PLVAP -5.780207917 0 18 0 0 -9.512538998 3.78344341
1 -9.262670087 GO:0032963 M1 1 0 GO Biological Processes 19 collagen metabolic process -9.262670087 8.997854763 10.06756491 28162 109 402 14 3.482587065 0.914409651
1277|1278|1306|1513|4312|4313|4314|4316|4318|4319|4321|5468|28984|55214
COL1A1|COL1A2|COL15A1|CTSK|MMP1|MMP2|MMP3|MMP7|MMP9|MMP10|MMP12|PPARG|RGCC|P3H2 -6.8284713 0 19 1 1 -9.262670087 38.21166893
1 -8.450355688 GO:0071492 M1 1 0 GO Biological Processes 19 cellular response to UV-A -8.450355688 38.21166893 14.85442719 28162 11 402 6 1.492537313 0.604761504 4312|4313|4314|4318|7076|23596
MMP1|MMP2|MMP3|MMP9|TIMP1|OPN3 -6.084614283 0 19 0 0 -9.262670087 38.21166893
1 -7.721431473 GO:0030574 M1 1 0 GO Biological Processes 19 collagen catabolic process -7.721431473 13.41473484 10.25049091 28162 47 402 9 2.23880597 0.737867616 1306|1513|4312|4313|4314|4316|4318|4319|4321
COL15A1|CTSK|MMP1|MMP2|MMP3|MMP7|MMP9|MMP10|MMP12 -5.46684052 0 19 0 0 -9.262670087 38.21166893
1 -7.653222962 GO:0070141 M1 1 0 GO Biological Processes 19 response to UV-A -7.653222962 30.02345416 13.07123662 28162 14 402 6 1.492537313 0.604761504 4312|4313|4314|4318|7076|23596
MMP1|MMP2|MMP3|MMP9|TIMP1|OPN3 -5.405580869 0 19 0 0

-9.262670087 38.21166893
1 -7.550747106 GO:0022617 M1 1 0 GO Biological Processes 19 extracellular matrix disassembly -7.550747106 9.284361326 9.095667842 28162 83 402 11 2.736318408 0.813665012
682|1513|3915|4312|4313|4314|4316|4318|4319|4321|7076
BSG|CTSK|LAMC1|MMP1|MMP2|MMP3|MMP7|MMP9|MMP10|MMP12|TIMP1
-5.323308398 0 19 0 0 -9.262670087 38.21166893
1 -6.40038318 GO:0004222 M1 1 0 GO Molecular Functions 21
metalloendopeptidase activity -6.40038318 7.201887757 7.734602271 28162
107 402 11 2.736318408 0.813665012
4224|4225|4312|4313|4314|4316|4318|4319|4321|9510|56999
MEP1A|MEP1B|MMP1|MMP2|MMP3|MMP7|MMP9|MMP10|MMP12|ADAMTS1|ADAMTS9 -4.307643047 0 19 0 0 -9.262670087 38.21166893
1 -6.227681475 GO:0008237 M1 1 0 GO Molecular Functions 21 metallopeptidase activity -6.227681475 5.216841325 6.980977378 28162 188 402 14 3.482587065 0.914409651
290|4224|4225|4312|4313|4314|4316|4318|4319|4321|9510|22802|29953|56999
ANPEP|MEP1A|MEP1B|MMP1|MMP2|MMP3|MMP7|MMP9|MMP10|MMP12|ADAMTS1|CLCA4|TRHDE|ADAMTS9 -4.153826686 0 19 0 0 -9.262670087 38.21166893
1 -5.740487909 GO:0004175 M1 1 0 GO Molecular Functions 21 endopeptidase activity -5.740487909 3.335939351 5.949818847 28162 441 402 21 5.223880597 1.109770192
629|716|834|1513|3426|4224|4225|4312|4313|4314|4316|4318|4319|4321|5122|5328|5655|9510|25825|56999|388743
CFB|C1S|CASP1|CTSK|CFI|MEP1A|MEP1B|MMP1|MMP2|MMP3|MMP7|MMP9|MMP10|MMP12|PCSK1|PLAU|KLK10|ADAMTS1|BACE2|ADAMTS9|CAPN8 -3.730931764 0 19 0 0 -9.262670087 38.21166893
1 -5.7073134 GO:0008233 M1 1 0 GO Molecular Functions 21 peptidase activity -5.7073134 2.859376586 5.712164979 28162 637 402 26 6.467661692 1.226708902
290|629|716|834|1513|3426|4224|4225|4312|4313|4314|4316|4318|4319|4321|5122|5328|5655|9510|22802|25825|29953|56999|57628|80150|388743
ANPEP|CFB|C1S|CASP1|CTSK|CFI|MEP1A|MEP1B|MMP1|MMP2|MMP3|MMP7|MMP9|MMP10|MMP12|PCSK1|PLAU|KLK10|ADAMTS1|CLCA4|BACE2|TRHDE|ADAMTS9|DPP10|ASRGL1|CAPN8 -3.699744876 0 19 0 0 -9.262670087 38.21166893
1 -5.167037744 GO:0071214 M1 1 0 GO Biological Processes 19 cellular response to abiotic stimulus -5.167037744 3.587139603 5.706366149 28162 332 402 17 4.228855721 1.003727321
834|1277|2697|3373|3553|4312|4313|4314|4316|4318|5105|5145|7076|7098|9397|10974|23596
CASP1|COL1A1|GJA1|HYAL1|IL1B|MMP1|MMP2|MMP3|MMP7|MMP9|PCK1|PDE6A|TIMP1|TLR3|NMT2|ADIRF|OPN3 -3.251994396 0 19 0 0 -9.262670087 38.21166893
1 -5.167037744 GO:0104004 M1 1 0 GO Biological Processes 19 cellular response to environmental stimulus -5.167037744 3.587139603 5.706366149 28162 332 402

17 4.228855721 1.003727321
834|1277|2697|3373|3553|4312|4313|4314|4316|4318|5105|5145|7076|7098|9397|10974|23596
CASP1|COL1A1|GJA1|HYAL1|IL1B|MMP1|MMP2|MMP3|MMP7|MMP9|PCK1|PDE6A|TIMP1|TLR3|NMT2|ADIRF|OPN3 -3.251994396 0 19 0 0 -9.262670087 38.21166893
1 -4.741585562 GO:0008236 M1 1 0 GO Molecular Functions 21 serine-type peptidase activity -4.741585562 4.471578279 5.747195011 28162 188 402 12 2.985074627 0.848757934
629|716|3426|4312|4313|4314|4316|4318|5122|5328|5655|57628
CFB|C1S|CFI|MMP1|MMP2|MMP3|MMP7|MMP9|PCSK1|PLAU|KLK10|DPP10
-2.88212364 0 19 0 0 -9.262670087 38.21166893
1 -4.695828867 GO:0017171 M1 1 0 GO Molecular Functions 21 serine hydrolase activity -4.695828867 4.424509034 5.699551587 28162 190 402 12 2.985074627 0.848757934 629|716|3426|4312|4313|4314|4316|4318|5122|5328|5655|57628
CFB|C1S|CFI|MMP1|MMP2|MMP3|MMP7|MMP9|PCSK1|PLAU|KLK10|DPP10
-2.84887549 0 19 0 0 -9.262670087 38.21166893
1 -4.44873216 GO:0004252 M1 1 0 GO Molecular Functions 21 serine-type endopeptidase activity -4.44873216 4.532952883 5.559972882 28162 170 402 11 2.736318408 0.813665012
629|716|3426|4312|4313|4314|4316|4318|5122|5328|5655
CFB|C1S|CFI|MMP1|MMP2|MMP3|MMP7|MMP9|PCSK1|PLAU|KLK10 -2.653387738 0 19 0 0 -9.262670087 38.21166893
1 -3.839777698 GO:0071482 M1 1 0 GO Biological Processes 19 cellular response to light stimulus -3.839777698 4.670315091 5.144098297 28162 135 402 9 2.23880597 0.737867616 3373|4312|4313|4314|4318|5145|7076|9397|23596
HYAL1|MMP1|MMP2|MMP3|MMP9|PDE6A|TIMP1|NMT2|OPN3 -2.15734605 0 19 0 0 -9.262670087 38.21166893
1 -3.557350717 GO:0009314 M1 1 0 GO Biological Processes 19 response to radiation -3.557350717 2.65832667 4.257815298 28162 448 402 17 4.228855721 1.003727321
301|1281|3373|3627|4312|4313|4314|4318|5145|6356|7056|7076|9397|10974|23596|23657|121457
ANXA1|COL3A1|HYAL1|CXCL10|MMP1|MMP2|MMP3|MMP9|PDE6A|CCL11|THBD|TIMP1|NMT2|ADIRF|OPN3|SLC7A11|IKBIP -1.928669366 0 19 0 0 -9.262670087 38.21166893
1 -3.538987991 GO:0034644 M1 1 0 GO Biological Processes 19 cellular response to UV -3.538987991 5.509922299 5.127987784 28162 89 402 7 1.741293532 0.652391792 3373|4312|4313|4314|4318|7076|23596
HYAL1|MMP1|MMP2|MMP3|MMP9|TIMP1|OPN3 -1.914431025 0 19 0 0 -9.262670087 38.21166893
1 -3.331804253 GO:0071478 M1 1 0 GO Biological Processes 19 cellular response to radiation -3.331804253 3.648683665 4.431612781 28162 192 402 10 2.487562189 0.776790586 3373|4312|4313|4314|4318|5145|7076|9397|10974|23596
HYAL1|MMP1|MMP2|MMP3|MMP9|PDE6A|TIMP1|NMT2|ADIRF|OPN3

-1.745719453 0 19 0 0 -9.262670087 38.21166893
1 -2.987400134 GO:1904645 M1 1 0 GO Biological Processes 19 response to amyloid-beta -2.987400134 6.486548738 4.856333247 28162 54 402 5 1.243781095 0.552765814 2697|4313|4314|4318|4321 GJA1|MMP2|MMP3|MMP9|MMP12 -1.469334192 0 19 0 0 -9.262670087 38.21166893
1 -2.468679459 GO:0022411 M1 1 0 GO Biological Processes 19 cellular component disassembly -2.468679459 2.107841324 3.201092177 28162 565 402 17 4.228855721 1.003727321 682|718|857|1513|3915|4312|4313|4314|4316|4318|4319|4321|7076|10891|11240|85477|130399 BSG|C3|CAV1|CTSK|LAMC1|MMP1|MMP2|MMP3|MMP7|MMP9|MMP10|MMP12|TIMP1|PPARGC1A|PADI2|SCIN|ACVR1C -1.067119086 0 19 0 0 -9.262670087 38.21166893
1 -2.275850012 GO:0009411 M1 1 0 GO Biological Processes 19 response to UV -2.275850012 3.335939351 3.4170572 28162 147 402 7 1.741293532 0.652391792 3373|4312|4313|4314|4318|7076|23596 HYAL1|MMP1|MMP2|MMP3|MMP9|TIMP1|OPN3 -0.921273604 0 19 0 0 -9.262670087 38.21166893
1 -9.136645356 GO:1901652 M1 1 0 GO Biological Processes 19 response to peptide -9.136645356 3.862332823 7.974486785 28162 526 402 29 7.213930348 1.290370231 301|682|857|1277|1906|2690|2697|3488|3553|4067|4313|4314|4318|4321|5068|5105|5166|5468|5967|5968|6678|7031|7076|11031|23433|51237|55198|55532|130399 ANXA1|BSG|CAV1|COL1A1|EDN1|GHR|GJA1|IGFBP5|IL1B|LYN|MMP2|MMP3|MMP9|MMP12|REG3A|PCK1|PDK4|PPARG|REG1A|REG1B|SPARC|TFF1|TIMP1|RAB31|RHOQ|MZB1|APPL2|SLC30A10|ACVR1C -6.723131169 0 20 1 1 -9.136645356 4.007707458
1 -8.270563373 GO:0043434 M1 1 0 GO Biological Processes 19 response to peptide hormone -8.270563373 4.007707458 7.625491929 28162 437 402 25 6.218905473 1.204485566 301|682|857|1277|1906|2690|2697|3488|3553|4067|5068|5105|5166|5468|5967|5968|6678|7031|7076|11031|23433|51237|55198|55532|130399 ANXA1|BSG|CAV1|COL1A1|EDN1|GHR|GJA1|IGFBP5|IL1B|LYN|REG3A|PCK1|PDK4|PPARG|REG1A|REG1B|SPARC|TFF1|TIMP1|RAB31|RHOQ|MZB1|APPL2|SLC30A10|ACVR1C -5.943740034 0 20 0 0 -9.136645356 4.007707458
1 -6.912966956 GO:1901699 M1 1 0 GO Biological Processes 19 cellular response to nitrogen compound -6.912966956 2.989533131 6.428561627 28162 703 402 30 7.462686567 1.31066897 343|366|857|1277|1278|1282|1290|1906|2690|2697|2769|3488|3553|4067|4313|4314|5028|5105|5166|5468|5967|6505|7098|10891|11031|23433|51237|55151|55198|55532 AQP8|AQP9|CAV1|COL1A1|COL1A2|COL4A1|COL5A2|EDN1|GHR|GJA1|GNA15|IGFBP5|IL1B|LYN|MMP2|MMP3|P2RY1|PCK1|PDK4|PPARG|REG1A|SLC1A1|TLR3|PPARGC1A|RAB31|RHOQ|MZB1|TMEM38B|APPL2|SLC30A10 -4.746797905 0 20 0 0 -9.136645356 4.007707458

1 -6.075906651 GO:0071417 M1 1 0 GO Biological Processes 19 cellular response to organonitrogen compound -6.075906651 2.918946932 5.947041005 28162 648 402 27 6.71641791 1.248413442 343|366|857|1277|1278|1282|1290|1906|2690|2697|2769|3488|3553|4067|4313|5028|5105|5166|5468|5967|10891|11031|23433|51237|55151|55198|55532 AQP8|AQP9|CAV1|COL1A1|COL1A2|COL4A1|COL5A2|EDN1|GHR|GJA1|GNA15|IGFBP5|IL1B|LYN|MMP2|P2RY1|PCK1|PDK4|PPARG|REG1A|PPARGC1A|RAB31|RHOQ|MZB1|TMEM38B|APPL2|SLC30A10 -4.03109007 0 20 0 0 -9.136645356 4.007707458

1 -4.91773769 GO:0032870 M1 1 0 GO Biological Processes 19 cellular response to hormone stimulus -4.91773769 2.707429038 5.177569564 28162 621 402 24 5.970149254 1.181714164 301|687|857|1906|2690|2697|3371|3490|3553|4067|5105|5166|5272|5468|5967|6338|7068|10891|11031|11240|23433|51237|55198|55532 ANXA1|KLF9|CAV1|EDN1|GHR|GJA1|TNC|IGFBP7|IL1B|LYN|PCK1|PDK4|SERPINB9|PPARG|REG1A|SCNN1B|THRB|PPARGC1A|RAB31|PADI2|RHOQ|MZB1|APPL2|SLC30A10 -3.033618041 0 20 0 0 -9.136645356 4.007707458

1 -3.54365849 GO:0071375 M1 1 0 GO Biological Processes 19 cellular response to peptide hormone stimulus -3.54365849 2.999284921 4.376029952 28162 327 402 14 3.482587065 0.914409651 857|1906|2690|3553|4067|5105|5166|5468|5967|11031|23433|51237|55198|55532 CAV1|EDN1|GHR|IL1B|LYN|PCK1|PDK4|PPARG|REG1A|RAB31|RHOQ|MZB1|APPL2|SLC30A10 -1.918279774 0 20 0 0 -9.136645356 4.007707458

1 -3.196980507 GO:1901653 M1 1 0 GO Biological Processes 19 cellular response to peptide -3.196980507 2.65358812 3.98791239 28162 396 402 15 3.731343284 0.945283402 857|1906|2690|2697|3553|4067|5105|5166|5468|5967|11031|23433|51237|55198|55532 CAV1|EDN1|GHR|GJA1|IL1B|LYN|PCK1|PDK4|PPARG|REG1A|RAB31|RHOQ|MZB1|APPL2|SLC30A10 -1.633585161 0 20 0 0 -9.136645356 4.007707458

1 -2.135881987 GO:0032868 M1 1 0 GO Biological Processes 19 response to insulin -2.135881987 2.501954513 3.039493132 28162 280 402 10 2.487562189 0.776790586 3553|4067|5105|5166|5468|11031|23433|51237|55198|130399 IL1B|LYN|PCK1|PDK4|PPARG|RAB31|RHOQ|MZB1|APPL2|ACVR1C -0.80781603 0 20 0 0 -9.136645356 4.007707458

1 -8.549068626 GO:0032526 M1 1 0 GO Biological Processes 19 response to retinoic acid -8.549068626 8.841858668 9.594643896 28162 103 402 13 3.233830846 0.882281956 360|952|1277|2263|2697|3294|3371|3490|4067|5105|5468|7474|339479 AQP3|CD38|COL1A1|FGFR2|GJA1|HSD17B2|TNC|IGFBP7|LYN|PCK1|PPARG|WNT5A|BRINP3 -6.174183841 0 21 1 1 -8.549068626 8.841858668

1 -5.450801136 GO:0071300 M1 1 0 GO Biological Processes 19 cellular response to retinoic acid -5.450801136 8.756840796 7.475917374 28162 64 402 8 1.990049751 0.696552772 1277|2263|3371|4067|5105|5468|7474|339479 COL1A1|FGFR2|TNC|LYN|PCK1|PPARG|WNT5A|BRINP3 -3.48480554 0 21 0 0

-8.549068626 8.841858668
1 -3.08714425 GO:0048608 M1 1 0 GO Biological Processes 19 reproductive structure development -3.08714425 2.588228807 3.879046432 28162 406 402 15 3.731343284 0.945283402 301|682|718|2263|2697|3294|3371|5268|5270|5468|6696|7098|7474|9510|56159 ANXA1|BSG|C3|FGFR2|GJA1|HSD17B2|TNC|SERPINB5|SERPINE2|PPARG|SPP1|TLR3|WNT5A|ADAMTS1|TEX11 -1.541220869 0 21 0 0 -8.549068626 8.841858668
1 -3.054994218 GO:0061458 M1 1 0 GO Biological Processes 19 reproductive system development -3.054994218 2.569244243 3.847021046 28162 409 402 15 3.731343284 0.945283402 301|682|718|2263|2697|3294|3371|5268|5270|5468|6696|7098|7474|9510|56159 ANXA1|BSG|C3|FGFR2|GJA1|HSD17B2|TNC|SERPINB5|SERPINE2|PPARG|SPP1|TLR3|WNT5A|ADAMTS1|TEX11 -1.519226276 0 21 0 0 -8.549068626 8.841858668
1 -8.509472284 GO:0052547 M1 1 0 GO Biological Processes 19 regulation of peptidase activity -8.509472284 3.976905864 7.729545058 28162 458 402 26 6.467661692 1.226708902 12|183|718|834|857|1293|1942|4067|4318|5266|5268|5270|5271|5272|5468|6091|6279|6280|6505|7057|7076|7980|11005|27074|27290|130399 SERPINA3|AGT|C3|CASP1|CAV1|COL6A3|EFNA1|LYN|MMP9|PI3|SERPINB5|SERPINE2|SERPINB8|SERPINB9|PPARG|ROBO1|S100A8|S100A9|SLC1A1|THBS1|TIMP1|TFPI2|SPINK5|LAMP3|SPINK4|ACVR1C -6.139183251 0 22 1 1 -8.509472284 7.863285613
1 -8.431706725 GO:0052548 M1 1 0 GO Biological Processes 19 regulation of endopeptidase activity -8.431706725 4.082443262 7.741990668 28162 429 402 25 6.218905473 1.204485566 12|183|718|834|1293|1942|4067|4318|5266|5268|5270|5271|5272|5468|6091|6279|6280|6505|7057|7076|7980|11005|27074|27290|130399 SERPINA3|AGT|C3|CASP1|COL6A3|EFNA1|LYN|MMP9|PI3|SERPINB5|SERPINE2|SERPINB8|SERPINB9|PPARG|ROBO1|S100A8|S100A9|SLC1A1|THBS1|TIMP1|TFPI2|SPINK5|LAMP3|SPINK4|ACVR1C -6.070465821 0 22 0 0 -8.509472284 7.863285613
1 -6.792355102 GO:0004867 M1 1 0 GO Molecular Functions 21 serine-type endopeptidase inhibitor activity -6.792355102 7.863285613 8.190259388 28162 98 402 11 2.736318408 0.813665012 12|183|1293|5266|5268|5270|5271|5272|7980|11005|27290 SERPINA3|AGT|COL6A3|PI3|SERPINB5|SERPINE2|SERPINB8|SERPINB9|TFPI2|SPINK5|SPINK4 -4.640242116 0 22 0 0 -8.509472284 7.863285613
1 -6.477100328 GO:0030162 M1 1 0 GO Biological Processes 19 regulation of proteolysis -6.477100328 2.851617084 6.129696008 28162 737 402 30 7.462686567 1.31066897 12|183|718|834|857|1293|1942|3553|4067|4318|5266|5268|5270|5271|5272|5468|6091|6279|6280|6505|7057|7076|7980|11005|27074|27290|28951|90865|130399|282679 SERPINA3|AGT|C3|CASP1|CAV1|COL6A3|EFNA1|IL1B|LYN|MMP9|PI3|SERPINB5|SERPINE2|SERPINB8|SERPINB9|PPARG|ROBO1|S100A8|S100A9|SLC1A1|THBS1|TIMP1|TFPI2|SPINK5|LAMP3|SPINK4|TRIB2|IL33|ACVR1C|AQP11 -4.364616137 0 22 0 0 -8.509472284 7.863285613

1 -6.257273913 GO:0010951 M1 1 0 GO Biological Processes 19 negative regulation of endopeptidase activity -6.257273913 4.575002538 6.763201944 28162 245 402 16 3.980099502 0.975022334 12|183|718|1293|4318|5266|5268|5270|5271|5272|7057|7076|7980|11005|27074|27290 SERPINA3|AGT|C3|COL6A3|MMP9|PI3|SERPINB5|SERPINE2|SERPINB8|SERPINB9|THBS1|TIMP1|TFPI2|SPINK5|LAMP3|SPINK4 -4.181102881 0 22 0 0 -8.509472284 7.863285613

1 -5.982014574 GO:0010466 M1 1 0 GO Biological Processes 19 negative regulation of peptidase activity -5.982014574 4.361383743 6.514348306 28162 257 402 16 3.980099502 0.975022334 12|183|718|1293|4318|5266|5268|5270|5271|5272|7057|7076|7980|11005|27074|27290 SERPINA3|AGT|C3|COL6A3|MMP9|PI3|SERPINB5|SERPINE2|SERPINB8|SERPINB9|THBS1|TIMP1|TFPI2|SPINK5|LAMP3|SPINK4 -3.945755797 0 22 0 0 -8.509472284 7.863285613

1 -5.896200688 GO:0061134 M1 1 0 GO Molecular Functions 21 peptidase regulator activity -5.896200688 4.568786502 6.539744796 28162 230 402 15 3.731343284 0.945283402 12|183|718|834|857|1293|5266|5268|5270|5271|5272|7076|7980|11005|27290 SERPINA3|AGT|C3|CASP1|CAV1|COL6A3|PI3|SERPINB5|SERPINE2|SERPINB8|SERPINB9|TIMP1|TFPI2|SPINK5|SPINK4 -3.866251384 0 22 0 0 -8.509472284 7.863285613

1 -5.676649228 GO:0004866 M1 1 0 GO Molecular Functions 21 endopeptidase inhibitor activity -5.676649228 5.059508015 6.575028076 28162 180 402 13 3.233830846 0.882281956 12|183|718|1293|5266|5268|5270|5271|5272|7076|7980|11005|27290 SERPINA3|AGT|C3|COL6A3|PI3|SERPINB5|SERPINE2|SERPINB8|SERPINB9|TIMP1|TFPI2|SPINK5|SPINK4 -3.673028863 0 22 0 0 -8.509472284 7.863285613

1 -5.53224477 GO:0045861 M1 1 0 GO Biological Processes 19 negative regulation of proteolysis -5.53224477 3.644465534 5.956017341 28162 346 402 18 4.47761194 1.031484687 12|183|718|1293|1942|4318|5266|5268|5270|5271|5272|7057|7076|7980|11005|27074|27290|282679 SERPINA3|AGT|C3|COL6A3|EFNA1|MMP9|PI3|SERPINB5|SERPINE2|SERPINB8|SERPINB9|THBS1|TIMP1|TFPI2|SPINK5|LAMP3|SPINK4|AQP11 -3.542166979 0 22 0 0 -8.509472284 7.863285613

1 -5.492836282 GO:0030414 M1 1 0 GO Molecular Functions 21 peptidase inhibitor activity -5.492836282 4.870114667 6.389794931 28162 187 402 13 3.233830846 0.882281956 12|183|718|1293|5266|5268|5270|5271|5272|7076|7980|11005|27290 SERPINA3|AGT|C3|COL6A3|PI3|SERPINB5|SERPINE2|SERPINB8|SERPINB9|TIMP1|TFPI2|SPINK5|SPINK4 -3.514829894 0 22 0 0 -8.509472284 7.863285613

1 -5.455988607 GO:0004857 M1 1 0 GO Molecular Functions 21 enzyme inhibitor activity -5.455988607 3.44828964 5.828394334 28162 386 402 19 4.726368159

1.058368993
12|183|301|308|718|1030|1293|5266|5268|5270|5271|5272|5570|7076|7453|7980|11005|27290|28951
SERPINA3|AGT|ANXA1|ANXA5|C3|CDKN2B|COL6A3|PI3|SERPINB5|SERPINE2|SERPINB8|SERPINB9|PKIB|TIMP1|WARS1|TFPI2|SPINK5|SPINK4|TRIB2 -3.487081851 0 22 0 0
-8.509472284 7.863285613
1 -5.366749757 GO:0061135 M1 1 0 GO Molecular Functions 21 endopeptidase regulator activity -5.366749757 4.743288765 6.263037958 28162 192 402 13
3.233830846 0.882281956
12|183|718|1293|5266|5268|5270|5271|5272|7076|7980|11005|27290
SERPINA3|AGT|C3|COL6A3|PI3|SERPINB5|SERPINE2|SERPINB8|SERPINB9|TIMP1|TFPI2|SPINK5|SPINK4 -3.413188799 0 22 0 0 -8.509472284 7.863285613
1 -3.469029762 GO:0051346 M1 1 0 GO Biological Processes 19 negative regulation of hydrolase activity -3.469029762 2.61168936 4.175458554 28162 456
402 17 4.228855721 1.003727321
12|183|301|718|1293|4318|5266|5268|5270|5271|5272|7057|7076|7980|11005|27074|27290
SERPINA3|AGT|ANXA1|C3|COL6A3|MMP9|PI3|SERPINB5|SERPINE2|SERPINB8|SERPINB9|THBS1|TIMP1|TFPI2|SPINK5|LAMP3|SPINK4 -1.862168722 0 22 0 0
-8.509472284 7.863285613
1 -8.380651988 GO:0042908 M1 1 0 GO Biological Processes 19 xenobiotic transport -8.380651988 15.76231343 11.24316664 28162 40 402 9 2.23880597
0.737867616 2697|5174|5243|6533|6584|8140|9076|9429|206358
GJA1|PDZK1|ABCB1|SLC6A6|SLC22A5|SLC7A5|CLDN1|ABCG2|SLC36A1 -6.028274544 0
23 1 1 -8.380651988 42.03283582
1 -5.322664099 GO:0010232 M1 1 0 GO Biological Processes 19 vascular transport
-5.322664099 7.164687924 6.96991716 28162 88 402 9 2.23880597
0.737867616 2697|5243|6505|6533|6566|6584|8140|8671|9429
GJA1|ABCB1|SLC1A1|SLC6A6|SLC16A1|SLC22A5|SLC7A5|SLC4A4|ABCG2
-3.379485178 0 23 0 0 -8.380651988 42.03283582
1 -4.548790617 GO:0098591 M1 1 0 GO Cellular Components20 external side of apical plasma membrane -4.548790617 42.03283582 11.04207458 28162 5 402
3 0.746268657 0.429247719 5243|8140|9429 ABCB1|SLC7A5|ABCG2
-2.738597074 0 23 0 0 -8.380651988 42.03283582
1 -4.454042211 GO:0150104 M1 1 0 GO Biological Processes 19 transport across blood-brain barrier -4.454042211 6.441813919 6.117454712 28162 87 402 8
1.990049751 0.696552772 5243|6505|6533|6566|6584|8140|8671|9429
ABCB1|SLC1A1|SLC6A6|SLC16A1|SLC22A5|SLC7A5|SLC4A4|ABCG2 -2.657479571 0 23
0 0 -8.380651988 42.03283582
1 -4.211879239 GO:0042910 M1 1 0 GO Molecular Functions 21 xenobiotic transmembrane transporter activity -4.211879239 11.67578773 7.040241953 28162
30 402 5 1.243781095 0.552765814 5243|6533|6584|9429|206358
ABCB1|SLC6A6|SLC22A5|ABCG2|SLC36A1 -2.461561787 0 23 0 0
-8.380651988 42.03283582

1 -3.029274715 GO:0015562 M1 1 0 GO Molecular Functions 21 efflux transmembrane transporter activity -3.029274715 15.01172708 6.31043428 28162 14 402 3 0.746268657 0.429247719 2697|5243|9429 GJA1|ABCB1|ABCG2 -1.500806012 0 23 0 0 -8.380651988 42.03283582

1 -8.302533439 GO:0002544 M1 1 0 GO Biological Processes 19 chronic inflammatory response -8.302533439 25.80963603 13.01785201 28162 19 402 7 1.741293532 0.652391792 2697|3620|6279|6280|6356|7057|8876 GJA1|IDO1|S100A8|S100A9|CCL11|THBS1|VNN1 -5.964633591 0 24 1 1 -8.302533439 35.02736318

1 -5.095538291 GO:0050786 M1 1 0 GO Molecular Functions 21 RAGE receptor binding -5.095538291 28.02189055 10.2846244 28162 10 402 4 0.995024876 0.495031064 2357|6279|6280|6283 FPR1|S100A8|S100A9|S100A12 -3.185276215 0 24 0 0 -8.302533439 35.02736318

1 -4.578220964 GO:0070486 M1 1 0 GO Biological Processes 19 leukocyte aggregation -4.578220964 21.55530042 8.920544234 28162 13 402 4 0.995024876 0.495031064 3553|4478|6279|6280 IL1B|MSN|S100A8|S100A9 -2.755253132 0 24 0 0 -8.302533439 35.02736318

1 -4.25239421 GO:0050544 M1 1 0 GO Molecular Functions 21 arachidonic acid binding -4.25239421 35.02736318 10.03103568 28162 6 402 3 0.746268657 0.429247719 5468|6279|6280 PPARG|S100A8|S100A9 -2.494331172 0 24 0 0 -8.302533439 35.02736318

1 -4.013986523 GO:0050542 M1 1 0 GO Molecular Functions 21 icosanoid binding -4.013986523 30.02345416 9.241610993 28162 7 402 3 0.746268657 0.429247719 5468|6279|6280 PPARG|S100A8|S100A9 -2.305460374 0 24 0 0 -8.302533439 35.02736318

1 -4.013986523 GO:0050543 M1 1 0 GO Molecular Functions 21 icosatetraenoic acid binding -4.013986523 30.02345416 9.241610993 28162 7 402 3 0.746268657 0.429247719 5468|6279|6280 PPARG|S100A8|S100A9 -2.305460374 0 24 0 0 -8.302533439 35.02736318

1 -3.705744461 GO:0006882 M1 1 0 GO Biological Processes 19 cellular zinc ion homeostasis -3.705744461 9.217727154 6.100034669 28162 38 402 5 1.243781095 0.552765814 4499|6279|6280|6505|55532 MT1M|S100A8|S100A9|SLC1A1|SLC30A10 -2.042577184 0 24 0 0 -8.302533439 35.02736318

1 -3.666965934 GO:0043281 M1 1 0 GO Biological Processes 19 regulation of cysteine-type endopeptidase activity involved in apoptotic process -3.666965934 3.70481726 4.711670897 28162 208 402 11 2.736318408 0.813665012 834|4318|5272|5468|6091|6279|6280|6505|7057|27074|130399 CASP1|MMP9|SERPINB9|PPARG|ROBO1|S100A8|S100A9|SLC1A1|THBS1|LAMP3|ACVR1C -2.010918398 0 24 0 0 -8.302533439 35.02736318

1 -3.651236676 GO:0050832 M1 1 0 GO Biological Processes 19 defense response to fungus -3.651236676 8.981375175 6.00214576 28162 39 402 5 1.243781095 0.552765814 1670|1671|6279|6280|6283DEFA5|DEFA6|S100A8|S100A9|S100A12 -1.998705714 0 24 0 0 -8.302533439 35.02736318

1 -3.598320886 GO:0055069 M1 1 0 GO Biological Processes 19 zinc ion homeostasis -3.598320886 8.756840796 5.907709119 28162 40 402 5 1.243781095 0.552765814 4499|6279|6280|6505|55532 MT1M|S100A8|S100A9|SLC1A1|SLC30A10 -1.962957949 0 24 0 0 -8.302533439 35.02736318

1 -3.248871258 GO:2000116 M1 1 0 GO Biological Processes 19 regulation of cysteine-type endopeptidase activity -3.248871258 3.307304678 4.255813091 28162 233 402 11 2.736318408 0.813665012 834|4318|5272|5468|6091|6279|6280|6505|7057|27074|130399 CASP1|MMP9|SERPINB9|PPARG|ROBO1|S100A8|S100A9|SLC1A1|THBS1|LAMP3|ACVR1C -1.678285442 0 24 0 0 -8.302533439 35.02736318

1 -3.143910417 GO:0010952 M1 1 0 GO Biological Processes 19 positive regulation of peptidase activity -3.143910417 3.450971742 4.217478119 28162 203 402 10 2.487562189 0.776790586 834|857|1942|4067|5468|6091|6279|6280|6505|130399 CASP1|CAV1|EFNA1|LYN|PPARG|ROBO1|S100A8|S100A9|SLC1A1|ACVR1C -1.588289468 0 24 0 0 -8.302533439 35.02736318

1 -3.129402513 GO:0036041 M1 1 0 GO Molecular Functions 21 long-chain fatty acid binding -3.129402513 16.16647532 6.581913723 28162 13 402 3 0.746268657 0.429247719 5468|6279|6280 PPARG|S100A8|S100A9 -1.577269892 0 24 0 0 -8.302533439 35.02736318

1 -3.049581341 GO:0055076 M1 1 0 GO Biological Processes 19 transition metal ion homeostasis -3.049581341 4.031926697 4.312172403 28162 139 402 8 1.990049751 0.696552772 3934|4499|6279|6280|6505|9429|55532|79689 LCN2|MT1M|S100A8|S100A9|SLC1A1|ABCG2|SLC30A10|STEAP4 -1.514984205 0 24 0 0 -8.302533439 35.02736318

1 -3.039509772 GO:0051345 M1 1 0 GO Biological Processes 19 positive regulation of hydrolase activity -3.039509772 2.068368044 3.638952922 28162 779 402 23 5.721393035 1.158362377 834|857|1942|2263|2769|4067|5468|6091|6279|6280|6356|6362|6505|7474|8490|9912|10451|10979|23255|26286|57733|63928|130399 CASP1|CAV1|EFNA1|FGFR2|GNA15|LYN|PPARG|ROBO1|S100A8|S100A9|CCL11|CCL18|SLC1A1|WNT5A|RGS5|ARHGAP44|VAV3|FERMT2|MTCL1|ARFGAP3|GBA3|CHP2|ACVR1C -1.506410315 0 24 0 0 -8.302533439 35.02736318

1 -3.024546631 GO:0009620 M1 1 0 GO Biological Processes 19 response to fungus -3.024546631 6.60893645 4.918391422 28162 53 402 5 1.243781095 0.552765814 1670|1671|6279|6280|6283DEFA5|DEFA6|S100A8|S100A9|S100A12 -1.499355638 0 24 0 0 -8.302533439 35.02736318

1 -2.85139329 GO:0017014 M1 1 0 GO Biological Processes 19 protein nitrosylation -2.85139329 13.13526119 5.842895378 28162 16 402 3 0.746268657 0.429247719 4843|6279|6280 NOS2|S100A8|S100A9 -1.363728798 0 24 0 0 -8.302533439 35.02736318

1 -2.85139329 GO:0018119 M1 1 0 GO Biological Processes 19 peptidyl-cysteine S-nitrosylation -2.85139329 13.13526119 5.842895378 28162 16 402 3 0.746268657 0.429247719 4843|6279|6280 NOS2|S100A8|S100A9 -1.363728798 0

24 0 0 -8.302533439 35.02736318
1 -2.85139329 GO:0051238 M1 1 0 GO Biological Processes 19 sequestering of metal ion -2.85139329 13.13526119 5.842895378 28162 16 402 3 0.746268657 0.429247719 3934|6279|6280 LCN2|S100A8|S100A9 -1.363728798 0 24 0 0 -8.302533439 35.02736318
1 -2.845359753 GO:0010950 M1 1 0 GO Biological Processes 19 positive regulation of endopeptidase activity -2.845359753 3.408067769 3.954406667 28162 185 402 9 2.23880597 0.737867616 834|1942|4067|5468|6091|6279|6280|6505|130399 CASP1|EFNA1|LYN|PPARG|ROBO1|S100A8|S100A9|SLC1A1|ACVR1C -1.359478494 0 24 0 0 -8.302533439 35.02736318
1 -2.807953792 GO:0046916 M1 1 0 GO Biological Processes 19 cellular transition metal ion homeostasis -2.807953792 4.155788852 4.133865042 28162 118 402 7 1.741293532 0.652391792 3934|4499|6279|6280|6505|9429|55532 LCN2|MT1M|S100A8|S100A9|SLC1A1|ABCG2|SLC30A10 -1.326228493 0 24 0 0 -8.302533439 35.02736318
1 -2.707997312 GO:0006919 M1 1 0 GO Biological Processes 19 activation of cysteine-type endopeptidase activity involved in apoptotic process -2.707997312 4.618992947 4.161087255 28162 91 402 6 1.492537313 0.604761504 834|5468|6091|6279|6280|6505CASP1|PPARG|ROBO1|S100A8|S100A9|SLC1A1 -1.252079276 0 24 0 0 -8.302533439 35.02736318
1 -2.703960949 GO:0031406 M1 1 0 GO Molecular Functions 21 carboxylic acid binding -2.703960949 3.249961533 3.784186957 28162 194 402 9 2.23880597 0.737867616 51|4843|5105|5468|6279|6280|6505|6999|55214 ACOX1|NOS2|PCK1|PPARG|S100A8|S100A9|SLC1A1|TDO2|P3H2 -1.249155064 0 24 0 0 -8.302533439 35.02736318
1 -2.697083141 GO:0002523 M1 1 0 GO Biological Processes 19 leukocyte migration involved in inflammatory response -2.697083141 11.67578773 5.452185248 28162 18 402 3 0.746268657 0.429247719 240|6279|6280 ALOX5|S100A8|S100A9 -1.24394016 0 24 0 0 -8.302533439 35.02736318
1 -2.686650474 GO:0005504 M1 1 0 GO Molecular Functions 21 fatty acid binding -2.686650474 7.374181723 4.73156735 28162 38 402 4 0.995024876 0.495031064 51|5468|6279|6280 ACOX1|PPARG|S100A8|S100A9 -1.237911023 0 24 0 0 -8.302533439 35.02736318
1 -2.574095933 GO:0042063 M1 1 0 GO Biological Processes 19 gliogenesis -2.574095933 2.72297523 3.505588921 28162 283 402 11 2.736318408 0.813665012 301|3553|4067|5028|5270|5468|6279|6280|25825|57211|90865 ANXA1|IL1B|LYN|P2RY1|SERPINE2|PPARG|S100A8|S100A9|BACE2|ADGRG6|IL33 -1.142010506 0 24 0 0 -8.302533439 35.02736318
1 -2.549607415 GO:0045862 M1 1 0 GO Biological Processes 19 positive regulation of proteolysis -2.549607415 2.454747824 3.394297591 28162 371 402 13 3.233830846 0.882281956 834|857|1942|3553|4067|5468|6091|6279|6280|6505|28951|90865|130399 CASP1|CAV1|EFNA1|IL1B|LYN|PPARG|ROBO1|S100A8|S100A9|SLC1A1|TRIB2|IL33|ACVR1C -1.126906868 0 24 0 0 -8.302533439 35.02736318

1 -2.425119983 GO:0043280 M1 1 0 GO Biological Processes 19 positive regulation of cysteine-type endopeptidase activity involved in apoptotic process -2.425119983 3.553500613 3.618576675 28162 138 402 7 1.741293532 0.652391792 834|5468|6091|6279|6280|6505|130399 CASP1|PPARG|ROBO1|S100A8|S100A9|SLC1A1|ACVR1C -1.034714632 0 24 0 0 -8.302533439 35.02736318

1 -2.167983422 GO:2001056 M1 1 0 GO Biological Processes 19 positive regulation of cysteine-type endopeptidase activity -2.167983422 3.184305744 3.270845807 28162 154 402 7 1.741293532 0.652391792 834|5468|6091|6279|6280|6505|130399 CASP1|PPARG|ROBO1|S100A8|S100A9|SLC1A1|ACVR1C -0.833650481 0 24 0 0 -8.302533439 35.02736318

1 -2.067697073 GO:0043177 M1 1 0 GO Molecular Functions 21 organic acid binding -2.067697073 3.417303725 3.23317974 28162 123 402 6 1.492537313 0.604761504 4843|5468|6279|6280|6505|55214 NOS2|PPARG|S100A8|S100A9|SLC1A1|P3H2 -0.753510129 0 24 0 0 -8.302533439 35.02736318

1 -8.278491748 GO:0030020 M1 1 0 GO Molecular Functions 21 extracellular matrix structural constituent conferring tensile strength -8.278491748 15.37786676 11.08659888 28162 41 402 9 2.23880597 0.737867616 1277|1278|1281|1282|1290|1292|1293|1303|1306 COL1A1|COL1A2|COL3A1|COL4A1|COL5A2|COL6A2|COL6A3|COL12A1|COL15A1 -5.947512449 0 25 1 1 -8.278491748 25.47444595

1 -6.32525551 GO:0005581 M1 1 0 GO Cellular Components 20 collagen trimer -6.32525551 8.052267399 7.927857709 28162 87 402 10 2.487562189 0.776790586 1277|1278|1281|1282|1290|1292|1293|1303|1306|115908 COL1A1|COL1A2|COL3A1|COL4A1|COL5A2|COL6A2|COL6A3|COL12A1|COL15A1|CTHRC1 -4.2444146 0 25 0 0 -8.278491748 25.47444595

1 -6.133841896 GO:0019838 M1 1 0 GO Molecular Functions 21 growth factor binding -6.133841896 6.091715336 7.21549165 28162 138 402 12 2.985074627 0.848757934 1277|1278|1281|1282|2263|2690|3488|3490|3557|7057|7837|130399 COL1A1|COL1A2|COL3A1|COL4A1|FGFR2|GHR|IGFBP5|IGFBP7|IL1RN|THBS1|PXDN|ACVR1C -4.078853581 0 25 0 0 -8.278491748 25.47444595

1 -6.025609062 GO:0030199 M1 1 0 GO Biological Processes 19 collagen fibril organization -6.025609062 10.37847798 8.30121386 28162 54 402 8 1.990049751 0.696552772 1277|1278|1281|1290|1303|4017|7837|26585 COL1A1|COL1A2|COL3A1|COL5A2|COL12A1|LOXL2|PXDN|GREM1 -3.985092462 0 25 0 0 -8.278491748 25.47444595

1 -5.011267619 GO:0098644 M1 1 0 GO Cellular Components 20 complex of collagen trimers -5.011267619 16.67969675 8.64977629 28162 21 402 5 1.243781095 0.552765814 1277|1278|1281|1282|1290 COL1A1|COL1A2|COL3A1|COL4A1|COL5A2 -3.114087232 0 25 0 0 -8.278491748 25.47444595

1 -4.904169045 GO:0048407 M1 1 0 GO Molecular Functions 21 platelet-derived growth factor binding -4.904169045 25.47444595 9.769889016 28162 11 402 4

0.995024876 0.495031064 1277|1278|1281|1282 COL1A1|COL1A2|COL3A1|COL4A1 -3.025978422 0 25 0 0 -8.278491748 25.47444595

1 -4.795808538 GO:0043200 M1 1 0 GO Biological Processes 19 response to amino acid -4.795808538 6.181299385 6.308444286 28162 102 402 9 2.23880597 0.737867616 1277|1278|1282|1290|1906|4067|4313|5105|10891 COL1A1|COL1A2|COL4A1|COL5A2|EDN1|LYN|MMP2|PCK1|PPARGC1A -2.933517326 0 25 0 0 -8.278491748 25.47444595

1 -4.733000928 GO:0005583 M1 1 0 GO Cellular Components20 fibrillar collagen trimer -4.733000928 23.35157546 9.319377311 28162 12 402 4 0.995024876 0.495031064 1277|1278|1281|1290 COL1A1|COL1A2|COL3A1|COL5A2 -2.880532627 0 25 0 0 -8.278491748 25.47444595

1 -4.733000928 GO:0098643 M1 1 0 GO Cellular Components20 banded collagen fibril -4.733000928 23.35157546 9.319377311 28162 12 402 4 0.995024876 0.495031064 1277|1278|1281|1290 COL1A1|COL1A2|COL3A1|COL5A2 -2.880532627 0 25 0 0 -8.278491748 25.47444595

1 -4.262486313 GO:0001101 M1 1 0 GO Biological Processes 19 response to acid chemical -4.262486313 5.298256616 5.654326949 28162 119 402 9 2.23880597 0.737867616 1277|1278|1282|1290|1906|4067|4313|5105|10891 COL1A1|COL1A2|COL4A1|COL5A2|EDN1|LYN|MMP2|PCK1|PPARGC1A -2.50218464 0 25 0 0 -8.278491748 25.47444595

1 -2.748284387 GO:0071230 M1 1 0 GO Biological Processes 19 cellular response to amino acid stimulus -2.748284387 5.742190686 4.461802836 28162 61 402 5 1.243781095 0.552765814 1277|1278|1282|1290|4313 COL1A1|COL1A2|COL4A1|COL5A2|MMP2 -1.284500518 0 25 0 0 -8.278491748 25.47444595

1 -2.512269787 GO:0071229 M1 1 0 GO Biological Processes 19 cellular response to acid chemical -2.512269787 5.076429447 4.079742455 28162 69 402 5 1.243781095 0.552765814 1277|1278|1282|1290|4313 COL1A1|COL1A2|COL4A1|COL5A2|MMP2 -1.0987556 0 25 0 0 -8.278491748 25.47444595

1 -8.172074945 GO:0005539 M1 1 0 GO Molecular Functions 21 glycosaminoglycan binding -8.172074945 5.482543803 8.21419059 28162 230 402 18 4.47761194 1.031484687 2263|3627|4316|5068|5270|5967|5968|6372|6373|6402|7057|7130|9388|9510|11167|57214|83716|83998 FGFR2|CXCL10|MMP7|REG3A|SERPINE2|REG1A|REG1B|CXCL6|CXCL11|SELL|THBS1|TNFAIP6|LIPG|ADAMTS1|FSTL1|CEMIP|CRISPLD2|REG4 -5.858647633 0 26 1 1 -8.172074945 5.553118554

1 -6.132399993 GO:0008201 M1 1 0 GO Molecular Functions 21 heparin binding -6.132399993 5.553118554 7.037115999 28162 164 402 13 3.233830846 0.882281956 2263|3627|4316|5270|6372|6373|6402|7057|9388|9510|11167|83716|83998 FGFR2|CXCL10|MMP7|SERPINE2|CXCL6|CXCL11|SELL|THBS1|LIPG|ADAMTS1|FSTL1|CRISPLD2|REG4 -4.078853581 0 26 0 0 -8.172074945 5.553118554

1 -5.872157784 GO:1901681 M1 1 0 GO Molecular Functions 21 sulfur compound binding -5.872157784 4.278151229 6.415121123 28162 262 402 16 3.980099502 0.975022334
2263|3627|4316|5270|6372|6373|6402|7057|9388|9510|11167|27010|51363|53354|83716|83998
FGFR2|CXCL10|MMP7|SERPINE2|CXCL6|CXCL11|SELL|THBS1|LIPG|ADAMTS1|FSTL1|TPK1|CHST15|PANK1|CRISPLD2|REG4 -3.844291432 0 26 0 0 -8.172074945 5.553118554
1 -8.169233774 GO:0010876 M1 1 0 GO Biological Processes 19 lipid localization -8.169233774 3.708779631 7.428871068 28162 510 402 27 6.71641791 1.248413442
183|301|366|718|857|1906|2182|2697|3553|4843|5243|5320|5468|6505|6696|7057|7436|9388|23657|26207|54762|84647|114876|123264|124976|130399|200931
AGT|ANXA1|AQP9|C3|CAV1|EDN1|ACSL4|GJA1|IL1B|NOS2|ABCB1|PLA2G2A|PPARG|SLC1A1|SPP1|THBS1|VLDLR|LIPG|SLC7A11|PITPNC1|GRAMD1C|PLA2G12B|OSBPL1A|SLC51B|SPNS2|ACVR1C|SLC51A -5.858647633 0 27 1 1 -8.169233774 5.1373466
1 -7.809691122 GO:0006869 M1 1 0 GO Biological Processes 19 lipid transport -7.809691122 3.799063252 7.291934914 28162 461 402 25 6.218905473 1.204485566
183|301|366|857|1906|2182|2697|3553|4843|5243|5320|5468|6505|6696|7057|7436|9388|23657|26207|54762|84647|114876|123264|124976|200931
AGT|ANXA1|AQP9|CAV1|EDN1|ACSL4|GJA1|IL1B|NOS2|ABCB1|PLA2G2A|PPARG|SLC1A1|SPP1|THBS1|VLDLR|LIPG|SLC7A11|PITPNC1|GRAMD1C|PLA2G12B|OSBPL1A|SLC51B|SPNS2|SLC51A -5.537225445 0 27 0 0 -8.169233774 5.1373466
1 -4.956150359 GO:0015908 M1 1 0 GO Biological Processes 19 fatty acid transport -4.956150359 5.1373466 6.113963018 28162 150 402 11 2.736318408 0.813665012 301|1906|2182|2697|3553|5320|5468|6505|7057|23657|84647
ANXA1|EDN1|ACSL4|GJA1|IL1B|PLA2G2A|PPARG|SLC1A1|THBS1|SLC7A11|PLA2G12B -3.066019617 0 27 0 0 -8.169233774 5.1373466
1 -3.252903389 GO:0005319 M1 1 0 GO Molecular Functions 21 lipid transporter activity -3.252903389 3.891929243 4.44215273 28162 162 402 9 2.23880597 0.737867616 5243|6505|23657|26207|54762|114876|123264|124976|200931
ABCB1|SLC1A1|SLC7A11|PITPNC1|GRAMD1C|OSBPL1A|SLC51B|SPNS2|SLC51A -1.681591934 0 27 0 0 -8.169233774 5.1373466
1 -7.878704645 GO:0042493 M1 1 0 GO Biological Processes 19 response to drug -7.878704645 4.293047298 7.556604541 28162 359 402 22 5.472636816 1.134394361
301|952|1001|1277|1503|1555|1906|3553|4067|4837|4843|5243|5468|6338|6505|6999|7057|7098|10451|10891|10974|29785
ANXA1|CD38|CDH3|COL1A1|CTPS1|CYP2B6|EDN1|IL1B|LYN|NNMT|NOS2|ABCB1|PPARG|SCNN1B|SLC1A1|TDO2|THBS1|TLR3|VAV3|PPARGC1A|ADIRF|CYP2S1 -5.602574014 0 28 1 1 -7.878704645 4.293047298
1 -4.345667645 GO:0070482 M1 1 0 GO Biological Processes 19 response to oxygen levels -4.345667645 3.09332558 4.976818558 28162 385 402 17 4.228855721 1.003727321

360|834|857|952|1277|1906|4017|4313|4843|5105|5328|5468|5967|6505|7057|10891|28984

AQP3|CASP1|CAV1|CD38|COL1A1|EDN1|LOXL2|MMP2|NOS2|PCK1|PLAU|PPARG|REG1A|SLC1A1|THBS1|PPARGC1A|RGCC -2.564092561 0 28 0 0 -7.878704645 4.293047298

1 -3.629490254 GO:0036293 M1 1 0 GO Biological Processes 19 response to decreased oxygen levels -3.629490254 2.918946932 4.40964398 28162 360 402 15 3.731343284 0.945283402

360|834|857|952|1906|4017|4313|4843|5105|5328|5967|6505|7057|10891|28984

AQP3|CASP1|CAV1|CD38|EDN1|LOXL2|MMP2|NOS2|PCK1|PLAU|REG1A|SLC1A1|THBS1|PPARGC1A|RGCC -1.984768994 0 28 0 0 -7.878704645 4.293047298

1 -3.274151596 GO:0001666 M1 1 0 GO Biological Processes 19 response to hypoxia -3.274151596 2.81829359 4.107232013 28162 348 402 14 3.482587065 0.914409651

360|834|857|952|1906|4017|4313|4843|5105|5328|5967|7057|10891|28984

AQP3|CASP1|CAV1|CD38|EDN1|LOXL2|MMP2|NOS2|PCK1|PLAU|REG1A|THBS1|PPARGC1A|RGCC -1.696990969 0 28 0 0 -7.878704645 4.293047298

1 -7.747119504 GO:0071674 M1 1 0 GO Biological Processes 19 mononuclear cell migration -7.747119504 5.868458753 8.124216979 28162 191 402 16 3.980099502 0.975022334

240|301|3579|3627|4067|4478|5175|6283|6356|6362|6373|7057|7474|11240|26585|124976

ALOX5|ANXA1|CXCR2|CXCL10|LYN|MSN|PECAM1|S100A12|CCL11|CCL18|CXCL11|THBS1|WNT5A|PADI2|GREM1|SPNS2 -5.482519097 0 29 1 1 -7.747119504 8.083237658

1 -5.034738375 GO:0002685 M1 1 0 GO Biological Processes 19 regulation of leukocyte migration -5.034738375 4.420929334 5.930175269 28162 206 402 13 3.233830846 0.882281956

301|1906|1908|3627|4067|4478|7057|7474|10562|11240|26585|83483|90865

ANXA1|EDN1|EDN3|CXCL10|LYN|MSN|THBS1|WNT5A|OLFM4|PADI2|GREM1|PLVAP|IL33 -3.129205505 0 29 0 0 -7.747119504 8.083237658

1 -4.238368007 GO:0002548 M1 1 0 GO Biological Processes 19 monocyte chemotaxis -4.238368007 7.107001226 6.111964525 28162 69 402 7 1.741293532 0.652391792 301|3627|4067|6283|6356|6362|26585

ANXA1|CXCL10|LYN|S100A12|CCL11|CCL18|GREM1 -2.481419976 0 29 0 0 -7.747119504 8.083237658

1 -3.522726827 GO:0072676 M1 1 0 GO Biological Processes 19 lymphocyte migration -3.522726827 4.749472974 4.911550163 28162 118 402 8 1.990049751 0.696552772 3627|4478|6356|6362|6373|7474|11240|124976

CXCL10|MSN|CCL11|CCL18|CXCL11|WNT5A|PADI2|SPNS2 -1.90062582 0 29 0 0 -7.747119504 8.083237658

1 -3.517207964 GO:0048247 M1 1 0 GO Biological Processes 19 lymphocyte chemotaxis -3.517207964 6.567630597 5.365992084 28162 64 402 6 1.492537313 0.604761504 3627|6356|6362|6373|7474|11240

CXCL10|CCL11|CCL18|CXCL11|WNT5A|PADI2 -1.896453039 0 29 0 0

-7.747119504 8.083237658
1 -3.497851435 GO:0002688 M1 1 0 GO Biological Processes 19 regulation of leukocyte chemotaxis -3.497851435 4.709561437 4.879902498 28162 119 402 8 1.990049751 0.696552772 1906|1908|3627|4067|7057|7474|11240|26585 EDN1|EDN3|CXCL10|LYN|THBS1|WNT5A|PADI2|GREM1 -1.882719663 0 29 0 0 -7.747119504 8.083237658
1 -2.962158995 GO:0071675 M1 1 0 GO Biological Processes 19 regulation of mononuclear cell migration -2.962158995 4.417865627 4.341798174 28162 111 402 7 1.741293532 0.652391792 3627|4067|4478|7057|7474|11240|26585 CXCL10|LYN|MSN|THBS1|WNT5A|PADI2|GREM1 -1.447293468 0 29 0 0 -7.747119504 8.083237658
1 -2.65563091 GO:0050922 M1 1 0 GO Biological Processes 19 negative regulation of chemotaxis -2.65563091 5.473025498 4.31102944 28162 64 402 5 1.243781095 0.552765814 6091|7057|7474|11240|26585 ROBO1|THBS1|WNT5A|PADI2|GREM1 -1.210708258 0 29 0 0 -7.747119504 8.083237658
1 -2.291377407 GO:0050920 M1 1 0 GO Biological Processes 19 regulation of chemotaxis -2.291377407 2.814698827 3.281392969 28162 224 402 9 2.23880597 0.737867616 1906|1908|3627|4067|6091|7057|7474|11240|26585 EDN1|EDN3|CXCL10|LYN|ROBO1|THBS1|WNT5A|PADI2|GREM1 -0.929240126 0 29 0 0 -7.747119504 8.083237658
1 -2.230434181 GO:1901623 M1 1 0 GO Biological Processes 19 regulation of lymphocyte chemotaxis -2.230434181 8.083237658 4.348251364 28162 26 402 3 0.746268657 0.429247719 3627|7474|11240 CXCL10|WNT5A|PADI2 -0.882189587 0 29 0 0 -7.747119504 8.083237658
1 -2.183845757 GO:0010818 M1 1 0 GO Biological Processes 19 T cell chemotaxis -2.183845757 7.783858485 4.243874515 28162 27 402 3 0.746268657 0.429247719 3627|6373|7474 CXCL10|CXCL11|WNT5A -0.847370775 0 29 0 0 -7.747119504 8.083237658
1 -2.183845757 GO:0090025 M1 1 0 GO Biological Processes 19 regulation of monocyte chemotaxis -2.183845757 7.783858485 4.243874515 28162 27 402 3 0.746268657 0.429247719 3627|4067|26585 CXCL10|LYN|GREM1 -0.847370775 0 29 0 0 -7.747119504 8.083237658
1 -2.096321068 GO:0043032 M1 1 0 GO Biological Processes 19 positive regulation of macrophage activation -2.096321068 7.247040659 4.050350813 28162 29 402 3 0.746268657 0.429247719 7057|7474|90865 THBS1|WNT5A|IL33 -0.778908152 0 29 0 0 -7.747119504 8.083237658
1 -2.005510056 GO:0002690 M1 1 0 GO Biological Processes 19 positive regulation of leukocyte chemotaxis -2.005510056 3.849160789 3.2759408 28162 91 402 5 1.243781095 0.552765814 1906|1908|3627|7057|7474 EDN1|EDN3|CXCL10|THBS1|WNT5A -0.705161612 0 29 0 0 -7.747119504 8.083237658
1 -7.744171905 GO:0003018 M1 1 0 GO Biological Processes 19 vascular process in circulatory system -7.744171905 5.146877856 7.845077612 28162 245 402 18

4.47761194 1.031484687
183|857|952|1906|1908|2697|5028|5243|6338|6505|6533|6566|6584|8140|8671|9429|10979|145741
AGT|CAV1|CD38|EDN1|EDN3|GJA1|P2RY1|ABCB1|SCNN1B|SLC1A1|SLC6A6|SLC16A1|SLC22A5|SLC7A5|SLC4A4|ABCG2|FERMT2|C2CD4A -5.482519097 0 30 1 1
-7.744171905 31.13543394
1 -7.118005613 GO:0003013 M1 1 0 GO Biological Processes 19 circulatory system process -7.118005613 3.210363892 6.647265589 28162 611 402 28 6.965174129
1.269625794
183|290|857|952|1278|1906|1908|2697|3627|3752|4843|5028|5243|5468|6338|6505|6533|6566|6584|7068|8140|8671|9429|10008|10979|29953|55151|145741
AGT|ANPEP|CAV1|CD38|COL1A2|EDN1|EDN3|GJA1|CXCL10|KCND3|NOS2|P2RY1|ABCB1|PPARG|SCNN1B|SLC1A1|SLC6A6|SLC16A1|SLC22A5|THRB|SLC7A5|SLC4A4|ABCG2|KCNE3|FERMT2|TRHDE|TMEM38B|C2CD4A -4.937310309 0 30 0 0 -7.744171905
31.13543394
1 -5.31245939 GO:0014824 M1 1 0 GO Biological Processes 19 artery smooth muscle contraction -5.31245939 31.13543394 10.88087187 28162 9 402 4
0.995024876 0.495031064 952|1906|1908|6338 CD38|EDN1|EDN3|SCNN1B
-3.373820868 0 30 0 0 -7.744171905 31.13543394
1 -4.904169045 GO:0014820 M1 1 0 GO Biological Processes 19 tonic smooth muscle contraction -4.904169045 25.47444595 9.769889016 28162 11 402 4
0.995024876 0.495031064 952|1906|1908|6338 CD38|EDN1|EDN3|SCNN1B
-3.025978422 0 30 0 0 -7.744171905 31.13543394
1 -4.554178074 GO:0008015 M1 1 0 GO Biological Processes 19 blood circulation -4.554178074 2.786267526 4.986191103 28162 528 402 21 5.223880597
1.109770192
183|290|857|952|1278|1906|1908|2697|3627|3752|4843|5028|5468|6338|6505|7068|10008|10979|29953|55151|145741
AGT|ANPEP|CAV1|CD38|COL1A2|EDN1|EDN3|GJA1|CXCL10|KCND3|NOS2|P2RY1|PPARG|SCNN1B|SLC1A1|THRB|KCNE3|FERMT2|TRHDE|TMEM38B|C2CD4A -2.738597074 0 30
0 0 -7.744171905 31.13543394
1 -3.862128923 GO:0042310 M1 1 0 GO Biological Processes 19 vasoconstriction -3.862128923 6.207380817 5.577485714 28162 79 402 7 1.741293532
0.652391792 183|857|952|1906|1908|2697|6338
AGT|CAV1|CD38|EDN1|EDN3|GJA1|SCNN1B -2.174981763 0 30 0 0
-7.744171905 31.13543394
1 -3.63204159 GO:0019229 M1 1 0 GO Biological Processes 19 regulation of vasoconstriction -3.63204159 6.890628823 5.542338157 28162 61 402 6
1.492537313 0.604761504 183|857|952|1906|1908|2697
AGT|CAV1|CD38|EDN1|EDN3|GJA1 -1.986459488 0 30 0 0 -7.744171905
31.13543394
1 -3.460135697 GO:0014829 M1 1 0 GO Biological Processes 19 vascular associated smooth muscle contraction -3.460135697 11.67578773 6.29631243 28162
24 402 4 0.995024876 0.495031064 952|1906|1908|6338

CD38|EDN1|EDN3|SCNN1B -1.854062137 0 30 0 0 -7.744171905 31.13543394
1 -3.307841302 GO:0035296 M1 1 0 GO Biological Processes 19 regulation of tube diameter -3.307841302 4.412896149 4.638760876 28162 127 402 8 1.990049751 0.696552772 183|857|952|1906|1908|2697|5028|6338
AGT|CAV1|CD38|EDN1|EDN3|GJA1|P2RY1|SCNN1B -1.72624151 0 30 0 0 -7.744171905 31.13543394
1 -3.307841302 GO:0097746 M1 1 0 GO Biological Processes 19 blood vessel diameter maintenance -3.307841302 4.412896149 4.638760876 28162 127 402 8 1.990049751 0.696552772 183|857|952|1906|1908|2697|5028|6338
AGT|CAV1|CD38|EDN1|EDN3|GJA1|P2RY1|SCNN1B -1.72624151 0 30 0 0 -7.744171905 31.13543394
1 -3.285152804 GO:0035150 M1 1 0 GO Biological Processes 19 regulation of tube size -3.285152804 4.378420398 4.610027041 28162 128 402 8 1.990049751 0.696552772 183|857|952|1906|1908|2697|5028|6338
AGT|CAV1|CD38|EDN1|EDN3|GJA1|P2RY1|SCNN1B -1.70725546 0 30 0 0 -7.744171905 31.13543394
1 -3.080094246 GO:0090066 M1 1 0 GO Biological Processes 19 regulation of anatomical structure size -3.080094246 2.410790179 3.806837603 28162 494 402 17 4.228855721 1.003727321
183|857|952|1906|1908|2697|3575|4478|5028|6338|6356|6696|7474|10451|53841|85477|282679
AGT|CAV1|CD38|EDN1|EDN3|GJA1|IL7R|MSN|P2RY1|SCNN1B|CCL11|SPP1|WNT5A|VAV3|CDHR5|SCIN|AQP11 -1.537522191 0 30 0 0 -7.744171905 31.13543394
1 -3.048749247 GO:0003012 M1 1 0 GO Biological Processes 19 muscle system process -3.048749247 2.474339121 3.806829238 28162 453 402 16 3.980099502 0.975022334
183|857|952|1134|1906|1908|2697|3488|3553|3752|5028|6338|10008|10891|55151|60675
AGT|CAV1|CD38|CHRNA1|EDN1|EDN3|GJA1|IGFBP5|IL1B|KCND3|P2RY1|SCNN1B|KCNE3|PPARGC1A|TMEM38B|PROK2 -1.514984205 0 30 0 0 -7.744171905 31.13543394
1 -2.979087124 GO:0008217 M1 1 0 GO Biological Processes 19 regulation of blood pressure -2.979087124 3.562104731 4.114794852 28162 177 402 9 2.23880597 0.737867616 183|290|1278|1906|1908|2697|4843|5468|29953
AGT|ANPEP|COL1A2|EDN1|EDN3|GJA1|NOS2|PPARG|TRHDE -1.46294426 0 30 0 0 -7.744171905 31.13543394
1 -2.915486499 GO:0051353 M1 1 0 GO Biological Processes 19 positive regulation of oxidoreductase activity -2.915486499 6.254886283 4.736801076 28162 56 402 5 1.243781095 0.552765814 183|1001|1906|1908|3553
AGT|CDH3|EDN1|EDN3|IL1B -1.413810972 0 30 0 0 -7.744171905 31.13543394
1 -2.880660695 GO:0051785 M1 1 0 GO Biological Processes 19 positive regulation of nuclear division -2.880660695 6.145151436 4.679194811 28162 57 402 5 1.243781095 0.552765814 1906|1908|3553|7474|28984
EDN1|EDN3|IL1B|WNT5A|RGCC -1.383294291 0 30 0 0 -7.744171905

31.13543394
1 -2.567910806 GO:0002027 M1 1 0 GO Biological Processes 19 regulation of heart rate -2.567910806 4.333282043 3.957334293 28162 97 402 6
1.492537313 0.604761504 857|1906|1908|3752|6505|10008
CAV1|EDN1|EDN3|KCND3|SLC1A1|KCNE3 -1.141064218 0 30 0 0
-7.744171905 31.13543394
1 -2.524955472 GO:0008016 M1 1 0 GO Biological Processes 19 regulation of heart contraction -2.524955472 2.847753104 3.502774918 28162 246 402 10
2.487562189 0.776790586 183|857|1906|1908|2697|3752|6505|7068|10008|55151
AGT|CAV1|EDN1|EDN3|GJA1|KCND3|SLC1A1|THRB|KCNE3|TMEM38B -1.108908949 0
30 0 0 -7.744171905 31.13543394
1 -2.501880095 GO:0006939 M1 1 0 GO Biological Processes 19 smooth muscle contraction -2.501880095 4.203283582 3.861565534 28162 100 402 6
1.492537313 0.604761504 857|952|1906|1908|6338|60675
CAV1|CD38|EDN1|EDN3|SCNN1B|PROK2 -1.089374722 0 30 0 0
-7.744171905 31.13543394
1 -2.487719498 GO:0045840 M1 1 0 GO Biological Processes 19 positive regulation of mitotic nuclear division -2.487719498 6.516718732 4.356547625 28162 43 402
4 0.995024876 0.495031064 1906|1908|3553|28984EDN1|EDN3|IL1B|RGCC
-1.080223345 0 30 0 0 -7.744171905 31.13543394
1 -2.482010158 GO:1903522 M1 1 0 GO Biological Processes 19 regulation of blood circulation -2.482010158 2.648116804 3.400834818 28162 291 402 11
2.736318408 0.813665012
183|857|952|1906|1908|2697|3752|6505|7068|10008|55151
AGT|CAV1|CD38|EDN1|EDN3|GJA1|KCND3|SLC1A1|THRB|KCNE3|TMEM38B
-1.076893306 0 30 0 0 -7.744171905 31.13543394
1 -2.298726562 GO:0051341 M1 1 0 GO Biological Processes 19 regulation of oxidoreductase activity -2.298726562 3.821166893 3.567553857 28162 110 402 6
1.492537313 0.604761504 183|857|1001|1906|1908|3553
AGT|CAV1|CDH3|EDN1|EDN3|IL1B -0.933888995 0 30 0 0 -7.744171905
31.13543394
1 -2.283592463 GO:0050886 M1 1 0 GO Biological Processes 19 endocrine process
-2.283592463 4.490687588 3.71497703 28162 78 402 5 1.243781095
0.552765814 183|1906|1908|2697|3553 AGT|EDN1|EDN3|GJA1|IL1B -0.923653081 0
30 0 0 -7.744171905 31.13543394
1 -2.269102711 GO:0044057 M1 1 0 GO Biological Processes 19 regulation of system process -2.269102711 2.011706669 2.993811244 28162 592 402 17
4.228855721 1.003727321
183|857|952|1906|1908|2697|3488|3752|4885|6505|6584|7032|7068|10008|10891|55151|60675
AGT|CAV1|CD38|EDN1|EDN3|GJA1|IGFBP5|KCND3|NPTX2|SLC1A1|SLC22A5|TFF2|THRB|KCNE3|PPARGC1A|TMEM38B|PROK2 -0.914966987 0 30 0 0 -7.744171905
31.13543394
1 -2.230434181 GO:0010460 M1 1 0 GO Biological Processes 19 positive regulation

of heart rate	-2.230434181 8.083237658 4.348251364 28162 26 402 3
0.746268657 0.429247719 1906|1908|6505 EDN1|EDN3|SLC1A1 -0.882189587 0
30 0 0 -7.744171905 31.13543394
1 -2.135881987 GO:0060047 M1 1 0 GO Biological Processes 19 heart contraction
-2.135881987 2.501954513 3.039493132 28162 280 402 10 2.487562189
0.776790586 183|857|1906|1908|2697|3752|6505|7068|10008|55151
AGT|CAV1|EDN1|EDN3|GJA1|KCND3|SLC1A1|THRB|KCNE3|TMEM38B -0.80781603 0
30 0 0 -7.744171905 31.13543394
1 -2.034676125 GO:0003015 M1 1 0 GO Biological Processes 19 heart process
-2.034676125 2.41568022 2.916133676 28162 290 402 10 2.487562189
0.776790586 183|857|1906|1908|2697|3752|6505|7068|10008|55151
AGT|CAV1|EDN1|EDN3|GJA1|KCND3|SLC1A1|THRB|KCNE3|TMEM38B -0.72963256 0
30 0 0 -7.744171905 31.13543394
1 -7.737687274 GO:0005773 M1 1 0 GO Cellular Components20 vacuole
-7.737687274 2.981052186 6.836200354 28162 799 402 34 8.457711443
1.387791708
12|288|290|301|718|1513|2040|2357|2697|3108|3373|3553|3579|4332|4688|6519|7056|7098|7436|8140|8876|10050|10410|10562|10970|11240|23650|27074|27163|55332|79888|81671|206358|255231
SERPINA3|ANK3|ANPEP|ANXA1|C3|CTSK|STOM|FPR1|GJA1|HLA-DMA|HYAL1|IL1B|CXCR2|MNDA|NCF2|SLC3A1|THBD|TLR3|VLDLR|SLC7A5|VNN1|SLC17A4|IFITM3|OLFM4|CKAP4|PADI2|TRIM29|LAMP3|NAAA|DRAM1|LPCAT1|VMP1|SLC36A1|MCOLN2 -5.479579747 0 31
1 1 -7.737687274 5.936841218
1 -7.416927715 GO:0000323 M1 1 0 GO Cellular Components20 lytic vacuole
-7.416927715 3.080420592 6.732036971 28162 705 402 31 7.711442786
1.330542411
12|288|290|301|718|1513|2040|2357|2697|3108|3373|3553|3579|4332|4688|7098|7436|8140|8876|10050|10410|10562|10970|11240|23650|27074|27163|55332|79888|206358|255231
SERPINA3|ANK3|ANPEP|ANXA1|C3|CTSK|STOM|FPR1|GJA1|HLA-DMA|HYAL1|IL1B|CXCR2|MNDA|NCF2|TLR3|VLDLR|SLC7A5|VNN1|SLC17A4|IFITM3|OLFM4|CKAP4|PADI2|TRIM29|LAMP3|NAAA|DRAM1|LPCAT1|SLC36A1|MCOLN2 -5.211929877 0 31 0 0
-7.737687274 5.936841218
1 -7.416927715 GO:0005764 M1 1 0 GO Cellular Components20 lysosome
-7.416927715 3.080420592 6.732036971 28162 705 402 31 7.711442786
1.330542411
12|288|290|301|718|1513|2040|2357|2697|3108|3373|3553|3579|4332|4688|7098|7436|8140|8876|10050|10410|10562|10970|11240|23650|27074|27163|55332|79888|206358|255231
SERPINA3|ANK3|ANPEP|ANXA1|C3|CTSK|STOM|FPR1|GJA1|HLA-DMA|HYAL1|IL1B|CXCR2|MNDA|NCF2|TLR3|VLDLR|SLC7A5|VNN1|SLC17A4|IFITM3|OLFM4|CKAP4|PADI2|TRIM29|LAMP3|NAAA|DRAM1|LPCAT1|SLC36A1|MCOLN2 -5.211929877 0 31 0 0
-7.737687274 5.936841218
1 -4.215282279 GO:0005774 M1 1 0 GO Cellular Components20 vacuolar

membrane -4.215282279 2.905495564 4.814163835 28162 434 402 18
4.47761194 1.031484687
290|2040|2357|3108|6519|7056|7098|7436|8140|8876|10410|10970|27074|55332|79888|81671|206358|255231
ANPEP|STOM|FPR1|HLA-DMA|SLC3A1|THBD|TLR3|VLDLR|SLC7A5|VNN1|IFITM3|CKAP4|LAMP3|DRAM1|LPCAT1|VMP1|SLC36A1|MCOLN2 -2.463866736 0 31 0 0
-7.737687274 5.936841218
1 -4.038725804 GO:0005766 M1 1 0 GO Cellular Components20 primary lysosome
-4.038725804 4.462084482 5.234763083 28162 157 402 10 2.487562189
0.776790586 12|718|2040|2357|4332|8876|10562|10970|11240|79888
SERPINA3|C3|STOM|FPR1|MNDA|VNN1|OLFM4|CKAP4|PADI2|LPCAT1 -2.317053533 0
31 0 0 -7.737687274 5.936841218
1 -4.038725804 GO:0042582 M1 1 0 GO Cellular Components20 azurophil granule
-4.038725804 4.462084482 5.234763083 28162 157 402 10 2.487562189
0.776790586 12|718|2040|2357|4332|8876|10562|10970|11240|79888
SERPINA3|C3|STOM|FPR1|MNDA|VNN1|OLFM4|CKAP4|PADI2|LPCAT1 -2.317053533 0
31 0 0 -7.737687274 5.936841218
1 -3.369819015 GO:0005765 M1 1 0 GO Cellular Components20 lysosomal
membrane -3.369819015 2.758060093 4.157666004 28162 381 402 15
3.731343284 0.945283402
290|2040|2357|3108|7098|7436|8140|8876|10410|10970|27074|55332|79888|206358|255231
ANPEP|STOM|FPR1|HLA-DMA|TLR3|VLDLR|SLC7A5|VNN1|IFITM3|CKAP4|LAMP3|DRAM1|LPCAT1|SLC36A1|MCOLN2-1.775389435 0 31 0 0 -7.737687274 5.936841218
1 -3.369819015 GO:0098852 M1 1 0 GO Cellular Components20 lytic vacuole
membrane -3.369819015 2.758060093 4.157666004 28162 381 402 15
3.731343284 0.945283402
290|2040|2357|3108|7098|7436|8140|8876|10410|10970|27074|55332|79888|206358|255231
ANPEP|STOM|FPR1|HLA-DMA|TLR3|VLDLR|SLC7A5|VNN1|IFITM3|CKAP4|LAMP3|DRAM1|LPCAT1|SLC36A1|MCOLN2-1.775389435 0 31 0 0 -7.737687274 5.936841218
1 -2.813136226 GO:0035577 M1 1 0 GO Cellular Components20 azurophil granule
membrane -2.813136226 5.936841218 4.568000633 28162 59 402 5
1.243781095 0.552765814 2040|2357|8876|10970|79888
STOM|FPR1|VNN1|CKAP4|LPCAT1 -1.330819648 0 31 0 0 -7.737687274
5.936841218
1 -7.632127143 GO:0014074 M1 1 0 GO Biological Processes 19 response to
purine-containing compound -7.632127143 6.763904615 8.37369818 28162 145 402
14 3.482587065 0.914409651
343|366|682|834|1277|3488|5028|5105|5468|6678|7056|10891|50506|55151
AQP8|AQP9|BSG|CASP1|COL1A1|IGFBP5|P2RY1|PCK1|PPARG|SPARC|THBD|PPARGC1A|DUOX2|TMEM38B -5.391324474 0 32 1 1 -7.632127143 6.763904615
1 -5.522706349 GO:0046683 M1 1 0 GO Biological Processes 19 response to
organophosphorus -5.522706349 5.882457939 6.740346064 28162 131 402 11

2.736318408 0.813665012 343|366|682|834|1277|3488|5028|5105|6678|7056|50506 AQP8|AQP9|BSG|CASP1|COL1A1|IGFBP5|P2RY1|PCK1|SPARC|THBD|DUOX2 -3.536421546 0 32 0 0 -7.632127143 6.763904615

1 -5.085411092 GO:0051591 M1 1 0 GO Biological Processes 19 response to cAMP -5.085411092 6.707367418 6.669931235 28162 94 402 9 2.23880597 0.737867616 343|366|682|1277|3488|5105|6678|7056|50506 AQP8|AQP9|BSG|COL1A1|IGFBP5|PCK1|SPARC|THBD|DUOX2 -3.176731147 0 32 0 0 -7.632127143 6.763904615

1 -2.132224823 GO:0071320 M1 1 0 GO Biological Processes 19 cellular response to cAMP -2.132224823 5.18923899 3.70803971 28162 54 402 4 0.995024876 0.495031064 343|366|3488|5105 AQP8|AQP9|IGFBP5|PCK1 -0.805814901 0 32 0 0 -7.632127143 6.763904615

1 -7.472616662 GO:0034330 M1 1 0 GO Biological Processes 19 cell junction organization -7.472616662 3.097997885 6.769135583 28162 701 402 31 7.711442786 1.330542411 183|288|718|857|1001|1009|1134|1282|1942|2555|2697|3371|3553|3915|4038|5175|6678|7057|7474|9073|9076|9912|10979|23657|23705|26585|57126|79983|81671|137075|143098 AGT|ANK3|C3|CAV1|CDH3|CDH11|CHRNA1|COL4A1|EFNA1|GABRA2|GJA1|TNC|IL1B|LAMC1|LRP4|PECAM1|SPARC|THBS1|WNT5A|CLDN8|CLDN1|ARHGAP44|FERMT2|SLC7A11|CADM1|GREM1|CD177|POF1B|VMP1|CLDN23|MPP7 -5.258142932 0 33 1 1 -7.472616662 9.137573005

1 -6.370097594 GO:0045216 M1 1 0 GO Biological Processes 19 cell-cell junction organization -6.370097594 4.980193818 6.983515562 28162 211 402 15 3.731343284 0.945283402 183|857|1001|1009|2697|3553|5175|9073|9076|10979|23705|57126|79983|137075|143098 AGT|CAV1|CDH3|CDH11|GJA1|IL1B|PECAM1|CLDN8|CLDN1|FERMT2|CADM1|CD177|POF1B|CLDN23|MPP7 -4.284536046 0 33 0 0 -7.472616662 9.137573005

1 -5.011511869 GO:0007043 M1 1 0 GO Biological Processes 19 cell-cell junction assembly -5.011511869 5.206770203 6.174750537 28162 148 402 11 2.736318408 0.813665012 183|857|1009|2697|3553|5175|9073|9076|79983|137075|143098 AGT|CAV1|CDH11|GJA1|IL1B|PECAM1|CLDN8|CLDN1|POF1B|CLDN23|MPP7 -3.114087232 0 33 0 0 -7.472616662 9.137573005

1 -4.848988557 GO:0034329 M1 1 0 GO Biological Processes 19 cell junction assembly -4.848988557 3.124506575 5.317020034 28162 426 402 19 4.726368159 1.058368993 183|857|1009|2555|2697|3553|3915|4038|5175|7057|7474|9073|9076|10979|26585|79983|81671|137075|143098 AGT|CAV1|CDH11|GABRA2|GJA1|IL1B|LAMC1|LRP4|PECAM1|THBS1|WNT5A|CLDN8|CLDN1|FERMT2|GREM1|POF1B|VMP1|CLDN23|MPP7 -2.982418547 0 33 0 0 -7.472616662 9.137573005

1 -3.862128923 GO:0070830 M1 1 0 GO Biological Processes 19 bicellular tight junction assembly -3.862128923 6.207380817 5.577485714 28162 79 402 7

1.741293532 0.652391792 2697|5175|9073|9076|79983|137075|143098
GJA1|PECAM1|CLDN8|CLDN1|POF1B|CLDN23|MPP7 -2.174981763 0 33 0 0
-7.472616662 9.137573005
1 -3.760232575 GO:0120192 M1 1 0 GO Biological Processes 19 tight junction assembly -3.760232575 5.980281519 5.43487549 28162 82 402 7 1.741293532
0.652391792 2697|5175|9073|9076|79983|137075|143098
GJA1|PECAM1|CLDN8|CLDN1|POF1B|CLDN23|MPP7 -2.090738316 0 33 0 0
-7.472616662 9.137573005
1 -3.662722038 GO:0120193 M1 1 0 GO Biological Processes 19 tight junction organization -3.662722038 5.76921276 5.299173525 28162 85 402 7
1.741293532 0.652391792 2697|5175|9073|9076|79983|137075|143098
GJA1|PECAM1|CLDN8|CLDN1|POF1B|CLDN23|MPP7 -2.008436349 0 33 0 0
-7.472616662 9.137573005
1 -3.599993817 GO:0043297 M1 1 0 GO Biological Processes 19 apical junction assembly -3.599993817 5.636587179 5.212253214 28162 87 402 7 1.741293532
0.652391792 2697|5175|9073|9076|79983|137075|143098
GJA1|PECAM1|CLDN8|CLDN1|POF1B|CLDN23|MPP7 -1.962957949 0 33 0 0
-7.472616662 9.137573005
1 -2.684771263 GO:0070160 M1 1 0 GO Cellular Components20 tight junction
-2.684771263 3.954702295 3.968061443 28162 124 402 7 1.741293532
0.652391792 288|2697|9073|9076|79983|137075|143098
ANK3|GJA1|CLDN8|CLDN1|POF1B|CLDN23|MPP7 -1.237125753 0 33 0 0
-7.472616662 9.137573005
1 -2.383476348 GO:0016338 M1 1 0 GO Biological Processes 19
calcium-independent cell-cell adhesion via plasma membrane cell-adhesion molecules
-2.383476348 9.137573005 4.698203434 28162 23 402 3 0.746268657
0.429247719 9073|9076|137075 CLDN8|CLDN1|CLDN23 -1.001605443 0 33 0
0 -7.472616662 9.137573005
1 -2.170151338 GO:0005923 M1 1 0 GO Cellular Components20 bicellular tight junction -2.170151338 3.59255007 3.381580211 28162 117 402 6 1.492537313
0.604761504 288|9073|9076|79983|137075|143098
ANK3|CLDN8|CLDN1|POF1B|CLDN23|MPP7 -0.835397365 0 33 0 0
-7.472616662 9.137573005
1 -7.362639184 GO:0001501 M1 1 0 GO Biological Processes 19 skeletal system development -7.362639184 3.603638188 6.967459266 28162 486 402 25
6.218905473 1.204485566
1009|1116|1277|1278|1281|1290|1906|2263|2697|3373|4017|4256|4313|4318|5308|5396|7045|7076|7474|26585|55151|55790|64764|85477|124976
CDH11|CHI3L1|COL1A1|COL1A2|COL3A1|COL5A2|EDN1|FGFR2|GJA1|HYAL1|LOXL2|MGP|MMP2|MMP9|PITX2|PRRX1|TGFBI|TIMP1|WNT5A|GREM1|TMEM38B|CSGALNACT1|CREB3L2|SCIN|SPNS2-5.160754581 0 34 1 1 -7.362639184 5.301438752
1 -6.312297746 GO:0051216 M1 1 0 GO Biological Processes 19 cartilage development -6.312297746 5.301438752 7.063604395 28162 185 402 14
3.482587065 0.914409651

1116|1277|1906|3373|4017|4256|5396|7045|7076|7474|26585|55790|64764|85477
CHI3L1|COL1A1|EDN1|HYAL1|LOXL2|MGP|PRRX1|TGFBI|TIMP1|WNT5A|GREM1|CSGALNACT1|CREB3L2|SCIN -4.233798052 0 34 0 0 -7.362639184 5.301438752
1 -5.555963892 GO:0061448 M1 1 0 GO Biological Processes 19 connective tissue development -5.555963892 4.28906488 6.222264111 28162 245 402 15
3.731343284 0.945283402
1116|1277|1906|3373|4017|4256|5396|7045|7076|7474|10891|26585|55790|64764|85477
CHI3L1|COL1A1|EDN1|HYAL1|LOXL2|MGP|PRRX1|TGFBI|TIMP1|WNT5A|PPARGC1A|GREM1|CSGALNACT1|CREB3L2|SCIN -3.562993844 0 34 0 0 -7.362639184 5.301438752
1 -7.359331692 GO:0005518 M1 1 0 GO Molecular Functions 21 collagen binding -7.359331692 10.30216564 9.241853245 28162 68 402 10 2.487562189
0.776790586 1292|1513|4318|4321|4811|6678|7045|7057|7450|57211
COL6A2|CTSK|MMP9|MMP12|NID1|SPARC|TGFBI|THBS1|VWF|ADGRG6
-5.160538166 0 35 1 1 -7.359331692 10.30216564
1 -4.88964122 GO:0050840 M1 1 0 GO Molecular Functions 21 extracellular matrix binding -4.88964122 8.916056083 7.071479591 28162 55 402 7
1.741293532 0.652391792 1755|4811|6678|6696|7045|7057|57211
DMBT1|NID1|SPARC|SPP1|TGFBI|THBS1|ADGRG6 -3.014385031 0 35 0 0
-7.359331692 10.30216564
1 -2.774916001 GO:0043394 M1 1 0 GO Molecular Functions 21 proteoglycan binding -2.774916001 7.783858485 4.901188162 28162 36 402 4 0.995024876
0.495031064 1513|3371|4811|7057 CTSK|TNC|NID1|THBS1 -1.303326622 0 35 0
0 -7.359331692 10.30216564
1 -2.139188431 GO:0001968 M1 1 0 GO Molecular Functions 21 fibronectin binding -2.139188431 7.505863539 4.144723426 28162 28 402 3 0.746268657
0.429247719 1513|3488|7057 CTSK|IGFBP5|THBS1 -0.810292083 0 35 0 0
-7.359331692 10.30216564
1 -2.096321068 GO:0043236 M1 1 0 GO Molecular Functions 21 laminin binding -2.096321068 7.247040659 4.050350813 28162 29 402 3 0.746268657
0.429247719 4811|7057|57211 NID1|THBS1|ADGRG6 -0.778908152 0 35 0 0
-7.359331692 10.30216564
1 -7.272000685 GO:0061041 M1 1 0 GO Biological Processes 19 regulation of wound healing -7.272000685 6.951995746 8.216867734 28162 131 402 13
3.233830846 0.882281956
240|301|857|1906|2697|5068|5270|5328|7056|7057|9076|10979|50506
ALOX5|ANXA1|CAV1|EDN1|GJA1|REG3A|SERPINE2|PLAU|THBD|THBS1|CLDN1|FERMT2|DUOX2 -5.079324084 0 36 1 1 -7.272000685 9.137573005
1 -6.956279295 GO:1903034 M1 1 0 GO Biological Processes 19 regulation of response to wounding -6.956279295 5.980281519 7.697321813 28162 164 402 14
3.482587065 0.914409651
240|301|857|1906|2697|5068|5270|5328|6696|7056|7057|9076|10979|50506
ALOX5|ANXA1|CAV1|EDN1|GJA1|REG3A|SERPINE2|PLAU|SPP1|THBD|THBS1|CLDN1|FERMT2|DUOX2 -4.787243604 0 36 0 0 -7.272000685 9.137573005

1 -4.685305856 GO:0090303 M1 1 0 GO Biological Processes 19 positive regulation of wound healing -4.685305856 8.311577705 6.765316143 28162 59 402 7 1.741293532 0.652391792 301|5068|7056|7057|9076|10979|50506 ANXA1|REG3A|THBD|THBS1|CLDN1|FERMT2|DUOX2 -2.839720338 0 36 0 0 -7.272000685 9.137573005

1 -4.347441174 GO:1903035 M1 1 0 GO Biological Processes 19 negative regulation of response to wounding -4.347441174 6.227086788 5.976839864 28162 90 402 8 1.990049751 0.696552772 240|1906|2697|5270|5328|6696|7056|7057 ALOX5|EDN1|GJA1|SERPINE2|PLAU|SPP1|THBD|THBS1 -2.5640946 0 36 0 0 -7.272000685 9.137573005

1 -4.158139095 GO:0050818 M1 1 0 GO Biological Processes 19 regulation of coagulation -4.158139095 6.906804008 5.996881503 28162 71 402 7 1.741293532 0.652391792 308|857|1906|5270|5328|7056|7057 ANXA5|CAV1|EDN1|SERPINE2|PLAU|THBD|THBS1 -2.414352509 0 36 0 0 -7.272000685 9.137573005

1 -4.119021843 GO:1903036 M1 1 0 GO Biological Processes 19 positive regulation of response to wounding -4.119021843 6.810876175 5.94099698 28162 72 402 7 1.741293532 0.652391792 301|5068|7056|7057|9076|10979|50506 ANXA1|REG3A|THBD|THBS1|CLDN1|FERMT2|DUOX2 -2.382732513 0 36 0 0 -7.272000685 9.137573005

1 -4.042686588 GO:0061045 M1 1 0 GO Biological Processes 19 negative regulation of wound healing -4.042686588 6.62679844 5.832357572 28162 74 402 7 1.741293532 0.652391792 240|1906|2697|5270|5328|7056|7057 ALOX5|EDN1|GJA1|SERPINE2|PLAU|THBD|THBS1 -2.31792324 0 36 0 0 -7.272000685 9.137573005

1 -4.020950187 GO:0050819 M1 1 0 GO Biological Processes 19 negative regulation of coagulation -4.020950187 8.083237658 6.15219928 28162 52 402 6 1.492537313 0.604761504 308|1906|5270|5328|7056|7057 ANXA5|EDN1|SERPINE2|PLAU|THBD|THBS1 -2.305460374 0 36 0 0 -7.272000685 9.137573005

1 -3.444138847 GO:0030193 M1 1 0 GO Biological Processes 19 regulation of blood coagulation -3.444138847 6.368611488 5.254592347 28162 66 402 6 1.492537313 0.604761504 857|1906|5270|5328|7056|7057 CAV1|EDN1|SERPINE2|PLAU|THBD|THBS1 -1.841981404 0 36 0 0 -7.272000685 9.137573005

1 -3.408584256 GO:1900046 M1 1 0 GO Biological Processes 19 regulation of hemostasis -3.408584256 6.273557585 5.200605462 28162 67 402 6 1.492537313 0.604761504 857|1906|5270|5328|7056|7057 CAV1|EDN1|SERPINE2|PLAU|THBD|THBS1 -1.810201542 0 36 0 0 -7.272000685 9.137573005

1 -3.223516943 GO:0030195 M1 1 0 GO Biological Processes 19 negative regulation of blood coagulation -3.223516943 7.29736733 5.254672543 28162 48 402 5 1.243781095 0.552765814 1906|5270|5328|7056|7057 EDN1|SERPINE2|PLAU|THBD|THBS1 -1.658692996 0 36 0 0 -7.272000685

9.137573005
1 -3.18183375 GO:1900047 M1 1 0 GO Biological Processes 19 negative regulation of hemostasis -3.18183375 7.148441466 5.183663705 28162 49 402 5 1.243781095 0.552765814 1906|5270|5328|7056|7057 EDN1|SERPINE2|PLAU|THBD|THBS1 -1.621281586 0 36 0 0 -7.272000685 9.137573005
1 -2.383476348 GO:0031639 M1 1 0 GO Biological Processes 19 plasminogen activation -2.383476348 9.137573005 4.698203434 28162 23 402 3 0.746268657 0.429247719 5270|5328|7057 SERPINE2|PLAU|THBS1 -1.001605443 0 36 0 0 -7.272000685 9.137573005
1 -2.279112201 GO:0042730 M1 1 0 GO Biological Processes 19 fibrinolysis -2.279112201 8.406567164 4.458362801 28162 25 402 3 0.746268657 0.429247719 5328|7056|7057 PLAU|THBD|THBS1-0.923653081 0 36 0 0 -7.272000685 9.137573005
1 -7.224221028 GO:0031528 M1 1 0 GO Cellular Components20 microvillus membrane -7.224221028 18.86088787 10.96442528 28162 26 402 7 1.741293532 0.652391792 1836|4478|5174|6286|6533|8140|53841 SLC26A2|MSN|PDZK1|S100P|SLC6A6|SLC7A5|CDHR5 -5.037576392 0 37 1 1 -7.224221028 18.86088787
1 -3.479625004 GO:0005902 M1 1 0 GO Cellular Components20 microvillus -3.479625004 5.388825105 5.046233711 28162 91 402 7 1.741293532 0.652391792 1836|4478|5174|6286|6533|8140|53841 SLC26A2|MSN|PDZK1|S100P|SLC6A6|SLC7A5|CDHR5 -1.87118471 0 37 0 0 -7.224221028 18.86088787
1 -7.009811482 GO:0001503 M1 1 0 GO Biological Processes 19 ossification -7.009811482 3.833840747 6.886619185 28162 402 402 22 5.472636816 1.134394361 240|1009|1277|1278|1290|1836|2263|3371|3488|4038|4256|4313|4318|5468|6678|6696|7474|10979|26585|55151|55790|115908 ALOX5|CDH11|COL1A1|COL1A2|COL5A2|SLC26A2|FGFR2|TNC|IGFBP5|LRP4|MGP|MMP2|MMP9|PPARG|SPARC|SPP1|WNT5A|FERMT2|GREM1|TMEM38B|CSGALNACT1|CTHRC1 -4.834985112 0 38 1 1 -7.009811482 5.125955588
1 -4.150466084 GO:0042476 M1 1 0 GO Biological Processes 19 odontogenesis -4.150466084 5.125955588 5.518527057 28162 123 402 9 2.23880597 0.737867616 360|682|1277|1278|1906|2263|3371|4038|5308 AQP3|BSG|COL1A1|COL1A2|EDN1|FGFR2|TNC|LRP4|PITX2 -2.409908469 0 38 0 0 -7.009811482 5.125955588
1 -2.660553226 GO:0031214 M1 1 0 GO Biological Processes 19 biomineral tissue development -2.660553226 3.502736318 3.820391706 28162 160 402 8 1.990049751 0.696552772 240|1277|1278|2263|4256|6696|26585|55151 ALOX5|COL1A1|COL1A2|FGFR2|MGP|SPP1|GREM1|TMEM38B -1.214543479 0 38 0 0 -7.009811482 5.125955588
1 -2.627039124 GO:0110148 M1 1 0 GO Biological Processes 19 biomineralization -2.627039124 3.45949266 3.777907797 28162 162 402 8 1.990049751

0.696552772 240|1277|1278|2263|4256|6696|26585|55151
ALOX5|COL1A1|COL1A2|FGFR2|MGP|SPP1|GREM1|TMEM38B -1.187511505 0 38
0 0 -7.009811482 5.125955588
1 -2.291377407 GO:0001649 M1 1 0 GO Biological Processes 19 osteoblast differentiation -2.291377407 2.814698827 3.281392969 28162 224 402 9
2.23880597 0.737867616 1277|2263|3371|3488|5468|6696|10979|26585|115908
COL1A1|FGFR2|TNC|IGFBP5|PPARG|SPP1|FERMT2|GREM1|CTHRC1 -0.929240126 0
38 0 0 -7.009811482 5.125955588
1 -2.242358017 GO:0030282 M1 1 0 GO Biological Processes 19 bone mineralization -2.242358017 3.719719984 3.486037502 28162 113 402 6
1.492537313 0.604761504 240|1278|2263|4256|26585|55151
ALOX5|COL1A2|FGFR2|MGP|GREM1|TMEM38B-0.891294627 0 38 0 0
-7.009811482 5.125955588
1 -2.170278416 GO:0060348 M1 1 0 GO Biological Processes 19 bone development -2.170278416 2.903822855 3.19369952 28162 193 402 8
1.990049751 0.696552772 1277|2263|2697|5308|26585|55151|55790|124976
COL1A1|FGFR2|GJA1|PITX2|GREM1|TMEM38B|CSGALNACT1|SPNS2 -0.835397365 0
38 0 0 -7.009811482 5.125955588
1 -6.885267506 GO:0048661 M1 1 0 GO Biological Processes 19 positive regulation of smooth muscle cell proliferation -6.885267506 8.027104063 8.299445416 28162
96 402 11 2.736318408 0.813665012
1906|2263|2697|3248|3488|4313|4318|7052|7057|9510|10891
EDN1|FGFR2|GJA1|HPGD|IGFBP5|MMP2|MMP9|TGM2|THBS1|ADAMTS1|PPARGC1A
-4.721946299 0 39 1 1 -6.885267506 10.43368265
1 -5.454883248 GO:0048660 M1 1 0 GO Biological Processes 19 regulation of smooth muscle cell proliferation -5.454883248 5.254104478 6.49383051 28162 160
402 12 2.985074627 0.848757934
1906|2263|2697|3248|3488|4313|4318|5468|7052|7057|9510|10891
EDN1|FGFR2|GJA1|HPGD|IGFBP5|MMP2|MMP9|PPARG|TGM2|THBS1|ADAMTS1|PPARGC1A -3.487081851 0 39 0 0 -6.885267506 10.43368265
1 -5.398907413 GO:0048659 M1 1 0 GO Biological Processes 19 smooth muscle cell proliferation -5.398907413 5.18923899 6.434887528 28162 162 402 12
2.985074627 0.848757934
1906|2263|2697|3248|3488|4313|4318|5468|7052|7057|9510|10891
EDN1|FGFR2|GJA1|HPGD|IGFBP5|MMP2|MMP9|PPARG|TGM2|THBS1|ADAMTS1|PPARGC1A -3.441829881 0 39 0 0 -6.885267506 10.43368265
1 -5.355806247 GO:1904707 M1 1 0 GO Biological Processes 19 positive regulation of vascular associated smooth muscle cell proliferation -5.355806247 10.43368265
7.789121521 28162 47 402 7 1.741293532 0.652391792
1906|2697|3248|3488|4313|4318|9510
EDN1|GJA1|HPGD|IGFBP5|MMP2|MMP9|ADAMTS1 -3.403992955 0 39 0 0
-6.885267506 10.43368265
1 -4.603219892 GO:1904705 M1 1 0 GO Biological Processes 19 regulation of vascular associated smooth muscle cell proliferation -4.603219892 6.752262782

6.315595077 28162 83 402 8 1.990049751 0.696552772
1906|2697|3248|3488|4313|4318|5468|9510
EDN1|GJA1|HPGD|IGFBP5|MMP2|MMP9|PPARG|ADAMTS1 -2.771081098 0 39 0
0 -6.885267506 10.43368265
1 -4.603219892 GO:1990874 M1 1 0 GO Biological Processes 19 vascular associated smooth muscle cell proliferation -4.603219892 6.752262782 6.315595077
28162 83 402 8 1.990049751 0.696552772
1906|2697|3248|3488|4313|4318|5468|9510
EDN1|GJA1|HPGD|IGFBP5|MMP2|MMP9|PPARG|ADAMTS1 -2.771081098 0 39 0
0 -6.885267506 10.43368265
1 -4.038858258 GO:0033002 M1 1 0 GO Biological Processes 19 muscle cell proliferation -4.038858258 3.786741966 5.016344752 28162 222 402 12
2.985074627 0.848757934
1906|2263|2697|3248|3488|4313|4318|5468|7052|7057|9510|10891
EDN1|FGFR2|GJA1|HPGD|IGFBP5|MMP2|MMP9|PPARG|TGM2|THBS1|ADAMTS1|PPARGC1A -2.317053533 0 39 0 0 -6.885267506 10.43368265
1 -2.480423272 GO:0014812 M1 1 0 GO Biological Processes 19 muscle cell migration -2.480423272 4.161666913 3.830474374 28162 101 402 6 1.492537313
0.604761504 301|3488|5328|6282|9510|10891
ANXA1|IGFBP5|PLAU|S100A11|ADAMTS1|PPARGC1A -1.076893306 0 39 0 0
-6.885267506 10.43368265
1 -2.214503711 GO:0014910 M1 1 0 GO Biological Processes 19 regulation of smooth muscle cell migration -2.214503711 4.324365825 3.605557 28162 81 402 5
1.243781095 0.552765814 3488|5328|6282|9510|10891
IGFBP5|PLAU|S100A11|ADAMTS1|PPARGC1A -0.870382238 0 39 0 0
-6.885267506 10.43368265
1 -2.065039701 GO:0014909 M1 1 0 GO Biological Processes 19 smooth muscle cell migration -2.065039701 3.98038218 3.369678588 28162 88 402 5
1.243781095 0.552765814 3488|5328|6282|9510|10891
IGFBP5|PLAU|S100A11|ADAMTS1|PPARGC1A -0.752056345 0 39 0 0
-6.885267506 10.43368265
1 -6.871225764 GO:1901654 M1 1 0 GO Biological Processes 19 response to ketone -6.871225764 5.444667852 7.456015959 28162 193 402 15 3.731343284
0.945283402
687|857|952|1906|3284|3371|3490|4478|5105|5468|6338|6696|7057|9076|79820
KLF9|CAV1|CD38|EDN1|HSD3B2|TNC|IGFBP7|MSN|PCK1|PPARG|SCNN1B|SPP1|THBS1|CLDN1|CATSPERB -4.710733846 0 40 1 1 -6.871225764 5.837893864
1 -5.201531336 GO:0048545 M1 1 0 GO Biological Processes 19 response to steroid hormone -5.201531336 3.608879843 5.736753263 28162 330 402 17
4.228855721 1.003727321
301|687|857|952|1277|1906|3284|3490|3557|5105|6338|6678|6696|7057|9076|11240|79820
ANXA1|KLF9|CAV1|CD38|COL1A1|EDN1|HSD3B2|IGFBP7|IL1RN|PCK1|SCNN1B|SPARC|SPP1|THBS1|CLDN1|PADI2|CATSPERB -3.2800071 0 40 0 0 -6.871225764

5.837893864
1 -5.182692858 GO:0071407 M1 1 0 GO Biological Processes 19 cellular response to organic cyclic compound -5.182692858 2.887560406 5.419500752 28162 558 402 23 5.721393035 1.158362377 301|343|366|687|857|1277|1906|2769|3371|3488|3553|4478|5028|5105|5468|6338|6566|6696|7098|10891|11240|54762|55151 ANXA1|AQP8|AQP9|KLF9|CAV1|COL1A1|EDN1|GNA15|TNC|IGFBP5|IL1B|MSN|P2RY1|PCK1|PPARG|SCNN1B|SLC16A1|SPP1|TLR3|PPARGC1A|PADI2|GRAMD1C|TMEM38B -3.2644325 0 40 0 0 -6.871225764 5.837893864
1 -4.90169722 GO:0031960 M1 1 0 GO Biological Processes 19 response to corticosteroid -4.90169722 5.069749935 6.054249246 28162 152 402 11 2.736318408 0.813665012 301|687|1277|1906|3284|3490|3557|5105|6338|6678|9076 ANXA1|KLF9|COL1A1|EDN1|HSD3B2|IGFBP7|IL1RN|PCK1|SCNN1B|SPARC|CLDN1 -3.024976293 0 40 0 0 -6.871225764 5.837893864
1 -3.839777698 GO:0051384 M1 1 0 GO Biological Processes 19 response to glucocorticoid -3.839777698 4.670315091 5.144098297 28162 135 402 9 2.23880597 0.737867616 301|687|1906|3284|3490|3557|5105|6678|9076 ANXA1|KLF9|EDN1|HSD3B2|IGFBP7|IL1RN|PCK1|SPARC|CLDN1 -2.15734605 0 40 0 0 -6.871225764 5.837893864
1 -3.450536196 GO:1901655 M1 1 0 GO Biological Processes 19 cellular response to ketone -3.450536196 5.330250919 5.006256553 28162 92 402 7 1.741293532 0.652391792 687|3371|4478|5105|5468|6338|6696 KLF9|TNC|MSN|PCK1|PPARG|SCNN1B|SPP1 -1.846033322 0 40 0 0 -6.871225764 5.837893864
1 -2.780387855 GO:0071384 M1 1 0 GO Biological Processes 19 cellular response to corticosteroid stimulus -2.780387855 5.837893864 4.514302722 28162 60 402 5 1.243781095 0.552765814 301|687|1906|5105|6338 ANXA1|KLF9|EDN1|PCK1|SCNN1B -1.305114044 0 40 0 0 -6.871225764 5.837893864
1 -2.104252425 GO:0071385 M1 1 0 GO Biological Processes 19 cellular response to glucocorticoid stimulus -2.104252425 5.09488919 3.657999013 28162 55 402 4 0.995024876 0.495031064 301|687|1906|5105 ANXA1|KLF9|EDN1|PCK1 -0.782364304 0 40 0 0 -6.871225764 5.837893864
1 -2.044913511 GO:0097306 M1 1 0 GO Biological Processes 19 cellular response to alcohol -2.044913511 3.935658785 3.337978215 28162 89 402 5 1.243781095 0.552765814 687|3371|5468|6338|54762 KLF9|TNC|PPARG|SCNN1B|GRAMD1C -0.738293558 0 40 0 0 -6.871225764 5.837893864
1 -6.757106539 GO:0140115 M1 1 0 GO Biological Processes 19 export across plasma membrane -6.757106539 10.50820896 8.872235475 28162 60 402 9 2.23880597 0.737867616 2697|3752|5174|5243|6584|8671|9429|10008|159371 GJA1|KCND3|PDZK1|ABCB1|SLC22A5|SLC4A4|ABCG2|KCNE3|SLC35G1 -4.615919777 0 41 1 1 -6.757106539 10.50820896

1 -2.314184917 GO:0070839 M1 1 0 GO Biological Processes 19 metal ion export -2.314184917 5.837893864 4.036853294 28162 48 402 4 0.995024876 0.495031064 3752|8671|10008|159371 KCND3|SLC4A4|KCNE3|SLC35G1 -0.947537787 0 41 0 0 -6.757106539 10.50820896

1 -6.613713619 GO:0009612 M1 1 0 GO Biological Processes 19 response to mechanical stimulus -6.613713619 5.202083641 7.212720875 28162 202 402 15 3.731343284 0.945283402 834|1116|1277|1281|1906|2697|3371|3553|3627|4316|5028|5270|5468|7057|7098 CASP1|CHI3L1|COL1A1|COL3A1|EDN1|GJA1|TNC|IL1B|CXCL10|MMP7|P2RY1|SERPINE2|PPARG|THBS1|TLR3 -4.485809069 0 42 1 1 -6.613713619 5.680112949

1 -3.339291551 GO:0071496 M1 1 0 GO Biological Processes 19 cellular response to external stimulus -3.339291551 3.005648326 4.223968491 28162 303 402 13 3.233830846 0.882281956 360|834|1030|1277|2697|3371|3553|4067|4316|5105|5166|5468|7098 AQP3|CASP1|CDKN2B|COL1A1|GJA1|TNC|IL1B|LYN|MMP7|PCK1|PDK4|PPARG|TLR3 -1.750946757 0 42 0 0 -6.613713619 5.680112949

1 -3.176229482 GO:0071260 M1 1 0 GO Biological Processes 19 cellular response to mechanical stimulus -3.176229482 5.680112949 4.851087609 28162 74 402 6 1.492537313 0.604761504 834|1277|2697|3553|4316|7098 CASP1|COL1A1|GJA1|IL1B|MMP7|TLR3 -1.61709196 0 42 0 0 -6.613713619 5.680112949

1 -3.083837017 GO:0043122 M1 1 0 GO Biological Processes 19 regulation of I-kappaB kinase/NF-kappaB signaling -3.083837017 3.158204877 4.074485375 28162 244 402 11 2.736318408 0.813665012 834|2697|3553|6283|6398|7052|7098|7474|10346|11040|79931 CASP1|GJA1|IL1B|S100A12|SECTM1|TGM2|TLR3|WNT5A|TRIM22|PIM2|TNIP3 -1.539281494 0 42 0 0 -6.613713619 5.680112949

1 -2.585877351 GO:0007249 M1 1 0 GO Biological Processes 19 I-kappaB kinase/NF-kappaB signaling -2.585877351 2.73263117 3.518938077 28162 282 402 11 2.736318408 0.813665012 834|2697|3553|6283|6398|7052|7098|7474|10346|11040|79931 CASP1|GJA1|IL1B|S100A12|SECTM1|TGM2|TLR3|WNT5A|TRIM22|PIM2|TNIP3 -1.153264548 0 42 0 0 -6.613713619 5.680112949

1 -2.320007429 GO:0043123 M1 1 0 GO Biological Processes 19 positive regulation of I-kappaB kinase/NF-kappaB signaling -2.320007429 3.079328632 3.386587527 28162 182 402 8 1.990049751 0.696552772 834|2697|6283|6398|7052|7098|10346|11040 CASP1|GJA1|S100A12|SECTM1|TGM2|TLR3|TRIM22|PIM2 -0.950175308 0 42 0 0 -6.613713619 5.680112949

1 -6.510079142 GO:0009615 M1 1 0 GO Biological Processes 19 response to virus -6.510079142 3.881148275 6.630042821 28162 361 402 20 4.975124378 1.084445172 1755|2633|3373|3437|3627|3669|4283|4321|5450|6356|7098|9582|10346|10410|10581|11040|50506|54941|90865|197259

DMBT1|GBP1|HYAL1|IFIT3|CXCL10|ISG20|CXCL9|MMP12|POU2AF1|CCL11|TLR3|APOBEC3B|TRIM22|IFITM3|IFITM2|PIM2|DUOX2|RNF125|IL33|MLKL -4.395062614 0 43 1 1 -6.510079142 13.3437574

1 -5.201508477 GO:0051607 M1 1 0 GO Biological Processes 19 defense response to virus -5.201508477 4.010766777 5.891867763 28162 262 402 15 3.731343284 0.945283402 1755|2633|3437|3627|3669|4283|4321|7098|9582|10346|10410|10581|54941|90865|197259 DMBT1|GBP1|IFIT3|CXCL10|ISG20|CXCL9|MMP12|TLR3|APOBEC3B|TRIM22|IFITM3|IFITM2|RNF125|IL33|MLKL -3.2800071 0 43 0 0 -6.510079142 13.3437574

1 -5.201508477 GO:0140546 M1 1 0 GO Biological Processes 19 defense response to symbiont -5.201508477 4.010766777 5.891867763 28162 262 402 15 3.731343284 0.945283402 1755|2633|3437|3627|3669|4283|4321|7098|9582|10346|10410|10581|54941|90865|197259 DMBT1|GBP1|IFIT3|CXCL10|ISG20|CXCL9|MMP12|TLR3|APOBEC3B|TRIM22|IFITM3|IFITM2|RNF125|IL33|MLKL -3.2800071 0 43 0 0 -6.510079142 13.3437574

1 -3.694747652 GO:0035455 M1 1 0 GO Biological Processes 19 response to interferon-alpha -3.694747652 13.3437574 6.809490119 28162 21 402 4 0.995024876 0.495031064 3437|10410|10581|27074 IFIT3|IFITM3|IFITM2|LAMP3 -2.035154836 0 43 0 0 -6.510079142 13.3437574

1 -3.022880942 GO:0035456 M1 1 0 GO Biological Processes 19 response to interferon-beta -3.022880942 9.039319531 5.389331326 28162 31 402 4 0.995024876 0.495031064 4332|7098|10410|10581 MNDA|TLR3|IFITM3|IFITM2 -1.498342532 0 43 0 0 -6.510079142 13.3437574

1 -2.613374383 GO:0060337 M1 1 0 GO Biological Processes 19 type I interferon signaling pathway -2.613374383 4.424509034 4.023365184 28162 95 402 6 1.492537313 0.604761504 3437|3669|4321|7474|10410|10581 IFIT3|ISG20|MMP12|WNT5A|IFITM3|IFITM2 -1.176519353 0 43 0 0 -6.510079142 13.3437574

1 -2.590494155 GO:0071357 M1 1 0 GO Biological Processes 19 cellular response to type I interferon -2.590494155 4.378420398 3.990123874 28162 96 402 6 1.492537313 0.604761504 3437|3669|4321|7474|10410|10581 IFIT3|ISG20|MMP12|WNT5A|IFITM3|IFITM2 -1.157353334 0 43 0 0 -6.510079142 13.3437574

1 -2.480423272 GO:0034340 M1 1 0 GO Biological Processes 19 response to type I interferon -2.480423272 4.161666913 3.830474374 28162 101 402 6 1.492537313 0.604761504 3437|3669|4321|7474|10410|10581 IFIT3|ISG20|MMP12|WNT5A|IFITM3|IFITM2 -1.076893306 0 43 0 0 -6.510079142 13.3437574

1 -2.314184917 GO:0052372 M1 1 0 GO Biological Processes 19 modulation by symbiont of entry into host -2.314184917 5.837893864 4.036853294 28162 48 402 4 0.995024876 0.495031064 857|10346|10410|10581 CAV1|TRIM22|IFITM3|IFITM2 -0.947537787 0 43 0 0 -6.510079142

13.3437574

1 -2.132224823 GO:0045071 M1 1 0 GO Biological Processes 19 negative regulation of viral genome replication -2.132224823 5.18923899 3.70803971 28162 54 402 4 0.995024876 0.495031064 3669|9582|10410|10581 ISG20|APOBEC3B|IFITM3|IFITM2 -0.805814901 0 43 0 0 -6.510079142 13.3437574

1 -2.118777626 GO:0043903 M1 1 0 GO Biological Processes 19 regulation of biological process involved in symbiotic interaction -2.118777626 2.844861984 3.126921042 28162 197 402 8 1.990049751 0.696552772 857|3669|6372|9582|10346|10410|10581|27074 CAV1|ISG20|CXCL6|APOBEC3B|TRIM22|IFITM3|IFITM2|LAMP3 -0.794428906 0 43 0 0 -6.510079142 13.3437574

1 -6.436526049 GO:0043270 M1 1 0 GO Biological Processes 19 positive regulation of ion transport -6.436526049 2.972018694 6.169190605 28162 660 402 28 6.965174129 1.269625794 288|718|857|952|1906|1908|2040|2182|2697|3553|3627|4283|5028|5174|5243|6279|6373|6696|9388|10008|23255|23433|23596|57214|58480|63928|123264|154043 ANK3|C3|CAV1|CD38|EDN1|EDN3|STOM|ACSL4|GJA1|IL1B|CXCL10|CXCL9|P2RY1|PDZK1|ABCB1|S100A8|CXCL11|SPP1|LIPG|KCNE3|MTCL1|RHOQ|OPN3|CEMIP|RHOU|CHP2|SLC51B|CNKSR3 -4.329062659 0 44 1 1 -6.436526049 14.01094527

1 -6.174175232 GO:0090313 M1 1 0 GO Biological Processes 19 regulation of protein targeting to membrane -6.174175232 13.62175235 9.118937745 28162 36 402 7 1.741293532 0.652391792 288|2040|5174|6505|23255|57214|123264 ANK3|STOM|PDZK1|SLC1A1|MTCL1|CEMIP|SLC51B -4.111719903 0 44 0 0 -6.436526049 14.01094527

1 -5.441134285 GO:0090314 M1 1 0 GO Biological Processes 19 positive regulation of protein targeting to membrane -5.441134285 14.01094527 8.580182099 28162 30 402 6 1.492537313 0.604761504 288|2040|5174|23255|57214|123264 ANK3|STOM|PDZK1|MTCL1|CEMIP|SLC51B-3.478727921 0 44 0 0 -6.436526049 14.01094527

1 -5.317469266 GO:1905475 M1 1 0 GO Biological Processes 19 regulation of protein localization to membrane -5.317469266 4.69438888 6.213554327 28162 194 402 13 3.233830846 0.882281956 288|2040|2633|4038|5174|6505|9912|23255|23433|23657|57214|57628|123264 ANK3|STOM|GBP1|LRP4|PDZK1|SLC1A1|ARHGAP44|MTCL1|RHOQ|SLC7A11|CEMIP|DPP10|SLC51B -3.375996808 0 44 0 0 -6.436526049 14.01094527

1 -5.198049713 GO:1903793 M1 1 0 GO Biological Processes 19 positive regulation of anion transport -5.198049713 3.077718104 5.513323602 28162 478 402 21 5.223880597 1.109770192 288|718|857|952|1906|2040|2182|2697|3553|5174|5243|6279|6696|9388|23255|23433|23596|57214|58480|63928|123264 ANK3|C3|CAV1|CD38|EDN1|STOM|ACSL4|GJA1|IL1B|PDZK1|ABCB1|S100A8|SPP1|LIPG|MTCL1|RHOQ|OPN3|CEMIP|RHOU|CHP2|SLC51B -3.278171868 0 44 0 0 -6.436526049 14.01094527

1 -4.681057712 GO:1903533 M1 1 0 GO Biological Processes 19 regulation of protein targeting -4.681057712 6.91898532 6.419642145 28162 81 402 8 1.990049751 0.696552772 288|2040|5174|6505|23255|57214|58480|123264 ANK3|STOM|PDZK1|SLC1A1|MTCL1|CEMIP|RHOU|SLC51B -2.836835756 0 44 0 0 -6.436526049 14.01094527

1 -4.433744575 GO:0090087 M1 1 0 GO Biological Processes 19 regulation of peptide transport -4.433744575 2.657248242 4.853063999 28162 580 402 22 5.472636816 1.134394361 240|288|301|952|2040|2182|2697|3553|4843|5174|6279|6505|6566|9912|23255|23657|57214|58480|63928|91319|123264|130399 ALOX5|ANK3|ANXA1|CD38|STOM|ACSL4|GJA1|IL1B|NOS2|PDZK1|S100A8|SLC1A1|SLC16A1|ARHGAP44|MTCL1|SLC7A11|CEMIP|RHOU|CHP2|DERL3|SLC51B|ACVR1C -2.641168835 0 44 0 0 -6.436526049 14.01094527

1 -4.321372842 GO:1903827 M1 1 0 GO Biological Processes 19 regulation of cellular protein localization -4.321372842 2.68457893 4.792303511 28162 548 402 21 5.223880597 1.109770192 288|2040|2633|3553|4038|4478|5174|6505|7474|9912|10979|23255|23433|23650|23657|57214|57628|58480|63928|91319|123264 ANK3|STOM|GBP1|IL1B|LRP4|MSN|PDZK1|SLC1A1|WNT5A|ARHGAP44|FERMT2|MTCL1|RHOQ|TRIM29|SLC7A11|CEMIP|DPP10|RHOU|CHP2|DERL3|SLC51B -2.546231806 0 44 0 0 -6.436526049 14.01094527

1 -4.310070432 GO:0051223 M1 1 0 GO Biological Processes 19 regulation of protein transport -4.310070432 2.679688987 4.782837023 28162 549 402 21 5.223880597 1.109770192 240|288|301|952|2040|2182|2697|3553|4843|5174|6505|6566|9912|23255|23657|57214|58480|63928|91319|123264|130399 ALOX5|ANK3|ANXA1|CD38|STOM|ACSL4|GJA1|IL1B|NOS2|PDZK1|SLC1A1|SLC16A1|ARHGAP44|MTCL1|SLC7A11|CEMIP|RHOU|CHP2|DERL3|SLC51B|ACVR1C -2.540697003 0 44 0 0 -6.436526049 14.01094527

1 -4.03738615 GO:0070201 M1 1 0 GO Biological Processes 19 regulation of establishment of protein localization -4.03738615 2.562977794 4.552772793 28162 574 402 21 5.223880597 1.109770192 240|288|301|952|2040|2182|2697|3553|4843|5174|6505|6566|9912|23255|23657|57214|58480|63928|91319|123264|130399 ALOX5|ANK3|ANXA1|CD38|STOM|ACSL4|GJA1|IL1B|NOS2|PDZK1|SLC1A1|SLC16A1|ARHGAP44|MTCL1|SLC7A11|CEMIP|RHOU|CHP2|DERL3|SLC51B|ACVR1C -2.316739369 0 44 0 0 -6.436526049 14.01094527

1 -3.60163366 GO:0072657 M1 1 0 GO Biological Processes 19 protein localization to membrane -3.60163366 2.324591222 4.153435956 28162 663 402 22 5.472636816 1.134394361 288|682|857|2040|2633|4038|5028|5174|6505|9912|10966|10979|11031|23255|23433|23657|55670|57214|57628|81693|123264|282679 ANK3|BSG|CAV1|STOM|GBP1|LRP4|P2RY1|PDZK1|SLC1A1|ARHGAP44|RAB40B|FERMT2|RAB31|MTCL1|RHOQ|SLC7A11|PEX26|CEMIP|DPP10|AMN|SLC51B|AQP11 -1.963738387 0

44 0 0 -6.436526049 14.01094527
1 -3.591006577 GO:0033157 M1 1 0 GO Biological Processes 19 regulation of intracellular protein transport -3.591006577 3.389744824 4.54876672 28162 248 402 12 2.985074627 0.848757934 288|2040|3553|5174|6505|9912|23255|57214|58480|63928|91319|123264 ANK3|STOM|IL1B|PDZK1|SLC1A1|ARHGAP44|MTCL1|CEMIP|RHOU|CHP2|DERL3|SLC51B -1.959831662 0 44 0 0 -6.436526049 14.01094527
1 -3.307841302 GO:1905477 M1 1 0 GO Biological Processes 19 positive regulation of protein localization to membrane -3.307841302 4.412896149 4.638760876 28162 127 402 8 1.990049751 0.696552772 288|2040|4038|5174|23255|57214|57628|123264 ANK3|STOM|LRP4|PDZK1|MTCL1|CEMIP|DPP10|SLC51B -1.72624151 0 44 0 0 -6.436526049 14.01094527
1 -2.985014234 GO:1990778 M1 1 0 GO Biological Processes 19 protein localization to cell periphery -2.985014234 2.751394087 3.857099144 28162 331 402 13 3.233830846 0.882281956 288|682|857|2633|5028|5174|6505|9912|10966|11031|23433|57628|81693 ANK3|BSG|CAV1|GBP1|P2RY1|PDZK1|SLC1A1|ARHGAP44|RAB40B|RAB31|RHOQ|DPP10|AMN -1.467590265 0 44 0 0 -6.436526049 14.01094527
1 -2.94490535 GO:0090316 M1 1 0 GO Biological Processes 19 positive regulation of intracellular protein transport -2.94490535 3.522304678 4.073842705 28162 179 402 9 2.23880597 0.737867616 288|2040|3553|5174|23255|57214|58480|63928|123264 ANK3|STOM|IL1B|PDZK1|MTCL1|CEMIP|RHOU|CHP2|SLC51B -1.432583283 0 44 0 0 -6.436526049 14.01094527
1 -2.925865338 GO:1903829 M1 1 0 GO Biological Processes 19 positive regulation of cellular protein localization -2.925865338 2.849683784 3.843179954 28162 295 402 12 2.985074627 0.848757934 288|2040|4038|4478|5174|10979|23255|57214|57628|58480|63928|123264 ANK3|STOM|LRP4|MSN|PDZK1|FERMT2|MTCL1|CEMIP|DPP10|RHOU|CHP2|SLC51B -1.421085478 0 44 0 0 -6.436526049 14.01094527
1 -2.893941804 GO:0032388 M1 1 0 GO Biological Processes 19 positive regulation of intracellular transport -2.893941804 3.198845953 3.931041951 28162 219 402 10 2.487562189 0.776790586 288|2040|3553|4478|5174|23255|57214|58480|63928|123264 ANK3|STOM|IL1B|MSN|PDZK1|MTCL1|CEMIP|RHOU|CHP2|SLC51B -1.394733867 0 44 0 0 -6.436526049 14.01094527
1 -2.768793447 GO:0032386 M1 1 0 GO Biological Processes 19 regulation of intracellular transport -2.768793447 2.602032694 3.629247769 28162 350 402 13 3.233830846 0.882281956 288|2040|3553|4478|5174|6505|9912|23255|57214|58480|63928|91319|123264 ANK3|STOM|IL1B|MSN|PDZK1|SLC1A1|ARHGAP44|MTCL1|CEMIP|RHOU|CHP2|DERL3|SLC51B -1.301022585 0 44 0 0 -6.436526049 14.01094527
1 -2.721568564 GO:0051222 M1 1 0 GO Biological Processes 19 positive regulation

of protein transport -2.721568564 2.694412553 3.621689294 28162 312 402 12
2.985074627 0.848757934
288|952|2040|2182|2697|3553|5174|23255|57214|58480|63928|123264
ANK3|CD38|STOM|ACSL4|GJA1|IL1B|PDZK1|MTCL1|CEMIP|RHOU|CHP2|SLC51B
-1.262857628 0 44 0 0 -6.436526049 14.01094527
1 -2.64571796 GO:0072659 M1 1 0 GO Biological Processes 19 protein localization to plasma membrane -2.64571796 2.781956643 3.586567634 28162 277 402 11 2.736318408 0.813665012
288|682|2633|5028|5174|9912|10966|11031|23433|57628|81693
ANK3|BSG|GBP1|P2RY1|PDZK1|ARHGAP44|RAB40B|RAB31|RHOQ|DPP10|AMN
-1.20187969 0 44 0 0 -6.436526049 14.01094527
1 -2.543845138 GO:1904951 M1 1 0 GO Biological Processes 19 positive regulation of establishment of protein localization -2.543845138 2.562977794 3.426330484 28162 328 402 12 2.985074627 0.848757934
288|952|2040|2182|2697|3553|5174|23255|57214|58480|63928|123264
ANK3|CD38|STOM|ACSL4|GJA1|IL1B|PDZK1|MTCL1|CEMIP|RHOU|CHP2|SLC51B
-1.123715909 0 44 0 0 -6.436526049 14.01094527
1 -2.460019602 GO:0006612 M1 1 0 GO Biological Processes 19 protein targeting to membrane -2.460019602 2.988116291 3.488282654 28162 211 402 9 2.23880597 0.737867616 288|2040|5174|6505|23255|55670|57214|123264|282679
ANK3|STOM|PDZK1|SLC1A1|MTCL1|PEX26|CEMIP|SLC51B|AQP11 -1.060908724 0 44 0 0 -6.436526049 14.01094527
1 -2.262658658 GO:0090150 M1 1 0 GO Biological Processes 19 establishment of protein localization to membrane -2.262658658 2.361395271 3.110765755 28162 356 402 12 2.985074627 0.848757934
288|2040|5174|6505|9912|11031|23255|55670|57214|81693|123264|282679
ANK3|STOM|PDZK1|SLC1A1|ARHGAP44|RAB31|MTCL1|PEX26|CEMIP|AMN|SLC51B|AQP11 -0.908963172 0 44 0 0 -6.436526049 14.01094527
1 -6.401447898 GO:0009636 M1 1 0 GO Biological Processes 19 response to toxic substance -6.401447898 4.689856159 6.893736522 28162 239 402 16 3.980099502 0.975022334
2053|4067|4499|5174|6280|6584|7837|9076|9429|9963|11254|23657|50506|55532|140803|493869
EPHX2|LYN|MT1M|PDZK1|S100A9|SLC22A5|PXDN|CLDN1|ABCG2|SLC23A1|SLC6A14|SLC7A11|DUOX2|SLC30A10|TRPM6|GPX8-4.307643047 0 45 1 1 -6.401447898 4.689856159
1 -3.767473868 GO:0098754 M1 1 0 GO Biological Processes 19 detoxification -3.767473868 4.568786502 5.057342665 28162 138 402 9 2.23880597 0.737867616 4499|5174|6280|6584|7837|9429|50506|55532|493869
MT1M|PDZK1|S100A9|SLC22A5|PXDN|ABCG2|DUOX2|SLC30A10|GPX8 -2.095239571 0 45 0 0 -6.401447898 4.689856159
1 -6.189449549 GO:0042445 M1 1 0 GO Biological Processes 19 hormone metabolic process -6.189449549 4.820279337 6.814067195 28162 218 402 15 3.731343284 0.945283402

126|1513|2690|3284|3294|5122|6696|10891|25825|29785|50506|79154|79644|195814|405753
ADH1C|CTSK|GHR|HSD3B2|HSD17B2|PCSK1|SPP1|PPARGC1A|BACE2|CYP2S1|DUOX2|DHRS11|SRD5A3|SDR16C5|DUOXA2 -4.122470278 0 46 1 1 -6.189449549 9.340630182
1 -3.815453699 GO:0034754 M1 1 0 GO Biological Processes 19 cellular hormone metabolic process -3.815453699 4.635974539 5.114898979 28162 136 402 9 2.23880597 0.737867616 126|3284|3294|6696|10891|29785|79154|79644|195814 ADH1C|HSD3B2|HSD17B2|SPP1|PPARGC1A|CYP2S1|DHRS11|SRD5A3|SDR16C5 -2.137653874 0 46 0 0 -6.189449549 9.340630182
1 -3.51692442 GO:0008202 M1 1 0 GO Biological Processes 19 steroid metabolic process -3.51692442 2.981052186 4.349518548 28162 329 402 14 3.482587065 0.914409651
183|1555|2053|2330|3158|3284|3294|3490|6696|7436|10891|79154|79644|114876
AGT|CYP2B6|EPHX2|FMO5|HMGCS2|HSD3B2|HSD17B2|IGFBP7|SPP1|VLDLR|PPARGC1A|DHRS11|SRD5A3|OSBPL1A -1.896453039 0 46 0 0 -6.189449549 9.340630182
1 -3.339335068 GO:0042446 M1 1 0 GO Biological Processes 19 hormone biosynthetic process -3.339335068 6.091715336 5.09585349 28162 69 402 6 1.492537313 0.604761504 3284|3294|10891|50506|79154|79644 HSD3B2|HSD17B2|PPARGC1A|DUOX2|DHRS11|SRD5A3 -1.750946757 0 46 0 0 -6.189449549 9.340630182
1 -3.18183375 GO:0120178 M1 1 0 GO Biological Processes 19 steroid hormone biosynthetic process -3.18183375 7.148441466 5.183663705 28162 49 402 5 1.243781095 0.552765814 3284|3294|10891|79154|79644 HSD3B2|HSD17B2|PPARGC1A|DHRS11|SRD5A3 -1.621281586 0 46 0 0 -6.189449549 9.340630182
1 -3.077999801 GO:0008209 M1 1 0 GO Biological Processes 19 androgen metabolic process -3.077999801 9.340630182 5.500301806 28162 30 402 4 0.995024876 0.495031064 3284|6696|10891|79644 HSD3B2|SPP1|PPARGC1A|SRD5A3 -1.537522191 0 46 0 0 -6.189449549 9.340630182
1 -2.774916001 GO:0016229 M1 1 0 GO Molecular Functions 21 steroid dehydrogenase activity -2.774916001 7.783858485 4.901188162 28162 36 402 4 0.995024876 0.495031064 3284|3294|79154|79644 HSD3B2|HSD17B2|DHRS11|SRD5A3 -1.303326622 0 46 0 0 -6.189449549 9.340630182
1 -2.724967491 GO:0016616 M1 1 0 GO Molecular Functions 21 oxidoreductase activity, acting on the CH-OH group of donors, NAD or NADP as acceptor -2.724967491 4.01953348 4.022148417 28162 122 402 7 1.741293532 0.652391792 126|3248|3284|3294|4199|79154|195814 ADH1C|HPGD|HSD3B2|HSD17B2|ME1|DHRS11|SDR16C5 -1.265695813 0 46 0 0 -6.189449549 9.340630182
1 -2.531988857 GO:0016614 M1 1 0 GO Molecular Functions 21 oxidoreductase activity, acting on CH-OH group of donors -2.531988857 3.715023368 3.762498371 28162 132 402 7 1.741293532 0.652391792

126|3248|3284|3294|4199|79154|195814
ADH1C|HPGD|HSD3B2|HSD17B2|ME1|DHRS11|SDR16C5 -1.112883909 0 46 0 0
-6.189449549 9.340630182
1 -2.196598898 GO:0006694 M1 1 0 GO Biological Processes 19 steroid biosynthetic process -2.196598898 2.934229377 3.22773591 28162 191 402 8
1.990049751 0.696552772 3158|3284|3294|3490|10891|79154|79644|114876
HMGCS2|HSD3B2|HSD17B2|IGFBP7|PPARGC1A|DHRS11|SRD5A3|OSBPL1A
-0.854624214 0 46 0 0 -6.189449549 9.340630182
1 -2.055117446 GO:0033764 M1 1 0 GO Molecular Functions 21 steroid dehydrogenase activity, acting on the CH-OH group of donors, NAD or NADP as acceptor
-2.055117446 7.005472637 3.96036166 28162 30 402 3 0.746268657
0.429247719 3284|3294|79154 HSD3B2|HSD17B2|DHRS11 -0.74572497 0 46 0
0 -6.189449549 9.340630182
1 -6.162693985 GO:0030324 M1 1 0 GO Biological Processes 19 lung development
-6.162693985 5.587186766 7.067996023 28162 163 402 13 3.233830846
0.882281956
1116|2263|3371|3488|4321|6678|7068|7474|9963|23657|55151|83716|284217
CHI3L1|FGFR2|TNC|IGFBP5|MMP12|SPARC|THRB|WNT5A|SLC23A1|SLC7A11|TMEM38B|CRISPLD2|LAMA1 -4.102483076 0 47 1 1 -6.162693985 9.217727154
1 -6.042864162 GO:0030323 M1 1 0 GO Biological Processes 19 respiratory tube development -6.042864162 5.453361933 6.945976731 28162 167 402 13
3.233830846 0.882281956
1116|2263|3371|3488|4321|6678|7068|7474|9963|23657|55151|83716|284217
CHI3L1|FGFR2|TNC|IGFBP5|MMP12|SPARC|THRB|WNT5A|SLC23A1|SLC7A11|TMEM38B|CRISPLD2|LAMA1 -4.000202893 0 47 0 0 -6.162693985 9.217727154
1 -5.467281984 GO:0060541 M1 1 0 GO Biological Processes 19 respiratory system development -5.467281984 4.844209802 6.364086195 28162 188 402 13
3.233830846 0.882281956
1116|2263|3371|3488|4321|6678|7068|7474|9963|23657|55151|83716|284217
CHI3L1|FGFR2|TNC|IGFBP5|MMP12|SPARC|THRB|WNT5A|SLC23A1|SLC7A11|TMEM38B|CRISPLD2|LAMA1 -3.495846303 0 47 0 0 -6.162693985 9.217727154
1 -3.705744461 GO:0048286 M1 1 0 GO Biological Processes 19 lung alveolus development -3.705744461 9.217727154 6.100034669 28162 38 402 5
1.243781095 0.552765814 2263|3488|4321|23657|55151
FGFR2|IGFBP5|MMP12|SLC7A11|TMEM38B -2.042577184 0 47 0 0
-6.162693985 9.217727154
1 -6.128980014 GO:1901137 M1 1 0 GO Biological Processes 19 carbohydrate derivative biosynthetic process -6.128980014 2.867737337 5.950844799 28162 684
402 28 6.965174129 1.269625794
957|1503|1836|2182|3373|3553|5166|6241|10891|22822|23333|23753|25825|51363|53354|55790|57214|60481|79644|80157|80235|90865|91319|123264|124872|282679|727897|727936
ENTPD5|CTPS1|SLC26A2|ACSL4|HYAL1|IL1B|PDK4|RRM2|PPARGC1A|PHLDA1|DPY19L1|SDF2L1|BACE2|CHST15|PANK1|CSGALNACT1|CEMIP|ELOVL5|SRD5A3|CWH43|PIGZ|IL33|DERL3

|SLC51B|B4GALNT2|AQP11|MUC5B|GXYLT2 -4.077632566 0 48 1 1
-6.128980014 3.002345416
1 -4.345078833 GO:0009100 M1 1 0 GO Biological Processes 19 glycoprotein metabolic process -4.345078833 2.974021402 4.928611038 28162 424 402 18
4.47761194 1.031484687
957|3373|4321|22822|23333|23753|25825|51363|55790|56999|79644|90865|91319|123264|124872|282679|727897|727936
ENTPD5|HYAL1|MMP12|PHLDA1|DPY19L1|SDF2L1|BACE2|CHST15|CSGALNACT1|ADAMTS9|SRD5A3|IL33|DERL3|SLC51B|B4GALNT2|AQP11|MUC5B|GXYLT2 -2.564092561 0 48
0 0 -6.128980014 3.002345416
1 -3.761042642 GO:0009101 M1 1 0 GO Biological Processes 19 glycoprotein biosynthetic process -3.761042642 3.002345416 4.536116935 28162 350 402 15
3.731343284 0.945283402
957|22822|23333|23753|25825|51363|55790|79644|90865|91319|123264|124872|282679|727897|727936
ENTPD5|PHLDA1|DPY19L1|SDF2L1|BACE2|CHST15|CSGALNACT1|SRD5A3|IL33|DERL3|SLC51B|B4GALNT2|AQP11|MUC5B|GXYLT2 -2.090738316 0 48 0 0 -6.128980014
3.002345416
1 -2.78308189 GO:0070085 M1 1 0 GO Biological Processes 19 glycosylation
-2.78308189 2.896999963 3.740800137 28162 266 402 11 2.736318408
0.813665012
957|23333|23753|79644|80235|91319|123264|124872|282679|727897|727936
ENTPD5|DPY19L1|SDF2L1|SRD5A3|PIGZ|DERL3|SLC51B|B4GALNT2|AQP11|MUC5B|GXYLT2 -1.305114044 0 48 0 0 -6.128980014 3.002345416
1 -2.438606207 GO:0006486 M1 1 0 GO Biological Processes 19 protein glycosylation -2.438606207 2.768961517 3.401213073 28162 253 402 10
2.487562189 0.776790586
957|23333|23753|79644|91319|123264|124872|282679|727897|727936
ENTPD5|DPY19L1|SDF2L1|SRD5A3|DERL3|SLC51B|B4GALNT2|AQP11|MUC5B|GXYLT2
-1.046281318 0 48 0 0 -6.128980014 3.002345416
1 -2.438606207 GO:0043413 M1 1 0 GO Biological Processes 19 macromolecule glycosylation -2.438606207 2.768961517 3.401213073 28162 253 402 10
2.487562189 0.776790586
957|23333|23753|79644|91319|123264|124872|282679|727897|727936
ENTPD5|DPY19L1|SDF2L1|SRD5A3|DERL3|SLC51B|B4GALNT2|AQP11|MUC5B|GXYLT2
-1.046281318 0 48 0 0 -6.128980014 3.002345416
1 -6.102332248 GO:0007584 M1 1 0 GO Biological Processes 19 response to nutrient -6.102332248 5.51946329 7.00648858 28162 165 402 13 3.233830846
0.882281956
360|1030|1277|2182|3371|3627|5468|6566|6678|6696|8942|9388|340024
AQP3|CDKN2B|COL1A1|ACSL4|TNC|CXCL10|PPARG|SLC16A1|SPARC|SPP1|KYNU|LIPG|SLC6A19 -4.055349605 0 49 1 1 -6.102332248 8.241732514
1 -5.758624914 GO:0009991 M1 1 0 GO Biological Processes 19 response to extracellular stimulus -5.758624914 3.231035598 5.913856162 28162 477 402 22

5.472636816 1.134394361
360|1030|1277|2182|3371|3627|4067|4316|4925|5105|5166|5468|5967|6566|6678|6696|8942|9388|10891|55198|130399|340024
AQP3|CDKN2B|COL1A1|ACSL4|TNC|CXCL10|LYN|MMP7|NUCB2|PCK1|PDK4|PPARG|REG1A|SLC16A1|SPARC|SPP1|KYNU|LIPG|PPARGC1A|APPL2|ACVR1C|SLC6A19 -3.743694235 0
49 0 0 -6.102332248 8.241732514
1 -5.587712828 GO:0031667 M1 1 0 GO Biological Processes 19 response to nutrient levels -5.587712828 3.261971738 5.827424802 28162 451 402 21
5.223880597 1.109770192
360|1030|1277|2182|3371|3627|4316|4925|5105|5166|5468|5967|6566|6678|6696|8942|9388|10891|55198|130399|340024
AQP3|CDKN2B|COL1A1|ACSL4|TNC|CXCL10|MMP7|NUCB2|PCK1|PDK4|PPARG|REG1A|SLC16A1|SPARC|SPP1|KYNU|LIPG|PPARGC1A|APPL2|ACVR1C|SLC6A19 -3.588005053 0 49
0 0 -6.102332248 8.241732514
1 -4.382495583 GO:0033273 M1 1 0 GO Biological Processes 19 response to vitamin -4.382495583 6.297054056 6.022992568 28162 89 402 8 1.990049751
0.696552772 360|1277|3371|3627|5468|6678|6696|8942
AQP3|COL1A1|TNC|CXCL10|PPARG|SPARC|SPP1|KYNU -2.596775809 0 49 0 0
-6.102332248 8.241732514
1 -2.869038279 GO:0033280 M1 1 0 GO Biological Processes 19 response to vitamin D -2.869038279 8.241732514 5.084401558 28162 34 402 4 0.995024876
0.495031064 360|3371|3627|6696 AQP3|TNC|CXCL10|SPP1 -1.375331683 0 49
0 0 -6.102332248 8.241732514
1 -5.983589614 GO:0034341 M1 1 0 GO Biological Processes 19 response to interferon-gamma -5.983589614 4.978508473 6.743313031 28162 197 402 14
3.482587065 0.914409651
834|1906|2633|4843|5468|6356|6362|7098|7474|8942|9076|10346|10410|10581
CASP1|EDN1|GBP1|NOS2|PPARG|CCL11|CCL18|TLR3|WNT5A|KYNU|CLDN1|TRIM22|IFITM3|IFITM2 -3.945755797 0 50 1 1 -5.983589614 4.978508473
1 -4.288666882 GO:0071346 M1 1 0 GO Biological Processes 19 cellular response to interferon-gamma -4.288666882 4.35368356 5.386087337 28162 177 402 11
2.736318408 0.813665012
834|1906|2633|4843|5468|6356|6362|7098|7474|9076|10346
CASP1|EDN1|GBP1|NOS2|PPARG|CCL11|CCL18|TLR3|WNT5A|CLDN1|TRIM22
-2.523853018 0 50 0 0 -5.983589614 4.978508473
1 -3.990117256 GO:0071356 M1 1 0 GO Biological Processes 19 cellular response to tumor necrosis factor -3.990117256 3.31339922 4.814890955 28162 296 402 14
3.482587065 0.914409651
608|834|1116|1277|1906|2633|3373|5105|6356|6362|7057|9076|10891|83483
TNFRSF17|CASP1|CHI3L1|COL1A1|EDN1|GBP1|HYAL1|PCK1|CCL11|CCL18|THBS1|CLDN1|PPARGC1A|PLVAP -2.285557294 0 50 0 0 -5.983589614 4.978508473
1 -3.703314028 GO:0070555 M1 1 0 GO Biological Processes 19 response to interleukin-1 -3.703314028 3.740786359 4.751148562 28162 206 402 11
2.736318408 0.813665012

301|952|1116|1906|2633|3373|3553|3557|5105|6356|6362
ANXA1|CD38|CHI3L1|EDN1|GBP1|HYAL1|IL1B|IL1RN|PCK1|CCL11|CCL18
-2.041937659 0 50 0 0 -5.983589614 4.978508473
1 -3.63910143 GO:0034612 M1 1 0 GO Biological Processes 19 response to tumor necrosis factor -3.63910143 3.064894279 4.47043647 28162 320 402 14
3.482587065 0.914409651
608|834|1116|1277|1906|2633|3373|5105|6356|6362|7057|9076|10891|83483
TNFRSF17|CASP1|CHI3L1|COL1A1|EDN1|GBP1|HYAL1|PCK1|CCL11|CCL18|THBS1|CLDN1|PPARGC1A|PLVAP -1.991792508 0 50 0 0 -5.983589614 4.978508473
1 -2.928005526 GO:0071347 M1 1 0 GO Biological Processes 19 cellular response to interleukin-1 -2.928005526 3.502736318 4.053585187 28162 180 402 9
2.23880597 0.737867616 1116|1906|2633|3373|3553|3557|5105|6356|6362
CHI3L1|EDN1|GBP1|HYAL1|IL1B|IL1RN|PCK1|CCL11|CCL18 -1.421352358 0 50 0
0 -5.983589614 4.978508473
1 -5.915772867 GO:0001894 M1 1 0 GO Biological Processes 19 tissue homeostasis -5.915772867 4.311060084 6.454511821 28162 260 402 16
3.980099502 0.975022334
12|952|1001|2697|3915|5166|5175|6505|6584|6696|7031|7032|9076|64081|79888|124220
SERPINA3|CD38|CDH3|GJA1|LAMC1|PDK4|PECAM1|SLC1A1|SLC22A5|SPP1|TFF1|TFF2|CLDN1|PBLD|LPCAT1|ZG16B -3.883730575 0 51 1 1 -5.915772867 15.92152872
1 -5.743477525 GO:0048871 M1 1 0 GO Biological Processes 19 multicellular organismal homeostasis -5.743477525 3.122594392 5.856026636 28162 516 402 23
5.721393035 1.158362377
12|360|857|952|1001|2697|3553|3915|3934|5166|5175|6338|6505|6584|6696|7031|7032|9076|10891|55198|64081|79888|124220
SERPINA3|AQP3|CAV1|CD38|CDH3|GJA1|IL1B|LAMC1|LCN2|PDK4|PECAM1|SCNN1B|SLC1A1|SLC22A5|SPP1|TFF1|TFF2|CLDN1|PPARGC1A|APPL2|PBLD|LPCAT1|ZG16B
-3.731924621 0 51 0 0 -5.915772867 15.92152872
1 -4.904400767 GO:0030277 M1 1 0 GO Biological Processes 19 maintenance of gastrointestinal epithelium -4.904400767 15.92152872 8.425388682 28162 22 402
5 1.243781095 0.552765814 12|6584|7031|7032|64081
SERPINA3|SLC22A5|TFF1|TFF2|PBLD -3.025978422 0 51 0 0 -5.915772867
15.92152872
1 -4.318091631 GO:0060249 M1 1 0 GO Biological Processes 19 anatomical structure homeostasis -4.318091631 2.856308586 4.862526021 28162 466 402 19
4.726368159 1.058368993
12|952|1001|1134|2697|3915|5028|5166|5175|5570|6505|6584|6696|7031|7032|9076|64081|79888|124220
SERPINA3|CD38|CDH3|CHRNA1|GJA1|LAMC1|P2RY1|PDK4|PECAM1|PKIB|SLC1A1|SLC22A5|SPP1|TFF1|TFF2|CLDN1|PBLD|LPCAT1|ZG16B -2.545266838 0 51 0 0
-5.915772867 15.92152872
1 -4.285967059 GO:0010669 M1 1 0 GO Biological Processes 19 epithelial structure maintenance -4.285967059 12.0784011 7.182826772 28162 29 402 5

1.243781095 0.552765814 12|6584|7031|7032|64081 SERPINA3|SLC22A5|TFF1|TFF2|PBLD -2.522285645 0 51 0 0 -5.915772867 15.92152872

1 -3.069995113 GO:0007586 M1 1 0 GO Biological Processes 19 digestion -3.069995113 4.061143558 4.33795967 28162 138 402 8 1.990049751 0.696552772 12|2981|6584|7031|7032|10891|64081|388743 SERPINA3|GUCA2B|SLC22A5|TFF1|TFF2|PPARGC1A|PBLD|CAPN8 -1.530193448 0 51 0 0 -5.915772867 15.92152872

1 -5.792122694 GO:0035821 M1 1 0 GO Biological Processes 19 modulation of process of other organism -5.792122694 6.26505683 7.042091936 28162 123 402 11 2.736318408 0.813665012 1670|1671|2040|4843|5068|5272|5967|5968|6280|6372|64922 DEFA5|DEFA6|STOM|NOS2|REG3A|SERPINB9|REG1A|REG1B|S100A9|CXCL6|LRRC19 -3.768392511 0 52 1 1 -5.792122694 13.13526119

1 -4.858742268 GO:0001906 M1 1 0 GO Biological Processes 19 cell killing -4.858742268 4.593752549 5.869281679 28162 183 402 12 2.985074627 0.848757934 718|1604|1670|1671|3575|4843|5272|6283|6372|11126|23705|57823 C3|CD55|DEFA5|DEFA6|IL7R|NOS2|SERPINB9|S100A12|CXCL6|CD160|CADM1|SLAMF7 -2.989698582 0 52 0 0 -5.792122694 13.13526119

1 -3.408584256 GO:0031640 M1 1 0 GO Biological Processes 19 killing of cells of other organism -3.408584256 6.273557585 5.200605462 28162 67 402 6 1.492537313 0.604761504 1670|1671|4843|5272|6283|6372 DEFA5|DEFA6|NOS2|SERPINB9|S100A12|CXCL6 -1.810201542 0 52 0 0 -5.792122694 13.13526119

1 -3.20501917 GO:0031341 M1 1 0 GO Biological Processes 19 regulation of cell killing -3.20501917 4.855278065 4.670809494 28162 101 402 7 1.741293532 0.652391792 1604|3575|4843|5272|6372|11126|23705 CD55|IL7R|NOS2|SERPINB9|CXCL6|CD160|CADM1 -1.640910111 0 52 0 0 -5.792122694 13.13526119

1 -3.025884506 GO:0001910 M1 1 0 GO Biological Processes 19 regulation of leukocyte mediated cytotoxicity -3.025884506 5.320612129 4.627691592 28162 79 402 6 1.492537313 0.604761504 3575|4843|5272|6372|11126|23705 IL7R|NOS2|SERPINB9|CXCL6|CD160|CADM1 -1.499355638 0 52 0 0 -5.792122694 13.13526119

1 -2.894604439 GO:0001909 M1 1 0 GO Biological Processes 19 leukocyte mediated cytotoxicity -2.894604439 4.301606005 4.25063735 28162 114 402 7 1.741293532 0.652391792 3575|4843|5272|6372|11126|23705|57823 IL7R|NOS2|SERPINB9|CXCL6|CD160|CADM1|SLAMF7 -1.394780918 0 52 0 0 -5.792122694 13.13526119

1 -2.85139329 GO:0051709 M1 1 0 GO Biological Processes 19 regulation of killing of cells of other organism -2.85139329 13.13526119 5.842895378 28162 16 402 3 0.746268657 0.429247719 4843|5272|6372 NOS2|SERPINB9|CXCL6 -1.363728798 0 52 0 0 -5.792122694 13.13526119

1 -5.789829459 GO:0043408 M1 1 0 GO Biological Processes 19 regulation of

MAPK cascade -5.789829459 2.754961149 5.707733155 28162 712 402 28
6.965174129 1.269625794
183|857|1116|1906|1908|1942|2263|2357|2633|2690|3553|4067|5028|5320|5801|6091|6283|6356|6362|7057|7098|7474|10979|26585|55532|60675|154043|197259
AGT|CAV1|CHI3L1|EDN1|EDN3|EFNA1|FGFR2|FPR1|GBP1|GHR|IL1B|LYN|P2RY1|PLA2G2A|PTPRR|ROBO1|S100A12|CCL11|CCL18|THBS1|TLR3|WNT5A|FERMT2|GREM1|SLC30A10|PROK2|CNKSR3|MLKL -3.768152682 0 53 1 1 -5.789829459 42.03283582
1 -5.128492463 GO:0033674 M1 1 0 GO Biological Processes 19 positive regulation of kinase activity -5.128492463 2.788247816 5.341730384 28162 603 402 24
5.970149254 1.181714164
183|994|1116|1906|1908|1942|2263|2357|2690|3553|4067|6091|6283|7057|7098|7436|7474|10451|10979|26585|28984|57214|60675|197259
AGT|CDC25B|CHI3L1|EDN1|EDN3|EFNA1|FGFR2|FPR1|GHR|IL1B|LYN|ROBO1|S100A12|THBS1|TLR3|VLDLR|WNT5A|VAV3|FERMT2|GREM1|RGCC|CEMIP|PROK2|MLKL
-3.216642471 0 53 0 0 -5.789829459 42.03283582
1 -5.068043357 GO:0043410 M1 1 0 GO Biological Processes 19 positive regulation of MAPK cascade -5.068043357 2.924485731 5.366827055 28162 527 402 22
5.472636816 1.134394361
183|1116|1906|1908|1942|2263|2357|2690|3553|5028|5320|6091|6283|6356|6362|7057|7098|7474|10979|55532|60675|197259
AGT|CHI3L1|EDN1|EDN3|EFNA1|FGFR2|FPR1|GHR|IL1B|P2RY1|PLA2G2A|ROBO1|S100A12|CCL11|CCL18|THBS1|TLR3|WNT5A|FERMT2|SLC30A10|PROK2|MLKL -3.1609398 0
53 0 0 -5.789829459 42.03283582
1 -4.94224474 GO:0071900 M1 1 0 GO Biological Processes 19 regulation of protein serine/threonine kinase activity -4.94224474 2.960058861 5.305292125
28162 497 402 21 5.223880597 1.109770192
183|857|1030|1906|1908|2357|2690|3553|3627|4067|5570|6091|6283|7057|7474|10979|28951|28984|57214|60675|197259
AGT|CAV1|CDKN2B|EDN1|EDN3|FPR1|GHR|IL1B|CXCL10|LYN|PKIB|ROBO1|S100A12|THBS1|WNT5A|FERMT2|TRIB2|RGCC|CEMIP|PROK2|MLKL -3.056479181 0 53 0 0
-5.789829459 42.03283582
1 -4.864334182 GO:0045859 M1 1 0 GO Biological Processes 19 regulation of protein kinase activity -4.864334182 2.458060574 5.028223271 28162 798 402 28
6.965174129 1.269625794
183|857|994|1030|1116|1906|1908|1942|2357|2690|3553|3627|4067|5570|6091|6283|7057|7098|7436|7453|7474|10979|26585|28951|28984|57214|60675|197259
AGT|CAV1|CDC25B|CDKN2B|CHI3L1|EDN1|EDN3|EFNA1|FPR1|GHR|IL1B|CXCL10|LYN|PKIB|ROBO1|S100A12|THBS1|TLR3|VLDLR|WARS1|WNT5A|FERMT2|GREM1|TRIB2|RGCC|CEMIP|PROK2|MLKL -2.993442799 0 53 0 0 -5.789829459 42.03283582
1 -4.723657233 GO:0051347 M1 1 0 GO Biological Processes 19 positive regulation of transferase activity -4.723657233 2.571759411 4.996555219 28162 681 402 25
6.218905473 1.204485566
183|994|1116|1906|1908|1942|2263|2357|2690|3553|4067|5570|6091|6283|7057|7098|7436|7474|10451|10979|26585|28984|57214|60675|197259

AGT|CDC25B|CHI3L1|EDN1|EDN3|EFNA1|FGFR2|FPR1|GHR|IL1B|LYN|PKIB|ROBO1|S100A12|THBS1|TLR3|VLDLR|WNT5A|VAV3|FERMT2|GREM1|RGCC|CEMIP|PROK2|MLKL -2.872574243 0 53 0 0 -5.789829459 42.03283582

1 -4.578220964 GO:0016264 M1 1 0 GO Biological Processes 19 gap junction assembly -4.578220964 21.55530042 8.920544234 28162 13 402 4 0.995024876 0.495031064 183|857|2697|3553 AGT|CAV1|GJA1|IL1B -2.755253132 0 53 0 0 -5.789829459 42.03283582

1 -4.548790617 GO:1903596 M1 1 0 GO Biological Processes 19 regulation of gap junction assembly -4.548790617 42.03283582 11.04207458 28162 5 402 3 0.746268657 0.429247719 183|857|3553AGT|CAV1|IL1B -2.738597074 0 53 0 0 -5.789829459 42.03283582

1 -4.530304245 GO:0045860 M1 1 0 GO Biological Processes 19 positive regulation of protein kinase activity -4.530304245 2.775753309 4.966400503 28162 530 402 21 5.223880597 1.109770192 183|994|1116|1906|1908|1942|2357|2690|3553|6091|6283|7057|7098|7436|7474|10979|26585|28984|57214|60675|197259 AGT|CDC25B|CHI3L1|EDN1|EDN3|EFNA1|FPR1|GHR|IL1B|ROBO1|S100A12|THBS1|TLR3|VLDLR|WNT5A|FERMT2|GREM1|RGCC|CEMIP|PROK2|MLKL -2.722621081 0 53 0 0 -5.789829459 42.03283582

1 -4.48850106 GO:0051091 M1 1 0 GO Biological Processes 19 positive regulation of DNA-binding transcription factor activity -4.48850106 3.687090861 5.298824492 28162 266 402 14 3.482587065 0.914409651 183|857|1906|3553|5468|6279|6280|6283|7098|7474|10346|10891|26585|28984 AGT|CAV1|EDN1|IL1B|PPARG|S100A8|S100A9|S100A12|TLR3|WNT5A|TRIM22|PPARGC1A|GREM1|RGCC -2.685795539 0 53 0 0 -5.789829459 42.03283582

1 -4.314217171 GO:0043405 M1 1 0 GO Biological Processes 19 regulation of MAP kinase activity -4.314217171 3.368015691 5.061469276 28162 312 402 15 3.731343284 0.945283402 183|857|1906|1908|2357|2690|3553|4067|6091|6283|7057|7474|28951|60675|197259 AGT|CAV1|EDN1|EDN3|FPR1|GHR|IL1B|LYN|ROBO1|S100A12|THBS1|WNT5A|TRIB2|PROK2|MLKL -2.543696332 0 53 0 0 -5.789829459 42.03283582

1 -4.28279234 GO:0071902 M1 1 0 GO Biological Processes 19 positive regulation of protein serine/threonine kinase activity -4.28279234 3.346563362 5.031847104 28162 314 402 15 3.731343284 0.945283402 183|1906|1908|2357|2690|3553|6091|6283|7057|7474|10979|28984|57214|60675|197259 AGT|EDN1|EDN3|FPR1|GHR|IL1B|ROBO1|S100A12|THBS1|WNT5A|FERMT2|RGCC|CEMIP|PROK2|MLKL -2.520240432 0 53 0 0 -5.789829459 42.03283582

1 -3.970462979 GO:0051092 M1 1 0 GO Biological Processes 19 positive regulation of NF-kappaB transcription factor activity -3.970462979 4.378420398 5.157111108 28162 160 402 10 2.487562189 0.776790586 183|857|3553|6279|6280|6283|7098|7474|10346|26585 AGT|CAV1|IL1B|S100A8|S100A9|S100A12|TLR3|WNT5A|TRIM22|GREM1

-2.269113421 0 53 0 0 -5.789829459 42.03283582
1 -3.841190965 GO:0043406 M1 1 0 GO Biological Processes 19 positive regulation of MAP kinase activity -3.841190965 3.60796874 4.810386599 28162 233 402 12 2.985074627 0.848757934 183|1906|1908|2357|2690|3553|6091|6283|7057|7474|60675|197259 AGT|EDN1|EDN3|FPR1|GHR|IL1B|ROBO1|S100A12|THBS1|WNT5A|PROK2|MLKL -2.15734605 0 53 0 0 -5.789829459 42.03283582
1 -3.340468474 GO:0007169 M1 1 0 GO Biological Processes 19 transmembrane receptor protein tyrosine kinase signaling pathway -3.340468474 2.17737663 3.905791355 28162 740 402 23 5.721393035 1.158362377 183|1001|1277|1282|1942|2263|2690|3488|3553|4067|4313|4318|4688|5166|5801|6091|7057|7474|10451|23433|26585|51237|55532 AGT|CDH3|COL1A1|COL4A1|EFNA1|FGFR2|GHR|IGFBP5|IL1B|LYN|MMP2|MMP9|NCF2|PDK4|PTPRR|ROBO1|THBS1|WNT5A|VAV3|RHOQ|GREM1|MZB1|SLC30A10 -1.750946757 0 53 0 0 -5.789829459 42.03283582
1 -2.746837451 GO:0000187 M1 1 0 GO Biological Processes 19 activation of MAPK activity -2.746837451 3.615727813 3.929637011 28162 155 402 8 1.990049751 0.696552772 183|2357|2690|3553|7057|7474|60675|197259 AGT|FPR1|GHR|IL1B|THBS1|WNT5A|PROK2|MLKL -1.284186033 0 53 0 0 -5.789829459 42.03283582
1 -2.688803131 GO:1901888 M1 1 0 GO Biological Processes 19 regulation of cell junction assembly -2.688803131 3.233295063 3.765891491 28162 195 402 9 2.23880597 0.737867616 183|857|2697|3553|7057|7474|9076|10979|26585 AGT|CAV1|GJA1|IL1B|THBS1|WNT5A|CLDN1|FERMT2|GREM1 -1.237911023 0 53 0 0 -5.789829459 42.03283582
1 -2.543845138 GO:0032147 M1 1 0 GO Biological Processes 19 activation of protein kinase activity -2.543845138 2.562977794 3.426330484 28162 328 402 12 2.985074627 0.848757934 183|1116|2357|2690|3553|7057|7098|7474|26585|28984|60675|197259 AGT|CHI3L1|FPR1|GHR|IL1B|THBS1|TLR3|WNT5A|GREM1|RGCC|PROK2|MLKL -1.123715909 0 53 0 0 -5.789829459 42.03283582
1 -2.415383565 GO:0090287 M1 1 0 GO Biological Processes 19 regulation of cellular response to growth factor stimulus -2.415383565 2.594619495 3.324551785 28162 297 402 11 2.736318408 0.813665012 183|857|1030|3553|5468|6091|7057|7474|11167|26585|64081 AGT|CAV1|CDKN2B|IL1B|PPARG|ROBO1|THBS1|WNT5A|FSTL1|GREM1|PBLD -1.027841704 0 53 0 0 -5.789829459 42.03283582
1 -2.297681472 GO:0051090 M1 1 0 GO Biological Processes 19 regulation of DNA-binding transcription factor activity -2.297681472 2.208932813 3.089868788 28162 444 402 14 3.482587065 0.914409651 183|857|1906|3553|5468|6279|6280|6283|7098|7474|10346|10891|26585|28984 AGT|CAV1|EDN1|IL1B|PPARG|S100A8|S100A9|S100A12|TLR3|WNT5A|TRIM22|PPARGC1A|GREM1|RGCC -0.933295121 0 53 0 0 -5.789829459 42.03283582
1 -5.653968644 GO:0070492 M1 1 0 GO Molecular Functions 21 oligosaccharide

binding -5.653968644 21.89210199 10.05914617 28162 16 402 5 1.243781095
0.552765814 4017|5068|5967|5968|6402LOXL2|REG3A|REG1A|REG1B|SELL
-3.65230898 0 54 1 1 -5.653968644 52.54104478
1 -4.942093822 GO:0044278 M1 1 0 GO Biological Processes 19 cell wall disruption in other organism -4.942093822 52.54104478 12.40536775 28162 4 402 3 0.746268657 0.429247719 5068|5967|5968 REG3A|REG1A|REG1B
-3.056479181 0 54 0 0 -5.653968644 52.54104478
1 -3.302350223 GO:0030246 M1 1 0 GO Molecular Functions 21 carbohydrate binding -3.302350223 3.148527028 4.244832923 28162 267 402 12 2.985074627
0.848757934 682|1116|4017|4885|5068|5967|5968|6402|22918|55214|83998|124220
BSG|CHI3L1|LOXL2|NPTX2|REG3A|REG1A|REG1B|SELL|CD93|P3H2|REG4|ZG16B
-1.72149345 0 54 0 0 -5.653968644 52.54104478
1 -2.697083141 GO:0042834 M1 1 0 GO Molecular Functions 21 peptidoglycan binding -2.697083141 11.67578773 5.452185248 28162 18 402 3 0.746268657
0.429247719 5068|5967|5968 REG3A|REG1A|REG1B -1.24394016 0 54 0 0
-5.653968644 52.54104478
1 -5.49177467 GO:0048306 M1 1 0 GO Molecular Functions 21
calcium-dependent protein binding -5.49177467 7.505863539 7.18602692
28162 84 402 9 2.23880597 0.737867616
301|1755|6279|6280|6282|6283|6286|7436|57126
ANXA1|DMBT1|S100A8|S100A9|S100A11|S100A12|S100P|VLDLR|CD177
-3.514829894 0 55 1 1 -5.49177467 11.67578773
1 -2.697083141 GO:0098641 M1 1 0 GO Molecular Functions 21 cadherin binding involved in cell-cell adhesion -2.697083141 11.67578773 5.452185248 28162 18 402 3 0.746268657 0.429247719 301|6282|23650 ANXA1|S100A11|TRIM29
-1.24394016 0 55 0 0 -5.49177467 11.67578773
1 -2.282036554 GO:0098632 M1 1 0 GO Molecular Functions 21 cell-cell adhesion mediator activity -2.282036554 5.718753173 3.978313804 28162 49 402 4
0.995024876 0.495031064 301|682|6282|23650 ANXA1|BSG|S100A11|TRIM29
-0.923653081 0 55 0 0 -5.49177467 11.67578773
1 -2.023879618 GO:0098631 M1 1 0 GO Molecular Functions 21 cell adhesion mediator activity -2.023879618 4.831360439 3.514878126 28162 58 402 4
0.995024876 0.495031064 301|682|6282|23650 ANXA1|BSG|S100A11|TRIM29
-0.719622106 0 55 0 0 -5.49177467 11.67578773
1 -5.487187569 GO:0030855 M1 1 0 GO Biological Processes 19 epithelial cell differentiation -5.487187569 2.601263847 5.46123789 28162 781 402 29
7.213930348 1.290370231
301|360|857|1001|1282|1306|1513|1755|2263|3553|4318|4478|5068|5105|5175|5266|5308|5468|7068|7474|9076|11005|11167|23596|23657|26585|55151|79983|284217
ANXA1|AQP3|CAV1|CDH3|COL4A1|COL15A1|CTSK|DMBT1|FGFR2|IL1B|MMP9|MSN|REG3A|PCK1|PECAM1|PI3|PITX2|PPARG|THRB|WNT5A|CLDN1|SPINK5|FSTL1|OPN3|SLC7A11|GREM1|TMEM38B|POF1B|LAMA1 -3.512086933 0 56 1 1 -5.487187569 2.601263847
1 -5.478297501 GO:0072593 M1 1 0 GO Biological Processes 19 reactive oxygen species metabolic process -5.478297501 3.988881217 6.059474671 28162 281 402

16 3.980099502 0.975022334
51|183|240|857|1906|3553|4314|4688|4843|5166|7057|7837|50506|55532|57126|405753
ACOX1|AGT|ALOX5|CAV1|EDN1|IL1B|MMP3|NCF2|NOS2|PDK4|THBS1|PXDN|DUOX2|SLC30A10|CD177|DUOXA2 -3.505033209 0 57 1 1 -5.478297501 6.834607451
1 -4.150466084 GO:1903409 M1 1 0 GO Biological Processes 19 reactive oxygen species biosynthetic process -4.150466084 5.125955588 5.518527057 28162 123 402 9 2.23880597 0.737867616 51|240|857|1906|3553|4843|50506|55532|405753
ACOX1|ALOX5|CAV1|EDN1|IL1B|NOS2|DUOX2|SLC30A10|DUOXA2 -2.409908469 0 57 0 0 -5.478297501 6.834607451
1 -3.793694029 GO:0140353 M1 1 0 GO Biological Processes 19 lipid export from cell -3.793694029 6.054112155 5.481613763 28162 81 402 7 1.741293532 0.652391792 183|1906|2697|3553|4843|6505|6696
AGT|EDN1|GJA1|IL1B|NOS2|SLC1A1|SPP1 -2.119623389 0 57 0 0 -5.478297501 6.834607451
1 -3.331804253 GO:2000377 M1 1 0 GO Biological Processes 19 regulation of reactive oxygen species metabolic process -3.331804253 3.648683665 4.431612781 28162 192 402 10 2.487562189 0.776790586
183|240|857|1906|3553|4314|7057|55532|57126|405753
AGT|ALOX5|CAV1|EDN1|IL1B|MMP3|THBS1|SLC30A10|CD177|DUOXA2
-1.745719453 0 57 0 0 -5.478297501 6.834607451
1 -2.78138543 GO:1905952 M1 1 0 GO Biological Processes 19 regulation of lipid localization -2.78138543 3.335939351 3.877486319 28162 189 402 9 2.23880597 0.737867616 183|718|857|1906|3553|5468|6696|7057|9388
AGT|C3|CAV1|EDN1|IL1B|PPARG|SPP1|THBS1|LIPG -1.305114044 0 57 0 0 -5.478297501 6.834607451
1 -2.729287486 GO:0032368 M1 1 0 GO Biological Processes 19 regulation of lipid transport -2.729287486 3.59255007 3.907430315 28162 156 402 8 1.990049751 0.696552772 183|857|1906|3553|5468|6696|7057|9388
AGT|CAV1|EDN1|IL1B|PPARG|SPP1|THBS1|LIPG -1.268892148 0 57 0 0 -5.478297501 6.834607451
1 -2.563886473 GO:0033574 M1 1 0 GO Biological Processes 19 response to testosterone -2.563886473 6.834607451 4.498995123 28162 41 402 4 0.995024876 0.495031064 1906|4478|6696|7057 EDN1|MSN|SPP1|THBS1
-1.138080112 0 57 0 0 -5.478297501 6.834607451
1 -2.523609777 GO:1903426 M1 1 0 GO Biological Processes 19 regulation of reactive oxygen species biosynthetic process -2.523609777 4.245740992 3.893065777 28162 99 402 6 1.492537313 0.604761504 240|857|1906|3553|55532|405753
ALOX5|CAV1|EDN1|IL1B|SLC30A10|DUOXA2 -1.108577961 0 57 0 0 -5.478297501 6.834607451
1 -2.480423272 GO:2000379 M1 1 0 GO Biological Processes 19 positive regulation of reactive oxygen species metabolic process -2.480423272 4.161666913 3.830474374 28162 101 402 6 1.492537313 0.604761504 183|1906|3553|7057|57126|405753
AGT|EDN1|IL1B|THBS1|CD177|DUOXA2 -1.076893306 0 57 0 0

-5.478297501 6.834607451
1 -2.279718431 GO:1905954 M1 1 0 GO Biological Processes 19 positive regulation of lipid localization -2.279718431 3.786741966 3.540066375 28162 111 402 6 1.492537313 0.604761504 718|857|1906|3553|6696|9388 C3|CAV1|EDN1|IL1B|SPP1|LIPG -0.923653081 0 57 0 0 -5.478297501 6.834607451
1 -2.127197601 GO:0032370 M1 1 0 GO Biological Processes 19 positive regulation of lipid transport -2.127197601 4.120866257 3.467660739 28162 85 402 5 1.243781095 0.552765814 857|1906|3553|6696|9388 CAV1|EDN1|IL1B|SPP1|LIPG -0.801613334 0 57 0 0 -5.478297501 6.834607451
1 -5.441134285 GO:0070633 M1 1 0 GO Biological Processes 19 transepithelial transport -5.441134285 14.01094527 8.580182099 28162 30 402 6 1.492537313 0.604761504 1906|5243|6338|6505|9429|9963 EDN1|ABCB1|SCNN1B|SLC1A1|ABCG2|SLC23A1 -3.478727921 0 58 1 1 -5.441134285 14.01094527
1 -4.636744896 GO:0007588 M1 1 0 GO Biological Processes 19 excretion -4.636744896 8.17305141 6.693269098 28162 60 402 7 1.741293532 0.652391792 183|366|1906|2981|6338|9429|81693 AGT|AQP9|EDN1|GUCA2B|SCNN1B|ABCG2|AMN -2.79658813 0 58 0 0 -5.441134285 14.01094527
1 -2.080705656 GO:0055067 M1 1 0 GO Biological Processes 19 monovalent inorganic cation homeostasis -2.080705656 3.064894279 3.152032005 28162 160 402 7 1.741293532 0.652391792 183|1906|5166|6338|6550|8671|282679 AGT|EDN1|PDK4|SCNN1B|SLC9A3|SLC4A4|AQP11 -0.763697299 0 58 0 0 -5.441134285 14.01094527
1 -5.428222497 GO:0010038 M1 1 0 GO Biological Processes 19 response to metal ion -5.428222497 3.582343962 5.866948257 28162 352 402 18 4.47761194 1.031484687 288|360|366|682|857|1906|4017|4318|4499|6279|6678|7031|7057|7474|9076|10891|55532|63928 ANK3|AQP3|AQP9|BSG|CAV1|EDN1|LOXL2|MMP9|MT1M|S100A8|SPARC|TFF1|THBS1|WNT5A|CLDN1|PPARGC1A|SLC30A10|CHP2 -3.467599685 0 59 1 1 -5.428222497 3.582343962
1 -4.879164162 GO:0010035 M1 1 0 GO Biological Processes 19 response to inorganic substance -4.879164162 2.843549779 5.215188458 28162 542 402 22 5.472636816 1.134394361 288|301|360|366|682|857|1277|1906|4017|4314|4318|4499|6279|6505|6678|7031|7057|7474|9076|10891|55532|63928 ANK3|ANXA1|AQP3|AQP9|BSG|CAV1|COL1A1|EDN1|LOXL2|MMP3|MMP9|MT1M|S100A8|SLC1A1|SPARC|TFF1|THBS1|WNT5A|CLDN1|PPARGC1A|SLC30A10|CHP2 -3.006822712 0 59 0 0 -5.428222497 3.582343962
1 -2.938867608 GO:0071241 M1 1 0 GO Biological Processes 19 cellular response to inorganic substance -2.938867608 3.243274369 3.982692483 28162 216 402 10 2.487562189 0.776790586 288|1906|4314|4318|4499|7474|9076|10891|55532|63928

ANK3|EDN1|MMP3|MMP9|MT1M|WNT5A|CLDN1|PPARGC1A|SLC30A10|CHP2 -1.429689558 0 59 0 0 -5.428222497 3.582343962
1 -2.78138543 GO:0071248 M1 1 0 GO Biological Processes 19 cellular response to metal ion -2.78138543 3.335939351 3.877486319 28162 189 402 9 2.23880597 0.737867616 288|1906|4318|4499|7474|9076|10891|55532|63928 ANK3|EDN1|MMP9|MT1M|WNT5A|CLDN1|PPARGC1A|SLC30A10|CHP2-1.305114044 0 59 0 0 -5.428222497 3.582343962
1 -2.291825379 GO:0051592 M1 1 0 GO Biological Processes 19 response to calcium ion -2.291825379 3.358788251 3.438663282 28162 146 402 7 1.741293532 0.652391792 360|857|1906|6678|7057|7474|63928 AQP3|CAV1|EDN1|SPARC|THBS1|WNT5A|CHP2 -0.929240126 0 59 0 0 -5.428222497 3.582343962
1 -5.380438795 GO:0055086 M1 1 0 GO Biological Processes 19 nucleobase-containing small molecule metabolic process -5.380438795 2.811184846 5.501233109 28162 623 402 25 6.218905473 1.204485566 957|1503|1836|2182|2981|3158|3620|4199|4837|4843|5166|5209|6241|6999|8671|8942|9582|10891|10924|23433|53354|55790|60481|80221|124872 ENTPD5|CTPS1|SLC26A2|ACSL4|GUCA2B|HMGCS2|IDO1|ME1|NNMT|NOS2|PDK4|PFKFB3|RRM2|TDO2|SLC4A4|KYNU|APOBEC3B|PPARGC1A|SMPDL3A|RHOQ|PANK1|CSGALNACT1|ELOVL5|ACSF2|B4GALNT2 -3.425123109 0 60 1 1 -5.380438795 7.374181723
1 -5.323910838 GO:0090407 M1 1 0 GO Biological Processes 19 organophosphate biosynthetic process -5.323910838 2.864247756 5.492780585 28162 587 402 24 5.970149254 1.181714164 1503|1836|2182|2981|3158|3620|4199|4837|4843|5028|5166|5320|6241|8942|10891|27010|53354|55151|56895|60481|79644|79888|80157|80235 CTPS1|SLC26A2|ACSL4|GUCA2B|HMGCS2|IDO1|ME1|NNMT|NOS2|P2RY1|PDK4|PLA2G2A|RRM2|KYNU|PPARGC1A|TPK1|PANK1|TMEM38B|AGPAT4|ELOVL5|SRD5A3|LPCAT1|CWH43|PIGZ -3.379485178 0 60 0 0 -5.380438795 7.374181723
1 -4.817914324 GO:0006753 M1 1 0 GO Biological Processes 19 nucleoside phosphate metabolic process -4.817914324 2.81755755 5.165788753 28162 547 402 22 5.472636816 1.134394361 957|1503|1836|2182|2981|3158|3620|4199|4837|4843|5166|5209|6241|6999|8671|8942|10891|10924|23433|53354|60481|80221 ENTPD5|CTPS1|SLC26A2|ACSL4|GUCA2B|HMGCS2|IDO1|ME1|NNMT|NOS2|PDK4|PFKFB3|RRM2|TDO2|SLC4A4|KYNU|PPARGC1A|SMPDL3A|RHOQ|PANK1|ELOVL5|ACSF2 -2.95277527 0 60 0 0 -5.380438795 7.374181723
1 -4.524416301 GO:0009165 M1 1 0 GO Biological Processes 19 nucleotide biosynthetic process -4.524416301 3.715023368 5.333549527 28162 264 402 14 3.482587065 0.914409651 1503|1836|2182|2981|3620|4199|4837|4843|5166|6241|8942|10891|53354|60481 CTPS1|SLC26A2|ACSL4|GUCA2B|IDO1|ME1|NNMT|NOS2|PDK4|RRM2|KYNU|PPARGC1A|PANK1|ELOVL5 -2.719229089 0 60 0 0 -5.380438795 7.374181723
1 -4.470678859 GO:1901293 M1 1 0 GO Biological Processes 19 nucleoside phosphate biosynthetic process -4.470678859 3.673281532 5.281587486 28162 267

402 14 3.482587065 0.914409651
1503|1836|2182|2981|3620|4199|4837|4843|5166|6241|8942|10891|53354|60481
CTPS1|SLC26A2|ACSL4|GUCA2B|IDO1|ME1|NNMT|NOS2|PDK4|RRM2|KYNU|PPARGC1A|PANK1|ELOVL5 -2.670541078 0 60 0 0 -5.380438795 7.374181723
1 -4.436144172 GO:0009117 M1 1 0 GO Biological Processes 19 nucleotide metabolic process -4.436144172 2.734478167 4.888144374 28162 538 402 21 5.223880597 1.109770192
957|1503|1836|2182|2981|3158|3620|4199|4837|4843|5166|5209|6241|6999|8671|8942|10891|23433|53354|60481|80221
ENTPD5|CTPS1|SLC26A2|ACSL4|GUCA2B|HMGCS2|IDO1|ME1|NNMT|NOS2|PDK4|PFKFB3|RRM2|TDO2|SLC4A4|KYNU|PPARGC1A|RHOQ|PANK1|ELOVL5|ACSF2 -2.64201456 0 60 0 0 -5.380438795 7.374181723
1 -3.911616916 GO:0006790 M1 1 0 GO Biological Processes 19 sulfur compound metabolic process -3.911616916 2.965279423 4.629138276 28162 378 402 16 3.980099502 0.975022334
1836|2182|2690|3158|3373|5166|6505|6999|8942|23657|27010|51363|55790|60481|80221|80704
SLC26A2|ACSL4|GHR|HMGCS2|HYAL1|PDK4|SLC1A1|TDO2|KYNU|SLC7A11|TPK1|CHST15|CSGALNACT1|ELOVL5|ACSF2|SLC19A3 -2.218742713 0 60 0 0 -5.380438795 7.374181723
1 -3.720362833 GO:0033865 M1 1 0 GO Biological Processes 19 nucleoside bisphosphate metabolic process -3.720362833 4.503518124 5.000876781 28162 140 402 9 2.23880597 0.737867616
1836|2182|3158|5166|6999|8942|53354|60481|80221
SLC26A2|ACSL4|HMGCS2|PDK4|TDO2|KYNU|PANK1|ELOVL5|ACSF2 -2.05449527 0 60 0 0 -5.380438795 7.374181723
1 -3.720362833 GO:0033875 M1 1 0 GO Biological Processes 19 ribonucleoside bisphosphate metabolic process -3.720362833 4.503518124 5.000876781 28162 140 402 9 2.23880597 0.737867616
1836|2182|3158|5166|6999|8942|53354|60481|80221
SLC26A2|ACSL4|HMGCS2|PDK4|TDO2|KYNU|PANK1|ELOVL5|ACSF2 -2.05449527 0 60 0 0 -5.380438795 7.374181723
1 -3.720362833 GO:0034032 M1 1 0 GO Biological Processes 19 purine nucleoside bisphosphate metabolic process -3.720362833 4.503518124 5.000876781 28162 140 402 9 2.23880597 0.737867616
1836|2182|3158|5166|6999|8942|53354|60481|80221
SLC26A2|ACSL4|HMGCS2|PDK4|TDO2|KYNU|PANK1|ELOVL5|ACSF2 -2.05449527 0 60 0 0 -5.380438795 7.374181723
1 -3.161255796 GO:0006163 M1 1 0 GO Biological Processes 19 purine nucleotide metabolic process -3.161255796 2.535917697 3.916605216 28162 442 402 16 3.980099502 0.975022334
957|1836|2182|2981|3158|4843|5166|5209|6999|8671|8942|10891|23433|53354|60481|80221
ENTPD5|SLC26A2|ACSL4|GUCA2B|HMGCS2|NOS2|PDK4|PFKFB3|TDO2|SLC4A4|KYNU|PP

ARGC1A|RHOQ|PANK1|ELOVL5|ACSF2-1.603528322 0 60 0 0 -5.380438795
7.374181723
1 -3.104419903 GO:0006637 M1 1 0 GO Biological Processes 19 acyl-CoA metabolic process -3.104419903 4.670315091 4.534242202 28162 105 402 7
1.741293532 0.652391792 2182|3158|5166|6999|8942|60481|80221
ACSL4|HMGCS2|PDK4|TDO2|KYNU|ELOVL5|ACSF2 -1.554366909 0 60 0 0
-5.380438795 7.374181723
1 -3.104419903 GO:0035383 M1 1 0 GO Biological Processes 19 thioester metabolic process -3.104419903 4.670315091 4.534242202 28162 105 402 7
1.741293532 0.652391792 2182|3158|5166|6999|8942|60481|80221
ACSL4|HMGCS2|PDK4|TDO2|KYNU|ELOVL5|ACSF2 -1.554366909 0 60 0 0
-5.380438795 7.374181723
1 -2.979354302 GO:0072521 M1 1 0 GO Biological Processes 19 purine-containing compound metabolic process -2.979354302 2.436686135 3.738630854 28162 460 402
16 3.980099502 0.975022334
957|1836|2182|2981|3158|4843|5166|5209|6999|8671|8942|10891|23433|53354|604
81|80221
ENTPD5|SLC26A2|ACSL4|GUCA2B|HMGCS2|NOS2|PDK4|PFKFB3|TDO2|SLC4A4|KYNU|PP
ARGC1A|RHOQ|PANK1|ELOVL5|ACSF2-1.46294426 0 60 0 0 -5.380438795
7.374181723
1 -2.879449987 GO:0009259 M1 1 0 GO Biological Processes 19 ribonucleotide metabolic process -2.879449987 2.466715717 3.670761458 28162 426 402 15
3.731343284 0.945283402
957|1503|1836|2182|3158|5166|5209|6999|8671|8942|10891|23433|53354|60481|80
221
ENTPD5|CTPS1|SLC26A2|ACSL4|HMGCS2|PDK4|PFKFB3|TDO2|SLC4A4|KYNU|PPARGC1A|
RHOQ|PANK1|ELOVL5|ACSF2 -1.382695697 0 60 0 0 -5.380438795 7.374181723
1 -2.781208263 GO:0019693 M1 1 0 GO Biological Processes 19 ribose phosphate metabolic process -2.781208263 2.410139669 3.570993807 28162 436 402 15
3.731343284 0.945283402
957|1503|1836|2182|3158|5166|5209|6999|8671|8942|10891|23433|53354|60481|80
221
ENTPD5|CTPS1|SLC26A2|ACSL4|HMGCS2|PDK4|PFKFB3|TDO2|SLC4A4|KYNU|PPARGC1A|
RHOQ|PANK1|ELOVL5|ACSF2 -1.305114044 0 60 0 0 -5.380438795 7.374181723
1 -2.716804039 GO:1901570 M1 1 0 GO Biological Processes 19 fatty acid derivative biosynthetic process -2.716804039 5.649574707 4.410453493 28162 62
402 5 1.243781095 0.552765814 240|2182|3158|3248|60481
ALOX5|ACSL4|HMGCS2|HPGD|ELOVL5-1.259212419 0 60 0 0 -5.380438795
7.374181723
1 -2.686650474 GO:0006084 M1 1 0 GO Biological Processes 19 acetyl-CoA metabolic process -2.686650474 7.374181723 4.73156735 28162 38 402 4
0.995024876 0.495031064 3158|5166|6999|8942 HMGCS2|PDK4|TDO2|KYNU
-1.237911023 0 60 0 0 -5.380438795 7.374181723
1 -2.611972743 GO:0009150 M1 1 0 GO Biological Processes 19 purine

ribonucleotide metabolic process -2.611972743 2.397961294 3.427118845 28162 409 402 14 3.482587065 0.914409651 957|1836|2182|3158|5166|5209|6999|8671|8942|10891|23433|53354|60481|80221 ENTPD5|SLC26A2|ACSL4|HMGCS2|PDK4|PFKFB3|TDO2|SLC4A4|KYNU|PPARGC1A|RHOQ|PANK1|ELOVL5|ACSF2 -1.175650263 0 60 0 0 -5.380438795 7.374181723

1 -2.568058643 GO:0033866 M1 1 0 GO Biological Processes 19 nucleoside bisphosphate biosynthetic process -2.568058643 5.227964654 4.169476813 28162 67 402 5 1.243781095 0.552765814 1836|2182|5166|53354|60481 SLC26A2|ACSL4|PDK4|PANK1|ELOVL5 -1.141064218 0 60 0 0 -5.380438795 7.374181723

1 -2.568058643 GO:0034030 M1 1 0 GO Biological Processes 19 ribonucleoside bisphosphate biosynthetic process -2.568058643 5.227964654 4.169476813 28162 67 402 5 1.243781095 0.552765814 1836|2182|5166|53354|60481 SLC26A2|ACSL4|PDK4|PANK1|ELOVL5 -1.141064218 0 60 0 0 -5.380438795 7.374181723

1 -2.568058643 GO:0034033 M1 1 0 GO Biological Processes 19 purine nucleoside bisphosphate biosynthetic process -2.568058643 5.227964654 4.169476813 28162 67 402 5 1.243781095 0.552765814 1836|2182|5166|53354|60481 SLC26A2|ACSL4|PDK4|PANK1|ELOVL5 -1.141064218 0 60 0 0 -5.380438795 7.374181723

1 -2.192178938 GO:1901568 M1 1 0 GO Biological Processes 19 fatty acid derivative metabolic process -2.192178938 4.271629657 3.570259987 28162 82 402 5 1.243781095 0.552765814 240|2182|3158|3248|60481 ALOX5|ACSL4|HMGCS2|HPGD|ELOVL5-0.851060007 0 60 0 0 -5.380438795 7.374181723

1 -2.170278416 GO:0044272 M1 1 0 GO Biological Processes 19 sulfur compound biosynthetic process -2.170278416 2.903822855 3.19369952 28162 193 402 8 1.990049751 0.696552772 1836|2182|5166|23657|27010|51363|55790|60481 SLC26A2|ACSL4|PDK4|SLC7A11|TPK1|CHST15|CSGALNACT1|ELOVL5 -0.835397365 0 60 0 0 -5.380438795 7.374181723

1 -2.118777626 GO:0006164 M1 1 0 GO Biological Processes 19 purine nucleotide biosynthetic process -2.118777626 2.844861984 3.126921042 28162 197 402 8 1.990049751 0.696552772 1836|2182|2981|4843|5166|10891|53354|60481 SLC26A2|ACSL4|GUCA2B|NOS2|PDK4|PPARGC1A|PANK1|ELOVL5 -0.794428906 0 60 0 0 -5.380438795 7.374181723

1 -5.343725946 GO:0071560 M1 1 0 GO Biological Processes 19 cellular response to transforming growth factor beta stimulus -5.343725946 4.120866257 6.02443057 28162 255 402 15 3.731343284 0.945283402 857|1030|1277|1278|1281|1906|2263|3248|7057|7474|9076|10891|10979|55198|64081 CAV1|CDKN2B|COL1A1|COL1A2|COL3A1|EDN1|FGFR2|HPGD|THBS1|WNT5A|CLDN1|PPARGC1A|FERMT2|APPL2|PBLD -3.393653316 0 61 1 1 -5.343725946 4.120866257

1 -5.221518727 GO:0071559 M1 1 0 GO Biological Processes 19 response to transforming growth factor beta -5.221518727 4.026133699 5.910521657 28162 261

402 15 3.731343284 0.945283402
857|1030|1277|1278|1281|1906|2263|3248|7057|7474|9076|10891|10979|55198|64081
CAV1|CDKN2B|COL1A1|COL1A2|COL3A1|EDN1|FGFR2|HPGD|THBS1|WNT5A|CLDN1|PPARGC1A|FERMT2|APPL2|PBLD -3.29180738 0 61 0 0 -5.343725946 4.120866257
1 -4.605529913 GO:0070848 M1 1 0 GO Biological Processes 19 response to growth factor -4.605529913 2.474759355 4.878723026 28162 736 402 26
6.467661692 1.226708902
183|301|857|1030|1277|1278|1281|1906|2263|3248|3371|3373|3553|5028|5468|6091|7057|7474|9076|10891|10979|11167|26585|55198|64081|130399
AGT|ANXA1|CAV1|CDKN2B|COL1A1|COL1A2|COL3A1|EDN1|FGFR2|HPGD|TNC|HYAL1|IL1B|P2RY1|PPARG|ROBO1|THBS1|WNT5A|CLDN1|PPARGC1A|FERMT2|FSTL1|GREM1|APPL2|PBLD|ACVR1C -2.771081098 0 61 0 0 -5.343725946 4.120866257
1 -4.027684498 GO:0071363 M1 1 0 GO Biological Processes 19 cellular response to growth factor stimulus -4.027684498 2.378095379 4.465865007 28162 707 402
24 5.970149254 1.181714164
183|301|857|1030|1277|1278|1281|1906|2263|3248|3373|3553|5468|6091|7057|7474|9076|10891|10979|11167|26585|55198|64081|130399
AGT|ANXA1|CAV1|CDKN2B|COL1A1|COL1A2|COL3A1|EDN1|FGFR2|HPGD|HYAL1|IL1B|PPARG|ROBO1|THBS1|WNT5A|CLDN1|PPARGC1A|FERMT2|FSTL1|GREM1|APPL2|PBLD|ACVR1C
-2.30806079 0 61 0 0 -5.343725946 4.120866257
1 -3.59105215 GO:0007178 M1 1 0 GO Biological Processes 19 transmembrane receptor protein serine/threonine kinase signaling pathway -3.59105215 2.894823404
4.372550137 28162 363 402 15 3.731343284 0.945283402
857|1030|1278|1281|3248|5468|7057|7474|10979|11030|11167|26585|55198|64081|130399
CAV1|CDKN2B|COL1A2|COL3A1|HPGD|PPARG|THBS1|WNT5A|FERMT2|RBPMS|FSTL1|GREM1|APPL2|PBLD|ACVR1C -1.959831662 0 61 0 0 -5.343725946 4.120866257
1 -2.52884799 GO:0007179 M1 1 0 GO Biological Processes 19 transforming growth factor beta receptor signaling pathway -2.52884799 3.060643385 3.572121855
28162 206 402 9 2.23880597 0.737867616
857|1030|1278|1281|3248|7057|10979|55198|64081
CAV1|CDKN2B|COL1A2|COL3A1|HPGD|THBS1|FERMT2|APPL2|PBLD -1.110254277 0
61 0 0 -5.343725946 4.120866257
1 -5.327751874 GO:0032102 M1 1 0 GO Biological Processes 19 negative regulation of response to external stimulus -5.327751874 3.378273607 5.720907934
28162 394 402 19 4.726368159 1.058368993
240|1906|2697|4321|4925|5270|5272|5328|5468|6091|6696|7056|7057|7130|7474|11005|11240|26585|54941
ALOX5|EDN1|GJA1|MMP12|NUCB2|SERPINE2|SERPINB9|PLAU|PPARG|ROBO1|SPP1|THBD|THBS1|TNFAIP6|WNT5A|SPINK5|PADI2|GREM1|RNF125 -3.379485178 0 62 1 1
-5.327751874 5.494488343
1 -5.029756688 GO:0048771 M1 1 0 GO Biological Processes 19 tissue remodeling
-5.029756688 4.776458616 6.047807801 28162 176 402 12 2.985074627

0.848757934 183|857|952|2697|3488|4313|5166|6696|7052|7076|26585|115908
AGT|CAV1|CD38|GJA1|IGFBP5|MMP2|PDK4|SPP1|TGM2|TIMP1|GREM1|CTHRC1
-3.125820702 0 62 0 0 -5.327751874 5.494488343
1 -3.054994218 GO:0001558 M1 1 0 GO Biological Processes 19 regulation of cell growth -3.054994218 2.569244243 3.847021046 28162 409 402 15 3.731343284
0.945283402
183|952|1906|2697|3373|3488|3490|5270|5468|5967|6279|6280|6696|7474|26585
AGT|CD38|EDN1|GJA1|HYAL1|IGFBP5|IGFBP7|SERPINE2|PPARG|REG1A|S100A8|S100A9|SPP1|WNT5A|GREM1 -1.519226276 0 62 0 0 -5.327751874 5.494488343
1 -2.75726963 GO:0046849 M1 1 0 GO Biological Processes 19 bone remodeling
-2.75726963 4.722790542 4.232983 28162 89 402 6 1.492537313
0.604761504 952|2697|5166|6696|26585|115908
CD38|GJA1|PDK4|SPP1|GREM1|CTHRC1 -1.291781529 0 62 0 0
-5.327751874 5.494488343
1 -2.446951571 GO:0016049 M1 1 0 GO Biological Processes 19 cell growth
-2.446951571 2.221608659 3.224306872 28162 473 402 15 3.731343284
0.945283402
183|952|1906|2697|3373|3488|3490|5270|5468|5967|6279|6280|6696|7474|26585
AGT|CD38|EDN1|GJA1|HYAL1|IGFBP5|IGFBP7|SERPINE2|PPARG|REG1A|S100A8|S100A9|SPP1|WNT5A|GREM1 -1.051246942 0 62 0 0 -5.327751874 5.494488343
1 -2.232781541 GO:0040008 M1 1 0 GO Biological Processes 19 regulation of growth -2.232781541 1.948972295 2.938652594 28162 647 402 18 4.47761194
1.031484687
183|952|1906|2263|2690|2697|3373|3488|3490|4499|5270|5468|5967|6279|6280|6696|7474|26585
AGT|CD38|EDN1|FGFR2|GHR|GJA1|HYAL1|IGFBP5|IGFBP7|MT1M|SERPINE2|PPARG|REG1A|S100A8|S100A9|SPP1|WNT5A|GREM1 -0.882189587 0 62 0 0 -5.327751874
5.494488343
1 -2.220014968 GO:0046850 M1 1 0 GO Biological Processes 19 regulation of bone remodeling -2.220014968 5.494488343 3.865934849 28162 51 402 4
0.995024876 0.495031064 952|5166|6696|26585 CD38|PDK4|SPP1|GREM1
-0.8750318 0 62 0 0 -5.327751874 5.494488343
1 -2.046859206 GO:0045926 M1 1 0 GO Biological Processes 19 negative regulation of growth -2.046859206 2.573438928 2.976637108 28162 245 402 9
2.23880597 0.737867616 2697|3373|3488|4499|5270|5468|6696|7474|26585
GJA1|HYAL1|IGFBP5|MT1M|SERPINE2|PPARG|SPP1|WNT5A|GREM1 -0.739448908 0
62 0 0 -5.327751874 5.494488343
1 -5.323996391 GO:0042803 M1 1 0 GO Molecular Functions 21 protein homodimerization activity -5.323996391 2.72260521 5.426440948 28162 669 402
26 6.467661692 1.226708902
51|1670|1671|2040|2053|2263|2633|2690|4038|4843|5175|6282|6286|6723|7453|8942|9429|10346|11030|11240|23255|23705|26585|53354|55198|200931
ACOX1|DEFA5|DEFA6|STOM|EPHX2|FGFR2|GBP1|GHR|LRP4|NOS2|PECAM1|S100A11|S100P|SRM|WARS1|KYNU|ABCG2|TRIM22|RBPMS|PADI2|MTCL1|CADM1|GREM1|PANK1|APPL2

|SLC51A -3.379485178 0 63 1 1 -5.323996391 2.72260521
1 -5.311900384 GO:0022612 M1 1 0 GO Biological Processes 19 gland morphogenesis -5.311900384 6.254886283 6.705531897 28162 112 402 10 2.487562189 0.776790586 857|2263|3371|3488|4478|5268|6356|7052|7474|284217 CAV1|FGFR2|TNC|IGFBP5|MSN|SERPINB5|CCL11|TGM2|WNT5A|LAMA1 -3.373820868 0 64 1 1 -5.311900384 12.36259877
1 -3.96092604 GO:0048732 M1 1 0 GO Biological Processes 19 gland development -3.96092604 2.876643353 4.629066338 28162 414 402 17 4.228855721 1.003727321 301|857|2263|3371|3488|4478|5105|5268|5270|5308|5967|6091|6356|7052|7474|9076|284217 ANXA1|CAV1|FGFR2|TNC|IGFBP5|MSN|PCK1|SERPINB5|SERPINE2|PITX2|REG1A|ROBO1|CCL11|TGM2|WNT5A|CLDN1|LAMA1 -2.262519693 0 64 0 0 -5.311900384 12.36259877
1 -3.692539009 GO:0061138 M1 1 0 GO Biological Processes 19 morphogenesis of a branching epithelium -3.692539009 4.049406148 4.841411128 28162 173 402 10 2.487562189 0.776790586 183|1282|1906|2263|3371|6356|7052|7474|26585|284217 AGT|COL4A1|EDN1|FGFR2|TNC|CCL11|TGM2|WNT5A|GREM1|LAMA1 -2.033835229 0 64 0 0 -5.311900384 12.36259877
1 -3.651269795 GO:0048754 M1 1 0 GO Biological Processes 19 branching morphogenesis of an epithelial tube -3.651269795 4.409038722 4.91814093 28162 143 402 9 2.23880597 0.737867616 183|1282|1906|2263|3371|6356|7474|26585|284217 AGT|COL4A1|EDN1|FGFR2|TNC|CCL11|WNT5A|GREM1|LAMA1 -1.998705714 0 64 0 0 -5.311900384 12.36259877
1 -3.598320886 GO:0060443 M1 1 0 GO Biological Processes 19 mammary gland morphogenesis -3.598320886 8.756840796 5.907709119 28162 40 402 5 1.243781095 0.552765814 857|2263|3488|6356|7474 CAV1|FGFR2|IGFBP5|CCL11|WNT5A -1.962957949 0 64 0 0 -5.311900384 12.36259877
1 -3.546913934 GO:0030850 M1 1 0 GO Biological Processes 19 prostate gland development -3.546913934 8.543259313 5.816515875 28162 41 402 5 1.243781095 0.552765814 301|2263|3371|5268|7474 ANXA1|FGFR2|TNC|SERPINB5|WNT5A-1.91988704 0 64 0 0 -5.311900384 12.36259877
1 -3.53516797 GO:0002009 M1 1 0 GO Biological Processes 19 morphogenesis of an epithelium -3.53516797 2.483283211 4.172293046 28162 536 402 19 4.726368159 1.058368993 183|1282|1906|2263|2697|3371|3488|3627|4321|4478|5268|6356|7052|7474|10979|26585|94234|115908|284217 AGT|COL4A1|EDN1|FGFR2|GJA1|TNC|IGFBP5|CXCL10|MMP12|MSN|SERPINB5|CCL11|TGM2|WNT5A|FERMT2|GREM1|FOXQ1|CTHRC1|LAMA1 -1.911503255 0 64 0 0 -5.311900384 12.36259877
1 -3.44045779 GO:0001763 M1 1 0 GO Biological Processes 19 morphogenesis of

a branching structure	-3.44045779	3.766383138	4.555156717	28162	186	402	10	2.487562189	0.776790586	183|1282|1906|2263|3371|6356|7052|7474|26585|284217	AGT|COL4A1|EDN1|FGFR2|TNC|CCL11|TGM2|WNT5A|GREM1|LAMA1	-1.839856959	0	64	0	0	-5.311900384	12.36259877

1	-3.256901045	GO:0060512	M1	1	0	GO Biological Processes	19	prostate gland morphogenesis	-3.256901045	10.37847798	5.867027412	28162	27	402	4	0.995024876	0.495031064	2263|3371|5268|7474	FGFR2|TNC|SERPINB5|WNT5A	-1.684862738	0	64	0	0	-5.311900384	12.36259877

1	-3.021064796	GO:0048729	M1	1	0	GO Biological Processes	19	tissue morphogenesis	-3.021064796	2.196072927	3.677388867	28162	638	402	20	4.975124378	1.084445172	183|1282|1906|2263|2697|3371|3488|3627|4321|4478|5268|6091|6356|7052|7474|10979|26585|94234|115908|284217	AGT|COL4A1|EDN1|FGFR2|GJA1|TNC|IGFBP5|CXCL10|MMP12|MSN|SERPINB5|ROBO1|CCL11|TGM2|WNT5A|FERMT2|GREM1|FOXQ1|CTHRC1|LAMA1	-1.49761169	0	64	0	0	-5.311900384	12.36259877

1	-2.771670055	GO:0060602	M1	1	0	GO Biological Processes	19	branch elongation of an epithelium	-2.771670055	12.36259877	5.639347249	28162	17	402	3	0.746268657	0.429247719	2263|3371|7474	FGFR2|TNC|WNT5A	-1.303326622	0	64	0	0	-5.311900384	12.36259877

1	-2.748284387	GO:1905330	M1	1	0	GO Biological Processes	19	regulation of morphogenesis of an epithelium	-2.748284387	5.742190686	4.461802836	28162	61	402	5	1.243781095	0.552765814	183|2697|3627|7474|26585	AGT|GJA1|CXCL10|WNT5A|GREM1	-1.284500518	0	64	0	0	-5.311900384	12.36259877

1	-2.561047129	GO:0060445	M1	1	0	GO Biological Processes	19	branching involved in salivary gland morphogenesis	-2.561047129	10.50820896	5.118745859	28162	20	402	3	0.746268657	0.429247719	2263|7052|284217	FGFR2|TGM2|LAMA1	-1.137313776	0	64	0	0	-5.311900384	12.36259877

1	-2.545627889	GO:0048736	M1	1	0	GO Biological Processes	19	appendage development	-2.545627889	3.355915036	3.67455672	28162	167	402	8	1.990049751	0.696552772	2263|4038|5308|5396|7474|10979|23657|26585	FGFR2|LRP4|PITX2|PRRX1|WNT5A|FERMT2|SLC7A11|GREM1	-1.124462116	0	64	0	0	-5.311900384	12.36259877

1	-2.545627889	GO:0060173	M1	1	0	GO Biological Processes	19	limb development	-2.545627889	3.355915036	3.67455672	28162	167	402	8	1.990049751	0.696552772	2263|4038|5308|5396|7474|10979|23657|26585	FGFR2|LRP4|PITX2|PRRX1|WNT5A|FERMT2|SLC7A11|GREM1	-1.124462116	0	64	0	0	-5.311900384	12.36259877

1	-2.498684669	GO:0002053	M1	1	0	GO Biological Processes	19	positive regulation of mesenchymal cell proliferation	-2.498684669	10.00781805	4.969203948	28162	21	402	3	0.746268657	0.429247719	2263|5396|7474	FGFR2|PRRX1|WNT5A	-1.087688138	0	64	0	0	-5.311900384	12.36259877

1	-2.314184917	GO:0060688	M1	1	0	GO Biological Processes	19	regulation of

morphogenesis of a branching structure -2.314184917 5.837893864 4.036853294 28162 48 402 4 0.995024876 0.495031064 183|2263|7474|26585 AGT|FGFR2|WNT5A|GREM1 -0.947537787 0 64 0 0 -5.311900384 12.36259877
1 -2.288029846 GO:0060562 M1 1 0 GO Biological Processes 19 epithelial tube morphogenesis -2.288029846 2.493857573 3.177457681 28162 309 402 11 2.736318408 0.813665012 183|1282|1906|2263|3371|3627|6356|7474|26585|115908|284217 AGT|COL4A1|EDN1|FGFR2|TNC|CXCL10|CCL11|WNT5A|GREM1|CTHRC1|LAMA1 -0.926340985 0 64 0 0 -5.311900384 12.36259877
1 -2.279112201 GO:0060740 M1 1 0 GO Biological Processes 19 prostate gland epithelium morphogenesis -2.279112201 8.406567164 4.458362801 28162 25 402 3 0.746268657 0.429247719 2263|3371|7474 FGFR2|TNC|WNT5A -0.923653081 0 64 0 0 -5.311900384 12.36259877
1 -2.230434181 GO:0010464 M1 1 0 GO Biological Processes 19 regulation of mesenchymal cell proliferation -2.230434181 8.083237658 4.348251364 28162 26 402 3 0.746268657 0.429247719 2263|5396|7474 FGFR2|PRRX1|WNT5A -0.882189587 0 64 0 0 -5.311900384 12.36259877
1 -2.183845757 GO:0003401 M1 1 0 GO Biological Processes 19 axis elongation -2.183845757 7.783858485 4.243874515 28162 27 402 3 0.746268657 0.429247719 2263|3371|7474 FGFR2|TNC|WNT5A -0.847370775 0 64 0 0 -5.311900384 12.36259877
1 -2.183845757 GO:0021884 M1 1 0 GO Biological Processes 19 forebrain neuron development -2.183845757 7.783858485 4.243874515 28162 27 402 3 0.746268657 0.429247719 2263|6091|7474 FGFR2|ROBO1|WNT5A -0.847370775 0 64 0 0 -5.311900384 12.36259877
1 -2.183845757 GO:0033688 M1 1 0 GO Biological Processes 19 regulation of osteoblast proliferation -2.183845757 7.783858485 4.243874515 28162 27 402 3 0.746268657 0.429247719 2263|26585|115908 FGFR2|GREM1|CTHRC1 -0.847370775 0 64 0 0 -5.311900384 12.36259877
1 -2.183845757 GO:0060441 M1 1 0 GO Biological Processes 19 epithelial tube branching involved in lung morphogenesis -2.183845757 7.783858485 4.243874515 28162 27 402 3 0.746268657 0.429247719 2263|3371|284217 FGFR2|TNC|LAMA1 -0.847370775 0 64 0 0 -5.311900384 12.36259877
1 -2.139188431 GO:0060603 M1 1 0 GO Biological Processes 19 mammary gland duct morphogenesis -2.139188431 7.505863539 4.144723426 28162 28 402 3 0.746268657 0.429247719 2263|6356|7474 FGFR2|CCL11|WNT5A -0.810292083 0 64 0 0 -5.311900384 12.36259877
1 -2.096321068 GO:0007435 M1 1 0 GO Biological Processes 19 salivary gland morphogenesis -2.096321068 7.247040659 4.050350813 28162 29 402 3 0.746268657 0.429247719 2263|7052|284217 FGFR2|TGM2|LAMA1 -0.778908152 0 64 0 0 -5.311900384 12.36259877
1 -2.057795497 GO:0090596 M1 1 0 GO Biological Processes 19 sensory organ morphogenesis -2.057795497 2.583985809 2.990414432 28162 244 402 9 2.23880597 0.737867616 1290|1906|2263|5308|5396|6505|7068|7474|115908

COL5A2|EDN1|FGFR2|PITX2|PRRX1|SLC1A1|THRB|WNT5A|CTHRC1 -0.74572497 0 64 0 0 -5.311900384 12.36259877

1 -2.015464226 GO:0007431 M1 1 0 GO Biological Processes 19 salivary gland development -2.015464226 6.779489649 3.8744056 28162 31 402 3 0.746268657 0.429247719 2263|7052|284217 FGFR2|TGM2|LAMA1 -0.713946752 0 64 0 0 -5.311900384 12.36259877

1 -5.268010822 GO:0042044 M1 1 0 GO Biological Processes 19 fluid transport -5.268010822 13.13526119 8.265451528 28162 32 402 6 1.492537313 0.604761504 343|360|366|1906|6338|282679AQP8|AQP3|AQP9|EDN1|SCNN1B|AQP11 -3.333297947 0 65 1 1 -5.268010822 42.03283582

1 -4.578220964 GO:0015250 M1 1 0 GO Molecular Functions 21 water channel activity -4.578220964 21.55530042 8.920544234 28162 13 402 4 0.995024876 0.495031064 343|360|366|282679 AQP8|AQP3|AQP9|AQP11 -2.755253132 0 65 0 0 -5.268010822 42.03283582

1 -4.548790617 GO:0015254 M1 1 0 GO Molecular Functions 21 glycerol channel activity -4.548790617 42.03283582 11.04207458 28162 5 402 3 0.746268657 0.429247719 360|366|282679 AQP3|AQP9|AQP11 -2.738597074 0 65 0 0 -5.268010822 42.03283582

1 -4.307229296 GO:0005372 M1 1 0 GO Molecular Functions 21 water transmembrane transporter activity -4.307229296 18.68126036 8.24271197 28162 15 402 4 0.995024876 0.495031064 343|360|366|282679 AQP8|AQP3|AQP9|AQP11 -2.540141634 0 65 0 0 -5.268010822 42.03283582

1 -4.168434275 GO:1901618 M1 1 0 GO Molecular Functions 21 organic hydroxy compound transmembrane transporter activity -4.168434275 8.578129759 6.389013606 28162 49 402 6 1.492537313 0.604761504 360|366|6566|123264|200931|282679 AQP3|AQP9|SLC16A1|SLC51B|SLC51A|AQP11 -2.42356601 0 65 0 0 -5.268010822 42.03283582

1 -4.013986523 GO:0015168 M1 1 0 GO Molecular Functions 21 glycerol transmembrane transporter activity -4.013986523 30.02345416 9.241610993 28162 7 402 3 0.746268657 0.429247719 360|366|282679 AQP3|AQP9|AQP11 -2.305460374 0 65 0 0 -5.268010822 42.03283582

1 -4.013986523 GO:0015793 M1 1 0 GO Biological Processes 19 glycerol transport -4.013986523 30.02345416 9.241610993 28162 7 402 3 0.746268657 0.429247719 360|366|282679 AQP3|AQP9|AQP11 -2.305460374 0 65 0 0 -5.268010822 42.03283582

1 -3.806116813 GO:0015850 M1 1 0 GO Biological Processes 19 organic hydroxy compound transport -3.806116813 3.360558829 4.698884422 28162 271 402 13 3.233830846 0.882281956 183|360|366|857|5028|6566|6696|9388|54762|114876|123264|200931|282679 AGT|AQP3|AQP9|CAV1|P2RY1|SLC16A1|SPP1|LIPG|GRAMD1C|OSBPL1A|SLC51B|SLC51A |AQP11 -2.130202032 0 65 0 0 -5.268010822 42.03283582

1 -3.643026298 GO:0015840 M1 1 0 GO Biological Processes 19 urea transport -3.643026298 23.35157546 8.070387472 28162 9 402 3 0.746268657 0.429247719 360|366|282679 AQP3|AQP9|AQP11 -1.994851385 0 65 0 0

-5.268010822 42.03283582
1 -3.612497705 GO:0006833 M1 1 0 GO Biological Processes 19 water transport -3.612497705 12.73722298 6.627381649 28162 22 402 4 0.995024876 0.495031064 343|360|366|282679 AQP8|AQP3|AQP9|AQP11 -1.972814426 0 65 0 0 -5.268010822 42.03283582
1 -3.359059655 GO:0015166 M1 1 0 GO Molecular Functions 21 polyol transmembrane transporter activity -3.359059655 19.10583446 7.227619926 28162 11 402 3 0.746268657 0.429247719 360|366|282679 AQP3|AQP9|AQP11 -1.767683125 0 65 0 0 -5.268010822 42.03283582
1 -3.23873503 GO:0015791 M1 1 0 GO Biological Processes 19 polyol transport -3.23873503 17.51368159 6.885296737 28162 12 402 3 0.746268657 0.429247719 360|366|282679 AQP3|AQP9|AQP11 -1.672477766 0 65 0 0 -5.268010822 42.03283582
1 -3.029274715 GO:0019755 M1 1 0 GO Biological Processes 19 one-carbon compound transport -3.029274715 15.01172708 6.31043428 28162 14 402 3 0.746268657 0.429247719 360|366|282679 AQP3|AQP9|AQP11 -1.500806012 0 65 0 0 -5.268010822 42.03283582
1 -2.686650474 GO:0015144 M1 1 0 GO Molecular Functions 21 carbohydrate transmembrane transporter activity -2.686650474 7.374181723 4.73156735 28162 38 402 4 0.995024876 0.495031064 360|366|9963|282679 AQP3|AQP9|SLC23A1|AQP11 -1.237911023 0 65 0 0 -5.268010822 42.03283582
1 -2.380245845 GO:0015267 M1 1 0 GO Molecular Functions 21 channel activity -2.380245845 2.184658826 3.153568559 28162 481 402 15 3.731343284 0.945283402 343|360|366|1134|2555|2697|3752|6338|10008|22802|55151|140738|140803|255231|282679 AQP8|AQP3|AQP9|CHRNA1|GABRA2|GJA1|KCND3|SCNN1B|KCNE3|CLCA4|TMEM38B|TMEM37|TRPM6|MCOLN2|AQP11 -0.998844193 0 65 0 0 -5.268010822 42.03283582
1 -2.372037506 GO:0022803 M1 1 0 GO Molecular Functions 21 passive transmembrane transporter activity -2.372037506 2.180126339 3.144823757 28162 482 402 15 3.731343284 0.945283402 343|360|366|1134|2555|2697|3752|6338|10008|22802|55151|140738|140803|255231|282679 AQP8|AQP3|AQP9|CHRNA1|GABRA2|GJA1|KCND3|SCNN1B|KCNE3|CLCA4|TMEM38B|TMEM37|TRPM6|MCOLN2|AQP11 -0.992507816 0 65 0 0 -5.268010822 42.03283582
1 -2.260201294 GO:0030104 M1 1 0 GO Biological Processes 19 water homeostasis -2.260201294 4.433843441 3.67789747 28162 79 402 5 1.243781095 0.552765814 360|366|6338|9076|282679 AQP3|AQP9|SCNN1B|CLDN1|AQP11 -0.90826231 0 65 0 0 -5.268010822 42.03283582
1 -2.07688532 GO:0042887 M1 1 0 GO Molecular Functions 21 amide transmembrane transporter activity -2.07688532 5.003909026 3.609159088 28162 56 402 4 0.995024876 0.495031064 360|366|2697|9429

AQP3|AQP9|GJA1|ABCG2 -0.762296437 0 65 0 0 -5.268010822 42.03283582
1 -5.158324907 GO:0098662 M1 1 0 GO Biological Processes 19 inorganic cation transmembrane transport -5.158324907 2.550757267 5.24704185 28162 769 402 28 6.965174129 1.269625794
288|857|1908|2040|3627|3752|4067|4283|6338|6373|6533|6550|6947|8671|9963|10008|10050|11254|55151|55532|57214|57628|140738|140803|159371|206358|255231|340024
ANK3|CAV1|EDN3|STOM|CXCL10|KCND3|LYN|CXCL9|SCNN1B|CXCL11|SLC6A6|SLC9A3|TCN1|SLC4A4|SLC23A1|KCNE3|SLC17A4|SLC6A14|TMEM38B|SLC30A10|CEMIP|DPP10|TMEM37|TRPM6|SLC35G1|SLC36A1|MCOLN2|SLC6A19 -3.244881172 0 66 1 1 -5.158324907 7.247040659
1 -5.006218062 GO:0006814 M1 1 0 GO Biological Processes 19 sodium ion transport -5.006218062 4.103624139 5.798482693 28162 239 402 14 3.482587065 0.914409651
288|2040|5270|6338|6533|6550|6584|8671|9963|10050|11254|55089|154043|340024
ANK3|STOM|SERPINE2|SCNN1B|SLC6A6|SLC9A3|SLC22A5|SLC4A4|SLC23A1|SLC17A4|SLC6A14|SLC38A4|CNKSR3|SLC6A19 -3.113050284 0 66 0 0 -5.158324907 7.247040659
1 -3.733513085 GO:0035725 M1 1 0 GO Biological Processes 19 sodium ion transmembrane transport -3.733513085 4.096767624 4.887927976 28162 171 402 10 2.487562189 0.776790586
288|2040|6338|6533|6550|8671|9963|10050|11254|340024
ANK3|STOM|SCNN1B|SLC6A6|SLC9A3|SLC4A4|SLC23A1|SLC17A4|SLC6A14|SLC6A19 -2.064928343 0 66 0 0 -5.158324907 7.247040659
1 -3.087470254 GO:0008324 M1 1 0 GO Molecular Functions 21 cation transmembrane transporter activity -3.087470254 2.223959567 3.73891677 28162 630 402 20 4.975124378 1.084445172
1134|3752|6338|6505|6533|6550|6584|8140|8671|9429|9963|10008|10050|11254|55151|55532|140738|140803|206358|255231
CHRNA1|KCND3|SCNN1B|SLC1A1|SLC6A6|SLC9A3|SLC22A5|SLC7A5|SLC4A4|ABCG2|SLC23A1|KCNE3|SLC17A4|SLC6A14|TMEM38B|SLC30A10|TMEM37|TRPM6|SLC36A1|MCOLN2 -1.541220869 0 66 0 0 -5.158324907 7.247040659
1 -3.078955437 GO:0022853 M1 1 0 GO Molecular Functions 21 active ion transmembrane transporter activity -3.078955437 3.384286298 4.143250194 28162 207 402 10 2.487562189 0.776790586
1836|6505|6533|6550|8671|9963|10050|23657|28231|206358
SLC26A2|SLC1A1|SLC6A6|SLC9A3|SLC4A4|SLC23A1|SLC17A4|SLC7A11|SLCO4A1|SLC36A1 -1.537522191 0 66 0 0 -5.158324907 7.247040659
1 -2.523609777 GO:0015294 M1 1 0 GO Molecular Functions 21 solute:cation symporter activity -2.523609777 4.245740992 3.893065777 28162 99 402 6 1.492537313 0.604761504 6505|6533|8671|9963|10050|206358
SLC1A1|SLC6A6|SLC4A4|SLC23A1|SLC17A4|SLC36A1 -1.108577961 0 66 0 0 -5.158324907 7.247040659
1 -2.454105557 GO:0046873 M1 1 0 GO Molecular Functions 21 metal ion transmembrane transporter activity -2.454105557 2.302268003 3.259196814 28162

426 402 14 3.482587065 0.914409651
3752|6338|6505|6533|6550|8671|9963|10008|10050|55151|55532|140738|140803|255231
KCND3|SCNN1B|SLC1A1|SLC6A6|SLC9A3|SLC4A4|SLC23A1|KCNE3|SLC17A4|TMEM38B|SLC30A10|TMEM37|TRPM6|MCOLN2 -1.056457773 0 66 0 0 -5.158324907 7.247040659
1 -2.432171716 GO:0015370 M1 1 0 GO Molecular Functions 21 solute:sodium symporter activity -2.432171716 4.864911553 3.951457091 28162 72 402 5 1.243781095 0.552765814 6505|6533|8671|9963|10050
SLC1A1|SLC6A6|SLC4A4|SLC23A1|SLC17A4 -1.041287275 0 66 0 0 -5.158324907 7.247040659
1 -2.291825379 GO:0015081 M1 1 0 GO Molecular Functions 21 sodium ion transmembrane transporter activity -2.291825379 3.358788251 3.438663282 28162 146 402 7 1.741293532 0.652391792 6338|6505|6533|6550|8671|9963|10050
SCNN1B|SLC1A1|SLC6A6|SLC9A3|SLC4A4|SLC23A1|SLC17A4 -0.929240126 0 66 0 0 -5.158324907 7.247040659
1 -2.096321068 GO:0005343 M1 1 0 GO Molecular Functions 21 organic acid:sodium symporter activity -2.096321068 7.247040659 4.050350813 28162 29 402 3 0.746268657 0.429247719 6505|6533|9963 SLC1A1|SLC6A6|SLC23A1 -0.778908152 0 66 0 0 -5.158324907 7.247040659
1 -5.014242659 GO:0008289 M1 1 0 GO Molecular Functions 21 lipid binding -5.014242659 2.505149857 5.140189899 28162 783 402 28 6.965174129 1.269625794
51|301|308|857|1604|2869|4067|5320|5468|6279|6280|6402|7057|7474|7873|9912|10979|22822|23596|26207|27071|54762|54852|55198|57758|85477|114876|284217
ACOX1|ANXA1|ANXA5|CAV1|CD55|GRK5|LYN|PLA2G2A|PPARG|S100A8|S100A9|SELL|THBS1|WNT5A|MANF|ARHGAP44|FERMT2|PHLDA1|OPN3|PITPNC1|DAPP1|GRAMD1C|PAQR5|APPL2|SCUBE2|SCIN|OSBPL1A|LAMA1 -3.114087232 0 67 1 1 -5.014242659 2.505149857
1 -2.223325776 GO:0005543 M1 1 0 GO Molecular Functions 21 phospholipid binding -2.223325776 2.165046731 3.008218323 28162 453 402 14 3.482587065 0.914409651
301|308|2869|5320|7057|7474|9912|10979|22822|26207|27071|55198|85477|114876
ANXA1|ANXA5|GRK5|PLA2G2A|THBS1|WNT5A|ARHGAP44|FERMT2|PHLDA1|PITPNC1|DAPP1|APPL2|SCIN|OSBPL1A -0.876598754 0 67 0 0 -5.014242659 2.505149857
1 -5.007691967 GO:0043588 M1 1 0 GO Biological Processes 19 skin development -5.007691967 3.207324822 5.451419587 28162 415 402 19 4.726368159 1.058368993
301|360|1001|1277|1278|1281|1290|1513|2263|3488|4038|5068|5266|7474|9076|11005|23596|94234|147495
ANXA1|AQP3|CDH3|COL1A1|COL1A2|COL3A1|COL5A2|CTSK|FGFR2|IGFBP5|LRP4|REG3A|PI3|WNT5A|CLDN1|SPINK5|OPN3|FOXQ1|APCDD1 -3.113050284 0 68 1 1 -5.007691967 7.783858485
1 -4.720838049 GO:0001942 M1 1 0 GO Biological Processes 19 hair follicle

development -4.720838049 7.005472637 6.472998411 28162 80 402 8
1.990049751 0.696552772 1001|2263|3488|4038|7474|11005|94234|147495
CDH3|FGFR2|IGFBP5|LRP4|WNT5A|SPINK5|FOXQ1|APCDD1 -2.872512494 0 68 0
0 -5.007691967 7.783858485
1 -4.603219892 GO:0022404 M1 1 0 GO Biological Processes 19 molting cycle process -4.603219892 6.752262782 6.315595077 28162 83 402 8 1.990049751
0.696552772 1001|2263|3488|4038|7474|11005|94234|147495
CDH3|FGFR2|IGFBP5|LRP4|WNT5A|SPINK5|FOXQ1|APCDD1 -2.771081098 0 68 0
0 -5.007691967 7.783858485
1 -4.603219892 GO:0022405 M1 1 0 GO Biological Processes 19 hair cycle process
-4.603219892 6.752262782 6.315595077 28162 83 402 8 1.990049751
0.696552772 1001|2263|3488|4038|7474|11005|94234|147495
CDH3|FGFR2|IGFBP5|LRP4|WNT5A|SPINK5|FOXQ1|APCDD1 -2.771081098 0 68 0
0 -5.007691967 7.783858485
1 -4.565133515 GO:0098773 M1 1 0 GO Biological Processes 19 skin epidermis development -4.565133515 6.671878702 6.264852128 28162 84 402 8
1.990049751 0.696552772 1001|2263|3488|4038|7474|11005|94234|147495
CDH3|FGFR2|IGFBP5|LRP4|WNT5A|SPINK5|FOXQ1|APCDD1 -2.743460154 0 68 0
0 -5.007691967 7.783858485
1 -3.872017831 GO:0042303 M1 1 0 GO Biological Processes 19 molting cycle
-3.872017831 5.337502961 5.35847427 28162 105 402 8 1.990049751
0.696552772 1001|2263|3488|4038|7474|11005|94234|147495
CDH3|FGFR2|IGFBP5|LRP4|WNT5A|SPINK5|FOXQ1|APCDD1 -2.182970035 0 68 0
0 -5.007691967 7.783858485
1 -3.872017831 GO:0042633 M1 1 0 GO Biological Processes 19 hair cycle
-3.872017831 5.337502961 5.35847427 28162 105 402 8 1.990049751
0.696552772 1001|2263|3488|4038|7474|11005|94234|147495
CDH3|FGFR2|IGFBP5|LRP4|WNT5A|SPINK5|FOXQ1|APCDD1 -2.182970035 0 68 0
0 -5.007691967 7.783858485
1 -2.533004508 GO:0008544 M1 1 0 GO Biological Processes 19 epidermis development -2.533004508 2.269591567 3.314742612 28162 463 402 15
3.731343284 0.945283402
301|360|1001|1513|2263|3488|4038|5068|5266|5308|7474|11005|23596|94234|147495
ANXA1|AQP3|CDH3|CTSK|FGFR2|IGFBP5|LRP4|REG3A|PI3|PITX2|WNT5A|SPINK5|OPN3|FOXQ1|APCDD1 -1.113387721 0 68 0 0 -5.007691967 7.783858485
1 -2.183845757 GO:0031069 M1 1 0 GO Biological Processes 19 hair follicle morphogenesis -2.183845757 7.783858485 4.243874515 28162 27 402 3
0.746268657 0.429247719 2263|3488|94234 FGFR2|IGFBP5|FOXQ1 -0.847370775 0
68 0 0 -5.007691967 7.783858485
1 -2.015464226 GO:0048730 M1 1 0 GO Biological Processes 19 epidermis morphogenesis -2.015464226 6.779489649 3.8744056 28162 31 402 3
0.746268657 0.429247719 2263|3488|94234 FGFR2|IGFBP5|FOXQ1 -0.713946752 0
68 0 0 -5.007691967 7.783858485

1 -4.950201889 GO:0098742 M1 1 0 GO Biological Processes 19 cell-cell adhesion via plasma-membrane adhesion molecules -4.950201889 3.821166893 5.657460874 28162 275 402 15 3.731343284 0.945283402 682|1001|1009|3557|5068|5175|5801|6091|6402|9073|9076|23705|53841|57126|137075 BSG|CDH3|CDH11|IL1RN|REG3A|PECAM1|PTPRR|ROBO1|SELL|CLDN8|CLDN1|CADM1|CDHR5|CD177|CLDN23 -3.061581738 0 69 1 1 -4.950201889 3.821166893

1 -2.513977917 GO:0007156 M1 1 0 GO Biological Processes 19 homophilic cell adhesion via plasma membrane adhesion molecules -2.513977917 3.316200065 3.634310523 28162 169 402 8 1.990049751 0.696552772 682|1001|1009|5175|5801|6091|23705|53841 BSG|CDH3|CDH11|PECAM1|PTPRR|ROBO1|CADM1|CDHR5 -1.099452567 0 69 0 0 -4.950201889 3.821166893

1 -4.904400767 GO:0060343 M1 1 0 GO Biological Processes 19 trabecula formation -4.904400767 15.92152872 8.425388682 28162 22 402 5 1.243781095 0.552765814 1277|4313|9510|26585|57211 COL1A1|MMP2|ADAMTS1|GREM1|ADGRG6 -3.025978422 0 70 1 1 -4.904400767 23.35157546

1 -3.643026298 GO:0060346 M1 1 0 GO Biological Processes 19 bone trabecula formation -3.643026298 23.35157546 8.070387472 28162 9 402 3 0.746268657 0.429247719 1277|4313|26585 COL1A1|MMP2|GREM1 -1.994851385 0 70 0 0 -4.904400767 23.35157546

1 -3.44832504 GO:0061383 M1 1 0 GO Biological Processes 19 trabecula morphogenesis -3.44832504 8.145898415 5.643109297 28162 43 402 5 1.243781095 0.552765814 1277|4313|9510|26585|57211 COL1A1|MMP2|ADAMTS1|GREM1|ADGRG6 -1.844605385 0 70 0 0 -4.904400767 23.35157546

1 -3.23873503 GO:0061430 M1 1 0 GO Biological Processes 19 bone trabecula morphogenesis -3.23873503 17.51368159 6.885296737 28162 12 402 3 0.746268657 0.429247719 1277|4313|26585 COL1A1|MMP2|GREM1 -1.672477766 0 70 0 0 -4.904400767 23.35157546

1 -2.451174411 GO:0060324 M1 1 0 GO Biological Processes 19 face development -2.451174411 6.368611488 4.288677933 28162 44 402 4 0.995024876 0.495031064 1277|4313|7474|83716COL1A1|MMP2|WNT5A|CRISPLD2 -1.054984808 0 70 0 0 -4.904400767 23.35157546

1 -2.096321068 GO:0060325 M1 1 0 GO Biological Processes 19 face morphogenesis -2.096321068 7.247040659 4.050350813 28162 29 402 3 0.746268657 0.429247719 1277|4313|83716 COL1A1|MMP2|CRISPLD2 -0.778908152 0 70 0 0 -4.904400767 23.35157546

1 -4.733000928 GO:0043455 M1 1 0 GO Biological Processes 19 regulation of secondary metabolic process -4.733000928 23.35157546 9.319377311 28162 12 402 4 0.995024876 0.495031064 1001|7474|23596|23657 CDH3|WNT5A|OPN3|SLC7A11 -2.880532627 0 71 1 1 -4.733000928 30.02345416

1 -4.733000928 GO:0048021 M1 1 0 GO Biological Processes 19 regulation of melanin biosynthetic process -4.733000928 23.35157546 9.319377311 28162 12 402 4 0.995024876 0.495031064 1001|7474|23596|23657 CDH3|WNT5A|OPN3|SLC7A11 -2.880532627 0 71 0 0 -4.733000928 30.02345416
1 -4.733000928 GO:1900376 M1 1 0 GO Biological Processes 19 regulation of secondary metabolite biosynthetic process -4.733000928 23.35157546 9.319377311 28162 12 402 4 0.995024876 0.495031064 1001|7474|23596|23657 CDH3|WNT5A|OPN3|SLC7A11 -2.880532627 0 71 0 0 -4.733000928 30.02345416
1 -4.013986523 GO:0044851 M1 1 0 GO Biological Processes 19 hair cycle phase -4.013986523 30.02345416 9.241610993 28162 7 402 3 0.746268657 0.429247719 1001|7474|11005 CDH3|WNT5A|SPINK5 -2.305460374 0 71 0 0 -4.733000928 30.02345416
1 -4.013986523 GO:0048819 M1 1 0 GO Biological Processes 19 regulation of hair follicle maturation -4.013986523 30.02345416 9.241610993 28162 7 402 3 0.746268657 0.429247719 1001|7474|11005 CDH3|WNT5A|SPINK5 -2.305460374 0 71 0 0 -4.733000928 30.02345416
1 -3.694747652 GO:0042438 M1 1 0 GO Biological Processes 19 melanin biosynthetic process -3.694747652 13.3437574 6.809490119 28162 21 402 4 0.995024876 0.495031064 1001|7474|23596|23657 CDH3|WNT5A|OPN3|SLC7A11 -2.035154836 0 71 0 0 -4.733000928 30.02345416
1 -3.643026298 GO:0044848 M1 1 0 GO Biological Processes 19 biological phase -3.643026298 23.35157546 8.070387472 28162 9 402 3 0.746268657 0.429247719 1001|7474|11005 CDH3|WNT5A|SPINK5 -1.994851385 0 71 0 0 -4.733000928 30.02345416
1 -3.612497705 GO:0006582 M1 1 0 GO Biological Processes 19 melanin metabolic process -3.612497705 12.73722298 6.627381649 28162 22 402 4 0.995024876 0.495031064 1001|7474|23596|23657 CDH3|WNT5A|OPN3|SLC7A11 -1.972814426 0 71 0 0 -4.733000928 30.02345416
1 -3.534421373 GO:0044550 M1 1 0 GO Biological Processes 19 secondary metabolite biosynthetic process -3.534421373 12.18343067 6.456720249 28162 23 402 4 0.995024876 0.495031064 1001|7474|23596|23657 CDH3|WNT5A|OPN3|SLC7A11 -1.911503255 0 71 0 0 -4.733000928 30.02345416
1 -3.349455065 GO:1901615 M1 1 0 GO Biological Processes 19 organic hydroxy compound metabolic process -3.349455065 2.398269912 4.003641925 28162 555 402 19 4.726368159 1.058368993 126|1001|1513|2053|2330|3158|5028|5105|7436|7474|23596|23657|27163|29968|50506|79644|114876|195814|405753 ADH1C|CDH3|CTSK|EPHX2|FMO5|HMGCS2|P2RY1|PCK1|VLDLR|WNT5A|OPN3|SLC7A11|NAAA|PSAT1|DUOX2|SRD5A3|OSBPL1A|SDR16C5|DUOXA2 -1.758838455 0 71 0 0 -4.733000928 30.02345416
1 -3.23873503 GO:0048820 M1 1 0 GO Biological Processes 19 hair follicle

maturation -3.23873503 17.51368159 6.885296737 28162 12 402 3
0.746268657 0.429247719 1001|7474|11005 CDH3|WNT5A|SPINK5 -1.672477766 0
71 0 0 -4.733000928 30.02345416
1 -3.080013373 GO:0018958 M1 1 0 GO Biological Processes 19 phenol-containing compound metabolic process -3.080013373 4.626255515 4.501173884 28162 106 402
7 1.741293532 0.652391792 1001|1513|7474|23596|23657|50506|405753
CDH3|CTSK|WNT5A|OPN3|SLC7A11|DUOX2|DUOXA2 -1.537522191 0 71 0 0
-4.733000928 30.02345416
1 -2.487719498 GO:0046189 M1 1 0 GO Biological Processes 19 phenol-containing compound biosynthetic process -2.487719498 6.516718732 4.356547625 28162 43
402 4 0.995024876 0.495031064 1001|7474|23596|23657
CDH3|WNT5A|OPN3|SLC7A11 -1.080223345 0 71 0 0 -4.733000928
30.02345416
1 -2.462920832 GO:1901617 M1 1 0 GO Biological Processes 19 organic hydroxy compound biosynthetic process -2.462920832 2.791024955 3.429873726 28162 251
402 10 2.487562189 0.776790586
1001|3158|5028|5105|7474|23596|23657|29968|79644|114876
CDH3|HMGCS2|P2RY1|PCK1|WNT5A|OPN3|SLC7A11|PSAT1|SRD5A3|OSBPL1A
-1.062341915 0 71 0 0 -4.733000928 30.02345416
1 -2.40637353 GO:0042440 M1 1 0 GO Biological Processes 19 pigment metabolic process -2.40637353 4.798268929 3.910266289 28162 73 402 5
1.243781095 0.552765814 1001|7474|10891|23596|23657
CDH3|WNT5A|PPARGC1A|OPN3|SLC7A11 -1.021203573 0 71 0 0
-4.733000928 30.02345416
1 -2.367431958 GO:0071695 M1 1 0 GO Biological Processes 19 anatomical structure maturation -2.367431958 2.892167602 3.374977599 28162 218 402 9
2.23880597 0.737867616 718|994|1001|1672|4313|5468|7474|11005|26585
C3|CDC25B|CDH3|DEFB1|MMP2|PPARG|WNT5A|SPINK5|GREM1 -0.988835232 0 71
0 0 -4.733000928 30.02345416
1 -2.07688532 GO:0019748 M1 1 0 GO Biological Processes 19 secondary metabolic process -2.07688532 5.003909026 3.609159088 28162 56 402 4
0.995024876 0.495031064 1001|7474|23596|23657 CDH3|WNT5A|OPN3|SLC7A11
-0.762296437 0 71 0 0 -4.733000928 30.02345416
1 -2.023879618 GO:0046148 M1 1 0 GO Biological Processes 19 pigment biosynthetic process -2.023879618 4.831360439 3.514878126 28162 58 402 4
0.995024876 0.495031064 1001|7474|23596|23657 CDH3|WNT5A|OPN3|SLC7A11
-0.719622106 0 71 0 0 -4.733000928 30.02345416
1 -4.720838049 GO:2000106 M1 1 0 GO Biological Processes 19 regulation of leukocyte apoptotic process -4.720838049 7.005472637 6.472998411 28162 80 402
8 1.990049751 0.696552772 301|1942|3575|3620|4067|7474|23657|29126
ANXA1|EFNA1|IL7R|IDO1|LYN|WNT5A|SLC7A11|CD274 -2.872512494 0 72 1 1
-4.720838049 16.16647532
1 -4.527234624 GO:2000108 M1 1 0 GO Biological Processes 19 positive regulation of leukocyte apoptotic process -4.527234624 13.47206276 7.656338321 28162 26

402 5 1.243781095 0.552765814 301|3620|4067|7474|29126
ANXA1|IDO1|LYN|WNT5A|CD274-2.72080123 0 72 0 0 -4.720838049
16.16647532
1 -3.960224808 GO:0071887 M1 1 0 GO Biological Processes 19 leukocyte apoptotic process -3.960224808 5.494488343 5.472223702 28162 102 402 8
1.990049751 0.696552772 301|1942|3575|3620|4067|7474|23657|29126
ANXA1|EFNA1|IL7R|IDO1|LYN|WNT5A|SLC7A11|CD274 -2.262519693 0 72 0 0
-4.720838049 16.16647532
1 -3.941624452 GO:0070232 M1 1 0 GO Biological Processes 19 regulation of T cell apoptotic process -3.941624452 10.30216564 6.531026297 28162 34 402 5
1.243781095 0.552765814 1942|3575|3620|7474|29126
EFNA1|IL7R|IDO1|WNT5A|CD274 -2.244889828 0 72 0 0 -4.720838049
16.16647532
1 -3.928037252 GO:0070228 M1 1 0 GO Biological Processes 19 regulation of lymphocyte apoptotic process -3.928037252 7.783858485 6.004626785 28162 54
402 6 1.492537313 0.604761504 1942|3575|3620|4067|7474|29126
EFNA1|IL7R|IDO1|LYN|WNT5A|CD274-2.234201155 0 72 0 0 -4.720838049
16.16647532
1 -3.272442991 GO:0070227 M1 1 0 GO Biological Processes 19 lymphocyte apoptotic process -3.272442991 5.920117721 4.995148461 28162 71 402 6
1.492537313 0.604761504 1942|3575|3620|4067|7474|29126
EFNA1|IL7R|IDO1|LYN|WNT5A|CD274-1.696017834 0 72 0 0 -4.720838049
16.16647532
1 -3.18183375 GO:0070231 M1 1 0 GO Biological Processes 19 T cell apoptotic process -3.18183375 7.148441466 5.183663705 28162 49 402 5 1.243781095
0.552765814 1942|3575|3620|7474|29126 EFNA1|IL7R|IDO1|WNT5A|CD274
-1.621281586 0 72 0 0 -4.720838049 16.16647532
1 -3.129402513 GO:0070234 M1 1 0 GO Biological Processes 19 positive regulation of T cell apoptotic process -3.129402513 16.16647532 6.581913723 28162 13 402
3 0.746268657 0.429247719 3620|7474|29126 IDO1|WNT5A|CD274
-1.577269892 0 72 0 0 -4.720838049 16.16647532
1 -2.85139329 GO:0070230 M1 1 0 GO Biological Processes 19 positive regulation of lymphocyte apoptotic process -2.85139329 13.13526119 5.842895378 28162 16
402 3 0.746268657 0.429247719 3620|7474|29126 IDO1|WNT5A|CD274
-1.363728798 0 72 0 0 -4.720838049 16.16647532
1 -2.782422644 GO:0070664 M1 1 0 GO Biological Processes 19 negative regulation of leukocyte proliferation -2.782422644 4.776458616 4.269738231 28162
88 402 6 1.492537313 0.604761504 3620|4067|4332|26585|29126|90865
IDO1|LYN|MNDA|GREM1|CD274|IL33 -1.305114044 0 72 0 0 -4.720838049
16.16647532
1 -2.614587017 GO:0050866 M1 1 0 GO Biological Processes 19 negative regulation of cell activation -2.614587017 3.152462687 3.676155264 28162 200 402
9 2.23880597 0.737867616 301|3598|3620|4067|4332|5270|7056|29126|55824
ANXA1|IL13RA2|IDO1|LYN|MNDA|SERPINE2|THBD|CD274|PAG1 -1.176664924 0 72

0 0 -4.720838049 16.16647532
1 -2.561047129 GO:0070233 M1 1 0 GO Biological Processes 19 negative regulation of T cell apoptotic process -2.561047129 10.50820896 5.118745859 28162 20 402 3 0.746268657 0.429247719 1942|3575|3620 EFNA1|IL7R|IDO1 -1.137313776 0 72 0 0 -4.720838049 16.16647532
1 -2.383476348 GO:0032693 M1 1 0 GO Biological Processes 19 negative regulation of interleukin-10 production -2.383476348 9.137573005 4.698203434 28162 23 402 3 0.746268657 0.429247719 3620|28951|29126 IDO1|TRIB2|CD274 -1.001605443 0 72 0 0 -4.720838049 16.16647532
1 -2.314184917 GO:0050798 M1 1 0 GO Biological Processes 19 activated T cell proliferation -2.314184917 5.837893864 4.036853294 28162 48 402 4 0.995024876 0.495031064 3620|11148|23705|29126 IDO1|HHLA2|CADM1|CD274 -0.947537787 0 72 0 0 -4.720838049 16.16647532
1 -2.139188431 GO:0002507 M1 1 0 GO Biological Processes 19 tolerance induction -2.139188431 7.505863539 4.144723426 28162 28 402 3 0.746268657 0.429247719 3620|4067|29126 IDO1|LYN|CD274 -0.810292083 0 72 0 0 -4.720838049 16.16647532
1 -2.015464226 GO:0070229 M1 1 0 GO Biological Processes 19 negative regulation of lymphocyte apoptotic process -2.015464226 6.779489649 3.8744056 28162 31 402 3 0.746268657 0.429247719 1942|3575|3620 EFNA1|IL7R|IDO1 -0.713946752 0 72 0 0 -4.720838049 16.16647532
1 -4.646840735 GO:0009897 M1 1 0 GO Cellular Components20 external side of plasma membrane -4.646840735 3.136778793 5.192663648 28162 402 402 18 4.47761194 1.031484687 301|762|2690|3575|3579|3598|3627|4162|4283|5243|5270|6338|7056|7057|8140|9429|11148|29126 ANXA1|CA4|GHR|IL7R|CXCR2|IL13RA2|CXCL10|MCAM|CXCL9|ABCB1|SERPINE2|SCNN1B|THBD|THBS1|SLC7A5|ABCG2|HHLA2|CD274 -2.805333129 0 73 1 1 -4.646840735 3.136778793
1 -3.647107297 GO:0098552 M1 1 0 GO Cellular Components20 side of membrane -3.647107297 2.399917217 4.217033046 28162 613 402 21 5.223880597 1.109770192 301|762|2690|2769|3575|3579|3598|3627|4067|4162|4283|5243|5270|6338|7056|7057|8140|9429|10979|11148|29126 ANXA1|CA4|GHR|GNA15|IL7R|CXCR2|IL13RA2|CXCL10|LYN|MCAM|CXCL9|ABCB1|SERPINE2|SCNN1B|THBD|THBS1|SLC7A5|ABCG2|FERMT2|HHLA2|CD274 -1.995451047 0 73 0 0 -4.646840735 3.136778793
1 -4.592740241 GO:0070820 M1 1 0 GO Cellular Components20 tertiary granule -4.592740241 4.698792622 5.716704371 28162 164 402 11 2.736318408 0.813665012 1604|2040|2357|2919|4318|5328|6947|7130|10562|22918|57126 CD55|STOM|FPR1|CXCL1|MMP9|PLAU|TCN1|TNFAIP6|OLFM4|CD93|CD177 -2.7619235 0 74 1 1 -4.592740241 6.368611488
1 -3.970462979 GO:0042581 M1 1 0 GO Cellular Components20 specific granule -3.970462979 4.378420398 5.157111108 28162 160 402 10 2.487562189

0.776790586 1116|2040|2919|3934|5328|6947|10562|10970|22918|57126 CHI3L1|STOM|CXCL1|LCN2|PLAU|TCN1|OLFM4|CKAP4|CD93|CD177 -2.269113421 0 74 0 0 -4.592740241 6.368611488

1 -2.951056812 GO:1904724 M1 1 0 GO Cellular Components20 tertiary granule lumen -2.951056812 6.368611488 4.795825872 28162 55 402 5 1.243781095 0.552765814 2919|4318|6947|7130|10562 CXCL1|MMP9|TCN1|TNFAIP6|OLFM4 -1.437464877 0 74 0 0 -4.592740241 6.368611488

1 -2.716804039 GO:0035580 M1 1 0 GO Cellular Components20 specific granule lumen -2.716804039 5.649574707 4.410453493 28162 62 402 5 1.243781095 0.552765814 1116|2919|3934|6947|10562 CHI3L1|CXCL1|LCN2|TCN1|OLFM4 -1.259212419 0 74 0 0 -4.592740241 6.368611488

1 -4.578220964 GO:0002921 M1 1 0 GO Biological Processes 19 negative regulation of humoral immune response -4.578220964 21.55530042 8.920544234 28162 13 402 4 0.995024876 0.495031064 722|725|1604|11005 C4BPA|C4BPB|CD55|SPINK5 -2.755253132 0 75 1 1 -4.578220964 42.03283582

1 -4.548790617 GO:0009608 M1 1 0 GO Biological Processes 19 response to symbiont -4.548790617 42.03283582 11.04207458 28162 5 402 3 0.746268657 0.429247719 722|725|6584C4BPA|C4BPB|SLC22A5 -2.738597074 0 75 0 0 -4.578220964 42.03283582

1 -4.548790617 GO:0009609 M1 1 0 GO Biological Processes 19 response to symbiotic bacterium -4.548790617 42.03283582 11.04207458 28162 5 402 3 0.746268657 0.429247719 722|725|6584C4BPA|C4BPB|SLC22A5 -2.738597074 0 75 0 0 -4.578220964 42.03283582

1 -3.643026298 GO:0045916 M1 1 0 GO Biological Processes 19 negative regulation of complement activation -3.643026298 23.35157546 8.070387472 28162 9 402 3 0.746268657 0.429247719 722|725|1604C4BPA|C4BPB|CD55 -1.994851385 0 75 0 0 -4.578220964 42.03283582

1 -3.062528266 GO:0002820 M1 1 0 GO Biological Processes 19 negative regulation of adaptive immune response -3.062528266 6.736031382 4.982073148 28162 52 402 5 1.243781095 0.552765814 722|725|3575|11126|90865 C4BPA|C4BPB|IL7R|CD160|IL33 -1.524748145 0 75 0 0 -4.578220964 42.03283582

1 -3.024546631 GO:0002704 M1 1 0 GO Biological Processes 19 negative regulation of leukocyte mediated immunity -3.024546631 6.60893645 4.918391422 28162 53 402 5 1.243781095 0.552765814 722|725|3575|3598|5272 C4BPA|C4BPB|IL7R|IL13RA2|SERPINB9 -1.499355638 0 75 0 0 -4.578220964 42.03283582

1 -2.415590886 GO:0002707 M1 1 0 GO Biological Processes 19 negative regulation of lymphocyte mediated immunity -2.415590886 6.227086788 4.222880671 28162 45 402 4 0.995024876 0.495031064 722|725|3575|5272 C4BPA|C4BPB|IL7R|SERPINB9 -1.027841704 0 75 0 0 -4.578220964 42.03283582

1 -2.347135782 GO:0002823 M1 1 0 GO Biological Processes 19 negative regulation of adaptive immune response based on somatic recombination of immune receptors

built from immunoglobulin superfamily domains -2.347135782 5.962104372 4.097067432 28162 47 402 4 0.995024876 0.495031064 722|725|3575|90865 C4BPA|C4BPB|IL7R|IL33 -0.971325982 0 75 0 0 -4.578220964 42.03283582

1 -4.578220964 GO:0060100 M1 1 0 GO Biological Processes 19 positive regulation of phagocytosis, engulfment -4.578220964 21.55530042 8.920544234 28162 13 402 4 0.995024876 0.495031064 718|5468|11031|55198C3|PPARG|RAB31|APPL2 -2.755253132 0 76 1 1 -4.578220964 21.55530042

1 -4.578220964 GO:1905155 M1 1 0 GO Biological Processes 19 positive regulation of membrane invagination -4.578220964 21.55530042 8.920544234 28162 13 402 4 0.995024876 0.495031064 718|5468|11031|55198C3|PPARG|RAB31|APPL2 -2.755253132 0 76 0 0 -4.578220964 21.55530042

1 -4.38784462 GO:0006909 M1 1 0 GO Biological Processes 19 phagocytosis -4.38784462 3.117618713 5.014650371 28162 382 402 17 4.228855721 1.003727321 301|718|722|725|2212|3553|4067|4688|5175|5468|7052|7057|10451|10562|11031|22918|55198 ANXA1|C3|C4BPA|C4BPB|FCGR2A|IL1B|LYN|NCF2|PECAM1|PPARG|TGM2|THBS1|VAV3|OLFM4|RAB31|CD93|APPL2 -2.600933365 0 76 0 0 -4.578220964 21.55530042

1 -4.307229296 GO:0060099 M1 1 0 GO Biological Processes 19 regulation of phagocytosis, engulfment -4.307229296 18.68126036 8.24271197 28162 15 402 4 0.995024876 0.495031064 718|5468|11031|55198C3|PPARG|RAB31|APPL2 -2.540141634 0 76 0 0 -4.578220964 21.55530042

1 -4.22914765 GO:0008643 M1 1 0 GO Biological Processes 19 carbohydrate transport -4.22914765 4.701659488 5.451764928 28162 149 402 10 2.487562189 0.776790586 360|366|718|1906|3553|9963|23433|23596|55198|282679 AQP3|AQP9|C3|EDN1|IL1B|SLC23A1|RHOQ|OPN3|APPL2|AQP11 -2.474421078 0 76 0 0 -4.578220964 21.55530042

1 -4.187204611 GO:1905153 M1 1 0 GO Biological Processes 19 regulation of membrane invagination -4.187204611 17.51368159 7.951020773 28162 16 402 4 0.995024876 0.495031064 718|5468|11031|55198C3|PPARG|RAB31|APPL2 -2.437982479 0 76 0 0 -4.578220964 21.55530042

1 -3.814909236 GO:0002526 M1 1 0 GO Biological Processes 19 acute inflammatory response -3.814909236 5.237736551 5.285037078 28162 107 402 8 1.990049751 0.696552772 12|718|3553|5068|5468|6279|8876|145741 SERPINA3|C3|IL1B|REG3A|PPARG|S100A8|VNN1|C2CD4A -2.137653874 0 76 0 0 -4.578220964 21.55530042

1 -3.704530929 GO:1903959 M1 1 0 GO Biological Processes 19 regulation of anion transmembrane transport -3.704530929 5.048989288 5.143529974 28162 111 402 8 1.990049751 0.696552772 718|1906|3553|5243|7057|23433|23596|55198 C3|EDN1|IL1B|ABCB1|THBS1|RHOQ|OPN3|APPL2 -2.042260029 0 76 0 0 -4.578220964 21.55530042

1 -3.084602541 GO:0010827 M1 1 0 GO Biological Processes 19 regulation of glucose transmembrane transport -3.084602541 5.458809847 4.714704001 28162 77 402 6 1.492537313 0.604761504 718|1906|3553|23433|23596|55198

C3|EDN1|IL1B|RHOQ|OPN3|APPL2 -1.539363628 0 76 0 0 -4.578220964 21.55530042
1 -2.894604439 GO:0015749 M1 1 0 GO Biological Processes 19 monosaccharide transmembrane transport -2.894604439 4.301606005 4.25063735 28162 114 402 7 1.741293532 0.652391792 718|1906|3553|9963|23433|23596|55198 C3|EDN1|IL1B|SLC23A1|RHOQ|OPN3|APPL2 -1.394780918 0 76 0 0 -4.578220964 21.55530042
1 -2.850804816 GO:0034219 M1 1 0 GO Biological Processes 19 carbohydrate transmembrane transport -2.850804816 4.227440384 4.191592848 28162 116 402 7 1.741293532 0.652391792 718|1906|3553|9963|23433|23596|55198 C3|EDN1|IL1B|SLC23A1|RHOQ|OPN3|APPL2 -1.363728798 0 76 0 0 -4.578220964 21.55530042
1 -2.613374383 GO:0050764 M1 1 0 GO Biological Processes 19 regulation of phagocytosis -2.613374383 4.424509034 4.023365184 28162 95 402 6 1.492537313 0.604761504 718|3553|5468|10562|11031|55198 C3|IL1B|PPARG|OLFM4|RAB31|APPL2 -1.176519353 0 76 0 0 -4.578220964 21.55530042
1 -2.568058643 GO:0050766 M1 1 0 GO Biological Processes 19 positive regulation of phagocytosis -2.568058643 5.227964654 4.169476813 28162 67 402 5 1.243781095 0.552765814 718|3553|5468|11031|55198 C3|IL1B|PPARG|RAB31|APPL2 -1.141064218 0 76 0 0 -4.578220964 21.55530042
1 -2.545618045 GO:0045807 M1 1 0 GO Biological Processes 19 positive regulation of endocytosis -2.545618045 4.28906488 3.924985208 28162 98 402 6 1.492537313 0.604761504 718|5468|7474|11031|26585|55198 C3|PPARG|WNT5A|RAB31|GREM1|APPL2 -1.124462116 0 76 0 0 -4.578220964 21.55530042
1 -2.463333621 GO:0060627 M1 1 0 GO Biological Processes 19 regulation of vesicle-mediated transport -2.463333621 2.163852552 3.217286472 28162 518 402 16 3.980099502 0.975022334 301|718|857|3553|3598|4067|4478|5028|5468|7474|9912|10562|11031|26585|55198|57126 ANXA1|C3|CAV1|IL1B|IL13RA2|LYN|MSN|P2RY1|PPARG|WNT5A|ARHGAP44|OLFM4|RAB31|GREM1|APPL2|CD177 -1.062341915 0 76 0 0 -4.578220964 21.55530042
1 -2.314184917 GO:0002673 M1 1 0 GO Biological Processes 19 regulation of acute inflammatory response -2.314184917 5.837893864 4.036853294 28162 48 402 4 0.995024876 0.495031064 718|3553|5468|145741C3|IL1B|PPARG|C2CD4A -0.947537787 0 76 0 0 -4.578220964 21.55530042
1 -2.298726562 GO:1904659 M1 1 0 GO Biological Processes 19 glucose transmembrane transport -2.298726562 3.821166893 3.567553857 28162 110 402 6 1.492537313 0.604761504 718|1906|3553|23433|23596|55198 C3|EDN1|IL1B|RHOQ|OPN3|APPL2 -0.933888995 0 76 0 0 -4.578220964 21.55530042
1 -2.260930261 GO:0008645 M1 1 0 GO Biological Processes 19 hexose

transmembrane transport -2.260930261 3.75293177 3.512896531 28162 112 402 6 1.492537313 0.604761504 718|1906|3553|23433|23596|55198 C3|EDN1|IL1B|RHOQ|OPN3|APPL2 -0.90826231 0 76 0 0 -4.578220964 21.55530042
1 -2.183845757 GO:0002675 M1 1 0 GO Biological Processes 19 positive regulation of acute inflammatory response -2.183845757 7.783858485 4.243874515 28162 27 402 3 0.746268657 0.429247719 718|3553|145741 C3|IL1B|C2CD4A -0.847370775 0 76 0 0 -4.578220964 21.55530042
1 -2.127197601 GO:0019915 M1 1 0 GO Biological Processes 19 lipid storage -2.127197601 4.120866257 3.467660739 28162 85 402 5 1.243781095 0.552765814 718|857|3553|5468|130399C3|CAV1|IL1B|PPARG|ACVR1C -0.801613334 0 76 0 0 -4.578220964 21.55530042
1 -4.548790617 GO:0019441 M1 1 0 GO Biological Processes 19 tryptophan catabolic process to kynurenine -4.548790617 42.03283582 11.04207458 28162 5 402 3 0.746268657 0.429247719 3620|6999|8942 IDO1|TDO2|KYNU -2.738597074 0 77 1 1 -4.548790617 42.03283582
1 -3.781615478 GO:0009074 M1 1 0 GO Biological Processes 19 aromatic amino acid family catabolic process -3.781615478 14.01094527 7.004444534 28162 20 402 4 0.995024876 0.495031064 3620|6999|8942|80150IDO1|TDO2|KYNU|ASRGL1 -2.10846398 0 77 0 0 -4.548790617 42.03283582
1 -3.492744975 GO:0006569 M1 1 0 GO Biological Processes 19 tryptophan catabolic process -3.492744975 21.01641791 7.618317439 28162 10 402 3 0.746268657 0.429247719 3620|6999|8942 IDO1|TDO2|KYNU -1.882719663 0 77 0 0 -4.548790617 42.03283582
1 -3.492744975 GO:0042436 M1 1 0 GO Biological Processes 19 indole-containing compound catabolic process -3.492744975 21.01641791 7.618317439 28162 10 402 3 0.746268657 0.429247719 3620|6999|8942 IDO1|TDO2|KYNU -1.882719663 0 77 0 0 -4.548790617 42.03283582
1 -3.492744975 GO:0046218 M1 1 0 GO Biological Processes 19 indolalkylamine catabolic process -3.492744975 21.01641791 7.618317439 28162 10 402 3 0.746268657 0.429247719 3620|6999|8942 IDO1|TDO2|KYNU -1.882719663 0 77 0 0 -4.548790617 42.03283582
1 -3.359059655 GO:0070189 M1 1 0 GO Biological Processes 19 kynurenine metabolic process -3.359059655 19.10583446 7.227619926 28162 11 402 3 0.746268657 0.429247719 3620|6999|8942 IDO1|TDO2|KYNU -1.767683125 0 77 0 0 -4.548790617 42.03283582
1 -3.23873503 GO:0006568 M1 1 0 GO Biological Processes 19 tryptophan metabolic process -3.23873503 17.51368159 6.885296737 28162 12 402 3 0.746268657 0.429247719 3620|6999|8942 IDO1|TDO2|KYNU -1.672477766 0 77 0 0 -4.548790617 42.03283582
1 -2.969765682 GO:0009072 M1 1 0 GO Biological Processes 19 aromatic amino acid family metabolic process -2.969765682 8.756840796 5.283264248 28162 32 402 4 0.995024876 0.495031064 3620|6999|8942|80150IDO1|TDO2|KYNU|ASRGL1 -1.454261956 0 77 0 0 -4.548790617 42.03283582

1 -2.82120143 GO:0072525 M1 1 0 GO Biological Processes 19 pyridine-containing compound biosynthetic process -2.82120143 8.006254442 4.99097682 28162 35 402 4 0.995024876 0.495031064 3620|4837|8942|29968IDO1|NNMT|KYNU|PSAT1 -1.337106169 0 77 0 0 -4.548790617 42.03283582
1 -2.771670055 GO:0006586 M1 1 0 GO Biological Processes 19 indolalkylamine metabolic process -2.771670055 12.36259877 5.639347249 28162 17 402 3 0.746268657 0.429247719 3620|6999|8942 IDO1|TDO2|KYNU -1.303326622 0 77 0 0 -4.548790617 42.03283582
1 -2.52527329 GO:0072524 M1 1 0 GO Biological Processes 19 pyridine-containing compound metabolic process -2.52527329 6.671878702 4.426609925 28162 42 402 4 0.995024876 0.495031064 3620|4837|8942|29968IDO1|NNMT|KYNU|PSAT1 -1.108908949 0 77 0 0 -4.548790617 42.03283582
1 -2.377043435 GO:0009063 M1 1 0 GO Biological Processes 19 cellular amino acid catabolic process -2.377043435 3.96536187 3.680824297 28162 106 402 6 1.492537313 0.604761504 18|3620|4843|6999|8942|80150 ABAT|IDO1|NOS2|TDO2|KYNU|ASRGL1 -0.996578772 0 77 0 0 -4.548790617 42.03283582
1 -2.330058653 GO:0016702 M1 1 0 GO Molecular Functions 21 oxidoreductase activity, acting on single donors with incorporation of molecular oxygen, incorporation of two atoms of oxygen -2.330058653 8.756840796 4.574790579 28162 24 402 3 0.746268657 0.429247719 240|3620|6999 ALOX5|IDO1|TDO2 -0.95976962 0 77 0 0 -4.548790617 42.03283582
1 -2.330058653 GO:0042430 M1 1 0 GO Biological Processes 19 indole-containing compound metabolic process -2.330058653 8.756840796 4.574790579 28162 24 402 3 0.746268657 0.429247719 3620|6999|8942 IDO1|TDO2|KYNU -0.95976962 0 77 0 0 -4.548790617 42.03283582
1 -2.279112201 GO:0016701 M1 1 0 GO Molecular Functions 21 oxidoreductase activity, acting on single donors with incorporation of molecular oxygen -2.279112201 8.406567164 4.458362801 28162 25 402 3 0.746268657 0.429247719 240|3620|6999 ALOX5|IDO1|TDO2 -0.923653081 0 77 0 0 -4.548790617 42.03283582
1 -2.279112201 GO:0042537 M1 1 0 GO Biological Processes 19 benzene-containing compound metabolic process -2.279112201 8.406567164 4.458362801 28162 25 402 3 0.746268657 0.429247719 3620|6999|8942 IDO1|TDO2|KYNU -0.923653081 0 77 0 0 -4.548790617 42.03283582
1 -2.183390299 GO:1901605 M1 1 0 GO Biological Processes 19 alpha-amino acid metabolic process -2.183390299 2.918946932 3.210662664 28162 192 402 8 1.990049751 0.696552772 1503|3620|4843|6999|8942|23657|29968|80150 CTPS1|IDO1|NOS2|TDO2|KYNU|SLC7A11|PSAT1|ASRGL1 -0.847370775 0 77 0 0 -4.548790617 42.03283582
1 -2.096321068 GO:0009435 M1 1 0 GO Biological Processes 19 NAD biosynthetic process -2.096321068 7.247040659 4.050350813 28162 29 402 3 0.746268657

0.429247719 3620|4837|8942 IDO1|NNMT|KYNU -0.778908152 0 77 0 0
-4.548790617 42.03283582
1 -2.065039701 GO:1901606 M1 1 0 GO Biological Processes 19 alpha-amino acid catabolic process -2.065039701 3.98038218 3.369678588 28162 88 402 5
1.243781095 0.552765814 3620|4843|6999|8942|80150
IDO1|NOS2|TDO2|KYNU|ASRGL1 -0.752056345 0 77 0 0 -4.548790617
42.03283582
1 -2.055117446 GO:0042402 M1 1 0 GO Biological Processes 19 cellular biogenic amine catabolic process -2.055117446 7.005472637 3.96036166 28162 30 402 3
0.746268657 0.429247719 3620|6999|8942 IDO1|TDO2|KYNU -0.74572497 0 77
0 0 -4.548790617 42.03283582
1 -2.044913511 GO:0051213 M1 1 0 GO Molecular Functions 21 dioxygenase activity -2.044913511 3.935658785 3.337978215 28162 89 402 5 1.243781095
0.552765814 240|3620|6999|55214|57168 ALOX5|IDO1|TDO2|P3H2|ASPHD2
-0.738293558 0 77 0 0 -4.548790617 42.03283582
1 -4.469550455 GO:0048762 M1 1 0 GO Biological Processes 19 mesenchymal cell differentiation -4.469550455 3.908632802 5.364960107 28162 233 402 13
3.233830846 0.882281956
1277|1906|1908|1942|2263|3553|4017|5308|7474|10979|26585|28984|64081
COL1A1|EDN1|EDN3|EFNA1|FGFR2|IL1B|LOXL2|PITX2|WNT5A|FERMT2|GREM1|RGCC|PBLD -2.670541078 0 78 1 1 -4.469550455 6.868110428
1 -4.11605097 GO:0060485 M1 1 0 GO Biological Processes 19 mesenchyme development -4.11605097 3.405438087 4.93762509 28162 288 402 14
3.482587065 0.914409651
1277|1906|1908|1942|2263|3553|4017|5308|6091|7474|10979|26585|28984|64081
COL1A1|EDN1|EDN3|EFNA1|FGFR2|IL1B|LOXL2|PITX2|ROBO1|WNT5A|FERMT2|GREM1|RGCC|PBLD -2.38167125 0 78 0 0 -4.469550455 6.868110428
1 -4.085162049 GO:0001837 M1 1 0 GO Biological Processes 19 epithelial to mesenchymal transition -4.085162049 4.519659766 5.287624047 28162 155 402 10
2.487562189 0.776790586
1277|1942|2263|3553|4017|7474|10979|26585|28984|64081
COL1A1|EFNA1|FGFR2|IL1B|LOXL2|WNT5A|FERMT2|GREM1|RGCC|PBLD
-2.355197507 0 78 0 0 -4.469550455 6.868110428
1 -4.020806939 GO:0010717 M1 1 0 GO Biological Processes 19 regulation of epithelial to mesenchymal transition -4.020806939 5.604378109 5.550588126 28162
100 402 8 1.990049751 0.696552772
1277|1942|3553|4017|10979|26585|28984|64081
COL1A1|EFNA1|IL1B|LOXL2|FERMT2|GREM1|RGCC|PBLD -2.305460374 0 78 0 0
-4.469550455 6.868110428
1 -3.101378943 GO:0010718 M1 1 0 GO Biological Processes 19 positive regulation of epithelial to mesenchymal transition -3.101378943 6.868110428 5.0474561 28162
51 402 5 1.243781095 0.552765814 1277|3553|4017|10979|28984
COL1A1|IL1B|LOXL2|FERMT2|RGCC -1.553395664 0 78 0 0 -4.469550455
6.868110428

1 -4.432878973 GO:0046718 M1 1 0 GO Biological Processes 19 viral entry into host cell -4.432878973 4.96842031 5.684767887 28162 141 402 10 2.487562189 0.776790586 290|682|857|1604|3373|9076|10346|10410|10581|27074 ANPEP|BSG|CAV1|CD55|HYAL1|CLDN1|TRIM22|IFITM3|IFITM2|LAMP3 -2.641168835 0 79 1 1 -4.432878973 4.96842031
1 -4.108665923 GO:0044409 M1 1 0 GO Biological Processes 19 entry into host -4.108665923 4.549008206 5.31439275 28162 154 402 10 2.487562189 0.776790586 290|682|857|1604|3373|9076|10346|10410|10581|27074 ANPEP|BSG|CAV1|CD55|HYAL1|CLDN1|TRIM22|IFITM3|IFITM2|LAMP3 -2.376603336 0 79 0 0 -4.432878973 4.96842031
1 -3.632216914 GO:0052126 M1 1 0 GO Biological Processes 19 movement in host environment -3.632216914 3.98038218 4.772931582 28162 176 402 10 2.487562189 0.776790586 290|682|857|1604|3373|9076|10346|10410|10581|27074 ANPEP|BSG|CAV1|CD55|HYAL1|CLDN1|TRIM22|IFITM3|IFITM2|LAMP3 -1.986459488 0 79 0 0 -4.432878973 4.96842031
1 -3.458269663 GO:0051701 M1 1 0 GO Biological Processes 19 biological process involved in interaction with host -3.458269663 3.502736318 4.484613543 28162 220 402 11 2.736318408 0.813665012 290|682|857|1604|3373|5272|9076|10346|10410|10581|27074 ANPEP|BSG|CAV1|CD55|HYAL1|SERPINB9|CLDN1|TRIM22|IFITM3|IFITM2|LAMP3 -1.852982156 0 79 0 0 -4.432878973 4.96842031
1 -3.261910978 GO:0019058 M1 1 0 GO Biological Processes 19 viral life cycle -3.261910978 2.81021825 4.094935509 28162 349 402 14 3.482587065 0.914409651 290|682|857|1604|2040|3373|3669|9076|9397|9582|10346|10410|10581|27074 ANPEP|BSG|CAV1|CD55|STOM|HYAL1|ISG20|CLDN1|NMT2|APOBEC3B|TRIM22|IFITM3|IFITM2|LAMP3 -1.687684784 0 79 0 0 -4.432878973 4.96842031
1 -2.283592463 GO:0001618 M1 1 0 GO Molecular Functions 21 virus receptor activity -2.283592463 4.490687588 3.71497703 28162 78 402 5 1.243781095 0.552765814 290|682|1604|3373|9076 ANPEP|BSG|CD55|HYAL1|CLDN1 -0.923653081 0 79 0 0 -4.432878973 4.96842031
1 -2.260201294 GO:0140272 M1 1 0 GO Molecular Functions 21 exogenous protein binding -2.260201294 4.433843441 3.67789747 28162 79 402 5 1.243781095 0.552765814 290|682|1604|3373|9076 ANPEP|BSG|CD55|HYAL1|CLDN1 -0.90826231 0 79 0 0 -4.432878973 4.96842031
1 -4.418025472 GO:0050829 M1 1 0 GO Biological Processes 19 defense response to Gram-negative bacterium -4.418025472 6.368611488 6.069857517 28162 88 402 8 1.990049751 0.696552772 1670|1671|1672|1755|4843|6372|11126|646627 DEFA5|DEFA6|DEFB1|DMBT1|NOS2|CXCL6|CD160|LYPD8 -2.627520036 0 80 1 1 -4.418025472 10.37847798
1 -3.761934469 GO:0002385 M1 1 0 GO Biological Processes 19 mucosal immune response -3.761934469 9.466854915 6.201604217 28162 37 402 5 1.243781095 0.552765814 1670|1671|1672|4843|11126 DEFA5|DEFA6|DEFB1|NOS2|CD160 -2.090738316 0 80 0 0 -4.418025472 10.37847798

1 -3.593035205 GO:0019731 M1 1 0 GO Biological Processes 19 antibacterial humoral response -3.593035205 6.779489649 5.482258467 28162 62 402 6 1.492537313 0.604761504 1670|1671|1672|1755|5266|11005 DEFA5|DEFA6|DEFB1|DMBT1|PI3|SPINK5 -1.960189924 0 80 0 0 -4.418025472 10.37847798

1 -3.546913934 GO:0002251 M1 1 0 GO Biological Processes 19 organ or tissue specific immune response -3.546913934 8.543259313 5.816515875 28162 41 402 5 1.243781095 0.552765814 1670|1671|1672|4843|11126 DEFA5|DEFA6|DEFB1|NOS2|CD160 -1.91988704 0 80 0 0 -4.418025472 10.37847798

1 -3.256901045 GO:0002227 M1 1 0 GO Biological Processes 19 innate immune response in mucosa -3.256901045 10.37847798 5.867027412 28162 27 402 4 0.995024876 0.495031064 1670|1671|1672|4843 DEFA5|DEFA6|DEFB1|NOS2 -1.684862738 0 80 0 0 -4.418025472 10.37847798

1 -2.480423272 GO:0050830 M1 1 0 GO Biological Processes 19 defense response to Gram-positive bacterium -2.480423272 4.161666913 3.830474374 28162 101 402 6 1.492537313 0.604761504 1670|1671|1672|1755|3575|5320 DEFA5|DEFA6|DEFB1|DMBT1|IL7R|PLA2G2A -1.076893306 0 80 0 0 -4.418025472 10.37847798

1 -4.410695559 GO:0016491 M1 1 0 GO Molecular Functions 21 oxidoreductase activity -4.410695559 2.409289531 4.726782607 28162 756 402 26 6.467661692 1.226708902 51|126|240|1555|2330|3248|3284|3294|3620|4017|4199|4688|4843|6241|6999|7837|8991|29785|50506|55214|57168|79154|79644|79689|195814|493869 ACOX1|ADH1C|ALOX5|CYP2B6|FMO5|HPGD|HSD3B2|HSD17B2|IDO1|LOXL2|ME1|NCF2|NOS2|RRM2|TDO2|PXDN|SELENBP1|CYP2S1|DUOX2|P3H2|ASPHD2|DHRS11|SRD5A3|STEAP4|SDR16C5|GPX8 -2.621391491 0 81 1 1 -4.410695559 4.203283582

1 -3.49698441 GO:0046906 M1 1 0 GO Molecular Functions 21 tetrapyrrole binding -3.49698441 4.203283582 4.733651601 28162 150 402 9 2.23880597 0.737867616 1555|3620|4843|6947|6999|7837|29785|50506|79689 CYP2B6|IDO1|NOS2|TCN1|TDO2|PXDN|CYP2S1|DUOX2|STEAP4 -1.882719663 0 81 0 0 -4.410695559 4.203283582

1 -3.029355999 GO:0020037 M1 1 0 GO Molecular Functions 21 heme binding -3.029355999 4.003127221 4.286625232 28162 140 402 8 1.990049751 0.696552772 1555|3620|4843|6999|7837|29785|50506|79689 CYP2B6|IDO1|NOS2|TDO2|PXDN|CYP2S1|DUOX2|STEAP4 -1.500806012 0 81 0 0 -4.410695559 4.203283582

1 -4.401375843 GO:0050839 M1 1 0 GO Molecular Functions 21 cell adhesion molecule binding -4.401375843 2.719314702 4.859164955 28162 541 402 21 5.223880597 1.109770192 288|301|682|1001|1009|1281|3553|4067|4478|6282|6286|6696|7045|7057|7450|10562|10979|23650|53841|57126|143098 ANK3|ANXA1|BSG|CDH3|CDH11|COL3A1|IL1B|LYN|MSN|S100A11|S100P|SPP1|TGFBI|THBS1|VWF|OLFM4|FERMT2|TRIM29|CDHR5|CD177|MPP7 -2.613269829 0 82 1 1

-4.401375843 4.440088291
1 -3.674095093 GO:0005178 M1 1 0 GO Molecular Functions 21 integrin binding -3.674095093 4.440088291 4.945463805 28162 142 402 9 2.23880597 0.737867616 1281|3553|4067|6696|7045|7057|7450|10979|57126 COL3A1|IL1B|LYN|SPP1|TGFBI|THBS1|VWF|FERMT2|CD177 -2.016278535 0 82 0 0 -4.401375843 4.440088291
1 -4.326819502 GO:0045981 M1 1 0 GO Biological Processes 19 positive regulation of nucleotide metabolic process -4.326819502 9.137573005 6.646980095 28162 46 402 6 1.492537313 0.604761504 957|2981|4843|5209|8671|10891 ENTPD5|GUCA2B|NOS2|PFKFB3|SLC4A4|PPARGC1A -2.550515695 0 83 1 1 -4.326819502 9.552917232
1 -4.326819502 GO:1900544 M1 1 0 GO Biological Processes 19 positive regulation of purine nucleotide metabolic process -4.326819502 9.137573005 6.646980095 28162 46 402 6 1.492537313 0.604761504 957|2981|4843|5209|8671|10891 ENTPD5|GUCA2B|NOS2|PFKFB3|SLC4A4|PPARGC1A -2.550515695 0 83 0 0 -4.326819502 9.552917232
1 -3.49698441 GO:0006090 M1 1 0 GO Biological Processes 19 pyruvate metabolic process -3.49698441 4.203283582 4.733651601 28162 150 402 9 2.23880597 0.737867616 682|957|4199|5105|5166|5209|6566|8671|10891 BSG|ENTPD5|ME1|PCK1|PDK4|PFKFB3|SLC16A1|SLC4A4|PPARGC1A -1.882719663 0 83 0 0 -4.326819502 9.552917232
1 -2.766018436 GO:1900542 M1 1 0 GO Biological Processes 19 regulation of purine nucleotide metabolic process -2.766018436 4.086525705 4.077400395 28162 120 402 7 1.741293532 0.652391792 957|2981|4843|5166|5209|8671|10891 ENTPD5|GUCA2B|NOS2|PDK4|PFKFB3|SLC4A4|PPARGC1A -1.299390454 0 83 0 0 -4.326819502 9.552917232
1 -2.724967491 GO:0006140 M1 1 0 GO Biological Processes 19 regulation of nucleotide metabolic process -2.724967491 4.01953348 4.022148417 28162 122 402 7 1.741293532 0.652391792 957|2981|4843|5166|5209|8671|10891 ENTPD5|GUCA2B|NOS2|PDK4|PFKFB3|SLC4A4|PPARGC1A -1.265695813 0 83 0 0 -4.326819502 9.552917232
1 -2.563886473 GO:1903580 M1 1 0 GO Biological Processes 19 positive regulation of ATP metabolic process -2.563886473 6.834607451 4.498995123 28162 41 402 4 0.995024876 0.495031064 957|5209|8671|10891 ENTPD5|PFKFB3|SLC4A4|PPARGC1A -1.138080112 0 83 0 0 -4.326819502 9.552917232
1 -2.52527329 GO:1900371 M1 1 0 GO Biological Processes 19 regulation of purine nucleotide biosynthetic process -2.52527329 6.671878702 4.426609925 28162 42 402 4 0.995024876 0.495031064 2981|4843|5166|10891 GUCA2B|NOS2|PDK4|PPARGC1A -1.108908949 0 83 0 0 -4.326819502 9.552917232
1 -2.487719498 GO:0030808 M1 1 0 GO Biological Processes 19 regulation of nucleotide biosynthetic process -2.487719498 6.516718732 4.356547625 28162 43 402 4 0.995024876 0.495031064 2981|4843|5166|10891

GUCA2B|NOS2|PDK4|PPARGC1A -1.080223345 0 83 0 0 -4.326819502
9.552917232
1 -2.439596569 GO:0030810 M1 1 0 GO Biological Processes 19 positive regulation of nucleotide biosynthetic process -2.439596569 9.552917232 4.829374617 28162 22
402 3 0.746268657 0.429247719 2981|4843|10891 GUCA2B|NOS2|PPARGC1A
-1.046308721 0 83 0 0 -4.326819502 9.552917232
1 -2.439596569 GO:0043457 M1 1 0 GO Biological Processes 19 regulation of cellular respiration -2.439596569 9.552917232 4.829374617 28162 22 402 3
0.746268657 0.429247719 4843|10891|23596 NOS2|PPARGC1A|OPN3
-1.046308721 0 83 0 0 -4.326819502 9.552917232
1 -2.439596569 GO:1900373 M1 1 0 GO Biological Processes 19 positive regulation of purine nucleotide biosynthetic process -2.439596569 9.552917232 4.829374617
28162 22 402 3 0.746268657 0.429247719 2981|4843|10891
GUCA2B|NOS2|PPARGC1A -1.046308721 0 83 0 0 -4.326819502 9.552917232
1 -2.330058653 GO:0045821 M1 1 0 GO Biological Processes 19 positive regulation of glycolytic process -2.330058653 8.756840796 4.574790579 28162 24 402 3
0.746268657 0.429247719 957|5209|8671 ENTPD5|PFKFB3|SLC4A4 -0.95976962
0 83 0 0 -4.326819502 9.552917232
1 -2.283592463 GO:0045913 M1 1 0 GO Biological Processes 19 positive regulation of carbohydrate metabolic process -2.283592463 4.490687588 3.71497703 28162
78 402 5 1.243781095 0.552765814 957|5028|5209|8671|10891
ENTPD5|P2RY1|PFKFB3|SLC4A4|PPARGC1A-0.923653081 0 83 0 0
-4.326819502 9.552917232
1 -4.178929961 GO:0032602 M1 1 0 GO Biological Processes 19 chemokine production -4.178929961 5.899345378 5.756111439 28162 95 402 8
1.990049751 0.696552772 3553|6279|6280|6372|7098|7474|90865|255231
IL1B|S100A8|S100A9|CXCL6|TLR3|WNT5A|IL33|MCOLN2-2.430800394 0 84 1 1
-4.178929961 5.899345378
1 -3.561957608 GO:0002700 M1 1 0 GO Biological Processes 19 regulation of production of molecular mediator of immune response -3.561957608 4.28906488
4.811307632 28162 147 402 9 2.23880597 0.737867616
3553|3598|7098|7474|8140|11005|11126|51237|90865
IL1B|IL13RA2|TLR3|WNT5A|SLC7A5|SPINK5|CD160|MZB1|IL33 -1.932446659 0 84
0 0 -4.178929961 5.899345378
1 -3.154112978 GO:0032637 M1 1 0 GO Biological Processes 19 interleukin-8 production -3.154112978 4.761000821 4.601646765 28162 103 402 7
1.741293532 0.652391792 301|1116|3553|4843|7098|7474|64922
ANXA1|CHI3L1|IL1B|NOS2|TLR3|WNT5A|LRRC19 -1.597088814 0 84 0 0
-4.178929961 5.899345378
1 -3.055892573 GO:0032649 M1 1 0 GO Biological Processes 19 regulation of interferon-gamma production -3.055892573 4.583019482 4.468515347 28162 107
402 7 1.741293532 0.652391792 3553|7098|7474|8140|11126|29126|90865
IL1B|TLR3|WNT5A|SLC7A5|CD160|CD274|IL33 -1.519226276 0 84 0 0
-4.178929961 5.899345378

1 -3.013788366 GO:0045766 M1 1 0 GO Biological Processes 19 positive regulation of angiogenesis -3.013788366 3.602814499 4.156343186 28162 175 402 9 2.23880597 0.737867616 718|1116|3373|3553|6356|7057|7098|7474|26585 C3|CHI3L1|HYAL1|IL1B|CCL11|THBS1|TLR3|WNT5A|GREM1 -1.49185053 0 84 0 0 -4.178929961 5.899345378

1 -3.013788366 GO:1904018 M1 1 0 GO Biological Processes 19 positive regulation of vasculature development -3.013788366 3.602814499 4.156343186 28162 175 402 9 2.23880597 0.737867616 718|1116|3373|3553|6356|7057|7098|7474|26585 C3|CHI3L1|HYAL1|IL1B|CCL11|THBS1|TLR3|WNT5A|GREM1 -1.49185053 0 84 0 0 -4.178929961 5.899345378

1 -2.939387393 GO:0032609 M1 1 0 GO Biological Processes 19 interferon-gamma production -2.939387393 4.378420398 4.311055372 28162 112 402 7 1.741293532 0.652391792 3553|7098|7474|8140|11126|29126|90865 IL1B|TLR3|WNT5A|SLC7A5|CD160|CD274|IL33 -1.429689558 0 84 0 0 -4.178929961 5.899345378

1 -2.780387855 GO:0032722 M1 1 0 GO Biological Processes 19 positive regulation of chemokine production -2.780387855 5.837893864 4.514302722 28162 60 402 5 1.243781095 0.552765814 3553|7098|7474|90865|255231 IL1B|TLR3|WNT5A|IL33|MCOLN2-1.305114044 0 84 0 0 -4.178929961 5.899345378

1 -2.75726963 GO:0032642 M1 1 0 GO Biological Processes 19 regulation of chemokine production -2.75726963 4.722790542 4.232983 28162 89 402 6 1.492537313 0.604761504 3553|6372|7098|7474|90865|255231 IL1B|CXCL6|TLR3|WNT5A|IL33|MCOLN2 -1.291781529 0 84 0 0 -4.178929961 5.899345378

1 -2.636557988 GO:0002367 M1 1 0 GO Biological Processes 19 cytokine production involved in immune response -2.636557988 4.471578279 4.057069871 28162 94 402 6 1.492537313 0.604761504 1604|3553|7098|7474|8140|11126 CD55|IL1B|TLR3|WNT5A|SLC7A5|CD160 -1.194880391 0 84 0 0 -4.178929961 5.899345378

1 -2.625899697 GO:0032729 M1 1 0 GO Biological Processes 19 positive regulation of interferon-gamma production -2.625899697 5.388825105 4.262872164 28162 65 402 5 1.243781095 0.552765814 3553|7098|7474|8140|11126 IL1B|TLR3|WNT5A|SLC7A5|CD160 -1.186907913 0 84 0 0 -4.178929961 5.899345378

1 -2.568058643 GO:1901224 M1 1 0 GO Biological Processes 19 positive regulation of NIK/NF-kappaB signaling -2.568058643 5.227964654 4.169476813 28162 67 402 5 1.243781095 0.552765814 1906|3553|7098|26585|64922 EDN1|IL1B|TLR3|GREM1|LRRC19 -1.141064218 0 84 0 0 -4.178929961 5.899345378

1 -2.501880095 GO:0002702 M1 1 0 GO Biological Processes 19 positive regulation of production of molecular mediator of immune response -2.501880095 4.203283582 3.861565534 28162 100 402 6 1.492537313 0.604761504 3553|7474|8140|11126|51237|90865IL1B|WNT5A|SLC7A5|CD160|MZB1|IL33

-1.089374722 0 84 0 0 -4.178929961 5.899345378
1 -2.160825932 GO:0002720 M1 1 0 GO Biological Processes 19 positive regulation of cytokine production involved in immune response -2.160825932 5.28714916 3.759336167 28162 53 402 4 0.995024876 0.495031064 3553|7474|8140|11126IL1B|WNT5A|SLC7A5|CD160 -0.827753644 0 84 0 0 -4.178929961 5.899345378
1 -2.148534079 GO:0002718 M1 1 0 GO Biological Processes 19 regulation of cytokine production involved in immune response -2.148534079 4.169924189 3.501327751 28162 84 402 5 1.243781095 0.552765814 3553|7098|7474|8140|11126 IL1B|TLR3|WNT5A|SLC7A5|CD160 -0.816300197 0 84 0 0 -4.178929961 5.899345378
1 -2.106174654 GO:0032755 M1 1 0 GO Biological Processes 19 positive regulation of interleukin-6 production -2.106174654 4.072949207 3.434506341 28162 86 402 5 1.243781095 0.552765814 3553|5450|7098|7474|90865 IL1B|POU2AF1|TLR3|WNT5A|IL33 -0.783467881 0 84 0 0 -4.178929961 5.899345378
1 -2.054485428 GO:0002440 M1 1 0 GO Biological Processes 19 production of molecular mediator of immune response -2.054485428 2.432455777 2.940391449 28162 288 402 10 2.487562189 0.776790586 1604|3553|3598|7098|7474|8140|11005|11126|51237|90865 CD55|IL1B|IL13RA2|TLR3|WNT5A|SLC7A5|SPINK5|CD160|MZB1|IL33 -0.745490112 0 84 0 0 -4.178929961 5.899345378
1 -4.174951985 GO:0001655 M1 1 0 GO Biological Processes 19 urogenital system development -4.174951985 3.273585344 4.930020147 28162 321 402 15 3.731343284 0.945283402 183|301|1282|2263|3248|3371|4038|4318|4811|5175|5268|7474|9510|26585|282679 AGT|ANXA1|COL4A1|FGFR2|HPGD|TNC|LRP4|MMP9|NID1|PECAM1|SERPINB5|WNT5A|ADAMTS1|GREM1|AQP11 -2.427912242 0 85 1 1 -4.174951985 3.273585344
1 -3.002121605 GO:0072001 M1 1 0 GO Biological Processes 19 renal system development -3.002121605 2.90884677 3.925142817 28162 289 402 12 2.985074627 0.848757934 183|1282|2263|3248|4038|4318|4811|5175|7474|9510|26585|282679 AGT|COL4A1|FGFR2|HPGD|LRP4|MMP9|NID1|PECAM1|WNT5A|ADAMTS1|GREM1|AQP11 -1.48147824 0 85 0 0 -4.174951985 3.273585344
1 -2.597720393 GO:0001822 M1 1 0 GO Biological Processes 19 kidney development -2.597720393 2.742355836 3.532345481 28162 281 402 11 2.736318408 0.813665012 183|2263|3248|4038|4318|4811|5175|7474|9510|26585|282679 AGT|FGFR2|HPGD|LRP4|MMP9|NID1|PECAM1|WNT5A|ADAMTS1|GREM1|AQP11 -1.163521607 0 85 0 0 -4.174951985 3.273585344
1 -4.140582614 GO:0072337 M1 1 0 GO Biological Processes 19 modified amino acid transport -4.140582614 11.29914941 6.904257052 28162 31 402 5 1.243781095 0.552765814 2697|5174|6519|6584|23657 GJA1|PDZK1|SLC3A1|SLC22A5|SLC7A11 -2.401096009 0 86 1 1

-4.140582614 12.73722298
1 -3.612497705 GO:0072349 M1 1 0 GO Molecular Functions 21 modified amino acid transmembrane transporter activity -3.612497705 12.73722298 6.627381649 28162 22 402 4 0.995024876 0.495031064 2697|6519|6584|23657 GJA1|SLC3A1|SLC22A5|SLC7A11 -1.972814426 0 86 0 0 -4.140582614 12.73722298
1 -2.886675727 GO:0030165 M1 1 0 GO Molecular Functions 21 PDZ domain binding -2.886675727 5.003909026 4.422502543 28162 84 402 6 1.492537313 0.604761504 51|2697|5174|6550|6584|23705 ACOX1|GJA1|PDZK1|SLC9A3|SLC22A5|CADM1 -1.388082502 0 86 0 0 -4.140582614 12.73722298
1 -4.114786673 GO:0045121 M1 1 0 GO Cellular Components 20 membrane raft -4.114786673 3.233295063 4.873084067 28162 325 402 15 3.731343284 0.945283402 682|857|1604|2040|2697|4038|4067|5174|5175|9429|10008|23433|55824|57126|83483 BSG|CAV1|CD55|STOM|GJA1|LRP4|LYN|PDZK1|PECAM1|ABCG2|KCNE3|RHOQ|PAG1|CD177|PLVAP -2.38167125 0 87 1 1 -4.114786673 3.233295063
1 -4.114786673 GO:0098857 M1 1 0 GO Cellular Components 20 membrane microdomain -4.114786673 3.233295063 4.873084067 28162 325 402 15 3.731343284 0.945283402 682|857|1604|2040|2697|4038|4067|5174|5175|9429|10008|23433|55824|57126|83483 BSG|CAV1|CD55|STOM|GJA1|LRP4|LYN|PDZK1|PECAM1|ABCG2|KCNE3|RHOQ|PAG1|CD177|PLVAP -2.38167125 0 87 0 0 -4.114786673 3.233295063
1 -4.052949363 GO:0006979 M1 1 0 GO Biological Processes 19 response to oxidative stress -4.052949363 2.820995693 4.670168719 28162 447 402 18 4.47761194 1.031484687 240|301|952|1277|1906|3373|4313|4314|4318|4688|6505|7837|8876|10891|11030|23657|50506|493869 ALOX5|ANXA1|CD38|COL1A1|EDN1|HYAL1|MMP2|MMP3|MMP9|NCF2|SLC1A1|PXDN|VNN1|PPARGC1A|RBPMS|SLC7A11|DUOX2|GPX8 -2.326113007 0 88 1 1 -4.052949363 2.820995693
1 -2.779730914 GO:0062197 M1 1 0 GO Biological Processes 19 cellular response to chemical stress -2.779730914 2.609488375 3.640859934 28162 349 402 13 3.233830846 0.882281956 240|301|857|4313|4314|4318|4688|5468|6505|8876|10891|23657|493869 ALOX5|ANXA1|CAV1|MMP2|MMP3|MMP9|NCF2|PPARG|SLC1A1|VNN1|PPARGC1A|SLC7A11|GPX8 -1.305038877 0 88 0 0 -4.052949363 2.820995693
1 -2.372078398 GO:0034599 M1 1 0 GO Biological Processes 19 cellular response to oxidative stress -2.372078398 2.560139502 3.274731239 28162 301 402 11 2.736318408 0.813665012 240|301|4313|4314|4318|4688|6505|8876|10891|23657|493869 ALOX5|ANXA1|MMP2|MMP3|MMP9|NCF2|SLC1A1|VNN1|PPARGC1A|SLC7A11|GPX8

-0.992507816 0 88 0 0 -4.052949363 2.820995693
1 -4.016613408 GO:0002576 M1 1 0 GO Biological Processes 19 platelet degranulation -4.016613408 4.925722948 5.3568493 28162 128 402 9 2.23880597 0.737867616 12|308|4067|5175|6678|7057|7076|7450|7873 SERPINA3|ANXA5|LYN|PECAM1|SPARC|THBS1|TIMP1|VWF|MANF -2.305460374 0 89 1 1 -4.016613408 5.388825105
1 -3.479625004 GO:0031091 M1 1 0 GO Cellular Components 20 platelet alpha granule -3.479625004 5.388825105 5.046233711 28162 91 402 7 1.741293532 0.652391792 12|5175|5270|6678|7057|7076|7450 SERPINA3|PECAM1|SERPINE2|SPARC|THBS1|TIMP1|VWF -1.87118471 0 89 0 0 -4.016613408 5.388825105
1 -2.568058643 GO:0031093 M1 1 0 GO Cellular Components 20 platelet alpha granule lumen -2.568058643 5.227964654 4.169476813 28162 67 402 5 1.243781095 0.552765814 12|6678|7057|7076|7450 SERPINA3|SPARC|THBS1|TIMP1|VWF -1.141064218 0 89 0 0 -4.016613408 5.388825105
1 -4.005618796 GO:0035633 M1 1 0 GO Biological Processes 19 maintenance of blood-brain barrier -4.005618796 10.61435248 6.650084986 28162 33 402 5 1.243781095 0.552765814 2697|3915|5175|6505|9076 GJA1|LAMC1|PECAM1|SLC1A1|CLDN1 -2.29906217 0 90 1 1 -4.005618796 10.61435248
1 -2.989459592 GO:0003158 M1 1 0 GO Biological Processes 19 endothelium development -2.989459592 3.946745148 4.236234723 28162 142 402 8 1.990049751 0.696552772 1306|2697|3553|3627|4478|5175|9076|11167 COL15A1|GJA1|IL1B|CXCL10|MSN|PECAM1|CLDN1|FSTL1 -1.47010685 0 90 0 0 -4.005618796 10.61435248
1 -2.458411739 GO:0001885 M1 1 0 GO Biological Processes 19 endothelial cell development -2.458411739 4.933431434 3.99341542 28162 71 402 5 1.243781095 0.552765814 1306|3553|4478|5175|9076 COL15A1|IL1B|MSN|PECAM1|CLDN1 -1.060276805 0 90 0 0 -4.005618796 10.61435248
1 -2.160825932 GO:0061028 M1 1 0 GO Biological Processes 19 establishment of endothelial barrier -2.160825932 5.28714916 3.759336167 28162 53 402 4 0.995024876 0.495031064 3553|4478|5175|9076 IL1B|MSN|PECAM1|CLDN1 -0.827753644 0 90 0 0 -4.005618796 10.61435248
1 -2.051262324 GO:0045446 M1 1 0 GO Biological Processes 19 endothelial cell differentiation -2.051262324 3.389744824 3.209343395 28162 124 402 6 1.492537313 0.604761504 1306|3553|4478|5175|9076|11167 COL15A1|IL1B|MSN|PECAM1|CLDN1|FSTL1 -0.743060239 0 90 0 0 -4.005618796 10.61435248
1 -4.005600698 GO:0007162 M1 1 0 GO Biological Processes 19 negative regulation of cell adhesion -4.005600698 3.324631082 4.830001565 28162 295 402 14 3.482587065 0.914409651 301|1277|2633|3371|3557|3620|4321|5270|7045|7057|28984|29126|55824|124872

ANXA1|COL1A1|GBP1|TNC|IL1RN|IDO1|MMP12|SERPINE2|TGFBI|THBS1|RGCC|CD274|PAG1|B4GALNT2 -2.29906217 0 91 1 1 -4.005600698 3.324631082

1 -2.223310459 GO:0022408 M1 1 0 GO Biological Processes 19 negative regulation of cell-cell adhesion -2.223310459 2.965279423 3.26221829 28162 189 402 8 1.990049751 0.696552772 301|3557|3620|5270|28984|29126|55824|124872 ANXA1|IL1RN|IDO1|SERPINE2|RGCC|CD274|PAG1|B4GALNT2 -0.876598754 0 91 0 0 -4.005600698 3.324631082

1 -3.992476093 GO:0045596 M1 1 0 GO Biological Processes 19 negative regulation of cell differentiation -3.992476093 2.419307367 4.460732126 28162 666 402 23 5.721393035 1.158362377 301|857|1290|1942|2633|3488|3553|3627|4038|4067|4318|5068|5468|6356|6696|7068|7098|7474|10979|26585|28951|55198|64081 ANXA1|CAV1|COL5A2|EFNA1|GBP1|IGFBP5|IL1B|CXCL10|LRP4|LYN|MMP9|REG3A|PPARG|CCL11|SPP1|THRB|TLR3|WNT5A|FERMT2|GREM1|TRIB2|APPL2|PBLD -2.286927975 0 92 1 1 -3.992476093 3.220906959

1 -2.437029619 GO:0010721 M1 1 0 GO Biological Processes 19 negative regulation of cell development -2.437029619 3.220906959 3.536277913 28162 174 402 8 1.990049751 0.696552772 2633|3553|4038|6356|6696|7068|7474|55198 GBP1|IL1B|LRP4|CCL11|SPP1|THRB|WNT5A|APPL2 -1.045185411 0 92 0 0 -3.992476093 3.220906959

1 -3.968766049 GO:0001960 M1 1 0 GO Biological Processes 19 negative regulation of cytokine-mediated signaling pathway -3.968766049 6.452409008 5.727664999 28162 76 402 7 1.741293532 0.652391792 857|3557|4321|5468|6091|7837|11240 CAV1|IL1RN|MMP12|PPARG|ROBO1|PXDN|PADI2 -2.269113421 0 93 1 1 -3.968766049 21.01641791

1 -3.793694029 GO:0060761 M1 1 0 GO Biological Processes 19 negative regulation of response to cytokine stimulus -3.793694029 6.054112155 5.481613763 28162 81 402 7 1.741293532 0.652391792 857|3557|4321|5468|6091|7837|11240 CAV1|IL1RN|MMP12|PPARG|ROBO1|PXDN|PADI2 -2.119623389 0 93 0 0 -3.968766049 21.01641791

1 -3.612406164 GO:0001959 M1 1 0 GO Biological Processes 19 regulation of cytokine-mediated signaling pathway -3.612406164 3.957894145 4.750441095 28162 177 402 10 2.487562189 0.776790586 834|857|1906|3557|4321|5468|6091|7474|7837|11240 CASP1|CAV1|EDN1|IL1RN|MMP12|PPARG|ROBO1|WNT5A|PXDN|PADI2 -1.972814426 0 93 0 0 -3.968766049 21.01641791

1 -3.492744975 GO:0070099 M1 1 0 GO Biological Processes 19 regulation of chemokine-mediated signaling pathway -3.492744975 21.01641791 7.618317439 28162 10 402 3 0.746268657 0.429247719 1906|6091|11240 EDN1|ROBO1|PADI2 -1.882719663 0 93 0 0 -3.968766049 21.01641791

1 -3.385559399 GO:0060759 M1 1 0 GO Biological Processes 19 regulation of response to cytokine stimulus -3.385559399 3.706599279 4.492754347 28162 189

402 10 2.487562189 0.776790586
834|857|1906|3557|4321|5468|6091|7474|7837|11240
CASP1|CAV1|EDN1|IL1RN|MMP12|PPARG|ROBO1|WNT5A|PXDN|PADI2
-1.788825865 0 93 0 0 -3.968766049 21.01641791
1 -2.282036554 GO:0001961 M1 1 0 GO Biological Processes 19 positive regulation of cytokine-mediated signaling pathway -2.282036554 5.718753173 3.978313804
28162 49 402 4 0.995024876 0.495031064 834|1906|4321|7474
CASP1|EDN1|MMP12|WNT5A -0.923653081 0 93 0 0 -3.968766049
21.01641791
1 -2.07688532 GO:0060760 M1 1 0 GO Biological Processes 19 positive regulation of response to cytokine stimulus -2.07688532 5.003909026 3.609159088 28162 56
402 4 0.995024876 0.495031064 834|1906|4321|7474
CASP1|EDN1|MMP12|WNT5A -0.762296437 0 93 0 0 -3.968766049
21.01641791
1 -2.055117446 GO:0110110 M1 1 0 GO Biological Processes 19 positive regulation of animal organ morphogenesis -2.055117446 7.005472637 3.96036166 28162 30
402 3 0.746268657 0.429247719 1906|6091|7474 EDN1|ROBO1|WNT5A
-0.74572497 0 93 0 0 -3.968766049 21.01641791
1 -3.939382329 GO:0002020 M1 1 0 GO Molecular Functions 21 protease binding
-3.939382329 4.812920132 5.263824395 28162 131 402 9 2.23880597
0.737867616 1277|1278|1281|5272|6402|7076|7450|57126|91319
COL1A1|COL1A2|COL3A1|SERPINB9|SELL|TIMP1|VWF|CD177|DERL3 -2.243616031 0
94 1 1 -3.939382329 4.812920132
1 -2.337419413 GO:0007229 M1 1 0 GO Biological Processes 19 integrin-mediated signaling pathway -2.337419413 3.891929243 3.6235101 28162 108 402 6
1.492537313 0.604761504 1281|7076|9510|10451|10979|57126
COL3A1|TIMP1|ADAMTS1|VAV3|FERMT2|CD177 -0.962996398 0 94 0 0
-3.939382329 4.812920132
1 -3.930474176 GO:0007200 M1 1 0 GO Biological Processes 19 phospholipase C-activating G protein-coupled receptor signaling pathway -3.930474176 5.441143796
5.433812954 28162 103 402 8 1.990049751 0.696552772
183|1906|2151|2357|2769|3579|5028|7052
AGT|EDN1|F2RL2|FPR1|GNA15|CXCR2|P2RY1|TGM2 -2.235674051 0 95 1 1
-3.930474176 8.491481984
1 -2.918524038 GO:0051482 M1 1 0 GO Biological Processes 19 positive regulation of cytosolic calcium ion concentration involved in phospholipase C-activating G protein-coupled signaling pathway -2.918524038 8.491481984 5.181731372 28162 33 402 4
0.995024876 0.495031064 1906|2151|2769|7052 EDN1|F2RL2|GNA15|TGM2
-1.414988576 0 95 0 0 -3.930474176 8.491481984
1 -2.736256654 GO:0007188 M1 1 0 GO Biological Processes 19 adenylate cyclase-modulating G protein-coupled receptor signaling pathway -2.736256654
3.045857668 3.749001805 28162 230 402 10 2.487562189 0.776790586
1906|2357|2769|2869|3627|4283|5028|6373|57211|64123
EDN1|FPR1|GNA15|GRK5|CXCL10|CXCL9|P2RY1|CXCL11|ADGRG6|ADGRL4 -1.27473474

0 95 0 0 -3.930474176 8.491481984
1 -3.879762513 GO:0018149 M1 1 0 GO Biological Processes 19 peptide cross-linking -3.879762513 10.00781805 6.416811075 28162 35 402 5 1.243781095 0.552765814 301|1281|5266|7052|7057 ANXA1|COL3A1|PI3|TGM2|THBS1 -2.188805728 0 96 1 1 -3.879762513 10.00781805
1 -2.432171716 GO:0015909 M1 1 0 GO Biological Processes 19 long-chain fatty acid transport -2.432171716 4.864911553 3.951457091 28162 72 402 5 1.243781095 0.552765814 301|5320|5468|7057|84647 ANXA1|PLA2G2A|PPARG|THBS1|PLA2G12B-1.041287275 0 96 0 0 -3.879762513 10.00781805
1 -2.220014968 GO:0043277 M1 1 0 GO Biological Processes 19 apoptotic cell clearance -2.220014968 5.494488343 3.865934849 28162 51 402 4 0.995024876 0.495031064 301|718|7052|7057 ANXA1|C3|TGM2|THBS1 -0.8750318 0 96 0 0 -3.879762513 10.00781805
1 -2.096321068 GO:0050482 M1 1 0 GO Biological Processes 19 arachidonic acid secretion -2.096321068 7.247040659 4.050350813 28162 29 402 3 0.746268657 0.429247719 301|5320|84647 ANXA1|PLA2G2A|PLA2G12B -0.778908152 0 96 0 0 -3.879762513 10.00781805
1 -2.096321068 GO:1903963 M1 1 0 GO Biological Processes 19 arachidonate transport -2.096321068 7.247040659 4.050350813 28162 29 402 3 0.746268657 0.429247719 301|5320|84647 ANXA1|PLA2G2A|PLA2G12B -0.778908152 0 96 0 0 -3.879762513 10.00781805
1 -3.846387332 GO:0042176 M1 1 0 GO Biological Processes 19 regulation of protein catabolic process -3.846387332 2.926568203 4.56828093 28162 383 402 16 3.980099502 0.975022334 722|725|857|1942|2697|3553|4478|4843|5270|7076|7474|27074|28951|79888|90865|282679 C4BPA|C4BPB|CAV1|EFNA1|GJA1|IL1B|MSN|NOS2|SERPINE2|TIMP1|WNT5A|LAMP3|TRIB2|LPCAT1|IL33|AQP11 -2.161132525 0 97 1 1 -3.846387332 3.099766653
1 -2.792355347 GO:0045732 M1 1 0 GO Biological Processes 19 positive regulation of protein catabolic process -2.792355347 3.099766653 3.813910303 28162 226 402 10 2.487562189 0.776790586 722|725|857|2697|3553|4478|7474|28951|79888|90865 C4BPA|C4BPB|CAV1|GJA1|IL1B|MSN|WNT5A|TRIB2|LPCAT1|IL33 -1.312399071 0 97 0 0 -3.846387332 3.099766653
1 -2.64036348 GO:0009896 M1 1 0 GO Biological Processes 19 positive regulation of catabolic process -2.64036348 2.329979813 3.426372318 28162 451 402 15 3.731343284 0.945283402 722|725|857|957|2697|3553|4478|5209|7474|8671|10346|11040|28951|79888|90865 C4BPA|C4BPB|CAV1|ENTPD5|GJA1|IL1B|MSN|PFKFB3|WNT5A|SLC4A4|TRIM22|PIM2|TRIB2|LPCAT1|IL33 -1.198146722 0 97 0 0 -3.846387332 3.099766653
1 -3.82384146 GO:0097305 M1 1 0 GO Biological Processes 19 response to alcohol -3.82384146 3.59255007 4.792283428 28162 234 402 12 2.985074627

0.848757934 687|2263|3248|3284|3371|3490|5468|6279|6338|6678|9076|54762 KLF9|FGFR2|HPGD|HSD3B2|TNC|IGFBP7|PPARG|S100A8|SCNN1B|SPARC|CLDN1|GRAMD1C -2.143576956 0 98 1 1 -3.82384146 3.59255007

1 -2.002996701 GO:0045471 M1 1 0 GO Biological Processes 19 response to ethanol -2.002996701 3.309672112 3.139274141 28162 127 402 6 1.492537313 0.604761504 2263|3248|3371|6279|6678|9076 FGFR2|HPGD|TNC|S100A8|SPARC|CLDN1 -0.703037236 0 98 0 0 -3.82384146 3.59255007

1 -3.823203677 GO:0006897 M1 1 0 GO Biological Processes 19 endocytosis -3.823203677 2.355641384 4.319243032 28162 684 402 23 5.721393035 1.158362377 718|722|725|857|1755|2690|3426|3579|4017|4038|5468|6678|7057|7436|7474|11031|23433|26585|54492|55198|57126|58480|81693 C3|C4BPA|C4BPB|CAV1|DMBT1|GHR|CFI|CXCR2|LOXL2|LRP4|PPARG|SPARC|THBS1|VLDLR|WNT5A|RAB31|RHOQ|GREM1|NEURL1B|APPL2|CD177|RHOU|AMN -2.143576956 0 99 1 1 -3.823203677 2.355641384

1 -3.81449367 GO:1901164 M1 1 0 GO Biological Processes 19 negative regulation of trophoblast cell migration -3.81449367 26.27052239 8.602337788 28162 8 402 3 0.746268657 0.429247719 2697|7076|130399 GJA1|TIMP1|ACVR1C -2.137653874 0 100 1 1 -3.81449367 26.27052239

1 -2.936968961 GO:1901163 M1 1 0 GO Biological Processes 19 regulation of trophoblast cell migration -2.936968961 14.01094527 6.065488101 28162 15 402 3 0.746268657 0.429247719 2697|7076|130399 GJA1|TIMP1|ACVR1C -1.429689558 0 100 0 0 -3.81449367 26.27052239

1 -2.85139329 GO:0061450 M1 1 0 GO Biological Processes 19 trophoblast cell migration -2.85139329 13.13526119 5.842895378 28162 16 402 3 0.746268657 0.429247719 2697|7076|130399 GJA1|TIMP1|ACVR1C -1.363728798 0 100 0 0 -3.81449367 26.27052239

1 -2.316401446 GO:0009743 M1 1 0 GO Biological Processes 19 response to carbohydrate -2.316401446 2.840056474 3.312239795 28162 222 402 9 2.23880597 0.737867616 1292|2697|4067|4199|5105|6678|7057|10891|130399 COL6A2|GJA1|LYN|ME1|PCK1|SPARC|THBS1|PPARGC1A|ACVR1C -0.947537787 0 100 0 0 -3.81449367 26.27052239

1 -2.066596041 GO:2000241 M1 1 0 GO Biological Processes 19 regulation of reproductive process -2.066596041 3.045857668 3.132772915 28162 161 402 7 1.741293532 0.652391792 994|1672|2697|5028|7076|7474|130399 CDC25B|DEFB1|GJA1|P2RY1|TIMP1|WNT5A|ACVR1C -0.752810664 0 100 0 0 -3.81449367 26.27052239

1 -2.050101368 GO:2000242 M1 1 0 GO Biological Processes 19 negative regulation of reproductive process -2.050101368 4.916121149 3.561468304 28162 57 402 4 0.995024876 0.495031064 2697|7076|7474|130399 GJA1|TIMP1|WNT5A|ACVR1C -0.742295357 0 100 0 0 -3.81449367 26.27052239

1 -3.662722038 GO:0006767 M1 1 0 GO Biological Processes 19 water-soluble

vitamin metabolic process -3.662722038 5.76921276 5.299173525 28162 85 402
7 1.741293532 0.652391792 6947|8876|9963|27010|29968|80704|81693
TCN1|VNN1|SLC23A1|TPK1|PSAT1|SLC19A3|AMN -2.008436349 0 101 1 1
-3.662722038 8.339848377
1 -3.496939014 GO:0051180 M1 1 0 GO Biological Processes 19 vitamin transport
-3.496939014 8.339848377 5.728374563 28162 42 402 5 1.243781095
0.552765814 6947|9429|9963|80704|81693 TCN1|ABCG2|SLC23A1|SLC19A3|AMN
-1.882719663 0 101 0 0 -3.662722038 8.339848377
1 -2.550419855 GO:0006766 M1 1 0 GO Biological Processes 19 vitamin metabolic
process -2.550419855 3.743382325 3.787302725 28162 131 402 7 1.741293532
0.652391792 6947|8876|9963|27010|29968|80704|81693
TCN1|VNN1|SLC23A1|TPK1|PSAT1|SLC19A3|AMN -1.127203212 0 101 0 0
-3.662722038 8.339848377
1 -2.055117446 GO:0090482 M1 1 0 GO Molecular Functions 21 vitamin
transmembrane transporter activity -2.055117446 7.005472637 3.96036166 28162
30 402 3 0.746268657 0.429247719 9429|9963|80704 ABCG2|SLC23A1|SLC19A3
-0.74572497 0 101 0 0 -3.662722038 8.339848377
1 -3.616269186 GO:0005911 M1 1 0 GO Cellular Components20 cell-cell junction
-3.616269186 2.599969226 4.277109056 28162 485 402 18 4.47761194
1.031484687
288|301|360|1001|1009|2697|4067|5175|6282|9073|9076|10979|23650|23705|79983
|137075|143098|284217
ANK3|ANXA1|AQP3|CDH3|CDH11|GJA1|LYN|PECAM1|S100A11|CLDN8|CLDN1|FERMT2|
TRIM29|CADM1|POF1B|CLDN23|MPP7|LAMA1 -1.973264506 0 102 1 1
-3.616269186 3.665654287
1 -3.066841312 GO:0005912 M1 1 0 GO Cellular Components20 adherens junction
-3.066841312 3.665654287 4.219820042 28162 172 402 9 2.23880597
0.737867616 301|1001|1009|4067|6282|10979|23650|79983|143098
ANXA1|CDH3|CDH11|LYN|S100A11|FERMT2|TRIM29|POF1B|MPP7 -1.527714541 0
102 0 0 -3.616269186 3.665654287
1 -2.340166283 GO:0019898 M1 1 0 GO Cellular Components20 extrinsic
component of membrane -2.340166283 2.534874967 3.237890517 28162 304 402
11 2.736318408 0.813665012
301|1001|1009|1672|1755|2769|4067|5270|9397|10979|27163
ANXA1|CDH3|CDH11|DEFB1|DMBT1|GNA15|LYN|SERPINE2|NMT2|FERMT2|NAAA
-0.965281498 0 102 0 0 -3.616269186 3.665654287
1 -3.593035205 GO:0016328 M1 1 0 GO Cellular Components20 lateral plasma
membrane -3.593035205 6.779489649 5.482258467 28162 62 402 6
1.492537313 0.604761504 288|301|6566|9076|23255|143098
ANK3|ANXA1|SLC16A1|CLDN1|MTCL1|MPP7 -1.960189924 0 103 1 1
-3.593035205 6.779489649
1 -3.505643507 GO:0048471 M1 1 0 GO Cellular Components20 perinuclear region
of cytoplasm -3.505643507 2.237859315 4.049085889 28162 720 402 23
5.721393035 1.158362377

240|762|857|1116|2040|4067|4478|4843|5320|5468|5801|5967|6696|7873|10410|10562|10966|10970|11005|27074|64764|83483|282679
ALOX5|CA4|CAV1|CHI3L1|STOM|LYN|MSN|NOS2|PLA2G2A|PPARG|PTPRR|REG1A|SPP1|MANF|IFITM3|OLFM4|RAB40B|CKAP4|SPINK5|LAMP3|CREB3L2|PLVAP|AQP11
-1.885984651 0 104 1 1 -3.505643507 2.237859315
1 -3.497851435 GO:0120254 M1 1 0 GO Biological Processes 19 olefinic compound metabolic process -3.497851435 4.709561437 4.879902498 28162 119 402 8 1.990049751 0.696552772 51|240|1555|2053|10891|29785|60481|195814
ACOX1|ALOX5|CYP2B6|EPHX2|PPARGC1A|CYP2S1|ELOVL5|SDR16C5 -1.882719663 0 105 1 1 -3.497851435 10.50820896
1 -3.473235883 GO:0001676 M1 1 0 GO Biological Processes 19 long-chain fatty acid metabolic process -3.473235883 4.670315091 4.848605077 28162 120 402 8 1.990049751 0.696552772 51|240|1555|2053|2182|3248|29785|60481
ACOX1|ALOX5|CYP2B6|EPHX2|ACSL4|HPGD|CYP2S1|ELOVL5 -1.865585934 0 105 0 0 -3.497851435 10.50820896
1 -2.561047129 GO:0019373 M1 1 0 GO Biological Processes 19 epoxygenase P450 pathway -2.561047129 10.50820896 5.118745859 28162 20 402 3 0.746268657 0.429247719 1555|2053|29785 CYP2B6|EPHX2|CYP2S1 -1.137313776 0 105 0 0 -3.497851435 10.50820896
1 -3.492744975 GO:0004955 M1 1 0 GO Molecular Functions 21 prostaglandin receptor activity -3.492744975 21.01641791 7.618317439 28162 10 402 3 0.746268657 0.429247719 3248|5468|5729 HPGD|PPARG|PTGDR -1.882719663 0 106 1 1 -3.492744975 21.01641791
1 -3.359059655 GO:0004954 M1 1 0 GO Molecular Functions 21 prostanoid receptor activity -3.359059655 19.10583446 7.227619926 28162 11 402 3 0.746268657 0.429247719 3248|5468|5729 HPGD|PPARG|PTGDR -1.767683125 0 106 0 0 -3.492744975 21.01641791
1 -2.936968961 GO:0004953 M1 1 0 GO Molecular Functions 21 icosanoid receptor activity -2.936968961 14.01094527 6.065488101 28162 15 402 3 0.746268657 0.429247719 3248|5468|5729 HPGD|PPARG|PTGDR -1.429689558 0 106 0 0 -3.492744975 21.01641791
1 -2.85139329 GO:0030728 M1 1 0 GO Biological Processes 19 ovulation -2.85139329 13.13526119 5.842895378 28162 16 402 3 0.746268657 0.429247719 3248|7130|9510 HPGD|TNFAIP6|ADAMTS1 -1.363728798 0 106 0 0 -3.492744975 21.01641791
1 -3.492744975 GO:0043208 M1 1 0 GO Molecular Functions 21 glycosphingolipid binding -3.492744975 21.01641791 7.618317439 28162 10 402 3 0.746268657 0.429247719 4067|6402|284217 LYN|SELL|LAMA1 -1.882719663 0 107 1 1 -3.492744975 21.01641791
1 -3.321648436 GO:0051861 M1 1 0 GO Molecular Functions 21 glycolipid binding -3.321648436 10.77765021 6.002294842 28162 26 402 4 0.995024876 0.495031064 4067|6402|7873|284217 LYN|SELL|MANF|LAMA1 -1.73781193 0 107 0 0 -3.492744975 21.01641791
1 -2.183845757 GO:0034110 M1 1 0 GO Biological Processes 19 regulation of

homotypic cell-cell adhesion -2.183845757 7.783858485 4.243874515 28162 27 402 3 0.746268657 0.429247719 288|4067|5270 ANK3|LYN|SERPINE2 -0.847370775 0 107 0 0 -3.492744975 21.01641791
1 -2.139188431 GO:0046625 M1 1 0 GO Molecular Functions 21 sphingolipid binding -2.139188431 7.505863539 4.144723426 28162 28 402 3 0.746268657 0.429247719 4067|6402|284217 LYN|SELL|LAMA1 -0.810292083 0 107 0 0 -3.492744975 21.01641791
1 -3.444138847 GO:0070265 M1 1 0 GO Biological Processes 19 necrotic cell death -3.444138847 6.368611488 5.254592347 28162 66 402 6 1.492537313 0.604761504 834|857|6533|7098|10562|197259 CASP1|CAV1|SLC6A6|TLR3|OLFM4|MLKL -1.841981404 0 108 1 1 -3.444138847 6.368611488
1 -2.220014968 GO:0097300 M1 1 0 GO Biological Processes 19 programmed necrotic cell death -2.220014968 5.494488343 3.865934849 28162 51 402 4 0.995024876 0.495031064 834|857|7098|197259 CASP1|CAV1|TLR3|MLKL -0.8750318 0 108 0 0 -3.444138847 6.368611488
1 -3.337930082 GO:0042100 M1 1 0 GO Biological Processes 19 B cell proliferation -3.337930082 5.108157131 4.851988182 28162 96 402 7 1.741293532 0.652391792 952|1503|3575|4067|4332|10451|51237 CD38|CTPS1|IL7R|LYN|MNDA|VAV3|MZB1 -1.750339927 0 109 1 1 -3.337930082 5.742190686
1 -2.748284387 GO:0030888 M1 1 0 GO Biological Processes 19 regulation of B cell proliferation -2.748284387 5.742190686 4.461802836 28162 61 402 5 1.243781095 0.552765814 952|4067|4332|10451|51237 CD38|LYN|MNDA|VAV3|MZB1 -1.284500518 0 109 0 0 -3.337930082 5.742190686
1 -3.325832929 GO:0007568 M1 1 0 GO Biological Processes 19 aging -3.325832929 2.995761325 4.21015184 28162 304 402 13 3.233830846 0.882281956 1030|1906|3488|4017|4316|5028|5105|6356|7076|8942|9076|10891|55532 CDKN2B|EDN1|IGFBP5|LOXL2|MMP7|P2RY1|PCK1|CCL11|TIMP1|KYNU|CLDN1|PPARGC1A|SLC30A10 -1.740498855 0 110 1 1 -3.325832929 7.374181723
1 -2.686650474 GO:0071398 M1 1 0 GO Biological Processes 19 cellular response to fatty acid -2.686650474 7.374181723 4.73156735 28162 38 402 4 0.995024876 0.495031064 1906|5166|9076|10891EDN1|PDK4|CLDN1|PPARGC1A -1.237911023 0 110 0 0 -3.325832929 7.374181723
1 -3.322744855 GO:0005769 M1 1 0 GO Cellular Components20 early endosome -3.322744855 2.729404923 4.111608151 28162 385 402 15 3.731343284 0.945283402 301|857|2697|6505|7098|9388|11031|27074|29126|54492|55198|55532|79689|149466|219285 ANXA1|CAV1|GJA1|SLC1A1|TLR3|LIPG|RAB31|LAMP3|CD274|NEURL1B|APPL2|SLC30A10|STEAP4|C1orf210|SAMD9L -1.73816021 0 111 1 1 -3.322744855 3.008485181
1 -2.038736779 GO:0031901 M1 1 0 GO Cellular Components20 early endosome

membrane -2.038736779 3.008485181 3.09469965 28162 163 402 7
1.741293532 0.652391792 301|857|6505|11031|29126|55198|79689
ANXA1|CAV1|SLC1A1|RAB31|CD274|APPL2|STEAP4 -0.732511459 0 111 0 0
-3.322744855 3.008485181
1 -3.291926051 GO:0002224 M1 1 0 GO Biological Processes 19 toll-like receptor signaling pathway -3.291926051 3.940578358 4.488751407 28162 160 402 9
2.23880597 0.737867616 857|1513|4067|6279|6280|7098|55198|64922|79931
CAV1|CTSK|LYN|S100A8|S100A9|TLR3|APPL2|LRRC19|TNIP3 -1.711811028 0 112 1
1 -3.291926051 3.940578358
1 -3.000123174 GO:0002221 M1 1 0 GO Biological Processes 19 pattern recognition receptor signaling pathway -3.000123174 3.304468225 4.052985785 28162
212 402 10 2.487562189 0.776790586
857|1513|4067|6279|6280|7098|54941|55198|64922|79931
CAV1|CTSK|LYN|S100A8|S100A9|TLR3|RNF125|APPL2|LRRC19|TNIP3 -1.4801256 0
112 0 0 -3.291926051 3.940578358
1 -3.285834385 GO:0019932 M1 1 0 GO Biological Processes 19
second-messenger-mediated signaling -3.285834385 2.966486784 4.169039121
28162 307 402 13 3.233830846 0.882281956
183|1672|1906|1908|2357|2633|2981|3579|4843|7057|55151|57211|63928
AGT|DEFB1|EDN1|EDN3|FPR1|GBP1|GUCA2B|CXCR2|NOS2|THBS1|TMEM38B|ADGRG6|CHP2 -1.70725546 0 113 1 1 -3.285834385 10.37847798
1 -3.256901045 GO:0007263 M1 1 0 GO Biological Processes 19 nitric oxide mediated signal transduction -3.256901045 10.37847798 5.867027412 28162 27 402
4 0.995024876 0.495031064 183|2357|4843|7057 AGT|FPR1|NOS2|THBS1
-1.684862738 0 113 0 0 -3.285834385 10.37847798
1 -3.285834385 GO:0070372 M1 1 0 GO Biological Processes 19 regulation of ERK1 and ERK2 cascade -3.285834385 2.966486784 4.169039121 28162 307 402 13
3.233830846 0.882281956
1116|2263|2633|3553|4067|5028|5320|5801|6356|6362|10979|55532|154043
CHI3L1|FGFR2|GBP1|IL1B|LYN|P2RY1|PLA2G2A|PTPRR|CCL11|CCL18|FERMT2|SLC30A10|CNKSR3 -1.70725546 0 114 1 1 -3.285834385 3.096341497
1 -3.03289998 GO:0070371 M1 1 0 GO Biological Processes 19 ERK1 and ERK2 cascade -3.03289998 2.785050284 3.907113701 28162 327 402 13 3.233830846
0.882281956
1116|2263|2633|3553|4067|5028|5320|5801|6356|6362|10979|55532|154043
CHI3L1|FGFR2|GBP1|IL1B|LYN|P2RY1|PLA2G2A|PTPRR|CCL11|CCL18|FERMT2|SLC30A10|CNKSR3 -1.500806012 0 114 0 0 -3.285834385 3.096341497
1 -2.407482958 GO:1902532 M1 1 0 GO Biological Processes 19 negative regulation of intracellular signal transduction -2.407482958 2.135001185 3.159059815
28162 525 402 16 3.980099502 0.975022334
183|857|1942|2633|3553|4067|4318|5270|5801|7057|8876|9912|23035|54941|79931|154043
AGT|CAV1|EFNA1|GBP1|IL1B|LYN|MMP9|SERPINE2|PTPRR|THBS1|VNN1|ARHGAP44|PHLPP2|RNF125|TNIP3|CNKSR3 -1.021446766 0 114 0 0 -3.285834385 3.096341497

1 -2.334243824 GO:0043409 M1 1 0 GO Biological Processes 19 negative regulation of MAPK cascade -2.334243824 3.096341497 3.40484256 28162 181 402 8 1.990049751 0.696552772 183|857|1942|2633|3553|4067|5801|154043 AGT|CAV1|EFNA1|GBP1|IL1B|LYN|PTPRR|CNKSR3 -0.960282089 0 114 0 0 -3.285834385 3.096341497

1 -2.197706586 GO:0001933 M1 1 0 GO Biological Processes 19 negative regulation of protein phosphorylation -2.197706586 2.315858723 3.036514235 28162 363 402 12 2.985074627 0.848757934 183|857|1030|3553|4067|5570|7453|10891|26585|28951|64081|154043 AGT|CAV1|CDKN2B|IL1B|LYN|PKIB|WARS1|PPARGC1A|GREM1|TRIB2|PBLD|CNKSR3 -0.855303393 0 114 0 0 -3.285834385 3.096341497

1 -3.266227724 GO:0007528 M1 1 0 GO Biological Processes 19 neuromuscular junction development -3.266227724 7.452630465 5.327752128 28162 47 402 5 1.243781095 0.552765814 288|1134|1282|3371|4038 ANK3|CHRNA1|COL4A1|TNC|LRP4 -1.691269779 0 115 1 1 -3.266227724 7.452630465

1 -3.18183375 GO:0043616 M1 1 0 GO Biological Processes 19 keratinocyte proliferation -3.18183375 7.148441466 5.183663705 28162 49 402 5 1.243781095 0.552765814 687|1001|2263|5068|195814 KLF9|CDH3|FGFR2|REG3A|SDR16C5 -1.621281586 0 116 1 1 -3.18183375 7.374181723

1 -3.143910417 GO:0050679 M1 1 0 GO Biological Processes 19 positive regulation of epithelial cell proliferation -3.143910417 3.450971742 4.217478119 28162 203 402 10 2.487562189 0.776790586 1001|2263|3373|3915|4321|5068|5967|6356|7474|9076 CDH3|FGFR2|HYAL1|LAMC1|MMP12|REG3A|REG1A|CCL11|WNT5A|CLDN1 -1.588289468 0 116 0 0 -3.18183375 7.374181723

1 -2.686650474 GO:0010837 M1 1 0 GO Biological Processes 19 regulation of keratinocyte proliferation -2.686650474 7.374181723 4.73156735 28162 38 402 4 0.995024876 0.495031064 687|1001|2263|5068 KLF9|CDH3|FGFR2|REG3A -1.237911023 0 116 0 0 -3.18183375 7.374181723

1 -3.171699376 GO:0051051 M1 1 0 GO Biological Processes 19 negative regulation of transport -3.171699376 2.541668077 3.926748259 28162 441 402 16 3.980099502 0.975022334 288|301|857|1906|2697|3553|3598|4318|5028|5270|7032|7057|10008|55198|91319|130399 ANK3|ANXA1|CAV1|EDN1|GJA1|IL1B|IL13RA2|MMP9|P2RY1|SERPINE2|TFF2|THBS1|KCNE3|APPL2|DERL3|ACVR1C -1.61326745 0 117 1 1 -3.171699376 15.01172708

1 -3.029274715 GO:0010649 M1 1 0 GO Biological Processes 19 regulation of cell communication by electrical coupling -3.029274715 15.01172708 6.31043428 28162 14 402 3 0.746268657 0.429247719 288|857|2697ANK3|CAV1|GJA1 -1.500806012 0 117 0 0 -3.171699376 15.01172708

1 -2.771670055 GO:1903817 M1 1 0 GO Biological Processes 19 negative regulation of voltage-gated potassium channel activity -2.771670055 12.36259877

5.639347249 28162 17 402 3 0.746268657 0.429247719 288|857|10008
ANK3|CAV1|KCNE3-1.303326622 0 117 0 0 -3.171699376 15.01172708
1 -2.766018436 GO:0034766 M1 1 0 GO Biological Processes 19 negative regulation of ion transmembrane transport -2.766018436 4.086525705 4.077400395 28162 120 402 7 1.741293532 0.652391792
288|857|3553|4318|7057|10008|55198 ANK3|CAV1|IL1B|MMP9|THBS1|KCNE3|APPL2 -1.299390454 0 117 0 0 -3.171699376 15.01172708
1 -2.745384303 GO:0034763 M1 1 0 GO Biological Processes 19 negative regulation of transmembrane transport -2.745384303 4.052752765 4.049625865 28162 121 402 7 1.741293532 0.652391792 288|857|3553|4318|7057|10008|55198 ANK3|CAV1|IL1B|MMP9|THBS1|KCNE3|APPL2 -1.283298004 0 117 0 0 -3.171699376 15.01172708
1 -2.65563091 GO:0032507 M1 1 0 GO Biological Processes 19 maintenance of protein location in cell -2.65563091 5.473025498 4.31102944 28162 64 402 5 1.243781095 0.552765814 288|857|2697|11015|85477ANK3|CAV1|GJA1|KDELR3|SCIN -1.210708258 0 117 0 0 -3.171699376 15.01172708
1 -2.63103803 GO:0043271 M1 1 0 GO Biological Processes 19 negative regulation of ion transport -2.63103803 2.627052239 3.522521101 28162 320 402 12 2.985074627 0.848757934
288|301|857|3553|4318|5028|5270|7057|10008|55198|91319|130399
ANK3|ANXA1|CAV1|IL1B|MMP9|P2RY1|SERPINE2|THBS1|KCNE3|APPL2|DERL3|ACVR1C -1.189898927 0 117 0 0 -3.171699376 15.01172708
1 -2.495668792 GO:0042383 M1 1 0 GO Cellular Components20 sarcolemma -2.495668792 3.659575258 3.713607163 28162 134 402 7 1.741293532 0.652391792 288|301|682|857|1292|1293|3752
ANK3|ANXA1|BSG|CAV1|COL6A2|COL6A3|KCND3 -1.086175878 0 117 0 0 -3.171699376 15.01172708
1 -2.477774174 GO:0001508 M1 1 0 GO Biological Processes 19 action potential -2.477774174 3.632467293 3.689511713 28162 135 402 7 1.741293532 0.652391792 288|857|1134|2697|2769|3752|10008
ANK3|CAV1|CHRNA1|GJA1|GNA15|KCND3|KCNE3 -1.075230121 0 117 0 0 -3.171699376 15.01172708
1 -2.347135782 GO:2001258 M1 1 0 GO Biological Processes 19 negative regulation of cation channel activity -2.347135782 5.962104372 4.097067432 28162 47 402 4 0.995024876 0.495031064 288|857|4318|10008
ANK3|CAV1|MMP9|KCNE3 -0.971325982 0 117 0 0 -3.171699376 15.01172708
1 -2.331508216 GO:0086001 M1 1 0 GO Biological Processes 19 cardiac muscle cell action potential -2.331508216 4.608863577 3.791051179 28162 76 402 5 1.243781095 0.552765814 288|857|2697|3752|10008
ANK3|CAV1|GJA1|KCND3|KCNE3 -0.95976962 0 117 0 0 -3.171699376 15.01172708
1 -2.279112201 GO:1901017 M1 1 0 GO Biological Processes 19 negative regulation of potassium ion transmembrane transporter activity -2.279112201 8.406567164 4.458362801 28162 25 402 3 0.746268657 0.429247719 288|857|10008

ANK3|CAV1|KCNE3-0.923653081 0 117 0 0 -3.171699376 15.01172708
1 -2.139188431 GO:0086011 M1 1 0 GO Biological Processes 19 membrane repolarization during action potential -2.139188431 7.505863539 4.144723426 28162 28 402 3 0.746268657 0.429247719 857|3752|10008 CAV1|KCND3|KCNE3 -0.810292083 0 117 0 0 -3.171699376 15.01172708
1 -2.132224823 GO:0086002 M1 1 0 GO Biological Processes 19 cardiac muscle cell action potential involved in contraction -2.132224823 5.18923899 3.70803971 28162 54 402 4 0.995024876 0.495031064 857|2697|3752|10008 CAV1|GJA1|KCND3|KCNE3 -0.805814901 0 117 0 0 -3.171699376 15.01172708
1 -2.106174654 GO:1901379 M1 1 0 GO Biological Processes 19 regulation of potassium ion transmembrane transport -2.106174654 4.072949207 3.434506341 28162 86 402 5 1.243781095 0.552765814 288|857|1908|10008|57628 ANK3|CAV1|EDN3|KCNE3|DPP10 -0.783467881 0 117 0 0 -3.171699376 15.01172708
1 -2.104946488 GO:0032409 M1 1 0 GO Biological Processes 19 regulation of transporter activity -2.104946488 2.475432027 3.001934142 28162 283 402 10 2.487562189 0.776790586 288|857|2040|2697|4318|5174|5243|5468|10008|10891 ANK3|CAV1|STOM|GJA1|MMP9|PDZK1|ABCB1|PPARG|KCNE3|PPARGC1A -0.782649234 0 117 0 0 -3.171699376 15.01172708
1 -2.096321068 GO:1901380 M1 1 0 GO Biological Processes 19 negative regulation of potassium ion transmembrane transport -2.096321068 7.247040659 4.050350813 28162 29 402 3 0.746268657 0.429247719 288|857|10008 ANK3|CAV1|KCNE3-0.778908152 0 117 0 0 -3.171699376 15.01172708
1 -2.015464226 GO:0045907 M1 1 0 GO Biological Processes 19 positive regulation of vasoconstriction -2.015464226 6.779489649 3.8744056 28162 31 402 3 0.746268657 0.429247719 857|952|2697CAV1|CD38|GJA1 -0.713946752 0 117 0 0 -3.171699376 15.01172708
1 -3.130595566 GO:0031589 M1 1 0 GO Biological Processes 19 cell-substrate adhesion -3.130595566 2.72435047 3.962471053 28162 360 402 14 3.482587065 0.914409651 1277|1281|1942|2633|3915|4321|4811|5328|7057|7450|10562|10979|26585|56999 COL1A1|COL3A1|EFNA1|GBP1|LAMC1|MMP12|NID1|PLAU|THBS1|VWF|OLFM4|FERMT2|GREM1|ADAMTS9 -1.577269892 0 118 1 1 -3.130595566 2.892167602
1 -2.367431958 GO:0010810 M1 1 0 GO Biological Processes 19 regulation of cell-substrate adhesion -2.367431958 2.892167602 3.374977599 28162 218 402 9 2.23880597 0.737867616 1277|2633|4321|4811|5328|7057|10562|10979|26585 COL1A1|GBP1|MMP12|NID1|PLAU|THBS1|OLFM4|FERMT2|GREM1 -0.988835232 0 118 0 0 -3.130595566 2.892167602
1 -3.129402513 GO:0071415 M1 1 0 GO Biological Processes 19 cellular response to purine-containing compound -3.129402513 16.16647532 6.581913723 28162 13 402 3 0.746268657 0.429247719 5028|10891|55151 P2RY1|PPARGC1A|TMEM38B -1.577269892 0 119 1 1 -3.129402513 16.16647532
1 -2.918524038 GO:0046320 M1 1 0 GO Biological Processes 19 regulation of fatty

acid oxidation	-2.918524038 8.491481984 5.181731372 28162 33 402 4
0.995024876 0.495031064 5166|5468|10891|55198 PDK4|PPARG|PPARGC1A|APPL2
-1.414988576 0 119 0 0 -3.129402513 16.16647532
1 -2.771670055 GO:0031000 M1 1 0 GO Biological Processes 19 response to caffeine -2.771670055 12.36259877 5.639347249 28162 17 402 3 0.746268657
0.429247719 5468|10891|55151 PPARG|PPARGC1A|TMEM38B -1.303326622 0
119 0 0 -3.129402513 16.16647532
1 -2.383476348 GO:0002021 M1 1 0 GO Biological Processes 19 response to dietary excess -2.383476348 9.137573005 4.698203434 28162 23 402 3
0.746268657 0.429247719 10891|55198|130399 PPARGC1A|APPL2|ACVR1C
-1.001605443 0 119 0 0 -3.129402513 16.16647532
1 -2.347135782 GO:0009409 M1 1 0 GO Biological Processes 19 response to cold
-2.347135782 5.962104372 4.097067432 28162 47 402 4 0.995024876
0.495031064 3627|5468|10891|55198 CXCL10|PPARG|PPARGC1A|APPL2
-0.971325982 0 119 0 0 -3.129402513 16.16647532
1 -2.298726562 GO:0034440 M1 1 0 GO Biological Processes 19 lipid oxidation
-2.298726562 3.821166893 3.567553857 28162 110 402 6 1.492537313
0.604761504 51|240|5166|5468|10891|55198
ACOX1|ALOX5|PDK4|PPARG|PPARGC1A|APPL2 -0.933888995 0 119 0 0
-3.129402513 16.16647532
1 -2.20627115 GO:0042593 M1 1 0 GO Biological Processes 19 glucose homeostasis -2.20627115 2.729404923 3.176045902 28162 231 402 9
2.23880597 0.737867616 240|3488|5105|5166|5468|6566|10891|25825|55198
ALOX5|IGFBP5|PCK1|PDK4|PPARG|SLC16A1|PPARGC1A|BACE2|APPL2 -0.863009667 0
119 0 0 -3.129402513 16.16647532
1 -2.194418354 GO:0033500 M1 1 0 GO Biological Processes 19 carbohydrate homeostasis -2.194418354 2.717640247 3.161317128 28162 232 402 9
2.23880597 0.737867616 240|3488|5105|5166|5468|6566|10891|25825|55198
ALOX5|IGFBP5|PCK1|PDK4|PPARG|SLC16A1|PPARGC1A|BACE2|APPL2 -0.852871757 0
119 0 0 -3.129402513 16.16647532
1 -3.102896117 GO:0031225 M1 1 0 GO Cellular Components20 anchored component of membrane -3.102896117 3.708779631 4.262932961 28162 170 402
9 2.23880597 0.737867616 762|1604|1942|2215|5067|8876|11126|57126|646627
CA4|CD55|EFNA1|FCGR3B|CNTN3|VNN1|CD160|CD177|LYPD8 -1.554224028 0 120
1 1 -3.102896117 3.708779631
1 -3.090600149 GO:0019955 M1 1 0 GO Molecular Functions 21 cytokine binding
-3.090600149 4.090786941 4.363991274 28162 137 402 8 1.990049751
0.696552772 2633|2690|3557|3579|3598|7057|7837|26585
GBP1|GHR|IL1RN|CXCR2|IL13RA2|THBS1|PXDN|GREM1 -1.543304589 0 121 1 1
-3.090600149 4.090786941
1 -2.460052904 GO:0140375 M1 1 0 GO Molecular Functions 21 immune receptor activity -2.460052904 3.605757975 3.665644152 28162 136 402 7 1.741293532
0.652391792 1439|2357|2690|3575|3579|3598|11126
CSF2RB|FPR1|GHR|IL7R|CXCR2|IL13RA2|CD160-1.060908724 0 121 0 0

-3.090600149 4.090786941
1 -3.077999801 GO:0071711 M1 1 0 GO Biological Processes 19 basement membrane organization -3.077999801 9.340630182 5.500301806 28162 30 402 4 0.995024876 0.495031064 857|1282|4811|7837 CAV1|COL4A1|NID1|PXDN -1.537522191 0 122 1 1 -3.077999801 9.340630182
1 -3.062528266 GO:0002886 M1 1 0 GO Biological Processes 19 regulation of myeloid leukocyte mediated immunity -3.062528266 6.736031382 4.982073148 28162 52 402 5 1.243781095 0.552765814 718|3598|4067|6372|57126 C3|IL13RA2|LYN|CXCL6|CD177 -1.524748145 0 123 1 1 -3.062528266 6.736031382
1 -2.250657086 GO:0002448 M1 1 0 GO Biological Processes 19 mast cell mediated immunity -2.250657086 5.604378109 3.921366574 28162 50 402 4 0.995024876 0.495031064 3598|4067|5272|5729 IL13RA2|LYN|SERPINB9|PTGDR -0.89915612 0 123 0 0 -3.062528266 6.736031382
1 -3.029274715 GO:0030213 M1 1 0 GO Biological Processes 19 hyaluronan biosynthetic process -3.029274715 15.01172708 6.31043428 28162 14 402 3 0.746268657 0.429247719 3373|3553|57214 HYAL1|IL1B|CEMIP -1.500806012 0 124 1 1 -3.029274715 15.01172708
1 -2.055117446 GO:0010800 M1 1 0 GO Biological Processes 19 positive regulation of peptidyl-threonine phosphorylation -2.055117446 7.005472637 3.96036166 28162 30 402 3 0.746268657 0.429247719 1116|7474|57214 CHI3L1|WNT5A|CEMIP -0.74572497 0 124 0 0 -3.029274715 15.01172708
1 -3.025884506 GO:0001776 M1 1 0 GO Biological Processes 19 leukocyte homeostasis -3.025884506 5.320612129 4.627691592 28162 79 402 6 1.492537313 0.604761504 301|608|4067|6372|23657|124976 ANXA1|TNFRSF17|LYN|CXCL6|SLC7A11|SPNS2 -1.499355638 0 125 1 1 -3.025884506 5.320612129
1 -2.475183944 GO:0048872 M1 1 0 GO Biological Processes 19 homeostasis of number of cells -2.475183944 2.802189055 3.444309788 28162 250 402 10 2.487562189 0.776790586 301|608|3575|4067|5320|6372|11167|23657|64922|124976 ANXA1|TNFRSF17|IL7R|LYN|PLA2G2A|CXCL6|FSTL1|SLC7A11|LRRC19|SPNS2 -1.073132009 0 125 0 0 -3.025884506 5.320612129
1 -2.987400134 GO:0006968 M1 1 0 GO Biological Processes 19 cellular defense response -2.987400134 6.486548738 4.856333247 28162 54 402 5 1.243781095 0.552765814 3579|4283|4332|4688|90865 CXCR2|CXCL9|MNDA|NCF2|IL33 -1.469334192 0 126 1 1 -2.987400134 6.486548738
1 -2.936968961 GO:0072672 M1 1 0 GO Biological Processes 19 neutrophil extravasation -2.936968961 14.01094527 6.065488101 28162 15 402 3 0.746268657 0.429247719 5175|10562|57126 PECAM1|OLFM4|CD177 -1.429689558 0 127 1 1 -2.936968961 14.01094527
1 -2.458411739 GO:0045123 M1 1 0 GO Biological Processes 19 cellular extravasation -2.458411739 4.933431434 3.99341542 28162 71 402 5 1.243781095 0.552765814 5175|6402|10562|57126|83483

PECAM1|SELL|OLFM4|CD177|PLVAP -1.060276805 0 127 0 0 -2.936968961
14.01094527
1 -2.915486499 GO:0042743 M1 1 0 GO Biological Processes 19 hydrogen peroxide metabolic process -2.915486499 6.254886283 4.736801076 28162 56 402
5 1.243781095 0.552765814 51|4314|7837|50506|405753
ACOX1|MMP3|PXDN|DUOX2|DUOXA2 -1.413810972 0 128 1 1
-2.915486499 12.36259877
1 -2.771670055 GO:0050665 M1 1 0 GO Biological Processes 19 hydrogen peroxide biosynthetic process -2.771670055 12.36259877 5.639347249 28162 17
402 3 0.746268657 0.429247719 51|50506|405753 ACOX1|DUOX2|DUOXA2
-1.303326622 0 128 0 0 -2.915486499 12.36259877
1 -2.498684669 GO:0006590 M1 1 0 GO Biological Processes 19 thyroid hormone generation -2.498684669 10.00781805 4.969203948 28162 21 402 3
0.746268657 0.429247719 1513|50506|405753 CTSK|DUOX2|DUOXA2
-1.087688138 0 128 0 0 -2.915486499 12.36259877
1 -2.230434181 GO:0042403 M1 1 0 GO Biological Processes 19 thyroid hormone metabolic process -2.230434181 8.083237658 4.348251364 28162 26 402 3
0.746268657 0.429247719 1513|50506|405753 CTSK|DUOX2|DUOXA2
-0.882189587 0 128 0 0 -2.915486499 12.36259877
1 -2.911810018 GO:0072562 M1 1 0 GO Cellular Components20 blood microparticle -2.911810018 3.838615143 4.138159355 28162 146 402 8
1.990049751 0.696552772 12|183|629|716|718|722|2040|4478
SERPINA3|AGT|CFB|C1S|C3|C4BPA|STOM|MSN-1.410752705 0 129 1 1
-2.911810018 3.838615143
1 -2.872583926 GO:0001704 M1 1 0 GO Biological Processes 19 formation of primary germ layer -2.872583926 4.264200735 4.22094704 28162 115 402 7
1.741293532 0.652391792 1290|1303|2263|2697|4313|4318|7474
COL5A2|COL12A1|FGFR2|GJA1|MMP2|MMP9|WNT5A -1.378269499 0 130 1 1
-2.872583926 6.368611488
1 -2.451174411 GO:0035987 M1 1 0 GO Biological Processes 19 endodermal cell differentiation -2.451174411 6.368611488 4.288677933 28162 44 402 4
0.995024876 0.495031064 1290|1303|4313|4318 COL5A2|COL12A1|MMP2|MMP9
-1.054984808 0 130 0 0 -2.872583926 6.368611488
1 -2.363045632 GO:0007369 M1 1 0 GO Biological Processes 19 gastrulation
-2.363045632 3.130937491 3.441734946 28162 179 402 8 1.990049751
0.696552772 1290|1303|2263|2697|3557|4313|4318|7474
COL5A2|COL12A1|FGFR2|GJA1|IL1RN|MMP2|MMP9|WNT5A -0.984914637 0 130 0
0 -2.872583926 6.368611488
1 -2.331508216 GO:0007492 M1 1 0 GO Biological Processes 19 endoderm development -2.331508216 4.608863577 3.791051179 28162 76 402 5
1.243781095 0.552765814 1290|1303|3915|4313|4318
COL5A2|COL12A1|LAMC1|MMP2|MMP9 -0.95976962 0 130 0 0
-2.872583926 6.368611488
1 -2.160825932 GO:0001706 M1 1 0 GO Biological Processes 19 endoderm

formation	-2.160825932	5.28714916	3.759336167	28162	53	402	4
0.995024876	0.495031064	1290|1303|4313|4318	COL5A2|COL12A1|MMP2|MMP9
-0.827753644	0	130	0	0	-2.872583926	6.368611488
1	-2.106174654	GO:0048013	M1	1	0	GO Biological Processes	19	ephrin receptor signaling pathway	-2.106174654	4.072949207	3.434506341	28162	86	402	5
1.243781095	0.552765814	1942|4067|4313|4318|10451
EFNA1|LYN|MMP2|MMP9|VAV3	-0.783467881	0	130	0	0	-2.872583926
6.368611488
1	-2.85139329	GO:0050664	M1	1	0	GO Molecular Functions	21	oxidoreductase activity, acting on NAD(P)H, oxygen as acceptor	-2.85139329	13.13526119	5.842895378
28162	16	402	3	0.746268657	0.429247719	2330|4688|50506
FMO5|NCF2|DUOX2	-1.363728798	0	131	1	1	-2.85139329	13.13526119
1	-2.82120143	GO:0042554	M1	1	0	GO Biological Processes	19	superoxide anion generation	-2.82120143	8.006254442	4.99097682	28162	35	402	4
0.995024876	0.495031064	1906|4688|50506|57126	EDN1|NCF2|DUOX2|CD177
-1.337106169	0	131	0	0	-2.85139329	13.13526119
1	-2.485106577	GO:0006801	M1	1	0	GO Biological Processes	19	superoxide metabolic process	-2.485106577	5.003909026	4.036167893	28162	70	402	5
1.243781095	0.552765814	1906|4688|4843|50506|57126
EDN1|NCF2|NOS2|DUOX2|CD177	-1.079101992	0	131	0	0	-2.85139329
13.13526119
1	-2.85139329	GO:0097067	M1	1	0	GO Biological Processes	19	cellular response to thyroid hormone stimulus	-2.85139329	13.13526119	5.842895378	28162	16	402
3	0.746268657	0.429247719	687|7068|10891	KLF9|THRB|PPARGC1A
-1.363728798	0	132	1	1	-2.85139329	13.13526119
1	-2.383476348	GO:0097066	M1	1	0	GO Biological Processes	19	response to thyroid hormone	-2.383476348	9.137573005	4.698203434	28162	23	402	3
0.746268657	0.429247719	687|7068|10891	KLF9|THRB|PPARGC1A	-1.001605443	0
132	0	0	-2.85139329	13.13526119
1	-2.803661304	GO:0022604	M1	1	0	GO Biological Processes	19	regulation of cell morphogenesis	-2.803661304	2.756251529	3.711049907	28162	305	402	12
2.985074627	0.848757934
301|2633|4478|5028|5967|6356|6678|7474|10562|10979|23433|58480
ANXA1|GBP1|MSN|P2RY1|REG1A|CCL11|SPARC|WNT5A|OLFM4|FERMT2|RHOQ|RHOU
-1.32252648	0	133	1	1	-2.803661304	3.247570097
1	-2.213382582	GO:0008360	M1	1	0	GO Biological Processes	19	regulation of cell shape	-2.213382582	3.247570097	3.332458457	28162	151	402	7	1.741293532
0.652391792	301|4478|5028|6356|10979|23433|58480
ANXA1|MSN|P2RY1|CCL11|FERMT2|RHOQ|RHOU	-0.869691318	0	133	0	0
-2.803661304	3.247570097
1	-2.771670055	GO:0045198	M1	1	0	GO Biological Processes	19	establishment of epithelial cell apical/basal polarity	-2.771670055	12.36259877	5.639347249	28162	17
402	3	0.746268657	0.429247719	4478|7474|284217	MSN|WNT5A|LAMA1
-1.303326622	0	134	1	1	-2.771670055	12.36259877

1 -2.52527329 GO:0045197 M1 1 0 GO Biological Processes 19 establishment or maintenance of epithelial cell apical/basal polarity -2.52527329 6.671878702 4.426609925 28162 42 402 4 0.995024876 0.495031064 4478|7474|23255|284217 MSN|WNT5A|MTCL1|LAMA1 -1.108908949 0 134 0 0 -2.771670055 12.36259877
1 -2.439596569 GO:0035089 M1 1 0 GO Biological Processes 19 establishment of apical/basal cell polarity -2.439596569 9.552917232 4.829374617 28162 22 402 3 0.746268657 0.429247719 4478|7474|284217 MSN|WNT5A|LAMA1 -1.046308721 0 134 0 0 -2.771670055 12.36259877
1 -2.347135782 GO:0035088 M1 1 0 GO Biological Processes 19 establishment or maintenance of apical/basal cell polarity -2.347135782 5.962104372 4.097067432 28162 47 402 4 0.995024876 0.495031064 4478|7474|23255|284217 MSN|WNT5A|MTCL1|LAMA1 -0.971325982 0 134 0 0 -2.771670055 12.36259877
1 -2.347135782 GO:0061245 M1 1 0 GO Biological Processes 19 establishment or maintenance of bipolar cell polarity -2.347135782 5.962104372 4.097067432 28162 47 402 4 0.995024876 0.495031064 4478|7474|23255|284217 MSN|WNT5A|MTCL1|LAMA1 -0.971325982 0 134 0 0 -2.771670055 12.36259877
1 -2.330058653 GO:0030859 M1 1 0 GO Biological Processes 19 polarized epithelial cell differentiation -2.330058653 8.756840796 4.574790579 28162 24 402 3 0.746268657 0.429247719 4478|7474|284217 MSN|WNT5A|LAMA1 -0.95976962 0 134 0 0 -2.771670055 12.36259877
1 -2.330058653 GO:0061162 M1 1 0 GO Biological Processes 19 establishment of monopolar cell polarity -2.330058653 8.756840796 4.574790579 28162 24 402 3 0.746268657 0.429247719 4478|7474|284217 MSN|WNT5A|LAMA1 -0.95976962 0 134 0 0 -2.771670055 12.36259877
1 -2.279112201 GO:0061339 M1 1 0 GO Biological Processes 19 establishment or maintenance of monopolar cell polarity -2.279112201 8.406567164 4.458362801 28162 25 402 3 0.746268657 0.429247719 4478|7474|284217 MSN|WNT5A|LAMA1 -0.923653081 0 134 0 0 -2.771670055 12.36259877
1 -2.015464226 GO:0090162 M1 1 0 GO Biological Processes 19 establishment of epithelial cell polarity -2.015464226 6.779489649 3.8744056 28162 31 402 3 0.746268657 0.429247719 4478|7474|284217 MSN|WNT5A|LAMA1 -0.713946752 0 134 0 0 -2.771670055 12.36259877
1 -2.771670055 GO:0070206 M1 1 0 GO Biological Processes 19 protein trimerization -2.771670055 12.36259877 5.639347249 28162 17 402 3 0.746268657 0.429247719 1278|79689|197259 COL1A2|STEAP4|MLKL -1.303326622 0 135 1 1 -2.771670055 12.36259877
1 -2.230202226 GO:0051259 M1 1 0 GO Biological Processes 19 protein complex oligomerization -2.230202226 2.753242521 3.205739861 28162 229 402 9 2.23880597 0.737867616 1278|3752|6999|9076|55198|79689|140803|197259|282679 COL1A2|KCND3|TDO2|CLDN1|APPL2|STEAP4|TRPM6|MLKL|AQP11 -0.882189587 0

135 0 0 -2.771670055 12.36259877
1 -2.771670055 GO:0042599 M1 1 0 GO Cellular Components20 lamellar body -2.771670055 12.36259877 5.639347249 28162 17 402 3 0.746268657 0.429247719 10970|11005|27074 CKAP4|SPINK5|LAMP3 -1.303326622 0 136 1 1 -2.771670055 12.36259877
1 -2.686650474 GO:0007618 M1 1 0 GO Biological Processes 19 mating -2.686650474 7.374181723 4.73156735 28162 38 402 4 0.995024876 0.495031064 5028|5266|5270|7068 P2RY1|PI3|SERPINE2|THRB -1.237911023 0 137 1 1 -2.686650474 11.06127258
1 -2.62704053 GO:0007620 M1 1 0 GO Biological Processes 19 copulation -2.62704053 11.06127258 5.279245426 28162 19 402 3 0.746268657 0.429247719 5028|5266|5270 P2RY1|PI3|SERPINE2 -1.187511505 0 137 0 0 -2.686650474 11.06127258
1 -2.644513742 GO:1901998 M1 1 0 GO Biological Processes 19 toxin transport -2.644513742 7.18510014 4.651312809 28162 39 402 4 0.995024876 0.495031064 834|4225|10966|29126CASP1|MEP1B|RAB40B|CD274 -1.201216649 0 138 1 1 -2.644513742 7.18510014
1 -2.627039124 GO:0055088 M1 1 0 GO Biological Processes 19 lipid homeostasis -2.627039124 3.45949266 3.777907797 28162 162 402 8 1.990049751 0.696552772 51|857|2053|5468|9388|79850|84647|148534 ACOX1|CAV1|EPHX2|PPARG|LIPG|TLCD3A|PLA2G12B|TLCD4 -1.187511505 0 139 1 1 -2.627039124 3.45949266
1 -2.621905677 GO:0019904 M1 1 0 GO Molecular Functions 21 protein domain specific binding -2.621905677 2.030571779 3.29826073 28162 690 402 20 4.975124378 1.084445172 51|834|2697|3553|4067|5174|5396|5468|6091|6338|6550|6584|7052|7453|7474|23433|23705|55824|143098|387755 ACOX1|CASP1|GJA1|IL1B|LYN|PDZK1|PRRX1|PPARG|ROBO1|SCNN1B|SLC9A3|SLC22A5|TGM2|WARS1|WNT5A|RHOQ|CADM1|PAG1|MPP7|INSC -1.183449068 0 140 1 1 -2.621905677 2.030571779
1 -2.603613649 GO:0150077 M1 1 0 GO Biological Processes 19 regulation of neuroinflammatory response -2.603613649 7.005472637 4.573844738 28162 40 402 4 0.995024876 0.495031064 3553|4314|4318|90865IL1B|MMP3|MMP9|IL33 -1.168884912 0 141 1 1 -2.603613649 7.783858485
1 -2.451174411 GO:0150076 M1 1 0 GO Biological Processes 19 neuroinflammatory response -2.451174411 6.368611488 4.288677933 28162 44 402 4 0.995024876 0.495031064 3553|4314|4318|90865IL1B|MMP3|MMP9|IL33 -1.054984808 0 141 0 0 -2.603613649 7.783858485
1 -2.183845757 GO:0002825 M1 1 0 GO Biological Processes 19 regulation of T-helper 1 type immune response -2.183845757 7.783858485 4.243874515 28162 27 402 3 0.746268657 0.429247719 301|3553|90865 ANXA1|IL1B|IL33 -0.847370775 0 141 0 0 -2.603613649 7.783858485
1 -2.055117446 GO:0002828 M1 1 0 GO Biological Processes 19 regulation of type 2 immune response -2.055117446 7.005472637 3.96036166 28162 30 402 3

0.746268657 0.429247719 301|3620|90865 ANXA1|IDO1|IL33 -0.74572497 0 141 0 0 -2.603613649 7.783858485

1 -2.569035305 GO:0005793 M1 1 0 GO Cellular Components20 endoplasmic reticulum-Golgi intermediate compartment -2.569035305 3.772177574 3.812352165 28162 130 402 7 1.741293532 0.652391792 290|762|1604|4925|6091|81671|146456 ANPEP|CA4|CD55|NUCB2|ROBO1|VMP1|TMED6 -1.141064218 0 142 1 1 -2.569035305 3.772177574

1 -2.52527329 GO:0031670 M1 1 0 GO Biological Processes 19 cellular response to nutrient -2.52527329 6.671878702 4.426609925 28162 42 402 4 0.995024876 0.495031064 1030|1277|3371|5468 CDKN2B|COL1A1|TNC|PPARG -1.108908949 0 143 1 1 -2.52527329 9.552917232

1 -2.439596569 GO:0055093 M1 1 0 GO Biological Processes 19 response to hyperoxia -2.439596569 9.552917232 4.829374617 28162 22 402 3 0.746268657 0.429247719 857|1277|5468 CAV1|COL1A1|PPARG -1.046308721 0 143 0 0 -2.52527329 9.552917232

1 -2.096321068 GO:0036296 M1 1 0 GO Biological Processes 19 response to increased oxygen levels -2.096321068 7.247040659 4.050350813 28162 29 402 3 0.746268657 0.429247719 857|1277|5468 CAV1|COL1A1|PPARG -0.778908152 0 143 0 0 -2.52527329 9.552917232

1 -2.055117446 GO:0034694 M1 1 0 GO Biological Processes 19 response to prostaglandin -2.055117446 7.005472637 3.96036166 28162 30 402 3 0.746268657 0.429247719 1906|3371|5468 EDN1|TNC|PPARG -0.74572497 0 143 0 0 -2.52527329 9.552917232

1 -2.055117446 GO:0071295 M1 1 0 GO Biological Processes 19 cellular response to vitamin -2.055117446 7.005472637 3.96036166 28162 30 402 3 0.746268657 0.429247719 1277|3371|5468 COL1A1|TNC|PPARG -0.74572497 0 143 0 0 -2.52527329 9.552917232

1 -2.498684669 GO:0016327 M1 1 0 GO Cellular Components20 apicolateral plasma membrane -2.498684669 10.00781805 4.969203948 28162 21 402 3 0.746268657 0.429247719 7056|9073|23255 THBD|CLDN8|MTCL1 -1.087688138 0 144 1 1 -2.498684669 10.00781805

1 -2.495833322 GO:0098797 M1 1 0 GO Cellular Components20 plasma membrane protein complex -2.495833322 1.978947073 3.174682939 28162 708 402 20 4.975124378 1.084445172 857|1001|1009|1134|1439|2697|2769|3108|3752|4067|4688|6338|6533|8140|10008|50506|57628|79820|130399|143098 CAV1|CDH3|CDH11|CHRNA1|CSF2RB|GJA1|GNA15|HLA-DMA|KCND3|LYN|NCF2|SCNN1B|SLC6A6|SLC7A5|KCNE3|DUOX2|DPP10|CATSPERB|ACVR1C|MPP7 -1.086175878 0 145 1 1 -2.495833322 1.978947073

1 -2.487719498 GO:0015701 M1 1 0 GO Biological Processes 19 bicarbonate transport -2.487719498 6.516718732 4.356547625 28162 43 402 4 0.995024876 0.495031064 759|762|1836|8671 CA1|CA4|SLC26A2|SLC4A4 -1.080223345 0 146 1 1 -2.487719498 6.516718732

1 -2.485106577 GO:0016529 M1 1 0 GO Cellular Components20 sarcoplasmic reticulum -2.485106577 5.003909026 4.036167893 28162 70 402 5 1.243781095 0.552765814 288|7057|7873|55151|55214 ANK3|THBS1|MANF|TMEM38B|P3H2 -1.079101992 0 147 1 1 -2.485106577 5.003909026

1 -2.260201294 GO:0016528 M1 1 0 GO Cellular Components20 sarcoplasm -2.260201294 4.433843441 3.67789747 28162 79 402 5 1.243781095 0.552765814 288|7057|7873|55151|55214 ANK3|THBS1|MANF|TMEM38B|P3H2 -0.90826231 0 147 0 0 -2.485106577 5.003909026

1 -2.415590886 GO:0006509 M1 1 0 GO Biological Processes 19 membrane protein ectodomain proteolysis -2.415590886 6.227086788 4.222880671 28162 45 402 4 0.995024876 0.495031064 3553|4316|7076|25825IL1B|MMP7|TIMP1|BACE2 -1.027841704 0 148 1 1 -2.415590886 6.227086788

1 -2.415590886 GO:0085029 M1 1 0 GO Biological Processes 19 extracellular matrix assembly -2.415590886 6.227086788 4.222880671 28162 45 402 4 0.995024876 0.495031064 183|1278|7837|28984 AGT|COL1A2|PXDN|RGCC -1.027841704 0 149 1 1 -2.415590886 6.227086788

1 -2.415590886 GO:0032570 M1 1 0 GO Biological Processes 19 response to progesterone -2.415590886 6.227086788 4.222880671 28162 45 402 4 0.995024876 0.495031064 857|952|7057|79820 CAV1|CD38|THBS1|CATSPERB -1.027841704 0 150 1 1 -2.415590886 6.227086788

1 -2.415590886 GO:1900087 M1 1 0 GO Biological Processes 19 positive regulation of G1/S transition of mitotic cell cycle -2.415590886 6.227086788 4.222880671 28162 45 402 4 0.995024876 0.495031064 301|3373|9510|28984 ANXA1|HYAL1|ADAMTS1|RGCC -1.027841704 0 151 1 1 -2.415590886 6.227086788

1 -2.07688532 GO:1902808 M1 1 0 GO Biological Processes 19 positive regulation of cell cycle G1/S phase transition -2.07688532 5.003909026 3.609159088 28162 56 402 4 0.995024876 0.495031064 301|3373|9510|28984 ANXA1|HYAL1|ADAMTS1|RGCC -0.762296437 0 151 0 0 -2.415590886 6.227086788

1 -2.005510056 GO:1901992 M1 1 0 GO Biological Processes 19 positive regulation of mitotic cell cycle phase transition -2.005510056 3.849160789 3.2759408 28162 91 402 5 1.243781095 0.552765814 301|994|3373|9510|28984 ANXA1|CDC25B|HYAL1|ADAMTS1|RGCC -0.705161612 0 151 0 0 -2.415590886 6.227086788

1 -2.408851764 GO:0016042 M1 1 0 GO Biological Processes 19 lipid catabolic process -2.408851764 2.465268963 3.275936719 28162 341 402 12 2.985074627 0.848757934 51|3553|5105|5320|6696|9388|10924|27163|54979|57733|79644|84647 ACOX1|IL1B|PCK1|PLA2G2A|SPP1|LIPG|SMPDL3A|NAAA|PLAAT2|GBA3|SRD5A3|PLA2G12B -1.021785322 0 152 1 1 -2.408851764 3.85622347

1 -2.317958801 GO:0004620 M1 1 0 GO Molecular Functions 21 phospholipase activity -2.317958801 3.85622347 3.595366011 28162 109 402 6 1.492537313 0.604761504 2151|5320|9388|10924|54979|84647 F2RL2|PLA2G2A|LIPG|SMPDL3A|PLAAT2|PLA2G12B -0.948583111 0 152 0 0

-2.408851764 3.85622347
1 -2.106698085 GO:0006644 M1 1 0 GO Biological Processes 19 phospholipid metabolic process -2.106698085 2.168360578 2.903086581 28162 420 402 13 3.233830846 0.882281956 3158|5320|9388|10924|27163|54979|55151|56895|79644|79888|80157|80235|84647 HMGCS2|PLA2G2A|LIPG|SMPDL3A|NAAA|PLAAT2|TMEM38B|AGPAT4|SRD5A3|LPCAT1|CWH43|PIGZ|PLA2G12B -0.783467881 0 152 0 0 -2.408851764 3.85622347
1 -2.330058653 GO:0019865 M1 1 0 GO Molecular Functions 21 immunoglobulin binding -2.330058653 8.756840796 4.574790579 28162 24 402 3 0.746268657 0.429247719 2212|2215|7450 FCGR2A|FCGR3B|VWF -0.95976962 0 153 1 1 -2.330058653 8.756840796
1 -2.291825379 GO:0019842 M1 1 0 GO Molecular Functions 21 vitamin binding -2.291825379 3.358788251 3.438663282 28162 146 402 7 1.741293532 0.652391792 18|6947|8942|23596|27010|29968|55214 ABAT|TCN1|KYNU|OPN3|TPK1|PSAT1|P3H2 -0.929240126 0 154 1 1 -2.291825379 3.358788251
1 -2.279112201 GO:0000030 M1 1 0 GO Molecular Functions 21 mannosyltransferase activity -2.279112201 8.406567164 4.458362801 28162 25 402 3 0.746268657 0.429247719 23333|23753|80235 DPY19L1|SDF2L1|PIGZ -0.923653081 0 155 1 1 -2.279112201 8.406567164
1 -2.279112201 GO:0035902 M1 1 0 GO Biological Processes 19 response to immobilization stress -2.279112201 8.406567164 4.458362801 28162 25 402 3 0.746268657 0.429247719 5468|5967|7031 PPARG|REG1A|TFF1 -0.923653081 0 156 1 1 -2.279112201 8.406567164
1 -2.183390299 GO:0031099 M1 1 0 GO Biological Processes 19 regeneration -2.183390299 2.918946932 3.210662664 28162 192 402 8 1.990049751 0.696552772 301|3371|4837|5396|5468|5967|6696|9076 ANXA1|TNC|NNMT|PRRX1|PPARG|REG1A|SPP1|CLDN1 -0.847370775 0 156 0 0 -2.279112201 8.406567164
1 -2.277941505 GO:0101002 M1 1 0 GO Cellular Components20 ficolin-1-rich granule -2.277941505 3.029393573 3.332567121 28162 185 402 8 1.990049751 0.696552772 240|1604|2357|4318|4332|7130|22918|83716 ALOX5|CD55|FPR1|MMP9|MNDA|TNFAIP6|CD93|CRISPLD2 -0.922923965 0 157 1 1 -2.277941505 3.029393573
1 -2.101103479 GO:0042177 M1 1 0 GO Biological Processes 19 negative regulation of protein catabolic process -2.101103479 3.473788084 3.281600897 28162 121 402 6 1.492537313 0.604761504 1942|4843|5270|7076|27074|282679 EFNA1|NOS2|SERPINE2|TIMP1|LAMP3|AQP11 -0.779624105 0 158 1 1 -2.101103479 3.473788084
1 -2.051262324 GO:0016810 M1 1 0 GO Molecular Functions 21 hydrolase activity, acting on carbon-nitrogen (but not peptide) bonds -2.051262324 3.389744824 3.209343395 28162 124 402 6 1.492537313 0.604761504 8876|9582|11240|27163|80150|166968 VNN1|APOBEC3B|PADI2|NAAA|ASRGL1|MIER3 -0.743060239 0 159 1 1

-2.051262324 3.389744824
1 -2.035002585 GO:0032355 M1 1 0 GO Biological Processes 19 response to estradiol -2.035002585 3.362626866 3.185750102 28162 125 402 6 1.492537313 0.604761504 301|952|1277|2697|3248|10891
ANXA1|CD38|COL1A1|GJA1|HPGD|PPARGC1A -0.72963256 0 160 1 1
-2.035002585 3.362626866
1 -2.005510056 GO:0035579 M1 1 0 GO Cellular Components20 specific granule membrane -2.005510056 3.849160789 3.2759408 28162 91 402 5
1.243781095 0.552765814 2040|5328|10970|22918|57126
STOM|PLAU|CKAP4|CD93|CD177-0.705161612 0 161 1 1 -2.005510056
3.849160789
1 -14.85800725 hsa04974M1 1 0 KEGG Pathway24 Protein digestion and absorption -14.85800725 13.41473484 14.50851533 28162 94 402 18 4.47761194
1.031484687
1277|1278|1281|1282|1290|1292|1293|1303|1306|4224|4225|4321|6505|6519|6550|10008|206358|340024
COL1A1|COL1A2|COL3A1|COL4A1|COL5A2|COL6A2|COL6A3|COL12A1|COL15A1|MEP1A|MEP1B|MMP12|SLC1A1|SLC3A1|SLC9A3|KCNE3|SLC36A1|SLC6A19 -11.95873406 0 1
1 1 -14.85800725 13.41473484
1 -13.96089793 ko04974 M1 1 0 KEGG Pathway24 Protein digestion and absorption -13.96089793 13.23255943 13.98715035 28162 90 402 17 4.228855721
1.003727321
1277|1278|1281|1282|1290|1292|1293|1303|1306|4224|4225|6505|6519|6550|10008|206358|340024
COL1A1|COL1A2|COL3A1|COL4A1|COL5A2|COL6A2|COL6A3|COL12A1|COL15A1|MEP1A|MEP1B|SLC1A1|SLC3A1|SLC9A3|KCNE3|SLC36A1|SLC6A19 -11.36265473 0 1 0 0
-14.85800725 13.41473484
1 -10.20673557 ko04657 M1 1 0 KEGG Pathway24 IL-17 signaling pathway
-10.20673557 10.54587279 11.09611575 28162 93 402 14 3.482587065
0.914409651
2919|2921|3553|3627|3934|4312|4314|4318|6279|6280|6356|6372|6374|727897
CXCL1|CXCL3|IL1B|CXCL10|LCN2|MMP1|MMP3|MMP9|S100A8|S100A9|CCL11|CXCL6|CXCL5|MUC5B -7.78458364 0 2 1 1 -10.20673557 10.54587279
1 -10.07868049 hsa04657M1 1 0 KEGG Pathway24 IL-17 signaling pathway
-10.07868049 10.32385441 10.9543502 28162 95 402 14 3.482587065
0.914409651
2919|2921|3553|3627|3934|4312|4314|4318|6279|6280|6356|6372|6374|727897
CXCL1|CXCL3|IL1B|CXCL10|LCN2|MMP1|MMP3|MMP9|S100A8|S100A9|CCL11|CXCL6|CXCL5|MUC5B -7.78146729 0 2 0 0 -10.20673557 10.54587279
1 -5.623230368 hsa04062M1 1 0 KEGG Pathway24 Chemokine signaling pathway -5.623230368 5.003909026 6.521137719 28162 182 402 13 3.233830846
0.882281956
2869|2919|2921|3579|3627|4067|4283|6356|6362|6372|6373|6374|10451
GRK5|CXCL1|CXCL3|CXCR2|CXCL10|LYN|CXCL9|CCL11|CCL18|CXCL6|CXCL11|CXCL5|VAV3

-3.958957846 0 2 0 0 -10.20673557 10.54587279
1 -5.623230368 ko04062 M1 1 0 KEGG Pathway 24 Chemokine signaling pathway -5.623230368 5.003909026 6.521137719 28162 182 402 13 3.233830846 0.882281956 2869|2919|2921|3579|3627|4067|4283|6356|6362|6372|6373|6374|10451 GRK5|CXCL1|CXCL3|CXCR2|CXCL10|LYN|CXCL9|CCL11|CCL18|CXCL6|CXCL11|CXCL5|VAV3 -3.958957846 0 2 0 0 -10.20673557 10.54587279
1 -5.454762305 ko04668 M1 1 0 KEGG Pathway 24 TNF signaling pathway -5.454762305 6.486548738 6.874499027 28162 108 402 10 2.487562189 0.776790586 1906|2919|2921|3553|3627|4314|4318|6374|64764|197259 EDN1|CXCL1|CXCL3|IL1B|CXCL10|MMP3|MMP9|CXCL5|CREB3L2|MLKL -3.834242719 0 2 0 0 -10.20673557 10.54587279
1 -5.208729545 hsa04668 M1 1 0 KEGG Pathway 24 TNF signaling pathway -5.208729545 6.091715336 6.584111249 28162 115 402 10 2.487562189 0.776790586 1906|2919|2921|3553|3627|4314|4318|6374|64764|197259 EDN1|CXCL1|CXCL3|IL1B|CXCL10|MMP3|MMP9|CXCL5|CREB3L2|MLKL -3.640181523 0 2 0 0 -10.20673557 10.54587279
1 -5.044962962 hsa04060 M1 1 0 KEGG Pathway 24 Cytokine-cytokine receptor interaction -5.044962962 3.891929243 5.745886573 28162 270 402 15 3.731343284 0.945283402 608|1439|2690|2919|2921|3553|3575|3579|3627|4283|6356|6362|6372|6373|6374 TNFRSF17|CSF2RB|GHR|CXCL1|CXCL3|IL1B|IL7R|CXCR2|CXCL10|CXCL9|CCL11|CCL18|CXCL6|CXCL11|CXCL5 -3.543629783 0 2 0 0 -10.20673557 10.54587279
1 -5.044962962 ko04060 M1 1 0 KEGG Pathway 24 Cytokine-cytokine receptor interaction -5.044962962 3.891929243 5.745886573 28162 270 402 15 3.731343284 0.945283402 608|1439|2690|2919|2921|3553|3575|3579|3627|4283|6356|6362|6372|6373|6374 TNFRSF17|CSF2RB|GHR|CXCL1|CXCL3|IL1B|IL7R|CXCR2|CXCL10|CXCL9|CCL11|CCL18|CXCL6|CXCL11|CXCL5 -3.543629783 0 2 0 0 -10.20673557 10.54587279
1 -4.347441174 ko05323 M1 1 0 KEGG Pathway 24 Rheumatoid arthritis -4.347441174 6.227086788 5.976839864 28162 90 402 8 1.990049751 0.696552772 1513|2919|3108|3553|4312|4314|6372|6374 CTSK|CXCL1|HLA-DMA|IL1B|MMP1|MMP3|CXCL6|CXCL5 -2.925289241 0 2 0 0 -10.20673557 10.54587279
1 -4.051653492 hsa05323 M1 1 0 KEGG Pathway 24 Rheumatoid arthritis -4.051653492 5.660987989 5.590567107 28162 99 402 8 1.990049751 0.696552772 1513|2919|3108|3553|4312|4314|6372|6374 CTSK|CXCL1|HLA-DMA|IL1B|MMP1|MMP3|CXCL6|CXCL5 -2.708682805 0 2 0 0 -10.20673557 10.54587279
1 -7.780515126 hsa04610 M1 1 0 KEGG Pathway 24 Complement and coagulation cascades -7.780515126 9.75445557 9.3766622 28162 79 402 11 2.736318408 0.813665012 629|716|718|722|725|1604|2151|3426|5328|7056|7450 CFB|C1S|C3|C4BPA|C4BPB|CD55|F2RL2|CFI|PLAU|THBD|VWF -5.667639906 0 3 1 1 -7.780515126 9.75445557

1 -7.780515126 ko04610 M1 1 0 KEGG Pathway 24 Complement and coagulation cascades -7.780515126 9.75445557 9.3766622 28162 79 402 11 2.736318408 0.813665012 629|716|718|722|725|1604|2151|3426|5328|7056|7450 CFB|C1S|C3|C4BPA|C4BPB|CD55|F2RL2|CFI|PLAU|THBD|VWF -5.667639906 0 3 0 0 -7.780515126 9.75445557

1 -7.721815054 hsa05150 M1 1 0 KEGG Pathway 24 Staphylococcus aureus infection -7.721815054 9.632524876 9.304566549 28162 80 402 11 2.736318408 0.813665012 629|716|718|1670|1671|1672|2212|2215|2357|3108|3426 CFB|C1S|C3|DEFA5|DEFA6|DEFB1|FCGR2A|FCGR3B|FPR1|HLA-DMA|CFI -5.667639906 0 4 1 1 -7.721815054 10.00781805

1 -5.901202089 ko05150 M1 1 0 KEGG Pathway 24 Staphylococcus aureus infection -5.901202089 10.00781805 8.11972704 28162 56 402 8 1.990049751 0.696552772 629|716|718|2212|2215|2357|3108|3426 CFB|C1S|C3|FCGR2A|FCGR3B|FPR1|HLA-DMA|CFI -4.115872254 0 4 0 0 -7.721815054 10.00781805

1 -4.080543744 hsa05140 M1 1 0 KEGG Pathway 24 Leishmania infection -4.080543744 6.717576501 5.886167373 28162 73 402 7 1.741293532 0.652391792 718|2212|2215|3108|3553|4688|4843 C3|FCGR2A|FCGR3B|HLA-DMA|IL1B|NCF2|NOS2 -2.712749474 0 4 0 0 -7.721815054 10.00781805

1 -4.080543744 ko05140 M1 1 0 KEGG Pathway 24 Leishmaniasis -4.080543744 6.717576501 5.886167373 28162 73 402 7 1.741293532 0.652391792 718|2212|2215|3108|3553|4688|4843 C3|FCGR2A|FCGR3B|HLA-DMA|IL1B|NCF2|NOS2 -2.712749474 0 4 0 0 -7.721815054 10.00781805

1 -2.764537887 ko04145 M1 1 0 KEGG Pathway 24 Phagosome -2.764537887 3.639206565 3.952028122 28162 154 402 8 1.990049751 0.696552772 718|2212|2215|3108|4688|7057|79861|84617 C3|FCGR2A|FCGR3B|HLA-DMA|NCF2|THBS1|TUBAL3|TUBB6 -1.55546078 0 4 0 0 -7.721815054 10.00781805

1 -2.52973919 hsa04145 M1 1 0 KEGG Pathway 24 Phagosome -2.52973919 3.335939351 3.654357711 28162 168 402 8 1.990049751 0.696552772 718|2212|2215|3108|4688|7057|79861|84617 C3|FCGR2A|FCGR3B|HLA-DMA|NCF2|THBS1|TUBAL3|TUBB6 -1.386340858 0 4 0 0 -7.721815054 10.00781805

1 -7.606953049 hsa04512 M1 1 0 KEGG Pathway 24 ECM-receptor interaction -7.606953049 9.397585245 9.164106496 28162 82 402 11 2.736318408 0.813665012 1277|1278|1282|1292|1293|3371|3915|6696|7057|7450|284217 COL1A1|COL1A2|COL4A1|COL6A2|COL6A3|TNC|LAMC1|SPP1|THBS1|VWF|LAMA1 -5.661922372 0 5 1 1 -7.606953049 9.397585245

1 -7.606953049 ko04512 M1 1 0 KEGG Pathway 24 ECM-receptor interaction -7.606953049 9.397585245 9.164106496 28162 82 402 11 2.736318408 0.813665012 1277|1278|1282|1292|1293|3371|3915|6696|7057|7450|284217 COL1A1|COL1A2|COL4A1|COL6A2|COL6A3|TNC|LAMC1|SPP1|THBS1|VWF|LAMA1

-5.661922372 0 5 0 0 -7.606953049 9.397585245
1 -6.159939434 hsa04151 M1 1 0 KEGG Pathway 24 PI3K-Akt signaling pathway -6.159939434 3.687090861 6.346287296 28162 380 402 20 4.975124378 1.084445172 1277|1278|1282|1292|1293|1942|2263|2690|3371|3575|3915|5105|6696|7057|7076|7450|23035|64764|154043|284217 COL1A1|COL1A2|COL4A1|COL6A2|COL6A3|EFNA1|FGFR2|GHR|TNC|IL7R|LAMC1|PCK1|SPP1|THBS1|TIMP1|VWF|PHLPP2|CREB3L2|CNKSR3|LAMA1 -4.339847493 0 5 0 0 -7.606953049 9.397585245
1 -5.602958528 ko04151 M1 1 0 KEGG Pathway 24 PI3K-Akt signaling pathway -5.602958528 3.687090861 6.016503506 28162 342 402 18 4.47761194 1.031484687 1277|1278|1282|1292|1293|1942|2263|2690|3371|3575|3915|5105|6696|7057|7450|23035|64764|284217 COL1A1|COL1A2|COL4A1|COL6A2|COL6A3|EFNA1|FGFR2|GHR|TNC|IL7R|LAMC1|PCK1|SPP1|THBS1|VWF|PHLPP2|CREB3L2|LAMA1-3.958957846 0 5 0 0 -7.606953049 9.397585245
1 -5.19703203 hsa04510 M1 1 0 KEGG Pathway 24 Focal adhesion -5.19703203 4.576439411 6.09274302 28162 199 402 13 3.233830846 0.882281956 857|1277|1278|1282|1292|1293|3371|3915|6696|7057|7450|10451|284217 CAV1|COL1A1|COL1A2|COL4A1|COL6A2|COL6A3|TNC|LAMC1|SPP1|THBS1|VWF|VAV3|LAMA1 -3.640181523 0 5 0 0 -7.606953049 9.397585245
1 -5.19703203 ko04510 M1 1 0 KEGG Pathway 24 Focal adhesion -5.19703203 4.576439411 6.09274302 28162 199 402 13 3.233830846 0.882281956 857|1277|1278|1282|1292|1293|3371|3915|6696|7057|7450|10451|284217 CAV1|COL1A1|COL1A2|COL4A1|COL6A2|COL6A3|TNC|LAMC1|SPP1|THBS1|VWF|VAV3|LAMA1 -3.640181523 0 5 0 0 -7.606953049 9.397585245
1 -6.885267506 ko05146 M1 1 0 KEGG Pathway 24 Amoebiasis -6.885267506 8.027104063 8.299445416 28162 96 402 11 2.736318408 0.813665012 1277|1278|1281|1282|2769|2919|3553|3915|4843|5272|284217 COL1A1|COL1A2|COL3A1|COL4A1|GNA15|CXCL1|IL1B|LAMC1|NOS2|SERPINB9|LAMA1 -4.985994319 0 6 1 1 -6.885267506 8.027104063
1 -6.40038318 hsa05146 M1 1 0 KEGG Pathway 24 Amoebiasis -6.40038318 7.201887757 7.734602271 28162 107 402 11 2.736318408 0.813665012 1277|1278|1281|1282|2769|2919|3553|3915|4843|5272|284217 COL1A1|COL1A2|COL3A1|COL4A1|GNA15|CXCL1|IL1B|LAMC1|NOS2|SERPINB9|LAMA1 -4.542502678 0 6 0 0 -6.885267506 8.027104063
1 -4.628346081 hsa04933 M1 1 0 KEGG Pathway 24 AGE-RAGE signaling pathway in diabetic complications -4.628346081 5.89245362 6.101558809 28162 107 402 9 2.23880597 0.737867616 183|1277|1278|1281|1282|1906|3553|4313|7056 AGT|COL1A1|COL1A2|COL3A1|COL4A1|EDN1|IL1B|MMP2|THBD -3.160436658 0 6 0 0 -6.885267506 8.027104063

1 -4.051653492 ko04933 M1 1 0 KEGG Pathway 24 AGE-RAGE signaling pathway in diabetic complications -4.051653492 5.660987989 5.590567107 28162 99 402 8 1.990049751 0.696552772 1277|1278|1281|1282|1906|3553|4313|7056 COL1A1|COL1A2|COL3A1|COL4A1|EDN1|IL1B|MMP2|THBD -2.708682805 0 6 0 0 -6.885267506 8.027104063
1 -2.569035305 hsa04611 M1 1 0 KEGG Pathway 24 Platelet activation -2.569035305 3.772177574 3.812352165 28162 130 402 7 1.741293532 0.652391792 1277|1278|1281|2212|4067|5028|7450 COL1A1|COL1A2|COL3A1|FCGR2A|LYN|P2RY1|VWF -1.412801313 0 6 0 0 -6.885267506 8.027104063
1 -5.860474113 ko05133 M1 1 0 KEGG Pathway 24 Pertussis -5.860474113 8.295954438 7.664278819 28162 76 402 9 2.23880597 0.737867616 716|718|722|725|834|3553|4843|6372|6374 C1S|C3|C4BPA|C4BPB|CASP1|IL1B|NOS2|CXCL6|CXCL5 -4.107328961 0 7 1 1 -5.860474113 8.295954438
1 -5.624986197 hsa05133 M1 1 0 KEGG Pathway 24 Pertussis -5.624986197 7.783858485 7.357670526 28162 81 402 9 2.23880597 0.737867616 716|718|722|725|834|3553|4843|6372|6374 C1S|C3|C4BPA|C4BPB|CASP1|IL1B|NOS2|CXCL6|CXCL5 -3.958957846 0 7 0 0 -5.860474113 8.295954438
1 -2.951056812 ko05134 M1 1 0 KEGG Pathway 24 Legionellosis -2.951056812 6.368611488 4.795825872 28162 55 402 5 1.243781095 0.552765814 718|834|2919|2921|3553 C3|CASP1|CXCL1|CXCL3|IL1B -1.714541456 0 7 0 0 -5.860474113 8.295954438
1 -2.813136226 hsa05134 M1 1 0 KEGG Pathway 24 Legionellosis -2.813136226 5.936841218 4.568000633 28162 59 402 5 1.243781095 0.552765814 718|834|2919|2921|3553 C3|CASP1|CXCL1|CXCL3|IL1B -1.595104276 0 7 0 0 -5.860474113 8.295954438
1 -2.106174654 ko05132 M1 1 0 KEGG Pathway 24 Salmonella infection -2.106174654 4.072949207 3.434506341 28162 86 402 5 1.243781095 0.552765814 834|2919|2921|3553|4843 CASP1|CXCL1|CXCL3|IL1B|NOS2 -1.045750558 0 7 0 0 -5.860474113 8.295954438
1 -5.107669933 ko04976 M1 1 0 KEGG Pathway 24 Bile secretion -5.107669933 7.893490295 6.998614544 28162 71 402 8 1.990049751 0.696552772 343|366|5243|6550|8671|9429|123264|200931 AQP8|AQP9|ABCB1|SLC9A3|SLC4A4|ABCG2|SLC51B|SLC51A -3.570124582 0 8 1 1 -5.107669933 12.18343067
1 -4.928985641 hsa04976 M1 1 0 KEGG Pathway 24 Bile secretion -4.928985641 7.472504146 6.754257657 28162 75 402 8 1.990049751 0.696552772 343|366|5243|6550|8671|9429|123264|200931 AQP8|AQP9|ABCB1|SLC9A3|SLC4A4|ABCG2|SLC51B|SLC51A -3.444685802 0 8 0 0 -5.107669933 12.18343067
1 -3.534421373 hsa04964 M1 1 0 KEGG Pathway 24 Proximal tubule bicarbonate reclamation -3.534421373 12.18343067 6.456720249 28162 23 402 4

0.995024876 0.495031064 762|5105|6550|8671 CA4|PCK1|SLC9A3|SLC4A4
-2.258397476 0 8 0 0 -5.107669933 12.18343067
1 -3.534421373 ko04964 M1 1 0 KEGG Pathway 24 Proximal tubule bicarbonate reclamation -3.534421373 12.18343067 6.456720249 28162 23 402 4
0.995024876 0.495031064 762|5105|6550|8671 CA4|PCK1|SLC9A3|SLC4A4
-2.258397476 0 8 0 0 -5.107669933 12.18343067
1 -4.409199603 hsa04670 M1 1 0 KEGG Pathway 24 Leukocyte transendothelial migration -4.409199603 5.530636292 5.832946697 28162 114 402 9
2.23880597 0.737867616 4313|4318|4478|4688|5175|9073|9076|10451|137075
MMP2|MMP9|MSN|NCF2|PECAM1|CLDN8|CLDN1|VAV3|CLDN23 -2.972324413 0 9
1 1 -4.409199603 6.834607451
1 -4.409199603 ko04670 M1 1 0 KEGG Pathway 24 Leukocyte transendothelial migration -4.409199603 5.530636292 5.832946697 28162 114 402 9 2.23880597
0.737867616 4313|4318|4478|4688|5175|9073|9076|10451|137075
MMP2|MMP9|MSN|NCF2|PECAM1|CLDN8|CLDN1|VAV3|CLDN23 -2.972324413 0 9
0 0 -4.409199603 6.834607451
1 -2.989459592 ko05418 M1 1 0 KEGG Pathway 24 Fluid shear stress and atherosclerosis -2.989459592 3.946745148 4.236234723 28162 142 402 8
1.990049751 0.696552772 857|1906|3553|4313|4318|4688|5175|7056
CAV1|EDN1|IL1B|MMP2|MMP9|NCF2|PECAM1|THBD -1.743398918 0 9 0 0
-4.409199603 6.834607451
1 -2.874016637 hsa05418 M1 1 0 KEGG Pathway 24 Fluid shear stress and atherosclerosis -2.874016637 3.786741966 4.090415121 28162 148 402 8
1.990049751 0.696552772 857|1906|3553|4313|4318|4688|5175|7056
CAV1|EDN1|IL1B|MMP2|MMP9|NCF2|PECAM1|THBD -1.646841308 0 9 0 0
-4.409199603 6.834607451
1 -2.563886473 hsa05219 M1 1 0 KEGG Pathway 24 Bladder cancer
-2.563886473 6.834607451 4.498995123 28162 41 402 4 0.995024876
0.495031064 4312|4313|4318|7057 MMP1|MMP2|MMP9|THBS1 -1.412801313 0
9 0 0 -4.409199603 6.834607451
1 -2.563886473 ko05219 M1 1 0 KEGG Pathway 24 Bladder cancer
-2.563886473 6.834607451 4.498995123 28162 41 402 4 0.995024876
0.495031064 4312|4313|4318|7057 MMP1|MMP2|MMP9|THBS1 -1.412801313 0
9 0 0 -4.409199603 6.834607451
1 -2.367431958 hsa05205 M1 1 0 KEGG Pathway 24 Proteoglycans in cancer
-2.367431958 2.892167602 3.374977599 28162 218 402 9 2.23880597
0.737867616 288|857|4313|4318|4478|5328|7057|7474|7837
ANK3|CAV1|MMP2|MMP9|MSN|PLAU|THBS1|WNT5A|PXDN -1.231586765 0 9 0
0 -4.409199603 6.834607451
1 -2.044259464 ko05205 M1 1 0 KEGG Pathway 24 Proteoglycans in cancer
-2.044259464 2.760777394 3.029835066 28162 203 402 8 1.990049751
0.696552772 288|857|4313|4318|4478|5328|7057|7474
ANK3|CAV1|MMP2|MMP9|MSN|PLAU|THBS1|WNT5A -0.990084317 0 9 0 0
-4.409199603 6.834607451

1 -4.119021843 hsa03320 M1 1 0 KEGG Pathway 24 PPAR signaling pathway -4.119021843 6.810876175 5.94099698 28162 72 402 7 1.741293532 0.652391792 51|2182|3158|4199|4312|5105|5468 ACOX1|ACSL4|HMGCS2|ME1|MMP1|PCK1|PPARG -2.724898634 0 10 1 1 -4.119021843 6.810876175

1 -4.119021843 ko03320 M1 1 0 KEGG Pathway 24 PPAR signaling pathway -4.119021843 6.810876175 5.94099698 28162 72 402 7 1.741293532 0.652391792 51|2182|3158|4199|4312|5105|5468 ACOX1|ACSL4|HMGCS2|ME1|MMP1|PCK1|PPARG -2.724898634 0 10 0 0 -4.119021843 6.810876175

1 -2.170191843 hsa04146 M1 1 0 KEGG Pathway 24 Peroxisome -2.170191843 4.220164239 3.535522313 28162 83 402 5 1.243781095 0.552765814 51|2053|2182|4843|55670 ACOX1|EPHX2|ACSL4|NOS2|PEX26 -1.070259205 0 10 0 0 -4.119021843 6.810876175

1 -2.170191843 ko04146 M1 1 0 KEGG Pathway 24 Peroxisome -2.170191843 4.220164239 3.535522313 28162 83 402 5 1.243781095 0.552765814 51|2053|2182|4843|55670 ACOX1|EPHX2|ACSL4|NOS2|PEX26 -1.070259205 0 10 0 0 -4.119021843 6.810876175

1 -3.628646254 hsa04514 M1 1 0 KEGG Pathway 24 Cell adhesion molecules (CAMs) -3.628646254 4.378420398 4.891068026 28162 144 402 9 2.23880597 0.737867616 1001|3108|5175|6402|9073|9076|23705|29126|137075 CDH3|HLA-DMA|PECAM1|SELL|CLDN8|CLDN1|CADM1|CD274|CLDN23 -2.309156664 0 11 1 1 -3.628646254 4.378420398

1 -3.628646254 ko04514 M1 1 0 KEGG Pathway 24 Cell adhesion molecules (CAMs) -3.628646254 4.378420398 4.891068026 28162 144 402 9 2.23880597 0.737867616 1001|3108|5175|6402|9073|9076|23705|29126|137075 CDH3|HLA-DMA|PECAM1|SELL|CLDN8|CLDN1|CADM1|CD274|CLDN23 -2.309156664 0 11 0 0 -3.628646254 4.378420398

1 -3.593035205 hsa00590 M1 1 0 KEGG Pathway 24 Arachidonic acid metabolism -3.593035205 6.779489649 5.482258467 28162 62 402 6 1.492537313 0.604761504 240|1555|2053|5320|84647|493869 ALOX5|CYP2B6|EPHX2|PLA2G2A|PLA2G12B|GPX8 -2.295822009 0 12 1 1 -3.593035205 8.406567164

1 -3.593035205 ko00590 M1 1 0 KEGG Pathway 24 Arachidonic acid metabolism -3.593035205 6.779489649 5.482258467 28162 62 402 6 1.492537313 0.604761504 240|1555|2053|5320|84647|493869 ALOX5|CYP2B6|EPHX2|PLA2G2A|PLA2G12B|GPX8 -2.295822009 0 12 0 0 -3.593035205 8.406567164

1 -2.279112201 hsa00592 M1 1 0 KEGG Pathway 24 alpha-Linolenic acid metabolism -2.279112201 8.406567164 4.458362801 28162 25 402 3 0.746268657 0.429247719 51|5320|84647 ACOX1|PLA2G2A|PLA2G12B -1.157990264 0 12 0 0 -3.593035205 8.406567164

1 -2.279112201 ko00592 M1 1 0 KEGG Pathway 24 alpha-Linolenic acid metabolism -2.279112201 8.406567164 4.458362801 28162 25 402 3 0.746268657

0.429247719 51|5320|84647 ACOX1|PLA2G2A|PLA2G12B -1.157990264 0 12 0
0 -3.593035205 8.406567164
1 -3.129117828 hsa04620 M1 1 0 KEGG Pathway 24 Toll-like receptor signaling pathway -3.129117828 4.715221967 4.567729867 28162 104 402 7 1.741293532
0.652391792 1513|3553|3627|4283|6373|6696|7098
CTSK|IL1B|CXCL10|CXCL9|CXCL11|SPP1|TLR3 -1.873297317 0 13 1 1
-3.129117828 4.715221967
1 -3.129117828 ko04620 M1 1 0 KEGG Pathway 24 Toll-like receptor signaling pathway -3.129117828 4.715221967 4.567729867 28162 104 402 7 1.741293532
0.652391792 1513|3553|3627|4283|6373|6696|7098
CTSK|IL1B|CXCL10|CXCL9|CXCL11|SPP1|TLR3 -1.873297317 0 13 0 0
-3.129117828 4.715221967
1 -2.625899697 hsa00830 M1 1 0 KEGG Pathway 24 Retinol metabolism
-2.625899697 5.388825105 4.262872164 28162 65 402 5 1.243781095
0.552765814 126|1555|29785|79799|195814
ADH1C|CYP2B6|CYP2S1|UGT2A3|SDR16C5 -1.434196686 0 14 1 1
-2.625899697 5.388825105
1 -2.625899697 ko00830 M1 1 0 KEGG Pathway 24 Retinol metabolism
-2.625899697 5.388825105 4.262872164 28162 65 402 5 1.243781095
0.552765814 126|1555|29785|79799|195814
ADH1C|CYP2B6|CYP2S1|UGT2A3|SDR16C5 -1.434196686 0 14 0 0
-2.625899697 5.388825105
1 -2.567910806 hsa04640 M1 1 0 KEGG Pathway 24 Hematopoietic cell lineage
-2.567910806 4.333282043 3.957334293 28162 97 402 6 1.492537313
0.604761504 290|952|1604|3108|3553|3575 ANPEP|CD38|CD55|HLA-DMA|IL1B|IL7R
-1.412801313 0 15 1 1 -2.567910806 4.333282043
1 -2.567910806 ko04640 M1 1 0 KEGG Pathway 24 Hematopoietic cell lineage
-2.567910806 4.333282043 3.957334293 28162 97 402 6 1.492537313
0.604761504 290|952|1604|3108|3553|3575 ANPEP|CD38|CD55|HLA-DMA|IL1B|IL7R
-1.412801313 0 15 0 0 -2.567910806 4.333282043
1 -2.220014968 ko04978 M1 1 0 KEGG Pathway 24 Mineral absorption
-2.220014968 5.494488343 3.865934849 28162 51 402 4 0.995024876
0.495031064 4499|6550|140803|340024 MT1M|SLC9A3|TRPM6|SLC6A19
-1.106071616 0 16 1 1 -2.220014968 5.494488343
1 -2.160825932 hsa04978 M1 1 0 KEGG Pathway 24 Mineral absorption
-2.160825932 5.28714916 3.759336167 28162 53 402 4 0.995024876
0.495031064 4499|6550|140803|340024 MT1M|SLC9A3|TRPM6|SLC6A19
-1.068804827 0 16 0 0 -2.220014968 5.494488343
1 -2.148534079 hsa05222 M1 1 0 KEGG Pathway 24 Small cell lung cancer
-2.148534079 4.169924189 3.501327751 28162 84 402 5 1.243781095
0.552765814 1030|1282|3915|4843|284217 CDKN2B|COL4A1|LAMC1|NOS2|LAMA1
-1.068804827 0 17 1 1 -2.148534079 4.169924189
1 -2.148534079 ko05222 M1 1 0 KEGG Pathway 24 Small cell lung cancer
-2.148534079 4.169924189 3.501327751 28162 84 402 5 1.243781095

0.552765814	1030|1282|3915|4843|284217	CDKN2B|COL4A1|LAMC1|NOS2|LAMA1
-1.068804827 0	17	0	0	-2.148534079 4.169924189
1	-2.132224823 hsa00480 M1	1	0	KEGG Pathway 24	Glutathione metabolism
-2.132224823 5.18923899	3.70803971	28162	54	402 4	0.995024876
0.495031064	290|6241|6723|493869 ANPEP|RRM2|SRM|GPX8	-1.065460549 0	18
1	1	-2.132224823 5.18923899
1	-2.132224823 ko00480	M1	1	0	KEGG Pathway 24	Glutathione metabolism
-2.132224823 5.18923899	3.70803971	28162	54	402 4	0.995024876
0.495031064	290|6241|6723|493869 ANPEP|RRM2|SRM|GPX8	-1.065460549 0	18
0	0	-2.132224823 5.18923899
1	-2.023879618 hsa00140 M1	1	0	KEGG Pathway 24	Steroid	hormone	biosynthesis
-2.023879618 4.831360439	3.514878126	28162	58	402 4	0.995024876
0.495031064	3284|3294|79644|79799	HSD3B2|HSD17B2|SRD5A3|UGT2A3
-0.981938927 0	19	1	1	-2.023879618 4.831360439
1	-2.023879618 ko00140	M1	1	0	KEGG Pathway 24	Steroid	hormone	biosynthesis
-2.023879618 4.831360439	3.514878126	28162	58	402 4	0.995024876
0.495031064	3284|3294|79644|79799	HSD3B2|HSD17B2|SRD5A3|UGT2A3
-0.981938927 0	19	0	0	-2.023879618 4.831360439
