# Supplementary material for: Identification of diagnostic biomarks and immune cell infiltration in ulcerative colitis
Source: Sci Rep. 2023 Apr 13;13:6081. doi: 10.1038/s41598-023-33388-5 (PMC10102327; doi:10.1038/s41598-023-33388-5)
Supplement: Supplementary file 4 — Supplementary Information 4. [file 41598_2023_33388_MOESM4_ESM.pdf]

Table 4. Top enrichment function of GSEA

Category	Enrich Function	NES	EnrichmentScore	Pvalue
GOBiological Processes	Divalent inorganic cation homeostasis	1.5939	0.418494069	0.001126
GOBiological Processes	Regulation of body fluid levels	1.681891	0.441597181	0.001126
GOBiological Processes	Positive regulation of mapk cascade	1.430607	0.375318791	0.001131
GOBiological Processes	Response to peptide	1.398077	0.366748989	0.001136
GOBiological Processes	Positive regulation of response to external stimulus	1.909546	0.502149628	0.001139
GOBiological Processes	Skeletal system development	1.513776	0.398255242	0.001139
GOBiological Processes	Positive regulation of cytokine production	1.98462	0.525755728	0.00114
GOBiological Processes	Regulation of protein serine threonine kinase activity	1.470796	0.387434746	0.001144
GOBiological Processes	Regulation of cell cycle phase transition	1.462534	0.387277316	0.001145

GOBiological Processes	Leukocyte migration	2.109316	0.559059823	0.001147
KEGG Pathway	Cytokine cytokine receptor interaction	2.126128	0.581330124	0.00122
KEGG Pathway	Focal adhesion	1.785912	0.5016776	0.00128
KEGG Pathway	Chemokine signaling pathway	2.039076	0.579948363	0.001285
KEGG Pathway	Cell adhesion molecules cams	1.831412	0.540533826	0.001346
KEGG Pathway	Toll like receptor signaling pathway	1.836812	0.563113257	0.001422
KEGG Pathway	Systemic lupus erythematosus	1.77301	0.546894285	0.001429
KEGG Pathway	Ecm receptor interaction	2.080976	0.652955629	0.001441
KEGG Pathway	Leishmania infection	2.084882	0.672141757	0.00146
KEGG Pathway	Viral myocarditis	1.962094	0.635095045	0.001462
KEGG Pathway	Antigen processing and presentation	1.797272	0.57785831	0.001479
