# Supplementary material for: Identification of diagnostic biomarks and immune cell infiltration in ulcerative colitis
Source: Sci Rep. 2023 Apr 13;13:6081. doi: 10.1038/s41598-023-33388-5 (PMC10102327; doi:10.1038/s41598-023-33388-5)
Supplement: Supplementary file 5 — Supplementary Information 5. [file 41598_2023_33388_MOESM5_ESM.pdf]

ID Description setSize enrichmentScore NES pvalue p.adjust qvalues rank leading_edge core_enrichment
GOBP_DIVALENT_INORGANIC_CATION_HOMEOSTASIS GOBP_DIVALENT_INORGANIC_CATION_HOMEOSTASIS GOBP_DIVALENT_INORGANIC_CATION_HOMEOSTASIS 476 0.418494069 1.593899616 0.001126126 0.030581767 0.024202852 2142 tags=22%, list=13%, signal=20% REG1A/S100A8/CD55/CCL11/GJA1/CEMIP/CXCL9/S100A9/CXCL11/CXCL13/CXCL10/PROK2/WNT5A/TGM2/CD38/CXCR2/CAV1/MS4A1/GNA15/F2RL2/FPR1/LYN/CDH5/CCR2/EDNRA/CCR10/TRPA1/STC1/S1PR3/MCUB/JPH1/APLNR/PTPRC/F2R/BNIP3/TCIRG1/CCR1/RAMP3/BOK/PLN/ACKR4/C5AR1/PDGFRA/CCR7/CD19/FYN/ANXA6/JAK2/TMEM165/THY1/CXCR4/CAV2/P2RX5/SLC41A1/P2RY8/PLCG2/SLC39A6/DIAPH1/CD40/SNX10/FPR3/CORO1A/S1PR1/CALR/CLIC2/ERO1A/ATP2A3/CCR6/NMU/SELENON/PIK3CG/FFAR4/C3AR1/LCK/TMC8/SLC24A3/CCL19/PRKCB/GSTO1/CYP27B1/ATP13A2/PKD2/HSP90B1/PRNP/TRPV2/WFS1/GRINA/IBTK/ITGAV/PTGFR/GPR65/PML/RAP1GDS1/SLC30A7/ATP2B4/FPR2/AP3D1/SLC39A10/ATP2A2/CYSLTR1/CCR5/SELENOK/GPR4/TMEM64/TNFSF11/P2RX7/TRPC6
GOBP_REGULATION_OF_BODY_FLUID_LEVELS GOBP_REGULATION_OF_BODY_FLUID_LEVELS GOBP_REGULATION_OF_BODY_FLUID_LEVELS 476 0.441597181 1.681891404 0.001126126 0.030581767 0.024202852 3086 tags=30%, list=18%, signal=25% C4BPB/TFPI2/GJA1/AQP3/PLAU/VWF/CLDN1/COL1A2/COL1A1/SLC7A11/CAV1/THBD/GNA15/F2RL2/ANXA5/COL3A1/SERPINE2/LYN/ITGA2/ADA/SERPING1/HEG1/F3/SERPINE1/SELP/SERPINA1/LCP2/HBB/PF4/TFPI/STXBP1/NME1/APLNR/PDPN/OAS2/F2R/HIF1A/FCER1G/PRKAR2B/XBP1/FN1/ST3GAL4/GNA14/KCNN4/PDGFRA/FYN/ENTPD1/JAK2/PLEK/SHH/PPAT/P2RX5/PLAT/GNAI2/MYL9/FAP/SCNN1G/HYAL2/MET/PLCG2/CD40/VIP/ABCA12/FLNA/PROCR/FLI1/LMAN1/ZFPM2/DGKZ/XDH/A2M/GPAT4/VAV1/IRF1/PAM/CBX5/ANO6/PIK3CG/AKR1B1/LCK/DOCK11/PRKCB/CHRM3/AGR2/CCND1/SH2B2/HK2/UMPS/HPSE/PRKCH/PIK3R5/WFS1/EXT2/VCL/EHD2/IL6/AXL/PEAR1/ADCY4/PHF21A/TLN1/CREB1/MMRN1/PABPC4/CAD/P2RX7/TRPC6/VKORC1/COPA/CD59/SERPINF2/DOCK9/GNA12/F12/BLK/AQP1/RAD51C/FOXA2/SYK/MICAL1/TYRO3/DOCK8/WAS/ATP7A/PRKAR1A/C1QTNF1/ADCY7/ARRB2/FBLN1/APOE/DGKA/PLAUR/EDNRB/CD34/STAT5B/GPI/PTPN11/PROS1/EMP2/STAT5A/GJA5/FA2H/PRCP
GOBP_POSITIVE_REGULATION_OF_MAPK_CASCADE GOBP_POSITIVE_REGULATION_OF_MAPK_CASCADE GOBP_POSITIVE_REGULATION_OF_MAPK_CASCADE 483 0.375318791 1.430606808 0.001131222 0.030581767 0.024202852 2551 tags=25%, list=15%, signal=22% CHI3L1/CCL11/PLA2G2A/CCL18/PROK2/IL1B/S100A12/AGT/WNT5A/MLKL/ROBO1/FERMT2/FPR1/PEA15/CD44/UNC5CL/CD74/TGFB1/MYDGF/PTPRC/CCL20/F2R/S100A7/RIPK2/CCR1/RAMP3/NQO2/CLEC7A/C5AR1/KIT/PDGFRA/CCR7/FCGR2B/CCL4/CXCR4/CAV2/MAP3K5/DUSP6/GNAI2/DUSP7/ICAM1/CD27/CCL2/PDGFRB/CCL24/IRAK1/CD81/CD40/IGFBP6/RASSF2/MAP3K20/MAP3K6/SH3RF3/KDR/LAPTM5/CCL22/MST1R/TEK/TPBG/XDH/MAP4K1/BANK1/PIK3CG/FFAR4/EZH2/CDK1/CSF1R/PLCB1/GPR183/FGFR1/DKK1/TRAF5/CCL19/SPHK1/IGFBP4/FCRL3/IRAK2/NRP1/PBK/AKAP12/MIF/PIK3R5/PRMT1/CFLAR/DUSP22/SDCBP/LIF/TPD52L1/DENND2B/AJUBA/IL6/ADAM8/ADORA2B/STK3/MAP2K1/ERN1/GPNMB/FPR2/FGD2/HAVCR2/TIMP2/FZD4/TNFSF11/KSR1/NOD2/TRAF1/SHC2/PDGFC/MAPKAPK2/SERPINF2/NPNT/HACD3/LRRK2/PRXL2C/PTPN1/CD4/RASGRP1/TGFB3/CCL7/SYK/FBXW7/TRAF3
GOBP_RESPONSE_TO_PEPTIDE GOBP_RESPONSE_TO_PEPTIDE

GOBP_RESPONSE_TO_PEPTIDE 493 0.366748989 1.398076895 0.001136364 0.030581767 0.024202852 1198 tags=14%, list=7%, signal=14% REG1A/REG1B/MMP3/REG3A/MMP12/TIMP1/IGFBP5/GJA1/ANXA1/IL1B/RHOQ/MZB1/SPARC/COL1A1/MMP9/RAB31/TFF1/CAV1/MMP2/LYN/PKM/ASS1/FBN1/PIK3R3/LPIN1/JAK3/LPL/GRB10/RAB12/GAL/TCIRG1/RIPK2/RAMP3/PRKAR2B/SOCS3/XBP1/VCAM1/PLN/UCP2/VIM/APOBEC1/FYN/FCGR2B/JAK2/PPAT/STAT1/CAV2/TRIM16/TLR2/GNAI2/ICAM1/ABCC1/HHEX/BAIAP2L1/RAB8B/OSBPL8/CD40/SERPINF1/PCSK9/RAB13/CASP4/STAT4/KLF2/IRS1/AGTRAP/TEK/STAT3/CDC6/INSIG1/USO1/AKR1B1

GOBP_POSITIVE_REGULATION_OF_RESPONSE_TO_EXTERNAL_STIMULUS GOBP_POSITIVE_REGULATION_OF_RESPONSE_TO_EXTERNAL_STIMULUS GOBP_POSITIVE_REGULATION_OF_RESPONSE_TO_EXTERNAL_STIMULUS 469 0.502149628 1.909546088 0.001138952 0.030581767 0.024202852 3916 tags=45%, list=23%, signal=35% S100A8/MMP12/C2CD4A/PLA2G2A/S100A9/CXCL13/CXCL8/IDO1/CXCL10/IL1B/S100A12/AGT/WNT5A/TGM2/MNDA/CADM1/THBD/IL33/C3/MUC5B/LYN/ITGA2/F3/SERPINE1/CCR2/CD74/IFI16/TGFB1/AIM2/LY96/PLA2G7/RAC2/LPL/NFKBIZ/S100A7/RIPK2/PTGS2/CCR1/STING1/FCER1G/MUC4/PSME4/PSMB9/TLR8/PARP9/CLEC4A/C5AR1/FCN1/CCR7/SMOC2/FYN/JAK2/CCL4/CEBPB/CXCR4/TLR2/HCK/PDGFRB/ABCC1/NLRC5/HYAL2/MET/FFAR2/CCL24/PLCG2/TYROBP/CD81/ICAM2/GBP5/FLNA/VEGFC/KDR/AKIRIN2/S1PR1/TRIM15/PSMD14/GPRC5B/CLEC4E/TPBG/CALR/VAV1/IL6ST/CCR6/CTSC/ANO6/PIK3CG/C3AR1/PSMD12/RIPOR2/CSF1R/PSMC2/FGFR1/MUC1/GPSM3/CCL19/PSMB2/PSMA3/PSMD1/NRP1/BTK/LAG3/CXCL14/CD47/IL23A/PSMA5/MUC5AC/IL6/TSLP/ADAM8/CREB3/PSMD6/CD180/MUC2/IFNG/AIF1/PSMB5/PSMC4/FPR2/HAVCR2/ZBP1/SLAMF6/GPR4/RELA/NCKAP1L/TNFSF11/NOD2/IRF7/SERPINF2/PSMB1/PSMA1/SWAP70/LY86/MALT1/IL16/LRRK2/CMKLR1/ARTN/NMI/RASGRP1/MDK/CCL7/PSME3/SYK/ETS1/BMP6/PARK7/POLR3G/CGAS/IL18/CUL1/PSMA7/PSMB8/PSMC5/PVR/PLA2G3/IRF3/TRIM5/PPM1F/FGF2/TBK1/AP1G1/PSMB10/HSP90AA1/STAT5B/PSMB6/HRAS/TLR10/IL18RAP/PLSCR1/PSMA4/SFPQ/CDH13/OSMR/NLRC4/PSMD13/IFI35/SLC15A4/PRKD1/PSME1/IL1RL1/COCH/USF1/HMGB1/CARD11/CX3CL1/XRCC5/CD209/PSMC1/DHX58/PRKD2/NECTIN2/CYBA/SUCNR1/DEFB124/PSMB4/HLA-E/ADAM17/MUC17/PSMB3/FADD/PSMD2/IKBKG/CCL26/CCN4/STAP1/PSMC6/LBP/PSMD7/MUC12/CLEC4D

GOBP_SKELETAL_SYSTEM_DEVELOPMENT GOBP_SKELETAL_SYSTEM_DEVELOPMENT GOBP_SKELETAL_SYSTEM_DEVELOPMENT 465 0.398255242 1.513776071 0.001138952 0.030581767 0.024202852 1618 tags=18%, list=10%, signal=16% CHI3L1/TIMP1/GREM1/GJA1/MGP/LOXL2/TGFBI/WNT5A/PRRX1/COL1A2/COL1A1/CREB3L2/MMP9/CSGALNACT1/SPNS2/COL5A2/CDH11/MMP2/COL3A1/HYAL1/ENG/CCN1/FBN1/CD44/TGFB1/BGN/SULF1/STC1/CHST11/CHSY1/TNFRSF11B/LUM/ECM1/PTPRC/VCAN/P3H1/HIF1A/RUNX2/TWIST1/KIT/TGFBR2/PLS3/PDGFRA/ANXA6/SHH/SH3PXD2B/CHRDL2/FGR/LOX/SULF2/HYAL2/SEMA4D/TYROBP/SNX10/RASSF2/SNAI2/FLI1/RAI1/LTF/TEK/PAPSS1/SERPINH1/RAB23/EFEMP1/MEIS1/SP5/INSIG1/RFLNB/CLDN18/FGFR1/RUNX3/TYMS/PIP4K2A/SLC9B2/HAPLN3/ANXA2/EXT2/PPIB/DCHS1/HOXB2/TRPS1/FST

GOBP_POSITIVE_REGULATION_OF_CYTOKINE_PRODUCTION GOBP_POSITIVE_REGULATION_OF_CYTOKINE_PRODUCTION GOBP_POSITIVE_REGULATION_OF_CYTOKINE_PRODUCTION 405 0.525755728 1.984619601 0.001140251 0.030581767 0.024202852 3034 tags=38%, list=18%, signal=32%

MMP12/POU2AF1/ANXA1/CD274/IDO1/IL1B/AGT/WNT5A/MNDA/RGCC/CADM1/SLC7A5/CASP1/IL33/C3/HEG1/F3/SERPINE1/CCR2/SERPINB7/CD74/IFI16/TGFB1/PDE4B/PF4/AIM2/LY96/SULF1/IRF4/LPL/IL27RA/LUM/PTPRC/CLU/F2R/RIPK2/HIF1A/PTGS2/STING1/FCER1G/XBP1/CLEC7A/IL1R1/TLR8/TWIST1/C5AR1/FCN1/CCR7/JAK2/DDX21/CEBPB/LILRB2/CD86/STAT1/TRIM16/POSTN/SASH3/FGR/TLR2/PTAFR/SULF2/HK1/HYAL2/FFAR2/PLCG2/TYROBP/IRAK1/CD81/CD40/HLA-DPA1/GBP5/IL1A/LAPTM5/AKIRIN2/TRIM15/GPRC5B/STAT3/IRF1/IL6ST/CD276/C3AR1/CSF1R/IL12RB1/PLCB1/GPSM3/HILPDA/CCL19/SPHK1/AFAP1L2/IL18R1/EBI3/CD2/MIF/TLR1/RORA/HPSE/CD200/IL23A/CASP8/IL6/TSLP/ADAM8/ARHGEF2/LILRB1/NLRP2/IFNG/AIF1/CREB1/HAVCR2/ZBP1/SLAMF6/SELENOK/RELA/LTB/P2RX7/NOD2/FURIN/NFKB2/PANX1/IRF7/MAPKAPK2/SERPINF2/TMF1/MALT1/IL16/CD4/LY9/RASGRP1/KPNA6/FERMT1/GLMN/POLR3H/MDK/SYK/CD6/ATF4/PARK7/POLR3G/CGAS/IL18/GSDMD/IL17B/PLA2G3/IRF3/NR4A3/TBK1/POLR1C/CD34/HSP90AA1/STAT5B/HLA-DPB1/PTPN11/HRAS/POLR2L

GOBP_REGULATION_OF_PROTEIN_SERINE_THREONINE_KINASE_ACTIVITY

GOBP_REGULATION_OF_PROTEIN_SERINE_THREONINE_KINASE_ACTIVITY

GOBP_REGULATION_OF_PROTEIN_SERINE_THREONINE_KINASE_ACTIVITY 459 0.387434746 1.470795891 0.001144165 0.030581767 0.024202852 3628 tags=35%, list=21%, signal=28%

CEMIP/CXCL10/PROK2/IL1B/S100A12/AGT/WNT5A/TRIB2/RGCC/CAV1/SERPINB3/MLKL/ROBO1/FERMT2/FPR1/LYN/PEA15/HEG1/LAX1/CD74/TGFB1/TCIM/PTPRC/RIPK2/PRKAR2B/CCNB1/DUSP4/DUSP14/C5AR1/KIT/THY1/CXCR4/MAP3K5/DUSP6/DUSP7/PDGFRB/LATS2/CD300A/HHEX/HYAL2/IRAK1/CD81/MYOCD/CD40/IGFBP6/CDC25A/RGS2/MAP3K20/MAP3K6/BLM/LTF/MST1R/DNAJA1/MAP4K1/CDC6/PIK3CG/EZH2/SPRED1/CDK1/CSF1R/FGFR1/ALS2/DKK1/RGS4/NUP62/CCL19/DUSP10/CCNO/CCND1/IRAK2/PLK1/PBK/PKD2/CCNI/PIK3R5/CDKN3/CKS2/CCNL2/TPD52L1/CDKN2C/AJUBA/GTPBP4/ADAM8/ADORA2B/STK3/SFRP1/PKIA/MAP2K1/ATP2B4/IFNG/ERN1/CDKN1C/CHORDC1/CCNQ/FGD2/BCCIP/FZD4/DBF4/CCNH/TNFSF11/KSR1/NOD2/TCL1A/SFRP2/PSRC1/SHC2/PDGFC/MAPKAPK2/CDC37/HACD3/CDK4/LRRK2/PTPN1/RASGRP1/CCNF/HSP90AB1/TGFB3/SYK/UCHL1/CDK5RAP3/AKT1/RGS3/MAP3K11/LIME1/UBE2N/PRKAR1A/APOE/FGF2/CCNA2/ADRB2/IRAK3/PTPN11/SPRY1/HRAS/GSTP1/NR2F2/RASIP1/PIH1D1/STK4/SHC1/CDK5RAP1/CCNC/YWHAG/AIDA/PKN1/CCNB2/EPHA4/IPO7/DRD4/S1PR2/CALM3/MAPKAPK3/CCND3/PKIG/DVL3/ACSL1/DUSP3/GADD45G/RALB

GOBP_REGULATION_OF_CELL_CYCLE_PHASE_TRANSITION

GOBP_REGULATION_OF_CELL_CYCLE_PHASE_TRANSITION

GOBP_REGULATION_OF_CELL_CYCLE_PHASE_TRANSITION 429 0.387277316 1.462533729 0.001145475 0.030581767 0.024202852 4461 tags=44%, list=26%, signal=33%

ANXA1/ADAMTS1/RGCC/CDC25B/HYAL1/FAM83D/TCIM/TRIP13/PRKAR2B/PSME4/PSMB9/CCNB1/NABP1/MAD2L2/SLFN11/ANLN/TUBG1/CCL2/BUB1/CDC20/DACT1/DONSON/NDC80/CDC25A/BLM/CEP78/NEK6/PSMD14/CDCA5/CEP164/CSNK1E/KIF14/CDC6/EZH2/PSMD12/CDK1/PSMC2/PLCB1/MUC1/TUBA4A/PSMB2/CENPE/AURKB/PSMA3/AURKA/ODF2/PSMD1/BUB1B/TUBB/CCND1/PLK1/PKD2/BID/UBE2C/LSM10/DLGAP5/PRMT1/CDC27/PSMA5/CDKN2C/TPX2/AVEN/PML/FHL1/TNKS1BP1/RCC2/CDC7/CDK2/ZWINT/PSMD6/PKIA/CNOT9/ATP2B4/AIF1/GPNMB/PSMB5/PSMC4/CETN2/RDX/EIF4G1/DBF4/PLK2/JADE1/NBN/CEP135/ATF5/ARID3A/HECW2/PSMB1/PSMA1/CDK4/CDC26/PAF1/PINX1/CHEK1/UIMC1/RAD51C/UBE2E2/PSME3/CLSPN/CDT1/CDK5RAP3/FBXW7/NABP2/AKT1/FAM107A/SPDL1/NPM1/CNOT6L/CUL1/PSMA7/PSMB8/NDE1/PSMC5/TUBA1A/KLHL22/TRIAP1/NACC2/KNTC1/PSMB10/HSP90AA1/PPP1R9B/PSMB6/ANAPC7/PSMA4/FBX

O5/INTS3/PCBP4/KLHL18/PSMD13/PSME1/VPS4A/YWHAG/DDB1/BRCA1/USP47/RINT1/PSMC1/DTL/CCND3/ZNF830/NEDD1/FBXL7/MDM2/ZFP36L1/ZNF207/PSMB4/PLK4/CENPF/DDRGK1/ADAM17/PSMB3/CCND2/PSMD2/BUB3/PAGR1/TTK/KANK2/NEK11/HAUS7/CHFR/PSMC6/MAD2L1/PSMD7/DYNC1LI1/PSMA2/HAUS1/MRE11/PSMD4/GLI1/NEK2/HAUS2/CUL4A/MDM4/MN1/PSMD9/ERCC3/CDK2AP2/SMARCD3/HMMR/MRNIP/CNOT11/WNT10B/GTSE1/TFDP1/ATM/CKAP5
GOBP_LEUKOCYTE_MIGRATION GOBP_LEUKOCYTE_MIGRATION
GOBP_LEUKOCYTE_MIGRATION 422 0.559059823 2.109316111 0.001146789
0.030581767 0.024202852 2132 tags=35%, list=13%, signal=32%
MMP1/S100A8/CXCL5/CXCL1/CXCL3/GREM1/CXCL6/CCL11/CXCL9/S100A9/ANXA1/CXCL11/CXCL13/CXCL8/CCL18/CXCL10/PECAM1/S100A12/WNT5A/CXCL2/COL1A2/COL1A1/MMP9/SELL/SLC7A11/CXCR2/CAV1/SPNS2/SLC7A5/THBD/IL33/MSN/PLVAP/LYN/ALOX5/ITGA2/ADA/TREM1/SERPINE1/SELP/CCR2/CD44/CD74/TGFB1/PDE4B/PF4/PLA2G7/RAC2/IL27RA/SLAMF8/ECM1/CCL20/S100A7/CCR1/FCER1G/ADGRE2/FN1/ITGA5/ST3GAL4/VCAM1/IL1R1/MADCAM1/C5AR1/KIT/CCR7/CSF3R/FYN/ITGB2/CCL4/THY1/CXCR4/SELE/HCK/ICAM1/PTAFR/CCL2/CD300A/FFAR2/CCL24/CD81/RHOH/CORO1A/SDC2/SIRPA/TNFRSF10A/VEGFC/ITGAM/CKLF/CCL22/EMILIN1/JAM2/S1PR1/TEK/CH25H/CALR/VAV1/CCR6/TNFRSF10B/ANO6/PIK3CG/LRCH1/C3AR1/RIPOR2/CSF1R/CD84/LYST/LCK/PLCB1/GPR183/SELPLG/PPBP/GPSM3/CCL19/SLC7A6/PIK3CD/ITGA6/CD2/JAM3/MIF/NKX2-3/CD200/PPIB/VPREB3/CXCL14/CD47/ITGAV/IL23A/SRP54/IL6/ADAM8/CREB3/RHOG/ITGAX/FUT4/STK10/NUP85/SDC3/AIF1/FPR2/APOD/GYPC/CCR5/SELENOK/HSD3B7/NCKAP1L/TNFSF11/NOD2/INPP5D/ESAM
GOBP_RIBONUCLEOPROTEIN_COMPLEX_BIOGENESIS
GOBP_RIBONUCLEOPROTEIN_COMPLEX_BIOGENESIS
GOBP_RIBONUCLEOPROTEIN_COMPLEX_BIOGENESIS 406 0.472906714 1.783677596
0.001146789 0.030581767 0.024202852 4517 tags=48%, list=27%, signal=36%
ISG20/NOP2/AGO2/DDX21/UTP4/GTF3A/GNL2/LYAR/ERI1/RRP36/TSR1/NIFK/RRS1/RUVBL1/WDR77/EIF2S2/NOP58/RIOX2/EIF3B/PA2G4/NOP16/RRP7A/DIMT1/BYSL/MRTO4/MRPL36/NAT10/ABCE1/EXOSC3/DDX56/UTP6/RPP40/NOP14/RAN/NOC4L/TRMT112/ATR/RRP12/UTP18/NOLC1/EIF3J/MAK16/RPL7L1/DCAF13/IMP4/MTERF3/AGO3/GTPBP4/SNRPG/MPHOSPH6/UTP14A/EXOSC7/RRP15/SNRPD1/PRMT5/SF1/RRP9/EIF3I/CELF2/WDR43/FTSJ3/USP36/POLR2D/DDX18/MRPL20/RIOK1/UTP11/TBL3/DICER1/MRM2/SETX/HEATR1/RRP1B/STRAP/SDAD1/MRPS2/EXOSC9/RPL26L1/MCTS1/PAK1IP1/SNRPF/NOC2L/WDR55/NHP2/TFB1M/HSP90AB1/RRP1/DDX10/GLUL/ADAR/LTV1/SUV39H1/RUVBL2/PRPF31/SNRPB/NPM1/BMS1/LUC7L2/GEMIN6/GEMIN8/EXOSC8/BUD23/CHD7/URB1/PWP1/RPUSD4/EIF3G/WDR75/HSP90AA1/GEMIN5/LUC7L/URB2/NOL9/UTP15/UTP3/PELP1/WDR12/HEATR3/DHX30/RRP8/POP7/POP5/AATF/SRFBP1/LSM6/PIH1D1/RPF2/MPHOSPH10/DHX29/BRIX1/NOB1/MYBBP1A/PES1/LAS1L/NOL10/TXNL4A/TARBP2/EIF4A3/XRCC5/MPV17L2/SNRPD2/WDR46/SNRPC/WDR3/TEX10/SRSF1/METTL5/EMG1/EXOSC10/WDR36/DDX23/DENR/DDX27/DDX51/SART3/ZRSR2/LSG1/SFSWAP/UTP25/NOP9/SART1/DDX20/SENP3/CD2BP2/SNRPE/NOP10/SF3A2/RBIS/DDX17/NOL6/SRSF6/THUMPD1/SNU13/CUL4A/PRPF3/EXOSC1/CPSF7/KHDC4/LUC7L3/NOL11/XAB2/NPM3/NUP88/MTREX/MRM3/GEMIN4/EIF3M/PTBP2/NOL8/ATM/MALSU1/NSUN5/PUF60/NLE1/EXOSC2
GOBP_ACTIVATION_OF_IMMUNE_RESPONSE GOBP_ACTIVATION_OF_IMMUNE_RESPONSE
GOBP_ACTIVATION_OF_IMMUNE_RESPONSE 424 0.519807019 1.960738018
0.001148106 0.030581767 0.024202852 3388 tags=40%, list=20%, signal=33%
CD55/C4BPB/C4BPA/CFB/IL1B/CFI/MNDA/CD38/FCGR2A/RGCC/GBP1/MS4A1/C1S/C3/MUC

5B/FPR1/LYN/ADA/SERPING1/C2/RFTN1/C1R/LAX1/LCP2/IFI16/KLHL6/PDE4B/AIM2/HLA-DQB1/HLA-DRA/NFKBIZ/PTPRC/CLU/CBFB/THEMIS2/RIPK2/STING1/FCER1G/MUC4/PSME4/PSMB9/CLEC4A/C5AR1/ARPC1B/KCNN4/FCN1/CCR7/CD19/FYN/FCGR2B/THY1/C1QB/FGR/HCK/CD300A/MICB/FFAR2/PLCG2/TYROBP/CD81/HLA-DQA1/FPR3/HLA-DPA1/ICAM2/FCN3/WIPF1/LAPTM5/C1QA/FYB1/PSMD14/CLEC4E/DGKZ/A2M/RAB29/VAV1/CD79B/CD79A/CD276/C3AR1/PSMD12/LCK/PSMC2/MUC1/PSMB2/PRKCB/PSMA3/PIK3CD/PSMD1/FCRL3/PRNP/SH2B2/BTN3A1/CR2/BTK/PRKCH/ITK/LPXN/CD47/ELMO1/PSMA5/MUC5AC/EIF2B3/CFP/LILRB4/C1RL/PSMD6/MUC2/PSMB5/PSMC4/FPR2/ZBP1/SLC39A10/BTN3A3/RELA/NCKAP1L/NOD2/INPP5D/MFAP4/EIF2B2/CD59/PSMB1/SPG21/PSMA1/MALT1/CMKLR1/BLK/CD4/LAT2/HSP90AB1/PSME3/CD3D/SYK/CGAS/CUL1/PSMA7/PSMB8/PSMC5/WAS/LIME1/UBE2N/ACTR3/LIMK1/BTN2A2/TRIM5/NR4A3/TBK1/PSMB10/HSP90AA1/EIF2B5/HLA-DPB1/CTLA4/PROS1/PSMB6/EIF2B4/HRAS/PLSCR1/PSMA4/RC3H2/SFPQ/LAT/NLRC4/PSMD13/WASF2/PSME1/MYO1G/CD247/HMGB1/CARD11/BTN2A1/CR1

GOBP_REGULATION_OF_LYMPHOCYTE_ACTIVATION GOBP_REGULATION_OF_LYMPHOCYTE_ACTIVATION GOBP_REGULATION_OF_LYMPHOCYTE_ACTIVATION 413 0.506471865 1.91089192 0.001148106 0.030581767 0.024202852 2777 tags=35%, list=16%, signal=30% CD55/VNN1/ANXA1/CD274/IDO1/SPINK5/IL1B/MZB1/MNDA/CD38/CAV1/IL7R/LYN/ADA/SLC7A1/CCR2/LAX1/CD74/TGFB1/JAK3/RAC2/IRF4/HLA-DRA/INHBA/IL27RA/LGALS1/SLAMF8/NFKBIZ/PNP/PTPRC/HLA-DMB/CBFB/GAL/RIPK2/XBP1/TNFSF13/CLEC7A/VCAM1/HSPH1/TNFSF13B/TGFBR2/CCR7/CD19/SAMSN1/MAD2L2/FYN/FCGR2B/SHH/CEBPB/THY1/LILRB2/CD86/SASH3/FGR/CD27/CCL2/CD300A/TYROBP/CD81/LST1/CD40/RHOH/HLA-DPA1/CORO1A/SIRPA/BCL6/IL1A/LEF1/LAPTM5/IGFBP2/VAV1/BANK1/IRF1/IL6ST/CD276/RIPOR2/LCK/IL12RB1/GPR183/CCL19/METTL3/DUSP10/FANCD2/FCRL3/TNFRSF1B/RUNX3/EXOSC3/TWSG1/EBI3/PRNP/CD2/MIF/BTK/IKZF3/AHR/ITPKB/LAG3/CD47/EGR3/IL23A/IL6/ADAM8/LILRB4/AXL/TNFRSF4/TMEM131L/SFRP1/LILRB1/IFNG/AIF1/GPNMB/AP3D1/HAVCR2/SLC39A10/MLH1/PRDM1/SELENOK/NCKAP1L/TNFSF11/NOD2/INPP5D/HLX/CD320/MALT1/SIT1/BLK/CD4/ZBTB16/GLMN/SIRPG/MDK/SYK/CD6/LRRC32/TYRO3/AKT1/TNFRSF18/RASAL3/THOC1/CGAS/IL18/DOCK8/PRKAR1A/DLG5/BTN2A2

GOBP_T_CELL_ACTIVATION GOBP_T_CELL_ACTIVATION GOBP_T_CELL_ACTIVATION 448 0.491072727 1.859941296 0.001148106 0.030581767 0.024202852 2807 tags=34%, list=17%, signal=29% CD55/VNN1/ANXA1/CD274/IDO1/SPINK5/IL1B/CTPS1/CAV1/IL7R/MSN/LYN/ADA/SLC7A1/CCR2/CD44/LAX1/CD74/JAK3/RAC2/IRF4/HLA-DRA/IL27RA/LGALS1/NFKBIZ/PNP/ENTPD7/PTPRC/HLA-DMB/CBFB/TCIRG1/RIPK2/FCER1G/XBP1/CLEC7A/VCAM1/HSPH1/CLEC4A/TNFSF13B/KIT/TGFBR2/CCR7/FYN/FCGR2B/SHH/CEBPB/THY1/LILRB2/CD86/SASH3/ICAM1/CD27/CCL2/CD300A/MICB/MAFB/CD81/RHOH/HLA-DPA1/CORO1A/SIRPA/BCL6/APBB1IP/IL1A/LEF1/LAPTM5/STAT3/LCP1/IGFBP2/RAB29/VAV1/IRF1/IL6ST/CCR6/CD276/PIK3CG/RIPOR2/LCK/IL12RB1/GPR183/DOCK2/CCL19/PIK3CD/IL18R1/METTL3/DUSP10/FANCD2/TNFRSF1B/RUNX3/TWSG1/EBI3/PRNP/CD2/BTN3A1/RORA/NKX2-3/ITPKB/ITK/LAG3/CD47/EGR3/IL23A/CASP8/IL6/MR1/ADAM8/CTSL/LILRB4/TNFRSF4/TMEM131L/LILRB1/IFNG/AIF1/GPNMB/AP3D1/HAVCR2/BATF/PRDM1/JMJD6/SLAMF6/SELENOK/WNT4/NCKAP1L/TNFSF11/PPP3CA/NOD2/HLX/SEMA4A/MALT1/SIT1/CD4/LY9/RASGRP1/ZBTB16/GLMN/SIRPG/MDK/CD3D/SYK/CD6/LRRC32/AKT1/TNFRSF18/RASAL3/CGAS/IL18/DOCK8/WAS/ATP7A/PRKAR1A/CHD7/DLG5/BTN2A2/DDOST

GOBP_NCRNA_METABOLIC_PROCESS GOBP_NCRNA_METABOLIC_PROCESS GOBP_NCRNA_METABOLIC_PROCESS 445 0.408182718 1.545125706 0.001150748

0.030581767 0.024202852 5176 tags=47%, list=31%, signal=34%
WARS1/ISG20/WDR4/GARS1/NOP2/FTSJ1/AGO2/YARS1/DDX21/UTP4/PPA1/LYAR/ERI1/RRP36/IARS1/TSR1/EEF1E1/NIFK/PELO/STAT3/AARS1/RRS1/PUSL1/DTD1/NOP58/PA2G4/METTL1/RRP7A/DIMT1/BYSL/MRTO4/DARS1/SND1/METTL3/NAT10/EXOSC3/DDX56/UTP6/RPP40/NOP14/RAN/NOC4L/TRMT112/RRP12/UTP18/NOLC1/MAK16/RPL7L1/DCAF13/PUS1/LCMT2/IMP4/AGO3/GTPBP4/IL6/MPHOSPH6/UTP14A/EXOSC7/RRP15/RRP9/WDR43/FTSJ3/USP36/TARS1/DDX18/RIOK1/EPRS1/DUS1L/UTP11/TBL3/DICER1/MRM2/RELA/EARS2/CARS1/HEATR1/RRP1B/INTS5/TSEN15/PUS3/EXOSC9/PUS7/FARSA/LARS2/WDR55/SRRT/NHP2/FARSB/TFB1M/RRP1/DDX10/ADAR/SUV39H1/NSUN2/TRMT10C/BMS1/TRMT6/DUS3L/EXOSC8/BUD23/CHD7/ELP1/POP1/URB1/METTL8/TRMT1/RPUSD4/WDR75/RARS1/TYW3/NOL9/UTP15/UTP3/PELP1/WDR12/HRAS/RRP8/POP7/POP5/SRFBP1/LSM6/PIH1D1/RC3H2/HARS1/INTS3/PNPT1/RPF2/MPHOSPH10/THUMPD2/NOB1/CDK5RAP1/PES1/LAS1L/NOL10/TARBP2/USB1/EIF4A3/WDR46/SLFN13/WDR3/TRMU/THUMPD3/TEX10/METTL5/EMG1/EXOSC10/WDR36/DDX27/DDX51/CTU2/SARS1/ELAC2/RTCB/UTP25/NOP9/SART1/SENP3/LAGE3/NOP10/XPO5/DDX17/NOL6/INTS6/TRMT11/THUMPD1/SNU13/TP53RK/EXOSC1/NOL11/NPM3/MTREX/MRM3/GEMIN4/YARS2/PRKRA/NOL8/RTRAF/THADA/NSUN5/EXOSC2/INTS8/PDCD11/VARS2/FBLL1/MYBL1/ELP5/GTF2H5/TYW5/PRKDC/MARS2/DPH3/ELP6/INTS10/NFKB1/ZNHIT6/SSB/TSR2/RPF1/CARS2/AIMP1/TSR3/PIN4/TRMT10A/RIOK2/WARS2/UTP20/DDX1/MDN1/GPAT2/HNRNPA2B1

GOBP_NEGATIVE_REGULATION_OF_HYDROLASE_ACTIVITY
GOBP_NEGATIVE_REGULATION_OF_HYDROLASE_ACTIVITY
GOBP_NEGATIVE_REGULATION_OF_HYDROLASE_ACTIVITY 411 0.431910941 1.628226485
0.001150748 0.030581767 0.024202852 3030 tags=25%, list=18%, signal=21%
SERPINB5/SPINK4/PI3/TIMP1/COL6A3/TFPI2/ANXA1/SERPINA3/SPINK5/AGT/LAMP3/MMP9/SERPINB9/SERPINB3/SERPINB8/SERPINE2/C3/SERPING1/SERPINE1/SERPINA1/IFI6/SERPINB7/CD44/IFI16/PI15/TFPI/SLPI/ECM1/TNFAIP8/PAPLN/PLN/CARD16/FICD/SERPINB4/BST2/CSTA/GNAI2/CD27/SEMA4D/USP14/SERPINF1/RHOH/SERPINB6/RGS2/CST7/LTF/SERPINH1/A2M/APOC1/WFDC1/PTTG1/LRCH1/SPRED1/SPOCK2/SPINK1/ABCE1/COL7A1/PPP1R14B/SERPINI1/NLRP7/PRNP/ANXA2/KNL1/SPOCK1/CD109/RCC2/SERPINB1/CRYAB/PHACTR1/BCL2L12/RDX/TIMP2/SH3BP4/NCKAP1L/CDC42SE1/RRP1B/FURIN/SFRP2/RARRES1/SERPINF2/DNAJB6/ARPP19/LRRK2/AQP1/GPX1/TIMP3/MICAL1/ANGPTL4/AKT1/PARK7/PAM16/TRIAP1/ARRB2/FARP1/GPSM1/ROCK1/PLAUR/PPP1R9B/SPRY1/PROS1/ITIH5/HRAS

GOBP_REGULATION_OF_CELL_CELL_ADHESION GOBP_REGULATION_OF_CELL_CELL_ADHESION
GOBP_REGULATION_OF_CELL_CELL_ADHESION 407 0.493440617 1.860090608
0.001150748 0.030581767 0.024202852 2994 tags=37%, list=18%, signal=31%
IL1RN/CD55/VNN1/ANXA1/CXCL13/CD274/IDO1/IL1B/WNT5A/RGCC/CAV1/IL7R/SERPINE2/LYN/ALOX5/ADA/SLC7A1/SELP/ASS1/CCR2/CD44/LAX1/CD74/TGFB1/JAK3/PODXL/HLA-DRA/IL27RA/LGALS1/NFKBIZ/PDPN/PNP/PTPRC/HLA-DMB/CBFB/RIPK2/XBP1/ST3GAL4/VCAM1/HSPH1/TNFSF13B/TGFBR2/FXYD5/CCR7/MAD2L2/FYN/ITGB2/FCGR2B/JAK2/SHH/CEBPB/THY1/LILRB2/CD86/ADAM19/SASH3/SELE/ICAM1/CD27/PTAFR/CCL2/CD300A/PIEZO1/IRAK1/CD81/RHOH/HLA-DPA1/CORO1A/SIRPA/BCL6/IL1A/LEF1/LAPTM5/IGFBP2/VAV1/IRF1/IL6ST/CD276/RIPOR2/LCK/IL12RB1/CCL19/METTL3/DUSP10/RUNX3/TWSG1/EBI3/PRNP/ITPKB/LAG3/CD47/EGR3/IL23A/GTPBP4/IL6/ADAM8/LILRB4/FUT4/TMEM131L/LILRB1/IFNG/AIF1/GPNMB/JAG1/RDX/AP3D1/HAVCR2/SELENOK/RELA/WNT4/NCKAP1L/TNFSF11/NOD2/HLX/SERPINF2/SWAP70/MALT1/BLK/CD4/CEACAM6/ZBTB16/FSTL3/GLMN/SIRPG/FOXA2/MDK/SYK/ETS1/CD6/LRRC32/BMP6/AKT1/RASAL3/ITGA4/

IL18/DOCK8/PRKAR1A/C1QTNF1/DLG5/BTN2A2/PPM1F/JAK1/NR4A3/PLAUR/STAT5B/HLA-DPB1/AKNA/CTLA4/PTPN11

GOBP_POSITIVE_REGULATION_OF_CELL_ADHESION GOBP_POSITIVE_REGULATION_OF_CELL_ADHESION GOBP_POSITIVE_REGULATION_OF_CELL_ADHESION 409 0.502278001 1.893267217 0.001152074 0.030581767 0.024202852 3043 tags=39%, list=18%, signal=32% OLFM4/CD55/VNN1/ANXA1/CXCL13/CD274/IL1B/WNT5A/TGM2/NID1/CAV1/IL7R/FERMT2/HYAL1/LYN/ALOX5/ITGA2/ADA/SLC7A1/CCN1/SELP/CCR2/CD44/CD74/JAK3/PODXL/HLA-DRA/IL27RA/LGALS1/NFKBIZ/PDPN/PNP/PTPRC/HLA-DMB/CBFB/RIPK2/XBP1/FN1/ITGA5/ST3GAL4/VCAM1/HSPH1/TNFSF13B/EFEMP2/TGFBR2/CCR7/FYN/ITGB2/JAK2/SHH/THY1/LILRB2/CD86/ADAM19/MAP4K4/FOXF1/SASH3/SELE/ICAM1/CD27/PTAFR/CCL2/PIEZO1/EGFL6/IRAK1/CD81/RHOH/HLA-DPA1/CORO1A/SIRPA/BCL6/FLNA/APBB1IP/COL8A1/IL1A/LEF1/KDR/COL16A1/P4HB/EMILIN1/TEK/IGFBP2/CALR/VAV1/IL6ST/CD276/LCK/IL12RB1/CCL19/ADAM9/SPOCK2/AGR2/DUSP10/RUNX3/NRP1/ITGA6/EBI3/ITPKB/TFE3/CD47/ITGAV/LIF/EGR3/IL23A/PLEKHA2/IL6/ADAM8/LILRB4/FUT4/SFRP1/LILRB1/IFNG/AIF1/AP3D1/HAVCR2/SELENOK/RELA/WNT4/NCKAP1L/TNFSF11/PPP3CA/NOD2/SFRP2/HLX/SERPINF2/NPNT/MALT1/CD4/CEACAM6/FERMT1/ZBTB16/FSTL3/SIRPG/FOXA2/MDK/SYK/ETS1/CD6/AKT1/TNFRSF18/RASAL3/FBLN2/ITGA4/IL18/DOCK8/FRMD5/RHOD/ROCK1/PPM1F/JAK1/NR4A3/PLAUR/STAT5B/HLA-DPB1/CTLA4/PTPN11/EMP2/ITGA3

GOBP_REGULATION_OF_PEPTIDASE_ACTIVITY GOBP_REGULATION_OF_PEPTIDASE_ACTIVITY GOBP_REGULATION_OF_PEPTIDASE_ACTIVITY 409 0.509355083 1.919943298 0.001152074 0.030581767 0.024202852 1843 tags=25%, list=11%, signal=23% SERPINB5/SPINK4/S100A8/PI3/TIMP1/COL6A3/TFPI2/S100A9/SERPINA3/SPINK5/AGT/LAMP3/MMP9/SERPINB9/CAV1/SERPINB3/ROBO1/CASP1/SERPINB8/SERPINE2/C3/LYN/CTSH/SERPING1/F3/CCN1/SERPINE1/SERPINA1/IFI6/SERPINB7/CD44/PCOLCE/IFI16/PI15/AIM2/TFPI/SLPI/BIRC3/ECM1/TNFAIP8/F2R/RIPK2/PAPLN/FN1/BOK/ANTXR1/PSME4/CLEC7A/PSMB9/CARD16/SERPINB4/BST2/FYN/JAK2/DLC1/CSTA/MAP3K5/CD27/USP14/SERPINF1/SERPINB6/TNFRSF10A/CST7/LAPTM5/LTF/AKIRIN2/PSMD14/ASPH/STAT3/SERPINH1/VCP/XDH/A2M/SEMG1/WFDC1/CASP10/ATP2A3/TNFSF15/TNFRSF10B/PTTG1/LCK/PSMA3/SPOCK2/SPINK1/COL7A1/ATP13A2/BID/SERPINI1/NLRP7/PRNP/ANXA2/DAP/CFLAR/SPOCK1/PML/CD109/CASP8/ADRM1/SERPINB1/CRYAB/APH1B/NLRP2

GOBP_ADAPTIVE_IMMUNE_RESPONSE GOBP_ADAPTIVE_IMMUNE_RESPONSE GOBP_ADAPTIVE_IMMUNE_RESPONSE 390 0.566940887 2.130908811 0.001153403 0.030581767 0.024202852 3388 tags=43%, list=20%, signal=35% CD55/C4BPB/C4BPA/IL13RA2/ANXA1/CXCL13/CD274/IL1B/CFI/LAMP3/IL7R/TNFRSF17/C1S/SLAMF7/IL33/C3/LYN/HLA-DMA/CTSH/ADA/SERPING1/C2/CCR2/RFTN1/C1R/LAX1/CD74/TGFB1/KLHL6/HLA-DQB1/AZGP1/JAK3/IRF4/HLA-DRA/ALCAM/IL27RA/NFKBIZ/FCGR1B/ENTPD7/PTPRC/HLA-DMB/CLU/TCIRG1/RIPK2/FCER1G/TNFSF13/IL1R1/TLR8/TAP2/CLEC4A/TNFSF13B/CD19/SAMSN1/MAD2L2/FYN/FCGR2B/JAK2/LILRB2/CD86/C1QB/TRIM27/SASH3/ICAM1/CD27/TAP1/MICB/CD81/HLA-DQA1/CD40/HLA-DPA1/BCL6/LEF1/C1QA/STAT3/HLA-DOB/CD79B/CD79A/IRF1/IL4I1/IL6ST/CCR6/CTSC/PIK3CG/CD84/IL12RB1/GPR183/CCL19/PRKCB/PIK3CD/IL18R1/DUSP10/TNFRSF1B/IL18BP/EXOSC3/EBI3/BTN3A1/JAM3/CR2/RORA/BTK/ORAI1/ITK/TFE3/LAG3/IL23A/EXO1/IL6/MSH6/LAIR1/MR1/CTSL/LILRB4/C1RL/LILRB1/IFNG/JAG1/LILRB3/HAVCR2/MLH1/BATF/PRDM1/SLAMF6/BTN3A3/NBN/NOD2/INPP5D/NFKB2/HLX/SEMA4A/RNF19B/IRF7/SWAP70/MALT1/SIT1/CD4/LY9/LAT2/CD3D/SYK/CD6/SIGLEC10/THOC1/IL18/HPRT1/WAS/PVR/LIME1/ADCY7/CD48/ADG

RE1/HLA-DPB1/CTLA4/RNF8/HRAS/PRR7/EMP2/HSPD1/RC3H2/BTLA/LAT/RAB27A/SLC15A4/IL1RL1/MYO1G/CD247/PKN1/EOMES/HMGB1/CR1

GOBP_REGULATION_OF_RESPONSE_TO_BIOTIC_STIMULUS GOBP_REGULATION_OF_RESPONSE_TO_BIOTIC_STIMULUS GOBP_REGULATION_OF_RESPONSE_TO_BIOTIC_STIMULUS390 0.480931716 1.807634016 0.001153403 0.030581767 0.024202852 3916 tags=44%, list=23%, signal=35% MMP12/CD55/CXCL6/CD274/SPINK5/WNT5A/MNDA/SERPINB9/CADM1/MUC5B/LYN/SERPING1/LRP8/HTRA1/IFI16/TIGAR/AIM2/LY96/BIRC3/SLAMF8/SEC14L1/RIPK2/STING1/FCER1G/SOCS3/MUC4/PSME4/PSMB9/TLR8/PARP9/CARD16/CLEC4A/SERPINB4/FCN1/FYN/FCGR2B/JAK2/STAT1/FGR/HCK/NLRC5/SAMHD1/MICB/FFAR2/PLCG2/TYROBP/LYAR/ICAM2/GBP5/APOBEC3G/PARP14/LTF/AKIRIN2/TRIM15/PSMD14/CLEC4E/A2M/VAV1/IRF1/PSMD12/IFNAR2/PSMC2/IL12RB1/MUC1/PSMB2/PSMA3/METTL3/PSMD1/ABCE1/DUSP10/USP18/LAG3/IL23A/PSMA5/MUC5AC/MR1/ADAM8/PSMD6/LILRB1/CD180/MUC2/IFNG/PSMB5/PSMC4/FPR2/HAVCR2/ZBP1/SLAMF6/SELENOK/RELA/NOD2/SYT11/IRF7/PSMB1/PSMA1/CDC37/LY86/MALT1/PTPN1/NMI/RASGRP1/HSP90AB1/ADAR/PSME3/SYK/TRAF3/BMP6/TYRO3/POLR3G/CGAS/CUL1/PSMA7/PSMB8/PSMC5/PVR/IRF3/ARRB2/TRIM5/TRIM21/APOE/JAK1/TBK1/IRAK3/AP1G1/PSMB10/HSP90AA1/STAT5B/PTPN11/PSMB6/HRAS/IL18RAP/HSPD1/PLSCR1/PSMA4/SFPQ/UFD1/TRAFD1/NLRC4/PSMD13/IFI35/SLC15A4/PSME1/IL2RA/COCH/HMGB1/CARD11/CX3CL1/CR1/MAPKBP1/TARBP2/XRCC5/CD209/PSMC1/DHX58/ILRUN/NECTIN2/CYBA/PSMB4/DTX3L/HLA-E/MUC17/PSMB3/FADD/PSMD2/IKBKG/PSMC6/LBP/HERC5/PSMD7/MUC12/CLEC4D

GOBP_AMEBOIDAL_TYPE_CELL_MIGRATIONGOBP_AMEBOIDAL_TYPE_CELL_MIGRATION GOBP_AMEBOIDAL_TYPE_CELL_MIGRATION397 0.406382884 1.52749235 0.001154734 0.030581767 0.024202852 3086 tags=32%, list=18%, signal=27% TIMP1/GREM1/S100P/GJA1/ANXA1/CXCL13/LOXL2/FSTL1/AGT/SPARC/WNT5A/ADAMTS9/MMP9/RGCC/ROBO1/HYAL1/ITGA2/CTSH/CDH5/PIK3R3/TGFB1/STC1/ANXA3/HIF1A/PTGS2/FN1/ENPP2/S100A2/TWIST1/KIT/AGO2/TGFBR2/SMOC2/ITGB2/ANXA6/SHH/PTPRM/DCN/ANLN/MAP4K4/ARHGDIB/FAP/SRPX2/HYAL2/SEMA4D/MET/PLCG2/CD40/SERPINF1/RAB13/DDR2/MMRN2/VEGFC/KDR/TEK/TPBG/CALR/CCR6/PIK3CG/ADGRA2/SPRED1/TACSTD2/FGFR1/MCC/ADAM9/SEMA4B/PIK3CD/ITGB1BP1/CLEC14A/DUSP10/NRP1/AKAP12/HDAC7/MACF1/LPXN/ACVR1/CORO1C/EGR3/EPHB4/PLXND1/AKT3/PML/RCC2/FGFBP1/LAMA5/FGF7/ATP2B4/IFNG/VASH1/NUS1/AMOTL1/ITGB4/SEMA3G/PLK2/TGFBR1/STRAP/SEMA4A/GNA12/RHOJ/GPX1/FERMT1/GLUL/TMEM201/FBXW7/SEMA3F/ETS1/ARID5B/ANGPT2/AKT1/ITGA4/JCAD/EFNB2/APOE/ROCK1/PPM1F/FGF2/EDNRB/EVL/GPI/PTPN11/SEMA4F/EMP2/ITGA3/STAT5A/SYDE1/NR2F2/PRCP

GOBP_CALCIUM_ION_TRANSPORT GOBP_CALCIUM_ION_TRANSPORT GOBP_CALCIUM_ION_TRANSPORT 384 0.415116735 1.562302518 0.001154734 0.030581767 0.024202852 2142 tags=22%, list=13%, signal=20% GJA1/CEMIP/CXCL9/CXCL11/CXCL10/CAV1/MS4A1/KCNE3/LYN/PDE4B/GEM/TRPA1/STC1/MCUB/JPH1/APLNR/PTPRC/F2R/CCR1/RAMP3/HOMER1/PLN/KCNN4/CCR7/CD19/FYN/ANXA6/CCL4/TMEM165/THY1/LILRB2/CXCR4/SESTD1/CALCRL/TRIM27/P2RX5/GNAI2/ICAM1/CCL2/PDGFRB/PLCG2/DIAPH1/CORO1A/GRAMD2A/GNB5/SEC61A1/SEMG1/CLIC2/ERO1A/ATP2A3/SELENON/ANO6/PIK3CG/CD84/LCK/SLC24A3/RGS4/CCL19/PRKCB/GSTO1/SPINK1/FCRL3/CYP27B1/PKD2/ORAI2/PRNP/TRPV2/WFS1/ANXA2/ORAI1/IBTK/ITGAV/GJA4/PML/LILRB1/ATP2B4/ATP2A2/BSPRY/CYSLTR1/CCR5/SELENOK/PPP3CA/P2RX7/TRPC6

GOBP_GLYCOPROTEIN_METABOLIC_PROCESS GOBP_GLYCOPROTEIN_METABOLIC_PROCESS

GOBP_GLYCOPROTEIN_METABOLIC_PROCESS 389 0.391912941 1.472434434 0.001156069 0.030581767 0.024202852 2838 tags=29%, list=17%, signal=25% MMP12/SRD5A3/ADAMTS9/DERL3/BACE2/SDF2L1/CSGALNACT1/CHST15/PHLDA1/IL33/DPY19L1/HYAL1/MUC5B/FBXO6/BGN/JAK3/SULF1/PGM3/CHST11/CHSY1/VCAN/FUT8/HIF1A/MUC4/ST3GAL4/DSE/AGO2/CCR7/TMEM165/DCN/GALNT2/TMTC1/TUSC3/NPC1/SULF2/CHPF/RPN2/PMM2/ST6GALNAC4/LMAN1/B3GALT6/HS3ST3B1/CSGALNACT2/UBE2J1/ST3GAL5/GALNT6/PLCB1/MUC1/DPAGT1/B3GALNT2/CCL19/MCFD2/OSTC/GFPT2/SPOCK2/MGAT1/GALNT10/GALNT18/HPSE/ALG5/EXT2/ST6GAL2/UGGT2/ALG2/SLC35C1/FUT11/MUC5AC/CHST12/TMEM258/ST3GAL2/ITM2A/DPY19L3/CTSL/FUT4/GALNT5/MUC2/PLOD3/ERP44/MAN2B1/B3GNT7/NUS1/DERL2/B3GNT6/SERP1/DOLK/EDEM1/ALG8/RPN1/ALG9/MAN1A2/B4GALT2/KRTCAP2/B4GALT6/HS6ST2/OGT/RFT1/STT3A/POGLUT1/EOGT/UGGT1/CHPF2/MVD/CHST3/ST6GALNAC2/PARK7/ST6GAL1/ATP7A/DDOST/EXTL2/GALNT8/GANAB/GPC1

GOBP_MONONUCLEAR_CELL_DIFFERENTIATION GOBP_MONONUCLEAR_CELL_DIFFERENTIATION GOBP_MONONUCLEAR_CELL_DIFFERENTIATION 392 0.453393844 1.703345713 0.001156069 0.030581767 0.024202852 2751 tags=31%, list=16%, signal=27% VNN1/POU2AF1/ANXA1/SPINK5/IL1B/MS4A1/IL7R/LYN/ADA/CCR2/CD74/IFI16/JAK3/IRF4/HLA-DRA/INHBA/LGALS1/SLAMF8/NFKBIZ/PNP/ENTPD7/PTPRC/DNAJB9/CBFB/TCIRG1/RIPK2/FCER1G/XBP1/VCAM1/KIT/TGFBR2/CCR7/CD19/FCGR2B/SHH/CEBPB/LILRB2/CD86/SASH3/CD27/HHEX/MAFB/PLCG2/RHOH/CMTM7/BCL6/IL1A/LEF1/MFNG/FASN/STAT3/VAV1/CD79B/CD79A/IRF1/CCR6/EZH2/CSF1R/LCK/IL12RB1/DOCK11/GPR183/DOCK2/CCL19/PIK3CD/IL18R1/METTL3/TCF3/DUSP10/FANCD2/BATF2/FCRL3/RUNX3/CD2/CR2/RORA/NKX2-3/BTK/IKZF3/ITPKB/ITK/LAG3/EGR3/IL23A/IL6/MR1/ADAM8/ITM2A/CTSL/LILRB4/AXL/TMEM131L/SFRP1/LILRB1/IFNG/BATF3/AP3D1/BATF/PRDM1/JMJD6/SLAMF6/WNT4/NCKAP1L/INPP5D/HLX/SEMA4A/IRF7/MALT1/BLK/CD4/LY9/ZBTB16/MDK/CD3D/SYK/LRRC8A/TYRO3/TNFRSF18/DLL1/ITGA4/IL18/ATP7A/CHD7

GOBP_NEGATIVE_REGULATION_OF_TRANSPORT GOBP_NEGATIVE_REGULATION_OF_TRANSPORT GOBP_NEGATIVE_REGULATION_OF_TRANSPORT 391 0.387701333 1.456970911 0.001156069 0.030581767 0.024202852 2140 tags=23%, list=13%, signal=20% GJA1/IL13RA2/ANXA1/IL1B/DERL3/MMP9/CAV1/KCNE3/SERPINE2/TFF2/ADA/PEA15/CCR2/CD74/MCTP1/GEM/STC1/INHBA/BAG3/GRB10/F2R/PIM3/PLN/UCP2/TWIST1/BST2/FCGR2B/VSNL1/SHH/LILRB2/SESTD1/TRIM27/FOXF1/TLR2/GNAI2/ICAM1/CD300A/RHBDF2/IDH2/VIP/PCSK9/CORO1A/SIRPA/RGS2/STXBP6/ERLEC1/LMAN1/GNB5/IRS1/TRIM15/RAB23/UBE2J1/SEMG1/CLIC2/APOC1/NMU/INSIG1/FFAR4/CD84/RGS4/PRKCB/ITGB1BP1/GSTO1/BARD1/SPINK1/WDR54/TNFRSF1B/PKD2/CD200/CD47/ITGAV/RANGAP1/SDCBP/LIF/OAZ3/CD300LF/CRYAB/NEDD4/SFRP1/MAP1B/LILRB1/PKIA/RHBDF1/APOD/DERL2/PPP3CA/RUBCN/SYT11

GOBP_OSSIFICATION GOBP_OSSIFICATION GOBP_OSSIFICATION 369 0.410813686 1.543210191 0.001157407 0.030581767 0.024202852 2602 tags=28%, list=15%, signal=24% CTHRC1/IGFBP5/TNC/GREM1/SPP1/MGP/SPARC/WNT5A/COL1A2/COL1A1/MMP9/CSGALNACT1/COL5A2/CDH11/FERMT2/MMP2/ALOX5/COL6A1/CCN1/TGFB1/SRGN/IFITM1/TPM4/STC1/CHSY1/ECM1/VCAN/CBFB/TCIRG1/HIF1A/CCR1/RUNX2/SBNO2/TWIST1/DDX21/SHH/CEBPB/CHRDL2/FGR/LOX/SEMA4D/SNX10/CREB3L1/RASSF2/SNAI2/DDR2/VEGFC/FHL2/IARS1/LEF1/LTF/S1PR1/TEK/FASN/WWTR1/IL6ST/ANO6/FFAR4/RFLNB/PSMC2/SLC24A3/DKK1/SND1/CYP27B1/RUNX

3/TWSG1/HDAC7/EXT2/DCHS1/ACVR1/ACP5/GTPBP4/SYNCRIP/IL6/SFRP1/JAG1/MRC2/TMEM119/SMAD5/CLEC5A/WNT4/TMEM64/RIOX1/TNFSF11/P2RX7/FIGNL1/ALPL/SFRP2/RRBP1/NAB1/ASPN/NPNT/MESD/ZBTB16/FSTL3/TGFB3/MDK/ADAR/NAB2/ATF4/BMP6/AKT1

GOBP_REGULATION_OF_HEMOPOIESIS GOBP_REGULATION_OF_HEMOPOIESIS GOBP_REGULATION_OF_HEMOPOIESIS 381 0.412666045 1.551116711 0.001157407 0.030581767 0.024202852 2623 tags=28%, list=15%, signal=24% VNN1/ANXA1/SPINK5/IL7R/LYN/ADA/CCR2/FBN1/HCLS1/CD74/TGFB1/PF4/JAK3/EVI2B/IRF4/HLA-DRA/INHBA/SLAMF8/NFKBIZ/NME1/TCIM/PNP/PTPRC/CBFB/RIPK2/HIF1A/CCR1/XBP1/TGFBR2/CSF3R/FCGR2B/GPR137B/SHH/CEBPB/LILRB2/CD86/STAT1/PIAS3/SASH3/MYL9/CD27/LOX/MAFB/TYROBP/RHOH/BCL6/RASSF2/LEF1/LTF/STAT3/IRF1/MEIS1/CLDN18/IL12RB1/CCL19/PRKCB/TESC/METTL3/DUSP10/FANCD2/FCRL3/RUNX3/CD2/BTK/SLC9B2/IKZF3/PRMT1/ITPKB/TFE3/LAG3/LIF/EGR3/IL23A/AGO3/CASP8/ADAM8/LILRB4/AXL/TMEM131L/SFRP1/LILRB1/IFNG/JAG1/LILRB3/CREB1/AP3D1/PRDM1/GPR171/TMEM64/NCKAP1L/TNFSF11/INPP5D/HLX/IRF7/MALT1/PAF1/CD4/HAX1/ZBTB16/FSTL3/MDK/SYK/NCAPG2/FBXW7/ETS1/TNFRSF18/DLL1

GOBP_EPITHELIAL_CELL_PROLIFERATION GOBP_EPITHELIAL_CELL_PROLIFERATION GOBP_EPITHELIAL_CELL_PROLIFERATION 368 0.434590506 1.630291198 0.001162791 0.030581767 0.024202852 2191 tags=24%, list=13%, signal=21% REG1A/REG3A/SERPINB5/MMP12/IGFBP5/CCL11/GJA1/LOXL2/PROK2/SDR16C5/SPARC/WNT5A/CLDN1/RGCC/CAV1/CDH3/ROBO1/LAMC1/HYAL1/ALOX5/F3/HTRA1/TGFB1/SULF1/MYDGF/NME1/APLNR/ECM1/HIF1A/XBP1/TWIST1/C5AR1/KIT/SHH/CEBPB/PTPRM/STAT1/CAV2/TIE1/FAP/CCL2/SULF2/CCL24/SERPINF1/VIP/SNAI2/VEGFC/COL8A1/KDR/TEK/UHRF1/XDH/WDR77/WFDC1/FGFR1/MCC/LRG1/LAMB1/PIK3CD/ITGB1BP1/MAGED1/IGFBP4/DUSP10/RUNX3/CCND1/NRP1/PPP1R16B/CFLAR/FST/EGR3/AKT3/CD109/IL6/SERPINB1/FGFBP1/SFRP1/FGF7/MAP2K1/ERN1/CDKN1C/VASH1/ANG/EGFL7/TNFSF11/NOD2/TGFBR1/SFRP2/STRAP

GOBP_IMMUNE_RESPONSE_REGULATING_SIGNALING_PATHWAY GOBP_IMMUNE_RESPONSE_REGULATING_SIGNALING_PATHWAY GOBP_IMMUNE_RESPONSE_REGULATING_SIGNALING_PATHWAY 378 0.476748548 1.789147176 0.001164144 0.030581767 0.024202852 3388 tags=39%, list=20%, signal=32% MNDA/CD38/FCGR2A/GBP1/MS4A1/MUC5B/FPR1/LYN/CTSH/ADA/RFTN1/LAX1/LCP2/KLHL6/PDE4B/HLA-DQB1/HLA-DRA/NFKBIZ/FCGR1B/PTPRC/CBFB/THEMIS2/RIPK2/FCER1G/MUC4/PSME4/PSMB9/CLEC4A/C5AR1/KIT/ARPC1B/KCNN4/FCN1/CCR7/CD19/FYN/FCGR2B/THY1/LILRB2/FGR/HCK/CD300A/MICB/FFAR2/PLCG2/TYROBP/CD81/HLA-DQA1/CD40/FPR3/HLA-DPA1/ICAM2/WIPF1/LAPTM5/FYB1/PSMD14/CLEC4E/DGKZ/RAB29/VAV1/CD79B/CD79A/CD276/C3AR1/PSMD12/LCK/PSMC2/MUC1/PSMB2/PRKCB/PSMA3/PIK3CD/PSMD1/FCRL3/PRNP/SH2B2/BTN3A1/CR2/BTK/PRKCH/ITK/LPXN/CD47/ELMO1/PSMA5/MUC5AC/EIF2B3/LILRB4/PSMD6/LILRB1/MUC2/PSMB5/PSMC4/FPR2/SLC39A10/BTN3A3/RELA/NCKAP1L/PPP3CA/INPP5D/EIF2B2/PSMB1/SPG21/PSMA1/MALT1/CMKLR1/BLK/CD4/PPP3R1/LAT2/HSP90AB1/PSME3/CD3D/SYK/CUL1/PSMA7/PSMB8/PSMC5/WAS/LIME1/UBE2N/ACTR3/LIMK1/BTN2A2/NR4A3/PSMB10/HSP90AA1/EIF2B5/HLA-DPB1/CTLA4/NFATC3/PSMB6/EIF2B4/HRAS/PLSCR1/PSMA4/RC3H2/BTLA/LAT/PSMD13/SHC1/WASF2/PSME1/MYO1G/CD247/CARD11/BTN2A1/CR1

GOBP_REGULATION_OF_IMMUNE_EFFECTOR_PROCESS GOBP_REGULATION_OF_IMMUNE_EFFECTOR_PROCESS GOBP_REGULATION_OF_IMMUNE_EFFECTOR_PROCESS 378 0.506173017 1.899571645

0.001164144 0.030581767 0.024202852 2123 tags=29%, list=13%, signal=26%
MMP12/CD55/C4BPB/CXCL6/C4BPA/IL13RA2/ANXA1/CFB/SPINK5/IL1B/CFI/MZB1/WNT5A/SERPINB9/CADM1/SLC7A5/IL7R/C1S/IL33/NOS2/C3/LYN/SERPING1/HTRA1/C2/CCR2/C1R/CD74/TGFB1/AIM2/AZGP1/JAK3/RAC2/IRF4/HLA-DRA/STXBP1/IL27RA/SLAMF8/NFKBIZ/PTPRC/HLA-DMB/CLU/SEC14L1/RIPK2/STING1/ADGRE2/XBP1/TNFSF13/CLEC7A/IL1R1/PARP9/C5AR1/SERPINB4/CD19/BST2/MAD2L2/ITGB2/FCGR2B/DDX21/CD86/STAT1/C1QB/FOXF1/SASH3/FGR/ICAM1/PTAFR/CD300A/HK1/MICB/FFAR2/TYROBP/CD81/CD40/BCL6/APOBEC3G/ITGAM/LAPTM5/C1QA/GPRC5B/A2M/VAV1/C3AR1/CD84/IL12RB1/CCL19/IL18R1/DUSP10/FCRL3/TNFRSF1B/EXOSC3/CR2/BTK/LAG3/CD47/IL23A/IL6/MR1/CFP/LILRB4/TNFRSF4/LILRB1/IFNG/HAVCR2/MLH1/SLAMF6/SELENOK/RBP4/NOD2

GOBP_EXTERNAL_ENCAPSULATING_STRUCTURE_ORGANIZATION
GOBP_EXTERNAL_ENCAPSULATING_STRUCTURE_ORGANIZATION
GOBP_EXTERNAL_ENCAPSULATING_STRUCTURE_ORGANIZATION 376 0.628758315
2.359432176 0.001165501 0.030581767 0.024202852 2289 tags=39%, list=14%, signal=34%
MMP1/MMP3/SERPINB5/MMP10/MMP12/TIMP1/TNC/COL6A3/GREM1/COL4A1/COL15A1/SPP1/LOXL2/COL12A1/SPINK5/PECAM1/TGFBI/CTSK/AGT/SPARC/ADAMTS9/VWF/MMP7/COL1A2/COL1A1/MMP9/NID1/CSGALNACT1/ADAMTS1/RGCC/CAV1/COL5A2/PXDN/MMP2/LAMC1/COL3A1/CRISPLD2/COL6A2/COL6A1/ITGA2/ENG/LAMC2/LAMA3/CCN1/SERPINE1/HTRA1/LOXL1/FBN1/CD44/COL4A2/TGFB1/BGN/PRDX4/SULF1/COL18A1/COL5A1/TNFRSF11B/LUM/PDPN/AEBP1/VCAN/LAMA4/PAPLN/FN1/ITGA5/ANTXR1/VCAM1/MADCAM1/EFEMP2/PDGFRA/SMOC2/ITGB2/SH3PXD2B/DCN/CAV2/TIE1/ADAM19/POSTN/FOXF1/FAP/ICAM1/LOX/SULF2/EGFL6/ICAM2/CREB3L1/DDR2/QSOX1/ITGAM/COL8A1/KDR/COL16A1/ITGA8/EMILIN1/JAM2/FBLN5/LCP1/SERPINH1/VWA1/A2M/COL14A1/ERO1A/MFAP5/MFAP2/LAMB1/SPOCK2/COL7A1/TNFRSF1B/ITGA9/ITGA6/LAMB2/FMOD/JAM3/FSCN1/ADAMTS5/CFLAR/CD47/ITGAV/NID2/ADAM15/IL6/ADAM8/ITGAX/CTSL/CRTAP/LAMA5/PLOD3/CTSG/AGRN/ADAMTS15/MPV17/TIMP2/PHLDB2/MMP25/ITGB4/TPSAB1/TGFBR1/FURIN/SFRP2/MFAP4/NFKB2/COL4A5/SERPINF2/KIF9/RAMP2/NPNT

GOBP_POST_TRANSLATIONAL_PROTEIN_MODIFICATION
GOBP_POST_TRANSLATIONAL_PROTEIN_MODIFICATION
GOBP_POST_TRANSLATIONAL_PROTEIN_MODIFICATION 349 0.424136904 1.586607351
0.001168224 0.030581767 0.024202852 3753 tags=32%, list=22%, signal=25%
TIMP1/IGFBP5/TNC/SPP1/FSTL1/KLHL5/IGFBP7/LAMC1/C3/CKAP4/SPARCL1/CCN1/SERPINA1/FBN1/FBXO6/CALU/LGALS1/VCAN/P3H1/HIF1A/SOCS3/RCN1/FN1/FAM20C/PSME4/PSMB9/DNAJC3/APOL1/TTL/PDIA6/PCSK9/RAB13/SDC2/QSOX1/P4HB/PSMD14/VWA1/PSMD12/PSMC2/PSMB2/LAMB1/PSMA3/PSMD1/IGFBP4/HSP90B1/LAMB2/MFGE8/WFS1/UCHL3/MXRA8/DCAF13/PSMA5/COPS3/KCTD6/IL6/CRTAP/PSMD6/PSMB5/PSMC4/UBE2F/SHISA5/PSMB1/PSMA1/STT3A/EVA1A/CCNF/ZBTB16/FSTL3/PSME3/FBXW7/ELOC/FBXO4/FUCA2/CUL1/PSMA7/PSMB8/DDB2/PSMC5/KLHL22/RAB38/KBTBD8/APOE/COPS8/KLHL21/COPS4/PSMB10/PSMB6/KLHL2/PSMA4/PSMD13/LRRC41/WDR5/PSME1/DDB1/MBTPS1/FBXO22/ICMT/DSCC1/PSMC1/KBTBD6/DTL/FBXL7/SKP2/COPS2/RHBDD1/UBE2M/PSMB4/PSMB3/STC2/PSMD2

GOBP_NEGATIVE_REGULATION_OF_IMMUNE_SYSTEM_PROCESS
GOBP_NEGATIVE_REGULATION_OF_IMMUNE_SYSTEM_PROCESS
GOBP_NEGATIVE_REGULATION_OF_IMMUNE_SYSTEM_PROCESS 365 0.488877015
1.831455864 0.001169591 0.030581767 0.024202852 2780 tags=33%, list=16%,

signal=29%

MMP12/CD55/C4BPB/GREM1/C4BPA/IL13RA2/ANXA1/CD274/IDO1/SPINK5/MNDA/SERPINB9/GBP1/IL7R/IL33/COL3A1/LYN/ADA/SERPING1/HTRA1/CCR2/FBN1/LAX1/CD74/IFI16/TGFB1/JAK3/INHBA/IL27RA/SLAMF8/NME1/PTPRC/CBFB/SEC14L1/GAL/SERPINB4/SAMSN1/BST2/FCGR2B/GPR137B/SHH/CEBPB/THY1/LILRB2/CD86/PIAS3/TRIM27/ADGRF5/FOXF1/FGR/CCL2/CD300A/NLRC5/SAMHD1/MICB/MAFB/TYROBP/LST1/LYAR/BCL6/CST7/PARP14/LAPTM5/EMILIN1/LTF/DGKZ/HLA-DOB/A2M/BANK1/IRF1/LRCH1/CLDN18/RIPOR2/CD84/PLCB1/METTL3/DUSP10/FCRL3/RUNX3/TWSG1/PRNP/MIF/BTK/CD200/LAG3/LPXN/CD47/CD300LF/LILRB4/AXL/TMEM131L/SFRP1/LILRB1/GPNMB/LILRB3/APOD/HAVCR2/NOD2/INPP5D/SYT11/HLX/TSC22D3/CD59/PSMA1/BLK/NMI/GPX1/FSTL3/GLMN/TGFB3/MDK/ADAR/FBXW7/LRRC32/TYRO3/AKT1/THOC1/PRKAR1A/ARRB2/DLG5/BTN2A2/TRIM21

GOBP_NEGATIVE_REGULATION_OF_RESPONSE_TO_EXTERNAL_STIMULUS

GOBP_NEGATIVE_REGULATION_OF_RESPONSE_TO_EXTERNAL_STIMULUS

GOBP_NEGATIVE_REGULATION_OF_RESPONSE_TO_EXTERNAL_STIMULUS 335

0.435804693 1.625157987 0.001175088 0.030581767 0.024202852 3528

tags=34%, list=21%, signal=27%

MMP12/GREM1/GJA1/CXCL13/SPP1/SPINK5/PLAU/WNT5A/SERPINB9/ROBO1/THBD/NUCB2/TNFAIP6/SERPINE2/ALOX5/ADA/SERPING1/SERPINE1/HTRA1/CDH5/IFI16/TFPI/SLAMF8/PTPRC/SEC14L1/SOCS3/CARD16/SERPINB4/PDGFRA/FCGR2B/CALCRL/PLAT/FOXF1/FAP/CCL2/NLRC5/SEMA4D/SAMHD1/MICB/SERPINF1/LYAR/SIRPA/CST7/PARP14/LTF/TEK/A2M/WFDC1/FFAR4/SEMA4B/METTL3/DUSP10/TNFRSF1B/NRP1/PBK/MIF/RORA/CD200/CD109/PTGIS/LILRB1/AIF1/FPR2/HAVCR2/PHLDB2/SEMA3G/WNT4/PTPRS/NOD2/SYT11/SEMA4A/SERPINF2/PLXNA3/PSMA1/F12/NMI/GPX1/MDK/ADAR/SEMA3F/BACE1/ANGPT2/SIGLEC10/TYRO3/C1QTNF1/ARRB2/DVL1/TRIM21/APOE/FGF2/PLAUR/IRAK3/CD34/GRIN3A/SEMA4F/PROS1/GSTP1/CHD8/UFD1/OTULIN/SELENOS/TRAFD1/IL2RA/USF1/CX3CL1/CR1/MAPKBP1/TARBP2/EPHA4/NOS3/SERPINB2/NENF/LDLR/DHX58

GOBP_REGULATION_OF_PROTEIN_CATABOLIC_PROCESS

GOBP_REGULATION_OF_PROTEIN_CATABOLIC_PROCESS

GOBP_REGULATION_OF_PROTEIN_CATABOLIC_PROCESS 361 0.396949855 1.485553251

0.001175088 0.030581767 0.024202852 2950 tags=27%, list=17%, signal=22%

TIMP1/C4BPB/LPCAT1/C4BPA/GJA1/IL1B/WNT5A/TRIB2/LAMP3/CAV1/IL33/MSN/NOS2/SERPINE2/BAG2/FAM83D/CLU/ECSCR/TRIM40/MAD2L2/FYN/SHH/ODC1/RNF144B/CDC20/DACT1/CD81/USP14/VIP/PCSK9/FLNA/LAPTM5/DAB2/PSMD14/VCP/MYCBP2/CSNK1E/CTSC/DESI1/PSMC2/ADAM9/SNRNP70/AURKA/PSMD1/BARD1/TNFRSF1B/ATP13A2/PLK1/PBK/ANXA2/SDCBP/OAZ3/PML/ADAM8/NEDD4/IFNG/PSMC4/RHBDF1/RDX/TIMP2/MAP1A/RELA/PLK2/NRDC/FURIN/SEC22B/RNF19B/HECW2/TMF1/OGT/LRRK2/GNA12/FOXF2/GPX1/GLMN/HSP90AB1/TIMP3/PSME3/CDK5RAP3/FBXW7/AKT1/PARK7/HSPBP1/SGSM3/DVL1/APOE/ROCK1/STX5/USP13/CBFA2T3/SF3B3/SOCS4/IRAK3/SMURF1/HSP90AA1/CCAR2

GOBP_NCRNA_PROCESSING GOBP_NCRNA_PROCESSING GOBP_NCRNA_PROCESSING 360

0.415253641 1.554067382 0.001176471 0.030581767 0.024202852 4659

tags=44%, list=28%, signal=33%

ISG20/WDR4/NOP2/FTSJ1/AGO2/DDX21/UTP4/LYAR/ERI1/RRP36/TSR1/NIFK/STAT3/AARS1/RRS1/PUSL1/NOP58/PA2G4/METTL1/RRP7A/DIMT1/BYSL/MRTO4/METTL3/NAT10/EXOSC3/DDX56/UTP6/RPP40/NOP14/NOC4L/TRMT112/RRP12/UTP18/NOLC1/MAK16/RPL7L1/DCAF13/PUS1/

LCMT2/IMP4/AGO3/GTPBP4/IL6/MPHOSPH6/UTP14A/EXOSC7/RRP15/RRP9/WDR43/FTSJ3/USP36/DDX18/RIOK1/DUS1L/UTP11/TBL3/DICER1/MRM2/HEATR1/RRP1B/INTS5/TSEN15/PUS3/EXOSC9/PUS7/WDR55/SRRT/NHP2/TFB1M/RRP1/DDX10/ADAR/SUV39H1/NSUN2/TRMT10C/BMS1/TRMT6/DUS3L/EXOSC8/BUD23/CHD7/ELP1/POP1/URB1/METTL8/TRMT1/RPUSD4/WDR75/TYW3/NOL9/UTP15/UTP3/PELP1/WDR12/RRP8/POP7/POP5/SRFBP1/LSM6/PIH1D1/INTS3/RPF2/MPHOSPH10/THUMPD2/NOB1/CDK5RAP1/PES1/LAS1L/NOL10/TARBP2/USB1/EIF4A3/WDR46/WDR3/TRMU/THUMPD3/TEX10/METTL5/EMG1/EXOSC10/WDR36/DDX27/DDX51/CTU2/SARS1/ELAC2/RTCB/UTP25/NOP9/SART1/SENP3/LAGE3/NOP10/DDX17/NOL6/INTS6/TRMT11/THUMPD1/SNU13/TP53RK/EXOSC1/NOL11/NPM3/MTREX/MRM3/GEMIN4/PRKRA/NOL8/RTRAF/THADA/NSUN5/EXOSC2/INTS8/PDCD11/FBLL1/ELP5/GTF2H5/TYW5

GOBP_CELL_SUBSTRATE_ADHESION GOBP_CELL_SUBSTRATE_ADHESION GOBP_CELL_SUBSTRATE_ADHESION 339 0.47895047 1.785158082 0.001177856 0.030581767 0.024202852 3311 tags=36%, list=20%, signal=29% OLFM4/MMP12/GREM1/PLAU/ADAMTS9/VWF/COL1A1/NID1/GBP1/FERMT2/LAMC1/COL3A1/ITGA2/CCN1/SERPINE1/CD44/RAC2/LGALS1/PDPN/MUC4/ZYX/FN1/ITGA5/ANTXR1/VCAM1/MADCAM1/EFEMP2/CCR7/ITGB2/JAK2/DLC1/THY1/MAP4K4/POSTN/FOXF1/EGFL6/ACTN1/BCR/CORO1A/BCL6/TRIP6/FLNA/COL8A1/KDR/COL16A1/ITGA8/P4HB/EMILIN1/TEK/FBLN5/CALR/KIF14/LIMCH1/TACSTD2/SGCE/ADAM9/LAMB1/SPOCK2/ITGB1BP1/AGR2/LYPD5/NRP1/ITGA6/LAMB2/JAM3/MACF1/HPSE/PARVB/LPXN/CORO1C/ITGAV/NID2/DUSP22/SPOCK1/ADAM15/VCL/PLEKHA2/AJUBA/RCC2/AXL/SFRP1/LAMA5/SNED1/JAG1/APOD/LYVE1/MICALL2/PHLDB2/FZD4/ITGB4/WNT4/NPNT/PARVA/PARVG/CEACAM6/FERMT1/COL5A3/MDK/ANGPT2/TYRO3/FAM107A/FBLN2/ITGA4/FBLN1/RHOD/ROCK1/PPM1F/ITGA7/CD34/CTTN/EMP2/ITGA3/HSD17B12/STK4/CDH13/GPM6B/LDB1/TBCD/NF2/MYO1G/FERMT3

GOBP_LEUKOCYTE_CELL_CELL_ADHESION GOBP_LEUKOCYTE_CELL_CELL_ADHESION GOBP_LEUKOCYTE_CELL_CELL_ADHESION 339 0.525233949 1.957667207 0.001177856 0.030581767 0.024202852 2777 tags=37%, list=16%, signal=32% S100A8/CD55/VNN1/S100A9/ANXA1/CD274/IDO1/IL1B/PECAM1/SELL/CAV1/IL7R/MSN/LYN/ALOX5/ADA/SLC7A1/SELP/ASS1/CCR2/CD44/LAX1/CD74/JAK3/RAC2/HLA-DRA/IL27RA/LGALS1/NFKBIZ/PNP/PTPRC/HLA-DMB/CBFB/RIPK2/XBP1/ITGA5/ST3GAL4/VCAM1/HSPH1/TNFSF13B/MADCAM1/TGFBR2/CCR7/FYN/ITGB2/FCGR2B/SHH/CEBPB/THY1/LILRB2/CD86/SASH3/SELE/ICAM1/CD27/PTAFR/CCL2/CD300A/SEMA4D/IRAK1/CD81/RHOH/HLA-DPA1/CORO1A/SIRPA/BCL6/IL1A/LEF1/LAPTM5/JAM2/IGFBP2/VAV1/IRF1/IL6ST/CD276/RIPOR2/LCK/IL12RB1/SELPLG/CCL19/DUSP10/RUNX3/TWSG1/EBI3/PRNP/ITPKB/LAG3/CD47/EGR3/IL23A/IL6/ADAM8/LILRB4/FUT4/TMEM131L/STK10/LILRB1/IFNG/AIF1/GPNMB/AP3D1/HAVCR2/SELENOK/RELA/NCKAP1L/TNFSF11/NOD2/HLX/MALT1/CD4/ZBTB16/GLMN/SIRPG/MDK/SYK/ETS1/CD6/LRRC32/AKT1/RASAL3/ITGA4/IL18/DOCK8/PRKAR1A/DLG5/BTN2A2

GOBP_POSITIVE_REGULATION_OF_DEFENSE_RESPONSE GOBP_POSITIVE_REGULATION_OF_DEFENSE_RESPONSE GOBP_POSITIVE_REGULATION_OF_DEFENSE_RESPONSE 330 0.547594807 2.04135132 0.001177856 0.030581767 0.024202852 3916 tags=48%, list=23%, signal=37% S100A8/MMP12/C2CD4A/GJA1/PLA2G2A/S100A9/IDO1/IL1B/S100A12/AGT/WNT5A/TGM2/MNDA/CADM1/IL33/C3/MUC5B/LYN/ITGA2/SERPINE1/CCR2/IFI16/AIM2/PLA2G7/LPL/NFKBIZ/RIPK2/PTGS2/STING1/FCER1G/MUC4/PSME4/PSMB9/TLR8/PARP9/CLEC4A/FCN1/CCR7/FYN/JAK2/CEBPB/TLR2/HCK/ABCC1/NLRC5/HYAL2/FFAR2/CCL24/PLCG2/TYROBP/CD81/ICAM2/GBP5/AKIRI

N2/PSMD14/GPRC5B/CLEC4E/VAV1/IL6ST/CTSC/PIK3CG/PSMD12/PSMC2/MUC1/GPSM3/PSMB2/PSMA3/PSMD1/BTK/LAG3/CD47/IL23A/PSMA5/MUC5AC/IL6/TSLP/ADAM8/PSMD6/MUC2/IFNG/PSMB5/PSMC4/FPR2/HAVCR2/ZBP1/SLAMF6/GPR4/RELA/TNFSF11/NOD2/IRF7/PSMB1/PSMA1/MALT1/IL16/LRRK2/NMI/RASGRP1/MDK/PSME3/SYK/ETS1/PARK7/POLR3G/CGAS/IL18/CUL1/PSMA7/PSMB8/PSMC5/PVR/PLA2G3/IRF3/TRIM5/TBK1/AP1G1/PSMB10/HSP90AA1/STAT5B/PSMB6/HRAS/TLR10/IL18RAP/PLSCR1/PSMA4/SFPQ/OSMR/NLRC4/PSMD13/IFI35/SLC15A4/PSME1/IL1RL1/COCH/HMGB1/CARD11/CX3CL1/XRCC5/CD209/PSMC1/NECTIN2/CYBA/SUCNR1/PSMB4/HLA-E/MUC17/PSMB3/FADD/PSMD2/IKBKG/CCN4/STAP1/PSMC6/LBP/PSMD7/MUC12/CLEC4D

GOBP_COAGULATION GOBP_COAGULATION GOBP_COAGULATION 326 0.452072233 1.684194411 0.001179245 0.030581767 0.024202852 3008 tags=30%, list=18%, signal=25%

C4BPB/TFPI2/PLAU/VWF/COL1A2/COL1A1/SLC7A11/CAV1/THBD/GNA15/F2RL2/ANXA5/COL3A1/SERPINE2/LYN/ITGA2/SERPING1/F3/SERPINE1/SELP/SERPINA1/LCP2/HBB/PF4/TFPI/STXBP1/PDPN/F2R/FCER1G/PRKAR2B/FN1/ST3GAL4/GNA14/PDGFRA/FYN/ENTPD1/JAK2/PLEK/SHH/P2RX5/PLAT/MYL9/FAP/PLCG2/CD40/FLNA/PROCR/LMAN1/ZFPM2/DGKZ/A2M/SEMG1/VAV1/IRF1/CBX5/ANO6/PIK3CG/LCK/DOCK11/PRKCB/SH2B2/HPSE/PRKCH/PIK3R5/VCL/EHD2/IL6/AXL/PEAR1/PHF21A/TLN1/MMRN1/PABPC4/P2RX7/TRPC6/VKORC1/CD59/SERPINF2/DOCK9/GNA12/F12/BLK/RAD51C/FOXA2/SYK/MICAL1/TYRO3/DOCK8/WAS/PRKAR1A/C1QTNF1/ARRB2/FBLN1/APOE/DGKA/PLAUR/CD34/PTPN11/PROS1

GOBP_POSITIVE_REGULATION_OF_CELL_ACTIVATION GOBP_POSITIVE_REGULATION_OF_CELL_ACTIVATION GOBP_POSITIVE_REGULATION_OF_CELL_ACTIVATION 320 0.496291579 1.845731596 0.001179245 0.030581767 0.024202852 2688 tags=36%, list=16%, signal=31%

CD55/VNN1/ANXA1/CD274/IL1B/WNT5A/CD38/CAV1/IL7R/IL33/LYN/ADA/SLC7A1/SELP/CCR2/CD74/TGFB1/JAK3/HLA-DRA/STXBP1/IL27RA/LGALS1/NFKBIZ/PNP/PTPRC/HLA-DMB/CBFB/RIPK2/XBP1/TNFSF13/CLEC7A/VCAM1/HSPH1/TNFSF13B/TGFBR2/CCR7/MAD2L2/FYN/ITGB2/JAK2/PLEK/SHH/THY1/LILRB2/CD86/SASH3/FGR/CD27/PTAFR/CCL2/PDGFRB/TYROBP/CD81/CD40/RHOH/HLA-DPA1/CORO1A/SIRPA/BCL6/ITGAM/IL1A/LEF1/IGFBP2/VAV1/IL6ST/CD276/CTSC/LCK/IL12RB1/GPR183/CCL19/DUSP10/FCRL3/RUNX3/EXOSC3/EBI3/CD2/MIF/BTK/ITPKB/CD47/EGR3/IL23A/IL6/TSLP/ADAM8/LILRB4/AXL/TNFRSF4/LILRB1/IFNG/AIF1/AP3D1/HAVCR2/SLC39A10/MLH1/SELENOK/NCKAP1L/TNFSF11/NOD2/INPP5D/HLX/CD320/MALT1/LRRK2/CD4/ZBTB16/SIRPG/MDK/SYK/CD6/AKT1/RASAL3/IL18/DOCK8

GOBP_REGULATION_OF_APOPTOTIC_SIGNALING_PATHWAY GOBP_REGULATION_OF_APOPTOTIC_SIGNALING_PATHWAY GOBP_REGULATION_OF_APOPTOTIC_SIGNALING_PATHWAY 320 0.426274824 1.585336006 0.001179245 0.030581767 0.024202852 3434 tags=33%, list=20%, signal=27%

S100A8/VNN1/S100A9/IL1B/AGT/MMP9/CAV1/PEA15/SERPINE1/IFI6/CD44/HYOU1/CD74/PF4/INHBA/PTPRC/CLU/HIF1A/SGMS1/XBP1/BOK/FYN/TNFRSF12A/GNAI2/ICAM1/G0S2/HYAL2/RNF183/SKIL/TNFRSF10A/CREB3L1/SNAI2/IL1A/P4HB/DNAJA1/NME5/ENO1/TNFRSF10B/CTSC/LCK/TMC8/FGFR1/MUC1/DDIAS/EYA3/BID/ITGA6/MIF/GRINA/PDIA3/CFLAR/ACVR1/ITGAV/ITPRIP/TPD52L1/PML/CASP8/CREB3/SLC35F6/STK3/ARHGEF2/SFRP1/PARP1/BCL2L12/PLEKHF1/SRPX/RELA/WNT4/TGFBR1/TRAF1/FIGNL1/SFRP2/INHBB/LRRK2/PTPN1/NOC2L/SOD2/FXN/GPX1/TIMP3/PSME3/FBXW7/CYLD/AKT1/PARK7/TRIAP1/ARRB2/NACC2/PLAUR/CCAR2/YBX3/CTTN/GSTP1/AATF

/PIH1D1/SFPQ/GCLM/TMEM161A/STK4/SELENOS/HERPUD1/BRCA1/NOX1/CX3CL1/USP47/FBH1/NOS3

GOBP_REGULATION_OF_CYTOSOLIC_CALCIUM_ION_CONCENTRATION GOBP_REGULATION_OF_CYTOSOLIC_CALCIUM_ION_CONCENTRATION GOBP_REGULATION_OF_CYTOSOLIC_CALCIUM_ION_CONCENTRATION 328 0.47179308 1.757557489 0.001179245 0.030581767 0.024202852 2142 tags=23%, list=13%, signal=20% CD55/GJA1/CEMIP/CXCL9/CXCL11/CXCL13/CXCL10/PROK2/WNT5A/TGM2/CD38/CXCR2/CAV1/MS4A1/GNA15/F2RL2/FPR1/LYN/CCR2/EDNRA/CCR10/TRPA1/S1PR3/JPH1/APLNR/PTPRC/F2R/CCR1/RAMP3/BOK/PLN/ACKR4/C5AR1/PDGFRA/CCR7/CD19/FYN/JAK2/THY1/CXCR4/CAV2/P2RX5/P2RY8/PLCG2/DIAPH1/FPR3/CORO1A/S1PR1/CLIC2/ERO1A/CCR6/NMU/SELENON/PIK3CG/FFAR4/C3AR1/LCK/CCL19/GSTO1/PKD2/PRNP/TRPV2/IBTK/ITGAV/PTGFR/GPR65/PML/ATP2B4/FPR2/CYSLTR1/CCR5/GPR4/TMEM64/P2RX7/TRPC6

GOBP_RESPONSE_TO_VIRUS GOBP_RESPONSE_TO_VIRUS GOBP_RESPONSE_TO_VIRUS 328 0.473558453 1.764133985 0.001179245 0.030581767 0.024202852 2073 tags=25%, list=12%, signal=23% DUOX2/MMP12/DMBT1/CCL11/CXCL9/POU2AF1/CXCL10/IFITM2/IFIT3/GBP1/MLKL/IFITM3/ISG20/IL33/HYAL1/PIM2/TRIM22/HTRA1/IFI6/IFI16/IFITM1/AIM2/BIRC3/IFI44/PTPRC/OAS2/CLU/SEC14L1/BNIP3/HIF1A/STING1/TLR8/PARP9/DNAJC3/APOBEC1/BST2/DDX21/CCL4/SLFN11/ODC1/CXCR4/STAT1/DDIT4/FGR/ACTA2/NLRC5/HYAL2/SAMHD1/MICB/CD40/RNASE6/FLNA/APOBEC3G/FCN3/HMGA1/CCL22/MST1R/TRIM15/IRF1/ENO1/OAS3/LYST/IFNAR2/IL12RB1/MX2/CCL19/KCNJ8/ABCE1/RTP4/IL23A/PML/IL6/IRF9/LILRB1/IFNG/MX1/BATF3/STAT2/ZBP1/CCT5/SELENOK/SHFL/RELA

GOBP_REGULATION_OF_T_CELL_ACTIVATION GOBP_REGULATION_OF_T_CELL_ACTIVATION GOBP_REGULATION_OF_T_CELL_ACTIVATION 309 0.506922485 1.879085442 0.001182033 0.030581767 0.024202852 2777 tags=36%, list=16%, signal=31% CD55/VNN1/ANXA1/CD274/IDO1/SPINK5/IL1B/CAV1/IL7R/LYN/ADA/SLC7A1/CCR2/LAX1/CD74/JAK3/RAC2/IRF4/HLA-DRA/IL27RA/LGALS1/NFKBIZ/PNP/PTPRC/HLA-DMB/CBFB/RIPK2/XBP1/VCAM1/HSPH1/TNFSF13B/TGFBR2/CCR7/FYN/FCGR2B/SHH/CEBPB/THY1/LILRB2/CD86/SASH3/CD27/CCL2/CD300A/CD81/RHOH/HLA-DPA1/CORO1A/SIRPA/BCL6/IL1A/LEF1/LAPTM5/IGFBP2/VAV1/IRF1/IL6ST/CD276/RIPOR2/LCK/IL12RB1/CCL19/METTL3/DUSP10/FANCD2/TNFRSF1B/RUNX3/TWSG1/EBI3/PRNP/CD2/ITPKB/LAG3/CD47/EGR3/IL23A/IL6/ADAM8/LILRB4/TMEM131L/LILRB1/IFNG/AIF1/GPNMB/AP3D1/HAVCR2/PRDM1/SELENOK/NCKAP1L/TNFSF11/NOD2/HLX/MALT1/SIT1/CD4/ZBTB16/GLMN/SIRPG/MDK/SYK/CD6/LRRC32/AKT1/TNFRSF18/RASAL3/CGAS/IL18/DOCK8/PRKAR1A/DLG5/BTN2A2

GOBP_REGULATION_OF_INFLAMMATORY_RESPONSE GOBP_REGULATION_OF_INFLAMMATORY_RESPONSE GOBP_REGULATION_OF_INFLAMMATORY_RESPONSE 307 0.534244162 1.97894918 0.001183432 0.030581767 0.024202852 2677 tags=34%, list=16%, signal=29% MMP3/S100A8/DUOXA2/C2CD4A/PLA2G2A/S100A9/ANXA1/IDO1/IL1B/S100A12/AGT/WNT5A/TGM2/MMP9/CASP1/IL33/TNFAIP6/C3/LYN/ALOX5/ITGA2/ADA/SERPINE1/CDH5/CCR2/BIRC3/PIK3AP1/PLA2G7/LPL/SLAMF8/NFKBIZ/PTPRC/PTGS2/STING1/SOCS3/ZYX/IL1R1/SBNO2/CCR7/FCGR2B/JAK2/CEBPB/CALCRL/FOXF1/SELE/TLR2/HCK/ABCC1/HYAL2/FFAR2/CCL24/CD81/SERPINF1/CASP4/SIRPA/BCL6/CST7/CASP5/TEK/GPRC5B/IL6ST/WFDC1/CTSC/PIK3CG/FFAR4/GPSM3/SPH

K1/DUSP10/FANCD2/TNFRSF1B/PBK/RORA/BTK/CD200/USP18/CD47/IL23A/IL6/TSLP/ADAM8/PTGIS/IFNG/FPR2/ZBP1/GPR4/RELA/TNFSF11/NOD2/SYT11/PSMA1/DNASE1L3/IL16/LRRK2/F12/NMI/GPX1/MDK/ETS1/CYLD/SIGLEC10/TYRO3/PARK7/SETD6/IL18

GOBP_RESPONSE_TO_MOLECULE_OF_BACTERIAL_ORIGIN GOBP_RESPONSE_TO_MOLECULE_OF_BACTERIAL_ORIGIN GOBP_RESPONSE_TO_MOLECULE_OF_BACTERIAL_ORIGIN 315 0.546071909 2.025939134 0.001183432 0.030581767 0.024202852 1479 tags=27%, list=9%, signal=25% DEFA5/S100A8/DEFA6/CXCL5/TNIP3/CXCL1/DMBT1/CD55/CXCL3/CXCL6/GJA1/CXCL9/S100A9/CXCL11/CXCL13/CXCL8/CD274/IDO1/CXCL10/IL1B/SPARC/WNT5A/CLDN1/CXCL2/CSF2RB/THBD/CASP1/NOS2/LYN/SERPINE1/SELP/LOXL1/ASS1/TGFB1/PDE4B/PF4/LY96/TFPI/SLPI/F2R/S100A7/RIPK2/SGMS1/XBP1/VCAM1/SBNO2/CARD16/KMO/C5AR1/VIM/CCR7/FCGR2B/JAK2/CEBPB/LILRB2/CD86/DCN/SELE/TLR2/HCK/ICAM1/PTAFR/CCL2/PLCG2/IRAK1/IL10RA/CD40/SIRPA/IL1A/LTF/AKIRIN2/MTDH/MRC1/PABPN1/PPBP/ADAM9/KCNJ8/DUSP10/TNFRSF1B/CYP27B1/IRAK2/AKAP12/NLRP7/TLR1

GOBP_LEUKOCYTE_PROLIFERATION GOBP_LEUKOCYTE_PROLIFERATION GOBP_LEUKOCYTE_PROLIFERATION 297 0.539625397 1.99256291 0.001191895 0.030581767 0.024202852 2987 tags=38%, list=18%, signal=32% CD55/GREM1/ANXA1/CD274/IDO1/IL1B/MZB1/MNDA/CTPS1/CD38/MS4A1/IL7R/IL33/MSN/LYN/ADA/SLC7A1/CCR2/CD74/JAK3/RAC2/IL27RA/PNP/PTPRC/HLA-DMB/CLU/GAL/TCIRG1/RIPK2/VCAM1/TNFSF13B/KIT/TGFBR2/CD19/BST2/FYN/FCGR2B/SHH/CEBPB/LILRB2/CD86/SASH3/CD300A/HHEX/TYROBP/CD81/LST1/CD40/HLA-DPA1/CORO1A/BCL6/IL1A/LEF1/IGFBP2/CD79A/IRF1/IL6ST/CD276/PIK3CG/CSF1R/IL12RB1/GPR183/DOCK2/CCL19/FCRL3/TNFRSF1B/TWSG1/EBI3/PRNP/BTN3A1/MIF/CR2/BTK/IKZF3/AHR/IL23A/IL6/LILRB4/TNFRSF4/TMEM131L/LILRB1/CD180/AIF1/GPNMB/HAVCR2/SLC39A10/SELENOK/WNT4/NCKAP1L/TNFSF11/INPP5D/CD320/MALT1/ACE/BLK/CD4/RASGRP1/GLMN/SYK/CD6/LRRC32/RASAL3/IL18/DOCK8/HPRT1/PRKAR1A/DLG5/BTN2A2/TBK1/PSMB10/STAT5B/HLA-DPB1/CTLA4

GOBP_NEGATIVE_REGULATION_OF_PROTEOLYSIS GOBP_NEGATIVE_REGULATION_OF_PROTEOLYSIS GOBP_NEGATIVE_REGULATION_OF_PROTEOLYSIS 303 0.479616978 1.772288608 0.001191895 0.030581767 0.024202852 3025 tags=29%, list=18%, signal=24% SERPINB5/SPINK4/PI3/TIMP1/COL6A3/TFPI2/SERPINA3/SPINK5/AGT/LAMP3/MMP9/SERPINB9/SERPINB3/SERPINB8/SERPINE2/C3/SERPING1/SERPINE1/SERPINA1/IFI6/SERPINB7/CD44/GAS1/IFI16/PI15/TFPI/SLPI/ECM1/TNFAIP8/PAPLN/CARD16/SERPINB4/BST2/SHH/CSTA/PLAT/CD27/USP14/SERPINF1/SERPINB6/CST7/DNAJC1/LTF/SERPINH1/A2M/WFDC1/PTTG1/SPOCK2/SPINK1/COL7A1/PBK/SERPINI1/NLRP7/PRNP/ANXA2/SPOCK1/SDCBP/PML/CD109/SERPINB1/CRYAB/BCL2L12/TIMP2/MAP1A/CHAC1/FURIN/SFRP2/RARRES1/SERPINF2/DNAJB6/OGT/LRRK2/AQP1/GPX1/HSP90AB1/TIMP3/MICAL1/AKT1/PARK7/TRIAP1/ARRB2/TRIM21/ROCK1/PLAUR/CCAR2/PROS1/ITIH5/IDE

GOBP_REGULATION_OF_INNATE_IMMUNE_RESPONSE GOBP_REGULATION_OF_INNATE_IMMUNE_RESPONSE GOBP_REGULATION_OF_INNATE_IMMUNE_RESPONSE 294 0.505190186 1.864394742 0.00120048 0.030581767 0.024202852 3983 tags=48%, list=24%, signal=37% MMP12/WNT5A/MNDA/SERPINB9/CADM1/MUC5B/LYN/SERPING1/LRP8/IFI16/AIM2/BIRC3/SLAMF8/STING1/FCER1G/SOCS3/MUC4/PSME4/PSMB9/TLR8/PARP9/CLEC4A/SERPINB4/FCN1/F

YN/FCGR2B/JAK2/STAT1/FGR/HCK/NLRC5/SAMHD1/FFAR2/PLCG2/TYROBP/LYAR/ICAM2/GBP5/PARP14/AKIRIN2/PSMD14/CLEC4E/A2M/VAV1/IRF1/PSMD12/IFNAR2/PSMC2/MUC1/PSMB2/PSMA3/METTL3/PSMD1/ABCE1/DUSP10/USP18/LAG3/PSMA5/MUC5AC/ADAM8/PSMD6/LILRB1/MUC2/IFNG/PSMB5/PSMC4/FPR2/HAVCR2/ZBP1/SLAMF6/RELA/NOD2/IRF7/PSMB1/PSMA1/CDC37/MALT1/PTPN1/NMI/RASGRP1/HSP90AB1/ADAR/PSME3/SYK/TYRO3/POLR3G/CGAS/CUL1/PSMA7/PSMB8/PSMC5/PVR/IRF3/ARRB2/TRIM5/TRIM21/APOE/JAK1/TBK1/IRAK3/AP1G1/PSMB10/HSP90AA1/STAT5B/PTPN11/PSMB6/HRAS/IL18RAP/PLSCR1/PSMA4/SFPQ/TRAFD1/NLRC4/PSMD13/IFI35/SLC15A4/PSME1/COCH/HMGB1/CARD11/CR1/XRCC5/CD209/PSMC1/DHX58/NECTIN2/PSMB4/HLA-E/MUC17/PSMB3/FADD/PSMD2/IKBKG/PSMC6/LBP/PSMD7/MUC12/CLEC4D/PSMA2/ERAP1

GOBP_TISSUE_MIGRATION GOBP_TISSUE_MIGRATION GOBP_TISSUE_MIGRATION 293 0.442287977 1.63148861 0.001201923 0.030581767 0.024202852 3086 tags=34%, list=18%, signal=29% GREM1/S100P/ANXA1/CXCL13/LOXL2/FSTL1/AGT/SPARC/WNT5A/ADAMTS9/MMP9/RGCC/ROBO1/HYAL1/ITGA2/CTSH/CDH5/PIK3R3/TGFB1/STC1/ANXA3/ACTG2/HIF1A/PTGS2/ENPP2/S100A2/KIT/TGFBR2/SMOC2/ITGB2/PTPRM/DCN/ANLN/MAP4K4/FOXF1/ACTA2/FAP/SRPX2/MET/PLCG2/CD40/SERPINF1/RAB13/MMRN2/VEGFC/KDR/TEK/CALR/CCR6/PIK3CG/ADGRA2/SPRED1/TACSTD2/FGFR1/MCC/ADAM9/PIK3CD/ITGB1BP1/CLEC14A/DUSP10/NRP1/HDAC7/MACF1/LPXN/CORO1C/EGR3/EPHB4/PLXND1/AKT3/FGFBP1/FGF7/ATP2B4/IFNG/VASH1/NUS1/PLK2/TGFBR1/STRAP/SEMA4A/RHOJ/GPX1/FERMT1/GLUL/FBXW7/ETS1/ANGPT2/AKT1/JCAD/EFNB2/APOE/ROCK1/PPM1F/FGF2/EVL/GPI/PTPN11/EMP2/ITGA3/STAT5A/NR2F2/PRCP

GOBP_RIBOSOME_BIOGENESIS GOBP_RIBOSOME_BIOGENESIS GOBP_RIBOSOME_BIOGENESIS 282 0.483999159 1.778667842 0.001207729 0.030581767 0.024202852 4717 tags=51%, list=28%, signal=37% ISG20/NOP2/DDX21/UTP4/GTF3A/GNL2/LYAR/ERI1/RRP36/TSR1/NIFK/RRS1/NOP58/RIOX2/PA2G4/NOP16/RRP7A/DIMT1/BYSL/MRTO4/MRPL36/NAT10/ABCE1/EXOSC3/DDX56/UTP6/RPP40/NOP14/RAN/NOC4L/TRMT112/RRP12/UTP18/NOLC1/MAK16/RPL7L1/DCAF13/IMP4/MTERF3/GTPBP4/MPHOSPH6/UTP14A/EXOSC7/RRP15/RRP9/WDR43/FTSJ3/USP36/DDX18/MRPL20/RIOK1/UTP11/TBL3/MRM2/HEATR1/RRP1B/SDAD1/MRPS2/EXOSC9/RPL26L1/PAK1IP1/NOC2L/WDR55/NHP2/TFB1M/RRP1/DDX10/GLUL/LTV1/SUV39H1/NPM1/BMS1/EXOSC8/BUD23/CHD7/URB1/PWP1/RPUSD4/WDR75/URB2/NOL9/UTP15/UTP3/PELP1/WDR12/HEATR3/DHX30/RRP8/POP7/POP5/AATF/SRFBP1/LSM6/PIH1D1/RPF2/MPHOSPH10/DHX29/BRIX1/NOB1/MYBBP1A/PES1/LAS1L/NOL10/EIF4A3/XRCC5/MPV17L2/WDR46/WDR3/TEX10/METTL5/EMG1/EXOSC10/WDR36/DDX27/DDX51/LSG1/UTP25/NOP9/SART1/SENP3/NOP10/RBIS/DDX17/NOL6/THUMPD1/SNU13/CUL4A/EXOSC1/NOL11/NPM3/NUP88/MTREX/MRM3/GEMIN4/NOL8/MALSU1/NSUN5/NLE1/EXOSC2/PDCD11/FBLL1/GTF2H5/PRKDC/XPO1

GOBP_CELL_CHEMOTAXIS GOBP_CELL_CHEMOTAXIS GOBP_CELL_CHEMOTAXIS 273 0.569063405 2.087458649 0.001210654 0.030581767 0.024202852 2346 tags=38%, list=14%, signal=34% S100A8/CXCL5/CXCL1/CXCL3/GREM1/CXCL6/CCL11/CXCL9/S100A9/ANXA1/CXCL11/CXCL13/CXCL8/CCL18/CXCL10/S100A12/WNT5A/CXCL2/CXCR2/LYN/ALOX5/SERPINE1/CCR2/CD74/CCR10/PDE4B/PF4/PLA2G7/RAC2/SLAMF8/CCL20/S100A7/CCR1/FCER1G/ADGRE2/VCAM1/ACKR4/C5AR1/KIT/PDGFRA/CCR7/SMOC2/CSF3R/ITGB2/CCL4/CXCR4/LOX/CCL2/PDGFRB/ABCC1/MET/FFAR2/CCL24/DOCK4/RAB13/CORO1A/VEGFC/LEF1/KDR/CKLF/CCL22/S1PR1/CH25H/TPBG/CALR/VAV1

/CCR6/ANO6/PIK3CG/C3AR1/RIPOR2/CSF1R/LYST/GPR183/FGFR1/PPBP/GPSM3/CCL19/PIK3CD/NRP1/JAM3/MIF/PPIB/CXCL14/EGR3/IL23A/SRP54/IL6/ADAM8/CREB3/RHOG/NUP85/AIF1/FPR2/CCR5/HSD3B7/NCKAP1L/TNFSF11/NOD2/CXCL16/PIP5K1C/SWAP70/PARVA/IL16/CMKLR1
GOBP_RESPONSE_TO_TUMOR_NECROSIS_FACTOR
GOBP_RESPONSE_TO_TUMOR_NECROSIS_FACTOR
GOBP_RESPONSE_TO_TUMOR_NECROSIS_FACTOR 285 0.437378726 1.607057207 0.001212121 0.030581767 0.024202852 2703 tags=31%, list=16%, signal=26%
CHI3L1/CCL11/CXCL8/CCL18/CLDN1/COL1A1/GBP1/TNFRSF17/CASP1/PLVAP/HYAL1/ASS1/AIM2/BIRC3/TNFRSF11B/CCL20/SGMS1/TNFSF13/PSME4/VCAM1/PSMB9/CARD16/TNFSF13B/JAK2/CCL4/TNFRSF9/STAT1/PIAS3/MAP3K5/TNFRSF12A/POSTN/SELE/ICAM1/CD27/CCL2/HYAL2/CCL24/CD40/CASP4/KLF2/LAPTM5/CCL22/PSMD14/TNFSF15/CLDN18/PSMD12/GBP2/PSMC2/TRAF5/CCL19/PSMB2/ADAM9/SPHK1/PSMA3/PSMD1/TNFRSF1B/AKAP12/RORA/PSMA5/CCDC3/CASP8/TNFRSF4/ARHGEF2/SFRP1/PSMD6/PSMB5/PSMC4/RELA/LTB/TNFSF11/TCL1A/TRAF1/CXCL16/PSMB1/NPNT/PSMA1/CLIP3/CCL7/PSME3/SYK/TRAF3/CYLD/AKT1/TNFRSF18/TANK/PSMA7/PSMB8/PSMC5
GOBP_HUMORAL_IMMUNE_RESPONSE GOBP_HUMORAL_IMMUNE_RESPONSE
GOBP_HUMORAL_IMMUNE_RESPONSE 248 0.70689458 2.572484991 0.001213592 0.030581767 0.024202852 1263 tags=27%, list=7%, signal=25%
REG1A/REG1B/REG3A/DEFA5/S100A8/LCN2/PI3/DEFA6/CXCL5/CXCL1/DMBT1/CD55/C4BPB/CXCL3/CXCL6/C4BPA/PLA2G2A/CXCL9/S100A9/POU2AF1/CXCL11/CXCL13/CFB/CXCL8/CXCL10/SPINK5/IL1B/CFI/S100A12/CXCL2/RGCC/SPNS2/MS4A1/C1S/C3/ALOX5/SERPING1/TREM1/C2/C1R/PF4/HLA-DQB1/SLPI/PTPRC/CLU/S100A7/C5AR1/FCN1/CCR7/CD19/FCGR2B/C1QB/CCL2/CD81/RNASE6/FCN3/LTF/C1QA/A2M/SEMG1/KRT6A/CCR6/LYZ/C3AR1/GPR183/PPBP
GOBP_REGULATION_OF_LEUKOCYTE_DIFFERENTIATION
GOBP_REGULATION_OF_LEUKOCYTE_DIFFERENTIATION
GOBP_REGULATION_OF_LEUKOCYTE_DIFFERENTIATION 266 0.481222551 1.759819245 0.001213592 0.030581767 0.024202852 2549 tags=33%, list=15%, signal=29%
VNN1/ANXA1/SPINK5/IL7R/LYN/ADA/CCR2/FBN1/HCLS1/CD74/TGFB1/PF4/JAK3/EVI2B/IRF4/HLA-DRA/INHBA/SLAMF8/NFKBIZ/NME1/PNP/PTPRC/CBFB/RIPK2/CCR1/XBP1/TGFBR2/FCGR2B/GPR137B/SHH/CEBPB/LILRB2/CD86/PIAS3/SASH3/CD27/MAFB/TYROBP/RHOH/BCL6/RASSF2/LEF1/LTF/IRF1/CLDN18/IL12RB1/CCL19/TESC/METTL3/DUSP10/FANCD2/FCRL3/RUNX3/CD2/BTK/SLC9B2/IKZF3/ITPKB/TFE3/LAG3/LIF/EGR3/IL23A/CASP8/ADAM8/LILRB4/AXL/TMEM131L/SFRP1/LILRB1/IFNG/LILRB3/CREB1/AP3D1/PRDM1/TMEM64/NCKAP1L/TNFSF11/INPP5D/HLX/IRF7/MALT1/CD4/HAX1/ZBTB16/FSTL3/MDK/SYK/FBXW7
GOBP_RESPONSE_TO_ENDOPLASMIC_RETICULUM_STRESS
GOBP_RESPONSE_TO_ENDOPLASMIC_RETICULUM_STRESS
GOBP_RESPONSE_TO_ENDOPLASMIC_RETICULUM_STRESS281 0.442884134 1.624791162 0.001213592 0.030581767 0.024202852 3469 tags=38%, list=20%, signal=31%
CXCL8/KDELR3/DERL3/CREB3L2/SDF2L1/MANF/CAV1/ALOX5/SRPRB/DNAJB11/HYOU1/FBXO6/NIBAN1/MYDGF/DNAJB9/CLU/XBP1/BOK/PDIA4/DNAJC10/DNAJC3/FICD/HSPA5/FCGR2B/CEBPB/PREB/MAP3K5/HM13/CCL2/PDIA6/RNF183/SRPRA/USP14/ASNS/CASP4/CREB3L1/ERLEC1/EIF2S1/P4HB/VCP/CALR/UBE2J1/RASGRF2/ERO1A/ATP2A3/TNFRSF10B/SSR1/FKBP14/SEC61B/AGR2/CCND1/HSP90B1/WFS1/GRINA/PDIA3/UGGT2/SEC16A/PML/CREB3/TBL2/SGTB/TLN1/ERN1/ERP44/ERP27/YIF1A/ATP2A2/GORASP2/SRPX/SELENOK/EIF4G1/DERL2/SERP1/CREB3L4/EDEM1/CHA

C1/BFAR/LRRK2/CANX/PTPN1/SEC31A/UGGT1/DERL1/CDK5RAP3/ATF4/PARK7/UBE2J2/ARFGAP1/EXTL2/UBXN8/TARDBP/PPP2R5B/USP13/EIF2B5/SEL1L/LMNA/UFD1/SELENOS/SHC1/HERPUD1/EDEM2/UFM1/MBTPS1/UBE2G2/BAK1/ITPR1/FAF2

GOBP_POSITIVE_REGULATION_OF_CELL_CELL_ADHESION GOBP_POSITIVE_REGULATION_OF_CELL_CELL_ADHESION GOBP_POSITIVE_REGULATION_OF_CELL_CELL_ADHESION 263 0.53827007 1.963975917 0.001215067 0.030581767 0.024202852 2994 tags=43%, list=18%, signal=36% CD55/VNN1/ANXA1/CXCL13/CD274/IL1B/WNT5A/CAV1/IL7R/LYN/ALOX5/ADA/SLC7A1/SELP/CCR2/CD44/CD74/JAK3/PODXL/HLA-DRA/IL27RA/LGALS1/NFKBIZ/PDPN/PNP/PTPRC/HLA-DMB/CBFB/RIPK2/XBP1/ST3GAL4/VCAM1/HSPH1/TNFSF13B/TGFBR2/CCR7/FYN/ITGB2/SHH/THY1/LILRB2/CD86/ADAM19/SASH3/SELE/ICAM1/CD27/PTAFR/CCL2/PIEZO1/IRAK1/CD81/RHOH/HLA-DPA1/CORO1A/SIRPA/BCL6/IL1A/LEF1/IGFBP2/VAV1/IL6ST/CD276/LCK/IL12RB1/CCL19/DUSP10/RUNX3/EBI3/ITPKB/CD47/EGR3/IL23A/IL6/ADAM8/LILRB4/FUT4/LILRB1/IFNG/AIF1/AP3D1/HAVCR2/SELENOK/RELA/NCKAP1L/TNFSF11/NOD2/HLX/SERPINF2/MALT1/CD4/CEACAM6/ZBTB16/FSTL3/SIRPG/FOXA2/MDK/SYK/ETS1/CD6/AKT1/RASAL3/ITGA4/IL18/DOCK8/JAK1/NR4A3/PLAUR/STAT5B/HLA-DPB1/CTLA4/PTPN11

GOBP_PHAGOCYTOSIS GOBP_PHAGOCYTOSIS GOBP_PHAGOCYTOSIS 254 0.47673577 1.737316331 0.001216545 0.030581767 0.024202852 2523 tags=30%, list=15%, signal=26% C4BPB/C4BPA/ANXA1/IL1B/PECAM1/CD93/TGM2/NCF2/RAB31/FCGR2A/C3/LYN/ITGA2/C2/RAC2/ANXA3/PTPRC/FCER1G/CLEC7A/ARPC1B/FCN1/FYN/ITGB2/FCGR2B/FGR/TLR2/HCK/CCL2/CD300A/MET/PLCG2/TYROBP/RHOH/LYAR/CORO1A/SIRPA/FCN3/ITGAM/WIPF1/MST1R/CALR/VAV1/ANO6/LYST/DOCK2/SPHK1/IL15RA/MFGE8/ARHGAP25/CORO1C/CD47/ITGAV/ELMO1/CD300LF/RHOG/AXL/PEAR1/NCF4/IFNG/AIF1/FPR2/JMJD6/SRPX/NCKAP1L/RUBCN/MYO7A/NOD2/CDC42SE1/SYT11/PIP5K1C/DYSF/MESD/SIRPG/HSP90AB1/SYK

GOBP_NEGATIVE_REGULATION_OF_CELL_ADHESION GOBP_NEGATIVE_REGULATION_OF_CELL_ADHESION GOBP_NEGATIVE_REGULATION_OF_CELL_ADHESION 264 0.448895255 1.63799815 0.001218027 0.030581767 0.024202852 2846 tags=33%, list=17%, signal=28% IL1RN/MMP12/TNC/ANXA1/CD274/IDO1/TGFBI/COL1A1/RGCC/GBP1/SERPINE2/SERPINE1/ASS1/LAX1/CD74/TGFB1/JAK3/PLXNA1/PODXL/LGALS1/PTPRC/CBFB/FXYD5/MAD2L2/FCGR2B/JAK2/SHH/DLC1/CEBPB/LILRB2/CD86/MAP4K4/POSTN/ARHGDIB/CD300A/SEMA4D/PLXNC1/BCL6/SNAI2/LAPTM5/IRF1/RIPOR2/TACSTD2/MUC1/ITGB1BP1/METTL3/RUNX3/TWSG1/PRNP/JAM3/LAG3/LPXN/CORO1C/DUSP22/SPOCK1/ADAM15/PLXND1/GTPBP4/ARHGDIA/RCC2/LILRB4/TMEM131L/LILRB1/GPNMB/JAG1/APOD/RDX/HAVCR2/PHLDB2/FZD4/HLX/PLXNA3/SWAP70/GLMN/MDK/ANGPT2/LRRC32/BMP6/AKT1/FAM107A/PRKAR1A/C1QTNF1/DLG5/BTN2A2/FBLN1/PPM1F

GOBP_PROTEIN_LOCALIZATION_TO_NUCLEUS GOBP_PROTEIN_LOCALIZATION_TO_NUCLEUS GOBP_PROTEIN_LOCALIZATION_TO_NUCLEUS 261 0.462986966 1.687958988 0.001218027 0.030581767 0.024202852 4498 tags=49%, list=27%, signal=37% MMP12/TRIM29/COL1A1/FERMT2/HCLS1/TGFB1/BAG3/PARP9/TRIM40/RASSF5/FYN/JAK2/SHH/IPO4/LATS2/KPNA2/HHEX/SEC13/HYAL2/OSBPL8/FLNA/STAT3/RRS1/RAB23/CALR/WWTR1/TNPO1/CLDN18/GBP2/CDK1/TBRG1/ECT2/BYSL/NUP62/TNPO2/BARD1/ATP13A2/PLK1/RAN/NOLC1/E2F3/NUP98/IPO9/RANGAP1/LIF/HIKESHI/PML/CCT3/ZPR1/LILRB4/PARP1/PKIA/NUP85/CCT8/INTS13/IFNG/NXT1/KPNB1/APOD/CCT5/SYNE1/CCT2/PPP3CA/CSE1L/NUP93/CCT6A/LRRK2/CCT

4/CCT7/PAF1/PINX1/KPNA6/FERMT1/ZBTB16/HSP90AB1/TRIM28/GLUL/SYK/CDK5RAP3/GLIS2/AKT1/PARK7/DCLK1/FBXO4/DVL1/TARDBP/NUP62CL/TRIM8/HEATR3/NUP58/LMNA/RPF2/NF2/ELAVL1/PRICKLE1/UFM1/MBTPS1/IPO7/NUP50/PKIG/ILRUN/MDM2/DTX3L/SOX9/CTDNEP1/LZTS2/NUP35/KPNA1/NUP155/NUP107/IPO13/POM121L12/RANBP2/CNEP1R1/TESK1/NUP188/NUP88/MDFIC/F2/TOR1AIP1/NUP54/GTSE1/SIX4/KPNA3/LAMTOR5/NOL8/HNRNPU/GCKR/CARD10

GOBP_REGULATION_OF_VASCULATURE_DEVELOPMENT

GOBP_REGULATION_OF_VASCULATURE_DEVELOPMENT

GOBP_REGULATION_OF_VASCULATURE_DEVELOPMENT 264 0.522685597 1.907255716 0.001218027 0.030581767 0.024202852 2858 tags=38%, list=17%, signal=32%

CHI3L1/GREM1/CCL11/CXCL13/CXCL8/WARS1/CXCL10/SPINK5/PROK2/IL1B/SPARC/WNT5A/ADAMTS9/ADAMTS1/RGCC/HYAL1/C3/ALOX5/CTSH/ENG/F3/SERPINE1/PKM/CDH5/CCR2/COL4A2/PF4/SULF1/MYDGF/ANXA3/APLNR/ECM1/HIF1A/ECSCR/XBP1/ITGA5/ENPP2/TWIST1/C5AR1/AGO2/TGFBR2/SMOC2/ITGB2/CXCR4/PTPRM/DCN/STAT1/TIE1/HHEX/CCL24/CD40/SERPINF1/CREB3L1/VEGFC/IL1A/KLF2/KDR/EMILIN1/TEK/MTDH/ADGRA2/C3AR1/SPRED1/LRG1/SPHK1/PRKCB/PIK3CD/HK2/PPP1R16B/PLXND1/AKT3/PML/IL6/ITGAX/PTGIS/SFRP1/THBS2/ATP2B4/GPNMB/VASH1/GPR4/PLK2/WNT4/SFRP2/SEMA4A/RAMP2/AQP1/RHOJ/MDK/GLUL/ETS1/ANGPTL4/ANGPT2/DLL1/JCAD/CYBB/NIBAN2/ROCK1/FGF2/JAK1

GOBP_B_CELL_ACTIVATION GOBP_B_CELL_ACTIVATION GOBP_B_CELL_ACTIVATION 244 0.540547084 1.960120582 0.001222494 0.030581767 0.024202852 2645 tags=37%, list=16%, signal=32%

POU2AF1/MZB1/MNDA/CTPS1/CD38/MS4A1/IL7R/LYN/ADA/LAX1/CD74/TGFB1/JAK3/INHBA/IL27RA/LGALS1/SLAMF8/PTPRC/DNAJB9/TCIRG1/XBP1/TNFSF13/VCAM1/TNFSF13B/KIT/CD19/SAMSN1/BST2/MAD2L2/FCGR2B/CD86/SASH3/CD27/CD300A/HHEX/PLCG2/TYROBP/CD81/CD40/CMTM7/BCL6/LEF1/LAPTM5/MFNG/CD79B/CD79A/BANK1/CCR6/EZH2/DOCK11/GPR183/PRKCB/PIK3CD/TCF3/FCRL3/EXOSC3/MIF/CR2/NKX2-3/BTK/IKZF3/AHR/EXO1/TXLNA/CASP8/IL6/MSH6/ITM2A/TNFRSF4/SFRP1/CD180/SLC39A10/MLH1/BATF/NCKAP1L/NBN/NOD2/INPP5D/CD320/AKAP17A/SWAP70/MALT1/BLK/RASGRP1/LAT2/SYK/LRRC8A/THOC1/DLL1/ITGA4

GOBP_ADAPTIVE_IMMUNE_RESPONSE_BASED_ON_SOMATIC_RECOMBINATION_OF_IMMUNE_RECEPTORS_BUILT_FROM_IMMUNOGLOBULIN_SUPERFAMILY_DOMAINS

GOBP_ADAPTIVE_IMMUNE_RESPONSE_BASED_ON_SOMATIC_RECOMBINATION_OF_IMMUNE_RECEPTORS_BUILT_FROM_IMMUNOGLOBULIN_SUPERFAMILY_DOMAINS

GOBP_ADAPTIVE_IMMUNE_RESPONSE_BASED_ON_SOMATIC_RECOMBINATION_OF_IMMUNE_RECEPTORS_BUILT_FROM_IMMUNOGLOBULIN_SUPERFAMILY_DOMAINS 243 0.609586339 2.208216649 0.00122549 0.030581767 0.024202852 2400 tags=37%, list=14%, signal=32%

CD55/C4BPB/C4BPA/IL13RA2/ANXA1/CXCL13/CD274/IL1B/CFI/IL7R/C1S/IL33/C3/CTSH/ADA/SERPING1/C2/CCR2/RFTN1/C1R/CD74/TGFB1/KLHL6/HLA-DQB1/AZGP1/JAK3/IRF4/HLA-DRA/IL27RA/NFKBIZ/ENTPD7/PTPRC/CLU/TCIRG1/RIPK2/FCER1G/TNFSF13/IL1R1/TLR8/TNFSF13B/CD19/MAD2L2/FCGR2B/C1QB/SASH3/ICAM1/CD27/CD81/CD40/BCL6/LEF1/C1QA/STAT3/CCR6/CTSC/IL12RB1/CCL19/IL18R1/TNFRSF1B/IL18BP/EXOSC3/EBI3/CR2/RORA/BTK/IL23A/EXO1/IL6/MSH6/MR1/LILRB4/C1RL/LILRB1/JAG1/HAVCR2/MLH1/BATF/SLAMF6/BTN3A3/NBN/NOD2/INPP5D/NFKB2/HLX/SEMA4A/IRF7/SWAP70/MALT1/CD4/LY9

GOBP_POSITIVE_REGULATION_OF_RESPONSE_TO_BIOTIC_STIMULUS

GOBP_POSITIVE_REGULATION_OF_RESPONSE_TO_BIOTIC_STIMULUS

GOBP_POSITIVE_REGULATION_OF_RESPONSE_TO_BIOTIC_STIMULUS 237 0.515091857 1.861584204 0.00122549 0.030581767 0.024202852 3961 tags=47%, list=23%, signal=37%

MMP12/CD274/WNT5A/MNDA/CADM1/MUC5B/LYN/IFI16/AIM2/LY96/STING1/FCER1G/MUC4/PSME4/PSMB9/TLR8/PARP9/CLEC4A/FCN1/FYN/HCK/NLRC5/FFAR2/PLCG2/TYROBP/ICAM2/GBP5/AKIRIN2/TRIM15/PSMD14/CLEC4E/VAV1/PSMD12/PSMC2/MUC1/PSMB2/PSMA3/PSMD1/LAG3/PSMA5/MUC5AC/MR1/ADAM8/PSMD6/CD180/MUC2/PSMB5/PSMC4/FPR2/HAVCR2/ZBP1/SLAMF6/RELA/NOD2/IRF7/PSMB1/PSMA1/LY86/MALT1/NMI/RASGRP1/PSME3/SYK/BMP6/POLR3G/CGAS/CUL1/PSMA7/PSMB8/PSMC5/PVR/IRF3/TRIM5/TBK1/AP1G1/PSMB10/HSP90AA1/STAT5B/PSMB6/HRAS/IL18RAP/HSPD1/PLSCR1/PSMA4/SFPQ/NLRC4/PSMD13/IFI35/SLC15A4/PSME1/COCH/HMGB1/CARD11/XRCC5/CD209/PSMC1/DHX58/NECTIN2/CYBA/PSMB4/HLA-E/MUC17/PSMB3/FADD/PSMD2/IKBKG/PSMC6/LBP/PSMD7/MUC12/CLEC4D/PSMA2

GOBP_ANTIGEN_RECEPTOR_MEDIATED_SIGNALING_PATHWAY

GOBP_ANTIGEN_RECEPTOR_MEDIATED_SIGNALING_PATHWAY

GOBP_ANTIGEN_RECEPTOR_MEDIATED_SIGNALING_PATHWAY 229 0.509939898 1.833310908 0.001234568 0.030581767 0.024202852 3993 tags=50%, list=24%, signal=39%

MNDA/CD38/GBP1/MS4A1/LYN/ADA/RFTN1/LAX1/LCP2/KLHL6/PDE4B/HLA-DQB1/HLA-DRA/NFKBIZ/PTPRC/CBFB/THEMIS2/RIPK2/PSME4/PSMB9/KCNN4/CCR7/CD19/FYN/FCGR2B/THY1/CD300A/PLCG2/CD81/HLA-DQA1/HLA-DPA1/LAPTM5/FYB1/PSMD14/DGKZ/RAB29/CD79B/CD79A/CD276/PSMD12/LCK/PSMC2/PSMB2/PRKCB/PSMA3/PIK3CD/PSMD1/FCRL3/PRNP/SH2B2/BTN3A1/BTK/PRKCH/ITK/LPXN/PSMA5/EIF2B3/LILRB4/PSMD6/PSMB5/PSMC4/SLC39A10/BTN3A3/RELA/NCKAP1L/INPP5D/EIF2B2/PSMB1/SPG21/PSMA1/MALT1/BLK/CD4/LAT2/PSME3/CD3D/SYK/CUL1/PSMA7/PSMB8/PSMC5/WAS/LIME1/UBE2N/BTN2A2/PSMB10/EIF2B5/HLA-DPB1/CTLA4/PSMB6/EIF2B4/HRAS/PSMA4/RC3H2/LAT/PSMD13/PSME1/CD247/CARD11/BTN2A1/PSMC1/NFATC2/PRKD2/DUSP3/NECTIN2/PSMB4/PSMB3/PSMD2/IKBKG/STAP1/PSMC6/PSMD7/PSMA2/CSK/ZAP70

GOBP_REGULATION_OF_LEUKOCYTE_PROLIFERATION

GOBP_REGULATION_OF_LEUKOCYTE_PROLIFERATION

GOBP_REGULATION_OF_LEUKOCYTE_PROLIFERATION 229 0.537300817 1.931677539

0.001234568 0.030581767 0.024202852 2777 tags=37%, list=16%, signal=31%

CD55/GREM1/ANXA1/CD274/IDO1/IL1B/MZB1/MNDA/CD38/IL33/LYN/ADA/SLC7A1/CCR2/CD74/JAK3/RAC2/IL27RA/PNP/PTPRC/HLA-DMB/GAL/RIPK2/VCAM1/TNFSF13B/TGFBR2/BST2/FCGR2B/SHH/CEBPB/LILRB2/CD86/SASH3/CD300A/HHEX/TYROBP/CD81/LST1/CD40/HLA-DPA1/CORO1A/BCL6/IL1A/IGFBP2/IRF1/IL6ST/CD276/CSF1R/IL12RB1/GPR183/CCL19/FCRL3/TNFRSF1B/TWSG1/EBI3/PRNP/MIF/BTK/IKZF3/AHR/IL23A/IL6/LILRB4/TNFRSF4/TMEM131L/LILRB1/AIF1/GPNMB/HAVCR2/SLC39A10/SELENOK/NCKAP1L/INPP5D/CD320/BLK/CD4/GLMN/SYK/CD6/LRRC32/RASAL3/IL18/PRKAR1A/DLG5/BTN2A2

GOBP_LYMPHOCYTE_MEDIATED_IMMUNITY GOBP_LYMPHOCYTE_MEDIATED_IMMUNITY

GOBP_LYMPHOCYTE_MEDIATED_IMMUNITY 232 0.589504384 2.123225078

0.001236094 0.030581767 0.024202852 2319 tags=34%, list=14%, signal=29%

CD55/C4BPB/C4BPA/IL13RA2/IL1B/CFI/SERPINB9/CADM1/IL7R/C1S/SLAMF7/C3/CTSH/SERPING1/C2/CCR2/RFTN1/C1R/CD74/TGFB1/HLA-DQB1/AZGP1/HLA-DRA/GZMB/IL27RA/PTPRC/CLU/TCIRG1/FCER1G/TNFSF13/IL1R1/TLR8/SERPINB4/CD19/MAD2L2/FCGR2B/C1QB/SASH3/ICAM1/

CD27/CD81/CD40/CORO1A/BCL6/C1QA/VAV1/CCR6/CTSC/LYST/IL12RB1/IL18R1/TUBB/TNFRSF1B/EXOSC3/CR2/BTK/LAG3/IL23A/EXO1/IL6/MSH6/MR1/LILRB4/C1RL/LILRB1/JAG1/HAVCR2/MLH1/BATF/SLAMF6/BTN3A3/NBN/NOD2/INPP5D/RNF19B/IRF7/SWAP70/MALT1
GOBP_REGULATION_OF_METAL_ION_TRANSPORT
GOBP_REGULATION_OF_METAL_ION_TRANSPORT
GOBP_REGULATION_OF_METAL_ION_TRANSPORT 235 0.476728575 1.717969969 0.001236094 0.030581767 0.024202852 1531 tags=22%, list=9%, signal=20%
GJA1/CEMIP/CXCL9/CXCL11/CXCL10/CAV1/KCNE3/LYN/PDE4B/GEM/STC1/JPH1/APLNR/F2R/CCR1/RAMP3/HOMER1/PLN/CD19/FYN/CCL4/THY1/LILRB2/CXCR4/SESTD1/TRIM27/P2RX5/GNAI2/ICAM1/CCL2/PDGFRB/PLCG2/DIAPH1/CORO1A/GRAMD2A/GNB5/SEMG1/CLIC2/SELENON/ANO6/PIK3CG/CD84/RGS4/GSTO1/SPINK1/FCRL3/PKD2/PRNP/TRPV2/WFS1/ORAI1
GOBP_T_CELL_DIFFERENTIATION GOBP_T_CELL_DIFFERENTIATION
GOBP_T_CELL_DIFFERENTIATION 235 0.479726366 1.728773004 0.001236094 0.030581767 0.024202852 2751 tags=35%, list=16%, signal=30%
VNN1/ANXA1/SPINK5/IL1B/IL7R/ADA/CCR2/CD74/JAK3/IRF4/HLA-DRA/NFKBIZ/PNP/ENTPD7/PTPRC/CBFB/TCIRG1/RIPK2/FCER1G/XBP1/KIT/TGFBR2/CCR7/SHH/LILRB2/CD86/SASH3/CD27/MAFB/RHOH/BCL6/IL1A/LEF1/STAT3/VAV1/IRF1/CCR6/LCK/IL12RB1/GPR183/DOCK2/CCL19/PIK3CD/IL18R1/METTL3/DUSP10/FANCD2/RUNX3/CD2/RORA/NKX2-3/ITPKB/ITK/LAG3/EGR3/IL23A/IL6/MR1/ADAM8/CTSL/LILRB4/TMEM131L/IFNG/AP3D1/BATF/PRDM1/JMJD6/SLAMF6/WNT4/NCKAP1L/HLX/SEMA4A/MALT1/CD4/LY9/ZBTB16/MDK/CD3D/SYK/TNFRSF18/IL18/ATP7A/CHD7
GOBP_DEFENSE_RESPONSE_TO_BACTERIUM GOBP_DEFENSE_RESPONSE_TO_BACTERIUM
GOBP_DEFENSE_RESPONSE_TO_BACTERIUM 224 0.513643468 1.845581744 0.001237624 0.030581767 0.024202852 1976 tags=20%, list=12%, signal=18%
DEFA5/S100A8/LCN2/PI3/DEFA6/DMBT1/CXCL6/PLA2G2A/S100A9/CXCL13/SPINK5/S100A12/IL7R/NOS2/SERPINE1/SELP/SLPI/ANXA3/IL27RA/SLAMF8/CCL20/S100A7/RIPK2/FCER1G/C5AR1/CEBPB/FGR/TLR2/RNASE6/LTF/SEMG1/KRT6A/LYZ/GBP2/LYST/PPBP/ADAMTS5/IL23A/IL6/MR1/CFP/FPR2/CTSG/ANG/HAVCR2
GOBP_CELL_MATRIX_ADHESION GOBP_CELL_MATRIX_ADHESION
GOBP_CELL_MATRIX_ADHESION 212 0.501744022 1.794668507 0.001243781 0.030581767 0.024202852 3487 tags=38%, list=21%, signal=31%
MMP12/GREM1/PLAU/ADAMTS9/NID1/FERMT2/COL3A1/ITGA2/SERPINE1/CD44/MUC4/ZYX/FN1/VCAM1/MADCAM1/EFEMP2/CCR7/ITGB2/DLC1/THY1/MAP4K4/POSTN/ACTN1/BCR/BCL6/TRIP6/KDR/COL16A1/ITGA8/EMILIN1/TEK/FBLN5/LIMCH1/SGCE/ADAM9/ITGB1BP1/LYPD5/NRP1/JAM3/MACF1/HPSE/CORO1C/ITGAV/NID2/DUSP22/ADAM15/VCL/PLEKHA2/AJUBA/RCC2/SFRP1/SNED1/JAG1/APOD/LYVE1/PHLDB2/ITGB4/WNT4/NPNT/PARVG/CEACAM6/FERMT1/COL5A3/FAM107A/ITGA4/RHOD/ROCK1/PPM1F/ITGA7/CD34/CTTN/EMP2/ITGA3/CDH13/GPM6B/LDB1/NF2/FERMT3/CX3CL1/EPHA1/ITGA11
GOBP_POSITIVE_REGULATION_OF_LEUKOCYTE_CELL_CELL_ADHESION
GOBP_POSITIVE_REGULATION_OF_LEUKOCYTE_CELL_CELL_ADHESION
GOBP_POSITIVE_REGULATION_OF_LEUKOCYTE_CELL_CELL_ADHESION 223 0.577490068 2.072454058 0.001243781 0.030581767 0.024202852 2688 tags=43%, list=16%, signal=36%
CD55/VNN1/ANXA1/CD274/IL1B/CAV1/IL7R/LYN/ALOX5/ADA/SLC7A1/SELP/CCR2/CD44/CD74/JAK3/HLA-DRA/IL27RA/LGALS1/NFKBIZ/PNP/PTPRC/HLA-DMB/CBFB/RIPK2/XBP1/ST3GAL4/VC

AM1/HSPH1/TNFSF13B/TGFBR2/CCR7/FYN/ITGB2/SHH/THY1/LILRB2/CD86/SASH3/SELE/ICAM1/CD27/PTAFR/CCL2/IRAK1/CD81/RHOH/HLA-DPA1/CORO1A/SIRPA/BCL6/IL1A/LEF1/IGFBP2/VAV1/IL6ST/CD276/LCK/IL12RB1/CCL19/DUSP10/RUNX3/EBI3/ITPKB/CD47/EGR3/IL23A/IL6/ADAM8/LILRB4/FUT4/LILRB1/IFNG/AIF1/AP3D1/HAVCR2/SELENOK/RELA/NCKAP1L/TNFSF11/NOD2/HLX/MALT1/CD4/ZBTB16/SIRPG/MDK/SYK/ETS1/CD6/AKT1/RASAL3/ITGA4/IL18/DOCK8

GOBP_NEGATIVE_REGULATION_OF_PEPTIDASE_ACTIVITY GOBP_NEGATIVE_REGULATION_OF_PEPTIDASE_ACTIVITY GOBP_NEGATIVE_REGULATION_OF_PEPTIDASE_ACTIVITY 225 0.559618577 2.007987126 0.00124533 0.030581767 0.024202852 2630 tags=30%, list=16%, signal=25% SERPINB5/SPINK4/PI3/TIMP1/COL6A3/TFPI2/SERPINA3/SPINK5/AGT/LAMP3/MMP9/SERPINB9/SERPINB3/SERPINB8/SERPINE2/C3/SERPING1/SERPINE1/SERPINA1/IFI6/SERPINB7/CD44/IFI16/PI15/TFPI/SLPI/ECM1/TNFAIP8/PAPLN/CARD16/SERPINB4/BST2/CSTA/CD27/USP14/SERPINF1/SERPINB6/CST7/LTF/SERPINH1/A2M/WFDC1/PTTG1/SPOCK2/SPINK1/COL7A1/SERPINI1/NLRP7/PRNP/ANXA2/SPOCK1/CD109/SERPINB1/CRYAB/BCL2L12/TIMP2/FURIN/SFRP2/RARRES1/SERPINF2/DNAJB6/AQP1/GPX1/TIMP3/MICAL1/AKT1/PARK7

GOBP_ANTIGEN_PROCESSING_AND_PRESENTATION GOBP_ANTIGEN_PROCESSING_AND_PRESENTATION GOBP_ANTIGEN_PROCESSING_AND_PRESENTATION 217 0.527215756 1.889219311 0.00125 0.030581767 0.024202852 3983 tags=50%, list=24%, signal=39% NCF2/HLA-DMA/CTSH/RFTN1/CD74/SEC24D/HLA-DQB1/AZGP1/HLA-DRA/FCGR1B/HLA-DMB/FCER1G/PSME4/PSMB9/TAP2/CLEC4A/CCR7/FCGR2B/LILRB2/ICAM1/TAP1/SEC13/RAB8B/HLA-DQA1/HLA-DPA1/PSMD14/HLA-DOB/CALR/AP1S3/PSMD12/PSMC2/KLC2/CCL19/PSMB2/CENPE/PSMA3/AP1S2/PSMD1/SEC24C/CTSE/KIF23/PDIA3/LAG3/ITGAV/PSMA5/SEC24A/MR1/CTSL/NCF4/PSMD6/PSMB5/PSMC4/AP1M1/AP3D1/KIF2C/KIF11/NOD2/SEC22B/PSMB1/KIF4A/PSMA1/ACE/CANX/SEC31A/PSME3/TAPBP/PSMA7/YTHDF1/PSMB8/PSMC5/WAS/CYBB/MARCHF1/AP1G1/PSMB10/HLA-DPB1/PSMB6/IDE/PSMA4/RACGAP1/DCTN6/WDFY4/AP2B1/PSMD13/RAB27A/PSME1/CD209/PSMC1/CTSF/KIF15/KIF18A/CYBA/KLC1/PSMB4/HLA-E/AP2S1/PSMB3/PSMD2/TAPBPL/ARF1/IKBKG/PSMC6/PSMD7/DYNC1LI1/PSMA2/MFSD6/CCL21/ERAP1

GOBP_RRNA_METABOLIC_PROCESS GOBP_RRNA_METABOLIC_PROCESS GOBP_RRNA_METABOLIC_PROCESS 218 0.470121376 1.684085926 0.00125 0.030581767 0.024202852 4653 tags=51%, list=27%, signal=37% ISG20/NOP2/DDX21/UTP4/LYAR/ERI1/RRP36/TSR1/NIFK/PELO/RRS1/NOP58/PA2G4/RRP7A/DIMT1/BYSL/MRTO4/NAT10/EXOSC3/DDX56/UTP6/RPP40/NOP14/NOC4L/TRMT112/RRP12/UTP18/NOLC1/MAK16/RPL7L1/DCAF13/IMP4/GTPBP4/MPHOSPH6/UTP14A/EXOSC7/RRP15/RRP9/WDR43/FTSJ3/USP36/DDX18/RIOK1/UTP11/TBL3/MRM2/HEATR1/RRP1B/EXOSC9/WDR55/NHP2/TFB1M/RRP1/DDX10/SUV39H1/BMS1/EXOSC8/BUD23/CHD7/URB1/RPUSD4/WDR75/NOL9/UTP15/UTP3/PELP1/WDR12/RRP8/POP7/POP5/SRFBP1/LSM6/PIH1D1/RPF2/MPHOSPH10/NOB1/PES1/LAS1L/NOL10/EIF4A3/WDR46/SLFN13/WDR3/TEX10/METTL5/EMG1/EXOSC10/WDR36/DDX27/DDX51/UTP25/NOP9/SART1/SENP3/NOP10/DDX17/NOL6/THUMPD1/SNU13/EXOSC1/NOL11/NPM3/MTREX/MRM3/GEMIN4/NOL8/NSUN5/EXOSC2/PDCD11/FBLL1/GTF2H5

GOBP_CELLULAR_RESPONSE_TO_BIOTIC_STIMULUS GOBP_CELLULAR_RESPONSE_TO_BIOTIC_STIMULUS GOBP_CELLULAR_RESPONSE_TO_BIOTIC_STIMULUS 208 0.593945978 2.11725885 0.001251564 0.030581767 0.024202852 2602 tags=38%, list=15%, signal=33%

DEFA5/DEFA6/CXCL5/TNIP3/CXCL1/CD55/CXCL3/CXCL6/CXCL9/CXCL11/CXCL13/CXCL8/CD274/CXCL10/IL1B/WNT5A/CXCL2/CASP1/NOS2/LYN/SERPINE1/ASS1/TGFB1/PDE4B/TIGAR/PF4/LY96/TFPI/RIPK2/SGMS1/XBP1/CLEC7A/SBNO2/CARD16/KMO/VIM/HSPA5/FCGR2B/CEBPB/LILRB2/CD86/TLR2/HCK/ICAM1/PTAFR/CCL2/IRAK1/CD40/SIRPA/IL1A/LTF/MTDH/MRC1/PABPN1/PPBP/ADAM9/TNFRSF1B/IRAK2/NLRP7/TLR1/BTK/WFS1/IL6/AXL/LILRB1/CD180/CTSG/HAVCR2/CCR5/RELA/NOD2/LY86/MALT1/CDK4/PAF1/IL24/SYK/CD6/BMP6/AKT1

GOBP_ENDOTHELIAL_CELL_MIGRATION GOBP_ENDOTHELIAL_CELL_MIGRATION GOBP_ENDOTHELIAL_CELL_MIGRATION 207 0.466520503 1.664426656 0.001254705 0.030581767 0.024202852 3086 tags=35%, list=18%, signal=29% GREM1/S100P/ANXA1/CXCL13/LOXL2/FSTL1/AGT/SPARC/WNT5A/ADAMTS9/RGCC/ROBO1/CDH5/PIK3R3/TGFB1/STC1/ANXA3/HIF1A/PTGS2/S100A2/SMOC2/ITGB2/PTPRM/DCN/FAP/SRPX2/MET/CD40/SERPINF1/RAB13/MMRN2/VEGFC/KDR/TEK/CALR/PIK3CG/ADGRA2/SPRED1/FGFR1/PIK3CD/ITGB1BP1/CLEC14A/NRP1/HDAC7/LPXN/EGR3/EPHB4/PLXND1/AKT3/FGFBP1/ATP2B4/VASH1/NUS1/PLK2/TGFBR1/SEMA4A/RHOJ/GPX1/GLUL/FBXW7/ETS1/ANGPT2/AKT1/JCAD/EFNB2/APOE/FGF2/GPI/EMP2/STAT5A/NR2F2/PRCP

GOBP_REGULATION_OF_CELL_SUBSTRATE_ADHESION GOBP_REGULATION_OF_CELL_SUBSTRATE_ADHESION GOBP_REGULATION_OF_CELL_SUBSTRATE_ADHESION 207 0.476833987 1.701222548 0.001254705 0.030581767 0.024202852 3285 tags=35%, list=19%, signal=29% OLFM4/MMP12/GREM1/PLAU/COL1A1/NID1/GBP1/FERMT2/CCN1/SERPINE1/RAC2/LGALS1/PDPN/FN1/ITGA5/EFEMP2/CCR7/JAK2/DLC1/THY1/MAP4K4/POSTN/FOXF1/EGFL6/BCL6/FLNA/COL8A1/KDR/COL16A1/P4HB/EMILIN1/TEK/CALR/LIMCH1/TACSTD2/SPOCK2/ITGB1BP1/AGR2/NRP1/ITGA6/MACF1/CORO1C/DUSP22/SPOCK1/ADAM15/VCL/PLEKHA2/RCC2/SFRP1/JAG1/APOD/PHLDB2/FZD4/WNT4/NPNT/CEACAM6/FERMT1/MDK/ANGPT2/FAM107A/FBLN2/FBLN1/ROCK1/PPM1F/EMP2/ITGA3/HSD17B12/STK4/CDH13/GPM6B/LDB1/TBCD/NF2

GOBP_REGULATION_OF_LEUKOCYTE_MIGRATION GOBP_REGULATION_OF_LEUKOCYTE_MIGRATION GOBP_REGULATION_OF_LEUKOCYTE_MIGRATION 193 0.504137461 1.784220105 0.001256281 0.030581767 0.024202852 2123 tags=36%, list=13%, signal=32% GREM1/ANXA1/CXCL13/CXCL8/CXCL10/WNT5A/IL33/MSN/PLVAP/LYN/ITGA2/ADA/SERPINE1/SELP/CCR2/CD74/PLA2G7/RAC2/IL27RA/SLAMF8/ECM1/CCL20/S100A7/CCR1/ST3GAL4/IL1R1/MADCAM1/C5AR1/CCR7/CCL4/THY1/SELE/ICAM1/PTAFR/CCL2/CD300A/CCL24/CD81/RHOH/VEGFC/EMILIN1/JAM2/CALR/CCR6/ANO6/LRCH1/C3AR1/RIPOR2/CSF1R/PLCB1/GPSM3/CCL19/JAM3/MIF/CD200/CXCL14/IL23A/IL6/ADAM8/CREB3/RHOG/FUT4/STK10/AIF1/FPR2/APOD/SELENOK/NCKAP1L/NOD2

GOBP_RESPONSE_TO_TOPOLOGICALLY_INCORRECT_PROTEIN GOBP_RESPONSE_TO_TOPOLOGICALLY_INCORRECT_PROTEIN GOBP_RESPONSE_TO_TOPOLOGICALLY_INCORRECT_PROTEIN 193 0.486811797 1.722901915 0.001256281 0.030581767 0.024202852 3338 tags=41%, list=20%, signal=34% CXCL8/KDELR3/DERL3/CREB3L2/SDF2L1/MANF/SRPRB/DNAJB11/HYOU1/FBXO6/MYDGF/BAG3/DNAJB9/CLU/XBP1/BOK/HSPH1/DNAJC3/FICD/HSPA5/HSPA6/PREB/CCL2/PDIA6/HSPA13/SRPRA/ASNS/CREB3L1/EIF2S1/SERPINH1/VCP/DNAJA1/CALR/ERO1A/SSR1/FKBP14/AGR2/CCND1/HSP90B1/WFS1/HSPA4L/UGGT2/CREB3/TBL2/TLN1/ERN1/ERP44/HSPB8/ERP27/YIF1A/DERL2/SERP

1/CREB3L4/EDEM1/CHAC1/BFAR/F12/CANX/PTPN1/SEC31A/UGGT1/HSP90AB1/DERL1/CDK5RAP3/ATF4/UBE2J2/ARFGAP1/EXTL2/PPP2R5B/HSP90AA1/HSPA4/HSPD1/LMNA/UFD1/SELENOS/SHC1/HSPA14/HERPUD1/EDEM2/MBTPS1

GOBP_MYELOID_LEUKOCYTE_DIFFERENTIATION GOBP_MYELOID_LEUKOCYTE_DIFFERENTIATION GOBP_MYELOID_LEUKOCYTE_DIFFERENTIATION 192 0.470321818 1.664842458 0.001259446 0.030581767 0.024202852 2549 tags=34%, list=15%, signal=30% MMP9/LYN/FBN1/HCLS1/CD74/IFI16/TGFB1/PF4/EVI2B/IRF4/INHBA/NME1/TCIRG1/CCR1/SBNO2/KIT/TGFBR2/CCR7/GPR137B/CEBPB/PIAS3/TLR2/MAFB/TYROBP/CD81/SNX10/RASSF2/EFNA2/LEF1/LTF/FASN/TSPAN2/CLDN18/CSF1R/GPR183/CCL19/TESC/PIK3CD/BATF2/NKX2-3/NRROS/SLC9B2/ANXA2/TFE3/LIF/IL23A/SRP54/CD109/CASP8/LILRB4/SFRP1/PARP1/LILRB1/IFNG/LILRB3/CREB1/BATF3/BATF/TMEM64/TNFSF11/INPP5D/IRF7/CD4/HAX1/FSTL3/FBXW7

GOBP_MYELOID_LEUKOCYTE_MIGRATION GOBP_MYELOID_LEUKOCYTE_MIGRATION GOBP_MYELOID_LEUKOCYTE_MIGRATION 201 0.578911005 2.054651195 0.001259446 0.030581767 0.024202852 1898 tags=37%, list=11%, signal=33% S100A8/CXCL5/CXCL1/CXCL3/GREM1/CXCL6/CCL11/CXCL9/S100A9/ANXA1/CXCL11/CXCL13/CXCL8/CCL18/CXCL10/PECAM1/S100A12/CXCL2/CXCR2/LYN/SERPINE1/CCR2/CD74/PDE4B/PF4/PLA2G7/RAC2/SLAMF8/CCL20/S100A7/CCR1/FCER1G/ADGRE2/IL1R1/C5AR1/KIT/CCR7/CSF3R/ITGB2/CCL4/CCL2/CD300A/CCL24/CD81/RHOH/SIRPA/VEGFC/CKLF/CCL22/EMILIN1/VAV1/ANO6/PIK3CG/C3AR1/RIPOR2/CSF1R/PLCB1/PPBP/CCL19/PIK3CD/JAM3/MIF/CD200/PPIB/CD47/IL23A/SRP54/IL6/ADAM8/CREB3/RHOG/NUP85/AIF1/FPR2

GOBP_PROTEIN_FOLDING GOBP_PROTEIN_FOLDING GOBP_PROTEIN_FOLDING 205 0.536965219 1.913291605 0.001259446 0.030581767 0.024202852 3814 tags=43%, list=23%, signal=34% SDF2L1/DNAJB11/CD74/PRDX4/BAG2/PPIL1/BAG3/P3H1/FKBP5/CLU/PDIA4/DNAJC10/HSPH1/DNAJC3/HSPA5/HSPA6/GNAI2/PDIA6/HSPA13/QSOX1/LMAN1/GNB5/DNAJC1/P4HB/DNAJC2/VCP/DNAJA1/CALR/GNB4/ERO1A/SIL1/SPHK1/SNRNP70/HSP90B1/ERO1B/SACS/WFS1/PDIA3/HSPA4L/GNB2/PPIB/CCT3/CRYAB/CRTAP/SGTB/CCT8/NUDC/DNLZ/CHORDC1/ERP44/DNAJC24/ERP27/CCT5/CCT2/CCT6A/DNAJB6/CDC37/CCT4/CCT7/MESD/CANX/UGGT1/HSP90AB1/DERL1/RUVBL2/HSPBP1/PFDN1/NKTR/GANAB/GRPEL2/HSP90AA1/HSPD1/MLEC/TBCD/HSPA14/POFUT2/UNC45A/FKBP8/PDRG1/TBCEL/PSMC1/GRPEL1/PPIF/TRAP1/MOGS/PPIH/DNAJB5/NUDCD3

GOBP_LEUKOCYTE_CHEMOTAXIS GOBP_LEUKOCYTE_CHEMOTAXIS GOBP_LEUKOCYTE_CHEMOTAXIS 204 0.592170045 2.107370209 0.001262626 0.030581767 0.024202852 2346 tags=42%, list=14%, signal=37% S100A8/CXCL5/CXCL1/CXCL3/GREM1/CXCL6/CCL11/CXCL9/S100A9/ANXA1/CXCL11/CXCL13/CXCL8/CCL18/CXCL10/S100A12/WNT5A/CXCL2/CXCR2/LYN/ALOX5/SERPINE1/CCR2/CD74/PDE4B/PF4/PLA2G7/RAC2/SLAMF8/CCL20/S100A7/CCR1/FCER1G/ADGRE2/C5AR1/KIT/CCR7/CSF3R/ITGB2/CCL4/CXCR4/CCL2/FFAR2/CCL24/CORO1A/VEGFC/CKLF/CCL22/S1PR1/CH25H/CALR/VAV1/CCR6/ANO6/PIK3CG/C3AR1/RIPOR2/CSF1R/LYST/GPR183/PPBP/GPSM3/CCL19/PIK3CD/JAM3/MIF/PPIB/CXCL14/IL23A/SRP54/IL6/ADAM8/CREB3/NUP85/AIF1/FPR2/CCR5/HSD3B7/NCKAP1L/TNFSF11/NOD2/CXCL16/PIP5K1C/SWAP70/IL16/CMKLR1

GOBP_POSITIVE_REGULATION_OF_IMMUNE_EFFECTOR_PROCESS GOBP_POSITIVE_REGULATION_OF_IMMUNE_EFFECTOR_PROCESS GOBP_POSITIVE_REGULATION_OF_IMMUNE_EFFECTOR_PROCESS 202 0.479464503 1.70214159 0.001262626 0.030581767 0.024202852 2185 tags=31%, list=13%,

signal=27%

IL13RA2/ANXA1/IL1B/MZB1/WNT5A/CADM1/SLC7A5/IL33/NOS2/C3/LYN/CCR2/CD74/TGFB1/AZGP1/RAC2/HLA-DRA/STXBP1/NFKBIZ/PTPRC/HLA-DMB/RIPK2/ADGRE2/XBP1/TNFSF13/CLEC7A/IL1R1/MAD2L2/ITGB2/DDX21/CD86/FOXF1/SASH3/FGR/PTAFR/CD300A/HK1/FFAR2/TYROBP/CD81/CD40/ITGAM/LAPTM5/GPRC5B/VAV1/CD84/IL12RB1/CCL19/IL18R1/EXOSC3/BTK/LAG3/IL23A/IL6/MR1/TNFRSF4/LILRB1/IFNG/MLH1/SLAMF6/RBP4/NOD2/HLX

GOBP_REGULATION_OF_LEUKOCYTE_MEDIATED_IMMUNITY

GOBP_REGULATION_OF_LEUKOCYTE_MEDIATED_IMMUNITY

GOBP_REGULATION_OF_LEUKOCYTE_MEDIATED_IMMUNITY 197 0.50625733

1.791882679 0.001262626 0.030581767 0.024202852 2956 tags=37%, list=17%, signal=31%

C4BPB/CXCL6/C4BPA/IL13RA2/IL1B/SERPINB9/CADM1/IL7R/NOS2/C3/LYN/CCR2/TGFB1/AZGP1/JAK3/RAC2/HLA-DRA/STXBP1/IL27RA/PTPRC/ADGRE2/TNFSF13/CLEC7A/IL1R1/SERPINB4/BST2/MAD2L2/ITGB2/FCGR2B/DDX21/FOXF1/SASH3/FGR/ICAM1/PTAFR/CD300A/TYROBP/CD81/CD40/BCL6/ITGAM/VAV1/CD84/IL12RB1/IL18R1/TNFRSF1B/EXOSC3/BTK/LAG3/IL23A/IL6/MR1/LILRB4/LILRB1/HAVCR2/MLH1/SLAMF6/NOD2/MALT1/DNASE1L3/BLK/RASGRP1/SYK/THOC1/IL18/WAS/PVR/PLA2G3/ARRB2/FES/AP1G1/STAT5B

GOBP_T_CELL_PROLIFERATION GOBP_T_CELL_PROLIFERATION

GOBP_T_CELL_PROLIFERATION 186 0.534037912 1.883837145 0.001269036

0.030581767 0.024202852 2987 tags=38%, list=18%, signal=32%

CD55/ANXA1/CD274/IDO1/IL1B/CTPS1/MSN/SLC7A1/CCR2/JAK3/RAC2/IL27RA/PNP/PTPRC/HLA-DMB/RIPK2/VCAM1/TNFSF13B/TGFBR2/FYN/SHH/CEBPB/LILRB2/CD86/SASH3/CD81/HLA-DPA1/CORO1A/IL1A/IGFBP2/IRF1/IL6ST/CD276/PIK3CG/IL12RB1/DOCK2/CCL19/TNFRSF1B/TWSG1/EBI3/PRNP/BTN3A1/IL23A/IL6/LILRB4/TNFRSF4/TMEM131L/LILRB1/AIF1/GPNMB/HAVCR2/SELENOK/WNT4/NCKAP1L/MALT1/CD4/RASGRP1/GLMN/SYK/CD6/LRRC32/RASAL3/IL18/DOCK8/PRKAR1A/DLG5/BTN2A2/PSMB10/STAT5B/HLA-DPB1/CTLA4

GOBP_T_CELL_RECEPTOR_SIGNALING_PATHWAY

GOBP_T_CELL_RECEPTOR_SIGNALING_PATHWAY

GOBP_T_CELL_RECEPTOR_SIGNALING_PATHWAY 186 0.498979309 1.760166713

0.001269036 0.030581767 0.024202852 4036 tags=49%, list=24%, signal=37%

GBP1/ADA/RFTN1/LCP2/PDE4B/HLA-DQB1/HLA-DRA/NFKBIZ/PTPRC/THEMIS2/RIPK2/PSME4/PSMB9/KCNN4/CCR7/FYN/THY1/CD300A/PLCG2/CD81/HLA-DQA1/HLA-DPA1/LAPTM5/FYB1/PSMD14/DGKZ/RAB29/CD276/PSMD12/LCK/PSMC2/PSMB2/PSMA3/PIK3CD/PSMD1/PRNP/BTN3A1/ITK/PSMA5/EIF2B3/LILRB4/PSMD6/PSMB5/PSMC4/BTN3A3/RELA/INPP5D/EIF2B2/PSMB1/PSMA1/MALT1/CD4/PSME3/CD3D/CUL1/PSMA7/PSMB8/PSMC5/WAS/LIME1/UBE2N/BTN2A2/PSMB10/EIF2B5/HLA-DPB1/CTLA4/PSMB6/EIF2B4/HRAS/PSMA4/RC3H2/LAT/PSMD13/PSME1/CD247/CARD11/BTN2A1/PSMC1/PRKD2/DUSP3/NECTIN2/PSMB4/PSMB3/PSMD2/IKBKG/PSMC6/PSMD7/PSMA2/CSK/ZAP70/PSMD4

GOBP_CELLULAR_RESPONSE_TO_MOLECULE_OF_BACTERIAL_ORIGIN

GOBP_CELLULAR_RESPONSE_TO_MOLECULE_OF_BACTERIAL_ORIGIN

GOBP_CELLULAR_RESPONSE_TO_MOLECULE_OF_BACTERIAL_ORIGIN 184 0.616732742

2.17370441 0.001270648 0.030581767 0.024202852 2362 tags=38%, list=14%, signal=33%

DEFA5/DEFA6/CXCL5/TNIP3/CXCL1/CD55/CXCL3/CXCL6/CXCL9/CXCL11/CXCL13/CXCL8/CD27

4/CXCL10/IL1B/WNT5A/CXCL2/CASP1/NOS2/LYN/SERPINE1/ASS1/TGFB1/PDE4B/PF4/LY96/TFPI/RIPK2/SGMS1/XBP1/SBNO2/CARD16/KMO/VIM/FCGR2B/CEBPB/LILRB2/CD86/TLR2/HCK/ICAM1/PTAFR/CCL2/IRAK1/CD40/SIRPA/IL1A/LTF/MTDH/MRC1/PABPN1/PPBP/ADAM9/TNFRSF1B/IRAK2/NLRP7/TLR1/IL6/AXL/LILRB1/CD180/CTSG/HAVCR2/CCR5/RELA/NOD2/LY86/MALT1/CDK4/PAF1

GOBP_NEGATIVE_REGULATION_OF_CELL_ACTIVATION GOBP_NEGATIVE_REGULATION_OF_CELL_ACTIVATION GOBP_NEGATIVE_REGULATION_OF_CELL_ACTIVATION 183 0.520128589 1.833401167 0.001270648 0.030581767 0.024202852 3434 tags=41%, list=20%, signal=33% IL13RA2/ANXA1/CD274/IDO1/MNDA/THBD/SERPINE2/LYN/CCR2/LAX1/CD74/JAK3/INHBA/PTPRC/CBFB/GAL/PDGFRA/SAMSN1/FCGR2B/SHH/CEBPB/LILRB2/CD86/ADGRF5/FOXF1/FGR/CD300A/TYROBP/LST1/BCL6/CST7/LAPTM5/EMILIN1/BANK1/IRF1/RIPOR2/CD84/RUNX3/TWSG1/PRNP/BTK/CD200/LAG3/CD300LF/LILRB4/AXL/TMEM131L/SFRP1/LILRB1/GPNMB/HAVCR2/INPP5D/SYT11/HLX/BLK/GLMN/MDK/LRRC32/TYRO3/THOC1/PRKAR1A/C1QTNF1/DLG5/BTN2A2/APOE/CTLA4/TBC1D10C/RC3H2/IL2RA/PLA2G2D/PKN1/HMGB1/CX3CL1/CR1/NOS3

GOBP_PRODUCTION_OF_MOLECULAR_MEDIATOR_OF_IMMUNE_RESPONSE GOBP_PRODUCTION_OF_MOLECULAR_MEDIATOR_OF_IMMUNE_RESPONSE GOBP_PRODUCTION_OF_MOLECULAR_MEDIATOR_OF_IMMUNE_RESPONSE 184 0.500361952 1.763549926 0.001270648 0.030581767 0.024202852 2677 tags=34%, list=16%, signal=29% CD55/IL13RA2/SPINK5/IL1B/MZB1/WNT5A/SLC7A5/IL33/CCR2/CD74/TGFB1/HLA-DQB1/JAK3/IL27RA/PTPRC/DNAJB9/XBP1/TNFSF13/CLEC7A/IL1R1/KIT/BST2/MAD2L2/FCGR2B/DDX21/CD86/SASH3/HK1/SAMHD1/FFAR2/CD81/CD40/BCL6/LAPTM5/GPRC5B/CCR6/IL18R1/TCF3/FCRL3/TNFRSF1B/EXOSC3/BTK/VPREB3/EXO1/IL6/MSH6/LILRB4/TNFRSF4/LILRB1/MLH1/BATF/RBP4/NBN/NOD2/MCM3AP/SWAP70/MALT1/TGFB3/THOC1/CGAS/POLB/IL18

GOBP_RESPONSE_TO_INTERLEUKIN_1 GOBP_RESPONSE_TO_INTERLEUKIN_1 GOBP_RESPONSE_TO_INTERLEUKIN_1 184 0.496637119 1.750421571 0.001270648 0.030581767 0.024202852 2745 tags=37%, list=16%, signal=31% IL1RN/CHI3L1/CCL11/ANXA1/CXCL8/CCL18/IL1B/CD38/GBP1/HYAL1/TFPI/CCL20/RIPK2/HIF1A/PSME4/IL1R1/PSMB9/KMO/CCL4/CEBPB/SELE/ICAM1/CCL2/HYAL2/CCL24/IRAK1/CD40/SIRPA/IL1A/KLF2/CCL22/PSMD14/PSMD12/GBP2/PSMC2/PLCB1/CCL19/PSMB2/PSMA3/PSMD1/IRAK2/AKAP12/NLRP7/RORA/CD47/PSMA5/IL6/PTGIS/SFRP1/PSMD6/PSMB5/PSMC4/ZBP1/VRK2/RELA/NOD2/PSMB1/MTHFR/PSMA1/CCL7/PSME3/ETS1/TANK/CUL1/PSMA7/PSMB8/PSMC5/UBE2N

GOBP_LYMPHOCYTE_ACTIVATION_INVOLVED_IN_IMMUNE_RESPONSE GOBP_LYMPHOCYTE_ACTIVATION_INVOLVED_IN_IMMUNE_RESPONSE GOBP_LYMPHOCYTE_ACTIVATION_INVOLVED_IN_IMMUNE_RESPONSE 180 0.515649836 1.811273759 0.001273885 0.030581767 0.024202852 3388 tags=42%, list=20%, signal=34% POU2AF1/ANXA1/ADA/CD74/TGFB1/JAK3/IRF4/HLA-DRA/IL27RA/LGALS1/NFKBIZ/ENTPD7/PTPRC/HLA-DMB/RIPK2/FCER1G/XBP1/TNFSF13/CD19/MAD2L2/FCGR2B/CD86/ICAM1/PLCG2/CD81/CD40/CORO1A/BCL6/APBB1IP/LEF1/MFNG/STAT3/LCP1/CCR6/IL12RB1/DOCK11/GPR183/CCL19/IL18R1/EXOSC3/RORA/NKX2-3/IL23A/EXO1/IL6/MSH6/ITM2A/LILRB1/CD180/IFNG/HAVCR2/MLH1/BATF/SLAMF6/NBN/HLX/SEMA4A/SWAP70/MALT1/LY9/MDK/THOC1/DLL1/IL18/ATP7A/AP1G1/RNF8/HSPD1/RC3H2/ITFG2/RAB27A/SLC15A4/EOMES/HMGB1/CR1

GOBP_ANTIGEN_PROCESSING_AND_PRESENTATION_OF_PEPTIDE_ANTIGEN
GOBP_ANTIGEN_PROCESSING_AND_PRESENTATION_OF_PEPTIDE_ANTIGEN
GOBP_ANTIGEN_PROCESSING_AND_PRESENTATION_OF_PEPTIDE_ANTIGEN 182
0.54317187 1.912534032 0.00127551 0.030581767 0.024202852 4036
tags=52%, list=24%, signal=40%
NCF2/HLA-DMA/CD74/SEC24D/HLA-DQB1/AZGP1/HLA-DRA/FCGR1B/HLA-DMB/FCER1G/PSME4/PSMB9/TAP2/CLEC4A/FCGR2B/TAP1/SEC13/HLA-DQA1/HLA-DPA1/PSMD14/HLA-DOB/CALR/AP1S3/PSMD12/PSMC2/KLC2/PSMB2/CENPE/PSMA3/AP1S2/PSMD1/SEC24C/CTSE/KIF23/PDIA3/LAG3/ITGAV/PSMA5/SEC24A/MR1/CTSL/NCF4/PSMD6/PSMB5/PSMC4/AP1M1/KIF2C/KIF11/SEC22B/PSMB1/KIF4A/PSMA1/ACE/CANX/SEC31A/PSME3/TAPBP/PSMA7/PSMB8/PSMC5/CYBB/MARCHF1/AP1G1/PSMB10/HLA-DPB1/PSMB6/IDE/PSMA4/RACGAP1/DCTN6/AP2B1/PSMD13/PSME1/CD209/PSMC1/CTSF/KIF15/KIF18A/CYBA/KLC1/PSMB4/HLA-E/AP2S1/PSMB3/PSMD2/TAPBPL/ARF1/IKBKG/PSMC6/PSMD7/DYNC1LI1/PSMA2/MFSD6/ERAP1/PSMD4
GOBP_RESPONSE_TO_INTERFERON_GAMMA GOBP_RESPONSE_TO_INTERFERON_GAMMA
GOBP_RESPONSE_TO_INTERFERON_GAMMA 176 0.555698755 1.942583239
0.00128866 0.030581767 0.024202852 2977 tags=43%, list=18%, signal=36%
CCL11/CCL18/KYNU/WNT5A/CLDN1/IFITM2/GBP1/IFITM3/CASP1/NOS2/TRIM22/ASS1/CD44/CD74/IFITM1/HLA-DQB1/IRF4/HLA-DRA/STXBP1/FCGR1B/CCL20/OAS2/SOCS3/ZYX/VCAM1/PARP9/VIM/BST2/JAK2/CCL4/STAT1/TLR2/HCK/ICAM1/PTAFR/CCL2/NLRC5/CCL24/HLA-DQA1/CD40/HLA-DPA1/SIRPA/GBP5/PARP14/CCL22/MRC1/IRF1/OAS3/GBP2/IL12RB1/CCL19/CYP27B1/CD47/SYNCRIP/PML/IRF9/IFNG/AIF1/EPRS1/SHFL/CXCL16/IRF7/CDC37/NMI/HSP90AB1/CCL7/CDC42EP2/WAS/ACTR3/IRF3/TRIM5/TRIM21/JAK1/GBP4/HLA-DPB1/TRIM8
GOBP_MONONUCLEAR_CELL_MIGRATION GOBP_MONONUCLEAR_CELL_MIGRATION
GOBP_MONONUCLEAR_CELL_MIGRATION 174 0.570682614 1.991950242 0.001295337
0.030581767 0.024202852 2688 tags=41%, list=16%, signal=35%
GREM1/CCL11/ANXA1/CXCL11/CXCL13/CCL18/CXCL10/PECAM1/S100A12/WNT5A/CXCR2/SPNS2/MSN/LYN/ALOX5/SERPINE1/CCR2/PLA2G7/IL27RA/SLAMF8/ECM1/CCL20/S100A7/CCR1/MADCAM1/C5AR1/CCR7/CCL4/CXCR4/ICAM1/CCL2/CCL24/SIRPA/CKLF/CCL22/JAM2/S1PR1/CH25H/CALR/CCR6/ANO6/PIK3CG/LRCH1/C3AR1/RIPOR2/CSF1R/PLCB1/GPR183/CCL19/PIK3CD/CD200/CXCL14/CD47/IL6/ADAM8/CREB3/STK10/AIF1/FPR2/APOD/CCR5/SELENOK/HSD3B7/TNFSF11/CXCL16/CMKLR1/ARTN/MDK/CCL7/AKT1/ITGA4/DOCK8
GOBP_REGULATION_OF_LYMPHOCYTE_DIFFERENTIATION
GOBP_REGULATION_OF_LYMPHOCYTE_DIFFERENTIATION
GOBP_REGULATION_OF_LYMPHOCYTE_DIFFERENTIATION 169 0.500471232 1.742838455
0.00130039 0.030581767 0.024202852 2677 tags=35%, list=16%, signal=30%
VNN1/ANXA1/SPINK5/IL7R/ADA/CCR2/CD74/JAK3/IRF4/HLA-DRA/INHBA/SLAMF8/NFKBIZ/PNP/PTPRC/CBFB/RIPK2/XBP1/TGFBR2/SHH/LILRB2/CD86/SASH3/CD27/RHOH/BCL6/LEF1/IRF1/IL12RB1/CCL19/METTL3/DUSP10/FANCD2/FCRL3/RUNX3/CD2/BTK/IKZF3/ITPKB/LAG3/EGR3/IL23A/ADAM8/LILRB4/AXL/TMEM131L/SFRP1/IFNG/AP3D1/PRDM1/NCKAP1L/INPP5D/HLX/MALT1/ZBTB16/MDK/SYK/TNFRSF18/IL18
GOBP_REGULATION_OF_RESPONSE_TO_CYTOKINE_STIMULUS
GOBP_REGULATION_OF_RESPONSE_TO_CYTOKINE_STIMULUS
GOBP_REGULATION_OF_RESPONSE_TO_CYTOKINE_STIMULUS 162 0.50046266
1.73328022 0.001310616 0.030581767 0.024202852 2567 tags=32%, list=15%,

signal=27%
IL1RN/MMP12/WNT5A/CAV1/ROBO1/PXDN/CASP1/CD74/BIRC3/ECM1/PTPRC/CBFB/RIPK2/HIF1A/SOCS3/IL1R1/PARP9/CARD16/JAK2/CXCR4/STAT1/TLR2/NLRC5/SAMHD1/IRAK1/CASP4/PARP14/LAPTM5/IFNAR2/SPHK1/METTL3/ABCE1/IRAK2/USP18/CCDC3/CASP8/IL6/TSLP/CD300LF/AXL/IFNG/ZBP1/VRK2/TRAF1/IRF7/CDC37/PTPN1/CLIP3/HSP90AB1/ADAR/SYK/CYLD
GOBP_REGULATION_OF_ADAPTIVE_IMMUNE_RESPONSE
GOBP_REGULATION_OF_ADAPTIVE_IMMUNE_RESPONSE
GOBP_REGULATION_OF_ADAPTIVE_IMMUNE_RESPONSE 161 0.546301554 1.889461401 0.001319261 0.030581767 0.024202852 2795 tags=35%, list=17%, signal=29%
C4BPB/C4BPA/ANXA1/CD274/IL1B/IL7R/IL33/C3/ADA/CCR2/TGFB1/AZGP1/JAK3/HLA-DRA/IL27RA/NFKBIZ/PTPRC/RIPK2/TNFSF13/IL1R1/TNFSF13B/SAMSN1/MAD2L2/FCGR2B/TRIM27/SASH3/CD81/CD40/BCL6/IRF1/IL6ST/IL12RB1/CCL19/IL18R1/DUSP10/TNFRSF1B/EXOSC3/BTK/IL23A/IL6/MR1/LILRB4/LILRB1/HAVCR2/MLH1/NOD2/HLX/IRF7/MALT1/CD4/THOC1/IL18/WAS/PVR/ADCY7/CD48
GOBP_TUMOR_NECROSIS_FACTOR_MEDIATED_SIGNALING_PATHWAY
GOBP_TUMOR_NECROSIS_FACTOR_MEDIATED_SIGNALING_PATHWAY
GOBP_TUMOR_NECROSIS_FACTOR_MEDIATED_SIGNALING_PATHWAY 161 0.52952612 1.831441181 0.001319261 0.030581767 0.024202852 4430 tags=50%, list=26%, signal=38%
TNFRSF17/CASP1/PLVAP/AIM2/BIRC3/TNFRSF11B/TNFSF13/PSME4/PSMB9/CARD16/TNFSF13B/JAK2/TNFRSF9/STAT1/PIAS3/TNFRSF12A/CD27/CD40/CASP4/LAPTM5/PSMD14/TNFSF15/CLDN18/PSMD12/PSMC2/TRAF5/PSMB2/SPHK1/PSMA3/PSMD1/TNFRSF1B/PSMA5/CCDC3/CASP8/TNFRSF4/PSMD6/PSMB5/PSMC4/RELA/LTB/TNFSF11/TRAF1/PSMB1/PSMA1/CLIP3/PSME3/SYK/TRAF3/CYLD/TNFRSF18/PSMA7/PSMB8/PSMC5/TXNDC17/PSMB10/PSMB6/GSTP1/PSMA4/OTULIN/PSMD13/PSME1/PSMC1/MAP3K14/PSMB4/TNFRSF25/ADAM17/PSMB3/PSMD2/IKBKG/PSMC6/PSMD7/TNFSF8/PSMA2/PSMD4/ACTN4/PSMD9/TNFRSF13B/CARD8/RBCK1/LTBR/ILK
GOBP_TISSUE_REMODELING GOBP_TISSUE_REMODELING GOBP_TISSUE_REMODELING 158 0.549029292 1.896673576 0.001321004 0.030581767 0.024202852 2912 tags=38%, list=17%, signal=32%
TIMP1/CTHRC1/IGFBP5/GREM1/GJA1/SPP1/AGT/TGM2/CD38/CAV1/MMP2/CCR2/TGFB1/BGN/RAC2/TNFRSF11B/RSPO3/F2R/TCIRG1/HIF1A/GPR137B/TIE1/SNX10/RASSF2/FLNA/EFNA2/DDR2/IL1A/S1PR1/CLDN18/CSF1R/LIPA/ACP5/LIF/IL23A/IL6/ADAM8/AXL/SFRP1/GPNMB/JAG1/RAB3D/DEF8/TMEM119/TMEM64/TNFSF11/PPP3CA/P2RX7/INPP5D/ACE/TGFB3/MDK/SYK/IL18/ATP7A/CHD7/SUCO/ROCK1/ADRB2/MITF
GOBP_PLATELET_ACTIVATION GOBP_PLATELET_ACTIVATION
GOBP_PLATELET_ACTIVATION 152 0.50621138 1.739416204 0.001324503 0.030581767 0.024202852 1874 tags=25%, list=11%, signal=23%
VWF/COL1A2/COL1A1/SLC7A11/THBD/GNA15/F2RL2/COL3A1/SERPINE2/LYN/SELP/LCP2/HBB/PF4/STXBP1/PDPN/F2R/FCER1G/FN1/GNA14/PDGFRA/FYN/PLEK/MYL9/PLCG2/CD40/FLNA/DGKZ/VAV1/PIK3CG/LCK/PRKCB/PRKCH/PIK3R5/VCL/IL6/AXL/PEAR1/TLN1
GOBP_CELL_KILLING GOBP_CELL_KILLING GOBP_CELL_KILLING 156 0.604333222 2.081514305 0.001329787 0.030581767 0.024202852 3101 tags=38%, list=18%, signal=31%
DEFA5/DEFA6/CD55/CXCL6/S100A12/SERPINB9/CADM1/IL7R/SLAMF7/NOS2/C3/CTSH/PF4/

AZGP1/HLA-DRA/GZMB/PTPRC/CLEC7A/APOL1/SERPINB4/FCGR2B/ICAM1/TYROBP/CORO1A/ITGAM/LTF/SEMG1/VAV1/KRT6A/CTSC/LYZ/LYST/IL12RB1/TUBB/LAG3/IL23A/CASP8/MR1/LILRB1/IFNG/CTSG/HAVCR2/SLAMF6/P2RX7/RNF19B/CD59/DNASE1L3/RASGRP1/HSP90AB1/SYK/GNLY/IL18/HPRT1/PVR/ARRB2/AP1G1/STAT5B/EMP2/IL18RAP

GOBP_CELLULAR_RESPONSE_TO_TOPOLOGICALLY_INCORRECT_PROTEIN

GOBP_CELLULAR_RESPONSE_TO_TOPOLOGICALLY_INCORRECT_PROTEIN

GOBP_CELLULAR_RESPONSE_TO_TOPOLOGICALLY_INCORRECT_PROTEIN 156 0.505474376 1.741013245 0.001329787 0.030581767 0.024202852 3338 tags=41%, list=20%, signal=33%

CXCL8/KDELR3/DERL3/CREB3L2/SDF2L1/SRPRB/DNAJB11/HYOU1/MYDGF/BAG3/DNAJB9/XBP1/BOK/DNAJC3/FICD/HSPA5/HSPA6/PREB/CCL2/PDIA6/HSPA13/SRPRA/ASNS/CREB3L1/EIF2S1/VCP/CALR/ERO1A/SSR1/FKBP14/AGR2/CCND1/HSP90B1/WFS1/UGGT2/CREB3/TBL2/TLN1/ERN1/HSPB8/YIF1A/DERL2/SERP1/CREB3L4/EDEM1/BFAR/CANX/PTPN1/SEC31A/UGGT1/DERL1/CDK5RAP3/ATF4/ARFGAP1/EXTL2/PPP2R5B/HSPD1/LMNA/UFD1/SELENOS/SHC1/HSPA14/HERPUD1/MBTPS1

GOBP_POSITIVE_REGULATION_OF_NF_KAPPAB_TRANSCRIPTION_FACTOR_ACTIVITY

GOBP_POSITIVE_REGULATION_OF_NF_KAPPAB_TRANSCRIPTION_FACTOR_ACTIVITY

GOBP_POSITIVE_REGULATION_OF_NF_KAPPAB_TRANSCRIPTION_FACTOR_ACTIVITY 153 0.510911271 1.751091093 0.001331558 0.030581767 0.024202852 3813 tags=41%, list=23%, signal=32%

S100A8/GREM1/S100A9/IL1B/S100A12/AGT/WNT5A/CAV1/TRIM22/AIM2/CLU/RIPK2/CARD16/ITGB2/TRIM27/TLR2/ICAM1/IRAK1/CD40/LTF/TRIM15/MTDH/STAT3/TRAF5/SPHK1/PRKCB/IL18R1/IRAK2/BTK/PRKCH/CFLAR/ADAM8/ARHGEF2/SLCO3A1/RNF25/RELA/TNFSF11/NOD2/TRAF1/NFKB2/MALT1/NPM1/IL18/UBE2N/TRIM5/TRIM21/IRAK3/TRIM8/IL18RAP/NLRC4/DHX33/PRKD1/ERC1/CARD11/CX3CL1/RHEBL1/PRKD2/MID2/DDRGK1/TERF2IP/IKBKG/FLOT2

GOBP_POSITIVE_REGULATION_OF_HEMOPOIESIS

GOBP_POSITIVE_REGULATION_OF_HEMOPOIESIS

GOBP_POSITIVE_REGULATION_OF_HEMOPOIESIS 149 0.507342025 1.739543075 0.001333333 0.030581767 0.024202852 2523 tags=38%, list=15%, signal=32%

VNN1/ANXA1/IL7R/ADA/HCLS1/CD74/TGFB1/PF4/EVI2B/HLA-DRA/NFKBIZ/PNP/PTPRC/CBFB/RIPK2/CCR1/XBP1/TGFBR2/SHH/LILRB2/CD86/SASH3/CD27/TYROBP/RHOH/BCL6/LEF1/IL12RB1/CCL19/TESC/DUSP10/RUNX3/BTK/SLC9B2/ITPKB/LIF/EGR3/IL23A/CASP8/ADAM8/LILRB4/AXL/IFNG/CREB1/AP3D1/TMEM64/NCKAP1L/TNFSF11/INPP5D/HLX/MALT1/CD4/HAX1/ZBTB16/MDK/SYK

GOBP_REGULATION_OF_RESPONSE_TO_WOUNDING

GOBP_REGULATION_OF_RESPONSE_TO_WOUNDING

GOBP_REGULATION_OF_RESPONSE_TO_WOUNDING 151 0.51843241 1.776506156 0.001336898 0.030581767 0.024202852 3030 tags=33%, list=18%, signal=27%

DUOX2/REG3A/GJA1/ANXA1/SPP1/PLAU/CLDN1/CAV1/THBD/FERMT2/SERPINE2/ALOX5/SERPING1/F3/SERPINE1/TFPI/F2R/XBP1/ST3GAL4/CLEC7A/PDGFRA/SMOC2/CXCR4/TNFRSF12A/PLAT/FAP/FLNA/WFDC1/ANO6/HPSE/CD109/MAP2K1/PHLDB2/WNT4/PTPRS/VKORC1/SERPINF2/F12/FERMT1/FOXA2/MDK/SIGLEC10/ATP7A/C1QTNF1/APOE/FGF2/PLAUR/CD34/PROS1/HRAS

GOBP_REGULATION_OF_LYMPHOCYTE_MEDIATED_IMMUNITY

GOBP_REGULATION_OF_LYMPHOCYTE_MEDIATED_IMMUNITY

GOBP_REGULATION_OF_LYMPHOCYTE_MEDIATED_IMMUNITY 146 0.514143648
1.759850457 0.001340483 0.030581767 0.024202852 3111 tags=34%, list=18%, signal=28%
C4BPB/C4BPA/IL1B/SERPINB9/CADM1/IL7R/C3/CCR2/TGFB1/AZGP1/HLA-DRA/IL27RA/PTPRC/TNFSF13/IL1R1/SERPINB4/MAD2L2/FCGR2B/SASH3/CD81/CD40/BCL6/VAV1/IL12RB1/IL18R1/TNFRSF1B/EXOSC3/BTK/LAG3/IL23A/IL6/MR1/LILRB4/LILRB1/HAVCR2/MLH1/SLAMF6/NOD2/MALT1/RASGRP1/THOC1/IL18/WAS/PVR/ARRB2/AP1G1/STAT5B/IL18RAP/HSPD1
GOBP_POSITIVE_REGULATION_OF_VASCULATURE_DEVELOPMENT
GOBP_POSITIVE_REGULATION_OF_VASCULATURE_DEVELOPMENT
GOBP_POSITIVE_REGULATION_OF_VASCULATURE_DEVELOPMENT 144 0.550977809
1.88640239 0.001347709 0.030581767 0.024202852 1805 tags=31%, list=11%, signal=28%
CHI3L1/GREM1/CCL11/CXCL8/IL1B/WNT5A/HYAL1/C3/CTSH/ENG/F3/SERPINE1/PKM/CDH5/MYDGF/ANXA3/APLNR/ECM1/HIF1A/XBP1/ITGA5/TWIST1/C5AR1/AGO2/TGFBR2/SMOC2/ITGB2/CXCR4/TIE1/CCL24/CD40/VEGFC/IL1A/KDR/TEK/MTDH/C3AR1/LRG1/SPHK1/PRKCB/PIK3CD/HK2/AKT3/ITGAX/PTGIS
GOBP_ACTIVATION_OF_INNATE_IMMUNE_RESPONSE
GOBP_ACTIVATION_OF_INNATE_IMMUNE_RESPONSE
GOBP_ACTIVATION_OF_INNATE_IMMUNE_RESPONSE 138 0.543584469 1.85344662
0.001358696 0.030581767 0.024202852 4036 tags=51%, list=24%, signal=40%
MNDA/MUC5B/LYN/IFI16/AIM2/STING1/FCER1G/MUC4/PSME4/PSMB9/CLEC4A/FCN1/FYN/HCK/FFAR2/PLCG2/TYROBP/ICAM2/PSMD14/CLEC4E/PSMD12/PSMC2/MUC1/PSMB2/PSMA3/PSMD1/PSMA5/MUC5AC/PSMD6/MUC2/PSMB5/PSMC4/ZBP1/RELA/PSMB1/PSMA1/MALT1/PSME3/SYK/CGAS/CUL1/PSMA7/PSMB8/PSMC5/TRIM5/TBK1/PSMB10/HSP90AA1/PSMB6/HRAS/PSMA4/SFPQ/NLRC4/PSMD13/PSME1/HMGB1/CARD11/XRCC5/CD209/PSMC1/PSMB4/MUC17/PSMB3/PSMD2/IKBKG/PSMC6/PSMD7/MUC12/CLEC4D/PSMA2/PSMD4
GOBP_ALPHA_BETA_T_CELL_ACTIVATION GOBP_ALPHA_BETA_T_CELL_ACTIVATION
GOBP_ALPHA_BETA_T_CELL_ACTIVATION 138 0.561027576 1.912921951 0.001358696
0.030581767 0.024202852 2728 tags=43%, list=16%, signal=36%
CD55/ANXA1/CD274/ADA/CCR2/JAK3/IRF4/HLA-DRA/NFKBIZ/PNP/ENTPD7/PTPRC/CBFB/TCIRG1/RIPK2/HSPH1/CLEC4A/TGFBR2/SHH/CD86/SASH3/CD300A/CD81/BCL6/LEF1/STAT3/IRF1/IL12RB1/GPR183/DOCK2/CCL19/IL18R1/RUNX3/TWSG1/EBI3/RORA/NKX2-3/ITPKB/ITK/IL23A/IL6/CTSL/LILRB4/LILRB1/IFNG/AP3D1/BATF/PRDM1/SLAMF6/NCKAP1L/HLX/SEMA4A/MALT1/LY9/ZBTB16/SYK/RASAL3/IL18/ATP7A
GOBP_GRANULOCYTE_MIGRATION GOBP_GRANULOCYTE_MIGRATION
GOBP_GRANULOCYTE_MIGRATION 138 0.600416265 2.047224598 0.001358696
0.030581767 0.024202852 1766 tags=38%, list=10%, signal=35%
S100A8/CXCL5/CXCL1/CXCL3/CXCL6/CCL11/CXCL9/S100A9/ANXA1/CXCL11/CXCL13/CXCL8/CCL18/CXCL10/PECAM1/S100A12/CXCL2/CXCR2/CD74/PDE4B/PF4/RAC2/SLAMF8/CCL20/S100A7/FCER1G/ADGRE2/IL1R1/C5AR1/CCR7/CSF3R/ITGB2/CCL4/CCL2/CD300A/CCL24/RHOH/CKLF/CCL22/VAV1/PIK3CG/C3AR1/RIPOR2/CSF1R/PPBP/CCL19/PIK3CD/JAM3/PPIB/IL23A/SRP54/ADAM8/RHOG
GOBP_REGULATION_OF_CALCIUM_ION_TRANSMEMBRANE_TRANSPORT
GOBP_REGULATION_OF_CALCIUM_ION_TRANSMEMBRANE_TRANSPORT

GOBP_REGULATION_OF_CALCIUM_ION_TRANSMEMBRANE_TRANSPORT 138 0.519914283 1.772738968 0.001358696 0.030581767 0.024202852 1441 tags=20%, list=9%, signal=18%
CEMIP/CXCL9/CXCL11/CXCL10/KCNE3/LYN/PDE4B/GEM/JPH1/APLNR/F2R/RAMP3/PLN/CD19/FYN/THY1/SESTD1/PLCG2/DIAPH1/CORO1A/GNB5/CLIC2/SELENON/PIK3CG/GSTO1/PKD2/PRNP

GOBP_NEGATIVE_REGULATION_OF_IMMUNE_RESPONSE
GOBP_NEGATIVE_REGULATION_OF_IMMUNE_RESPONSE
GOBP_NEGATIVE_REGULATION_OF_IMMUNE_RESPONSE 139 0.56207536 1.915862682 0.001362398 0.030581767 0.024202852 3388 tags=41%, list=20%, signal=33%
MMP12/CD55/C4BPB/C4BPA/IL13RA2/ANXA1/SPINK5/SERPINB9/IL7R/IL33/COL3A1/LYN/SERPING1/CCR2/IFI16/JAK3/IL27RA/SLAMF8/PTPRC/SERPINB4/SAMSN1/FCGR2B/TRIM27/FOXF1/CD300A/NLRC5/SAMHD1/LYAR/BCL6/PARP14/A2M/CD84/METTL3/DUSP10/LILRB4/LILRB1/HAVCR2/NOD2/INPP5D/HLX/CD59/PSMA1/NMI/GPX1/ADAR/TYRO3/THOC1/ARRB2/TRIM21/IRAK3/CTLA4/RC3H2/SELENOS/TRAFD1/IL1RL1/IL2RA/CR1

GOBP_NEGATIVE_REGULATION_OF_LYMPHOCYTE_ACTIVATION
GOBP_NEGATIVE_REGULATION_OF_LYMPHOCYTE_ACTIVATION
GOBP_NEGATIVE_REGULATION_OF_LYMPHOCYTE_ACTIVATION 142 0.513726002 1.754862658 0.001362398 0.030581767 0.024202852 3388 tags=40%, list=20%, signal=32%
ANXA1/CD274/IDO1/MNDA/LYN/LAX1/CD74/JAK3/INHBA/CBFB/GAL/SAMSN1/FCGR2B/SHH/CEBPB/LILRB2/CD86/FGR/CD300A/TYROBP/LST1/BCL6/LAPTM5/BANK1/IRF1/RIPOR2/RUNX3/TWSG1/PRNP/BTK/LAG3/LILRB4/AXL/TMEM131L/SFRP1/LILRB1/GPNMB/HAVCR2/INPP5D/HLX/BLK/GLMN/MDK/LRRC32/TYRO3/THOC1/PRKAR1A/DLG5/BTN2A2/CTLA4/TBC1D10C/RC3H2/IL2RA/PLA2G2D/PKN1/HMGB1/CR1

GOBP_POSITIVE_REGULATION_OF_LEUKOCYTE_PROLIFERATION
GOBP_POSITIVE_REGULATION_OF_LEUKOCYTE_PROLIFERATION
GOBP_POSITIVE_REGULATION_OF_LEUKOCYTE_PROLIFERATION 141 0.557873097 1.904216667 0.00136612 0.030581767 0.024202852 2677 tags=38%, list=16%, signal=33%
CD55/ANXA1/CD274/IL1B/CD38/LYN/ADA/SLC7A1/CCR2/CD74/JAK3/IL27RA/PNP/PTPRC/HLA-DMB/RIPK2/VCAM1/TNFSF13B/TGFBR2/BST2/SHH/LILRB2/CD86/SASH3/CD81/CD40/HLA-DPA1/CORO1A/BCL6/IL1A/IGFBP2/IL6ST/CD276/CSF1R/IL12RB1/GPR183/CCL19/FCRL3/EBI3/MIF/IL23A/IL6/TNFRSF4/AIF1/HAVCR2/SLC39A10/SELENOK/NCKAP1L/CD320/CD4/SYK/CD6/RASAL3/IL18

GOBP_B_CELL_MEDIATED_IMMUNITY GOBP_B_CELL_MEDIATED_IMMUNITY
GOBP_B_CELL_MEDIATED_IMMUNITY 111 0.665193851 2.198624435 0.001371742 0.030581767 0.024202852 2303 tags=39%, list=14%, signal=34%
CD55/C4BPB/C4BPA/IL13RA2/CFI/C1S/C3/SERPING1/C2/C1R/CD74/TGFB1/HLA-DQB1/IL27RA/PTPRC/CLU/TCIRG1/FCER1G/TNFSF13/TLR8/CD19/MAD2L2/FCGR2B/C1QB/CD27/CD81/CD40/BCL6/C1QA/CCR6/EXOSC3/CR2/BTK/EXO1/MSH6/C1RL/MLH1/BATF/NBN/NOD2/INPP5D/IRF7/SWAP70

GOBP_REGULATION_OF_WOUND_HEALING GOBP_REGULATION_OF_WOUND_HEALING
GOBP_REGULATION_OF_WOUND_HEALING 121 0.554567481 1.855125928 0.001373626 0.030581767 0.024202852 736 tags=21%, list=4%, signal=20%

DUOX2/REG3A/GJA1/ANXA1/PLAU/CLDN1/CAV1/THBD/FERMT2/SERPINE2/ALOX5/SERPING1/F3/SERPINE1/TFPI/F2R/XBP1/ST3GAL4/CLEC7A/PDGFRA/SMOC2/CXCR4/TNFRSF12A/PLAT/FAP

GOBP_GRANULOCYTE_CHEMOTAXIS GOBP_GRANULOCYTE_CHEMOTAXIS GOBP_GRANULOCYTE_CHEMOTAXIS 114 0.63411483 2.098923659 0.00137931 0.030581767 0.024202852 1687 tags=40%, list=10%, signal=37% S100A8/CXCL5/CXCL1/CXCL3/CXCL6/CCL11/CXCL9/S100A9/ANXA1/CXCL11/CXCL13/CXCL8/CCL18/CXCL10/S100A12/CXCL2/CXCR2/CD74/PDE4B/PF4/RAC2/CCL20/S100A7/FCER1G/ADGRE2/C5AR1/CCR7/CSF3R/ITGB2/CCL4/CCL2/CCL24/CKLF/CCL22/VAV1/PIK3CG/C3AR1/RIPOR2/CSF1R/PPBP/CCL19/PIK3CD/JAM3/PPIB/IL23A/SRP54

GOBP_NEUTROPHIL_MIGRATION GOBP_NEUTROPHIL_MIGRATION GOBP_NEUTROPHIL_MIGRATION 113 0.620696031 2.053553567 0.00137931 0.030581767 0.024202852 1766 tags=42%, list=10%, signal=38% S100A8/CXCL5/CXCL1/CXCL3/CXCL6/CCL11/CXCL9/S100A9/CXCL11/CXCL13/CXCL8/CCL18/CXCL10/PECAM1/S100A12/CXCL2/CXCR2/CD74/PDE4B/PF4/RAC2/SLAMF8/CCL20/FCER1G/IL1R1/C5AR1/CCR7/CSF3R/ITGB2/CCL4/CCL2/CCL24/RHOH/CKLF/CCL22/VAV1/PIK3CG/C3AR1/RIPOR2/PPBP/CCL19/PIK3CD/JAM3/PPIB/IL23A/SRP54/ADAM8/RHOG

GOBP_LYMPHOCYTE_MIGRATION GOBP_LYMPHOCYTE_MIGRATION GOBP_LYMPHOCYTE_MIGRATION 104 0.573465898 1.875023687 0.001381215 0.030581767 0.024202852 2688 tags=43%, list=16%, signal=37% CCL11/CXCL11/CXCL13/CCL18/CXCL10/WNT5A/SPNS2/MSN/CCR2/IL27RA/ECM1/CCL20/S100A7/MADCAM1/CCR7/CCL4/ICAM1/CCL2/CCL24/CKLF/CCL22/JAM2/S1PR1/CH25H/CCR6/PIK3CG/LRCH1/RIPOR2/GPR183/CCL19/PIK3CD/CD200/CXCL14/ADAM8/STK10/AIF1/APOD/SELENOK/HSD3B7/CXCL16/ARTN/CCL7/AKT1/ITGA4/DOCK8

GOBP_REGULATION_OF_B_CELL_ACTIVATION GOBP_REGULATION_OF_B_CELL_ACTIVATION GOBP_REGULATION_OF_B_CELL_ACTIVATION 117 0.550732775 1.829162399 0.001381215 0.030581767 0.024202852 2366 tags=38%, list=14%, signal=33% MZB1/MNDA/CD38/LYN/ADA/CD74/TGFB1/INHBA/IL27RA/SLAMF8/PTPRC/XBP1/TNFSF13/TNFSF13B/CD19/SAMSN1/MAD2L2/FCGR2B/SASH3/CD27/CD300A/TYROBP/CD81/CD40/BCL6/LAPTM5/BANK1/GPR183/FCRL3/EXOSC3/MIF/BTK/IKZF3/AHR/IL6/TNFRSF4/SFRP1/SLC39A10/MLH1/NCKAP1L/NOD2/INPP5D/CD320/BLK

GOBP_ANTIMICROBIAL_HUMORAL_RESPONSE GOBP_ANTIMICROBIAL_HUMORAL_RESPONSE GOBP_ANTIMICROBIAL_HUMORAL_RESPONSE 124 0.741428565 2.485570878 0.001385042 0.030581767 0.024202852 486 tags=22%, list=3%, signal=21% REG1A/REG1B/REG3A/DEFA5/S100A8/LCN2/PI3/DEFA6/CXCL5/CXCL1/DMBT1/CXCL3/CXCL6/PLA2G2A/CXCL9/S100A9/CXCL11/CXCL13/CXCL8/CXCL10/SPINK5/S100A12/CXCL2/PF4/SLPI/CLU/S100A7

GOBP_POSITIVE_REGULATION_OF_INFLAMMATORY_RESPONSE GOBP_POSITIVE_REGULATION_OF_INFLAMMATORY_RESPONSE GOBP_POSITIVE_REGULATION_OF_INFLAMMATORY_RESPONSE 118 0.61358332 2.037854355 0.001386963 0.030581767 0.024202852 2755 tags=43%, list=16%, signal=36% S100A8/C2CD4A/PLA2G2A/S100A9/IDO1/IL1B/S100A12/AGT/WNT5A/TGM2/IL33/C3/ITGA2/SERPINE1/CCR2/PLA2G7/LPL/NFKBIZ/PTGS2/CCR7/JAK2/CEBPB/TLR2/ABCC1/HYAL2/FFAR2/CCL24/CD81/GPRC5B/IL6ST/CTSC/PIK3CG/GPSM3/BTK/CD47/IL23A/IL6/TSLP/ADAM8/IFNG/ZBP1/GP

R4/TNFSF11/IL16/LRRK2/NMI/MDK/ETS1/PARK7/IL18/PLA2G3
GOBP_SPROUTING_ANGIOGENESIS GOBP_SPROUTING_ANGIOGENESIS GOBP_SPROUTING_ANGIOGENESIS 118 0.546527589 1.815146519 0.001386963 0.030581767 0.024202852 2858 tags=38%, list=17%, signal=32% GREM1/ANXA1/LOXL2/ADAMTS9/ROBO1/ALOX5/PKM/PIK3R3/APLNR/RSPO3/PTGS2/ITGA5/SRPX2/RNF213/CREB3L1/MMRN2/VEGFC/KLF2/LEF1/KDR/TEK/ADGRA2/SPRED1/ITGB1BP1/CLEC14A/NRP1/HDAC7/PPP1R16B/EGR3/EPHB4/AKT3/FGFBP1/JMJD6/PLK2/RAMP2/PARVA/RHOJ/GLUL/FBXW7/AKT1/DLL1/JCAD/EFNB2/FGF2/JAK1
GOBP_LEUKOCYTE_MEDIATED_CYTOTOXICITY GOBP_LEUKOCYTE_MEDIATED_CYTOTOXICITY GOBP_LEUKOCYTE_MEDIATED_CYTOTOXICITY 101 0.571335481 1.859091553 0.001396648 0.030581767 0.024202852 3101 tags=40%, list=18%, signal=33% CXCL6/SERPINB9/CADM1/IL7R/SLAMF7/NOS2/CTSH/AZGP1/HLA-DRA/GZMB/PTPRC/SERPINB4/FCGR2B/ICAM1/TYROBP/CORO1A/ITGAM/VAV1/CTSC/LYST/IL12RB1/TUBB/LAG3/IL23A/MR1/LILRB1/CTSG/HAVCR2/SLAMF6/RNF19B/DNASE1L3/RASGRP1/IL18/HPRT1/PVR/ARRB2/AP1G1/STAT5B/EMP2/IL18RAP
GOBP_INTERLEUKIN_1_PRODUCTION GOBP_INTERLEUKIN_1_PRODUCTION GOBP_INTERLEUKIN_1_PRODUCTION 100 0.577191854 1.875194688 0.00140056 0.030581767 0.024202852 2341 tags=39%, list=14%, signal=34% ANXA1/WNT5A/MNDA/CASP1/IFI16/AIM2/LPL/S1PR3/F2R/RIPK2/CLEC7A/TLR8/CARD16/CCR7/JAK2/TRIM16/HK1/TYROBP/SIRPA/GBP5/STAT3/FFAR4/CCL19/SPHK1/S100A13/NLRP7/PML/CASP8/IL6/LILRB4/SERPINB1/NLRP2/IFNG/HAVCR2/P2RX7/NOD2/PANX1/MALT1/IL16
GOBP_POSITIVE_REGULATION_OF_T_CELL_PROLIFERATION GOBP_POSITIVE_REGULATION_OF_T_CELL_PROLIFERATION GOBP_POSITIVE_REGULATION_OF_T_CELL_PROLIFERATION 95 0.598505914 1.928106792 0.001404494 0.030581767 0.024202852 2677 tags=42%, list=16%, signal=36% CD55/ANXA1/CD274/IL1B/SLC7A1/CCR2/JAK3/IL27RA/PNP/PTPRC/HLA-DMB/RIPK2/VCAM1/TNFSF13B/TGFBR2/SHH/LILRB2/CD86/SASH3/CD81/HLA-DPA1/CORO1A/IL1A/IGFBP2/IL6ST/CD276/IL12RB1/CCL19/EBI3/IL23A/IL6/AIF1/HAVCR2/SELENOK/NCKAP1L/CD4/SYK/CD6/RASAL3/IL18
GOBP_RESPONSE_TO_TYPE_I_INTERFERON GOBP_RESPONSE_TO_TYPE_I_INTERFERON GOBP_RESPONSE_TO_TYPE_I_INTERFERON 95 0.580854321 1.871241594 0.001404494 0.030581767 0.024202852 2994 tags=43%, list=18%, signal=36% MMP12/WNT5A/IFITM2/IFIT3/IFITM3/ISG20/IFI6/IFITM1/IRF4/OAS2/BST2/STAT1/NLRC5/SAMHD1/IRAK1/SHMT2/IRF1/XAF1/OAS3/GBP2/IFNAR2/MX2/METTL3/ABCE1/USP18/IRF9/MX1/STAT2/ZBP1/SHFL/IRF7/CDC37/PTPN1/HSP90AB1/ADAR/PSMB8/TYK2/IRF3/JAK1/TBK1/PTPN11
GOBP_ACUTE_INFLAMMATORY_RESPONSE GOBP_ACUTE_INFLAMMATORY_RESPONSE GOBP_ACUTE_INFLAMMATORY_RESPONSE 99 0.624215461 2.021287368 0.00140647 0.030581767 0.024202852 1746 tags=29%, list=10%, signal=26% REG3A/S100A8/C2CD4A/VNN1/SERPINA3/IL1B/C3/TREM1/F3/SERPINA1/ASS1/PTGS2/FN1/CD163/VCAM1/CCR7/FCGR2B/CEBPB/ICAM1/FFAR2/IL1A/IL6ST/ANO6/PIK3CG/APOL2/BTK/ACVR1/IL6/ADAM8
GOBP_COLLAGEN_METABOLIC_PROCESS GOBP_COLLAGEN_METABOLIC_PROCESS GOBP_COLLAGEN_METABOLIC_PROCESS 99 0.655799185 2.123559398 0.00140647 0.030581767 0.024202852 2253 tags=40%, list=13%, signal=35%

MMP1/MMP3/MMP10/MMP12/COL15A1/CTSK/MMP7/COL1A2/COL1A1/MMP9/RGCC/MMP2/P3H2/ITGA2/ENG/SERPINB7/TGFB1/COL5A1/P3H1/F2R/HIF1A/VIM/FAP/PDGFRB/TRAM2/CREB3L1/EMILIN1/SERPINH1/CTSB/ADAM15/IL6/CTSL/PLOD3/MRC2/MMP25/WNT4/FURIN/MFAP4/KLK6/SERPINF2

GOBP_IMMUNOGLOBULIN_PRODUCTION GOBP_IMMUNOGLOBULIN_PRODUCTION

GOBP_IMMUNOGLOBULIN_PRODUCTION 99 0.582276308 1.885483167 0.00140647

0.030581767 0.024202852 3508 tags=43%, list=21%, signal=35%

IL13RA2/MZB1/IL33/TGFB1/HLA-DQB1/IL27RA/PTPRC/DNAJB9/XBP1/TNFSF13/MAD2L2/FCGR2B/CD86/SASH3/SAMHD1/CD40/BCL6/CCR6/TCF3/FCRL3/EXOSC3/BTK/VPREB3/EXO1/IL6/MSH6/TNFRSF4/MLH1/BATF/RBP4/NBN/MCM3AP/SWAP70/THOC1/CGAS/POLB/GPI/RNF8/HSPD1/SLC15A4/PKN1/CR1/CTNNBL1

GOBP_POSITIVE_REGULATION_OF_ADAPTIVE_IMMUNE_RESPONSE

GOBP_POSITIVE_REGULATION_OF_ADAPTIVE_IMMUNE_RESPONSE

GOBP_POSITIVE_REGULATION_OF_ADAPTIVE_IMMUNE_RESPONSE 97 0.566245457

1.826788391 0.001408451 0.030581767 0.024202852 2378 tags=32%, list=14%, signal=28%

CD274/IL1B/C3/ADA/CCR2/TGFB1/AZGP1/HLA-DRA/IL27RA/NFKBIZ/PTPRC/RIPK2/TNFSF13/IL1R1/TNFSF13B/MAD2L2/SASH3/CD81/CD40/IL6ST/IL12RB1/IL18R1/EXOSC3/BTK/IL23A/IL6/MR1/MLH1/NOD2/MALT1/CD4

GOBP_POSITIVE_REGULATION_OF_LYMPHOCYTE_DIFFERENTIATION

GOBP_POSITIVE_REGULATION_OF_LYMPHOCYTE_DIFFERENTIATION

GOBP_POSITIVE_REGULATION_OF_LYMPHOCYTE_DIFFERENTIATION 98 0.555234865

1.795176254 0.001408451 0.030581767 0.024202852 2523 tags=42%, list=15%, signal=36%

VNN1/ANXA1/IL7R/ADA/CD74/HLA-DRA/NFKBIZ/PNP/PTPRC/CBFB/RIPK2/XBP1/TGFBR2/SHH/LILRB2/CD86/SASH3/CD27/RHOH/BCL6/LEF1/IL12RB1/CCL19/DUSP10/RUNX3/BTK/ITPKB/EGR3/IL23A/ADAM8/LILRB4/AXL/IFNG/AP3D1/NCKAP1L/INPP5D/HLX/MALT1/ZBTB16/MDK/SYK

GOBP_REGULATION_OF_ALPHA_BETA_T_CELL_ACTIVATION

GOBP_REGULATION_OF_ALPHA_BETA_T_CELL_ACTIVATION

GOBP_REGULATION_OF_ALPHA_BETA_T_CELL_ACTIVATION 92 0.610195037

1.950903797 0.001420455 0.030581767 0.024202852 2677 tags=45%, list=16%, signal=38%

CD55/ANXA1/CD274/ADA/CCR2/JAK3/IRF4/HLA-DRA/NFKBIZ/PNP/PTPRC/CBFB/RIPK2/HSPH1/TGFBR2/SHH/CD86/SASH3/CD300A/CD81/BCL6/IRF1/IL12RB1/CCL19/RUNX3/TWSG1/EBI3/ITPKB/IL23A/LILRB4/LILRB1/IFNG/AP3D1/PRDM1/NCKAP1L/HLX/MALT1/ZBTB16/SYK/RASAL3/IL18

GOBP_ANTIGEN_PROCESSING_AND_PRESENTATION_OF_PEPTIDE_ANTIGEN_VIA_MHC_CLASS_I

GOBP_ANTIGEN_PROCESSING_AND_PRESENTATION_OF_PEPTIDE_ANTIGEN_VIA_MHC_CLASS_I

GOBP_ANTIGEN_PROCESSING_AND_PRESENTATION_OF_PEPTIDE_ANTIGEN_VIA_MHC_CLASS_I 93 0.645047894 2.061541157 0.001422475 0.030581767 0.024202852 4036

tags=65%, list=24%, signal=49%

NCF2/SEC24D/AZGP1/FCGR1B/FCER1G/PSME4/PSMB9/TAP2/CLEC4A/TAP1/SEC13/PSMD14/CALR/PSMD12/PSMC2/PSMB2/PSMA3/PSMD1/SEC24C/PDIA3/ITGAV/PSMA5/SEC24A/MR1/NCF4/PSMD6/PSMB5/PSMC4/SEC22B/PSMB1/PSMA1/ACE/CANX/SEC31A/PSME3/TAPBP/PSMA7/P

SMB8/PSMC5/CYBB/PSMB10/PSMB6/IDE/PSMA4/PSMD13/PSME1/PSMC1/CYBA/PSMB4/HLA-E/PSMB3/PSMD2/TAPBPL/IKBKG/PSMC6/PSMD7/PSMA2/MFSD6/ERAP1/PSMD4

GOBP_B_CELL_PROLIFERATION GOBP_B_CELL_PROLIFERATION GOBP_B_CELL_PROLIFERATION 93 0.587724804 1.878339397 0.001422475 0.030581767 0.024202852 2421 tags=39%, list=14%, signal=33% MZB1/MNDA/CTPS1/CD38/MS4A1/IL7R/LYN/ADA/CD74/PTPRC/TNFSF13B/CD19/FCGR2B/SASH3/CD300A/TYROBP/CD81/CD40/BCL6/LEF1/CD79A/GPR183/FCRL3/MIF/CR2/BTK/IKZF3/AHR/TNFRSF4/CD180/SLC39A10/NCKAP1L/INPP5D/CD320/BLK/RASGRP1

GOBP_NEUTROPHIL_CHEMOTAXISGOBP_NEUTROPHIL_CHEMOTAXIS GOBP_NEUTROPHIL_CHEMOTAXIS93 0.657650765 2.101819308 0.001422475 0.030581767 0.024202852 1687 tags=45%, list=10%, signal=41% S100A8/CXCL5/CXCL1/CXCL3/CXCL6/CCL11/CXCL9/S100A9/CXCL11/CXCL13/CXCL8/CCL18/CXCL10/S100A12/CXCL2/CXCR2/CD74/PDE4B/PF4/RAC2/CCL20/FCER1G/C5AR1/CCR7/CSF3R/ITGB2/CCL4/CCL2/CCL24/CKLF/CCL22/VAV1/PIK3CG/C3AR1/RIPOR2/PPBP/CCL19/PIK3CD/JAM3/PPIB/IL23A/SRP54

GOBP_RESPONSE_TO_CHEMOKINE GOBP_RESPONSE_TO_CHEMOKINE GOBP_RESPONSE_TO_CHEMOKINE 89 0.718846786 2.285047524 0.001426534 0.030581767 0.024202852 1293 tags=40%, list=8%, signal=38% REG1A/CXCL5/CXCL1/CXCL3/CXCL6/CCL11/CXCL9/CXCL11/CXCL13/CXCL8/CCL18/CXCL10/CXCL2/CXCR2/ROBO1/TFF2/CCR2/CCR10/PF4/CCL20/HIF1A/CCR1/ACKR4/CCR7/ACKR1/CCL4/CXCR4/LOX/CCL2/CCL24/CCL22/CCR6/LRCH1/RIPOR2/PPBP/CCL19

GOBP_REGULATION_OF_CELL_KILLING GOBP_REGULATION_OF_CELL_KILLING GOBP_REGULATION_OF_CELL_KILLING 91 0.559539116 1.780370923 0.001432665 0.030581767 0.024202852 2956 tags=40%, list=17%, signal=33% CD55/CXCL6/SERPINB9/CADM1/IL7R/NOS2/AZGP1/HLA-DRA/PTPRC/CLEC7A/SERPINB4/FCGR2B/ICAM1/TYROBP/ITGAM/VAV1/KRT6A/IL12RB1/LAG3/IL23A/CASP8/MR1/LILRB1/IFNG/HAVCR2/SLAMF6/P2RX7/CD59/DNASE1L3/RASGRP1/HSP90AB1/SYK/PVR/ARRB2/AP1G1/STAT5B

GOBP_ANAPHASE_PROMOTING_COMPLEX_DEPENDENT_CATABOLIC_PROCESS GOBP_ANAPHASE_PROMOTING_COMPLEX_DEPENDENT_CATABOLIC_PROCESS GOBP_ANAPHASE_PROMOTING_COMPLEX_DEPENDENT_CATABOLIC_PROCESS 78 0.632809285 1.976043602 0.001445087 0.030581767 0.024202852 4036 tags=62%, list=24%, signal=47% PSME4/PSMB9/CCNB1/CDC20/PSMD14/PTTG1/PSMD12/CDK1/PSMC2/PSMB2/AURKB/PSMA3/AURKA/PSMD1/BUB1B/PLK1/UBE2C/CDC27/PSMA5/UBE2S/CDK2/PSMD6/PSMB5/PSMC4/PSMB1/PSMA1/CDC26/PSME3/PSMA7/PSMB8/PSMC5/PSMB10/PSMB6/ANAPC7/PSMA4/FBXO5/PSMD13/PSME1/PSMC1/PSMB4/PSMB3/PSMD2/BUB3/PSMC6/MAD2L1/PSMD7/PSMA2/PSMD4

GOBP_INTERLEUKIN_1_BETA_PRODUCTION GOBP_INTERLEUKIN_1_BETA_PRODUCTION GOBP_INTERLEUKIN_1_BETA_PRODUCTION 86 0.585898594 1.850925045 0.001445087 0.030581767 0.024202852 2319 tags=41%, list=14%, signal=35% WNT5A/MNDA/CASP1/IFI16/AIM2/LPL/S1PR3/F2R/RIPK2/CLEC7A/TLR8/CARD16/CCR7/JAK2/TRIM16/HK1/TYROBP/SIRPA/GBP5/STAT3/FFAR4/CCL19/SPHK1/NLRP7/PML/CASP8/IL6/LILRB4/SERPINB1/NLRP2/IFNG/P2RX7/NOD2/PANX1/MALT1

GOBP_EXTRACELLULAR_MATRIX_DISASSEMBLY GOBP_EXTRACELLULAR_MATRIX_DISASSEMBLY GOBP_EXTRACELLULAR_MATRIX_DISASSEMBLY 76 0.677837851 2.098771063

0.001451379 0.030581767 0.024202852 2276 tags=39%, list=13%, signal=34%
MMP1/MMP3/MMP10/MMP12/TIMP1/CTSK/MMP7/MMP9/MMP2/LAMC1/HTRA1/CD44/TGFB1/PDPN/SH3PXD2B/FAP/DDR2/LCP1/A2M/FSCN1/ADAMTS5/ADAM15/IL6/ADAM8/CTSL/CTSG/TIMP2/TPSAB1/FURIN/KIF9
GOBP_REGULATION_OF_LEUKOCYTE_APOPTOTIC_PROCESS
GOBP_REGULATION_OF_LEUKOCYTE_APOPTOTIC_PROCESS
GOBP_REGULATION_OF_LEUKOCYTE_APOPTOTIC_PROCESS 76 0.614042559
1.90124342 0.001451379 0.030581767 0.024202852 2408 tags=39%, list=14%, signal=34%
ANXA1/CD274/IDO1/WNT5A/SLC7A11/IL7R/LYN/ADA/HCLS1/CD74/JAK3/HIF1A/CCR7/GIMAP8/CD27/BCL6/CCL19/AURKB/PIK3CD/BTK/ITPKB/ADAM8/AXL/LILRB1/SLC39A10/NOD2/TSC22D3/IRF7/BLK/NOC2L
GOBP_ANTIMICROBIAL_HUMORAL_IMMUNE_RESPONSE_MEDIATED_BY_ANTIMICROBIAL_PEPTIDE
GOBP_ANTIMICROBIAL_HUMORAL_IMMUNE_RESPONSE_MEDIATED_BY_ANTIMICROBIAL_PEPTIDE
GOBP_ANTIMICROBIAL_HUMORAL_IMMUNE_RESPONSE_MEDIATED_BY_ANTIMICROBIAL_PEPTIDE 70 0.790893735 2.432889963 0.001461988 0.030581767 0.024202852 1263
tags=36%, list=7%, signal=33%
REG1A/REG1B/REG3A/DEFA5/DEFA6/CXCL5/CXCL1/CXCL3/CXCL6/CXCL9/S100A9/CXCL11/CXCL13/CXCL8/CXCL10/SPINK5/S100A12/CXCL2/PF4/S100A7/RNASE6/LTF/SEMG1/KRT6A/PPBP
GOBP_REGULATION_OF_HUMORAL_IMMUNE_RESPONSE
GOBP_REGULATION_OF_HUMORAL_IMMUNE_RESPONSE
GOBP_REGULATION_OF_HUMORAL_IMMUNE_RESPONSE 70 0.742050554 2.282642111
0.001461988 0.030581767 0.024202852 1186 tags=36%, list=7%, signal=33%
CD55/C4BPB/C4BPA/CXCL13/CFB/SPINK5/IL1B/CFI/SPNS2/C1S/C3/SERPING1/C2/C1R/PTPRC/CLU/C5AR1/CCR7/CD19/FCGR2B/C1QB/CD81/C1QA/A2M/C3AR1
GOBP_AMINOGLYCAN_CATABOLIC_PROCESS GOBP_AMINOGLYCAN_CATABOLIC_PROCESS
GOBP_AMINOGLYCAN_CATABOLIC_PROCESS 67 0.618481839 1.888512259
0.001466276 0.030581767 0.024202852 1983 tags=28%, list=12%, signal=25%
CHI3L1/CEMIP/HYAL1/CD44/TGFB1/BGN/CHI3L2/LUM/VCAN/DCN/HYAL2/SDC2/GPC6/FMOD/HPSE/HGSNAT/SDC3/AGRN/LYVE1
GOBP_COMPLEMENT_ACTIVATION GOBP_COMPLEMENT_ACTIVATION
GOBP_COMPLEMENT_ACTIVATION 64 0.741505154 2.251195175 0.001466276
0.030581767 0.024202852 1806 tags=39%, list=11%, signal=35%
CD55/C4BPB/C4BPA/CFB/IL1B/CFI/RGCC/C1S/C3/SERPING1/C2/C1R/CLU/C5AR1/FCN1/CD19/C1QB/CD81/FCN3/C1QA/A2M/C3AR1/CR2/CFP/C1RL
GOBP_KILLING_OF_CELLS_OF_OTHER_ORGANISM
GOBP_KILLING_OF_CELLS_OF_OTHER_ORGANISM
GOBP_KILLING_OF_CELLS_OF_OTHER_ORGANISM 56 0.659101368 1.970484433
0.001472754 0.030581767 0.024202852 2122 tags=30%, list=13%, signal=27%
DEFA5/DEFA6/CXCL6/S100A12/SERPINB9/NOS2/PF4/CLEC7A/APOL1/LTF/SEMG1/KRT6A/LYZ/CASP8/IFNG/CTSG/P2RX7
GOBP_B_CELL_RECEPTOR_SIGNALING_PATHWAY

GOBP_B_CELL_RECEPTOR_SIGNALING_PATHWAY
GOBP_B_CELL_RECEPTOR_SIGNALING_PATHWAY 57 0.608696493 1.821654261 0.001474926 0.030581767 0.024202852 2523 tags=50%, list=15%, signal=43% MNDA/CD38/MS4A1/LYN/RFTN1/KLHL6/PTPRC/CBFB/CD19/FCGR2B/CD300A/PLCG2/CD81/CD79B/CD79A/LCK/PRKCB/PIK3CD/FCRL3/SH2B2/BTK/PRKCH/ITK/LPXN/SLC39A10/NCKAP1L/BLK/LAT2/SYK

GOBP_INTERLEUKIN_2_PRODUCTION GOBP_INTERLEUKIN_2_PRODUCTION GOBP_INTERLEUKIN_2_PRODUCTION 58 0.61176593 1.834210104 0.001474926 0.030581767 0.024202852 2480 tags=41%, list=15%, signal=35% ANXA1/IL1B/GBP1/CCR2/PDE4B/IRF4/PNP/PTPRC/RIPK2/CLEC7A/CD86/TRIM27/SASH3/IL1A/LAPTM5/PRNP/HDAC7/LAG3/LILRB4/HAVCR2/NOD2/MALT1/CD4/GLMN

GOBP_MONOCYTE_CHEMOTAXIS GOBP_MONOCYTE_CHEMOTAXIS GOBP_MONOCYTE_CHEMOTAXIS 58 0.650166944 1.949344871 0.001474926 0.030581767 0.024202852 2102 tags=43%, list=12%, signal=38% GREM1/CCL11/ANXA1/CCL18/CXCL10/S100A12/LYN/SERPINE1/CCR2/PLA2G7/SLAMF8/CCL20/S100A7/CCR1/CCL4/CCL2/CCL24/CCL22/ANO6/CCL19/IL6/CREB3/AIF1/FPR2/TNFSF11

GOBP_POSITIVE_REGULATION_OF_INTERLEUKIN_1_PRODUCTION GOBP_POSITIVE_REGULATION_OF_INTERLEUKIN_1_PRODUCTION GOBP_POSITIVE_REGULATION_OF_INTERLEUKIN_1_PRODUCTION 57 0.611191824 1.829122071 0.001474926 0.030581767 0.024202852 2341 tags=44%, list=14%, signal=38% WNT5A/MNDA/CASP1/IFI16/AIM2/LPL/RIPK2/CLEC7A/TLR8/JAK2/TRIM16/HK1/TYROBP/STAT3/CCL19/CASP8/IL6/NLRP2/IFNG/HAVCR2/P2RX7/NOD2/PANX1/MALT1/IL16

GOBP_REGULATION_OF_CELLULAR_AMINO_ACID_METABOLIC_PROCESS GOBP_REGULATION_OF_CELLULAR_AMINO_ACID_METABOLIC_PROCESS GOBP_REGULATION_OF_CELLULAR_AMINO_ACID_METABOLIC_PROCESS 58 0.603082172 1.808174268 0.001474926 0.030581767 0.024202852 4368 tags=66%, list=26%, signal=49% SLC7A11/PSME4/PSMB9/ODC1/PSMD14/PSMD12/PSMC2/PSMB2/PSMA3/PSMD1/PSMA5/OAZ3/PSMD6/ATP2B4/PSMB5/PSMC4/PSMB1/PSMA1/PSME3/PARK7/PSMA7/PSMB8/PSMC5/PSMB10/PSMB6/PSMA4/PSMD13/PSME1/PSMC1/PSMB4/PSMB3/PSMD2/PSMC6/PSMD7/PSMA2/PSMD4/PSMD9/SLC7A7

GOBP_POSITIVE_REGULATION_OF_ALPHA_BETA_T_CELL_ACTIVATION GOBP_POSITIVE_REGULATION_OF_ALPHA_BETA_T_CELL_ACTIVATION GOBP_POSITIVE_REGULATION_OF_ALPHA_BETA_T_CELL_ACTIVATION 62 0.652095369 1.963451646 0.001477105 0.030581767 0.024202852 2677 tags=52%, list=16%, signal=44% CD55/ANXA1/ADA/CCR2/HLA-DRA/NFKBIZ/PNP/PTPRC/CBFB/RIPK2/HSPH1/TGFBR2/SHH/CD86/SASH3/CD81/IL12RB1/CCL19/RUNX3/EBI3/ITPKB/IL23A/LILRB4/IFNG/AP3D1/NCKAP1L/HLX/MALT1/ZBTB16/SYK/RASAL3/IL18

GOBP_ANTIGEN_PROCESSING_AND_PRESENTATION_OF_EXOGENOUS_PEPTIDE_ANTIGEN_VIA_MHC_CLASS_I GOBP_ANTIGEN_PROCESSING_AND_PRESENTATION_OF_EXOGENOUS_PEPTIDE_ANTIGEN_VIA_MHC_CLASS_I

GOBP_ANTIGEN_PROCESSING_AND_PRESENTATION_OF_EXOGENOUS_PEPTIDE_ANTIGEN_VIA_MHC_CLASS_I 74 0.666272962 2.055550516 0.00147929 0.030581767 0.024202852 4036 tags=65%, list=24%, signal=50% NCF2/FCGR1B/FCER1G/PSME4/PSMB9/TAP2/CLEC4A/TAP1/PSMD14/CALR/PSMD12/PSMC2/PSMB2/PSMA3/PSMD1/PDIA3/ITGAV/PSMA5/NCF4/PSMD6/PSMB5/PSMC4/SEC22B/PSMB1/PSMA1/PSME3/TAPBP/PSMA7/PSMB8/PSMC5/CYBB/PSMB10/PSMB6/PSMA4/PSMD13/PSME1/PSMC1/CYBA/PSMB4/HLA-E/PSMB3/PSMD2/IKBKG/PSMC6/PSMD7/PSMA2/MFSD6/PSMD4

GOBP_INFLAMMATORY_RESPONSE_TO_ANTIGENIC_STIMULUS GOBP_INFLAMMATORY_RESPONSE_TO_ANTIGENIC_STIMULUS GOBP_INFLAMMATORY_RESPONSE_TO_ANTIGENIC_STIMULUS 54 0.623185396 1.85343975 0.001481481 0.030581767 0.024202852 3705 tags=41%, list=22%, signal=32% IL1RN/C3/CCR7/FCGR2B/ICAM1/CD81/PNMA1/BTK/CYSLTR1/NOD2/PSMA1/GPX1/CD6/PARK7/AHCY/SELENOS/IL2RA/PLA2G2D/HMGB1/PSMB4/HLA-E/KDM6B

GOBP_POSITIVE_REGULATION_OF_WOUND_HEALING GOBP_POSITIVE_REGULATION_OF_WOUND_HEALING GOBP_POSITIVE_REGULATION_OF_WOUND_HEALING54 0.650893598 1.935847783 0.001481481 0.030581767 0.024202852 657 tags=26%, list=4%, signal=25% DUOX2/REG3A/ANXA1/CLDN1/THBD/FERMT2/F3/SERPINE1/F2R/XBP1/ST3GAL4/CLEC7A/SMOC2/CXCR4

GOBP_SYNCYTIUM_FORMATION GOBP_SYNCYTIUM_FORMATION GOBP_SYNCYTIUM_FORMATION 53 0.594795374 1.762230113 0.00148368 0.030581767 0.024202852 1720 tags=26%, list=10%, signal=24% CXCL9/CXCL10/CD53/SBNO2/TYROBP/CD81/JAM2/PLEKHO1/RIPOR2/ADAM9/MYOF/CFLAR/CD109/EHD2

GOBP_NEGATIVE_REGULATION_OF_LEUKOCYTE_MEDIATED_IMMUNITY GOBP_NEGATIVE_REGULATION_OF_LEUKOCYTE_MEDIATED_IMMUNITY GOBP_NEGATIVE_REGULATION_OF_LEUKOCYTE_MEDIATED_IMMUNITY 52 0.619009966 1.826823601 0.001485884 0.030581767 0.024202852 2123 tags=37%, list=13%, signal=32% C4BPB/C4BPA/IL13RA2/SERPINB9/IL7R/CCR2/JAK3/PTPRC/SERPINB4/BST2/FCGR2B/FOXF1/CD300A/BCL6/CD84/LILRB4/LILRB1/HAVCR2/NOD2

GOBP_REGULATION_OF_LYMPHOCYTE_APOPTOTIC_PROCESS GOBP_REGULATION_OF_LYMPHOCYTE_APOPTOTIC_PROCESS GOBP_REGULATION_OF_LYMPHOCYTE_APOPTOTIC_PROCESS 52 0.599713171 1.769874856 0.001485884 0.030581767 0.024202852 2408 tags=37%, list=14%, signal=31% CD274/IDO1/WNT5A/IL7R/LYN/ADA/CD74/JAK3/HIF1A/GIMAP8/CD27/BCL6/AURKB/BTK/ADAM8/SLC39A10/TSC22D3/BLK/NOC2L

GOBP_ANTIBACTERIAL_HUMORAL_RESPONSE GOBP_ANTIBACTERIAL_HUMORAL_RESPONSE GOBP_ANTIBACTERIAL_HUMORAL_RESPONSE 51 0.613286777 1.800268768 0.001501502 0.030581767 0.024202852 1085 tags=18%, list=6%, signal=17% DEFA5/PI3/DEFA6/DMBT1/SPINK5/SLPI/RNASE6/LTF/SEMG1

GOBP_COLLAGEN_FIBRIL_ORGANIZATION GOBP_COLLAGEN_FIBRIL_ORGANIZATION

GOBP_COLLAGEN_FIBRIL_ORGANIZATION 51 0.717514701 2.106223965 0.001501502 0.030581767 0.024202852 2478 tags=51%, list=15%, signal=44% GREM1/LOXL2/COL12A1/COL1A2/COL1A1/COL5A2/PXDN/COL3A1/LOXL1/COL5A1/LUM/AEBP1/EFEMP2/LOX/DDR2/EMILIN1/SERPINH1/COL14A1/FMOD/CRTAP/PLOD3/TGFBR1/SFRP2/SERPINF2/COL5A3/COLGALT1

GOBP_LYMPHOCYTE_CHEMOTAXIS GOBP_LYMPHOCYTE_CHEMOTAXIS GOBP_LYMPHOCYTE_CHEMOTAXIS 51 0.615731683 1.807445652 0.001501502 0.030581767 0.024202852 2198 tags=45%, list=13%, signal=39% CCL11/CXCL11/CXCL13/CCL18/CXCL10/WNT5A/CCR2/CCL20/S100A7/CCL4/CCL2/CCL24/CKLF/CCL22/CH25H/PIK3CG/GPR183/CCL19/PIK3CD/CXCL14/ADAM8/HSD3B7/CXCL16

GOBP_POSITIVE_REGULATION_OF_RESPONSE_TO_CYTOKINE_STIMULUS GOBP_POSITIVE_REGULATION_OF_RESPONSE_TO_CYTOKINE_STIMULUS GOBP_POSITIVE_REGULATION_OF_RESPONSE_TO_CYTOKINE_STIMULUS 51 0.61388445 1.802023203 0.001501502 0.030581767 0.024202852 2237 tags=37%, list=13%, signal=32% MMP12/WNT5A/CASP1/CD74/RIPK2/HIF1A/IL1R1/PARP9/CXCR4/TLR2/NLRC5/CASP4/PARP14/LAPTM5/TSLP/CD300LF/AXL/ZBP1/IRF7

GOBP_HYDROGEN_PEROXIDE_METABOLIC_PROCESS GOBP_HYDROGEN_PEROXIDE_METABOLIC_PROCESS GOBP_HYDROGEN_PEROXIDE_METABOLIC_PROCESS 50 0.609222381 1.783831298 0.001517451 0.030581767 0.024202852 620 tags=16%, list=4%, signal=15% MMP3/DUOX2/DUOXA2/PXDN/HBB/PRDX4/RAC2/FYN

GOBP_PYRIMIDINE_CONTAINING_COMPOUND_BIOSYNTHETIC_PROCESS GOBP_PYRIMIDINE_CONTAINING_COMPOUND_BIOSYNTHETIC_PROCESS GOBP_PYRIMIDINE_CONTAINING_COMPOUND_BIOSYNTHETIC_PROCESS 36 0.687229245 1.90145081 0.001536098 0.030581767 0.024202852 2060 tags=42%, list=12%, signal=37% CTPS1/TPK1/NME1/TYMP/PRPS1/NME5/UPP1/DTYMK/TK1/DCTD/TYMS/UMPS/NME4/NME7/CAD

GOBP_POSITIVE_REGULATION_OF_INTERLEUKIN_12_PRODUCTION GOBP_POSITIVE_REGULATION_OF_INTERLEUKIN_12_PRODUCTION GOBP_POSITIVE_REGULATION_OF_INTERLEUKIN_12_PRODUCTION 39 0.608163891 1.70983953 0.001538462 0.030581767 0.024202852 2523 tags=46%, list=15%, signal=39% IDO1/RIPK2/CLEC7A/CCR7/TLR2/CD40/LAPTM5/IRF1/PLCB1/CCL19/IL23A/IFNG/RELA/LTB/NOD2/IL16/MDK/SYK

GOBP_T_HELPER_1_TYPE_IMMUNE_RESPONSE GOBP_T_HELPER_1_TYPE_IMMUNE_RESPONSE GOBP_T_HELPER_1_TYPE_IMMUNE_RESPONSE 39 0.639522481 1.798003521 0.001538462 0.030581767 0.024202852 2193 tags=46%, list=13%, signal=40% ANXA1/IL1B/IL33/CCR2/JAK3/IL27RA/RIPK2/IL1R1/LEF1/IL12RB1/CCL19/IL18R1/IL18BP/EBI3/IL23A/HAVCR2/HLX/SEMA4A

GOBP_CHONDROITIN_SULFATE_PROTEOGLYCAN_METABOLIC_PROCESS GOBP_CHONDROITIN_SULFATE_PROTEOGLYCAN_METABOLIC_PROCESS GOBP_CHONDROITIN_SULFATE_PROTEOGLYCAN_METABOLIC_PROCESS 42 0.633168948

1.804334196 0.001540832 0.030581767 0.024202852 1702 tags=33%, list=10%, signal=30%
CSGALNACT1/CHST15/HYAL1/BGN/CHST11/CHSY1/VCAN/DSE/DCN/CHPF/B3GALT6/CSGALNACT2/SPOCK2/CHST12
GOBP_ENDODERMAL_CELL_DIFFERENTIATION GOBP_ENDODERMAL_CELL_DIFFERENTIATION GOBP_ENDODERMAL_CELL_DIFFERENTIATION 42 0.650835396 1.854678068 0.001540832 0.030581767 0.024202852 1602 tags=40%, list=9%, signal=37% COL12A1/MMP9/COL5A2/MMP2/COL6A1/LAMA3/COL4A2/INHBA/COL5A1/FN1/ITGA5/ITGB2/COL8A1/DKK1/LAMB1/COL7A1/ITGAV
GOBP_HUMORAL_IMMUNE_RESPONSE_MEDIATED_BY_CIRCULATING_IMMUNOGLOBULIN GOBP_HUMORAL_IMMUNE_RESPONSE_MEDIATED_BY_CIRCULATING_IMMUNOGLOBULIN GOBP_HUMORAL_IMMUNE_RESPONSE_MEDIATED_BY_CIRCULATING_IMMUNOGLOBULIN 43 0.760322008 2.17676425 0.001540832 0.030581767 0.024202852 1806 tags=44%, list=11%, signal=40% CD55/C4BPB/C4BPA/CFI/C1S/C3/SERPING1/C2/C1R/HLA-DQB1/PTPRC/CLU/FCGR2B/C1QB/CD81/C1QA/CR2/EXO1/C1RL
GOBP_RESPONSE_TO_AMYLOID_BETA GOBP_RESPONSE_TO_AMYLOID_BETA GOBP_RESPONSE_TO_AMYLOID_BETA 43 0.641343498 1.836134668 0.001540832 0.030581767 0.024202852 881 tags=28%, list=5%, signal=27% MMP3/MMP12/GJA1/MMP9/MMP2/RAMP3/VCAM1/FYN/FCGR2B/ICAM1/ABCC1/CASP4
GOBP_ACUTE_PHASE_RESPONSE GOBP_ACUTE_PHASE_RESPONSE GOBP_ACUTE_PHASE_RESPONSE 41 0.675117995 1.914526431 0.001545595 0.030581767 0.024202852 2253 tags=34%, list=13%, signal=30% REG3A/SERPINA3/IL1B/SERPINA1/ASS1/PTGS2/FN1/CD163/CEBPB/IL1A/APOL2/IL6/TNFSF11/SERPINF2
GOBP_CELLULAR_DEFENSE_RESPONSE GOBP_CELLULAR_DEFENSE_RESPONSE GOBP_CELLULAR_DEFENSE_RESPONSE 49 0.661601715 1.924881532 0.001545595 0.030581767 0.024202852 2073 tags=41%, list=12%, signal=36% CXCL9/NCF2/MNDA/CXCR2/IL33/CCR2/LY96/TCIRG1/C5AR1/LILRB2/TYROBP/RAB23/CCR6/ITK/FCMR/LSP1/ADORA2B/CCR5/CLEC5A/RELA
GOBP_NEGATIVE_REGULATION_OF_EPITHELIAL_CELL_DIFFERENTIATION GOBP_NEGATIVE_REGULATION_OF_EPITHELIAL_CELL_DIFFERENTIATION GOBP_NEGATIVE_REGULATION_OF_EPITHELIAL_CELL_DIFFERENTIATION 41 0.648590195 1.839297841 0.001545595 0.030581767 0.024202852 1892 tags=29%, list=11%, signal=26% REG3A/MMP9/CAV1/S1PR3/ZEB1/STAT1/XDH/EZH2/SPRED1/CCND1/IFNG/JAG1
GOBP_POSITIVE_REGULATION_OF_EPITHELIAL_TO_MESENCHYMAL_TRANSITION GOBP_POSITIVE_REGULATION_OF_EPITHELIAL_TO_MESENCHYMAL_TRANSITION GOBP_POSITIVE_REGULATION_OF_EPITHELIAL_TO_MESENCHYMAL_TRANSITION 48 0.612218146 1.776920957 0.001550388 0.030581767 0.024202852 2506 tags=50%, list=15%, signal=43% LOXL2/IL1B/COL1A1/RGCC/SERPINB3/FERMT2/ENG/TGFB1/PDPN/TWIST1/TGFBR2/TGFB1I1/LEF1/DAB2/WWTR1/EZH2/OLFM1/ACVR1/SDCBP/IL6/JAG1/TGFBR1/TGFB3/MDK
GOBP_POSITIVE_REGULATION_OF_MUSCLE_CONTRACTION GOBP_POSITIVE_REGULATION_OF_MUSCLE_CONTRACTION

GOBP_POSITIVE_REGULATION_OF_MUSCLE_CONTRACTION 37 0.621609539 1.726572889 0.001550388 0.030581767 0.024202852 1354 tags=35%, list=8%, signal=32%
PROK2/ITGA2/ADA/F2R/KIT/PTAFR/MYOCD/RGS2/ENO1/NMU/SPHK1/GSTO1/CHRM3
GOBP_REGULATION_OF_COMPLEMENT_ACTIVATION
GOBP_REGULATION_OF_COMPLEMENT_ACTIVATION
GOBP_REGULATION_OF_COMPLEMENT_ACTIVATION 48 0.770191595 2.235428002 0.001550388 0.030581767 0.024202852 1741 tags=44%, list=10%, signal=39%
CD55/C4BPB/C4BPA/CFB/IL1B/CFI/C1S/C3/SERPING1/C2/C1R/CLU/C5AR1/CD19/C1QB/CD81/C1QA/A2M/C3AR1/CR2/CFP
GOBP_REGULATION_OF_CALCIUM_ION_IMPORT
GOBP_REGULATION_OF_CALCIUM_ION_IMPORT
GOBP_REGULATION_OF_CALCIUM_ION_IMPORT 38 0.640430468 1.787029908 0.001552795 0.030581767 0.024202852 1520 tags=37%, list=9%, signal=34%
STC1/RAMP3/HOMER1/PLN/FYN/TRIM27/CCL2/PDGFRB/SEMG1/SPINK1/FCRL3/PKD2/PRNP/TRPV2
GOBP_LEUKOCYTE_ADHESION_TO_VASCULAR_ENDOTHELIAL_CELL
GOBP_LEUKOCYTE_ADHESION_TO_VASCULAR_ENDOTHELIAL_CELL
GOBP_LEUKOCYTE_ADHESION_TO_VASCULAR_ENDOTHELIAL_CELL 44 0.609933844 1.746871036 0.001560062 0.030581767 0.024202852 2843 tags=45%, list=17%, signal=38%
SELL/ALOX5/SELP/ST3GAL4/VCAM1/MADCAM1/ITGB2/SELE/ICAM1/PTAFR/IRAK1/JAM2/SELPLG/IL6/FUT4/RELA/MDK/ETS1/ITGA4/ROCK1
GOBP_NEGATIVE_REGULATION_OF_B_CELL_ACTIVATION
GOBP_NEGATIVE_REGULATION_OF_B_CELL_ACTIVATION
GOBP_NEGATIVE_REGULATION_OF_B_CELL_ACTIVATION 33 0.633559816 1.732676705 0.001560062 0.030581767 0.024202852 3388 tags=58%, list=20%, signal=46%
MNDA/LYN/INHBA/SAMSN1/FCGR2B/CD300A/TYROBP/BCL6/LAPTM5/BANK1/BTK/SFRP1/INPP5D/BLK/THOC1/CTLA4/TBC1D10C/PKN1/CR1
GOBP_NEUROINFLAMMATORY_RESPONSE GOBP_NEUROINFLAMMATORY_RESPONSE
GOBP_NEUROINFLAMMATORY_RESPONSE 33 0.715586663 1.957005967 0.001560062 0.030581767 0.024202852 2342 tags=45%, list=14%, signal=39%
MMP3/IL1B/MMP9/IL33/PTPRC/PTGS2/CST7/CTSC/SPHK1/TNFRSF1B/CD200/IL6/IFNG/SYT11/LRRK2
GOBP_PEPTIDE_CROSS_LINKING GOBP_PEPTIDE_CROSS_LINKING
GOBP_PEPTIDE_CROSS_LINKING 33 0.638748512 1.746866892 0.001560062 0.030581767 0.024202852 1331 tags=30%, list=8%, signal=28%
PI3/ANXA1/TGM2/COL3A1/BGN/FN1/DCN/CSTA/SPRR1B/SPOCK2
GOBP_REGULATION_OF_CELLULAR_EXTRAVASATION
GOBP_REGULATION_OF_CELLULAR_EXTRAVASATION
GOBP_REGULATION_OF_CELLULAR_EXTRAVASATION 33 0.64841352 1.77329902 0.001560062 0.030581767 0.024202852 1779 tags=45%, list=11%, signal=41%
PLVAP/SELP/CCR2/IL27RA/ST3GAL4/IL1R1/THY1/SELE/ICAM1/PTAFR/RIPOR2/PLCB1/JAM3/ADAM8/FUT4

GOBP_REGULATION_OF_PLATELET_ACTIVATION GOBP_REGULATION_OF_PLATELET_ACTIVATION GOBP_REGULATION_OF_PLATELET_ACTIVATION 33 0.632033038 1.728501231 0.001560062 0.030581767 0.024202852 638 tags=24%, list=4%, signal=23% THBD/SERPINE2/LYN/SELP/PDPN/FCER1G/PDGFRA/PLEK

GOBP_REGULATION_OF_T_CELL_APOPTOTIC_PROCESS GOBP_REGULATION_OF_T_CELL_APOPTOTIC_PROCESS GOBP_REGULATION_OF_T_CELL_APOPTOTIC_PROCESS 33 0.625644128 1.711028664 0.001560062 0.030581767 0.024202852 740 tags=27%, list=4%, signal=26% CD274/IDO1/WNT5A/IL7R/ADA/JAK3/HIF1A/GIMAP8/CD27

GOBP_REGULATION_OF_TYPE_I_INTERFERON_MEDIATED_SIGNALING_PATHWAY GOBP_REGULATION_OF_TYPE_I_INTERFERON_MEDIATED_SIGNALING_PATHWAY GOBP_REGULATION_OF_TYPE_I_INTERFERON_MEDIATED_SIGNALING_PATHWAY 33 0.621301306 1.699151797 0.001560062 0.030581767 0.024202852 2994 tags=52%, list=18%, signal=42% MMP12/WNT5A/NLRC5/SAMHD1/IFNAR2/METTL3/ABCE1/USP18/ZBP1/IRF7/CDC37/PTPN1/HSP90AB1/ADAR/IRF3/TBK1/PTPN11

GOBP_RESPONSE_TO_FUNGUS GOBP_RESPONSE_TO_FUNGUS GOBP_RESPONSE_TO_FUNGUS 44 0.686594852 1.966430739 0.001560062 0.030581767 0.024202852 988 tags=18%, list=6%, signal=17% DEFA5/S100A8/DEFA6/S100A9/S100A12/CLEC7A/COTL1/LTF

GOBP_DEFENSE_RESPONSE_TO_FUNGUS GOBP_DEFENSE_RESPONSE_TO_FUNGUS GOBP_DEFENSE_RESPONSE_TO_FUNGUS 32 0.7630524 2.084425745 0.0015625 0.030581767 0.024202852 988 tags=22%, list=6%, signal=21% DEFA5/S100A8/DEFA6/S100A9/S100A12/COTL1/LTF

GOBP_EPIBOLY GOBP_EPIBOLY GOBP_EPIBOLY 32 0.665244539 1.817244588 0.0015625 0.030581767 0.024202852 2025 tags=34%, list=12%, signal=30% MMP12/FERMT2/CCN1/CD44/COL5A1/PDPN/ITGA5/FLNA/AJUBA/ARHGAP24/PHLDB2

GOBP_NUCLEOBASE_METABOLIC_PROCESS GOBP_NUCLEOBASE_METABOLIC_PROCESS GOBP_NUCLEOBASE_METABOLIC_PROCESS 32 0.615902941 1.682458436 0.0015625 0.030581767 0.024202852 2694 tags=50%, list=16%, signal=42% CTPS1/ADA/PPAT/TYMP/SHMT2/GART/PRPS1/XDH/GMPR/TYMS/UMPS/GMPS/CAD/RRM1/PAICS/HPRT1

GOBP_TYPE_2_IMMUNE_RESPONSE GOBP_TYPE_2_IMMUNE_RESPONSE GOBP_TYPE_2_IMMUNE_RESPONSE 35 0.639049182 1.757729214 0.001564945 0.030581767 0.024202852 2185 tags=40%, list=13%, signal=35% ANXA1/IDO1/IL33/CCR2/CD74/IL27RA/ECM1/CD86/CD81/BCL6/IL6/BATF/NOD2/HLX

GOBP_VASCULAR_ENDOTHELIAL_GROWTH_FACTOR_PRODUCTION GOBP_VASCULAR_ENDOTHELIAL_GROWTH_FACTOR_PRODUCTION GOBP_VASCULAR_ENDOTHELIAL_GROWTH_FACTOR_PRODUCTION 35 0.652824801 1.795619582 0.001564945 0.030581767 0.024202852 1721 tags=43%, list=10%, signal=39% IL1B/C3/CCR2/TGFB1/SULF1/HIF1A/PTGS2/C5AR1/SULF2/IL1A/IL6ST/C3AR1/RORA/HPSE/IL6

GOBP_COLLAGEN_CATABOLIC_PROCESS GOBP_COLLAGEN_CATABOLIC_PROCESS

GOBP_COLLAGEN_CATABOLIC_PROCESS 46 0.689800336 1.986042568 0.001567398 0.030581767 0.024202852 2247 tags=37%, list=13%, signal=32% MMP1/MMP3/MMP10/MMP12/COL15A1/CTSK/MMP7/MMP9/MMP2/FAP/CTSB/ADAM15/CTSL/MRC2/MMP25/FURIN/KLK6

GOBP_MODULATION_BY_SYMBIONT_OF_ENTRY_INTO_HOST GOBP_MODULATION_BY_SYMBIONT_OF_ENTRY_INTO_HOST GOBP_MODULATION_BY_SYMBIONT_OF_ENTRY_INTO_HOST 46 0.622283166 1.791650123 0.001567398 0.030581767 0.024202852 1602 tags=33%, list=9%, signal=30% CXCL8/IFITM2/CAV1/IFITM3/TRIM22/CD74/IFITM1/LGALS1/FCN1/TRIM27/FCN3/P4HB/KRT6A/LY6E/ITGAV

GOBP_POSITIVE_REGULATION_OF_ALPHA_BETA_T_CELL_DIFFERENTIATION GOBP_POSITIVE_REGULATION_OF_ALPHA_BETA_T_CELL_DIFFERENTIATION GOBP_POSITIVE_REGULATION_OF_ALPHA_BETA_T_CELL_DIFFERENTIATION 46 0.685938164 1.974922773 0.001567398 0.030581767 0.024202852 2677 tags=54%, list=16%, signal=46% ANXA1/ADA/HLA-DRA/NFKBIZ/PNP/CBFB/RIPK2/TGFBR2/SHH/CD86/SASH3/IL12RB1/CCL19/RUNX3/ITPKB/IL23A/LILRB4/IFNG/AP3D1/NCKAP1L/HLX/MALT1/ZBTB16/SYK/IL18

GOBP_ALPHA_BETA_T_CELL_PROLIFERATION GOBP_ALPHA_BETA_T_CELL_PROLIFERATION GOBP_ALPHA_BETA_T_CELL_PROLIFERATION 34 0.631650444 1.732441735 0.001569859 0.030581767 0.024202852 2677 tags=44%, list=16%, signal=37% CD55/CD274/CCR2/PTPRC/RIPK2/TGFBR2/CD81/IRF1/DOCK2/TWSG1/EBI3/IL23A/SYK/RASAL3/IL18

GOBP_CHONDROITIN_SULFATE_PROTEOGLYCAN_BIOSYNTHETIC_PROCESS GOBP_CHONDROITIN_SULFATE_PROTEOGLYCAN_BIOSYNTHETIC_PROCESS GOBP_CHONDROITIN_SULFATE_PROTEOGLYCAN_BIOSYNTHETIC_PROCESS 30 0.662997077 1.779849261 0.001569859 0.030581767 0.024202852 1043 tags=37%, list=6%, signal=34% CSGALNACT1/CHST15/BGN/CHST11/CHSY1/VCAN/DSE/DCN/CHPF/B3GALT6/CSGALNACT2

GOBP_INTERLEUKIN_4_PRODUCTION GOBP_INTERLEUKIN_4_PRODUCTION GOBP_INTERLEUKIN_4_PRODUCTION 30 0.646830604 1.736449543 0.001569859 0.030581767 0.024202852 947 tags=27%, list=6%, signal=25% SLC7A5/IL33/IRF4/FCER1G/CEBPB/CD86/SASH3/LEF1

GOBP_MATURE_B_CELL_DIFFERENTIATION GOBP_MATURE_B_CELL_DIFFERENTIATION GOBP_MATURE_B_CELL_DIFFERENTIATION 30 0.65058337 1.746524034 0.001569859 0.030581767 0.024202852 1757 tags=50%, list=10%, signal=45% POU2AF1/ADA/LGALS1/SLAMF8/XBP1/CD19/FCGR2B/PLCG2/CMTM7/MFNG/DOCK11/GPR183/NKX2-3/IL6/ITM2A

GOBP_POSITIVE_REGULATION_OF_CD4_POSITIVE_ALPHA_BETA_T_CELL_DIFFERENTIATION GOBP_POSITIVE_REGULATION_OF_CD4_POSITIVE_ALPHA_BETA_T_CELL_DIFFERENTIATION GOBP_POSITIVE_REGULATION_OF_CD4_POSITIVE_ALPHA_BETA_T_CELL_DIFFERENTIATION 30 0.657501569 1.76509629 0.001569859 0.030581767 0.024202852 2677 tags=47%, list=16%, signal=39% ANXA1/HLA-DRA/NFKBIZ/RIPK2/CD86/SASH3/IL12RB1/CCL19/IL23A/IFNG/NCKAP1L/HLX/M

ALT1/IL18
GOBP_POSITIVE_REGULATION_OF_VASOCONSTRICTION
GOBP_POSITIVE_REGULATION_OF_VASOCONSTRICTION
GOBP_POSITIVE_REGULATION_OF_VASOCONSTRICTION 30 0.639461858 1.716667771 0.001569859 0.030581767 0.024202852 743 tags=20%, list=4%, signal=19% GJA1/CD38/CAV1/F2R/ICAM1/PTAFR
GOBP_REGULATION_OF_TYPE_2_IMMUNE_RESPONSE
GOBP_REGULATION_OF_TYPE_2_IMMUNE_RESPONSE
GOBP_REGULATION_OF_TYPE_2_IMMUNE_RESPONSE 30 0.661473693 1.775759659 0.001569859 0.030581767 0.024202852 2185 tags=43%, list=13%, signal=38% ANXA1/IDO1/IL33/CCR2/CD74/IL27RA/ECM1/CD86/CD81/BCL6/IL6/NOD2/HLX
GOBP_DENDRITIC_CELL_MIGRATION GOBP_DENDRITIC_CELL_MIGRATION
GOBP_DENDRITIC_CELL_MIGRATION 29 0.66682826 1.782013738 0.001572327 0.030581767 0.024202852 1293 tags=45%, list=8%, signal=41% CXCR2/ALOX5/CCR2/SLAMF8/CCR1/CCR7/CXCR4/CALR/CCR6/ANO6/PIK3CG/GPR183/CCL19
GOBP_GLYCOLYTIC_PROCESS_THROUGH_FRUCTOSE_6_PHOSPHATE
GOBP_GLYCOLYTIC_PROCESS_THROUGH_FRUCTOSE_6_PHOSPHATE
GOBP_GLYCOLYTIC_PROCESS_THROUGH_FRUCTOSE_6_PHOSPHATE 29 0.643909956 1.720767485 0.001572327 0.030581767 0.024202852 3341 tags=55%, list=20%, signal=44%
ALDOB/PKM/PFKP/HK1/ENO1/ENO2/HK2/PGAM1/HK3/PGM2L1/GALK1/ADPGK/FOXK2/GPI/ENO3/PGK1
GOBP_POSITIVE_REGULATION_OF_INTERLEUKIN_2_PRODUCTION
GOBP_POSITIVE_REGULATION_OF_INTERLEUKIN_2_PRODUCTION
GOBP_POSITIVE_REGULATION_OF_INTERLEUKIN_2_PRODUCTION 29 0.720681354 1.925929285 0.001572327 0.030581767 0.024202852 941 tags=38%, list=6%, signal=36% ANXA1/IL1B/CCR2/PDE4B/IRF4/PTPRC/RIPK2/CLEC7A/CD86/SASH3/IL1A
GOBP_RESPONSE_TO_INTERFERON_BETA GOBP_RESPONSE_TO_INTERFERON_BETA
GOBP_RESPONSE_TO_INTERFERON_BETA 29 0.668599934 1.786748311 0.001572327 0.030581767 0.024202852 1220 tags=41%, list=7%, signal=38% MNDA/IFITM2/IFITM3/IFI16/IFITM1/AIM2/STING1/BST2/STAT1/IRF1/XAF1/IFNAR2
GOBP_RESPIRATORY_BURST GOBP_RESPIRATORY_BURST GOBP_RESPIRATORY_BURST 31 0.651613648 1.762846506 0.001574803 0.030581767 0.024202852 2271 tags=45%, list=13%, signal=39% CD55/NCF2/RAC2/SLAMF8/CLEC7A/HCK/PIK3CG/PIK3CD/DUSP10/CYBC1/NCF4/PGAM1/SELENOK/CD52
GOBP_SIGNALING_RECEPTOR_LIGAND_PRECURSOR_PROCESSING
GOBP_SIGNALING_RECEPTOR_LIGAND_PRECURSOR_PROCESSING
GOBP_SIGNALING_RECEPTOR_LIGAND_PRECURSOR_PROCESSING 31 0.679156081 1.837358577 0.001574803 0.030581767 0.024202852 2339 tags=39%, list=14%, signal=33% PCSK1/BACE2/CASP1/CPA3/MME/ECE2/CTSL/AOPEP/CTSG/SCG5/FURIN/ACE
GOBP_AROMATIC_AMINO_ACID_FAMILY_CATABOLIC_PROCESS
GOBP_AROMATIC_AMINO_ACID_FAMILY_CATABOLIC_PROCESS
GOBP_AROMATIC_AMINO_ACID_FAMILY_CATABOLIC_PROCESS 19 0.758523827

1.86829622 0.001589825 0.030581767 0.024202852 1668 tags=37%, list=10%, signal=33% IDO1/KYNU/TDO2/ASRGL1/KMO/IL4I1/HGD
GOBP_CHRONIC_INFLAMMATORY_RESPONSE GOBP_CHRONIC_INFLAMMATORY_RESPONSE GOBP_CHRONIC_INFLAMMATORY_RESPONSE 19 0.77614912 1.911708527 0.001589825 0.030581767 0.024202852 545 tags=42%, list=3%, signal=41% S100A8/CCL11/VNN1/GJA1/S100A9/CXCL13/IDO1/VCAM1
GOBP_DENDRITIC_CELL_CHEMOTAXIS GOBP_DENDRITIC_CELL_CHEMOTAXIS GOBP_DENDRITIC_CELL_CHEMOTAXIS 24 0.702636406 1.803903125 0.001589825 0.030581767 0.024202852 1293 tags=50%, list=8%, signal=46% CXCR2/CCR2/SLAMF8/CCR1/CCR7/CXCR4/CALR/CCR6/ANO6/PIK3CG/GPR183/CCL19
GOBP_NEGATIVE_REGULATION_OF_INTERLEUKIN_10_PRODUCTION GOBP_NEGATIVE_REGULATION_OF_INTERLEUKIN_10_PRODUCTION GOBP_NEGATIVE_REGULATION_OF_INTERLEUKIN_10_PRODUCTION 19 0.757600974 1.86602317 0.001589825 0.030581767 0.024202852 1832 tags=47%, list=11%, signal=42% CD274/IDO1/TRIB2/JAK3/FCGR2B/TYROBP/IL23A/LILRB4/LILRB1
GOBP_POSITIVE_REGULATION_OF_ACUTE_INFLAMMATORY_RESPONSE GOBP_POSITIVE_REGULATION_OF_ACUTE_INFLAMMATORY_RESPONSE GOBP_POSITIVE_REGULATION_OF_ACUTE_INFLAMMATORY_RESPONSE 24 0.739759051 1.899209396 0.001589825 0.030581767 0.024202852 2102 tags=50%, list=12%, signal=44% C2CD4A/IL1B/C3/PTGS2/CCR7/FFAR2/IL6ST/PIK3CG/BTK/IL6/ADAM8/TNFSF11
GOBP_POSITIVE_REGULATION_OF_HUMORAL_IMMUNE_RESPONSE GOBP_POSITIVE_REGULATION_OF_HUMORAL_IMMUNE_RESPONSE GOBP_POSITIVE_REGULATION_OF_HUMORAL_IMMUNE_RESPONSE 19 0.732330448 1.803780132 0.001589825 0.030581767 0.024202852 625 tags=26%, list=4%, signal=25% IL1B/C3/PTPRC/CCR7/FCGR2B
GOBP_PYRIMIDINE_RIBONUCLEOTIDE_BIOSYNTHETIC_PROCESS GOBP_PYRIMIDINE_RIBONUCLEOTIDE_BIOSYNTHETIC_PROCESS GOBP_PYRIMIDINE_RIBONUCLEOTIDE_BIOSYNTHETIC_PROCESS19 0.738119815 1.818039739 0.001589825 0.030581767 0.024202852 2060 tags=42%, list=12%, signal=37% CTPS1/NME1/NME5/UPP1/UMPS/NME4/NME7/CAD
GOBP_ENDOPLASMIC_RETICULUM_TO_CYTOSOL_TRANSPORT GOBP_ENDOPLASMIC_RETICULUM_TO_CYTOSOL_TRANSPORT GOBP_ENDOPLASMIC_RETICULUM_TO_CYTOSOL_TRANSPORT 27 0.678116298 1.788433345 0.001594896 0.030581767 0.024202852 3650 tags=67%, list=22%, signal=52% DERL3/HM13/ERLEC1/VCP/UBE2J1/SEC61B/HSP90B1/DERL2/EDEM1/DERL1/SEL1L/UFD1/SELENOS/HERPUD1/EDEM2/UBE2G2/FAF2/RHBDD1
GOBP_LEUKOCYTE_MIGRATION_INVOLVED_IN_INFLAMMATORY_RESPONSE GOBP_LEUKOCYTE_MIGRATION_INVOLVED_IN_INFLAMMATORY_RESPONSE GOBP_LEUKOCYTE_MIGRATION_INVOLVED_IN_INFLAMMATORY_RESPONSE 18 0.791827748 1.920564544 0.001594896 0.030581767 0.024202852 1746 tags=56%, list=10%, signal=50% S100A8/S100A9/ALOX5/SLAMF8/ITGB2/SELE/FFAR2/CCR6/JAM3/ADAM8
GOBP_NEGATIVE_REGULATION_OF_TISSUE_REMODELING

GOBP_NEGATIVE_REGULATION_OF_TISSUE_REMODELING
GOBP_NEGATIVE_REGULATION_OF_TISSUE_REMODELING 20 0.727667976 1.808861611 0.001594896 0.030581767 0.024202852 2126 tags=50%, list=13%, signal=44% GREM1/CD38/TNFRSF11B/GPR137B/CLDN18/IL6/SFRP1/TMEM119/P2RX7/INPP5D

GOBP_POSITIVE_REGULATION_OF_FATTY_ACID_BIOSYNTHETIC_PROCESS
GOBP_POSITIVE_REGULATION_OF_FATTY_ACID_BIOSYNTHETIC_PROCESS
GOBP_POSITIVE_REGULATION_OF_FATTY_ACID_BIOSYNTHETIC_PROCESS 21 0.750810439 1.880132069 0.001594896 0.030581767 0.024202852 1232 tags=33%, list=7%, signal=31% ANXA1/ELOVL5/IL1B/LPGAT1/CD74/PTGS2/MID1IP1

GOBP_POSITIVE_REGULATION_OF_LEUKOCYTE_ADHESION_TO_VASCULAR_ENDOTHELIAL_CELL
GOBP_POSITIVE_REGULATION_OF_LEUKOCYTE_ADHESION_TO_VASCULAR_ENDOTHELIAL_CELL
GOBP_POSITIVE_REGULATION_OF_LEUKOCYTE_ADHESION_TO_VASCULAR_ENDOTHELIAL_CELL 20 0.786819027 1.955901289 0.001594896 0.030581767 0.024202852 2645 tags=70%, list=16%, signal=59% ALOX5/SELP/ST3GAL4/ITGB2/SELE/ICAM1/PTAFR/IRAK1/IL6/FUT4/RELA/MDK/ETS1/ITGA4

GOBP_POSITIVE_REGULATION_OF_VASCULAR_ENDOTHELIAL_GROWTH_FACTOR_PRODUCTION
GOBP_POSITIVE_REGULATION_OF_VASCULAR_ENDOTHELIAL_GROWTH_FACTOR_PRODUCTION
GOBP_POSITIVE_REGULATION_OF_VASCULAR_ENDOTHELIAL_GROWTH_FACTOR_PRODUCTION 27 0.745936419 1.967299074 0.001594896 0.030581767 0.024202852 1721 tags=52%, list=10%, signal=47% IL1B/C3/TGFB1/SULF1/HIF1A/PTGS2/C5AR1/SULF2/IL1A/IL6ST/C3AR1/RORA/HPSE/IL6

GOBP_REGULATION_OF_HYDROGEN_PEROXIDE_METABOLIC_PROCESS
GOBP_REGULATION_OF_HYDROGEN_PEROXIDE_METABOLIC_PROCESS
GOBP_REGULATION_OF_HYDROGEN_PEROXIDE_METABOLIC_PROCESS 18 0.767631491 1.861876941 0.001594896 0.030581767 0.024202852 620 tags=22%, list=4%, signal=21% MMP3/DUOXA2/RAC2/FYN

GOBP_REGULATION_OF_LEUKOCYTE_ADHESION_TO_VASCULAR_ENDOTHELIAL_CELL
GOBP_REGULATION_OF_LEUKOCYTE_ADHESION_TO_VASCULAR_ENDOTHELIAL_CELL
GOBP_REGULATION_OF_LEUKOCYTE_ADHESION_TO_VASCULAR_ENDOTHELIAL_CELL 27 0.659808112 1.74014816 0.001594896 0.030581767 0.024202852 2645 tags=52%, list=16%, signal=44% ALOX5/SELP/ST3GAL4/ITGB2/SELE/ICAM1/PTAFR/IRAK1/IL6/FUT4/RELA/MDK/ETS1/ITGA4

GOBP_REGULATION_OF_MONOCYTE_CHEMOTAXIS
GOBP_REGULATION_OF_MONOCYTE_CHEMOTAXIS
GOBP_REGULATION_OF_MONOCYTE_CHEMOTAXIS 27 0.681155189 1.796447978 0.001594896 0.030581767 0.024202852 1898 tags=48%, list=11%, signal=43% GREM1/CXCL10/LYN/SERPINE1/CCR2/PLA2G7/SLAMF8/S100A7/CCR1/ANO6/CREB3/AIF1/FPR2

GOBP_REGULATION_OF_T_HELPER_1_TYPE_IMMUNE_RESPONSE
GOBP_REGULATION_OF_T_HELPER_1_TYPE_IMMUNE_RESPONSE
GOBP_REGULATION_OF_T_HELPER_1_TYPE_IMMUNE_RESPONSE 27 0.699643896 1.845209262 0.001594896 0.030581767 0.024202852 2185 tags=52%, list=13%,

signal=45%
ANXA1/IL1B/IL33/CCR2/JAK3/IL27RA/RIPK2/IL1R1/IL12RB1/CCL19/IL18R1/IL23A/HAVCR2/HLX
GOBP_RESPONSE_TO_INTERFERON_ALPHA GOBP_RESPONSE_TO_INTERFERON_ALPHA GOBP_RESPONSE_TO_INTERFERON_ALPHA 21 0.733545729 1.836898874 0.001594896 0.030581767 0.024202852 1774 tags=43%, list=10%, signal=38% LAMP3/IFITM2/IFIT3/IFITM3/IFITM1/BST2/IFNAR2/MX2/AXL
GOBP_THYROID_HORMONE_GENERATION GOBP_THYROID_HORMONE_GENERATION GOBP_THYROID_HORMONE_GENERATION 21 0.732403783 1.834039285 0.001594896 0.030581767 0.024202852 107 tags=14%, list=1%, signal=14% DUOX2/DUOXA2/CTSK
GOBP_PHAGOCYTOSIS_RECOGNITION GOBP_PHAGOCYTOSIS_RECOGNITION GOBP_PHAGOCYTOSIS_RECOGNITION 22 0.711223401 1.801024011 0.001602564 0.030581767 0.024202852 2024 tags=36%, list=12%, signal=32% C4BPB/C4BPA/CLEC7A/FCN1/FCN3/MFGE8/PEAR1/JMJD6
GOBP_CLEAVAGE_INVOLVED_IN_RRNA_PROCESSING GOBP_CLEAVAGE_INVOLVED_IN_RRNA_PROCESSING GOBP_CLEAVAGE_INVOLVED_IN_RRNA_PROCESSING 26 0.664857639 1.735261442 0.001605136 0.030581767 0.024202852 3721 tags=65%, list=22%, signal=51% ERI1/RRP36/TSR1/RRS1/EXOSC3/RPP40/NOP14/EXOSC7/TBL3/EXOSC9/NHP2/BMS1/EXOSC8/NOL9/NOB1/EXOSC10/NOP9
GOBP_POSITIVE_REGULATION_OF_INTERLEUKIN_4_PRODUCTION GOBP_POSITIVE_REGULATION_OF_INTERLEUKIN_4_PRODUCTION GOBP_POSITIVE_REGULATION_OF_INTERLEUKIN_4_PRODUCTION 23 0.694773864 1.772158088 0.001605136 0.030581767 0.024202852 712 tags=30%, list=4%, signal=29% SLC7A5/IL33/IRF4/FCER1G/CEBPB/CD86/SASH3
GOBP_PYRIMIDINE_NUCLEOTIDE_BIOSYNTHETIC_PROCESS GOBP_PYRIMIDINE_NUCLEOTIDE_BIOSYNTHETIC_PROCESS GOBP_PYRIMIDINE_NUCLEOTIDE_BIOSYNTHETIC_PROCESS26 0.717665965 1.873089823 0.001605136 0.030581767 0.024202852 2060 tags=46%, list=12%, signal=41% CTPS1/NME1/PRPS1/NME5/UPP1/DTYMK/DCTD/TYMS/UMPS/NME4/NME7/CAD
GOBP_REGULATION_OF_INFLAMMATORY_RESPONSE_TO_ANTIGENIC_STIMULUS GOBP_REGULATION_OF_INFLAMMATORY_RESPONSE_TO_ANTIGENIC_STIMULUS GOBP_REGULATION_OF_INFLAMMATORY_RESPONSE_TO_ANTIGENIC_STIMULUS 26 0.670104209 1.748954856 0.001605136 0.030581767 0.024202852 3688 tags=50%, list=22%, signal=39% C3/CCR7/FCGR2B/CD81/BTK/NOD2/PSMA1/GPX1/PARK7/SELENOS/PLA2G2D/PSMB4/HLA-E
GOBP_POSITIVE_REGULATION_OF_MACROPHAGE_DERIVED_FOAM_CELL_DIFFERENTIATION GOBP_POSITIVE_REGULATION_OF_MACROPHAGE_DERIVED_FOAM_CELL_DIFFERENTIATION GOBP_POSITIVE_REGULATION_OF_MACROPHAGE_DERIVED_FOAM_CELL_DIFFERENTIATION 17 0.722211198 1.715124438 0.001626016 0.030581767 0.024202852 2755 tags=41%, list=16%, signal=35% PLA2G2A/AGT/PF4/LPL/PRKCH/IL18/PLA2G3
GOBP_VASCULAR_WOUND_HEALING GOBP_VASCULAR_WOUND_HEALING GOBP_VASCULAR_WOUND_HEALING 17 0.724090827 1.719588227 0.001626016

0.030581767 0.024202852 1498 tags=47%, list=9%, signal=43% MCAM/ALOX5/SERPINE1/XBP1/SMOC2/CXCR4/KDR/HPSE
GOBP_WOUND_HEALING_SPREADING_OF_EPIDERMAL_CELLS GOBP_WOUND_HEALING_SPREADING_OF_EPIDERMAL_CELLS GOBP_WOUND_HEALING_SPREADING_OF_EPIDERMAL_CELLS 17 0.744146431 1.767216754 0.001626016 0.030581767 0.024202852 2465 tags=47%, list=15%, signal=40% MMP12/FERMT2/COL5A1/ITGA5/AJUBA/ARHGAP24/PHLDB2/FERMT1
GOBP_CORTICOSTEROID_HORMONE_SECRETION GOBP_CORTICOSTEROID_HORMONE_SECRETION GOBP_CORTICOSTEROID_HORMONE_SECRETION 16 0.709201295 1.670255155 0.001636661 0.030581767 0.024202852 2994 tags=44%, list=18%, signal=36% AGT/SELENOM/GAL/BMP6/C1QTNF1/CRY1/PTPN11
GOBP_DEOXYRIBONUCLEOSIDE_MONOPHOSPHATE_METABOLIC_PROCESS GOBP_DEOXYRIBONUCLEOSIDE_MONOPHOSPHATE_METABOLIC_PROCESS GOBP_DEOXYRIBONUCLEOSIDE_MONOPHOSPHATE_METABOLIC_PROCESS 16 0.73103992 1.721687768 0.001636661 0.030581767 0.024202852 1404 tags=44%, list=8%, signal=40% ADA/TYMP/XDH/UPP1/TK1/DCTD/TYMS
GOBP_DERMATAN_SULFATE_PROTEOGLYCAN_METABOLIC_PROCESS GOBP_DERMATAN_SULFATE_PROTEOGLYCAN_METABOLIC_PROCESS GOBP_DERMATAN_SULFATE_PROTEOGLYCAN_METABOLIC_PROCESS 16 0.778317713 1.833032711 0.001636661 0.030581767 0.024202852 1702 tags=44%, list=10%, signal=39% CSGALNACT1/BGN/VCAN/DSE/DCN/CSGALNACT2/CHST12
GOBP_ESTABLISHMENT_OF_PROTEIN_LOCALIZATION_TO_TELOMERE GOBP_ESTABLISHMENT_OF_PROTEIN_LOCALIZATION_TO_TELOMERE GOBP_ESTABLISHMENT_OF_PROTEIN_LOCALIZATION_TO_TELOMERE 16 0.716850324 1.688269548 0.001636661 0.030581767 0.024202852 2598 tags=62%, list=15%, signal=53% ACD/ATR/CCT3/CCT8/CCT5/CCT2/CCT6A/CCT4/CCT7/NABP2
GOBP_MULTI_ORGANISM_CELLULAR_PROCESS GOBP_MULTI_ORGANISM_CELLULAR_PROCESS GOBP_MULTI_ORGANISM_CELLULAR_PROCESS 16 0.70763332 1.666562383 0.001636661 0.030581767 0.024202852 2378 tags=44%, list=14%, signal=38% CAV1/CXCR4/CAV2/HYAL2/CTSL/CCR5/CD4
GOBP_NEGATIVE_REGULATION_OF_BONE_RESORPTION GOBP_NEGATIVE_REGULATION_OF_BONE_RESORPTION GOBP_NEGATIVE_REGULATION_OF_BONE_RESORPTION 16 0.709778426 1.671614369 0.001636661 0.030581767 0.024202852 2126 tags=50%, list=13%, signal=44% CD38/TNFRSF11B/GPR137B/CLDN18/IL6/TMEM119/P2RX7/INPP5D
GOBP_OSTEOCLAST_DEVELOPMENT GOBP_OSTEOCLAST_DEVELOPMENT GOBP_OSTEOCLAST_DEVELOPMENT 16 0.700864701 1.650621465 0.001636661 0.030581767 0.024202852 2549 tags=56%, list=15%, signal=48% FBN1/TYROBP/LTF/CLDN18/SLC9B2/ANXA2/LILRB1/TNFSF11/FBXW7
GOBP_POSITIVE_REGULATION_OF_CELLULAR_EXTRAVASATION GOBP_POSITIVE_REGULATION_OF_CELLULAR_EXTRAVASATION GOBP_POSITIVE_REGULATION_OF_CELLULAR_EXTRAVASATION 16 0.733009693 1.726326824 0.001636661 0.030581767 0.024202852 1746 tags=56%, list=10%,

signal=50% PLVAP/CCR2/IL1R1/THY1/ICAM1/PTAFR/RIPOR2/JAM3/ADAM8
GOBP_PYRIMIDINE_NUCLEOSIDE_TRIPHOSPHATE_BIOSYNTHETIC_PROCESS GOBP_PYRIMIDINE_NUCLEOSIDE_TRIPHOSPHATE_BIOSYNTHETIC_PROCESS GOBP_PYRIMIDINE_NUCLEOSIDE_TRIPHOSPHATE_BIOSYNTHETIC_PROCESS 16 0.742997916 1.749850302 0.001636661 0.030581767 0.024202852 2060 tags=50%, list=12%, signal=44% CTPS1/NME1/NME5/DTYMK/TYMS/NME4/NME7/CAD
GOBP_PODOSOME_ASSEMBLY GOBP_PODOSOME_ASSEMBLY GOBP_PODOSOME_ASSEMBLY 15 0.754598719 1.7611589 0.001652893 0.030581767 0.024202852 2276 tags=47%, list=13%, signal=40% MSN/ASAP1/SH3PXD2B/HCK/LCP1/FSCN1/KIF9
GOBP_POSITIVE_REGULATION_OF_NITRIC_OXIDE_SYNTHASE_BIOSYNTHETIC_PROCESS GOBP_POSITIVE_REGULATION_OF_NITRIC_OXIDE_SYNTHASE_BIOSYNTHETIC_PROCESS GOBP_POSITIVE_REGULATION_OF_NITRIC_OXIDE_SYNTHASE_BIOSYNTHETIC_PROCESS 15 0.780398699 1.821373505 0.001652893 0.030581767 0.024202852 2342 tags=67%, list=14%, signal=57% IL33/JAK2/TLR2/CCL2/KDR/NAMPT/AKAP12/IFNG/NOD2/LRRK2
GOBP_REGULATION_OF_EXTRACELLULAR_MATRIX_ASSEMBLY GOBP_REGULATION_OF_EXTRACELLULAR_MATRIX_ASSEMBLY GOBP_REGULATION_OF_EXTRACELLULAR_MATRIX_ASSEMBLY 15 0.737774545 1.721892938 0.001652893 0.030581767 0.024202852 986 tags=40%, list=6%, signal=38% AGT/RGCC/TGFB1/ANTXR1/TIE1/EMILIN1
GOBP_CELL_ADHESION_MOLECULE_PRODUCTION GOBP_CELL_ADHESION_MOLECULE_PRODUCTION GOBP_CELL_ADHESION_MOLECULE_PRODUCTION 13 0.767519701 1.712570617 0.001666667 0.030581767 0.024202852 814 tags=31%, list=5%, signal=29% CXCL8/IL1B/CAV1/MYOCD
GOBP_COLLAGEN_ACTIVATED_SIGNALING_PATHWAY GOBP_COLLAGEN_ACTIVATED_SIGNALING_PATHWAY GOBP_COLLAGEN_ACTIVATED_SIGNALING_PATHWAY 13 0.773987493 1.727002234 0.001666667 0.030581767 0.024202852 2523 tags=54%, list=15%, signal=46% COL4A1/COL1A1/ITGA2/COL4A2/DDR2/COL4A5/SYK
GOBP_GAP_JUNCTION_ASSEMBLYGOBP_GAP_JUNCTION_ASSEMBLY GOBP_GAP_JUNCTION_ASSEMBLY13 0.770861829 1.720027926 0.001666667 0.030581767 0.024202852 166 tags=31%, list=1%, signal=30% GJA1/IL1B/AGT/CAV1
GOBP_LEUKOCYTE_AGGREGATION GOBP_LEUKOCYTE_AGGREGATION GOBP_LEUKOCYTE_AGGREGATION 13 0.837359716 1.868404999 0.001666667 0.030581767 0.024202852 994 tags=62%, list=6%, signal=58% S100A8/S100A9/IL1B/MSN/CD44/RAC2/SEMA4D/JAM2
GOBP_MIDGUT_DEVELOPMENT GOBP_MIDGUT_DEVELOPMENT GOBP_MIDGUT_DEVELOPMENT 13 0.790451671 1.76373884 0.001666667 0.030581767 0.024202852 710 tags=31%, list=4%, signal=30% REG1A/WNT5A/ASS1/FOXF1
GOBP_NEGATIVE_REGULATION_OF_CARDIAC_MUSCLE_CELL_PROLIFERATION GOBP_NEGATIVE_REGULATION_OF_CARDIAC_MUSCLE_CELL_PROLIFERATION

GOBP_NEGATIVE_REGULATION_OF_CARDIAC_MUSCLE_CELL_PROLIFERATION 13 0.756085476 1.687057372 0.001666667 0.030581767 0.024202852 2089 tags=31%, list=12%, signal=27% GJA1/TGFBR2/MEIS1/RBP4
GOBP_NEGATIVE_REGULATION_OF_HUMORAL_IMMUNE_RESPONSE GOBP_NEGATIVE_REGULATION_OF_HUMORAL_IMMUNE_RESPONSE GOBP_NEGATIVE_REGULATION_OF_HUMORAL_IMMUNE_RESPONSE 13 0.852096428 1.9012871 0.001666667 0.030581767 0.024202852 1075 tags=54%, list=6%, signal=50% CD55/C4BPB/C4BPA/SPINK5/SERPING1/FCGR2B/A2M
GOBP_NEUTROPHIL_HOMEOSTASIS GOBP_NEUTROPHIL_HOMEOSTASIS GOBP_NEUTROPHIL_HOMEOSTASIS 13 0.798292877 1.781234962 0.001666667 0.030581767 0.024202852 1774 tags=62%, list=10%, signal=55% ANXA1/SLC7A11/PDE4B/PIK3CD/JAM3/ITPKB/IL6/AXL
GOBP_REGULATION_OF_HUMORAL_IMMUNE_RESPONSE_MEDIATED_BY_CIRCULATING_IMMUNOGLOBULIN GOBP_REGULATION_OF_HUMORAL_IMMUNE_RESPONSE_MEDIATED_BY_CIRCULATING_IMMUNOGLOBULIN GOBP_REGULATION_OF_HUMORAL_IMMUNE_RESPONSE_MEDIATED_BY_CIRCULATING_IMMUNOGLOBULIN 13 0.7870134 1.756067009 0.001666667 0.030581767 0.024202852 625 tags=31%, list=4%, signal=30% C4BPB/C4BPA/PTPRC/FCGR2B
GOBP_RESPONSE_TO_UV_A GOBP_RESPONSE_TO_UV_A GOBP_RESPONSE_TO_UV_A 13 0.797958743 1.780489406 0.001666667 0.030581767 0.024202852 212 tags=38%, list=1%, signal=38% MMP1/MMP3/TIMP1/MMP9/MMP2
GOBP_CELLULAR_RESPONSE_TO_UV_A GOBP_CELLULAR_RESPONSE_TO_UV_A GOBP_CELLULAR_RESPONSE_TO_UV_A 10 0.855677635 1.82097443 0.001694915 0.030581767 0.024202852 212 tags=50%, list=1%, signal=49% MMP1/MMP3/TIMP1/MMP9/MMP2
GOBP_PROSTATIC_BUD_FORMATION GOBP_PROSTATIC_BUD_FORMATION GOBP_PROSTATIC_BUD_FORMATION 10 0.844021717 1.796169377 0.001694915 0.030581767 0.024202852 642 tags=30%, list=4%, signal=29% WNT5A/SULF1/SHH
GOBP_SURFACTANT_HOMEOSTASIS GOBP_SURFACTANT_HOMEOSTASIS GOBP_SURFACTANT_HOMEOSTASIS 10 0.842751339 1.793465876 0.001694915 0.030581767 0.024202852 858 tags=40%, list=5%, signal=38% LPCAT1/CTSH/ADGRF5/ABCA12
GOBP_TRYPTOPHAN_CATABOLIC_PROCESS GOBP_TRYPTOPHAN_CATABOLIC_PROCESS GOBP_TRYPTOPHAN_CATABOLIC_PROCESS 10 0.896324765 1.907475913 0.001694915 0.030581767 0.024202852 563 tags=40%, list=3%, signal=39% IDO1/KYNU/TDO2/KMO
GOBP_CHEMICAL_HOMEOSTASIS_WITHIN_A_TISSUE GOBP_CHEMICAL_HOMEOSTASIS_WITHIN_A_TISSUE GOBP_CHEMICAL_HOMEOSTASIS_WITHIN_A_TISSUE 12 0.845869924 1.853219 0.001697793 0.030581767 0.024202852 858 tags=42%, list=5%, signal=40% LPCAT1/CTSH/HOMER1/ADGRF5/ABCA12
GOBP_COMPLEMENT_ACTIVATION_LECTIN_PATHWAY GOBP_COMPLEMENT_ACTIVATION_LECTIN_PATHWAY

GOBP_COMPLEMENT_ACTIVATION_LECTIN_PATHWAY 12 0.759422571 1.66382123 0.001697793 0.030581767 0.024202852 2178 tags=42%, list=13%, signal=36% SERPING1/FCN1/FCN3/A2M/MFAP4
GOBP_GASTRO_INTESTINAL_SYSTEM_SMOOTH_MUSCLE_CONTRACTION GOBP_GASTRO_INTESTINAL_SYSTEM_SMOOTH_MUSCLE_CONTRACTION GOBP_GASTRO_INTESTINAL_SYSTEM_SMOOTH_MUSCLE_CONTRACTION 12 0.847595611 1.856999814 0.001697793 0.030581767 0.024202852 1140 tags=42%, list=7%, signal=39% SULF1/KIT/PTAFR/SULF2/NMU
GOBP_NEGATIVE_REGULATION_OF_B_CELL_MEDIATED_IMMUNITY GOBP_NEGATIVE_REGULATION_OF_B_CELL_MEDIATED_IMMUNITY GOBP_NEGATIVE_REGULATION_OF_B_CELL_MEDIATED_IMMUNITY 12 0.775279333 1.69856186 0.001697793 0.030581767 0.024202852 889 tags=33%, list=5%, signal=32% C4BPB/C4BPA/FCGR2B/BCL6
GOBP_NEGATIVE_T_CELL_SELECTION GOBP_NEGATIVE_T_CELL_SELECTION GOBP_NEGATIVE_T_CELL_SELECTION 12 0.814979339 1.785540724 0.001697793 0.030581767 0.024202852 1267 tags=42%, list=7%, signal=39% CD74/PTPRC/CCR7/SHH/DOCK2
GOBP_PROTEIN_LOCALIZATION_TO_NUCLEOPLASM GOBP_PROTEIN_LOCALIZATION_TO_NUCLEOPLASM GOBP_PROTEIN_LOCALIZATION_TO_NUCLEOPLASM 12 0.795886275 1.743709672 0.001697793 0.030581767 0.024202852 2344 tags=67%, list=14%, signal=57% TBRG1/CCT3/CCT8/CCT5/CCT2/CCT6A/CCT4/CCT7
GOBP_PYRIMIDINE_RIBONUCLEOSIDE_TRIPHOSPHATE_BIOSYNTHETIC_PROCESS GOBP_PYRIMIDINE_RIBONUCLEOSIDE_TRIPHOSPHATE_BIOSYNTHETIC_PROCESS GOBP_PYRIMIDINE_RIBONUCLEOSIDE_TRIPHOSPHATE_BIOSYNTHETIC_PROCESS 12 0.799192063 1.750952333 0.001697793 0.030581767 0.024202852 2060 tags=50%, list=12%, signal=44% CTPS1/NME1/NME5/NME4/NME7/CAD
GOBP_REGULATION_OF_PODOSOME_ASSEMBLY GOBP_REGULATION_OF_PODOSOME_ASSEMBLY GOBP_REGULATION_OF_PODOSOME_ASSEMBLY 12 0.774334426 1.69649166 0.001697793 0.030581767 0.024202852 2276 tags=50%, list=13%, signal=43% MSN/ASAP1/HCK/LCP1/FSCN1/KIF9
GOBP_REGULATION_OF_UNSATURATED_FATTY_ACID_BIOSYNTHETIC_PROCESS GOBP_REGULATION_OF_UNSATURATED_FATTY_ACID_BIOSYNTHETIC_PROCESS GOBP_REGULATION_OF_UNSATURATED_FATTY_ACID_BIOSYNTHETIC_PROCESS 12 0.782797931 1.715034379 0.001697793 0.030581767 0.024202852 492 tags=33%, list=3%, signal=32% ANXA1/IL1B/CD74/PTGS2
GOBP_TRYPTOPHAN_METABOLIC_PROCESS GOBP_TRYPTOPHAN_METABOLIC_PROCESS GOBP_TRYPTOPHAN_METABOLIC_PROCESS 12 0.833784137 1.826740213 0.001697793 0.030581767 0.024202852 563 tags=33%, list=3%, signal=32% IDO1/KYNU/TDO2/KMO
GOBP_INTERLEUKIN_27_MEDIATED_SIGNALING_PATHWAY GOBP_INTERLEUKIN_27_MEDIATED_SIGNALING_PATHWAY GOBP_INTERLEUKIN_27_MEDIATED_SIGNALING_PATHWAY 11 0.805461745 1.730031881

0.00170068 0.030581767 0.024202852 2858 tags=82%, list=17%, signal=68% IL27RA/JAK2/STAT1/STAT3/IL6ST/EBI3/CANX/TYK2/JAK1
GOBP_INTERLEUKIN_35_MEDIATED_SIGNALING_PATHWAY GOBP_INTERLEUKIN_35_MEDIATED_SIGNALING_PATHWAY GOBP_INTERLEUKIN_35_MEDIATED_SIGNALING_PATHWAY11 0.80194809 1.722484988 0.00170068 0.030581767 0.024202852 2858 tags=82%, list=17%, signal=68% IL27RA/JAK2/STAT1/STAT4/STAT3/IL6ST/EBI3/CANX/JAK1
GOBP_KYNURENINE_METABOLIC_PROCESS GOBP_KYNURENINE_METABOLIC_PROCESS GOBP_KYNURENINE_METABOLIC_PROCESS 11 0.89925518 1.931488542 0.00170068 0.030581767 0.024202852 563 tags=36%, list=3%, signal=35% IDO1/KYNU/TDO2/KMO
GOBP_PROTEIN_FOLDING_IN_ENDOPLASMIC_RETICULUM GOBP_PROTEIN_FOLDING_IN_ENDOPLASMIC_RETICULUM GOBP_PROTEIN_FOLDING_IN_ENDOPLASMIC_RETICULUM 11 0.857005769 1.84074205 0.00170068 0.030581767 0.024202852 2367 tags=91%, list=14%, signal=78% DNAJC10/DNAJC3/HSPA5/P4HB/CALR/ERO1A/HSP90B1/ERO1B/PDIA3/CANX
GOBP_PEPTIDYL_TYROSINE_MODIFICATION GOBP_PEPTIDYL_TYROSINE_MODIFICATION GOBP_PEPTIDYL_TYROSINE_MODIFICATION 349 0.399654291 1.495023021 0.002336449 0.041708234 0.033008499 2917 tags=29%, list=17%, signal=24% GREM1/PECAM1/AGT/CAV1/LYN/LRP8/CD44/HCLS1/CD74/TGFB1/JAK3/SH3BP5/PTPRC/TPST2/RIPK2/SOCS3/ITGA5/CLEC7A/ENPP2/PARP9/KIT/PDGFRA/SAMSN1/FYN/ITGB2/JAK2/THY1/TIE1/TTL/FGR/HCK/ICAM1/PDGFRB/CD300A/HYAL2/SEMA4D/MET/CD81/CD40/DDR2/PARP14/KDR/MST1R/TEK/GPRC5B/STAT3/EFEMP1/BANK1/MELK/IL6ST/CSF1R/LCK/FGFR1/AFAP1L2/SPINK1/NRP1/PRNP/MIF/BTK/GPRC5A/ITK/IBTK/ACVR1/DUSP22/LIF/IL23A/EPHB4/IL6/TSLP/LILRB4/AXL/TPST1/ARHGEF2/SFRP1/FGF7/MAP2K1/IFNG/SCYL1/NOD2/SFRP2/PDGFC/DYRK4/ACE/BLK/PTPN1/CD4/HAX1/IL24/SYK/NCAPG2/FBXW7/BMP6/TYRO3/TNFRSF18/IL18/TYK2/ARRB2/PPP2R5B/JAK1/FES/SOCS4
GOBP_NUCLEAR_TRANSPORT GOBP_NUCLEAR_TRANSPORT GOBP_NUCLEAR_TRANSPORT 316 0.425750036 1.58044692 0.002366864 0.041708234 0.033008499 4469 tags=48%, list=26%, signal=36% MMP12/IL1B/HCLS1/TGFB1/BAG3/JAK2/SHH/IPO4/KPNA2/HHEX/SEC13/HYAL2/FLNA/PTPN14/HMGA1/MAGOHB/STAT3/XPOT/RRS1/RAB23/CALR/TNPO1/DDX39A/DESI1/MX2/PABPN1/ECT2/NUP62/TNPO2/BARD1/ABCE1/RAN/DDX19A/NOLC1/E2F3/NUP98/IPO9/RANGAP1/HIKESHI/PML/SNRPG/ZPR1/NEDD4/PKIA/NUP85/SNRPD1/IFNG/NXT1/KPNB1/POLR2D/APOD/SRSF7/PPP3CA/CSE1L/MCM3AP/SDAD1/NUP93/MALT1/NUP210/LRRK2/RAE1/SNRPF/KPNA6/FERMT1/RSRC1/HSP90AB1/TRIM28/NUP37/SYK/SMG7/LTV1/NSUN2/AKT1/THOC1/PARK7/SNRPB/NPM1/GEMIN6/GEMIN8/TARDBP/NUP62CL/NXF3/SMURF1/GEMIN5/PTPN11/HEATR3/NUP58/CPSF3/RAPGEF3/EMD/ALYREF/NUP205/UPF1/ELAVL1/PRICKLE1/UFM1/MBTPS1/SLBP/EIF4A3/IPO7/ALKBH5/UPF2/AAAS/SNRPD2/IWS1/NUP50/RBM8A/FYTTD1/PPP1R12A/PKIG/SRSF1/CHTOP/LSG1/RBM15B/XPO7/NOP9/LZTS2/NUP35/KPNA1/NUP155/NUP107/DDX20/SUPT6H/IPO13/SRSF3/SNRPE/AGFG1/POM121L12/RANBP2/XPO5/NOL6/SRSF4/BANF1/SRSF6/BACH2/NDC1/NUP188/STRADA/NUP88/MDFIC/GEMIN4/THOC7/THOC5/NUP54/GTSE1/U2AF1/KPNA3/NXF1/ENY2/GCKR/SRRM1
GOBP_MAINTENANCE_OF_LOCATION GOBP_MAINTENANCE_OF_LOCATION GOBP_MAINTENANCE_OF_LOCATION 295 0.418179231 1.544313644 0.002389486

0.041708234 0.033008499 1762 tags=20%, list=10%, signal=18%
S100A8/LCN2/GJA1/CEMIP/CXCL9/S100A9/CXCL11/KDELR3/CXCL10/IL1B/CAV1/C3/LYN/FBN1/SRGN/TRPA1/SQLE/LPL/JPH1/APLNR/PTPRC/F2R/STARD4/S100A7/PLN/CCR7/CD19/HSPA5/ANXA6/THY1/HK1/FFAR2/PLCG2/OSBPL8/DIAPH1/CORO1A/FLNA/CALR/CLIC2/ERO1A/PLIN3/SELENON/INSIG1/LCK/HILPDA/CCL19/GSTO1/GM2A/BARD1/PKD2/HSP90B1/HK2/NRROS/IBTK/ITGAV/RANGAP1/PML/IL6/SLC30A7

GOBP_INTRINSIC_APOPTOTIC_SIGNALING_PATHWAY GOBP_INTRINSIC_APOPTOTIC_SIGNALING_PATHWAY GOBP_INTRINSIC_APOPTOTIC_SIGNALING_PATHWAY 268 0.437564346 1.601580236 0.002415459 0.041708234 0.033008499 3464 tags=35%, list=20%, signal=28%
S100A8/VNN1/S100A9/BCL2A1/MMP9/CAV1/IFI6/CD44/HYOU1/CD74/IFI16/CLU/BNIP3/HIF1A/SGMS1/XBP1/BOK/DNAJC10/JAK2/CEBPB/MAP3K5/DDIT4/RNF183/SKIL/CASP4/CREB3L1/SNAI2/P4HB/DNAJA1/NME5/ERO1A/ENO1/MELK/ATP2A3/TNFRSF10B/LCK/MUC1/DDIAS/TNFRSF1B/BID/AEN/MIF/PDK1/GRINA/TMEM109/HIC1/PML/MSH6/CREB3/ARHGEF2/PARP1/ERN1/BCL2L12/PLEKHF1/MLH1/SELENOK/NBN/CHAC1/FIGNL1/SFRP2/SHISA5/LRRK2/PTPN1/NOC2L/SOD2/GPX1/FBXW7/CYLD/ATF4/AKT1/PARK7/POLB/CUL1/TRIAP1/PPM1F/NACC2/PLAUR/MLLT11/CCAR2/YBX3/HRAS/RRP8/SFPQ/TMEM161A/SELENOS/HERPUD1/BRCA1/MYBBP1A/NOX1/USP47/FBH1/BAK1/ITPR1

GOBP_ETHANOL_OXIDATION GOBP_ETHANOL_OXIDATION GOBP_ETHANOL_OXIDATION 12 -0.818069823 -1.953475359 0.002421308 0.041708234 0.033008499 2423 tags=75%, list=14%, signal=64%
ADH5/ALDH1B1/ACSS1/ADH4/ALDH1A1/ACSS2/ADH1A/ADH6/ADH1C

GOBP_BILE_ACID_SECRETION GOBP_BILE_ACID_SECRETION GOBP_BILE_ACID_SECRETION 10 -0.870868812 -1.950844529 0.002427184 0.041708234 0.033008499 1252 tags=50%, list=7%, signal=46%
PRKAA1/SLC9A3R1/ABCB11/SLC51B/SLC51A

GOBP_DEFENSE_RESPONSE_TO_VIRUS GOBP_DEFENSE_RESPONSE_TO_VIRUS GOBP_DEFENSE_RESPONSE_TO_VIRUS 241 0.458681851 1.659692985 0.00245098 0.041708234 0.033008499 2073 tags=26%, list=12%, signal=23%
MMP12/DMBT1/CXCL9/CXCL10/IFITM2/IFIT3/GBP1/MLKL/IFITM3/ISG20/IL33/TRIM22/HTRA1/IFI6/IFI16/IFITM1/AIM2/BIRC3/PTPRC/OAS2/SEC14L1/BNIP3/STING1/TLR8/PARP9/DNAJC3/APOBEC1/BST2/DDX21/SLFN11/STAT1/DDIT4/NLRC5/HYAL2/SAMHD1/MICB/CD40/RNASE6/FLNA/APOBEC3G/FCN3/TRIM15/IRF1/OAS3/LYST/IFNAR2/IL12RB1/MX2/KCNJ8/ABCE1/RTP4/IL23A/PML/IL6/IRF9/LILRB1/IFNG/MX1/STAT2/ZBP1/SELENOK/SHFL/RELA

GOBP_MAINTENANCE_OF_LOCATION_IN_CELL GOBP_MAINTENANCE_OF_LOCATION_IN_CELL GOBP_MAINTENANCE_OF_LOCATION_IN_CELL 196 0.472692188 1.674233369 0.002515723 0.041708234 0.033008499 2031 tags=23%, list=12%, signal=21%
S100A8/LCN2/GJA1/CEMIP/CXCL9/S100A9/CXCL11/KDELR3/CXCL10/CAV1/LYN/SRGN/TRPA1/JPH1/APLNR/PTPRC/F2R/PLN/CCR7/CD19/HSPA5/ANXA6/THY1/HK1/PLCG2/DIAPH1/CORO1A/CALR/CLIC2/ERO1A/SELENON/INSIG1/LCK/CCL19/GSTO1/BARD1/PKD2/HSP90B1/HK2/IBTK/RANGAP1/PML/SLC30A7/AP3D1/CCR5/SYNE1

GOBP_FATTY_ACID_BETA_OXIDATION_USING_ACYL_COA_OXIDASE GOBP_FATTY_ACID_BETA_OXIDATION_USING_ACYL_COA_OXIDASE GOBP_FATTY_ACID_BETA_OXIDATION_USING_ACYL_COA_OXIDASE 15 -0.790717618

-1.984667436 0.002518892 0.041708234 0.033008499 1901 tags=53%, list=11%, signal=47% CRAT/SCP2/ACAA1/ACOT8/ACOX2/CROT/EHHADH/ACOX1
GOBP_STRESS_RESPONSE_TO_METAL_ION GOBP_STRESS_RESPONSE_TO_METAL_ION GOBP_STRESS_RESPONSE_TO_METAL_ION 15 -0.781383205 -1.96123846 0.002518892 0.041708234 0.033008499 989 tags=73%, list=6%, signal=69% MT3/MT2A/SLC30A1/MT1HL1/MT1E/MT1X/MT1H/MT1G/MT1F/MT1M/SLC30A10
GOBP_DETOXIFICATION_OF_INORGANIC_COMPOUND GOBP_DETOXIFICATION_OF_INORGANIC_COMPOUND GOBP_DETOXIFICATION_OF_INORGANIC_COMPOUND 14 -0.801617035 -1.978133465 0.002538071 0.041708234 0.033008499 1554 tags=71%, list=9%, signal=65% MT3/MT2A/SLC30A1/MT1HL1/MT1E/MT1X/MT1H/MT1G/MT1F/MT1M
GOBP_NUCLEAR_EXPORT GOBP_NUCLEAR_EXPORT GOBP_NUCLEAR_EXPORT 186 0.455522626 1.606871765 0.002538071 0.041708234 0.033008499 4717 tags=51%, list=28%, signal=37% IL1B/TGFB1/BAG3/HHEX/SEC13/PTPN14/MAGOHB/XPOT/RRS1/CALR/DDX39A/DESI1/PABPN1/NUP62/BARD1/ABCE1/RAN/DDX19A/NUP98/RANGAP1/NUP85/NXT1/POLR2D/SRSF7/CSE1L/MCM3AP/SDAD1/NUP93/MALT1/NUP210/RAE1/NUP37/SMG7/LTV1/NSUN2/THOC1/PARK7/NPM1/NUP62CL/NXF3/SMURF1/PTPN11/NUP58/CPSF3/RAPGEF3/EMD/ALYREF/NUP205/UPF1/SLBP/EIF4A3/ALKBH5/UPF2/AAAS/IWS1/NUP50/RBM8A/FYTTD1/SRSF1/CHTOP/LSG1/RBM15B/XPO7/NOP9/LZTS2/NUP35/NUP155/NUP107/SUPT6H/SRSF3/AGFG1/POM121L12/RANBP2/XPO5/NOL6/SRSF4/SRSF6/NDC1/NUP188/STRADA/NUP88/THOC7/THOC5/NUP54/GTSE1/U2AF1/NXF1/ENY2/SRRM1/XPO6/NUP214/CPSF6/SYMPK/XPO1
GOBP_BONE_DEVELOPMENT GOBP_BONE_DEVELOPMENT GOBP_BONE_DEVELOPMENT 183 0.45840437 1.615829478 0.002541296 0.041708234 0.033008499 2647 tags=30%, list=16%, signal=26% GREM1/GJA1/COL1A1/CSGALNACT1/SPNS2/ENG/FBN1/BGN/SULF1/STC1/CHSY1/PTPRC/P3H1/TWIST1/KIT/TGFBR2/PLS3/ANXA6/SH3PXD2B/LOX/SULF2/SEMA4D/TYROBP/SNX10/FLI1/LTF/TEK/SERPINH1/RAB23/MEIS1/SP5/INSIG1/RFLNB/CLDN18/PIP4K2A/SLC9B2/ANXA2/EXT2/PPIB/DCHS1/LILRB1/TMEM119/SMAD5/TNFSF11/ALPL/SFRP2/NAB1/VKORC1/PDGFC/TGFB3/NAB2/FBXW7/BMP6/SLC38A10/TULP3
GOBP_POSITIVE_REGULATION_OF_PEPTIDASE_ACTIVITY GOBP_POSITIVE_REGULATION_OF_PEPTIDASE_ACTIVITY GOBP_POSITIVE_REGULATION_OF_PEPTIDASE_ACTIVITY 184 0.446009294 1.571981353 0.002541296 0.041708234 0.033008499 1843 tags=25%, list=11%, signal=23% S100A8/S100A9/CAV1/SERPINB3/ROBO1/CASP1/LYN/CTSH/F3/CCN1/PCOLCE/IFI16/AIM2/F2R/RIPK2/FN1/BOK/ANTXR1/PSME4/CLEC7A/FYN/JAK2/DLC1/MAP3K5/TNFRSF10A/LAPTM5/AKIRIN2/PSMD14/ASPH/STAT3/VCP/XDH/SEMG1/CASP10/ATP2A3/TNFSF15/TNFRSF10B/LCK/BID/DAP/CFLAR/PML/CASP8/ADRM1/APH1B/NLRP2
GOBP_REGENERATION GOBP_REGENERATION GOBP_REGENERATION 179 0.456368445 1.600453194 0.002554278 0.041708234 0.033008499 2575 tags=27%, list=15%, signal=23% REG1A/TNC/NNMT/ANXA1/SPP1/CLDN1/PRRX1/PKM/LPIN1/ANXA3/CCNB1/UCP2/C5AR1/TGFBR2/JAK2/CEBPB/THY1/PPAT/POSTN/SULF2/CD81/FLNA/LCP1/EZH2/AURKA/DUSP10/CCND1/TYMS/LAMB2/JAM3/CFLAR/ADAM15/FPGS/IL6/AXL/MAP1B/MAP2K1/PRMT5/APOD/CAD/PTPRS/

PPP3CA/KLK6/NINJ2/CDK4/NINJ1/GPX1/MDK/ANGPT2
GOBP_RESPIRATORY_SYSTEM_DEVELOPMENT GOBP_RESPIRATORY_SYSTEM_DEVELOPMENT GOBP_RESPIRATORY_SYSTEM_DEVELOPMENT 181 0.442016403 1.552808561 0.002557545 0.041708234 0.033008499 3148 tags=32%, list=19%, signal=26% CHI3L1/MMP12/IGFBP5/TNC/SPARC/WNT5A/SLC7A11/CRISPLD2/BASP1/CTSH/ADA/HEG1/ASS1/PDPN/HSD11B1/TGFBR2/PDGFRA/SHH/FOXF1/LOX/PDGFRB/MYOCD/ABCA12/ALDH1A3/KLF2/LEF1/ZFPM2/MME/LIPA/STK40/LIF/HIKESHI/LAMA5/FGF7/TIMELESS/MAP2K1/CELSR1/PLOD3/CREB1/JMJD6/RBP4/MAN1A2/WNT2/FSTL3/TGFB3/FBXW7/TULP3/SPDEF/ATP7A/CHD7/DLG5/MSC/FGF2/SPRY1/ITGA3/NFIB/RC3H2/ERRFI1
GOBP_POSITIVE_REGULATION_OF_EPITHELIAL_CELL_PROLIFERATION GOBP_POSITIVE_REGULATION_OF_EPITHELIAL_CELL_PROLIFERATION GOBP_POSITIVE_REGULATION_OF_EPITHELIAL_CELL_PROLIFERATION 176 0.480090526 1.678275868 0.00257732 0.041708234 0.033008499 2859 tags=30%, list=17%, signal=25% REG1A/REG3A/MMP12/CCL11/WNT5A/CLDN1/CDH3/LAMC1/HYAL1/F3/HTRA1/MYDGF/NME1/APLNR/ECM1/HIF1A/XBP1/TWIST1/C5AR1/SHH/CAV2/CCL24/VIP/VEGFC/KDR/TEK/FGFR1/LRG1/LAMB1/PIK3CD/CCND1/NRP1/PPP1R16B/CFLAR/EGR3/AKT3/FGFBP1/SFRP1/FGF7/ANG/EGFL7/NOD2/TGFBR1/WNT2/MDK/BMP6/AKT1/ITGA4/JCAD/ATP7A/FGF2/NR4A3
GOBP_AMINOGLYCAN_METABOLIC_PROCESS GOBP_AMINOGLYCAN_METABOLIC_PROCESS GOBP_AMINOGLYCAN_METABOLIC_PROCESS 170 0.478602668 1.667807686 0.002590674 0.041708234 0.033008499 2852 tags=29%, list=17%, signal=25% CHI3L1/CEMIP/IL1B/CSGALNACT1/CHST15/HYAL1/CD44/TGFB1/BGN/CHI3L2/CHST2/CHST11/CHSY1/LUM/VCAN/ST3GAL4/DSE/DCN/PDGFRB/HYAL2/CHPF/SDC2/B3GALT6/HS3ST3B1/CSGALNACT2/SPOCK2/HS3ST1/GPC6/FMOD/HPSE/EXT2/HGSNAT/CHST12/ST3GAL2/GALNT5/SDC3/AGRN/B3GNT7/LYVE1/B3GNT6/B4GALT2/B4GALT6/HS6ST2/CHPF2/CHST3/AKT1/NAGLU/HS3ST3A1/GPC1/FGF2
GOBP_CARTILAGE_DEVELOPMENT GOBP_CARTILAGE_DEVELOPMENT GOBP_CARTILAGE_DEVELOPMENT 174 0.475365386 1.659248367 0.002590674 0.041708234 0.033008499 1404 tags=20%, list=8%, signal=19% CHI3L1/TIMP1/GREM1/MGP/LOXL2/TGFBI/WNT5A/PRRX1/COL1A1/CREB3L2/CSGALNACT1/HYAL1/CCN1/CD44/TGFB1/BGN/SULF1/STC1/CHST11/CHSY1/LUM/ECM1/HIF1A/RUNX2/TGFBR2/ANXA6/CHRDL2/SULF2/HYAL2/SNAI2/SERPINH1/EFEMP1/RFLNB/RUNX3/TYMS
GOBP_NEGATIVE_REGULATION_OF_CELL_CELL_ADHESION GOBP_NEGATIVE_REGULATION_OF_CELL_CELL_ADHESION GOBP_NEGATIVE_REGULATION_OF_CELL_CELL_ADHESION 169 0.455193548 1.585163678 0.00260078 0.041708234 0.033008499 3388 tags=35%, list=20%, signal=28% IL1RN/ANXA1/CD274/IDO1/RGCC/SERPINE2/ASS1/LAX1/CD74/TGFB1/JAK3/PODXL/CBFB/FXYD5/MAD2L2/FCGR2B/JAK2/SHH/CEBPB/LILRB2/CD86/CD300A/BCL6/LAPTM5/IRF1/RIPOR2/METTL3/RUNX3/TWSG1/PRNP/LAG3/GTPBP4/LILRB4/TMEM131L/LILRB1/GPNMB/JAG1/RDX/HAVCR2/HLX/SWAP70/GLMN/MDK/LRRC32/BMP6/AKT1/PRKAR1A/C1QTNF1/DLG5/BTN2A2/PPM1F/AKNA/CTLA4/RC3H2/NF2/IL2RA/PLA2G2D/HMGB1/CR1
GOBP_MOVEMENT_IN_HOST_ENVIRONMENT GOBP_MOVEMENT_IN_HOST_ENVIRONMENT GOBP_MOVEMENT_IN_HOST_ENVIRONMENT 167 0.446412991 1.552914627 0.002604167 0.041708234 0.033008499 3530 tags=37%, list=21%, signal=30%

CD55/CXCL8/CLDN1/IFITM2/CAV1/SERPINB3/IFITM3/HYAL1/TRIM22/ITGA2/CD74/IFITM1/LGALS1/ITGA5/FCN1/CXCR4/CD86/CAV2/TRIM27/NPC1/ICAM1/HYAL2/MET/CD81/FCN3/P4HB/TRIM15/MRC1/KRT6A/CDK1/LY6E/SELPLG/NRP1/CR2/ITGAV/PML/CTSL/AXL/TNFRSF4/CCR5/CLEC5A/CD4/TRIM28/SLC1A5/NECTIN4/TYRO3/SLC52A2/FUCA2/PVR/TRIM5/TRIM21/EFNB2/TRIM8/IDE/PLSCR1/HYAL3/VPS4A/DDB1/CR1/CD209/LDLR/TRIM26

GOBP_CYTOSOLIC_CALCIUM_ION_TRANSPORT GOBP_CYTOSOLIC_CALCIUM_ION_TRANSPORT GOBP_CYTOSOLIC_CALCIUM_ION_TRANSPORT 164 0.44236613 1.535824293 0.002610966 0.041708234 0.033008499 2122 tags=24%, list=13%, signal=21% CEMIP/CXCL9/CXCL11/CXCL10/CAV1/MS4A1/LYN/TRPA1/MCUB/JPH1/APLNR/PTPRC/F2R/RAMP3/PLN/CCR7/CD19/FYN/THY1/P2RX5/PLCG2/DIAPH1/CORO1A/CLIC2/ERO1A/ATP2A3/SELENON/LCK/CCL19/GSTO1/PKD2/PRNP/TRPV2/IBTK/PML/ATP2B4/ATP2A2/CCR5/P2RX7

GOBP_NIK_NF_KAPPAB_SIGNALING GOBP_NIK_NF_KAPPAB_SIGNALING GOBP_NIK_NF_KAPPAB_SIGNALING 161 0.468990172 1.62206902 0.002638522 0.041708234 0.033008499 4138 tags=47%, list=24%, signal=36% CHI3L1/GREM1/IL1B/BIRC3/TCIM/PSME4/PSMB9/TRIM40/CD86/TLR2/CD27/IRAK1/TNFRSF10A/RASSF2/TRIP6/LAPTM5/PSMD14/CALR/TNFSF15/TNFRSF10B/PSMD12/PSMC2/CCL19/PSMB2/SPHK1/PSMA3/IL18R1/PSMD1/HDAC7/IL23A/PSMA5/AGO3/PSMD6/PSMB5/PSMC4/HAVCR2/DICER1/RELA/NOD2/NFKB2/REL/PSMB1/PSMA1/MALT1/NMI/PSME3/CYLD/AKT1/IL18/CUL1/PSMA7/PSMB8/PSMC5/LIME1/COPS8/PSMB10/PSMB6/PSMA4/RC3H2/PSMD13/IFI35/PSME1/HMGB1/ADGRG3/PSMC1/MAP3K14/PSMB4/TERF2IP/PSMB3/PSMD2/PSMC6/PSMD7/PSMA2/PSMD4/ACTN4/SASH1

GOBP_MITOTIC_SISTER_CHROMATID_SEGREGATION GOBP_MITOTIC_SISTER_CHROMATID_SEGREGATION GOBP_MITOTIC_SISTER_CHROMATID_SEGREGATION 159 0.450859233 1.554494907 0.002659574 0.041708234 0.033008499 3908 tags=43%, list=23%, signal=33% TRIP13/CCNB1/MAD2L2/TUBG1/BUB1/CDC20/PRC1/NCAPG/NDC80/NEK6/RRS1/CDCA5/TACC3/PTTG1/KIF14/CDC6/MIS12/KNSTRN/TENT4A/NUP62/CENPE/AURKB/BUB1B/NUSAP1/PLK1/UBE2C/RAN/SMC4/DLGAP5/KIF23/CDC27/ZWINT/NUDC/KPNB1/KIF2C/PSRC1/HECW2/CDCA8/KIF4A/CDC26/PINX1/SPAG5/NCAPH/NCAPG2/CDT1/SPDL1/KLHL22/EML3/KNTC1/ANAPC7/FBXO5/SGO2/RACGAP1/SMC2/TUBG2/VPS4A/NUF2/DSCC1/KIF18A/REC8/NAA10/KIF18B/ZNF207/CENPF/BUB3/TTK/MAD2L1/DYNC1LI1

GOBP_DNA_DEPENDENT_DNA_REPLICATION GOBP_DNA_DEPENDENT_DNA_REPLICATION GOBP_DNA_DEPENDENT_DNA_REPLICATION 153 0.497907157 1.70652095 0.002663116 0.041708234 0.033008499 3668 tags=38%, list=22%, signal=30% FEN1/NOC3L/DDX21/SLFN11/POLD3/SAMHD1/DONSON/RTEL1/BCL6/HMGA1/BLM/MCM6/CDC45/CDK2AP1/CDC6/MCM10/ATR/TWNK/TIPIN/DACH1/RFC3/GINS3/ZPR1/CDC7/TIMELESS/POLE2/ORC6/DBF4/NBN/CHTF18/MCM3/GINS1/MCM5/SSBP1/RFC4/CDT1/POLD2/THOC1/POLB/MCM7/RECQL/PNKP/ORC5/EME1/GINS2/FBXO5/ALYREF/BAZ1A/UPF1/CENPX/POLA2/FBH1/POLG/MCM2/DSCC1/ZNF830/DDX23/DDX11

GOBP_CELLULAR_RESPONSE_TO_COPPER_ION GOBP_CELLULAR_RESPONSE_TO_COPPER_ION GOBP_CELLULAR_RESPONSE_TO_COPPER_ION 25 -0.692592534 -1.929190757 0.002673797 0.041708234 0.033008499 1554 tags=48%, list=9%, signal=44% MT3/CYP1A1/MT2A/MT1HL1/MT1E/AOC1/MT1X/MT1H/APP/MT1G/MT1F/MT1M

GOBP_REGULATION_OF_INTRINSIC_APOPTOTIC_SIGNALING_PATHWAY

GOBP_REGULATION_OF_INTRINSIC_APOPTOTIC_SIGNALING_PATHWAY
GOBP_REGULATION_OF_INTRINSIC_APOPTOTIC_SIGNALING_PATHWAY 147 0.483774875 1.654982996 0.002677376 0.041708234 0.033008499 3639 tags=39%, list=21%, signal=31%
S100A8/VNN1/S100A9/MMP9/CAV1/CD44/HYOU1/CD74/CLU/HIF1A/SGMS1/XBP1/BOK/RNF183/SKIL/CREB3L1/SNAI2/P4HB/DNAJA1/NME5/ENO1/LCK/MUC1/DDIAS/BID/MIF/GRINA/CREB3/ARHGEF2/PARP1/BCL2L12/PLEKHF1/FIGNL1/SFRP2/LRRK2/PTPN1/NOC2L/SOD2/GPX1/FBXW7/CYLD/AKT1/PARK7/TRIAP1/NACC2/PLAUR/CCAR2/YBX3/SFPQ/TMEM161A/SELENOS/HERPUD1/NOX1/USP47/FBH1/PPIF/MDM2/TRAP1

GOBP_ENTRY_INTO_HOST GOBP_ENTRY_INTO_HOST GOBP_ENTRY_INTO_HOST 146 0.482445447 1.651351414 0.002680965 0.041708234 0.033008499 2822 tags=34%, list=17%, signal=28%
CD55/CXCL8/CLDN1/IFITM2/CAV1/SERPINB3/IFITM3/HYAL1/TRIM22/ITGA2/CD74/IFITM1/LGALS1/ITGA5/FCN1/CXCR4/CD86/CAV2/TRIM27/NPC1/ICAM1/HYAL2/MET/CD81/FCN3/P4HB/MRC1/KRT6A/CDK1/LY6E/SELPLG/NRP1/CR2/ITGAV/CTSL/AXL/TNFRSF4/CCR5/CLEC5A/CD4/SLC1A5/NECTIN4/TYRO3/SLC52A2/FUCA2/PVR/TRIM5/TRIM21/EFNB2

GOBP_EPOXYGENASE_P450_PATHWAY GOBP_EPOXYGENASE_P450_PATHWAY GOBP_EPOXYGENASE_P450_PATHWAY 19 -0.823237376 -2.151220045 0.002680965 0.041708234 0.033008499 1398 tags=37%, list=8%, signal=34%
CYP2J2/CYP1A1/CYP4F12/CYP4F2/EPHX2/CYP2S1/CYP2B6

GOBP_CALCIUM_ION_TRANSPORT_INTO_CYTOSOL
GOBP_CALCIUM_ION_TRANSPORT_INTO_CYTOSOL
GOBP_CALCIUM_ION_TRANSPORT_INTO_CYTOSOL 143 0.491540739 1.680842105 0.002702703 0.041708234 0.033008499 2122 tags=25%, list=13%, signal=22%
CEMIP/CXCL9/CXCL11/CXCL10/CAV1/MS4A1/LYN/TRPA1/JPH1/APLNR/PTPRC/F2R/RAMP3/PLN/CCR7/CD19/FYN/THY1/P2RX5/PLCG2/DIAPH1/CORO1A/CLIC2/ERO1A/SELENON/LCK/CCL19/GSTO1/PKD2/PRNP/TRPV2/IBTK/PML/ATP2B4/CCR5/P2RX7

GOBP_TUMOR_NECROSIS_FACTOR_SUPERFAMILY_CYTOKINE_PRODUCTION
GOBP_TUMOR_NECROSIS_FACTOR_SUPERFAMILY_CYTOKINE_PRODUCTION
GOBP_TUMOR_NECROSIS_FACTOR_SUPERFAMILY_CYTOKINE_PRODUCTION 138 0.494339391 1.685536887 0.002717391 0.041708234 0.033008499 2249 tags=32%, list=13%, signal=28%
CD274/WNT5A/IL33/CCR2/PF4/LY96/LPL/IL27RA/PTPRC/CLU/RIPK2/CLEC7A/CLEC4A/TWIST1/JAK2/CD86/TRIM27/SASH3/TLR2/PTAFR/TYROBP/SIRPA/IL1A/LTF/STAT3/UBE2J1/CCL19/CD2/MIF/TLR1/CD47/IL23A/IL6/ADAM8/AXL/ARHGEF2/LILRB1/IFNG/HAVCR2/SELENOK/DICER1/NOD2/SYT11/MAPKAPK2

GOBP_ENDOTHELIAL_CELL_PROLIFERATION GOBP_ENDOTHELIAL_CELL_PROLIFERATION GOBP_ENDOTHELIAL_CELL_PROLIFERATION 140 0.508198463 1.732571859 0.002724796 0.041708234 0.033008499 3079 tags=36%, list=18%, signal=30%
CCL11/GJA1/LOXL2/PROK2/SPARC/WNT5A/RGCC/CAV1/ALOX5/F3/SULF1/MYDGF/APLNR/ECM1/HIF1A/PTPRM/STAT1/CAV2/TIE1/CCL2/CCL24/VIP/VEGFC/KDR/TEK/XDH/FGFR1/LRG1/PIK3CD/ITGB1BP1/NRP1/PPP1R16B/EGR3/AKT3/FGFBP1/ERN1/VASH1/ANG/EGFL7/TGFBR1/WNT2/MDK/BMP6/AKT1/ITGA4/JCAD/APOE/FGF2/CD34/STAT5A/NR2F2

GOBP_REGULATION_OF_EPITHELIAL_CELL_DIFFERENTIATION

GOBP_REGULATION_OF_EPITHELIAL_CELL_DIFFERENTIATION
GOBP_REGULATION_OF_EPITHELIAL_CELL_DIFFERENTIATION 139 0.489142513 1.667267335 0.002724796 0.041708234 0.033008499 1892 tags=22%, list=11%, signal=20%
REG3A/AQP3/IL1B/CTSK/MMP9/CAV1/SERPINE1/CDH5/S1PR3/CBFB/ZEB1/CEBPB/STAT1/TRIM16/XDH/WWTR1/EZH2/SPRED1/APOLD1/PLCB1/CYP27B1/CCND1/PRKCH/LIF/VCL/CD109/AHI1/CTSL/ZBED2/IFNG/JAG1
GOBP_REGULATION_OF_PRODUCTION_OF_MOLECULAR_MEDIATOR_OF_IMMUNE_RESPONSE
GOBP_REGULATION_OF_PRODUCTION_OF_MOLECULAR_MEDIATOR_OF_IMMUNE_RESPONSE
GOBP_REGULATION_OF_PRODUCTION_OF_MOLECULAR_MEDIATOR_OF_IMMUNE_RESPONSE 141 0.499624745 1.705394598 0.00273224 0.041708234 0.033008499 2123 tags=29%, list=13%, signal=26%
IL13RA2/SPINK5/IL1B/MZB1/WNT5A/SLC7A5/IL33/CCR2/CD74/TGFB1/JAK3/IL27RA/PTPRC/XBP1/TNFSF13/CLEC7A/IL1R1/BST2/MAD2L2/FCGR2B/DDX21/SASH3/HK1/FFAR2/CD81/CD40/BCL6/LAPTM5/GPRC5B/IL18R1/FCRL3/TNFRSF1B/EXOSC3/BTK/IL6/LILRB4/TNFRSF4/LILRB1/MLH1/RBP4/NOD2
GOBP_REGULATION_OF_T_CELL_DIFFERENTIATION
GOBP_REGULATION_OF_T_CELL_DIFFERENTIATION
GOBP_REGULATION_OF_T_CELL_DIFFERENTIATION 141 0.502551068 1.715383164 0.00273224 0.041708234 0.033008499 2677 tags=36%, list=16%, signal=31%
VNN1/ANXA1/SPINK5/IL7R/ADA/CCR2/CD74/JAK3/IRF4/HLA-DRA/NFKBIZ/PNP/PTPRC/CBFB/RIPK2/XBP1/TGFBR2/SHH/LILRB2/CD86/SASH3/CD27/RHOH/BCL6/LEF1/IRF1/IL12RB1/CCL19/METTL3/DUSP10/FANCD2/RUNX3/CD2/ITPKB/LAG3/EGR3/IL23A/ADAM8/LILRB4/TMEM131L/IFNG/AP3D1/PRDM1/NCKAP1L/HLX/MALT1/ZBTB16/MDK/SYK/TNFRSF18/IL18
GOBP_TRANSEPITHELIAL_TRANSPORT GOBP_TRANSEPITHELIAL_TRANSPORT
GOBP_TRANSEPITHELIAL_TRANSPORT 29 -0.68249162 -1.959989665 0.00273224 0.041708234 0.033008499 983 tags=28%, list=6%, signal=26%
CFTR/CXADR/SCNN1B/SLC23A1/SLC1A1/EDN1/ABCB1/ABCG2
GOBP_CELLULAR_RESPONSE_TO_CADMIUM_ION
GOBP_CELLULAR_RESPONSE_TO_CADMIUM_ION
GOBP_CELLULAR_RESPONSE_TO_CADMIUM_ION 34 -0.63169459 -1.873659085 0.002739726 0.041708234 0.033008499 1117 tags=50%, list=7%, signal=47%
MT4/MAPK3/MAPK1/EGFR/MT3/AKR1C3/OGG1/MT2A/MT1HL1/MT1E/MT1X/MT1H/JUN/MT1G/MT1F/HMOX1/MT1M
GOBP_INTERLEUKIN_6_PRODUCTION GOBP_INTERLEUKIN_6_PRODUCTION
GOBP_INTERLEUKIN_6_PRODUCTION 130 0.512553673 1.729547239 0.002754821 0.041708234 0.033008499 2341 tags=34%, list=14%, signal=29%
POU2AF1/IL1B/WNT5A/IL33/NOS2/CD74/LPL/IL27RA/F2R/RIPK2/XBP1/CLEC7A/TLR8/TWIST1/CEBPB/LILRB2/TLR2/PTAFR/HYAL2/TYROBP/SIRPA/IL1A/KLF2/AKIRIN2/STAT3/BANK1/AFAP1L2/TLR1/CD200/CD47/IL6/TSLP/LILRB4/ARHGEF2/IFNG/AIF1/HAVCR2/SELENOK/NCKAP1L/NOD2/INPP5D/SYT11/MAPKAPK2/IL16
GOBP_PLATELET_DEGRANULATION GOBP_PLATELET_DEGRANULATION
GOBP_PLATELET_DEGRANULATION 120 0.54514415 1.820030378 0.002754821

0.041708234 0.033008499 2523 tags=34%, list=15%, signal=29%
TIMP1/SERPINA3/PECAM1/SPARC/VWF/MANF/ANXA5/LYN/SERPING1/SERPINE1/SELP/SERPINA1/TGFB1/SRGN/PF4/STXBP1/ECM1/CLU/FN1/PLEK/ACTN1/FLNA/QSOX1/VEGFC/TOR4A/A2M/PPBP/TUBA4A/MAGED2/VCL/CD109/TLN1/TMX3/FAM3C/MMRN1/ISLR/SERPINF2/BLK/TIMP3/TGFB3/SYK
GOBP_B_CELL_DIFFERENTIATION GOBP_B_CELL_DIFFERENTIATION
GOBP_B_CELL_DIFFERENTIATION 131 0.492623122 1.66244423 0.002758621
0.041708234 0.033008499 2645 tags=32%, list=16%, signal=27%
POU2AF1/MS4A1/ADA/JAK3/INHBA/LGALS1/SLAMF8/PTPRC/DNAJB9/TCIRG1/XBP1/VCAM1/KIT/CD19/FCGR2B/CD27/HHEX/PLCG2/CMTM7/BCL6/MFNG/CD79B/CD79A/EZH2/DOCK11/GPR183/TCF3/FCRL3/CR2/NKX2-3/BTK/IKZF3/IL6/ITM2A/SFRP1/NCKAP1L/INPP5D/MALT1/SYK/LRRC8A/DLL1/ITGA4
GOBP_MODULATION_OF_PROCESS_OF_OTHER_ORGANISM
GOBP_MODULATION_OF_PROCESS_OF_OTHER_ORGANISM
GOBP_MODULATION_OF_PROCESS_OF_OTHER_ORGANISM 114 0.539573397
1.785990982 0.002758621 0.041708234 0.033008499 2151 tags=25%, list=13%, signal=22%
REG1A/REG1B/REG3A/DEFA5/DEFA6/CXCL6/S100A9/SERPINB9/STOM/NOS2/SLPI/CLEC7A/CCL4/CAV2/KPNA2/HYAL2/LEF1/LTF/KRT6A/CSF1R/PABPN1/TYMS/ANXA2/PPIB/CASP8/IFNG/KPNB1/P2RX7/RRP1B
GOBP_CRISTAE_FORMATION GOBP_CRISTAE_FORMATION GOBP_CRISTAE_FORMATION 32
-0.671607726 -1.964644183 0.002762431 0.041708234 0.033008499 4070
tags=62%, list=24%, signal=48%
ATP5F1A/ATP5MC1/ATP5PO/MICOS13/ATP5F1E/LETM1/DNAJC11/ATP5F1D/ATP5PB/IMMT/ATP5MC2/ATP5PF/ATP5F1B/OMA1/ATP5MC3/PINK1/CHCHD10/APOOL/AFG3L2/DMAC2L
GOBP_ERBB2_SIGNALING_PATHWAY GOBP_ERBB2_SIGNALING_PATHWAY
GOBP_ERBB2_SIGNALING_PATHWAY 32 -0.603733895 -1.766093864 0.002762431
0.041708234 0.033008499 2247 tags=38%, list=13%, signal=33%
BTC/EGFR/SOS1/PRKCA/GRB7/PTK6/EREG/GAB1/ERBIN/ERBB2/ERBB3/PTPRR
GOBP_NEGATIVE_REGULATION_OF_IMMUNE_EFFECTOR_PROCESS
GOBP_NEGATIVE_REGULATION_OF_IMMUNE_EFFECTOR_PROCESS
GOBP_NEGATIVE_REGULATION_OF_IMMUNE_EFFECTOR_PROCESS 122 0.534602581
1.789441663 0.002762431 0.041708234 0.033008499 2246 tags=29%, list=13%, signal=25%
CD55/C4BPB/C4BPA/IL13RA2/ANXA1/SPINK5/SERPINB9/IL7R/IL33/SERPING1/HTRA1/CCR2/TGFB1/JAK3/SLAMF8/PTPRC/SEC14L1/SERPINB4/BST2/FCGR2B/FOXF1/CD300A/MICB/BCL6/A2M/CD84/DUSP10/FCRL3/CD47/LILRB4/LILRB1/HAVCR2/NOD2/HLX/CD59
GOBP_NEURONAL_ACTION_POTENTIAL GOBP_NEURONAL_ACTION_POTENTIAL
GOBP_NEURONAL_ACTION_POTENTIAL 32 -0.653912798 -1.91288147 0.002762431
0.041708234 0.033008499 1222 tags=34%, list=7%, signal=32%
SCN7A/MYH14/CACNA1G/P2RX1/CACNA1I/GPR35/FMR1/SCN9A/P2RX4/CHRNA1/ANK3
GOBP_POSITIVE_REGULATION_OF_LEUKOCYTE_MIGRATION
GOBP_POSITIVE_REGULATION_OF_LEUKOCYTE_MIGRATION
GOBP_POSITIVE_REGULATION_OF_LEUKOCYTE_MIGRATION 128 0.49370779

1.663899297 0.002762431 0.041708234 0.033008499 2688 tags=40%, list=16%, signal=34%

CXCL13/CXCL8/CXCL10/WNT5A/PLVAP/ITGA2/SERPINE1/SELP/CCR2/CD74/PLA2G7/RAC2/CCL20/S100A7/CCR1/IL1R1/MADCAM1/C5AR1/CCR7/CCL4/THY1/ICAM1/PTAFR/CCL24/VEGFC/JAM2/CALR/CCR6/ANO6/C3AR1/RIPOR2/CSF1R/GPSM3/CCL19/JAM3/CXCL14/IL23A/IL6/ADAM8/CREB3/AIF1/FPR2/SELENOK/NCKAP1L/SWAP70/CMKLR1/MDK/CCL7/TNFRSF18/ITGA4/DOCK8

GOBP_RESPONSE_TO_MINERALOCORTICOID GOBP_RESPONSE_TO_MINERALOCORTICOID

GOBP_RESPONSE_TO_MINERALOCORTICOID 32 -0.602291717 -1.761875082

0.002762431 0.041708234 0.033008499 1041 tags=22%, list=6%, signal=21%

SGK1/HSD3B1/ATP2B1/SCNN1B/HSD3B2/EDN1/FOSB

GOBP_RNA_EXPORT_FROM_NUCLEUS GOBP_RNA_EXPORT_FROM_NUCLEUS

GOBP_RNA_EXPORT_FROM_NUCLEUS 128 0.515778163 1.738281104 0.002762431

0.041708234 0.033008499 4717 tags=58%, list=28%, signal=42%

HHEX/SEC13/MAGOHB/XPOT/RRS1/DDX39A/PABPN1/NUP62/ABCE1/RAN/DDX19A/NUP98/NUP85/NXT1/POLR2D/SRSF7/MCM3AP/SDAD1/NUP93/NUP210/RAE1/NUP37/SMG7/LTV1/NSUN2/THOC1/NPM1/NUP62CL/NXF3/NUP58/CPSF3/ALYREF/NUP205/UPF1/SLBP/EIF4A3/ALKBH5/UPF2/AAAS/IWS1/NUP50/RBM8A/FYTTD1/SRSF1/CHTOP/LSG1/RBM15B/NOP9/NUP35/NUP155/NUP107/SUPT6H/SRSF3/AGFG1/POM121L12/RANBP2/XPO5/NOL6/SRSF4/SRSF6/NDC1/NUP188/NUP88/THOC7/THOC5/NUP54/U2AF1/NXF1/ENY2/SRRM1/NUP214/CPSF6/SYMPK/XPO1

GOBP_ENDOPLASMIC_RETICULUM_UNFOLDED_PROTEIN_RESPONSE

GOBP_ENDOPLASMIC_RETICULUM_UNFOLDED_PROTEIN_RESPONSE

GOBP_ENDOPLASMIC_RETICULUM_UNFOLDED_PROTEIN_RESPONSE 119 0.514771985

1.713458761 0.002770083 0.041708234 0.033008499 2854 tags=41%, list=17%, signal=34%

CXCL8/KDELR3/DERL3/CREB3L2/SRPRB/DNAJB11/HYOU1/MYDGF/DNAJB9/XBP1/BOK/DNAJC3/FICD/HSPA5/PREB/CCL2/PDIA6/SRPRA/ASNS/CREB3L1/EIF2S1/VCP/CALR/ERO1A/SSR1/FKBP14/AGR2/CCND1/HSP90B1/WFS1/CREB3/TBL2/TLN1/ERN1/YIF1A/DERL2/SERP1/CREB3L4/EDEM1/BFAR/CANX/PTPN1/SEC31A/DERL1/CDK5RAP3/ATF4/ARFGAP1/EXTL2/PPP2R5B

GOBP_INNATE_IMMUNE_RESPONSE_ACTIVATING_SIGNAL_TRANSDUCTION

GOBP_INNATE_IMMUNE_RESPONSE_ACTIVATING_SIGNAL_TRANSDUCTION

GOBP_INNATE_IMMUNE_RESPONSE_ACTIVATING_SIGNAL_TRANSDUCTION 110

0.542075873 1.783989529 0.002770083 0.041708234 0.033008499 4036

tags=53%, list=24%, signal=40%

MUC5B/LYN/FCER1G/MUC4/PSME4/PSMB9/CLEC4A/FCN1/FYN/HCK/FFAR2/PLCG2/TYROBP/ICAM2/PSMD14/CLEC4E/PSMD12/PSMC2/MUC1/PSMB2/PSMA3/PSMD1/PSMA5/MUC5AC/PSMD6/MUC2/PSMB5/PSMC4/RELA/PSMB1/PSMA1/MALT1/PSME3/SYK/CUL1/PSMA7/PSMB8/PSMC5/PSMB10/PSMB6/HRAS/PSMA4/PSMD13/PSME1/CARD11/CD209/PSMC1/PSMB4/MUC17/PSMB3/PSMD2/IKBKG/PSMC6/PSMD7/MUC12/CLEC4D/PSMA2/PSMD4

GOBP_REGULATION_OF_PROTEIN_LOCALIZATION_TO_NUCLEUS

GOBP_REGULATION_OF_PROTEIN_LOCALIZATION_TO_NUCLEUS

GOBP_REGULATION_OF_PROTEIN_LOCALIZATION_TO_NUCLEUS 127 0.50474495

1.698295183 0.002770083 0.041708234 0.033008499 2668 tags=39%, list=16%, signal=33%

TRIM29/FERMT2/HCLS1/TGFB1/BAG3/PARP9/TRIM40/RASSF5/FYN/JAK2/SHH/LATS2/HYAL2

/FLNA/RAB23/WWTR1/CLDN18/CDK1/ECT2/BYSL/NUP62/ATP13A2/PLK1/RAN/NOLC1/LIF/CCT3/ZPR1/LILRB4/PARP1/PKIA/CCT8/IFNG/APOD/CCT5/CCT2/CCT6A/CCT4/CCT7/PINX1/FERMT1/HSP90AB1/TRIM28/GLUL/CDK5RAP3/GLIS2/AKT1/PARK7/DCLK1/FBXO4

GOBP_TRICARBOXYLIC_ACID_CYCLE GOBP_TRICARBOXYLIC_ACID_CYCLE GOBP_TRICARBOXYLIC_ACID_CYCLE 33 -0.602757204 -1.776404581 0.002770083 0.041708234 0.033008499 3540 tags=64%, list=21%, signal=50% OGDH/OGDHL/IDH1/SDHA/PDHA1/IDH3B/IDH3A/MDH1/FAHD1/DLAT/ME2/IREB2/SUCLG1/DLD/SUCLA2/SDHB/ACO2/NNT/SDHD/SUCLG2/ME3

GOBP_REGULATION_OF_MONONUCLEAR_CELL_MIGRATION GOBP_REGULATION_OF_MONONUCLEAR_CELL_MIGRATION GOBP_REGULATION_OF_MONONUCLEAR_CELL_MIGRATION 107 0.531143086 1.739611209 0.002773925 0.041708234 0.033008499 2688 tags=41%, list=16%, signal=35% GREM1/CXCL13/CXCL10/WNT5A/MSN/LYN/SERPINE1/CCR2/PLA2G7/IL27RA/SLAMF8/ECM1/CCL20/S100A7/CCR1/MADCAM1/C5AR1/CCR7/CCL4/CCL2/JAM2/CALR/CCR6/ANO6/LRCH1/C3AR1/RIPOR2/CSF1R/PLCB1/CD200/CXCL14/ADAM8/CREB3/STK10/AIF1/FPR2/APOD/SELENOK/CMKLR1/MDK/CCL7/AKT1/ITGA4/DOCK8

GOBP_ENDOTHELIUM_DEVELOPMENT GOBP_ENDOTHELIUM_DEVELOPMENT GOBP_ENDOTHELIUM_DEVELOPMENT 125 0.540096864 1.81322833 0.002781641 0.041708234 0.033008499 1955 tags=29%, list=12%, signal=26% GJA1/COL15A1/CXCL10/IL1B/PECAM1/FSTL1/CLDN1/MSN/ENG/HEG1/CDH5/COL18A1/STC1/S1PR3/PDPN/ZEB1/CXCR4/TIE1/ICAM1/MET/KDR/S1PR1/FASN/XDH/ROBO4/APOLD1/PLCB1/NRP1/PPP1R16B/ACVR1/GJA4/VCL/ITGAX/PLOD3/JAG1/RDX

GOBP_ALPHA_BETA_T_CELL_DIFFERENTIATION GOBP_ALPHA_BETA_T_CELL_DIFFERENTIATION GOBP_ALPHA_BETA_T_CELL_DIFFERENTIATION 101 0.54967285 1.788602645 0.002793296 0.041708234 0.033008499 2728 tags=45%, list=16%, signal=38% ANXA1/ADA/JAK3/IRF4/HLA-DRA/NFKBIZ/PNP/ENTPD7/CBFB/RIPK2/TGFBR2/SHH/CD86/SASH3/BCL6/LEF1/STAT3/IRF1/IL12RB1/GPR183/CCL19/IL18R1/RUNX3/RORA/NKX2-3/ITPKB/ITK/IL23A/IL6/CTSL/LILRB4/IFNG/AP3D1/BATF/PRDM1/SLAMF6/NCKAP1L/HLX/SEMA4A/MALT1/LY9/ZBTB16/SYK/IL18/ATP7A

GOBP_DRUG_METABOLIC_PROCESS GOBP_DRUG_METABOLIC_PROCESS GOBP_DRUG_METABOLIC_PROCESS 41 -0.687451083 -2.134375142 0.002816901 0.041708234 0.033008499 1398 tags=32%, list=8%, signal=29% CYP2J2/CYP1A1/TPMT/ADH1A/BCHE/CYP4F12/NR1I2/CYP4F2/FMO4/ABCB11/CYP2S1/FMO5/CYP2B6

GOBP_BICARBONATE_TRANSPORT GOBP_BICARBONATE_TRANSPORT GOBP_BICARBONATE_TRANSPORT 39 -0.624704656 -1.913053679 0.002840909 0.041708234 0.033008499 419 tags=21%, list=2%, signal=20% CFTR/CA12/CA2/SLC26A3/SLC4A4/CA4/CA1/SLC26A2

GOBP_CD4_POSITIVE_ALPHA_BETA_T_CELL_ACTIVATION GOBP_CD4_POSITIVE_ALPHA_BETA_T_CELL_ACTIVATION GOBP_CD4_POSITIVE_ALPHA_BETA_T_CELL_ACTIVATION 90 0.551080944 1.752254696 0.00284495 0.041708234 0.033008499 2400 tags=41%, list=14%, signal=35% CD55/ANXA1/CD274/JAK3/IRF4/HLA-DRA/NFKBIZ/ENTPD7/CBFB/TCIRG1/RIPK2/TGFBR2/CD

86/SASH3/CD81/BCL6/LEF1/STAT3/IL12RB1/GPR183/CCL19/IL18R1/RUNX3/TWSG1/RORA/NKX2-3/IL23A/IL6/CTSL/IFNG/BATF/SLAMF6/NCKAP1L/HLX/SEMA4A/MALT1/LY9

GOBP_B_CELL_ACTIVATION_INVOLVED_IN_IMMUNE_RESPONSE GOBP_B_CELL_ACTIVATION_INVOLVED_IN_IMMUNE_RESPONSE GOBP_B_CELL_ACTIVATION_INVOLVED_IN_IMMUNE_RESPONSE 77 0.584905531 1.820786519 0.002881844 0.041708234 0.033008499 3388 tags=47%, list=20%, signal=38% POU2AF1/ADA/TGFB1/IL27RA/LGALS1/PTPRC/XBP1/TNFSF13/CD19/MAD2L2/FCGR2B/PLCG2/CD40/BCL6/MFNG/CCR6/DOCK11/GPR183/EXOSC3/NKX2-3/EXO1/IL6/MSH6/ITM2A/CD180/MLH1/BATF/NBN/SWAP70/THOC1/DLL1/RNF8/HSPD1/ITFG2/SLC15A4/CR1

GOBP_DEFENSE_RESPONSE_TO_GRAM_POSITIVE_BACTERIUM GOBP_DEFENSE_RESPONSE_TO_GRAM_POSITIVE_BACTERIUM GOBP_DEFENSE_RESPONSE_TO_GRAM_POSITIVE_BACTERIUM 77 0.555377359 1.728866553 0.002881844 0.041708234 0.033008499 2123 tags=26%, list=13%, signal=23% DEFA5/DEFA6/DMBT1/PLA2G2A/IL7R/IL27RA/RIPK2/C5AR1/FGR/TLR2/RNASE6/KRT6A/LYZ/GBP2/IL6/MR1/CTSG/ANG/HAVCR2/NOD2

GOBP_LEUKOCYTE_HOMEOSTASIS GOBP_LEUKOCYTE_HOMEOSTASIS GOBP_LEUKOCYTE_HOMEOSTASIS 77 0.56432199 1.756710816 0.002881844 0.041708234 0.033008499 3745 tags=49%, list=22%, signal=39% CXCL6/ANXA1/SLC7A11/SPNS2/TNFRSF17/LYN/ADA/CD74/PDE4B/JAK3/TCIRG1/HIF1A/TNFSF13B/SKIL/CORO1A/DOCK11/GPR183/PIK3CD/JAM3/NKX2-3/ITPKB/IL6/AXL/NCKAP1L/TSC22D3/SIT1/AKT1/STAT5B/RC3H2/SLC15A4/IL2RA/PKN1/HMGB1/CCNB2/BAK1/MPL/ADAM17/FADD

GOBP_HEMATOPOIETIC_STEM_CELL_DIFFERENTIATION GOBP_HEMATOPOIETIC_STEM_CELL_DIFFERENTIATION GOBP_HEMATOPOIETIC_STEM_CELL_DIFFERENTIATION 84 0.564659256 1.774408265 0.002894356 0.041708234 0.033008499 5163 tags=64%, list=30%, signal=45% MEOX1/CBFB/PSME4/PSMB9/LMO2/PSMD14/PSMD12/PSMC2/PSMB2/PSMA3/METTL3/PSMD1/TCF3/PSMA5/SFRP1/PSMD6/PSMB5/PSMC4/BATF/PUS7/PSMB1/PSMA1/ACE/PSME3/PSMA7/PSMB8/PSMC5/PSMB10/PSMB6/PSMA4/LDB1/PSMD13/PSME1/XRCC5/PSMC1/PSMB4/PSMB3/PSMD2/PSMC6/PSMD7/PSMA2/PSMD4/HOXB4/GATA2/PSMD9/EIF2AK2/PSMB7/PRKDC/SRF/EXT1/PSMC3/SETD1A/PSMD8/ITCH

GOBP_INTERFERON_GAMMA_MEDIATED_SIGNALING_PATHWAY GOBP_INTERFERON_GAMMA_MEDIATED_SIGNALING_PATHWAY GOBP_INTERFERON_GAMMA_MEDIATED_SIGNALING_PATHWAY 82 0.559658252 1.754337552 0.002898551 0.041708234 0.033008499 2977 tags=44%, list=18%, signal=36% GBP1/TRIM22/CD44/HLA-DQB1/IRF4/HLA-DRA/FCGR1B/OAS2/SOCS3/VCAM1/PARP9/JAK2/STAT1/HCK/ICAM1/PTAFR/NLRC5/HLA-DQA1/HLA-DPA1/PARP14/IRF1/OAS3/GBP2/PML/IRF9/IFNG/IRF7/CDC37/NMI/HSP90AB1/IRF3/TRIM5/TRIM21/JAK1/HLA-DPB1/TRIM8

GOBP_REGULATION_OF_ALPHA_BETA_T_CELL_DIFFERENTIATION GOBP_REGULATION_OF_ALPHA_BETA_T_CELL_DIFFERENTIATION GOBP_REGULATION_OF_ALPHA_BETA_T_CELL_DIFFERENTIATION 63 0.612077351 1.853144513 0.002919708 0.041708234 0.033008499 2677 tags=46%, list=16%,

signal=39%

ANXA1/ADA/JAK3/IRF4/HLA-DRA/NFKBIZ/PNP/CBFB/RIPK2/TGFBR2/SHH/CD86/SASH3/BCL6/IL12RB1/CCL19/RUNX3/ITPKB/IL23A/LILRB4/IFNG/AP3D1/PRDM1/NCKAP1L/HLX/MALT1/ZBTB16/SYK/IL18

GOBP_REGULATION_OF_HEMATOPOIETIC_STEM_CELL_DIFFERENTIATION

GOBP_REGULATION_OF_HEMATOPOIETIC_STEM_CELL_DIFFERENTIATION

GOBP_REGULATION_OF_HEMATOPOIETIC_STEM_CELL_DIFFERENTIATION 70 0.596332972 1.834396251 0.002923977 0.041708234 0.033008499 5163 tags=66%, list=30%, signal=46%

CBFB/PSME4/PSMB9/LMO2/PSMD14/PSMD12/PSMC2/PSMB2/PSMA3/METTL3/PSMD1/TCF3/PSMA5/PSMD6/PSMB5/PSMC4/PUS7/PSMB1/PSMA1/PSME3/PSMA7/PSMB8/PSMC5/PSMB10/PSMB6/PSMA4/LDB1/PSMD13/PSME1/PSMC1/PSMB4/PSMB3/PSMD2/PSMC6/PSMD7/PSMA2/PSMD4/GATA2/PSMD9/EIF2AK2/PSMB7/PRKDC/PSMC3/SETD1A/PSMD8/ITCH

GOBP_CELL_ADHESION_MEDIATED_BY_INTEGRIN

GOBP_CELL_ADHESION_MEDIATED_BY_INTEGRIN

GOBP_CELL_ADHESION_MEDIATED_BY_INTEGRIN 69 0.592382719 1.818429957 0.002928258 0.041708234 0.033008499 2645 tags=45%, list=16%, signal=38%

CXCL13/PLAU/LYN/ITGA2/ADA/SERPINE1/FBN1/PODXL/ITGA5/ITGB2/ICAM1/PIEZO1/SNAI2/COL16A1/PIK3CG/MUC1/ADAM9/TESC/ITGB1BP1/JAM3/LPXN/ITGAV/LIF/ITGB4/NCKAP1L/SFRP2/NPNT/SWAP70/FERMT1/SYK/ITGA4

GOBP_POSITIVE_REGULATION_OF_RESPONSE_TO_WOUNDING

GOBP_POSITIVE_REGULATION_OF_RESPONSE_TO_WOUNDING

GOBP_POSITIVE_REGULATION_OF_RESPONSE_TO_WOUNDING 64 0.6219418 1.888203165 0.002932551 0.041708234 0.033008499 1174 tags=25%, list=7%, signal=23%

DUOX2/REG3A/ANXA1/CLDN1/THBD/FERMT2/F3/SERPINE1/F2R/XBP1/ST3GAL4/CLEC7A/SMOC2/CXCR4/FLNA/ANO6

GOBP_CHAPERONE_MEDIATED_PROTEIN_FOLDING

GOBP_CHAPERONE_MEDIATED_PROTEIN_FOLDING

GOBP_CHAPERONE_MEDIATED_PROTEIN_FOLDING 56 0.573137182 1.713481339 0.002945508 0.041708234 0.033008499 2058 tags=30%, list=12%, signal=27%

SDF2L1/CD74/P3H1/FKBP5/CLU/PDIA4/HSPH1/HSPA5/HSPA6/HSPA13/ERO1A/PPIB/CRTAP/SGTB/CHORDC1/DNAJC24/CCT2

GOBP_REGULATION_OF_RELEASE_OF_SEQUESTERED_CALCIUM_ION_INTO_CYTOSOL

GOBP_REGULATION_OF_RELEASE_OF_SEQUESTERED_CALCIUM_ION_INTO_CYTOSOL

GOBP_REGULATION_OF_RELEASE_OF_SEQUESTERED_CALCIUM_ION_INTO_CYTOSOL 71 0.556443042 1.711392027 0.002945508 0.041708234 0.033008499 1411 tags=24%, list=8%, signal=22%

CEMIP/CXCL9/CXCL11/CXCL10/LYN/JPH1/APLNR/F2R/PLN/CD19/THY1/DIAPH1/CORO1A/CLIC2/SELENON/GSTO1/PKD2

GOBP_HETEROTYPIC_CELL_CELL_ADHESION GOBP_HETEROTYPIC_CELL_CELL_ADHESION

GOBP_HETEROTYPIC_CELL_CELL_ADHESION 58 0.567151872 1.700447251 0.002949853 0.041708234 0.033008499 1767 tags=31%, list=10%, signal=28%

IL1RN/IL1B/CD44/PTPRC/ITGA5/VCAM1/MADCAM1/ITGB2/THY1/LILRB2/SIRPA/LCK/CD2/JA

M3/CD200/CD47/ITGAV/ITGAX
GOBP_REGULATION_OF_B_CELL_PROLIFERATION
GOBP_REGULATION_OF_B_CELL_PROLIFERATION
GOBP_REGULATION_OF_B_CELL_PROLIFERATION 60 0.59081985 1.774673235 0.002949853 0.041708234 0.033008499 2366 tags=45%, list=14%, signal=39%
MZB1/MNDA/CD38/LYN/ADA/CD74/PTPRC/TNFSF13B/FCGR2B/SASH3/CD300A/TYROBP/CD81/CD40/BCL6/GPR183/FCRL3/MIF/BTK/IKZF3/AHR/TNFRSF4/SLC39A10/NCKAP1L/INPP5D/CD320/BLK
GOBP_REGULATION_OF_CD4_POSITIVE_ALPHA_BETA_T_CELL_ACTIVATION
GOBP_REGULATION_OF_CD4_POSITIVE_ALPHA_BETA_T_CELL_ACTIVATION
GOBP_REGULATION_OF_CD4_POSITIVE_ALPHA_BETA_T_CELL_ACTIVATION 58 0.580133502 1.739369058 0.002949853 0.041708234 0.033008499 2319 tags=40%, list=14%, signal=34%
CD55/ANXA1/CD274/JAK3/IRF4/HLA-DRA/NFKBIZ/CBFB/RIPK2/TGFBR2/CD86/SASH3/CD81/BCL6/IL12RB1/CCL19/RUNX3/TWSG1/IL23A/IFNG/NCKAP1L/HLX/MALT1
GOBP_IRE1_MEDIATED_UNFOLDED_PROTEIN_RESPONSE
GOBP_IRE1_MEDIATED_UNFOLDED_PROTEIN_RESPONSE
GOBP_IRE1_MEDIATED_UNFOLDED_PROTEIN_RESPONSE 62 0.598051816 1.800727128 0.00295421 0.041708234 0.033008499 3700 tags=53%, list=22%, signal=42%
KDELR3/SRPRB/DNAJB11/HYOU1/MYDGF/DNAJB9/XBP1/DNAJC3/FICD/HSPA5/PREB/PDIA6/SRPRA/SSR1/FKBP14/AGR2/WFS1/TLN1/ERN1/YIF1A/SERP1/EDEM1/BFAR/PTPN1/SEC31A/ARFGAP1/EXTL2/PPP2R5B/LMNA/SHC1/BAK1/DDX11/DDRGK1
GOBP_REGULATION_OF_CELLULAR_AMINE_METABOLIC_PROCESS
GOBP_REGULATION_OF_CELLULAR_AMINE_METABOLIC_PROCESS
GOBP_REGULATION_OF_CELLULAR_AMINE_METABOLIC_PROCESS 73 0.558594326 1.722440629 0.002962963 0.041708234 0.033008499 4036 tags=55%, list=24%, signal=42%
SLC7A11/PSME4/PSMB9/ITGB2/ODC1/ITGAM/PSMD14/PSMD12/PSMC2/PSMB2/PSMA3/PSMD1/PSMA5/OAZ3/PSMD6/ATP2B4/PSMB5/PSMC4/PSMB1/PSMA1/PSME3/PARK7/PSMA7/HPRT1/PSMB8/PSMC5/PSMB10/PSMB6/PSMA4/PSMD13/PSME1/DRD4/PSMC1/PSMB4/PSMB3/PSMD2/PSMC6/PSMD7/PSMA2/PSMD4
GOBP_REGULATION_OF_LEUKOCYTE_MEDIATED_CYTOTOXICITY
GOBP_REGULATION_OF_LEUKOCYTE_MEDIATED_CYTOTOXICITY
GOBP_REGULATION_OF_LEUKOCYTE_MEDIATED_CYTOTOXICITY72 0.552979413 1.700462412 0.002962963 0.041708234 0.033008499 3101 tags=39%, list=18%, signal=32%
CXCL6/SERPINB9/CADM1/IL7R/NOS2/AZGP1/HLA-DRA/PTPRC/SERPINB4/FCGR2B/ICAM1/TYROBP/ITGAM/VAV1/IL12RB1/LAG3/IL23A/MR1/LILRB1/HAVCR2/SLAMF6/DNASE1L3/RASGRP1/PVR/ARRB2/AP1G1/STAT5B/IL18RAP
GOBP_REGULATION_OF_B_CELL_MEDIATED_IMMUNITY
GOBP_REGULATION_OF_B_CELL_MEDIATED_IMMUNITY
GOBP_REGULATION_OF_B_CELL_MEDIATED_IMMUNITY 53 0.588679206 1.744109435 0.002967359 0.041708234 0.033008499 2123 tags=28%, list=13%, signal=25%
C4BPB/C4BPA/C3/TGFB1/IL27RA/PTPRC/TNFSF13/MAD2L2/FCGR2B/CD40/BCL6/EXOSC3/BT

K/MLH1/NOD2
GOBP_LIPOPOLYSACCHARIDE_MEDIATED_SIGNALING_PATHWAY
GOBP_LIPOPOLYSACCHARIDE_MEDIATED_SIGNALING_PATHWAY
GOBP_LIPOPOLYSACCHARIDE_MEDIATED_SIGNALING_PATHWAY 55 0.585052974
1.740317148 0.00297619 0.041708234 0.033008499 2779 tags=44%, list=16%, signal=37%
CD55/IL1B/LYN/TGFB1/LY96/RIPK2/CARD16/TLR2/HCK/PTAFR/CCL2/IRAK1/LTF/MTDH/IRAK2/CD180/LY86/MALT1/CD6/BMP6/AKT1/IL18/IRF3/TRIM5
GOBP_POSITIVE_REGULATION_OF_INTERLEUKIN_8_PRODUCTION
GOBP_POSITIVE_REGULATION_OF_INTERLEUKIN_8_PRODUCTION
GOBP_POSITIVE_REGULATION_OF_INTERLEUKIN_8_PRODUCTION 55 0.587315938
1.74704863 0.00297619 0.041708234 0.033008499 2630 tags=38%, list=16%, signal=32%
IL1B/WNT5A/F3/SERPINE1/CD74/F2R/CLEC7A/TLR8/FCN1/TLR2/HYAL2/FFAR2/STAT3/AFAP1L2/CD2/TLR1/IL6/RELA/NOD2/SYK/PARK7
GOBP_REGULATION_OF_SMOOTH_MUSCLE_CONTRACTION
GOBP_REGULATION_OF_SMOOTH_MUSCLE_CONTRACTION
GOBP_REGULATION_OF_SMOOTH_MUSCLE_CONTRACTION 55 0.563241335
1.675435553 0.00297619 0.041708234 0.033008499 1354 tags=29%, list=8%, signal=27%
PROK2/CAV1/ITGA2/ADA/GUCY1A1/F2R/KIT/CALCRL/PTAFR/MYOCD/DOCK4/RGS2/NMU/SPHK1/CNN1/CHRM3
GOBP_ENDODERM_FORMATION GOBP_ENDODERM_FORMATION
GOBP_ENDODERM_FORMATION 51 0.593125252 1.741085747 0.003003003
0.041966758 0.033213099 1602 tags=35%, list=9%, signal=32%
COL12A1/MMP9/COL5A2/MMP2/COL6A1/LAMA3/COL4A2/INHBA/COL5A1/FN1/ITGA5/DUSP4/ITGB2/COL8A1/DKK1/LAMB1/COL7A1/ITGAV
GOBP_FATTY_ACID_BETA_OXIDATION GOBP_FATTY_ACID_BETA_OXIDATION
GOBP_FATTY_ACID_BETA_OXIDATION 72 -0.653644136 -2.260409915 0.003058104
0.042540022 0.033666788 2302 tags=53%, list=14%, signal=46%
TYSND1/ACACB/IVD/ECHS1/ETFBKMT/IRS2/ACAD10/CRAT/SCP2/DECR1/ECHDC2/ETFB/HADHB/ECI1/PPARA/ETFA/ECH1/PPARD/ACAA1/ACOT8/ACOX2/ABCD3/BDH2/HADH/HADHA/ACAA2/CPT2/FABP1/CROT/CPT1A/ACADS/AUH/EHHADH/ACADM/ACAT1/ETFDH/ABCB11/ACOX1
GOBP_POSITIVE_REGULATION_OF_CELL_CYCLE_G1_S_PHASE_TRANSITION
GOBP_POSITIVE_REGULATION_OF_CELL_CYCLE_G1_S_PHASE_TRANSITION
GOBP_POSITIVE_REGULATION_OF_CELL_CYCLE_G1_S_PHASE_TRANSITION 43
0.602907233 1.726093544 0.003081664 0.042540022 0.033666788 4156
tags=53%, list=25%, signal=40%
ANXA1/ADAMTS1/RGCC/HYAL1/FAM83D/CDC6/EZH2/PLCB1/CCND1/LSM10/AIF1/RDX/EIF4G1/PAF1/UBE2E2/AKT1/CCND3/DDRGK1/ADAM17/CCND2/PAGR1/GLI1/CUL4A
GOBP_POSITIVE_REGULATION_OF_IMMUNOGLOBULIN_PRODUCTION
GOBP_POSITIVE_REGULATION_OF_IMMUNOGLOBULIN_PRODUCTION
GOBP_POSITIVE_REGULATION_OF_IMMUNOGLOBULIN_PRODUCTION 43 0.609208554
1.744133915 0.003081664 0.042540022 0.033666788 2089 tags=33%, list=12%,

signal=29%
MZB1/IL33/TGFB1/PTPRC/XBP1/TNFSF13/MAD2L2/SASH3/CD40/EXOSC3/IL6/TNFRSF4/MLH1/RBP4
GOBP_EXCRETION GOBP_EXCRETION GOBP_EXCRETION 57 -0.574308393 -1.89423129 0.00308642 0.042540022 0.033666788 301 tags=16%, list=2%, signal=16% SLC9A3R1/SLC26A3/KCNK5/HMOX1/SCNN1B/AMN/EDN1/GUCA2B/ABCG2
GOBP_NEGATIVE_REGULATION_OF_INTRACELLULAR_PROTEIN_TRANSPORT GOBP_NEGATIVE_REGULATION_OF_INTRACELLULAR_PROTEIN_TRANSPORT GOBP_NEGATIVE_REGULATION_OF_INTRACELLULAR_PROTEIN_TRANSPORT 40 0.606105734 1.710860352 0.00308642 0.042540022 0.033666788 3555 tags=50%, list=21%, signal=40% DERL3/BAG3/ERLEC1/LMAN1/RAB23/UBE2J1/INSIG1/ITGB1BP1/BARD1/RANGAP1/PKIA/APOD/DERL2/LRRK2/FERMT1/PARK7/UFM1/UBE2G2/GDI1/PKIG
GOBP_PEROXISOMAL_TRANSPORT GOBP_PEROXISOMAL_TRANSPORT GOBP_PEROXISOMAL_TRANSPORT 69 -0.574596972 -1.97085747 0.003134796 0.042789278 0.033864053 3537 tags=49%, list=21%, signal=39% ECI2/MLYCD/SLC27A2/PEX2/PEX1/PEX14/IDH1/ZFAND6/AGXT/UBC/TYSND1/DAO/PECR/DDO/CRAT/SCP2/AGPS/HMGCL/PEX13/PEX19/CAT/ECH1/ACAA1/ACOT8/ACOX2/ABCD3/ACOT4/CROT/EHHADH/PHYH/NUDT7/EPHX2/PEX26/ACOX1
GOBP_CHONDROITIN_SULFATE_BIOSYNTHETIC_PROCESS GOBP_CHONDROITIN_SULFATE_BIOSYNTHETIC_PROCESS GOBP_CHONDROITIN_SULFATE_BIOSYNTHETIC_PROCESS 25 0.681172448 1.767302126 0.003184713 0.042789278 0.033864053 1043 tags=44%, list=6%, signal=41% CSGALNACT1/CHST15/BGN/CHST11/CHSY1/VCAN/DSE/DCN/CHPF/B3GALT6/CSGALNACT2
GOBP_INTEGRIN_ACTIVATION GOBP_INTEGRIN_ACTIVATION GOBP_INTEGRIN_ACTIVATION 25 0.665907973 1.727698442 0.003184713 0.042789278 0.033864053 1874 tags=48%, list=11%, signal=43% CXCL13/MZB1/FERMT2/SELP/FN1/PLEK/PIEZO1/COL16A1/KIF14/ITGB1BP1/JAM3/TLN1
GOBP_MATURE_B_CELL_DIFFERENTIATION_INVOLVED_IN_IMMUNE_RESPONSE GOBP_MATURE_B_CELL_DIFFERENTIATION_INVOLVED_IN_IMMUNE_RESPONSE GOBP_MATURE_B_CELL_DIFFERENTIATION_INVOLVED_IN_IMMUNE_RESPONSE 25 0.684184705 1.775117429 0.003184713 0.042789278 0.033864053 1757 tags=48%, list=10%, signal=43% POU2AF1/ADA/LGALS1/XBP1/FCGR2B/PLCG2/MFNG/DOCK11/GPR183/NKX2-3/IL6/ITM2A
GOBP_NEGATIVE_REGULATION_OF_CELL_KILLING GOBP_NEGATIVE_REGULATION_OF_CELL_KILLING GOBP_NEGATIVE_REGULATION_OF_CELL_KILLING 25 0.6677286 1.732422059 0.003184713 0.042789278 0.033864053 2774 tags=40%, list=16%, signal=33% SERPINB9/IL7R/PTPRC/SERPINB4/FCGR2B/KRT6A/LILRB1/HAVCR2/HSP90AB1/ARRB2
GOBP_POSITIVE_REGULATION_OF_COLLAGEN_METABOLIC_PROCESS GOBP_POSITIVE_REGULATION_OF_COLLAGEN_METABOLIC_PROCESS GOBP_POSITIVE_REGULATION_OF_COLLAGEN_METABOLIC_PROCESS 25 0.685941983 1.779676688 0.003184713 0.042789278 0.033864053 2826 tags=56%, list=17%, signal=47%

RGCC/ITGA2/ENG/SERPINB7/TGFB1/F2R/VIM/PDGFRB/CREB3L1/WNT4/SERPINF2/TGFB3/ARRB2/SUCO
GOBP_CALCIUM_ION_IMPORT_INTO_CYTOSOL GOBP_CALCIUM_ION_IMPORT_INTO_CYTOSOL GOBP_CALCIUM_ION_IMPORT_INTO_CYTOSOL 18 0.714930145 1.734050736 0.003189793 0.042789278 0.033864053 1865 tags=33%, list=11%, signal=30% MS4A1/RAMP3/FYN/PRNP/TRPV2/ATP2B4
GOBP_NUCLEOBASE_BIOSYNTHETIC_PROCESS GOBP_NUCLEOBASE_BIOSYNTHETIC_PROCESS GOBP_NUCLEOBASE_BIOSYNTHETIC_PROCESS 18 0.725965477 1.760816743 0.003189793 0.042789278 0.033864053 2694 tags=61%, list=16%, signal=51% CTPS1/ADA/PPAT/SHMT2/GART/PRPS1/UMPS/GMPS/CAD/PAICS/HPRT1
GOBP_PROTEASOMAL_UBIQUITIN_INDEPENDENT_PROTEIN_CATABOLIC_PROCESS GOBP_PROTEASOMAL_UBIQUITIN_INDEPENDENT_PROTEIN_CATABOLIC_PROCESS GOBP_PROTEASOMAL_UBIQUITIN_INDEPENDENT_PROTEIN_CATABOLIC_PROCESS 20 0.718750227 1.786693569 0.003189793 0.042789278 0.033864053 3961 tags=85%, list=23%, signal=65% PSME4/PSMB9/ENC1/PSMB2/PSMA3/PSMA5/PSMB5/PSMB1/PSMA1/PSMA7/PSMB8/PSMB10/PSMB6/PSMA4/PSMB4/PSMB3/PSMA2
GOBP_REGULATION_OF_B_CELL_APOPTOTIC_PROCESS GOBP_REGULATION_OF_B_CELL_APOPTOTIC_PROCESS GOBP_REGULATION_OF_B_CELL_APOPTOTIC_PROCESS 18 0.696154587 1.688510942 0.003189793 0.042789278 0.033864053 2408 tags=50%, list=14%, signal=43% LYN/ADA/CD74/BCL6/AURKB/BTK/SLC39A10/BLK/NOC2L
GOBP_PEROXISOME_ORGANIZATION GOBP_PEROXISOME_ORGANIZATION GOBP_PEROXISOME_ORGANIZATION 80 -0.542991601 -1.911394041 0.003205128 0.042834944 0.033900194 3623 tags=46%, list=21%, signal=37% MAVS/ECI2/MLYCD/SLC27A2/PEX2/PEX1/PEX14/IDH1/ZFAND6/AGXT/UBC/TYSND1/DAO/PECR/DDO/CRAT/SCP2/AGPS/HMGCL/PEX13/TMEM135/PEX19/CAT/ECH1/ACAA1/ACOT8/ACOX2/ABCD3/ACOT4/CROT/PEX11A/EHHADH/PHYH/NUDT7/EPHX2/PEX26/ACOX1
GOBP_MATURATION_OF_LSU_RRNA GOBP_MATURATION_OF_LSU_RRNA GOBP_MATURATION_OF_LSU_RRNA 26 0.655360023 1.710472908 0.003210273 0.042834944 0.033900194 3356 tags=58%, list=20%, signal=46% NOP2/NIFK/MAK16/RPL7L1/GTPBP4/RRP15/FTSJ3/DDX18/NHP2/URB1/NOL9/WDR12/RPF2/PES1/LAS1L
GOBP_ATP_SYNTHESIS_COUPLED_ELECTRON_TRANSPORT GOBP_ATP_SYNTHESIS_COUPLED_ELECTRON_TRANSPORT GOBP_ATP_SYNTHESIS_COUPLED_ELECTRON_TRANSPORT 79 -0.493708639 -1.731795363 0.003225806 0.04292804 0.033973871 4309 tags=61%, list=25%, signal=46% NDUFB11/COX7C/COX6B1/NDUFA6/NDUFB2/SDHAF2/UQCRQ/AFG1L/NDUFA3/NDUFS1/SNCA/UQCR11/NDUFS3/COX10/COX7B/NDUFS4/NDUFA13/SDHA/UQCR10/NDUFS7/CYC1/UQCRFS1/NDUFS2/COX8A/COX6A1/COX4I1/UQCRC2/NDUFB10/NDUFB5/NDUFB9/CYCS/UQCRC1/NDUFA1/GHITM/NDUFB1/COX5A/COX6C/DLD/NDUFV1/COQ9/COX5B/COX15/NDUFA10/PINK1/NDUFA2/NDUFB7/SDHD/NDUFA5
GOBP_MITOCHONDRIAL_RESPIRATORY_CHAIN_COMPLEX_ASSEMBLY

GOBP_MITOCHONDRIAL_RESPIRATORY_CHAIN_COMPLEX_ASSEMBLY
GOBP_MITOCHONDRIAL_RESPIRATORY_CHAIN_COMPLEX_ASSEMBLY 91
-0.462377034 -1.656925882 0.003289474 0.043324698 0.034287792 4309
tags=47%, list=25%, signal=35%
NDUFB11/UQCC1/TFAM/NDUFA6/C12orf73/NDUFB2/SDHAF2/IMMP2L/TACO1/NDUFA3/NDUFS1/NDUFAF6/COX20/NDUFS3/NDUFS4/NDUFA13/NDUFS7/FASTKD3/UQCRFS1/SDHAF3/NDUFS2/ECSIT/COX14/NDUFB10/NDUFB5/AIFM1/COA5/NDUFB9/OMA1/NDUFA1/DMAC2/COA3/NDUFB1/NDUFV1/SMIM20/NDUFA10/NDUFA2/NDUFB7/SLC25A33/NDUFA5/SDHAF4/NUBPL/LYRM7
GOBP_PRIMARY_ALCOHOL_METABOLIC_PROCESS
GOBP_PRIMARY_ALCOHOL_METABOLIC_PROCESS
GOBP_PRIMARY_ALCOHOL_METABOLIC_PROCESS 88 -0.510527684 -1.828557763
0.003289474 0.043324698 0.034287792 2212 tags=36%, list=13%, signal=32%
TTR/ALDH1B1/AKR1C1/ACSS1/DHRS3/PNPLA4/PECR/ADH4/GPD1/CBR4/ALDH1A1/AKR1C2/CYP1A1/AKR1C3/DGKQ/CHKA/AKR1B10/ACSS2/ADH1A/BMP2/GDE1/SULT1A1/NAPEPLD/ADH6/SULT1A2/CLCN2/SULT1B1/BCO1/GDPD1/RETSAT/NAAA/ADH1C
GOBP_ALANINE_TRANSPORT GOBP_ALANINE_TRANSPORT GOBP_ALANINE_TRANSPORT 13
0.749379833 1.672095038 0.003333333 0.043324698 0.034287792 768 tags=38%, list=5%, signal=37% SLC6A14/SLC6A6/SLC38A5/SLC1A4/SLC36A4
GOBP_DERMATAN_SULFATE_METABOLIC_PROCESS
GOBP_DERMATAN_SULFATE_METABOLIC_PROCESS
GOBP_DERMATAN_SULFATE_METABOLIC_PROCESS 13 0.732680646 1.634834057
0.003333333 0.043324698 0.034287792 1702 tags=38%, list=10%, signal=35%
BGN/VCAN/DSE/DCN/CHST12
GOBP_HEMATOPOIETIC_STEM_CELL_HOMEOSTASIS
GOBP_HEMATOPOIETIC_STEM_CELL_HOMEOSTASIS
GOBP_HEMATOPOIETIC_STEM_CELL_HOMEOSTASIS 13 0.735615839 1.641383369
0.003333333 0.043324698 0.034287792 2957 tags=69%, list=17%, signal=57%
FSTL1/TCIRG1/EMCN/FOXA3/MYCT1/ADAR/GLIS2/ARMCX1/UBAP2L
GOBP_MAST_CELL_MIGRATION GOBP_MAST_CELL_MIGRATION
GOBP_MAST_CELL_MIGRATION 13 0.736743454 1.64389942 0.003333333
0.043324698 0.034287792 2956 tags=54%, list=17%, signal=44%
CCL11/RAC2/KIT/VEGFC/PIK3CD/SWAP70/STAT5B
GOBP_NEGATIVE_REGULATION_OF_TYPE_2_IMMUNE_RESPONSE
GOBP_NEGATIVE_REGULATION_OF_TYPE_2_IMMUNE_RESPONSE
GOBP_NEGATIVE_REGULATION_OF_TYPE_2_IMMUNE_RESPONSE 13 0.746183032
1.664961999 0.003333333 0.043324698 0.034287792 889 tags=31%, list=5%, signal=29% ANXA1/CCR2/IL27RA/BCL6
GOBP_POSITIVE_REGULATION_OF_TELOMERASE_RNA_LOCALIZATION_TO_CAJAL_BODY
GOBP_POSITIVE_REGULATION_OF_TELOMERASE_RNA_LOCALIZATION_TO_CAJAL_BODY
GOBP_POSITIVE_REGULATION_OF_TELOMERASE_RNA_LOCALIZATION_TO_CAJAL_BODY 13
0.736683182 1.643764935 0.003333333 0.043324698 0.034287792 2571
tags=77%, list=15%, signal=65%
RUVBL1/CCT3/CCT8/CCT5/CCT2/CCT6A/CCT4/CCT7/NHP2/RUVBL2
GOBP_RESPIRATORY_BURST_INVOLVED_IN_DEFENSE_RESPONSE

GOBP_RESPIRATORY_BURST_INVOLVED_IN_DEFENSE_RESPONSE
GOBP_RESPIRATORY_BURST_INVOLVED_IN_DEFENSE_RESPONSE 13 0.7483524 1.669802521 0.003333333 0.043324698 0.034287792 2039 tags=54%, list=12%, signal=47% SLAMF8/HCK/PIK3CG/PIK3CD/DUSP10/CYBC1/SELENOK
GOBP_COLLAGEN_ACTIVATED_TYROSINE_KINASE_RECEPTOR_SIGNALING_PATHWAY
GOBP_COLLAGEN_ACTIVATED_TYROSINE_KINASE_RECEPTOR_SIGNALING_PATHWAY
GOBP_COLLAGEN_ACTIVATED_TYROSINE_KINASE_RECEPTOR_SIGNALING_PATHWAY 10 0.809795711 1.72333274 0.003389831 0.043532209 0.034452019 2523 tags=60%, list=15%, signal=51% COL4A1/COL1A1/COL4A2/DDR2/COL4A5/SYK
GOBP_PROTEIN_LOCALIZATION_TO_NUCLEAR_BODY
GOBP_PROTEIN_LOCALIZATION_TO_NUCLEAR_BODY
GOBP_PROTEIN_LOCALIZATION_TO_NUCLEAR_BODY 10 0.811160636 1.726237447 0.003389831 0.043532209 0.034452019 2344 tags=70%, list=14%, signal=60% CCT3/CCT8/CCT5/CCT2/CCT6A/CCT4/CCT7
GOBP_POSITIVE_REGULATION_OF_T_CELL_APOPTOTIC_PROCESS
GOBP_POSITIVE_REGULATION_OF_T_CELL_APOPTOTIC_PROCESS
GOBP_POSITIVE_REGULATION_OF_T_CELL_APOPTOTIC_PROCESS 12 0.754887295 1.65388488 0.003395586 0.043532209 0.034452019 113 tags=25%, list=1%, signal=25% CD274/IDO1/WNT5A
GOBP_REGULATION_OF_OSTEOCLAST_DEVELOPMENT
GOBP_REGULATION_OF_OSTEOCLAST_DEVELOPMENT
GOBP_REGULATION_OF_OSTEOCLAST_DEVELOPMENT 12 0.755826389 1.655942345 0.003395586 0.043532209 0.034452019 2549 tags=67%, list=15%, signal=57% FBN1/TYROBP/LTF/CLDN18/SLC9B2/LILRB1/TNFSF11/FBXW7
GOBP_COMPLEMENT_RECEPTOR_MEDIATED_SIGNALING_PATHWAY
GOBP_COMPLEMENT_RECEPTOR_MEDIATED_SIGNALING_PATHWAY
GOBP_COMPLEMENT_RECEPTOR_MEDIATED_SIGNALING_PATHWAY 11 0.785223143 1.686561876 0.003401361 0.043532209 0.034452019 2346 tags=64%, list=14%, signal=55% FPR1/C5AR1/FPR3/C3AR1/CR2/FPR2/CMKLR1
GOBP_G_PROTEIN_COUPLED_RECEPTOR_SIGNALING_PATHWAY_INVOLVED_IN_HEART_PROCESS
GOBP_G_PROTEIN_COUPLED_RECEPTOR_SIGNALING_PATHWAY_INVOLVED_IN_HEART_PROCESS
GOBP_G_PROTEIN_COUPLED_RECEPTOR_SIGNALING_PATHWAY_INVOLVED_IN_HEART_PROCESS 11 0.796222146 1.710186369 0.003401361 0.043532209 0.034452019 1865 tags=64%, list=11%, signal=57% CAV1/PDE4B/RAMP3/PLN/GNAI2/RGS2/ATP2B4
GOBP_METAL_ION_TRANSPORT GOBP_METAL_ION_TRANSPORT
GOBP_METAL_ION_TRANSPORT 458 0.364762372 1.384993965 0.003428571 0.043727611 0.034606663 2142 tags=20%, list=13%, signal=18% GJA1/CEMIP/CXCL9/CXCL11/KCND3/CXCL10/CAV1/MS4A1/KCNE3/LYN/PDE4B/GEM/TRPA1/STC1/MCUB/JPH1/APLNR/PTPRC/F2R/CCR1/RAMP3/HOMER1/PLN/KCNN4/CCR7/CD19/FYN/ANXA6/CCL4/TMEM165/THY1/LILRB2/CXCR4/SESTD1/CALCRL/TRIM27/P2RX5/TUSC3/GNAI2/ICAM1/CCL2/PDGFRB/SLC41A1/PLCG2/SLC39A6/DIAPH1/TMEM163/CORO1A/GRAMD2A/GNB5/SEC61A1/SEMG1/CLIC2/ERO1A/ATP2A3/SELENON/ANO6/PIK3CG/CD84/LCK/SLC24A3/RGS4/CCL19/PRKCB/GSTO1/SPINK1/FCRL3/CYP27B1/PKD2/ORAI2/PRNP/TRPV2/WFS1/ANXA2/ORAI1/IBTK/ITGAV/G

JA4/PML/SLC30A7/LILRB1/ATP2B4/SLC39A10/ATP2A2/BSPRY/CYSLTR1/CCR5/SELENOK/PPP3CA/P2RX7/TRPC6
GOBP_REGULATION_OF_PH GOBP_REGULATION_OF_PH GOBP_REGULATION_OF_PH 96 -0.422415951 -1.5297902 0.003448276 0.043727611 0.034606663 2851 tags=27%, list=17%, signal=23% CHP1/RAB20/CCKBR/CLN3/MAPK3/CLN5/MAPK1/SLC9A8/ATP5F1B/RAB7A/ATP6V0D2/CA7/BCL2/CCDC115/CFTR/LACC1/TTPA/CA2/SLC26A3/SLC9A2/PDK2/AQP11/SLC9A3/PDK4/SLC4A4/EDN1
GOBP_RESPIRATORY_ELECTRON_TRANSPORT_CHAIN GOBP_RESPIRATORY_ELECTRON_TRANSPORT_CHAIN GOBP_RESPIRATORY_ELECTRON_TRANSPORT_CHAIN 96 -0.516493549 -1.870494634 0.003448276 0.043727611 0.034606663 4075 tags=58%, list=24%, signal=45% COX6B1/NDUFA6/NDUFB2/SDHAF2/UQCRQ/SLC25A13/IMMP2L/AFG1L/NDUFA3/NDUFS1/SNCA/UQCR11/NDUFS3/COX10/COX7B/NDUFS4/NDUFA13/SDHA/UQCR10/NDUFS7/CYC1/UQCRFS1/NDUFS2/COX8A/ETFRF1/COX6A1/COX4I1/UQCRC2/NDUFB10/NDUFB5/NDUFB9/CYCS/UQCRC1/NDUFA1/GHITM/NDUFB1/COX5A/COX6C/GPD1/SLC25A12/DLD/NDUFV1/COQ9/COX5B/SDHB/COX15/NDUFA10/PINK1/ETFB/NDUFA2/NDUFB7/ETFA/SDHD/NDUFA5/ETFDH/PPARGC1A
GOBP_RESPONSE_TO_OXIDATIVE_STRESS GOBP_RESPONSE_TO_OXIDATIVE_STRESS GOBP_RESPONSE_TO_OXIDATIVE_STRESS 409 0.374632492 1.412125186 0.003456221 0.043727611 0.034606663 3352 tags=30%, list=20%, signal=25% MMP3/DUOX2/VNN1/GPX8/ANXA1/RBPMS/NCF2/COL1A1/MMP9/SLC7A11/CD38/PXDN/MMP2/HYAL1/ALOX5/ADA/GPX7/GUCY1B1/HBB/MCTP1/PRDX4/TRPA1/FUT8/BNIP3/S100A7/HIF1A/PTGS2/UCP2/PDGFRA/CCR7/FYN/JAK2/MAP3K5/PTGS1/PDGFRB/HYAL2/MICB/MET/SOD3/SIRPA/CYGB/KLF2/EIF2S1/P4HB/FBLN5/GPX2/NME5/ERO1A/MELK/SELENON/EZH2/ECT2/CCL19/ADAM9/SPHK1/HNRNPD/STX2/NCOA7/FANCD2/ATP13A2/PKD2/SRXN1/PRNP/PDK1/BTK/CFLAR/PML/ATOX1/IL6/CRYAB/AXL/CDK2/ALDH3B1/PARP1/SCARA3/NCF4/AIF1/ERN1/PSMB5/APOD/ATP2A2/MPV17/VRK2/SELENOK/RELA/SETX/TRPC6/LRRK2/ETV5/SOD2/AQP1/FXN/GPX1/GLRX2/FBXW7/ETS1/ATF4/AKT1/PYCR1/PARK7/ATP7A/CYBB/TRA2B/APOE/NR4A3/CCNA2/PNKP/LDHA/GSTP1/LONP1/IL18RAP/SFPQ/GCLM/PNPT1/TMEM161A/SLC23A2/SELENOS/NUDT1/TRPM2/PRKD1/NOX1/HDAC2/AGAP3
GOBP_MYELOID_CELL_DIFFERENTIATION GOBP_MYELOID_CELL_DIFFERENTIATION GOBP_MYELOID_CELL_DIFFERENTIATION 385 0.375326057 1.412596513 0.003460208 0.043727611 0.034606663 2561 tags=26%, list=15%, signal=23% MMP9/LYN/FBN1/HCLS1/CD74/IFI16/TGFB1/PF4/JAK3/EVI2B/IRF4/INHBA/NME1/CBFB/TCIRG1/HIF1A/CCR1/SBNO2/KIT/TGFBR2/CCR7/CSF3R/GPR137B/JAK2/CEBPB/STAT1/PIAS3/ADGRF5/TLR2/MYL9/LOX/MAFB/TYROBP/CD81/ACTN1/SNX10/LYAR/BCL6/RASSF2/EFNA2/FLI1/KLF2/LEF1/LTF/FASN/STAT3/TSPAN2/MEIS1/CLDN18/CSF1R/GPR183/CCL19/PRKCB/TESC/PIK3CD/BATF2/PIP4K2A/NKX2-3/NRROS/SLC9B2/ANXA2/PRMT1/ITPKB/TFE3/LIF/IL23A/SRP54/AGO3/PML/CD109/CASP8/LILRB4/SFRP1/PARP1/LILRB1/IFNG/JAG1/LILRB3/CREB1/BATF3/BATF/PABPC4/GPR171/JMJD6/SMAD5/TMEM64/NCKAP1L/TNFSF11/INPP5D/NBEAL2/IRF7/PAF1/CD4/HAX1/ZBTB16/FSTL3/ADAR/NCAPG2/CDK5RAP3/FBXW7/ETS1
GOBP_POSITIVE_REGULATION_OF_PROTEOLYSIS GOBP_POSITIVE_REGULATION_OF_PROTEOLYSIS GOBP_POSITIVE_REGULATION_OF_PROTEOLYSIS 342 0.391676403 1.461643993

0.003525264 0.044132653 0.03492722 3704 tags=34%, list=22%, signal=27% S100A8/S100A9/IL1B/TRIB2/CAV1/SERPINB3/ROBO1/CASP1/IL33/LYN/CTSH/F3/CCN1/PCOLCE/IFI16/AIM2/BAG2/CLU/F2R/RIPK2/ECSCR/FN1/BOK/ANTXR1/PSME4/CLEC7A/FYN/JAK2/DLC1/RNF144B/MAP3K5/CDC20/TNFRSF10A/LAPTM5/DAB2/AKIRIN2/PSMD14/ASPH/STAT3/VCP/XDH/SEMG1/ENO1/CASP10/ATP2A3/TNFSF15/CSNK1E/TNFRSF10B/CTSC/LCK/PSMC2/ADAM9/AURKA/TNFRSF1B/PLK1/BID/DAP/CFLAR/PML/CASP8/ADAM8/ADRM1/APH1B/NLRP2/IFNG/PSMC4/PLK2/TIMM17A/SOX7/NRDC/FURIN/SFRP2/RNF19B/MALT1/OGT/LRRK2/F12/PSME3/SYK/FBXW7/AKT1/HIP1/TANK/HSPBP1/DVL1/FBLN1/APOE/PPM1F/USP13/CBFA2T3/SOCS4/SMURF1/PRR7/HSPD1/PRSS22/RAD23A/NLRC4/PSME1/HERPUD1/PRICKLE1/HDAC2/HMGB1/CR1/FBXO22/EPHA4/FGFR4/BAK1/PSMC1/MDM2/MMP14/RHBDD1/SART3/FASLG/DDRGK1/USP5/HPN

GOBP_RESPONSE_TO_OXYGEN_LEVELS GOBP_RESPONSE_TO_OXYGEN_LEVELS GOBP_RESPONSE_TO_OXYGEN_LEVELS 362 0.3785757 1.415898893 0.003537736 0.044132653 0.03492722 3478 tags=34%, list=21%, signal=28% REG1A/AQP3/LOXL2/PLAU/COL1A1/CD38/RGCC/CAV1/CASP1/MMP2/NOS2/ITGA2/ADA/PKM/HYOU1/EDNRA/TIGAR/STC1/PDPN/BNIP3/HIF1A/PTGS2/PSME4/VCAM1/PSMB9/CCNB1/UCP2/CARD16/TWIST1/TGFBR2/CXCR4/CPEB4/POSTN/DDIT4/TLR2/ICAM1/PDGFRB/PLOD1/SOD3/IRAK1/MYOCD/VEGFC/P4HB/AGTRAP/TEK/PSMD14/ERO1A/ENO1/PAM/PSMD12/PSMC2/APOLD1/HILPDA/PSMB2/PSMA3/PSMD1/HSP90B1/HK2/PDK1/RORA/CFLAR/ADAM15/PSMA5/AJUBA/PML/ADAM8/CRYAB/PTGIS/TBL2/SFRP1/PSMD6/KCNMB1/PSMB5/PSMC4/CREB1/ANG/TRPC6/PSMB1/MTHFR/RAMP2/PSMA1/CDK4/SUV39H2/VASN/SOD2/AQP1/TGFB3/PSME3/SUV39H1/ELOC/ETS1/ANGPTL4/ANGPT2/ATF4/AKT1/POLB/PSMA7/PSMB8/PSMC5/ATP7A/CYBB/SLC2A1/CBFA2T3/CCNA2/SCFD1/CD34/PSMB10/PSMB6/LDHA/LONP1/LMNA/PSMA4/AHCY/PSMD13/PSME1/NOX1/PGK1/HDAC2/USF1/WTIP/ALKBH5/POLG/ITPR1/PSMC1

GOBP_FATTY_ACID_CATABOLIC_PROCESS GOBP_FATTY_ACID_CATABOLIC_PROCESS GOBP_FATTY_ACID_CATABOLIC_PROCESS 103 -0.640809321 -2.378267531 0.003546099 0.044132653 0.03492722 2302 tags=47%, list=14%, signal=41% IVD/ECHS1/ETFBKMT/ADTRP/IRS2/ACAD10/PECR/CRAT/FAAH/SCP2/DECR1/ECHDC2/ETFB/PEX13/HADHB/ACBD5/LPIN2/ECI1/PPARA/PCCA/ETFA/PCK2/ECH1/PPARD/ACAA1/ACOT8/ACOX2/ABCD3/BDH2/HADH/HADHA/ACAA2/ABHD3/CPT2/FABP1/CROT/CPT1A/ACADS/AUH/EHHADH/PHYH/NUDT7/ACADM/ACAT1/ETFDH/ABCB11/ACOX1/PCK1

GOBP_SKIN_DEVELOPMENT GOBP_SKIN_DEVELOPMENT GOBP_SKIN_DEVELOPMENT 317 0.40386487 1.499704593 0.003550296 0.044132653 0.03492722 2167 tags=18%, list=13%, signal=16% REG3A/PI3/IGFBP5/ANXA1/AQP3/SPINK5/CTSK/WNT5A/CLDN1/FOXQ1/COL1A2/COL1A1/APCDD1/CDH3/COL5A2/COL3A1/ITGA2/INHBA/DSG3/COL5A1/LDB2/CBFB/GAL/S100A7/SHH/CSTA/TRIM16/MET/BCR/GJB3/ABCA12/PALLD/KRT6A/SPRR1B/ADAM9/ABCB6/CYP27B1/ITGA6/HPSE/PRKCH/FST/SCEL/KAZN/CD109/KLK12/CTSL/ZBED2/LAMA5/FGF7/MAP2K1/JAG1/ITGB4/NSDHL/RELA/LTB/KRT23/FURIN

GOBP_UROGENITAL_SYSTEM_DEVELOPMENT GOBP_UROGENITAL_SYSTEM_DEVELOPMENT GOBP_UROGENITAL_SYSTEM_DEVELOPMENT 314 0.395639151 1.468028353 0.003550296 0.044132653 0.03492722 2623 tags=23%, list=15%, signal=20% SERPINB5/TNC/GREM1/COL4A1/ANXA1/PECAM1/AGT/WNT5A/MMP9/NID1/ADAMTS1/BASP1/CTSH/ASS1/SERPINB7/FBN1/SULF1/PODXL/PDGFRA/SHH/PPAT/DCN/STAT1/FOXF1/ACTA2/PDGFRB/SULF2/HYAL2/MYOCD/SERPINF1/PCSK9/ITGA8/TEK/SEC61A1/ARL3/WWTR1/BICC1/WDR7

7/MME/AKR1B1/TACSTD2/KCNJ8/PKD2/LAMB2/WFS1/CFLAR/DCHS1/LIF/PLXND1/AHI1/SFRP1/LAMA5/NUP85/JAG1/MPV17/JMJD6/SMAD5/GPR4/WNT4/HOXB13/RBP4/TGFBR1/NPNT/SALL1/ACE/LRRK2/ZBTB16/FSTL3/GLIS2/ARID5B/ANGPT2/BMP6/DLL1
GOBP_LIPID_OXIDATIONGOBP_LIPID_OXIDATIONGOBP_LIPID_OXIDATION107 -0.611305445 -2.282757569 0.003558719 0.044132653 0.03492722 2591 tags=47%, list=15%, signal=40%
TYSND1/ACACB/CYP4V2/ADH5/IVD/ECHS1/ETFBKMT/IRS2/ACAD10/PECR/ADH4/CRAT/SCP2/DECR1/ECHDC2/PRKAG2/ETFB/PEX13/HADHB/PRKAA1/ECI1/PPARA/ETFA/ECH1/PPARD/ACAA1/ACOT8/ACOX2/ABCD3/BDH2/HADH/HADHA/ACAA2/CPT2/FABP1/CROT/CPT1A/ACADS/AUH/EHHADH/PHYH/ACADM/ACAT1/ETFDH/ABCB11/PDK4/PPARG/ACOX1/PPARGC1A/APPL2
GOBP_MONOCARBOXYLIC_ACID_CATABOLIC_PROCESS GOBP_MONOCARBOXYLIC_ACID_CATABOLIC_PROCESS GOBP_MONOCARBOXYLIC_ACID_CATABOLIC_PROCESS 127 -0.599888985 -2.287096317 0.003571429 0.044132653 0.03492722 2752 tags=46%, list=16%, signal=39%
ACAT2/AGXT/MMUT/TYSND1/ACACB/FAH/IVD/ECHS1/ETFBKMT/ADTRP/IRS2/ACAD10/PECR/CYP4F3/CRAT/FAAH/SCP2/DECR1/ECHDC2/ETFB/PEX13/HADHB/ACBD5/LPIN2/ECI1/PPARA/PCCA/ETFA/PCK2/ECH1/PPARD/ACAA1/ACOT8/ACOX2/ABCD3/BDH2/HADH/CRYL1/HADHA/ACAA2/ABHD3/CYP4F12/CPT2/LDHD/FABP1/CROT/CYP4F2/CPT1A/ACADS/AUH/EHHADH/PHYH/NUDT7/ACADM/ACAT1/ETFDH/ABCB11/ACOX1/PCK1
GOBP_OXIDATIVE_PHOSPHORYLATION GOBP_OXIDATIVE_PHOSPHORYLATION GOBP_OXIDATIVE_PHOSPHORYLATION 124 -0.428589624 -1.622769484 0.003571429 0.044132653 0.03492722 4075 tags=52%, list=24%, signal=39%
COX6B1/ATP5F1A/NDUFA6/COX7A2/NDUFB2/SDHAF2/ATP5MC1/UQCRQ/ATP5PO/AFG1L/ATP5F1E/NDUFA3/NDUFS1/SNCA/UQCR11/NDUFS3/COX10/COX7B/NDUFS4/NDUFA13/SDHA/UQCR10/NDUFS7/CYC1/ATP5F1D/ATP5PB/UQCRFS1/NDUFS2/COX8A/MSH2/COX6A1/COX4I1/UQCRC2/NDUFB10/ATP5MC2/NDUFB5/ATP5PF/ATP5F1B/NDUFB9/CYCS/AK4/UQCRC1/NDUFA1/GHITM/ATP5MC3/NDUFB1/COX5A/COX6C/DLD/NDUFV1/COQ9/COX5B/COX15/NDUFA10/PINK1/CHCHD10/NDUFA2/NDUFB7/NIPSNAP2/SLC25A33/SDHD/NDUFA5/DMAC2L/SLC25A23
GOBP_POLYOL_METABOLIC_PROCESS GOBP_POLYOL_METABOLIC_PROCESS GOBP_POLYOL_METABOLIC_PROCESS 123 -0.435992343 -1.649766513 0.003571429 0.044132653 0.03492722 1419 tags=21%, list=8%, signal=20%
PLCD3/SPR/SPHK2/PCK2/IMPA2/INPP5J/SGPP2/INPP4B/PPIP5K2/SPTLC3/IMPA1/MINPP1/OCRL/PLCH1/PCBD2/PLCD1/MOGAT2/ITPKA/IP6K2/PLCE1/BPNT1/CYP27A1/PLPP1/P2RY1/NAAA/PCK1
GOBP_DNA_REPLICATION GOBP_DNA_REPLICATION GOBP_DNA_REPLICATION 272 0.417122482 1.530115686 0.00362757 0.044716256 0.035389092 3587 tags=34%, list=21%, signal=27%
S100A11/RRM2/FEN1/NOC3L/DDX21/SLFN11/POLD3/SAMHD1/NFIC/DONSON/RTEL1/BCL6/HMGA1/BLM/MCM6/DNAJC2/PCLAF/CDC45/DTD1/CDK2AP1/CDC6/CDK1/TBRG1/MCM10/BARD1/RBMS1/ATR/TWNK/TOP1/TIPIN/EXO1/GTPBP4/DACH1/RFC3/GINS3/ZPR1/CDC7/CDK2/TIMELESS/SSRP1/POLE2/ORC6/DBF4/NBN/CHTF18/MCM3/RBBP8/GRWD1/GINS1/RRM1/MCM5/SSBP1/CHEK1/RAD1/RFC4/CLSPN/CDT1/POLD2/PTMS/THOC1/POLB/MCM7/RECQL/RNASEH2A/PIF1/CCNA2/TTF1/PNKP/ORC5/HRAS/EME1/NFIB/GINS2/FBXO5/TNFAIP1/ALYREF/CACYBP/BAZ1A/UPF

1/BRCA1/CENPX/POLA2/NAP1L1/FBH1/POLG/CHAF1A/MCM2/DSCC1/SUPT16H/DTL/ZNF830/DDX23
GOBP_PROTEIN_MATURATION GOBP_PROTEIN_MATURATION
GOBP_PROTEIN_MATURATION 278 0.416332713 1.526487838 0.003649635 0.044877988 0.035517088 2574 tags=26%, list=15%, signal=22%
DUOXA2/PLAU/PCSK1/BACE2/CASP1/SERPINE2/CTSH/F3/SERPINE1/C1R/DNAJB11/GAS1/IFI16/SRGN/PRDX4/BAG2/CPA3/RIPK2/CPXM1/SEC11C/CARD16/SHH/GALNT2/HM13/ADAM19/PLAT/SPCS3/PCSK9/CASP4/CST7/ASPH/SERPINH1/LDLRAD3/CALR/ERO1A/ENO1/YAE1/MME/GLRX3/TESC/NLRP7/PRNP/CTSE/WFS1/ECE2/CASP8/ADAM8/LMF2/CTSL/C1RL/PARP1/APH1B/AOPEP/NAA15/CTSG/SCG5/BCL2L12/SPCS2/TIMM17A/CHAC1/FURIN/KLK6/SERPINF2/OGT/ACE/LRRK2/F12/FXN/TMEM208/BACE1/PITRM1
GOBP_HOMEOSTASIS_OF_NUMBER_OF_CELLS GOBP_HOMEOSTASIS_OF_NUMBER_OF_CELLS
GOBP_HOMEOSTASIS_OF_NUMBER_OF_CELLS 238 0.427025748 1.544979876 0.003663004 0.044877988 0.035517088 3745 tags=35%, list=22%, signal=28%
CXCL6/PLA2G2A/ANXA1/FSTL1/SLC7A11/SPNS2/IL7R/TNFRSF17/LYN/ADA/HCLS1/CD74/PDE4B/JAK3/INHBA/F2R/TCIRG1/HIF1A/TNFSF13B/KIT/CCR7/JAK2/STAT1/ADGRF5/SASH3/EMCN/MAFB/SKIL/LYAR/CORO1A/BCL6/RASSF2/KLF2/STAT3/EZH2/DOCK11/GPR183/LIPA/PIK3CD/JAM3/NKX2-3/PRMT1/ITPKB/AKT3/FOXA3/IL6/AXL/JMJD6/SMAD5/NCKAP1L/INPP5D/TSC22D3/SIT1/MYCT1/ADAR/NCAPG2/CDK5RAP3/GLIS2/ETS1/AKT1/POLB/ARMCX1/STAT5B/UBAP2L/PTPN11/RC3H2/SPI1/LDB1/SLC15A4/IL2RA/PKN1/HMGB1/CCNB2/CARD11/NOS3/BAK1/G6PD/ZFP36L1/MPL/IKZF1/RCOR1/ADAM17/SOX9/FADD
GOBP_CHLORIDE_TRANSPORT GOBP_CHLORIDE_TRANSPORT
GOBP_CHLORIDE_TRANSPORT 105 -0.470485842 -1.737441717 0.003676471 0.044877988 0.035517088 1710 tags=19%, list=10%, signal=17%
PCYOX1/GLRA2/CA7/ANO10/ANO5/CFTR/ATP8B1/BEST4/FXYD3/CLDN4/CLIC5/CLCN2/BEST2/PRKG2/SLC26A3/CLCA4/GABRA2/SLC1A1/ABCB1/SLC26A2
GOBP_REGULATION_OF_PEPTIDYL_TYROSINE_PHOSPHORYLATION
GOBP_REGULATION_OF_PEPTIDYL_TYROSINE_PHOSPHORYLATION
GOBP_REGULATION_OF_PEPTIDYL_TYROSINE_PHOSPHORYLATION 243 0.426525025 1.545079999 0.003676471 0.044877988 0.035517088 2677 tags=28%, list=16%, signal=24%
GREM1/PECAM1/AGT/CAV1/LYN/LRP8/CD44/HCLS1/CD74/TGFB1/SH3BP5/PTPRC/RIPK2/SOCS3/ITGA5/CLEC7A/ENPP2/PARP9/KIT/SAMSN1/FYN/ITGB2/JAK2/THY1/ICAM1/CD300A/HYAL2/SEMA4D/CD81/CD40/PARP14/GPRC5B/STAT3/BANK1/IL6ST/CSF1R/AFAP1L2/SPINK1/NRP1/PRNP/MIF/GPRC5A/IBTK/ACVR1/DUSP22/LIF/IL23A/IL6/TSLP/LILRB4/ARHGEF2/SFRP1/FGF7/IFNG/NOD2/SFRP2/PDGFC/ACE/PTPN1/CD4/HAX1/IL24/SYK/NCAPG2/FBXW7/BMP6/TNFRSF18/IL18
GOBP_CONNECTIVE_TISSUE_DEVELOPMENT GOBP_CONNECTIVE_TISSUE_DEVELOPMENT
GOBP_CONNECTIVE_TISSUE_DEVELOPMENT 230 0.439384373 1.580536759 0.003703704 0.044959494 0.035581594 1184 tags=19%, list=7%, signal=18%
CHI3L1/TIMP1/GREM1/MGP/LOXL2/TGFBI/WNT5A/PRRX1/COL1A1/CREB3L2/CSGALNACT1/HYAL1/CCN1/SERPINB7/CD44/TGFB1/BGN/SULF1/STC1/SELENOM/CHST11/COL5A1/CHSY1/LUM/ECM1/HIF1A/RUNX2/XBP1/TGFBR2/ANXA6/SH3PXD2B/CHRDL2/ACTA2/LOX/PDGFRB/SULF2/HYAL2/OXCT1/SNAI2/SERPINH1/EFEMP1/PLAAT3/RFLNB
GOBP_PROTEIN_PROCESSING GOBP_PROTEIN_PROCESSING

GOBP_PROTEIN_PROCESSING 211 0.43068642 1.54074718 0.003726708 0.044959494 0.035581594 2574 tags=27%, list=15%, signal=24% PLAU/PCSK1/BACE2/CASP1/SERPINE2/CTSH/F3/SERPINE1/C1R/GAS1/IFI16/SRGN/BAG2/CPA3/RIPK2/CPXM1/SEC11C/CARD16/SHH/HM13/ADAM19/PLAT/SPCS3/PCSK9/CASP4/CST7/ASPH/LDLRAD3/ENO1/MME/NLRP7/PRNP/CTSE/ECE2/CASP8/ADAM8/CTSL/C1RL/PARP1/APH1B/AOPEP/CTSG/SCG5/BCL2L12/SPCS2/TIMM17A/CHAC1/FURIN/KLK6/SERPINF2/OGT/ACE/LRRK2/F12/FXN/TMEM208/BACE1/PITRM1

GOBP_RNA_LOCALIZATION GOBP_RNA_LOCALIZATION GOBP_RNA_LOCALIZATION 214 0.431768177 1.547652041 0.003726708 0.044959494 0.035581594 4717 tags=50%, list=28%, signal=37% RFTN1/QKI/TGFBR2/HHEX/SEC13/MAGOHB/XPOT/RRS1/RUVBL1/DDX39A/NOP58/MX2/PABPN1/NUP62/ABCE1/EXOSC3/RAN/ATR/DDX19A/NUP98/CCT3/IGF2BP3/NUP85/CCT8/NXT1/CETN2/POLR2D/CCT5/SRSF7/CCT2/MCM3AP/SDAD1/NUP93/CCT6A/NUP210/CCT4/CCT7/RAE1/NHP2/NUP37/SMG7/LTV1/RUVBL2/NSUN2/THOC1/NPM1/NUP62CL/NXF3/NUP58/CPSF3/PIH1D1/SFPQ/PNPT1/ALYREF/NUP205/UPF1/SLBP/EIF4A3/ALKBH5/UPF2/AAAS/IWS1/NUP50/RBM8A/FYTTD1/SRSF1/EXOSC10/ZFP36L1/CHTOP/LSG1/RBM15B/NOP9/NUP35/NUP155/NUP107/SUPT6H/DHX36/SRSF3/AGFG1/POM121L12/RANBP2/NOP10/XPO5/MRPL18/NOL6/SRSF4/SRSF6/EIF5A2/NDC1/NUP188/NUP88/THOC7/THOC5/NUP54/U2AF1/NXF1/ATM/HNRNPU/ENY2/CKAP5/RTRAF/SRRM1/EXOSC2/NUP214/CPSF6/SYMPK/XPO1

GOBP_REGULATION_OF_CELL_CYCLE_G2_M_PHASE_TRANSITION GOBP_REGULATION_OF_CELL_CYCLE_G2_M_PHASE_TRANSITION GOBP_REGULATION_OF_CELL_CYCLE_G2_M_PHASE_TRANSITION 209 0.442934212 1.581011691 0.00373599 0.044959494 0.035581594 4461 tags=49%, list=26%, signal=36% CDC25B/PRKAR2B/PSME4/PSMB9/CCNB1/NABP1/TUBG1/DONSON/CDC25A/BLM/CEP78/PSMD14/CEP164/CSNK1E/KIF14/CDC6/PSMD12/CDK1/PSMC2/TUBA4A/PSMB2/AURKB/PSMA3/AURKA/ODF2/PSMD1/TUBB/CCND1/PLK1/PSMA5/TPX2/AVEN/FHL1/RCC2/CDC7/CDK2/PSMD6/PKIA/PSMB5/PSMC4/CETN2/NBN/CEP135/ATF5/PSMB1/PSMA1/CDK4/PINX1/CHEK1/UIMC1/RAD51C/PSME3/CLSPN/CDK5RAP3/NABP2/NPM1/CUL1/PSMA7/PSMB8/NDE1/PSMC5/TUBA1A/PSMB10/HSP90AA1/PSMB6/PSMA4/FBXO5/INTS3/PSMD13/PSME1/VPS4A/YWHAG/BRCA1/USP47/RINT1/PSMC1/DTL/ZNF830/NEDD1/FBXL7/PSMB4/PLK4/CENPF/PSMB3/PSMD2/HAUS7/PSMC6/PSMD7/PSMA2/HAUS1/MRE11/PSMD4/NEK2/HAUS2/PSMD9/SMARCD3/HMMR/MRNIP/WNT10B/GTSE1/ATM/CKAP5

GOBP_REGULATION_OF_CYSTEINE_TYPE_ENDOPEPTIDASE_ACTIVITY GOBP_REGULATION_OF_CYSTEINE_TYPE_ENDOPEPTIDASE_ACTIVITY GOBP_REGULATION_OF_CYSTEINE_TYPE_ENDOPEPTIDASE_ACTIVITY216 0.418579868 1.501559079 0.003745318 0.044959494 0.035581594 2895 tags=29%, list=17%, signal=24% S100A8/S100A9/LAMP3/MMP9/SERPINB9/ROBO1/CASP1/CTSH/F3/CCN1/IFI6/CD44/IFI16/AIM2/BIRC3/TNFAIP8/F2R/RIPK2/BOK/CLEC7A/PSMB9/CARD16/FYN/JAK2/DLC1/MAP3K5/CD27/TNFRSF10A/CST7/LAPTM5/LTF/ASPH/VCP/XDH/CASP10/ATP2A3/TNFSF15/TNFRSF10B/LCK/BID/DAP/CFLAR/PML/CASP8/CRYAB/NLRP2/BCL2L12/SOX7/SFRP2/DNAJB6/MALT1/AQP1/GPX1/SYK/MICAL1/AKT1/PARK7/HIP1/TRIAP1/ARRB2/PPM1F/PLAUR

GOBP_NEGATIVE_REGULATION_OF_APOPTOTIC_SIGNALING_PATHWAY

GOBP_NEGATIVE_REGULATION_OF_APOPTOTIC_SIGNALING_PATHWAY
GOBP_NEGATIVE_REGULATION_OF_APOPTOTIC_SIGNALING_PATHWAY 208 0.420595333 1.499310079 0.003754693 0.044959494 0.035581594 3794 tags=38%, list=22%, signal=29%
VNN1/IL1B/MMP9/PEA15/SERPINE1/IFI6/CD44/HYOU1/CD74/PF4/CLU/HIF1A/XBP1/BOK/FYN/GNAI2/ICAM1/TNFRSF10A/CREB3L1/SNAI2/IL1A/DNAJA1/NME5/ENO1/TNFRSF10B/MUC1/DDIAS/EYA3/BID/ITGA6/MIF/GRINA/CFLAR/ACVR1/ITGAV/ITPRIP/CASP8/CREB3/SLC35F6/ARHGEF2/BCL2L12/RELA/WNT4/TGFBR1/FIGNL1/SFRP2/LRRK2/PTPN1/NOC2L/SOD2/FXN/GPX1/PSME3/AKT1/PARK7/TRIAP1/ARRB2/PLAUR/CCAR2/YBX3/CTTN/GSTP1/AATF/PIH1D1/GCLM/TMEM161A/SELENOS/HERPUD1/BRCA1/CX3CL1/USP47/NOS3/PPIF/MDM2/TRAP1/FASLG/FADD/IL19
GOBP_REGULATION_OF_CHEMOTAXIS GOBP_REGULATION_OF_CHEMOTAXIS
GOBP_REGULATION_OF_CHEMOTAXIS 207 0.445644488 1.589946337 0.003764115 0.044959494 0.035581594 2575 tags=33%, list=15%, signal=29%
GREM1/CXCL13/CXCL8/CXCL10/WNT5A/ROBO1/LYN/ITGA2/F3/SERPINE1/CCR2/CD74/TGFB1/PLA2G7/RAC2/SLAMF8/S100A7/CCR1/C5AR1/PDGFRA/CCR7/SMOC2/CCL4/CXCR4/CCL2/PDGFRB/SEMA4D/MET/USP14/VEGFC/KDR/S1PR1/MYCBP2/TPBG/CALR/CCR6/ANO6/ADGRA2/C3AR1/RIPOR2/CSF1R/GPR183/FGFR1/GPSM3/CCL19/SEMA4B/NRP1/JAM3/MIF/CXCL14/IL23A/IL6/CREB3/AIF1/FPR2/SEMA3G/NCKAP1L/NOD2/SEMA4A/PLXNA3/SWAP70/IL16/CMKLR1/ARTN/ZSWIM8/MDK/CCL7/SEMA3F/ANGPT2
GOBP_MULTI_MULTICELLULAR_ORGANISM_PROCESS
GOBP_MULTI_MULTICELLULAR_ORGANISM_PROCESS
GOBP_MULTI_MULTICELLULAR_ORGANISM_PROCESS 196 0.441351006 1.563225714 0.003773585 0.044959494 0.035581594 3116 tags=31%, list=18%, signal=25%
TIMP1/IGFBP5/GJA1/SPP1/IDO1/IL1B/MMP7/MMP9/CD38/THBD/IGFBP7/MMP2/PNOC/SERPINE2/VMP1/ACSL4/ITGA2/STS/STC1/ITGA5/UCP2/AGO2/TGFBR2/PPAT/ARHGDIB/PTAFR/RGS2/COL16A1/IGFBP2/CALR/SEMG1/PAM/AKR1B1/CYP27B1/APOL2/UMPS/MAGED2/PTGFR/LIF/HAVCR2/ITGB4/CAD/WNT4/RAMP2/POLR1B/KPNA6/TRIM28/TGFB3/ETS1/ANGPT2/AKT1/ATP7A/ADCY7/SLC2A1/STAT5B/EMP2/ITGA3/SYDE1/NR2F2/TEAD3
GOBP_BIOLOGICAL_PROCESS_INVOLVED_IN_INTERACTION_WITH_HOST
GOBP_BIOLOGICAL_PROCESS_INVOLVED_IN_INTERACTION_WITH_HOST
GOBP_BIOLOGICAL_PROCESS_INVOLVED_IN_INTERACTION_WITH_HOST 205 0.424909817 1.514020569 0.003778338 0.044959494 0.035581594 2822 tags=29%, list=17%, signal=25%
CD55/CXCL8/CLDN1/IFITM2/SERPINB9/CAV1/SERPINB3/IFITM3/HYAL1/TRIM22/ITGA2/CD74/IFITM1/LGALS1/ITGA5/FCN1/CXCR4/CD86/CAV2/TRIM27/NPC1/ICAM1/KPNA2/HYAL2/MET/CD81/FCN3/P4HB/TRIM15/MRC1/KRT6A/CDK1/LY6E/SELPLG/PABPN1/NRP1/TYMS/CR2/ITGAV/PML/CASP8/CTSL/AXL/TNFRSF4/KPNB1/CCR5/CLEC5A/CD4/KPNA6/TRIM28/SLC1A5/NECTIN4/TYRO3/THOC1/SLC52A2/FUCA2/PVR/TRIM5/TRIM21/EFNB2
GOBP_OSTEOBLAST_DIFFERENTIATION GOBP_OSTEOBLAST_DIFFERENTIATION
GOBP_OSTEOBLAST_DIFFERENTIATION 199 0.448566556 1.590222007 0.003778338 0.044959494 0.035581594 2179 tags=28%, list=13%, signal=24%
CTHRC1/IGFBP5/TNC/GREM1/SPP1/COL1A1/FERMT2/COL6A1/CCN1/IFITM1/TPM4/VCAN/CBFB/TCIRG1/RUNX2/TWIST1/DDX21/SHH/CEBPB/LOX/SEMA4D/CREB3L1/RASSF2/SNAI2/DDR2/VEGFC/FHL2/IARS1/LEF1/LTF/FASN/WWTR1/IL6ST/FFAR4/PSMC2/SND1/TWSG1/HDAC7/ACVR1/G

TPBP4/SYNCRIP/IL6/SFRP1/JAG1/MRC2/TMEM119/SMAD5/CLEC5A/WNT4/TMEM64/RIOX1/FIGNL1/ALPL/SFRP2/RRBP1

GOBP_NEGATIVE_REGULATION_OF_WNT_SIGNALING_PATHWAY GOBP_NEGATIVE_REGULATION_OF_WNT_SIGNALING_PATHWAY GOBP_NEGATIVE_REGULATION_OF_WNT_SIGNALING_PATHWAY 200 0.438935175 1.557725357 0.003783102 0.044959494 0.035581594 4077 tags=40%, list=24%, signal=30%

CTHRC1/GREM1/WNT5A/APCDD1/CAV1/DKK3/GRB10/PSME4/PSMB9/MAD2L2/SHH/LATS2/DACT1/RNF213/IGFBP6/SNAI2/DAB2/PSMD14/IGFBP2/WWTR1/BICC1/PSMD12/PSMC2/DKK1/MCC/PSMB2/PSMA3/PSMD1/IGFBP4/HIC1/PSMA5/STK3/TMEM131L/SFRP1/PSMD6/PSMB5/PSMC4/FZD4/JADE1/TMEM64/SFRP2/PSMB1/PSMA1/FERMT1/MDK/PSME3/CYLD/RUVBL2/PSMA7/PSMB8/PSMC5/TLE1/DVL1/C12orf43/APOE/PSMB10/PSMB6/PSMA4/CHD8/EMD/STK4/ALPK2/PSMD13/PSME1/PRICKLE1/PSMC1/DVL3/PSMB4/SOX9/PSMB3/LZTS2/PSMD2/PSMC6/PSMD7/GSC/PSMA2/ANKRD6/PSMD4/GLI1

GOBP_NEGATIVE_REGULATION_OF_DEFENSE_RESPONSE GOBP_NEGATIVE_REGULATION_OF_DEFENSE_RESPONSE GOBP_NEGATIVE_REGULATION_OF_DEFENSE_RESPONSE 189 0.440987502 1.557942055 0.003797468 0.044959494 0.035581594 3688 tags=36%, list=22%, signal=28%

MMP12/SPINK5/SERPINB9/TNFAIP6/ALOX5/ADA/SERPING1/HTRA1/CDH5/IFI16/SLAMF8/PTPRC/SEC14L1/SOCS3/SERPINB4/FCGR2B/CALCRL/FOXF1/NLRC5/SAMHD1/MICB/SERPINF1/LYAR/SIRPA/CST7/PARP14/TEK/A2M/WFDC1/FFAR4/METTL3/DUSP10/TNFRSF1B/PBK/RORA/CD200/PTGIS/LILRB1/FPR2/HAVCR2/NOD2/SYT11/PSMA1/NMI/GPX1/MDK/ADAR/SIGLEC10/TYRO3/ARRB2/TRIM21/APOE/IRAK3/GSTP1/UFD1/OTULIN/SELENOS/TRAFD1/IL2RA/CX3CL1/CR1/MAPKBP1/TARBP2/LDLR/DHX58/ILRUN/PSMB4/HLA-E

GOBP_POSITIVE_REGULATION_OF_ERK1_AND_ERK2_CASCADE GOBP_POSITIVE_REGULATION_OF_ERK1_AND_ERK2_CASCADE GOBP_POSITIVE_REGULATION_OF_ERK1_AND_ERK2_CASCADE 185 0.431468666 1.52084244 0.003816794 0.044959494 0.035581594 2549 tags=28%, list=15%, signal=24%

CHI3L1/CCL11/PLA2G2A/CCL18/FERMT2/CD44/CD74/TGFB1/PTPRC/CCL20/F2R/S100A7/RIPK2/CCR1/RAMP3/NQO2/C5AR1/PDGFRA/CCR7/CCL4/GNAI2/ICAM1/CCL2/PDGFRB/CCL24/KDR/CCL22/TEK/TPBG/FFAR4/CSF1R/GPR183/CCL19/NRP1/AKAP12/MIF/CFLAR/DENND2B/MAP2K1/GPNMB/FPR2/HAVCR2/TNFSF11/NOD2/PDGFC/SERPINF2/NPNT/PRXL2C/CD4/RASGRP1/CCL7/FBXW7

GOBP_SISTER_CHROMATID_SEGREGATION GOBP_SISTER_CHROMATID_SEGREGATION GOBP_SISTER_CHROMATID_SEGREGATION 187 0.430917026 1.518839481 0.003816794 0.044959494 0.035581594 3908 tags=40%, list=23%, signal=31%

FEN1/TRIP13/CCNB1/MAD2L2/TUBG1/BUB1/CDC20/PRC1/NCAPG/NDC80/NEK6/RRS1/CDCA5/TACC3/PTTG1/KIF14/CDC6/MIS12/KNSTRN/TENT4A/NUP62/CENPE/AURKB/BUB1B/NUSAP1/PLK1/UBE2C/RAN/SMC4/DLGAP5/KIF23/CDC27/ZWINT/NUDC/KPNB1/KIF2C/PSRC1/HECW2/CDCA8/KIF4A/CDC26/PINX1/RAD51C/SPAG5/NCAPH/NCAPG2/CDT1/FBXW7/SPDL1/KLHL22/EML3/KNTC1/ANAPC7/FBXO5/SGO2/RACGAP1/SFPQ/SMC2/TUBG2/VPS4A/NUF2/DSCC1/KIF18A/REC8/NAA10/KIF18B/ZNF207/DDX11/CENPF/BUB3/TTK/CHFR/TOP2A/MAD2L1/DYNC1LI1

GOBP_POSITIVE_REGULATION_OF_PEPTIDYL_TYROSINE_PHOSPHORYLATION

GOBP_POSITIVE_REGULATION_OF_PEPTIDYL_TYROSINE_PHOSPHORYLATION

GOBP_POSITIVE_REGULATION_OF_PEPTIDYL_TYROSINE_PHOSPHORYLATION 182 0.43331109 1.52570899 0.003826531 0.044959494 0.035581594 2677 tags=29%, list=16%, signal=25% GREM1/PECAM1/AGT/LYN/LRP8/CD44/HCLS1/CD74/TGFB1/PTPRC/RIPK2/SOCS3/ITGA5/CLEC7A/ENPP2/PARP9/KIT/FYN/JAK2/ICAM1/SEMA4D/CD81/CD40/PARP14/GPRC5B/STAT3/BANK1/IL6ST/CSF1R/AFAP1L2/NRP1/PRNP/MIF/ACVR1/LIF/IL23A/IL6/TSLP/ARHGEF2/FGF7/IFNG/NOD2/ACE/PTPN1/CD4/HAX1/IL24/SYK/NCAPG2/FBXW7/BMP6/TNFRSF18/IL18

GOBP_REGULATION_OF_CELL_JUNCTION_ASSEMBLY

GOBP_REGULATION_OF_CELL_JUNCTION_ASSEMBLY

GOBP_REGULATION_OF_CELL_JUNCTION_ASSEMBLY 182 0.438175957 1.542838418 0.003826531 0.044959494 0.035581594 2193 tags=23%, list=13%, signal=20% GREM1/GJA1/IL1B/AGT/WNT5A/CLDN1/CAV1/FERMT2/CBFB/ADGRL2/DLC1/THY1/MAP4K4/TLR2/SRPX2/SEMA4D/SNAI2/KDR/COL16A1/TEK/TPBG/LIMCH1/FLRT3/ITGB1BP1/NRP1/GPC6/MACF1/PRKCH/CORO1C/DUSP22/VCL/SETD5/RCC2/SFRP1/AGRN/APOD/PHLDB2/EIF4G1/WNT4/PTPRS/SEMA4A

GOBP_NEGATIVE_REGULATION_OF_CANONICAL_WNT_SIGNALING_PATHWAY

GOBP_NEGATIVE_REGULATION_OF_CANONICAL_WNT_SIGNALING_PATHWAY

GOBP_NEGATIVE_REGULATION_OF_CANONICAL_WNT_SIGNALING_PATHWAY 170 0.445983942 1.554139783 0.00388601 0.045551668 0.036050249 4077 tags=42%, list=24%, signal=32% CTHRC1/GREM1/WNT5A/CAV1/DKK3/PSME4/PSMB9/MAD2L2/SHH/LATS2/DACT1/IGFBP6/SNAI2/DAB2/PSMD14/IGFBP2/WWTR1/BICC1/PSMD12/PSMC2/DKK1/MCC/PSMB2/PSMA3/PSMD1/IGFBP4/PSMA5/STK3/TMEM131L/SFRP1/PSMD6/PSMB5/PSMC4/FZD4/JADE1/TMEM64/SFRP2/PSMB1/PSMA1/FERMT1/MDK/PSME3/CYLD/RUVBL2/PSMA7/PSMB8/PSMC5/TLE1/DVL1/APOE/PSMB10/PSMB6/PSMA4/CHD8/EMD/STK4/PSMD13/PSME1/PRICKLE1/PSMC1/DVL3/PSMB4/SOX9/PSMB3/LZTS2/PSMD2/PSMC6/PSMD7/PSMA2/ANKRD6/PSMD4/GLI1

GOBP_HEMATOPOIETIC_PROGENITOR_CELL_DIFFERENTIATION

GOBP_HEMATOPOIETIC_PROGENITOR_CELL_DIFFERENTIATION

GOBP_HEMATOPOIETIC_PROGENITOR_CELL_DIFFERENTIATION 154 0.45299162 1.557211765 0.00397878 0.046530393 0.036824825 5163 tags=51%, list=30%, signal=36%

TGFB1/INHBA/MEOX1/PTPRC/CBFB/PSME4/PSMB9/KIT/PDGFRA/PLEK/SHH/LMO2/HYAL2/KDR/PSMD14/PSMD12/CSF1R/PSMC2/PSMB2/PSMA3/METTL3/PSMD1/TCF3/JAM3/FST/PSMA5/SFRP1/PSMD6/PSMB5/PSMC4/BATF/PUS7/PSMB1/PSMA1/ACE/FSTL3/ADAR/PSME3/PSMA7/PSMB8/PSMC5/ZBTB24/PSMB10/PSMB6/PSMA4/SPI1/LDB1/PSMD13/PSME1/XRCC5/PSMC1/PSMB4/PSMB3/PSMD2/TOP2A/PSMC6/PSMD7/DHX36/PSMA2/PSMD4/HOXB4/GATA2/PSMD9/TNFRSF13B/EIF2AK2/PSMB7/PYGO1/PRKDC/AP3B1/PDCD2/SRF/ACP6/PRRC2C/EXT1/FLT3/PSMC3/SETD1A/PSMD8/ITCH

GOBP_SEQUESTERING_OF_CALCIUM_ION GOBP_SEQUESTERING_OF_CALCIUM_ION

GOBP_SEQUESTERING_OF_CALCIUM_ION 112 0.509660152 1.687820776 0.004103967 0.047330189 0.037457795 1558 tags=25%, list=9%, signal=23% CEMIP/CXCL9/CXCL11/CXCL10/LYN/TRPA1/JPH1/APLNR/PTPRC/F2R/PLN/CCR7/CD19/ANXA6/THY1/PLCG2/DIAPH1/CORO1A/CALR/CLIC2/ERO1A/SELENON/LCK/CCL19/GSTO1/PKD2/HSP90B

1/IBTK
GOBP_INTERFERON_GAMMA_PRODUCTION GOBP_INTERFERON_GAMMA_PRODUCTION GOBP_INTERFERON_GAMMA_PRODUCTION 102 0.541414076 1.768162697 0.004137931 0.047330189 0.037457795 2123 tags=33%, list=13%, signal=29% CD274/IL1B/WNT5A/SLC7A5/IL33/CCR2/PDE4B/INHBA/IL27RA/RIPK2/CLEC7A/IL1R1/TLR8/CCR7/TRIM27/SASH3/HLA-DPA1/SIRPA/LAPTM5/CD276/IL12RB1/IL18R1/EBI3/PRNP/CD2/BTN3A1/CD47/IL23A/LILRB4/AXL/LILRB1/HAVCR2/SLAMF6/NOD2
GOBP_MUCOPOLYSACCHARIDE_METABOLIC_PROCESS GOBP_MUCOPOLYSACCHARIDE_METABOLIC_PROCESS GOBP_MUCOPOLYSACCHARIDE_METABOLIC_PROCESS 113 0.50129355 1.65851416 0.004137931 0.047330189 0.037457795 2602 tags=28%, list=15%, signal=24% CEMIP/IL1B/CSGALNACT1/CHST15/HYAL1/CD44/TGFB1/BGN/CHST2/CHST11/CHSY1/LUM/VCAN/ST3GAL4/DSE/DCN/HYAL2/CHPF/B3GALT6/CSGALNACT2/SPOCK2/FMOD/EXT2/CHST12/ST3GAL2/B3GNT7/LYVE1/B4GALT2/B4GALT6/CHPF2/CHST3/AKT1
GOBP_POSITIVE_REGULATION_OF_CALCIUM_ION_TRANSPORT GOBP_POSITIVE_REGULATION_OF_CALCIUM_ION_TRANSPORT GOBP_POSITIVE_REGULATION_OF_CALCIUM_ION_TRANSPORT 114 0.50804277 1.681624431 0.004137931 0.047330189 0.037457795 1531 tags=21%, list=9%, signal=19% CEMIP/CXCL9/CXCL11/CXCL10/CAV1/KCNE3/STC1/APLNR/F2R/CCR1/RAMP3/HOMER1/CD19/CCL4/THY1/P2RX5/CCL2/PDGFRB/PLCG2/GSTO1/PKD2/TRPV2/WFS1/ORAI1
GOBP_CELL_SUBSTRATE_JUNCTION_ORGANIZATION GOBP_CELL_SUBSTRATE_JUNCTION_ORGANIZATION GOBP_CELL_SUBSTRATE_JUNCTION_ORGANIZATION 104 0.51201099 1.674088619 0.004143646 0.047330189 0.037457795 2081 tags=33%, list=12%, signal=29% GREM1/FERMT2/LAMC1/ITGA2/LAMC2/LAMA3/FN1/ITGA5/DLC1/THY1/MAP4K4/RAB8B/ACTN1/BCR/TRIP6/KDR/COL16A1/TEK/LIMCH1/ITGB1BP1/NRP1/ITGA6/MACF1/CORO1C/DUSP22/VCL/AJUBA/RCC2/SFRP1/TLN1/APOD/PHLDB2/ITGB4/WNT4
GOBP_GLIAL_CELL_DEVELOPMENT GOBP_GLIAL_CELL_DEVELOPMENT GOBP_GLIAL_CELL_DEVELOPMENT 104 0.489399875 1.600158547 0.004143646 0.047330189 0.037457795 3071 tags=31%, list=18%, signal=25% S100A8/S100A9/ADGRG6/BACE2/LYN/CLU/C5AR1/VIM/SHH/TLR2/C1QA/TSPAN2/LAMB2/NRROS/MXRA8/PHGDH/IL6/EIF2B3/IFNG/FPR2/DICER1/ITGB4/EIF2B2/B4GALT6/MDK/AKT1/DLL1/EIF2B5/ARHGEF10/EIF2B4/FA2H/GSTP1
GOBP_INTEGRIN_MEDIATED_SIGNALING_PATHWAY GOBP_INTEGRIN_MEDIATED_SIGNALING_PATHWAY GOBP_INTEGRIN_MEDIATED_SIGNALING_PATHWAY 104 0.518886737 1.696569798 0.004143646 0.047330189 0.037457795 3311 tags=44%, list=20%, signal=35% TIMP1/ADAMTS1/FERMT2/COL3A1/ITGA2/LAMA3/FUT8/ZYX/FN1/ITGA5/MADCAM1/ITGB2/PLEK/THY1/FGR/HCK/FLNA/ITGAM/COL16A1/ITGA8/FYB1/VAV1/ADAM9/ITGB1BP1/ITGA9/NRP1/ITGA6/CD47/ITGAV/ADAM15/RCC2/ITGAX/LAMA5/TLN1/ITGB4/FERMT1/SYK/ITGA4/ITGA7/PTPN11/EMP2/ITGA3/CCM2/LAT/PRKD1/FERMT3
GOBP_POSITIVE_REGULATION_OF_CHEMOTAXIS GOBP_POSITIVE_REGULATION_OF_CHEMOTAXIS

GOBP_POSITIVE_REGULATION_OF_CHEMOTAXIS 135 0.470584081 1.593904109 0.004149378 0.047330189 0.037457795 2508 tags=36%, list=15%, signal=31% CXCL13/CXCL8/CXCL10/WNT5A/ITGA2/F3/SERPINE1/CCR2/CD74/TGFB1/PLA2G7/RAC2/S100A7/CCR1/C5AR1/CCR7/SMOC2/CCL4/CXCR4/PDGFRB/MET/VEGFC/KDR/S1PR1/TPBG/CALR/CCR6/ANO6/C3AR1/RIPOR2/CSF1R/FGFR1/GPSM3/CCL19/NRP1/CXCL14/IL23A/IL6/CREB3/AIF1/FPR2/NCKAP1L/SWAP70/IL16/CMKLR1/ARTN/MDK/CCL7

GOBP_NEGATIVE_REGULATION_OF_CELL_CYCLE_G2_M_PHASE_TRANSITION GOBP_NEGATIVE_REGULATION_OF_CELL_CYCLE_G2_M_PHASE_TRANSITION GOBP_NEGATIVE_REGULATION_OF_CELL_CYCLE_G2_M_PHASE_TRANSITION 103 0.502229931 1.640261252 0.004166667 0.047330189 0.037457795 4036 tags=57%, list=24%, signal=44% PSME4/PSMB9/NABP1/DONSON/BLM/PSMD14/CDC6/PSMD12/CDK1/PSMC2/PSMB2/AURKB/PSMA3/AURKA/PSMD1/PLK1/PSMA5/AVEN/FHL1/PSMD6/PSMB5/PSMC4/NBN/ATF5/PSMB1/PSMA1/PINX1/CHEK1/UIMC1/PSME3/CLSPN/CDK5RAP3/NABP2/CUL1/PSMA7/PSMB8/PSMC5/PSMB10/PSMB6/PSMA4/INTS3/PSMD13/PSME1/VPS4A/BRCA1/USP47/RINT1/PSMC1/DTL/ZNF830/FBXL7/PSMB4/PSMB3/PSMD2/PSMC6/PSMD7/PSMA2/MRE11/PSMD4

GOBP_MRNA_EXPORT_FROM_NUCLEUS GOBP_MRNA_EXPORT_FROM_NUCLEUS GOBP_MRNA_EXPORT_FROM_NUCLEUS 100 0.514783119 1.672439697 0.004201681 0.047330189 0.037457795 4714 tags=59%, list=28%, signal=43% HHEX/SEC13/MAGOHB/DDX39A/PABPN1/NUP62/DDX19A/NUP98/NUP85/NXT1/POLR2D/SRSF7/MCM3AP/NUP93/NUP210/RAE1/NUP37/SMG7/NSUN2/THOC1/NXF3/NUP58/CPSF3/ALYREF/NUP205/UPF1/SLBP/EIF4A3/ALKBH5/UPF2/AAAS/IWS1/NUP50/RBM8A/FYTTD1/SRSF1/CHTOP/RBM15B/NUP35/NUP155/NUP107/SUPT6H/SRSF3/AGFG1/RANBP2/SRSF4/SRSF6/NDC1/NUP188/NUP88/THOC7/THOC5/NUP54/U2AF1/NXF1/ENY2/SRRM1/NUP214/SYMPK

GOBP_INTERLEUKIN_1_MEDIATED_SIGNALING_PATHWAY GOBP_INTERLEUKIN_1_MEDIATED_SIGNALING_PATHWAY GOBP_INTERLEUKIN_1_MEDIATED_SIGNALING_PATHWAY 96 0.537566273 1.733511349 0.004213483 0.047330189 0.037457795 4036 tags=51%, list=24%, signal=39% IL1RN/IL1B/RIPK2/PSME4/IL1R1/PSMB9/IRAK1/IL1A/PSMD14/PSMD12/PSMC2/PLCB1/PSMB2/PSMA3/PSMD1/IRAK2/PSMA5/IL6/PSMD6/PSMB5/PSMC4/ZBP1/VRK2/RELA/NOD2/PSMB1/PSMA1/PSME3/CUL1/PSMA7/PSMB8/PSMC5/UBE2N/IRAK3/PSMB10/PSMB6/PSMA4/PSMD13/PSME1/PSMC1/PSMB4/PSMB3/PSMD2/IKBKG/PSMC6/PSMD7/PSMA2/RPS6KA4/PSMD4

GOBP_POSITIVE_REGULATION_OF_PRODUCTION_OF_MOLECULAR_MEDIATOR_OF_IMMUNE_RESPONSE GOBP_POSITIVE_REGULATION_OF_PRODUCTION_OF_MOLECULAR_MEDIATOR_OF_IMMUNE_RESPONSE GOBP_POSITIVE_REGULATION_OF_PRODUCTION_OF_MOLECULAR_MEDIATOR_OF_IMMUNE_RESPONSE 95 0.538335489 1.734265757 0.004213483 0.047330189 0.037457795 2123 tags=31%, list=13%, signal=27% IL1B/MZB1/WNT5A/SLC7A5/IL33/CD74/TGFB1/PTPRC/XBP1/TNFSF13/CLEC7A/IL1R1/MAD2L2/DDX21/SASH3/HK1/FFAR2/CD81/CD40/LAPTM5/GPRC5B/IL18R1/EXOSC3/IL6/TNFRSF4/LILRB1/MLH1/RBP4/NOD2

GOBP_LEUKOCYTE_APOPTOTIC_PROCESS GOBP_LEUKOCYTE_APOPTOTIC_PROCESS GOBP_LEUKOCYTE_APOPTOTIC_PROCESS 98 0.54283815 1.755095398 0.004225352

0.047330189	0.037457795	2768	tags=37%,	list=16%,	signal=31%
ANXA1/CD274/IDO1/WNT5A/SLC7A11/IL7R/LYN/ADA/HCLS1/CD74/JAK3/HIF1A/CCR7/GIMAP8/CD27/BCL6/CCL19/AURKB/PIK3CD/PLEKHO2/BTK/ITPKB/IL6/ADAM8/CTSL/AXL/LILRB1/SLC39A10/NOD2/TSC22D3/IRF7/BLK/NOC2L/AKT1/DOCK8/IRF3
GOBP_CELLULAR_RESPIRATION	GOBP_CELLULAR_RESPIRATION
GOBP_CELLULAR_RESPIRATION	164	-0.41749033	-1.65888802	0.004237288
0.047330189	0.037457795	3654	tags=49%,	list=22%,	signal=39%
SLC25A13/IMMP2L/AFG1L/OGDH/NDUFA3/NDUFS1/OGDHL/SNCA/UQCR11/NOA1/NDUFS3/SIRT3/COX10/COX7B/IDH1/NDUFS4/NDUFA13/SDHA/UQCR10/MTFR1L/NDUFS7/PRELID1/CYC1/ATP5F1D/UQCRFS1/NDUFS2/COX8A/ETFRF1/COX6A1/PDHA1/IDH3B/COX4I1/UQCRC2/NDUFB10/NDUFB5/NDUFB9/IDH3A/MDH1/CYCS/UQCRC1/NDUFA1/GHITM/FAHD1/NDUFB1/COX5A/COX6C/GPD1/SLC25A12/DLAT/SLC25A25/BLOC1S1/ME2/IREB2/SUCLG1/DLD/NDUFV1/COQ9/SUCLA2/COX5B/SDHB/COX15/NDUFA10/PINK1/ACO2/ETFB/NDUFA2/NDUFB7/ETFA/NNT/CAT/SDHD/NDUFA5/SDHAF4/SLC25A23/SUCLG2/LYRM7/ME3/ETFDH/OPN3/PPARGC1A
GOBP_INORGANIC_ANION_TRANSPORT	GOBP_INORGANIC_ANION_TRANSPORT
GOBP_INORGANIC_ANION_TRANSPORT	166	-0.41572309	-1.656899323	0.004237288
0.047330189	0.037457795	1710	tags=17%,	list=10%,	signal=16%
PCYOX1/GLRA2/CA7/SLC39A14/ABCC6/ANO10/ANO5/CFTR/SLC20A1/ATP8B1/BEST4/FXYD3/CLDN4/ANKH/CLIC5/SLC37A2/SLC20A2/CLCN2/IP6K2/BEST2/PRKG2/SLC26A3/ENPP1/CLCA4/SLC4A4/GABRA2/SLC1A1/ABCB1/SLC26A2
GOBP_POSITIVE_REGULATION_OF_CELL_CYCLE_PHASE_TRANSITION
GOBP_POSITIVE_REGULATION_OF_CELL_CYCLE_PHASE_TRANSITION
GOBP_POSITIVE_REGULATION_OF_CELL_CYCLE_PHASE_TRANSITION	93	0.52415952
1.675187895	0.004267425	0.047330189	0.037457795	3810	tags=42%,	list=23%,	signal=33%
ANXA1/ADAMTS1/RGCC/CDC25B/HYAL1/FAM83D/CCNB1/CDC25A/CDCA5/CDC6/EZH2/CDK1/PLCB1/CCND1/UBE2C/LSM10/DLGAP5/CDC27/RCC2/CDC7/AIF1/RDX/EIF4G1/CDK4/PAF1/RAD51C/UBE2E2/CDT1/AKT1/NPM1/ANAPC7/FBXO5/KLHL18/DTL/CCND3/DDRGK1/ADAM17/CCND2/PAGR1
GOBP_REGULATION_OF_CALCIUM_ION_TRANSPORT_INTO_CYTOSOL
GOBP_REGULATION_OF_CALCIUM_ION_TRANSPORT_INTO_CYTOSOL
GOBP_REGULATION_OF_CALCIUM_ION_TRANSPORT_INTO_CYTOSOL	90	0.533642976
1.696807739	0.004267425	0.047330189	0.037457795	1441	tags=24%,	list=9%,	signal=22%
CEMIP/CXCL9/CXCL11/CXCL10/CAV1/LYN/JPH1/APLNR/F2R/RAMP3/PLN/CD19/FYN/THY1/P2RX5/DIAPH1/CORO1A/CLIC2/SELENON/GSTO1/PKD2/PRNP
GOBP_MACROPHAGE_ACTIVATION	GOBP_MACROPHAGE_ACTIVATION
GOBP_MACROPHAGE_ACTIVATION	89	0.532659907	1.693202538	0.004279601
0.047330189	0.037457795	2523	tags=42%,	list=15%,	signal=36%
CD93/WNT5A/IL33/CD74/PTPRC/CLU/SBNO2/TLR8/C5AR1/ITGB2/FCGR2B/GPR137B/JAK2/ADGRF5/TLR2/TYROBP/ITGAM/CST7/CRTC3/C1QA/CTSC/SPHK1/MIF/TLR1/RORA/CD200/IL6/IFNG/AIF1/FPR2/HAVCR2/JMJD6/SYT11/LRRK2/DYSF/NMI/SYK
GOBP_CELLULAR_RESPONSE_TO_UV	GOBP_CELLULAR_RESPONSE_TO_UV
GOBP_CELLULAR_RESPONSE_TO_UV	85	0.540663942	1.707631292	0.004291845

0.047330189 0.037457795 3450 tags=40%, list=20%, signal=32%
MMP1/MMP3/TIMP1/MMP9/MMP2/HYAL1/CARD16/POLD3/HYAL2/CDC25A/EIF2S1/MME/AURKB/METTL3/PBK/ATR/NEDD4/PARP1/MFAP4/NOC2L/AQP1/CHEK1/RUVBL2/ATF4/NPM1/DDB2/COPS9/TRIAP1/TMEM161A/HYAL3/DDB1/ACTR5/USP47/BAK1
GOBP_VASCULAR_ENDOTHELIAL_GROWTH_FACTOR_RECEPTOR_SIGNALING_PATHWAY
GOBP_VASCULAR_ENDOTHELIAL_GROWTH_FACTOR_RECEPTOR_SIGNALING_PATHWAY
GOBP_VASCULAR_ENDOTHELIAL_GROWTH_FACTOR_RECEPTOR_SIGNALING_PATHWAY 87
0.515268818 1.635773224 0.004291845 0.047330189 0.037457795 3632
tags=43%, list=21%, signal=34%
IL1B/NCF2/SULF1/GRB10/HIF1A/TMEM204/ITGA5/FYN/HHEX/MMRN2/VEGFC/KDR/EMILIN1/VAV1/SHB/PRKCB/CLEC14A/NRP1/ITGAV/ELMO1/AXL/NEDD4/NCF4/FZD4/NCKAP1L/SHC2/MAPKAPK2/PTPN1/CYBB/NIBAN2/ROCK1/HSP90AA1/WASF2/PRKD1/MAPKAPK3/PRKD2/CYBA
GOBP_NEGATIVE_REGULATION_OF_INTRINSIC_APOPTOTIC_SIGNALING_PATHWAY
GOBP_NEGATIVE_REGULATION_OF_INTRINSIC_APOPTOTIC_SIGNALING_PATHWAY
GOBP_NEGATIVE_REGULATION_OF_INTRINSIC_APOPTOTIC_SIGNALING_PATHWAY 88
0.529866595 1.682081621 0.004297994 0.047330189 0.037457795 3639
tags=47%, list=21%, signal=37%
VNN1/MMP9/CD44/HYOU1/CD74/CLU/HIF1A/XBP1/CREB3L1/SNAI2/DNAJA1/NME5/ENO1/MUC1/DDIAS/BID/MIF/GRINA/CREB3/ARHGEF2/BCL2L12/FIGNL1/SFRP2/LRRK2/PTPN1/NOC2L/SOD2/GPX1/AKT1/PARK7/TRIAP1/PLAUR/CCAR2/YBX3/TMEM161A/SELENOS/HERPUD1/USP47/PPIF/MDM2/TRAP1
GOBP_INTERLEUKIN_8_PRODUCTION GOBP_INTERLEUKIN_8_PRODUCTION
GOBP_INTERLEUKIN_8_PRODUCTION 79 0.540545921 1.692184597 0.00433526
0.047330189 0.037457795 1479 tags=25%, list=9%, signal=23%
CHI3L1/ANXA1/IL1B/WNT5A/NOS2/F3/SERPINE1/CD74/PTPRC/F2R/CLEC7A/TLR8/FCN1/TLR2/HYAL2/FFAR2/STAT3/AFAP1L2/CD2/TLR1
GOBP_REGULATION_OF_PHAGOCYTOSIS GOBP_REGULATION_OF_PHAGOCYTOSIS
GOBP_REGULATION_OF_PHAGOCYTOSIS 86 0.528076523 1.668258077 0.00433526
0.047330189 0.037457795 2523 tags=38%, list=15%, signal=33%
IL1B/RAB31/C3/ITGA2/C2/PTPRC/FCER1G/CLEC7A/FCGR2B/FGR/TLR2/HCK/CCL2/CD300A/LYAR/SIRPA/CALR/ANO6/DOCK2/SPHK1/IL15RA/MFGE8/CD47/ITGAV/CD300LF/IFNG/FPR2/NCKAP1L/NOD2/SYT11/DYSF/SIRPG/SYK
GOBP_POSITIVE_REGULATION_OF_TUMOR_NECROSIS_FACTOR_SUPERFAMILY_CYTOKINE_PRODUCTION
GOBP_POSITIVE_REGULATION_OF_TUMOR_NECROSIS_FACTOR_SUPERFAMILY_CYTOKINE_PRODUCTION
GOBP_POSITIVE_REGULATION_OF_TUMOR_NECROSIS_FACTOR_SUPERFAMILY_CYTOKINE_PRODUCTION 82 0.540911941 1.695574267 0.004347826 0.047330189 0.037457795
2523 tags=41%, list=15%, signal=35%
WNT5A/IL33/CCR2/PF4/LY96/LPL/PTPRC/CLU/RIPK2/CLEC7A/TWIST1/JAK2/CD86/SASH3/TLR2/PTAFR/TYROBP/IL1A/STAT3/CCL19/CD2/MIF/TLR1/IL23A/IL6/ADAM8/ARHGEF2/IFNG/HAVCR2/SELENOK/NOD2/MAPKAPK2/RASGRP1/SYK
GOBP_CD4_POSITIVE_ALPHA_BETA_T_CELL_DIFFERENTIATION
GOBP_CD4_POSITIVE_ALPHA_BETA_T_CELL_DIFFERENTIATION

GOBP_CD4_POSITIVE_ALPHA_BETA_T_CELL_DIFFERENTIATION 76 0.53998566 1.67194304 0.004354136 0.047330189 0.037457795 2728 tags=43%, list=16%, signal=37%
ANXA1/JAK3/IRF4/HLA-DRA/NFKBIZ/ENTPD7/CBFB/RIPK2/CD86/SASH3/BCL6/LEF1/STAT3/IL12RB1/GPR183/CCL19/IL18R1/RUNX3/RORA/NKX2-3/IL23A/IL6/CTSL/IFNG/BATF/SLAMF6/NCKAP1L/HLX/SEMA4A/MALT1/LY9/IL18/ATP7A
GOBP_POSITIVE_REGULATION_OF_INTERLEUKIN_6_PRODUCTION GOBP_POSITIVE_REGULATION_OF_INTERLEUKIN_6_PRODUCTION GOBP_POSITIVE_REGULATION_OF_INTERLEUKIN_6_PRODUCTION 81 0.534916775 1.67402716 0.004366812 0.047330189 0.037457795 2123 tags=35%, list=13%, signal=30%
POU2AF1/IL1B/WNT5A/IL33/CD74/LPL/F2R/RIPK2/XBP1/CLEC7A/TLR8/TWIST1/LILRB2/TLR2/PTAFR/HYAL2/TYROBP/IL1A/AKIRIN2/STAT3/TLR1/IL6/TSLP/ARHGEF2/IFNG/AIF1/SELENOK/NOD2
GOBP_ENDOTHELIAL_CELL_DEVELOPMENT GOBP_ENDOTHELIAL_CELL_DEVELOPMENT GOBP_ENDOTHELIAL_CELL_DEVELOPMENT 66 0.548396735 1.672600481 0.004373178 0.047330189 0.037457795 2098 tags=32%, list=12%, signal=28%
COL15A1/IL1B/PECAM1/CLDN1/MSN/ENG/HEG1/CDH5/COL18A1/STC1/S1PR3/ICAM1/MET/FASN/ROBO4/PLCB1/PPP1R16B/VCL/PLOD3/RDX/PTPRS
GOBP_CELL_CYCLE_DNA_REPLICATION GOBP_CELL_CYCLE_DNA_REPLICATION GOBP_CELL_CYCLE_DNA_REPLICATION 63 0.570747084 1.728011706 0.004379562 0.047330189 0.037457795 3571 tags=43%, list=21%, signal=34%
FEN1/POLD3/DONSON/RTEL1/BCL6/MCM6/CDC45/TIPIN/DACH1/RFC3/GINS3/ZPR1/CDC7/POLE2/DBF4/MCM3/GINS1/MCM5/RFC4/CDT1/POLD2/MCM7/FBXO5/UPF1/POLA2/MCM2/ZNF830
GOBP_NEGATIVE_REGULATION_OF_LEUKOCYTE_PROLIFERATION GOBP_NEGATIVE_REGULATION_OF_LEUKOCYTE_PROLIFERATION GOBP_NEGATIVE_REGULATION_OF_LEUKOCYTE_PROLIFERATION 83 0.551083487 1.725903137 0.004379562 0.047330189 0.037457795 3388 tags=43%, list=20%, signal=35%
GREM1/CD274/IDO1/MNDA/IL33/LYN/GAL/FCGR2B/SHH/CEBPB/LILRB2/CD86/CD300A/TYROBP/LST1/TWSG1/PRNP/BTK/LILRB4/TMEM131L/LILRB1/GPNMB/HAVCR2/INPP5D/BLK/GLMN/LRRC32/PRKAR1A/DLG5/BTN2A2/CTLA4/GSTP1/IL2RA/PLA2G2D/PKN1/CR1
GOBP_POSITIVE_REGULATION_OF_B_CELL_ACTIVATION GOBP_POSITIVE_REGULATION_OF_B_CELL_ACTIVATION GOBP_POSITIVE_REGULATION_OF_B_CELL_ACTIVATION 75 0.533174067 1.648333167 0.004379562 0.047330189 0.037457795 2239 tags=36%, list=13%, signal=31%
CD38/ADA/CD74/TGFB1/PTPRC/XBP1/TNFSF13/TNFSF13B/MAD2L2/SASH3/CD27/CD81/CD40/BCL6/GPR183/FCRL3/EXOSC3/MIF/BTK/IL6/TNFRSF4/SLC39A10/MLH1/NCKAP1L/NOD2/INPP5D/CD320
GOBP_CELLULAR_EXTRAVASATION GOBP_CELLULAR_EXTRAVASATION GOBP_CELLULAR_EXTRAVASATION 69 0.578247215 1.775038374 0.004392387 0.047330189 0.037457795 1779 tags=39%, list=11%, signal=35%
PECAM1/SELL/PLVAP/SELP/CCR2/IL27RA/ST3GAL4/VCAM1/IL1R1/MADCAM1/ITGB2/THY1/S

ELE/ICAM1/PTAFR/CCL2/SIRPA/JAM2/PIK3CG/RIPOR2/PLCB1/SELPLG/PIK3CD/JAM3/CD47/ADAM8/FUT4
GOBP_REGULATION_OF_IMMUNOGLOBULIN_PRODUCTION
GOBP_REGULATION_OF_IMMUNOGLOBULIN_PRODUCTION
GOBP_REGULATION_OF_IMMUNOGLOBULIN_PRODUCTION 65 0.570339104
1.733899004 0.004392387 0.047330189 0.037457795 2089 tags=29%, list=12%, signal=26%
IL13RA2/MZB1/IL33/TGFB1/IL27RA/PTPRC/XBP1/TNFSF13/MAD2L2/FCGR2B/SASH3/CD40/BCL6/FCRL3/EXOSC3/IL6/TNFRSF4/MLH1/RBP4
GOBP_POSITIVE_REGULATION_OF_CALCIUM_ION_TRANSMEMBRANE_TRANSPORT
GOBP_POSITIVE_REGULATION_OF_CALCIUM_ION_TRANSMEMBRANE_TRANSPORT
GOBP_POSITIVE_REGULATION_OF_CALCIUM_ION_TRANSMEMBRANE_TRANSPORT 67
0.553241643 1.689303646 0.004398827 0.047330189 0.037457795 1411
tags=19%, list=8%, signal=18%
CEMIP/CXCL9/CXCL11/CXCL10/KCNE3/APLNR/F2R/RAMP3/CD19/THY1/PLCG2/GSTO1/PKD2
GOBP_RIBOSOMAL_LARGE_SUBUNIT_BIOGENESIS
GOBP_RIBOSOMAL_LARGE_SUBUNIT_BIOGENESIS
GOBP_RIBOSOMAL_LARGE_SUBUNIT_BIOGENESIS 64 0.564007558 1.712315937
0.004398827 0.047330189 0.037457795 3356 tags=44%, list=20%, signal=35%
NOP2/GTF3A/NIFK/RRS1/NOP16/MRTO4/MAK16/RPL7L1/GTPBP4/RRP15/FTSJ3/DDX18/MRPL20/SDAD1/RPL26L1/PAK1IP1/NOC2L/NHP2/NPM1/URB1/NOL9/WDR12/HEATR3/DHX30/RPF2/BRIX1/PES1/LAS1L
GOBP_POSITIVE_REGULATION_OF_MONONUCLEAR_CELL_MIGRATION
GOBP_POSITIVE_REGULATION_OF_MONONUCLEAR_CELL_MIGRATION
GOBP_POSITIVE_REGULATION_OF_MONONUCLEAR_CELL_MIGRATION 61 0.572705751
1.726325338 0.004411765 0.047330189 0.037457795 2039 tags=36%, list=12%, signal=32%
CXCL13/CXCL10/WNT5A/SERPINE1/CCR2/PLA2G7/CCL20/S100A7/CCR1/MADCAM1/CCR7/CCL4/JAM2/CALR/CCR6/ANO6/CXCL14/ADAM8/CREB3/AIF1/FPR2/SELENOK
GOBP_POSITIVE_CHEMOTAXIS GOBP_POSITIVE_CHEMOTAXIS
GOBP_POSITIVE_CHEMOTAXIS 56 0.549034076 1.641421415 0.004418262
0.047330189 0.037457795 3386 tags=39%, list=20%, signal=32%
CXCL8/CXCL10/WNT5A/ITGA2/F3/MET/CORO1A/VEGFC/KDR/S1PR1/NRP1/MIF/FGF7/GPNMB/FPR2/IL16/ARTN/ANGPT2/FGF2/CDH13/HMGB1/CX3CL1
GOBP_CELLULAR_RESPONSE_TO_VASCULAR_ENDOTHELIAL_GROWTH_FACTOR_STIMULUS
GOBP_CELLULAR_RESPONSE_TO_VASCULAR_ENDOTHELIAL_GROWTH_FACTOR_STIMULUS
GOBP_CELLULAR_RESPONSE_TO_VASCULAR_ENDOTHELIAL_GROWTH_FACTOR_STIMULUS
58 0.554627214 1.662895544 0.004424779 0.047330189 0.037457795 2686
tags=45%, list=16%, signal=38%
ANXA1/ROBO1/GAS1/VCAM1/PDGFRA/SMOC2/DCN/PDGFRB/VEGFC/KDR/XDH/ADGRA2/SPHK1/PIK3CD/ITGB1BP1/NRP1/EGR3/ATP2B4/ERN1/NUS1/RELA/MAPKAPK2/RAMP2/AKT1/DLL1/JCAD
GOBP_REGULATION_OF_ANTIGEN_RECEPTOR_MEDIATED_SIGNALING_PATHWAY
GOBP_REGULATION_OF_ANTIGEN_RECEPTOR_MEDIATED_SIGNALING_PATHWAY

GOBP_REGULATION_OF_ANTIGEN_RECEPTOR_MEDIATED_SIGNALING_PATHWAY 62 0.58214283 1.752825355 0.004431315 0.047330189 0.037457795 2366 tags=42%, list=14%, signal=36% GBP1/LYN/ADA/PTPRC/CBFB/KCNN4/CCR7/CD19/FCGR2B/THY1/CD300A/CD81/LAPTM5/DGKZ/RAB29/LCK/PRKCB/FCRL3/PRNP/PRKCH/LPXN/LILRB4/SLC39A10/RELA/MALT1/BLK

GOBP_T_CELL_MIGRATION GOBP_T_CELL_MIGRATION GOBP_T_CELL_MIGRATION 62 0.581386167 1.750547053 0.004431315 0.047330189 0.037457795 2198 tags=40%, list=13%, signal=35% CXCL11/CXCL13/CXCL10/WNT5A/MSN/CCR2/IL27RA/ECM1/CCL20/S100A7/ICAM1/CCL2/S1PR1/CCR6/PIK3CG/LRCH1/RIPOR2/GPR183/PIK3CD/CD200/ADAM8/AIF1/APOD/SELENOK/CXCL16

GOBP_PEPTIDYL_PROLINE_MODIFICATION GOBP_PEPTIDYL_PROLINE_MODIFICATION GOBP_PEPTIDYL_PROLINE_MODIFICATION 52 0.564618424 1.666303162 0.004457652 0.047330189 0.037457795 4113 tags=46%, list=24%, signal=35% FKBP11/P3H2/PRDX4/PPIL1/P3H1/FKBP5/FKBP7/P4HB/ERO1A/FKBP14/PPIB/CRTAP/OGFOD1/NTMT1/PPWD1/NKTR/FKBP2/FKBP8/PPIF/PPIH/PTPA/RANBP2/P4HA1/PPID

GOBP_NEGATIVE_REGULATION_OF_PEPTIDYL_TYROSINE_PHOSPHORYLATION GOBP_NEGATIVE_REGULATION_OF_PEPTIDYL_TYROSINE_PHOSPHORYLATION GOBP_NEGATIVE_REGULATION_OF_PEPTIDYL_TYROSINE_PHOSPHORYLATION 51 0.569199125 1.670851951 0.004504505 0.047330189 0.037457795 2177 tags=33%, list=13%, signal=29% CAV1/SH3BP5/PTPRC/SOCS3/SAMSN1/THY1/CD300A/HYAL2/SEMA4D/PARP14/SPINK1/GPRC5A/IBTK/DUSP22/LILRB4/SFRP1/SFRP2

GOBP_POSITIVE_REGULATION_OF_CALCIUM_ION_TRANSPORT_INTO_CYTOSOL GOBP_POSITIVE_REGULATION_OF_CALCIUM_ION_TRANSPORT_INTO_CYTOSOL GOBP_POSITIVE_REGULATION_OF_CALCIUM_ION_TRANSPORT_INTO_CYTOSOL 51 0.576556024 1.692447713 0.004504505 0.047330189 0.037457795 1411 tags=25%, list=8%, signal=23% CEMIP/CXCL9/CXCL11/CXCL10/CAV1/APLNR/F2R/RAMP3/CD19/THY1/P2RX5/GSTO1/PKD2

GOBP_STEROID_BIOSYNTHETIC_PROCESS GOBP_STEROID_BIOSYNTHETIC_PROCESS GOBP_STEROID_BIOSYNTHETIC_PROCESS 177 -0.358276639 -1.423348502 0.004504505 0.047330189 0.037457795 1911 tags=22%, list=11%, signal=20% SCARB1/OSBPL7/FDX1/SREBF2/SCP2/HINT2/PBX1/SCAP/PLPP6/PRKAG2/CYP1A1/SIRT1/PRKAA1/ERLIN2/ARV1/AKR1C3/HSD3B1/DGKQ/CFTR/SRD5A1/ABCG1/ACOT8/ACOX2/BMP2/HSD11B2/ACAA2/NR1H4/HSD17B11/CLCN2/LPCAT3/PRLR/CYP27A1/ABCB11/OSBPL1A/PPARGC1A/HSD3B2/HSD17B2/DHRS11/HMGCS2

GOBP_HORMONE_MEDIATED_SIGNALING_PATHWAY GOBP_HORMONE_MEDIATED_SIGNALING_PATHWAY GOBP_HORMONE_MEDIATED_SIGNALING_PATHWAY 179 -0.391003082 -1.550972024 0.00456621 0.047330189 0.037457795 2285 tags=22%, list=13%, signal=20% WBP2/UFL1/RNF6/TAF7/CALCOCO1/SCGB2A1/EP300/ESRRA/LGR5/NR1D2/NCOA4/SSTR2/LATS1/NR3C1/THRA/RXRA/ARNTL/YWHAH/PGRMC2/PAK1/SIRT1/RNF14/KDM3A/PPARA/ZMIZ1/PPARD/LGR4/APPL1/SST/PAQR8/NR3C2/PPARGC1B/NR5A2/SSTR1/PRLR/PLPP1/PPARG/PADI2/APPL2/THRB

GOBP_RESPONSE_TO_STARVATION GOBP_RESPONSE_TO_STARVATION
GOBP_RESPONSE_TO_STARVATION 182 -0.361895007 -1.438172009 0.004587156 0.047330189 0.037457795 2913 tags=31%, list=17%, signal=26% PPM1D/DDIT3/MAP1LC3A/BECN1/MTMR3/MAPK3/MAPK1/CDKN1A/HFE/RRAGC/ULK2/DSC2/SLC39A4/SIK2/GABARAPL2/DEPDC5/ZFYVE1/MYOD1/FAS/SREBF2/PMAIP1/FOXO3/SSTR2/GPT/LAMP2/LARS1/BMT2/PIK3C2B/PRKAG2/HMGCL/SIRT1/PRKAA1/SESN1/BCL2/KRT20/PPARA/IMPACT/AKR1C3/PCK2/MAX/SRD5A1/ZFP36/RNF152/MAP1LC3B/ATG14/AOC1/SESN3/SLC39A5/JUN/KLF10/SSTR1/PDK4/PPARG/ATF3/PPARGC1A/PCK1
GOBP_MATURATION_OF_SSU_RRNA_FROM_TRICISTRONIC_RRNA_TRANSCRIPT_SSU_RRNA_5_8S_RRNA_LSU_RRNA
GOBP_MATURATION_OF_SSU_RRNA_FROM_TRICISTRONIC_RRNA_TRANSCRIPT_SSU_RRNA_5_8S_RRNA_LSU_RRNA
GOBP_MATURATION_OF_SSU_RRNA_FROM_TRICISTRONIC_RRNA_TRANSCRIPT_SSU_RRNA_5_8S_RRNA_LSU_RRNA 36 0.613207695 1.6966453 0.004608295 0.047330189 0.037457795 3721 tags=47%, list=22%, signal=37% UTP4/RRP36/TSR1/RRS1/BYSL/UTP6/RPP40/NOP14/DCAF13/TBL3/HEATR1/BMS1/UTP3/NOL10/WDR46/UTP25/NOP9
GOBP_POSITIVE_REGULATION_OF_CD4_POSITIVE_ALPHA_BETA_T_CELL_ACTIVATION
GOBP_POSITIVE_REGULATION_OF_CD4_POSITIVE_ALPHA_BETA_T_CELL_ACTIVATION
GOBP_POSITIVE_REGULATION_OF_CD4_POSITIVE_ALPHA_BETA_T_CELL_ACTIVATION 36 0.607184953 1.679981359 0.004608295 0.047330189 0.037457795 2319 tags=44%, list=14%, signal=38% CD55/ANXA1/HLA-DRA/NFKBIZ/RIPK2/TGFBR2/CD86/SASH3/CD81/IL12RB1/CCL19/IL23A/IFNG/NCKAP1L/HLX/MALT1
GOBP_REGULATION_OF_EXTRACELLULAR_MATRIX_ORGANIZATION
GOBP_REGULATION_OF_EXTRACELLULAR_MATRIX_ORGANIZATION
GOBP_REGULATION_OF_EXTRACELLULAR_MATRIX_ORGANIZATION 43 0.585149269 1.675253373 0.004622496 0.047330189 0.037457795 2561 tags=42%, list=15%, signal=36%
AGT/RGCC/TGFB1/PDPN/AEBP1/ANTXR1/EFEMP2/TIE1/FAP/DDR2/EMILIN1/TNFRSF1B/FSCN1/CFLAR/IL6/PHLDB2/COLGALT1/ETS1
GOBP_TRABECULA_MORPHOGENESIS GOBP_TRABECULA_MORPHOGENESIS
GOBP_TRABECULA_MORPHOGENESIS 43 0.59953651 1.716443333 0.004622496 0.047330189 0.037457795 998 tags=28%, list=6%, signal=26% GREM1/ADGRG6/COL1A1/ADAMTS1/MMP2/ENG/HEG1/SBNO2/SEMA4D/FHL2/S1PR1/TEK
GOBP_PYRIDINE_CONTAINING_COMPOUND_METABOLIC_PROCESS
GOBP_PYRIDINE_CONTAINING_COMPOUND_METABOLIC_PROCESS
GOBP_PYRIDINE_CONTAINING_COMPOUND_METABOLIC_PROCESS 40 0.59093193 1.668029112 0.00462963 0.047330189 0.037457795 1805 tags=30%, list=11%, signal=27% NNMT/PSAT1/IDO1/KYNU/PNP/PTGS2/PARP9/KMO/IDH2/NAMPT/PDXK/PTGIS
GOBP_MICROGLIAL_CELL_ACTIVATION GOBP_MICROGLIAL_CELL_ACTIVATION
GOBP_MICROGLIAL_CELL_ACTIVATION 41 0.618282758 1.753350806 0.004636785 0.047330189 0.037457795 2342 tags=51%, list=14%, signal=44% IL33/PTPRC/CLU/TLR8/C5AR1/ITGB2/JAK2/TLR2/TYROBP/ITGAM/CST7/C1QA/CTSC/SPHK1/T

LR1/IL6/IFNG/AIF1/FPR2/SYT11/LRRK2
GOBP_NEGATIVE_REGULATION_OF_REPRODUCTIVE_PROCESS
GOBP_NEGATIVE_REGULATION_OF_REPRODUCTIVE_PROCESS
GOBP_NEGATIVE_REGULATION_OF_REPRODUCTIVE_PROCESS 49 0.56964555
1.657341834 0.004636785 0.047330189 0.037457795 1117 tags=27%, list=7%, signal=25%
TIMP1/GJA1/WNT5A/ADA/PRDX4/SULF1/TRIP13/ARHGDIB/SERPINF1/ZFPM2/CALR/SEMG1/WDR77
GOBP_ASTROCYTE_DEVELOPMENT GOBP_ASTROCYTE_DEVELOPMENT
GOBP_ASTROCYTE_DEVELOPMENT 37 0.605671573 1.682303844 0.004651163
0.047330189 0.037457795 1898 tags=30%, list=11%, signal=26%
S100A8/S100A9/BACE2/C5AR1/VIM/C1QA/TSPAN2/LAMB2/IL6/IFNG/FPR2
GOBP_GLYCOSYL_COMPOUND_BIOSYNTHETIC_PROCESS
GOBP_GLYCOSYL_COMPOUND_BIOSYNTHETIC_PROCESS
GOBP_GLYCOSYL_COMPOUND_BIOSYNTHETIC_PROCESS 38 0.605239441 1.688834364
0.004658385 0.047330189 0.037457795 2060 tags=39%, list=12%, signal=35%
ADA/NME1/ADA2/TYMP/NME5/UPP1/DTYMK/TK1/UMPS/NME4/LCMT2/IMPDH1/GMPS/NME7/CAD
GOBP_POSITIVE_REGULATION_OF_RELEASE_OF_SEQUESTERED_CALCIUM_ION_INTO_CYTOSOL
GOBP_POSITIVE_REGULATION_OF_RELEASE_OF_SEQUESTERED_CALCIUM_ION_INTO_CYTOSOL
GOBP_POSITIVE_REGULATION_OF_RELEASE_OF_SEQUESTERED_CALCIUM_ION_INTO_CYTOSOL 38 0.605422578 1.689345384 0.004658385 0.047330189 0.037457795 1411
tags=26%, list=8%, signal=24%
CEMIP/CXCL9/CXCL11/CXCL10/APLNR/F2R/CD19/THY1/GSTO1/PKD2
GOBP_AMINO_ACID_ACTIVATION GOBP_AMINO_ACID_ACTIVATION
GOBP_AMINO_ACID_ACTIVATION 45 0.58281231 1.67542006 0.004672897
0.047330189 0.037457795 3149 tags=38%, list=19%, signal=31%
WARS1/GARS1/YARS1/PPA1/IARS1/EEF1E1/AARS1/DARS1/TARS1/EPRS1/EARS2/CARS1/FARSA/LARS2/FARSB/RARS1/HARS1
GOBP_APOPTOTIC_CELL_CLEARANCE GOBP_APOPTOTIC_CELL_CLEARANCE
GOBP_APOPTOTIC_CELL_CLEARANCE 45 0.594482314 1.708968013 0.004672897
0.047330189 0.037457795 2024 tags=38%, list=12%, signal=33%
ANXA1/TGM2/C3/C2/RAC2/FCN1/CCL2/TYROBP/RHOH/FCN3/MFGE8/ITGAV/CD300LF/RHOG/AXL/PEAR1/JMJD6
GOBP_NEGATIVE_REGULATION_OF_LEUKOCYTE_APOPTOTIC_PROCESS
GOBP_NEGATIVE_REGULATION_OF_LEUKOCYTE_APOPTOTIC_PROCESS
GOBP_NEGATIVE_REGULATION_OF_LEUKOCYTE_APOPTOTIC_PROCESS 45 0.583322398
1.676886418 0.004672897 0.047330189 0.037457795 2408 tags=44%, list=14%, signal=38%
IDO1/IL7R/ADA/HCLS1/CD74/JAK3/HIF1A/CCR7/CD27/BCL6/CCL19/AURKB/ITPKB/AXL/LILRB1/SLC39A10/NOD2/TSC22D3/IRF7/NOC2L
GOBP_REGULATION_OF_ACUTE_INFLAMMATORY_RESPONSE
GOBP_REGULATION_OF_ACUTE_INFLAMMATORY_RESPONSE

GOBP_REGULATION_OF_ACUTE_INFLAMMATORY_RESPONSE 45 0.580661623 1.669237446 0.004672897 0.047330189 0.037457795 3348 tags=47%, list=20%, signal=38%
C2CD4A/IL1B/C3/PTGS2/CCR7/FCGR2B/FFAR2/IL6ST/PIK3CG/BTK/IL6/ADAM8/TNFSF11/DNASE1L3/F12/PARK7/EDNRB/GSTP1/SELENOS/OSMR/PLA2G2D
GOBP_CELLULAR_METABOLIC_COMPOUND_SALVAGE
GOBP_CELLULAR_METABOLIC_COMPOUND_SALVAGE
GOBP_CELLULAR_METABOLIC_COMPOUND_SALVAGE 33 0.608607785 1.664437209 0.004680187 0.047330189 0.037457795 2062 tags=33%, list=12%, signal=29%
APIP/ADA/PNP/TYMP/AMPD3/GMPR/UPP1/AMPD2/TK1/PDXK/ENOPH1
GOBP_EMBRYO_IMPLANTATION GOBP_EMBRYO_IMPLANTATION
GOBP_EMBRYO_IMPLANTATION 44 0.57621939 1.650311706 0.004680187 0.047330189 0.037457795 1073 tags=30%, list=6%, signal=28%
TIMP1/GJA1/SPP1/IL1B/MMP9/IGFBP7/MMP2/VMP1/STC1/AGO2/TGFBR2/ARHGDIB/CALR
GOBP_ENDOCRINE_HORMONE_SECRETION GOBP_ENDOCRINE_HORMONE_SECRETION
GOBP_ENDOCRINE_HORMONE_SECRETION 44 0.579095091 1.658547808 0.004680187 0.047330189 0.037457795 2994 tags=34%, list=18%, signal=28%
GJA1/IL1B/AGT/SELENOM/INHBA/GAL/RAB8B/FZD4/INHBB/TMF1/BMP6/C1QTNF1/CRY1/NIBAN2/PTPN11
GOBP_EXTRACELLULAR_MATRIX_ASSEMBLY GOBP_EXTRACELLULAR_MATRIX_ASSEMBLY
GOBP_EXTRACELLULAR_MATRIX_ASSEMBLY 44 0.577443851 1.653818604 0.004680187 0.047330189 0.037457795 2286 tags=41%, list=14%, signal=35%
AGT/COL1A2/RGCC/PXDN/TGFB1/ANTXR1/EFEMP2/TIE1/LOX/QSOX1/EMILIN1/FBLN5/LAMB1/LAMB2/PLOD3/PHLDB2/MFAP4/RAMP2
GOBP_POSITIVE_REGULATION_OF_G1_S_TRANSITION_OF_MITOTIC_CELL_CYCLE
GOBP_POSITIVE_REGULATION_OF_G1_S_TRANSITION_OF_MITOTIC_CELL_CYCLE
GOBP_POSITIVE_REGULATION_OF_G1_S_TRANSITION_OF_MITOTIC_CELL_CYCLE 33 0.609263214 1.666229693 0.004680187 0.047330189 0.037457795 2602 tags=39%, list=15%, signal=33%
ANXA1/ADAMTS1/RGCC/HYAL1/CDC6/PLCB1/CCND1/LSM10/AIF1/RDX/EIF4G1/UBE2E2/AKT1
GOBP_NEGATIVE_REGULATION_OF_CELL_MATRIX_ADHESION
GOBP_NEGATIVE_REGULATION_OF_CELL_MATRIX_ADHESION
GOBP_NEGATIVE_REGULATION_OF_CELL_MATRIX_ADHESION 35 0.618926521 1.702381066 0.004694836 0.047330189 0.037457795 2025 tags=40%, list=12%, signal=35%
MMP12/SERPINE1/DLC1/MAP4K4/POSTN/BCL6/ITGB1BP1/CORO1C/DUSP22/ADAM15/RCC2/JAG1/APOD/PHLDB2
GOBP_PYRIDINE_CONTAINING_COMPOUND_BIOSYNTHETIC_PROCESS
GOBP_PYRIDINE_CONTAINING_COMPOUND_BIOSYNTHETIC_PROCESS
GOBP_PYRIDINE_CONTAINING_COMPOUND_BIOSYNTHETIC_PROCESS 35 0.610725243 1.679823137 0.004694836 0.047330189 0.037457795 1805 tags=31%, list=11%, signal=28% NNMT/PSAT1/IDO1/KYNU/PTGS2/PARP9/KMO/IDH2/NAMPT/PDXK/PTGIS
GOBP_REGULATION_OF_CD4_POSITIVE_ALPHA_BETA_T_CELL_DIFFERENTIATION

GOBP_REGULATION_OF_CD4_POSITIVE_ALPHA_BETA_T_CELL_DIFFERENTIATION
GOBP_REGULATION_OF_CD4_POSITIVE_ALPHA_BETA_T_CELL_DIFFERENTIATION 47
0.581613247 1.67669618 0.004702194 0.047330189 0.037457795 2319
tags=38%, list=14%, signal=33%
ANXA1/JAK3/IRF4/HLA-DRA/NFKBIZ/CBFB/RIPK2/CD86/SASH3/BCL6/IL12RB1/CCL19/RUNX3/IL23A/IFNG/NCKAP1L/HLX/MALT1

GOBP_REGULATION_OF_CELL_ADHESION_MEDIATED_BY_INTEGRIN
GOBP_REGULATION_OF_CELL_ADHESION_MEDIATED_BY_INTEGRIN
GOBP_REGULATION_OF_CELL_ADHESION_MEDIATED_BY_INTEGRIN 47 0.575105479
1.657935347 0.004702194 0.047330189 0.037457795 2523 tags=45%, list=15%, signal=38%
CXCL13/PLAU/LYN/ADA/SERPINE1/PODXL/PIEZO1/SNAI2/PIK3CG/MUC1/ADAM9/TESC/ITGB1BP1/JAM3/LPXN/LIF/NCKAP1L/SFRP2/SWAP70/FERMT1/SYK

GOBP_CALCIUM_DEPENDENT_CELL_CELL_ADHESION_VIA_PLASMA_MEMBRANE_CELL_ADHESION_MOLECULES
GOBP_CALCIUM_DEPENDENT_CELL_CELL_ADHESION_VIA_PLASMA_MEMBRANE_CELL_ADHESION_MOLECULES
GOBP_CALCIUM_DEPENDENT_CELL_CELL_ADHESION_VIA_PLASMA_MEMBRANE_CELL_ADHESION_MOLECULES 30 0.612502995 1.644295338 0.004709576 0.047330189
0.037457795 2121 tags=27%, list=13%, signal=23%
SELL/CDH11/SELP/CDH5/FXYD5/DCHS1/AJUBA/CDH19

GOBP_POSITIVE_REGULATION_OF_PEPTIDYL_THREONINE_PHOSPHORYLATION
GOBP_POSITIVE_REGULATION_OF_PEPTIDYL_THREONINE_PHOSPHORYLATION
GOBP_POSITIVE_REGULATION_OF_PEPTIDYL_THREONINE_PHOSPHORYLATION 29
0.625932095 1.672723937 0.004716981 0.047330189 0.037457795 2142
tags=31%, list=13%, signal=27%
CHI3L1/CEMIP/WNT5A/TGFB1/RIPK2/SPHK1/PLK1/TNKS1BP1/TRPC6

GOBP_REGULATION_OF_DEFENSE_RESPONSE_TO_VIRUS_BY_VIRUS
GOBP_REGULATION_OF_DEFENSE_RESPONSE_TO_VIRUS_BY_VIRUS
GOBP_REGULATION_OF_DEFENSE_RESPONSE_TO_VIRUS_BY_VIRUS 29 0.623414887
1.665997018 0.004716981 0.047330189 0.037457795 4214 tags=59%, list=25%, signal=44%
FYN/HCK/AP1S3/LCK/DOCK2/AP1S2/ELMO1/AP1M1/CD4/AP1G1/PACS1/AP2B1/CD247/AP2S1/ARF1/CD8B/AP2A2

GOBP_CELLULAR_RESPONSE_TO_INORGANIC_SUBSTANCE
GOBP_CELLULAR_RESPONSE_TO_INORGANIC_SUBSTANCE
GOBP_CELLULAR_RESPONSE_TO_INORGANIC_SUBSTANCE 202 -0.333134636 -1.35850993
0.004761905 0.047534227 0.037619274 1691 tags=18%, list=10%, signal=17%
LGMN/GLRA2/MT3/CAPN3/CPNE2/CEBPA/NQO1/CYP1A1/PRKAA1/FMR1/CDH1/AKR1C3/OGG1/SLC25A24/MT2A/JUND/SYT17/SLC25A23/MT1HL1/ALAD/MT1E/AOC1/MT1X/MT1H/APP/JUN/P2RX4/MT1G/MT1F/HMOX1/PPARGC1A/EDN1/FOSB/ANK3/MT1M/SLC30A10/CHP2

GOBP_MEMBRANE_RAFT_ORGANIZATION GOBP_MEMBRANE_RAFT_ORGANIZATION
GOBP_MEMBRANE_RAFT_ORGANIZATION 24 0.66827052 1.715674379 0.004769475
0.047534227 0.037619274 3066 tags=50%, list=18%, signal=41%

CAV1/RFTN1/PTPRC/CAV2/NPC1/PPT1/DOCK2/CD2/ANXA2/MAL/EMP2/FA2H
GOBP_POSITIVE_REGULATION_OF_LEUKOCYTE_APOPTOTIC_PROCESS
GOBP_POSITIVE_REGULATION_OF_LEUKOCYTE_APOPTOTIC_PROCESS
GOBP_POSITIVE_REGULATION_OF_LEUKOCYTE_APOPTOTIC_PROCESS 24 0.670312142 1.720915907 0.004769475 0.047534227 0.037619274 246 tags=21%, list=1%, signal=21% ANXA1/CD274/IDO1/WNT5A/LYN
GOBP_POSITIVE_REGULATION_OF_ANTIGEN_RECEPTOR_MEDIATED_SIGNALING_PATHWAY
GOBP_POSITIVE_REGULATION_OF_ANTIGEN_RECEPTOR_MEDIATED_SIGNALING_PATHWAY
GOBP_POSITIVE_REGULATION_OF_ANTIGEN_RECEPTOR_MEDIATED_SIGNALING_PATHWAY 21 0.705057371 1.765560125 0.004784689 0.047534227 0.037619274 3861 tags=71%, list=23%, signal=55% ADA/PTPRC/KCNN4/CCR7/CD81/RAB29/LCK/PRKCB/PRKCH/SLC39A10/RELA/CARD11/PRKD2/NECTIN2/STAP1
GOBP_STEROID_HORMONE_SECRETION GOBP_STEROID_HORMONE_SECRETION GOBP_STEROID_HORMONE_SECRETION 21 0.696125567 1.743193666 0.004784689 0.047534227 0.037619274 2994 tags=43%, list=18%, signal=35% AGT/SELENOM/INHBA/GAL/FZD4/BMP6/C1QTNF1/CRY1/PTPN11
GOBP_CELLULAR_RESPONSE_TO_STEROID_HORMONE_STIMULUS
GOBP_CELLULAR_RESPONSE_TO_STEROID_HORMONE_STIMULUS
GOBP_CELLULAR_RESPONSE_TO_STEROID_HORMONE_STIMULUS 192 -0.336542272 -1.354920708 0.004807692 0.047556907 0.037637223 2285 tags=24%, list=13%, signal=21%
WBP2/UFL1/EGFR/RNF6/USP8/TAF7/CALCOCO1/SCGB2A1/EP300/ADTRP/MYOD1/ESRRA/NCOA4/SSTR2/LATS1/NR3C1/RXRA/ARNTL/YWHAH/PGRMC2/PAK1/ZFP36L2/SIRT1/RNF14/HEY1/KDM3A/PPARA/AKR1C3/PCK2/SGK1/SRD5A1/ZMIZ1/ZFP36/PPARD/ATP2B1/PAQR8/BCL2L11/NR3C2/PPARGC1B/FBXO32/PLPP1/SCNN1B/KLF9/PADI2/EDN1/PCK1
GOBP_REGULATION_OF_VASCULAR_ENDOTHELIAL_GROWTH_FACTOR_RECEPTOR_SIGNALING_PATHWAY
GOBP_REGULATION_OF_VASCULAR_ENDOTHELIAL_GROWTH_FACTOR_RECEPTOR_SIGNALING_PATHWAY
GOBP_REGULATION_OF_VASCULAR_ENDOTHELIAL_GROWTH_FACTOR_RECEPTOR_SIGNALING_PATHWAY 26 0.644721884 1.682707635 0.004815409 0.047556907 0.037637223 2377 tags=50%, list=14%, signal=43% IL1B/GRB10/HIF1A/TMEM204/ITGA5/HHEX/MMRN2/VEGFC/EMILIN1/PRKCB/NEDD4/FZD4/PTPN1
GOBP_T_CELL_CHEMOTAXIS GOBP_T_CELL_CHEMOTAXIS GOBP_T_CELL_CHEMOTAXIS 23 0.670323143 1.709791689 0.004815409 0.047556907 0.037637223 1338 tags=39%, list=8%, signal=36% CXCL11/CXCL13/CXCL10/WNT5A/CCR2/S100A7/PIK3CG/GPR183/PIK3CD
GOBP_CARNITINE_METABOLIC_PROCESS GOBP_CARNITINE_METABOLIC_PROCESS GOBP_CARNITINE_METABOLIC_PROCESS 12 -0.801331301 -1.913505312 0.004842615 0.047638038 0.037701432 1901 tags=58%, list=11%, signal=52% CRAT/TMLHE/CPT2/CROT/CPT1A/ACADM/SLC22A4
GOBP_DRUG_TRANSPORT GOBP_DRUG_TRANSPORT GOBP_DRUG_TRANSPORT 12

-0.754495002 -1.801664546 0.004842615 0.047638038 0.037701432 2177 tags=50%, list=13%, signal=44% SLC19A1/ABCC3/ABCC6/ATP8B1/SLC46A1/ABCB11

GOBP_REGULATION_OF_CELLULAR_RESPONSE_TO_GROWTH_FACTOR_STIMULUS GOBP_REGULATION_OF_CELLULAR_RESPONSE_TO_GROWTH_FACTOR_STIMULUS GOBP_REGULATION_OF_CELLULAR_RESPONSE_TO_GROWTH_FACTOR_STIMULUS 260 0.423090326 1.544199219 0.004854369 0.047660213 0.037718982 2503 tags=27%, list=15%, signal=23% GREM1/CXCL13/IL1B/FSTL1/AGT/WNT5A/CAV1/ROBO1/ENG/CCN1/HTRA1/CDH5/DKK3/FBN1/TGFB1/SULF1/CHST11/GRB10/HIF1A/TMEM204/PMEPA1/ITGA5/TGFBR2/SMOC2/HSPA5/DCN/TGFB1I1/CAV2/LOX/LATS2/HHEX/SULF2/MYOCD/SKIL/CREB3L1/MMRN2/VEGFC/DAB2/EMILIN1/PELO/XDH/ADGRA2/SPRED1/FGFR1/LRG1/PRKCB/TWSG1/NRROS/CFLAR/FST/SDCBP/CD109/FGFBP1/NEDD4/SFRP1/ATP2B4/CDKN1C/ZNF451/FZD4/WNT4/TGFBR1/SFRP2/STRAP/ASPN/PTPN1/VASN/FERMT1/FSTL3/HSP90AB1/TGFB3

GOBP_RECEPTOR_MEDIATED_ENDOCYTOSIS GOBP_RECEPTOR_MEDIATED_ENDOCYTOSIS GOBP_RECEPTOR_MEDIATED_ENDOCYTOSIS 254 0.422166011 1.538453692 0.00486618 0.047682862 0.037736906 2945 tags=26%, list=17%, signal=22% GREM1/CXCL8/SPARC/RAB31/CXCR2/CAV1/C3/SERPINE1/HYOU1/HBB/FCGR1B/CLU/RAMP3/FCER1G/CD163/HSPH1/APOL1/TGFBR2/ITGB2/FCGR2B/CAV2/CALCRL/SELE/PPT1/PLCG2/CD81/PCSK9/ITGAM/DAB2/LDLRAD3/CALR/MRC1/APOC1/FCHSD2/MX2/DKK1/CCL19/WDR54/HSP90B1/SLC9B2/ANXA2/ITGAV/CLTB/SDCBP/AHI1/DNAJC6/CTSL/LILRB4/NEDD4/LILRB1/FPR2/MX1/SYT11/CXCL16/RAMP2/PIP5K1C/CANX/GRK3/SYK/DLL1/HIP1/ITGA4/ARRB2/APOE/ADRB2/AP1G1/HSP90AA1

GOBP_TISSUE_HOMEOSTASIS GOBP_TISSUE_HOMEOSTASIS GOBP_TISSUE_HOMEOSTASIS 246 0.42023332 1.526139411 0.004878049 0.047705986 0.037755207 2191 tags=24%, list=13%, signal=22% LPCAT1/GJA1/SERPINA3/SPP1/PECAM1/CLDN1/CD38/TFF1/CDH3/ZG16B/LAMC1/TFF2/CTSH/CDH5/AZGP1/RAC2/TNFRSF11B/MFSD2A/LDB2/F2R/TCIRG1/MUC4/HOMER1/GPR137B/VSIG1/ADGRF5/SASH3/SNX10/ABCA12/CORO1A/LTF/JAM2/S1PR1/WWTR1/LYZ/CLDN18/AKR1B1/CSF1R/CLDN12/LIPA/BARD1/JAM3/ACP5/VCL/ACACA/AKT3/IL6/ADAM8/MUC2/RAB3D/DEF8/TMEM119/RBP4/TMEM64/TNFSF11/P2RX7/NOD2/INPP5D/ESAM/STRAP

GOBP_POSITIVE_REGULATION_OF_DNA_BINDING_TRANSCRIPTION_FACTOR_ACTIVITY GOBP_POSITIVE_REGULATION_OF_DNA_BINDING_TRANSCRIPTION_FACTOR_ACTIVITY GOBP_POSITIVE_REGULATION_OF_DNA_BINDING_TRANSCRIPTION_FACTOR_ACTIVITY 257 0.420823591 1.530981273 0.004914005 0.047841704 0.037862616 2780 tags=27%, list=16%, signal=23% S100A8/GREM1/S100A9/IL1B/S100A12/AGT/WNT5A/RGCC/CAV1/TRIM22/LRP8/HCLS1/AIM2/ANXA3/CLU/RIPK2/STING1/CARD16/KIT/ITGB2/JAK2/SHH/TRIM27/TLR2/ICAM1/IRAK1/MYOCD/CD40/DDR2/CRTC3/LTF/TRIM15/MTDH/STAT3/PPRC1/TRAF5/SPHK1/PRKCB/IL18R1/TCF3/IRAK2/ADGRF1/BTK/PRKCH/CD200/CFLAR/IL6/ADAM8/STK3/ARHGEF2/SLCO3A1/RNF25/FZD4/RELA/TNFSF11/PPP3CA/NOD2/TRAF1/NFKB2/MALT1/WNT2/ARID5B/AKT1/PARK7/NPM1/IL18/UBE2N/TRIM5/TRIM21

GOBP_CHONDROITIN_SULFATE_CATABOLIC_PROCESS GOBP_CHONDROITIN_SULFATE_CATABOLIC_PROCESS GOBP_CHONDROITIN_SULFATE_CATABOLIC_PROCESS 14 0.742375646 1.698886422

0.004934211 0.047841704 0.037862616 663 tags=29%, list=4%, signal=27% HYAL1/BGN/VCAN/DCN
GOBP_POSITIVE_REGULATION_OF_KERATINOCYTE_PROLIFERATION GOBP_POSITIVE_REGULATION_OF_KERATINOCYTE_PROLIFERATION GOBP_POSITIVE_REGULATION_OF_KERATINOCYTE_PROLIFERATION 14 0.756424066 1.731035468 0.004934211 0.047841704 0.037862616 174 tags=14%, list=1%, signal=14% REG3A/CDH3
GOBP_RESPONSE_TO_TOXIC_SUBSTANCE GOBP_RESPONSE_TO_TOXIC_SUBSTANCE GOBP_RESPONSE_TO_TOXIC_SUBSTANCE 218 -0.342574841 -1.403959208 0.004950495 0.047841704 0.037862616 2061 tags=27%, list=12%, signal=24% GSR/GLYAT/AKR7A3/SLC22A8/ADH4/TP53INP1/GSTM1/MGST3/KDM5B/GPX4/ERCC6/MT3/GSTM2/PRDX5/NQO1/PINK1/BPHL/CYP1A1/SLC6A4/GSTM3/SESN1/BCL2/CDH1/PIM1/MT2A/IYD/SRD5A1/GUCY2C/NNT/CAT/CHKA/AKR1B10/SCN9A/CCL5/APOM/EPHX1/SLC30A1/MT1HL1/MPST/ALAD/SLC30A4/MT1E/FABP1/PPP1R9A/MT1X/MT1H/DDC/PRXL2A/PRDX6/MT1G/PDZD3/MT1F/EPHX2/SLC23A1/PDZK1/SLC22A5/MT1M/SLC30A10/TRPM6/ABCG2
GOBP_POSITIVE_REGULATION_OF_LYMPHOCYTE_APOPTOTIC_PROCESS GOBP_POSITIVE_REGULATION_OF_LYMPHOCYTE_APOPTOTIC_PROCESS GOBP_POSITIVE_REGULATION_OF_LYMPHOCYTE_APOPTOTIC_PROCESS 15 0.709974758 1.657010982 0.004958678 0.047841704 0.037862616 113 tags=20%, list=1%, signal=20% CD274/IDO1/WNT5A
GOBP_POSITIVE_REGULATION_OF_T_HELPER_1_TYPE_IMMUNE_RESPONSE GOBP_POSITIVE_REGULATION_OF_T_HELPER_1_TYPE_IMMUNE_RESPONSE GOBP_POSITIVE_REGULATION_OF_T_HELPER_1_TYPE_IMMUNE_RESPONSE 15 0.712935925 1.663922053 0.004958678 0.047841704 0.037862616 1645 tags=53%, list=10%, signal=48% IL1B/CCR2/IL27RA/RIPK2/IL1R1/IL12RB1/IL18R1/IL23A
GOBP_REGULATION_OF_T_CELL_CHEMOTAXIS GOBP_REGULATION_OF_T_CELL_CHEMOTAXIS GOBP_REGULATION_OF_T_CELL_CHEMOTAXIS 15 0.710541825 1.658334459 0.004958678 0.047841704 0.037862616 486 tags=33%, list=3%, signal=32% CXCL13/CXCL10/WNT5A/CCR2/S100A7
GOBP_CELLULAR_LIPID_CATABOLIC_PROCESS GOBP_CELLULAR_LIPID_CATABOLIC_PROCESS GOBP_CELLULAR_LIPID_CATABOLIC_PROCESS 215 -0.495993799 -2.022314301 0.004975124 0.047908251 0.037915283 2302 tags=34%, list=14%, signal=30% IVD/PNPLA7/ECHS1/ETFBKMT/ADTRP/IRS2/ACAD10/PNPLA4/PECR/SCARB1/CRAT/FAAH/SCP2/FABP2/PLA2G4C/NEU4/DECR1/MT3/ECHDC2/ABHD5/ETFB/PEX13/HADHB/DDHD2/LIPH/ACBD5/LPIN2/ECI1/PPARA/AKR1C3/PCCA/ETFA/PCK2/ECH1/ABHD12/FUCA1/AKR1B10/PPARD/ACAA1/ACOT8/ACOX2/ABCD3/BDH2/HADH/GDE1/HADHA/ACAA2/PNLIPRP2/ABHD3/CPT2/LIPC/FABP1/MGLL/NEU1/CROT/NAPEPLD/CPT1A/ACADS/AUH/SORL1/EHHADH/PRDX6/BCO1/GDPD1/PHYH/NUDT7/ACADM/ACAT1/ETFDH/ABCB11/ACOX1/GBA3/PCK1
GOBP_POSITIVE_REGULATION_OF_PROTEIN_CATABOLIC_PROCESS GOBP_POSITIVE_REGULATION_OF_PROTEIN_CATABOLIC_PROCESS GOBP_POSITIVE_REGULATION_OF_PROTEIN_CATABOLIC_PROCESS 216 0.409541103 1.469134588 0.004993758 0.047995561 0.037984381 3731 tags=32%, list=22%, signal=25%
C4BPB/LPCAT1/C4BPA/GJA1/IL1B/WNT5A/TRIB2/CAV1/IL33/MSN/BAG2/CLU/ECSCR/RNF14

4B/CDC20/DACT1/CD81/VIP/PCSK9/LAPTM5/DAB2/VCP/CSNK1E/CTSC/PSMC2/ADAM9/AURKA/BARD1/TNFRSF1B/PLK1/OAZ3/ADAM8/NEDD4/IFNG/PSMC4/RDX/PLK2/NRDC/FURIN/SEC22B/RNF19B/HECW2/LRRK2/FBXW7/AKT1/HSPBP1/SGSM3/DVL1/APOE/STX5/USP13/CBFA2T3/SOCS4/SMURF1/HSP90AA1/IDE/RAD23A/HERPUD1/DDB1/PRICKLE1/FBXO22/PSMC1/SMURF2/LDLR/DTL/MDM2/DDRGK1/USP5/SOX9

GOBP_RESPONSE_TO_MECHANICAL_STIMULUS GOBP_RESPONSE_TO_MECHANICAL_STIMULUS GOBP_RESPONSE_TO_MECHANICAL_STIMULUS 196 0.410016629 1.452242156 0.005031447 0.048233892 0.038172999 1204 tags=16%, list=7%, signal=15% CHI3L1/TNC/GJA1/CXCL10/IL1B/MMP7/COL1A1/CASP1/COL3A1/SERPINE2/ITGA2/ENG/TRPA1/BAG3/BNIP3/TLR8/CCNB1/KIT/TGFBR2/FYN/CXCR4/DCN/POSTN/PIEZO1/CD40/ASNS/TNFRSF10A/CASP5/IGFBP2/IRF1/TNFRSF10B/RIPOR2

GOBP_GLIAL_CELL_DIFFERENTIATION GOBP_GLIAL_CELL_DIFFERENTIATION GOBP_GLIAL_CELL_DIFFERENTIATION 201 0.416674225 1.478845948 0.005037783 0.048233892 0.038172999 3110 tags=27%, list=18%, signal=23% S100A8/S100A9/ADGRG6/BACE2/SERPINE2/LYN/TGFB1/CLU/BNIP3/BOK/C5AR1/VIM/SHH/CXCR4/TLR2/LEF1/C1QA/STAT3/TSPAN2/IL6ST/CDK1/METTL3/DUSP10/TNFRSF1B/LAMB2/NRROS/MXRA8/PHGDH/LIF/CDKN2C/IL6/EIF2B3/MAP2K1/IFNG/PRMT5/FPR2/DICER1/ITGB4/RELA/NAB1/EIF2B2/B4GALT6/MDK/NAB2/AKT1/DLL1/SLC45A3/GPC1/EIF2B5/ARHGEF10/PTPN11/EIF2B4/FA2H/GSTP1/NFIB

GOBP_ESTABLISHMENT_OF_RNA_LOCALIZATION GOBP_ESTABLISHMENT_OF_RNA_LOCALIZATION GOBP_ESTABLISHMENT_OF_RNA_LOCALIZATION 183 0.419166132 1.477518623 0.005082592 0.048570218 0.038439173 4717 tags=49%, list=28%, signal=36% RFTN1/QKI/TGFBR2/HHEX/SEC13/MAGOHB/XPOT/RRS1/DDX39A/MX2/PABPN1/NUP62/ABCE1/RAN/ATR/DDX19A/NUP98/IGF2BP3/NUP85/NXT1/CETN2/POLR2D/SRSF7/MCM3AP/SDAD1/NUP93/NUP210/RAE1/NUP37/SMG7/LTV1/NSUN2/THOC1/NPM1/NUP62CL/NXF3/NUP58/CPSF3/SFPQ/PNPT1/ALYREF/NUP205/UPF1/SLBP/EIF4A3/ALKBH5/UPF2/AAAS/IWS1/NUP50/RBM8A/FYTTD1/SRSF1/ZFP36L1/CHTOP/LSG1/RBM15B/NOP9/NUP35/NUP155/NUP107/SUPT6H/SRSF3/AGFG1/POM121L12/RANBP2/XPO5/MRPL18/NOL6/SRSF4/SRSF6/EIF5A2/NDC1/NUP188/NUP88/THOC7/THOC5/NUP54/U2AF1/NXF1/ATM/HNRNPU/ENY2/CKAP5/RTRAF/SRRM1/NUP214/CPSF6/SYMPK/XPO1

GOBP_RESPONSE_TO_PARATHYROID_HORMONE GOBP_RESPONSE_TO_PARATHYROID_HORMONE GOBP_RESPONSE_TO_PARATHYROID_HORMONE 12 0.752553243 1.648771199 0.005093379 0.048580761 0.038447517 254 tags=17%, list=2%, signal=16% GJA1/ITGA2

GOBP_SODIUM_ION_TRANSPORT GOBP_SODIUM_ION_TRANSPORT GOBP_SODIUM_ION_TRANSPORT 227 -0.353510334 -1.457503962 0.005154639 0.049071774 0.038836111 1584 tags=15%, list=9%, signal=13% TRPM4/DMD/SLC5A11/PKP2/YWHAH/SLC6A4/PRSS8/NEDD4L/SGK1/ATP1B3/SLC20A1/SCN9A/CNNM4/GPD1L/FXYD3/SLC20A2/SHROOM2/SLC9A3R1/P2RX4/SLC9A2/SLC22A4/ABCB11/NETO2/SLC9A3/SCNN1B/SLC6A19/SLC23A1/SLC4A4/SLC22A5/CNKSR3/ANK3/SLC17A4/SLC38A4

GOBP_SHORT_CHAIN_FATTY_ACID_METABOLIC_PROCESS GOBP_SHORT_CHAIN_FATTY_ACID_METABOLIC_PROCESS

GOBP_SHORT_CHAIN_FATTY_ACID_METABOLIC_PROCESS 17 -0.702885156 -1.811471803 0.005167959 0.049105395 0.038862719 2142 tags=53%, list=13%, signal=46% ACSS1/CRAT/PCCA/PCK2/ACSS2/ACOT4/ACADS/PHYH/PCK1

GOBP_GASTRULATION GOBP_GASTRULATION GOBP_GASTRULATION 165 0.435431972 1.513573987 0.005194805 0.049267179 0.038990758 3043 tags=28%, list=18%, signal=23% IL1RN/GJA1/COL12A1/WNT5A/MMP9/COL5A2/MMP2/COL6A1/ITGA2/LAMA3/COL4A2/INHBA/COL5A1/APLNR/FN1/ITGA5/DUSP4/TGFBR2/ITGB2/FOXF1/COL8A1/LEF1/ITGA8/TRIM15/DKK1/LAMB1/COL7A1/TWSG1/EXT2/ACVR1/ITGAV/SFRP1/PHLDB2/ITGB4/SOX7/SFRP2/PAF1/POGLUT1/FOXA2/ITGA4/PRKAR1A/DVL1/NR4A3/ITGA7/HOXA11/ITGA3

GOBP_DRUG_CATABOLIC_PROCESS GOBP_DRUG_CATABOLIC_PROCESS GOBP_DRUG_CATABOLIC_PROCESS 23 -0.710448872 -1.956496189 0.005277045 0.049952706 0.039533293 2071 tags=26%, list=12%, signal=23% NUDT15/CYP2J2/NR1I2/FMO4/CYP2S1/CYP2B6

GOBP_IMPORT_INTO_NUCLEUS GOBP_IMPORT_INTO_NUCLEUS GOBP_IMPORT_INTO_NUCLEUS 150 0.44719222 1.533804991 0.005326232 0.050201376 0.039730094 4467 tags=51%, list=26%, signal=38% MMP12/HCLS1/TGFB1/BAG3/JAK2/SHH/IPO4/KPNA2/SEC13/HYAL2/FLNA/STAT3/RAB23/TNPO1/ECT2/NUP62/TNPO2/RAN/NOLC1/E2F3/NUP98/IPO9/HIKESHI/PML/SNRPG/ZPR1/PKIA/NUP85/SNRPD1/IFNG/NXT1/KPNB1/APOD/PPP3CA/CSE1L/NUP93/LRRK2/SNRPF/KPNA6/FERMT1/HSP90AB1/TRIM28/SYK/AKT1/SNRPB/GEMIN6/GEMIN8/TARDBP/NUP62CL/GEMIN5/HEATR3/NUP58/ELAVL1/PRICKLE1/UFM1/MBTPS1/IPO7/SNRPD2/NUP50/PKIG/NUP35/KPNA1/NUP155/NUP107/DDX20/IPO13/SNRPE/POM121L12/RANBP2/BACH2/NUP188/NUP88/MDFIC/GEMIN4/NUP54/KPNA3/GCKR

GOBP_CELLULAR_RESPONSE_TO_ZINC_ION GOBP_CELLULAR_RESPONSE_TO_ZINC_ION GOBP_CELLULAR_RESPONSE_TO_ZINC_ION 21 -0.716344823 -1.909909443 0.005333333 0.050201376 0.039730094 1620 tags=52%, list=10%, signal=47% GLRA2/MT3/MT2A/MT1HL1/MT1E/MT1X/MT1H/P2RX4/MT1G/MT1F/MT1M

GOBP_ETHANOL_METABOLIC_PROCESS GOBP_ETHANOL_METABOLIC_PROCESS GOBP_ETHANOL_METABOLIC_PROCESS 20 -0.738190717 -1.951366092 0.005333333 0.050201376 0.039730094 2423 tags=60%, list=14%, signal=51% ADH5/ALDH1B1/ACSS1/ADH4/ALDH1A1/ACSS2/ADH1A/SULT1A1/ADH6/SULT1A2/SULT1B1/ADH1C

GOBP_ADENYLATE_CYCLASE_ACTIVATING_G_PROTEIN_COUPLED_RECEPTOR_SIGNALING_PATHWAY GOBP_ADENYLATE_CYCLASE_ACTIVATING_G_PROTEIN_COUPLED_RECEPTOR_SIGNALING_PATHWAY GOBP_ADENYLATE_CYCLASE_ACTIVATING_G_PROTEIN_COUPLED_RECEPTOR_SIGNALING_PATHWAY 138 0.458995508 1.565025714 0.005434783 0.050695971 0.040121524 2879 tags=22%, list=17%, signal=19% CXCL9/CXCL11/ADGRG6/CXCL10/ADGRL4/PDE4B/PF4/S1PR3/MRAP2/RAMP3/ADGRE2/ADGRL2/PLN/CALCRL/GNAI2/VIP/S1PR1/ADGRE5/ADGRF1/PTGFR/GPR65/ADCY4/ATP2B4/GPR4/RAMP2/PTGER2/ADCY7/ADGRE3/ARRDC3/ADGRE1/ADRB2

GOBP_RESPONSE_TO_IONIZING_RADIATION GOBP_RESPONSE_TO_IONIZING_RADIATION

GOBP_RESPONSE_TO_IONIZING_RADIATION 138 0.452428878 1.542635639 0.005434783 0.050695971 0.040121524 3466 tags=37%, list=20%, signal=30% ANXA1/CXCL10/THBD/IKBIP/IFI16/TIGAR/PTPRC/VCAM1/HSPA5/NABP1/ICAM1/H2AX/MAP3K20/BLM/ECT2/EYA3/FANCD2/CCND1/AEN/ATR/TMEM109/PML/TNKS1BP1/CRYAB/SFRP1/PARP1/FIGNL1/SFRP2/RRM1/UIMC1/RAD1/GPX1/CCL7/NABP2/FBXO4/TANK/POLB/TSPYL5/ABCG5/MTA1/RNF8/HRAS/EEF1D/INTS3/RAD54L/BRCA1/POLG/XRCC5/BAK1/RAD51AP1/CLK2

GOBP_RESPONSE_TO_UV GOBP_RESPONSE_TO_UV GOBP_RESPONSE_TO_UV 141 0.463095968 1.580709062 0.005464481 0.050695971 0.040121524 3450 tags=34%, list=20%, signal=27% MMP1/MMP3/TIMP1/MMP9/MMP2/HYAL1/FEN1/CARD16/POLD3/HYAL2/CDC25A/EIF2S1/PCLAF/MME/AURKB/METTL3/CCND1/PBK/ATR/UVSSA/TIPIN/PML/MSH6/NEDD4/PARP1/SCARA3/RELA/MFAP4/NOC2L/AQP1/CHEK1/GPX1/RUVBL2/ATF4/AKT1/NPM1/DDB2/COPS9/TRIAP1/CCAR2/RO60/TMEM161A/HYAL3/DDB1/USF1/ACTR5/USP47/BAK1

GOBP_CGMP_MEDIATED_SIGNALING GOBP_CGMP_MEDIATED_SIGNALING GOBP_CGMP_MEDIATED_SIGNALING 28 -0.600676927 -1.706125954 0.005479452 0.050695971 0.040121524 377 tags=14%, list=2%, signal=14% PDE3A/PDZD3/THBS1/GUCA2B

GOBP_FORMATION_OF_PRIMARY_GERM_LAYER GOBP_FORMATION_OF_PRIMARY_GERM_LAYER GOBP_FORMATION_OF_PRIMARY_GERM_LAYER 105 0.504169789 1.655217629 0.005479452 0.050695971 0.040121524 1602 tags=27%, list=9%, signal=24% GJA1/COL12A1/WNT5A/MMP9/COL5A2/MMP2/COL6A1/ITGA2/LAMA3/COL4A2/INHBA/COL5A1/FN1/ITGA5/DUSP4/ITGB2/FOXF1/COL8A1/LEF1/ITGA8/TRIM15/DKK1/LAMB1/COL7A1/TWSG1/EXT2/ACVR1/ITGAV

GOBP_PHOTOTRANSDUCTION_VISIBLE_LIGHT GOBP_PHOTOTRANSDUCTION_VISIBLE_LIGHT GOBP_PHOTOTRANSDUCTION_VISIBLE_LIGHT 34 -0.568838326 -1.687222137 0.005479452 0.050695971 0.040121524 430 tags=12%, list=3%, signal=11% GNA11/CNGA1/NMT2/PDE6A

GOBP_REGULATION_OF_CELL_MATRIX_ADHESION GOBP_REGULATION_OF_CELL_MATRIX_ADHESION GOBP_REGULATION_OF_CELL_MATRIX_ADHESION 116 0.489471899 1.625760034 0.005486968 0.050695971 0.040121524 3618 tags=38%, list=21%, signal=30% MMP12/GREM1/PLAU/FERMT2/SERPINE1/EFEMP2/CCR7/DLC1/THY1/MAP4K4/POSTN/BCL6/KDR/COL16A1/TEK/LIMCH1/ITGB1BP1/NRP1/MACF1/CORO1C/DUSP22/ADAM15/VCL/PLEKHA2/RCC2/SFRP1/JAG1/APOD/PHLDB2/WNT4/CEACAM6/FERMT1/FAM107A/ROCK1/PPM1F/EMP2/CDH13/GPM6B/LDB1/NF2/CX3CL1/EPHA1/DUSP3/MMP14

GOBP_ORGANIC_ACID_CATABOLIC_PROCESS GOBP_ORGANIC_ACID_CATABOLIC_PROCESS GOBP_ORGANIC_ACID_CATABOLIC_PROCESS 246 -0.459178521 -1.885004098 0.005494505 0.050695971 0.040121524 2304 tags=34%, list=14%, signal=30% TAT/IVD/ECHS1/DAO/ETFBKMT/ALDH6A1/OTC/SDSL/RIDA/ADTRP/IRS2/ACAD10/PECR/DDO/CYP4F3/CRAT/FAAH/SCP2/GLUD1/DLD/GPT/DECR1/ECHDC2/HMGCL/ETFB/PEX13/HADHB/ACBD5/LPIN2/HIBCH/GLS/ECI1/PPARA/PCCA/ETFA/PCK2/GLUD2/PHYKPL/QPRT/AMT/ECH1/PPARD/ACAA1/ACOT8/ACOX2/ABCD3/BDH2/ACOT4/HADH/CRYL1/HADHA/BCKDHB/TST/MPST/ACAA2/ABHD3/BCAT2/CYP4F12/CPT2/ACADSB/LDHD/FABP1/CROT/ASPA/CYP4F2/CBS/CTH/ALDH5A1/HNMT/C

PT1A/ACADS/AUH/EHHADH/PHYH/NUDT7/ACADM/ACAT1/ETFDH/DDAH2/ABCB11/ABAT/ACOX1/PCK1

GOBP_SULFUR_COMPOUND_TRANSPORT GOBP_SULFUR_COMPOUND_TRANSPORT GOBP_SULFUR_COMPOUND_TRANSPORT 47 -0.535791105 -1.728093591 0.005494505 0.050695971 0.040121524 301 tags=17%, list=2%, signal=17% SLC9A3R1/SLC26A3/SLC36A1/SLC1A1/SLC19A3/SLC3A1/SLC26A2/ABCG2

GOBP_ESTABLISHMENT_OF_TISSUE_POLARITY GOBP_ESTABLISHMENT_OF_TISSUE_POLARITY GOBP_ESTABLISHMENT_OF_TISSUE_POLARITY 120 0.477901401 1.595532241 0.005509642 0.050695971 0.040121524 4282 tags=50%, list=25%, signal=38% CTHRC1/WNT5A/RSPO3/PSME4/PSMB9/DACT1/DAB2/PSMD14/PSMD12/PSMC2/DKK1/PSMB2/PSMA3/PSMD1/GPC6/PSMA5/SFRP1/PSMD6/CELSR1/PSMB5/PSMC4/VANGL1/FZD4/SFRP2/PSMB1/PSMA1/FOXF2/PSME3/PLEKHA4/PSMA7/PSMB8/PSMC5/ARRB2/DVL1/SMURF1/PSMB10/PSMB6/PSMA4/AP2B1/PSMD13/PSME1/PRICKLE1/SAPCD2/PSMC1/SMURF2/DVL3/PSMB4/ARHGEF19/AP2S1/PSMB3/PSMD2/PSMC6/PSMD7/PSMA2/ANKRD6/PSMD4/AP2A2/PSMD9/IFT20/TIAM1

GOBP_GLAND_MORPHOGENESIS GOBP_GLAND_MORPHOGENESIS GOBP_GLAND_MORPHOGENESIS 106 0.489564781 1.605650777 0.005509642 0.050695971 0.040121524 2082 tags=26%, list=12%, signal=23% SERPINB5/IGFBP5/TNC/CCL11/WNT5A/TGM2/CAV1/MSN/TGFB1/SULF1/PLXNA1/XBP1/TGFBR2/SHH/CEBPB/SULF2/CSF1R/TWSG1/NKX2-3/CFLAR/PLXND1/PML/IL6/SFRP1/LAMA5/FGF7/WNT4/HOXB13

GOBP_POSITIVE_REGULATION_OF_CELL_SUBSTRATE_ADHESION GOBP_POSITIVE_REGULATION_OF_CELL_SUBSTRATE_ADHESION GOBP_POSITIVE_REGULATION_OF_CELL_SUBSTRATE_ADHESION 120 0.477111336 1.592894511 0.005509642 0.050695971 0.040121524 3504 tags=36%, list=21%, signal=29% OLFM4/NID1/FERMT2/CCN1/FN1/ITGA5/EFEMP2/CCR7/JAK2/THY1/MAP4K4/FOXF1/EGFL6/FLNA/COL8A1/KDR/COL16A1/P4HB/EMILIN1/TEK/CALR/SPOCK2/AGR2/NRP1/ITGA6/PLEKHA2/SFRP1/WNT4/NPNT/CEACAM6/FERMT1/MDK/FBLN2/ROCK1/PPM1F/EMP2/ITGA3/HSD17B12/STK4/CDH13/CX3CL1/EPHA1/UNC13D

GOBP_AMINOGLYCAN_BIOSYNTHETIC_PROCESS GOBP_AMINOGLYCAN_BIOSYNTHETIC_PROCESS GOBP_AMINOGLYCAN_BIOSYNTHETIC_PROCESS 114 0.493038836 1.631961325 0.005517241 0.050695971 0.040121524 2288 tags=31%, list=14%, signal=27% CEMIP/IL1B/CSGALNACT1/CHST15/HYAL1/TGFB1/BGN/CHST2/CHST11/CHSY1/LUM/VCAN/ST3GAL4/DSE/DCN/PDGFRB/CHPF/SDC2/B3GALT6/HS3ST3B1/CSGALNACT2/HS3ST1/GPC6/FMOD/EXT2/CHST12/ST3GAL2/GALNT5/SDC3/AGRN/B3GNT7/B3GNT6/B4GALT2/B4GALT6/HS6ST2

GOBP_ENERGY_DERIVATION_BY_OXIDATION_OF_ORGANIC_COMPOUNDS GOBP_ENERGY_DERIVATION_BY_OXIDATION_OF_ORGANIC_COMPOUNDS GOBP_ENERGY_DERIVATION_BY_OXIDATION_OF_ORGANIC_COMPOUNDS 248 -0.354041523 -1.457803623 0.005617978 0.051442165 0.040712073 3654 tags=42%, list=22%, signal=33% SLC25A13/IMMP2L/AFG1L/KHK/OGDH/ADRB3/NDUFA3/NDUFS1/OGDHL/SNCA/ESRRB/UQCR11/NOA1/NDUFS3/SIRT3/COX10/COX7B/IDH1/NDUFS4/NDUFA13/SDHA/UQCR10/MTFR1L/ND

UFS7/PRELID1/CYC1/ATP5F1D/UQCRFS1/NDUFS2/COX8A/IGF1/ETFRF1/PRLH/COX6A1/PDHA1/IDH3B/COX4I1/UQCRC2/NDUFB10/NDUFB5/PPP1CC/NDUFB9/IDH3A/MDH1/CYCS/PHKB/UQCRC1/NDUFA1/PYGB/IRS2/GHITM/FAHD1/NDUFB1/COX5A/COX6C/GPD1/SLC25A12/DLAT/RB1CC1/INPP5K/SLC25A25/BLOC1S1/ME2/IREB2/PPP1CB/SUCLG1/DLD/NDUFV1/COQ9/PHKA2/MT3/SUCLA2/COX5B/SDHB/PRKAG2/COX15/NDUFA10/PINK1/PID1/ACO2/ETFB/NDUFA2/NDUFB7/ETFA/PGM1/PER2/NNT/CAT/AGL/INSR/SDHD/NDUFA5/SDHAF4/DYRK2/SLC25A23/UGP2/SUCLG2/LYRM7/ME3/ETFDH/ENPP1/PPP1R3B/OPN3/PPARGC1A

GOBP_NEGATIVE_REGULATION_OF_VASCULATURE_DEVELOPMENT GOBP_NEGATIVE_REGULATION_OF_VASCULATURE_DEVELOPMENT GOBP_NEGATIVE_REGULATION_OF_VASCULATURE_DEVELOPMENT 97 0.502144806 1.619990571 0.005633803 0.051442165 0.040712073 2193 tags=33%, list=13%, signal=29% CXCL10/SPINK5/SPARC/ADAMTS9/ADAMTS1/RGCC/ALOX5/SERPINE1/CCR2/COL4A2/PF4/SULF1/ECSCR/PTPRM/DCN/STAT1/TIE1/HHEX/SERPINF1/CREB3L1/KLF2/EMILIN1/TEK/SPRED1/PML/THBS2/ATP2B4/VASH1/GPR4/PLK2/WNT4/SEMA4A

GOBP_T_CELL_MEDIATED_IMMUNITY GOBP_T_CELL_MEDIATED_IMMUNITY GOBP_T_CELL_MEDIATED_IMMUNITY 94 0.506431187 1.626530928 0.005633803 0.051442165 0.040712073 3362 tags=38%, list=20%, signal=31% CD55/IL1B/IL7R/CTSH/CCR2/RFTN1/AZGP1/HLA-DRA/PTPRC/IL1R1/FCGR2B/SASH3/ICAM1/CD81/CTSC/IL12RB1/IL18R1/TNFRSF1B/IL23A/IL6/MR1/LILRB4/LILRB1/JAG1/BTN3A3/NOD2/MALT1/IL18/HPRT1/WAS/PVR/EMP2/HSPD1/RAB27A/MYO1G/HMGB1

GOBP_LIPID_MODIFICATION GOBP_LIPID_MODIFICATION GOBP_LIPID_MODIFICATION 249 -0.447117389 -1.842233739 0.005649718 0.051442165 0.040712073 2021 tags=33%, list=12%, signal=29% TYSND1/ACACB/ITPKC/CYP4V2/ADH5/INPP5A/SACM1L/SYNJ1/IVD/ECHS1/ETFBKMT/IRS2/ACAD10/PECR/ADH4/CRAT/SCP2/INPP5K/SOCS2/DECR1/ECHDC2/PIK3C2B/PLPP6/PRKAG2/ETFB/CYP1A1/PEX13/HADHB/PRKAA1/ABO/ECI1/PPARA/SPHK2/FAM126B/ETFA/SOAT1/IMPA2/INPP5J/SGPP2/DGKQ/IMPA1/ECH1/OCRL/ABCG1/PPARD/ACAA1/ACOT8/SOCS6/ACOX2/ABCD3/BDH2/HADH/HADHA/ACAA2/CPT2/FABP1/MTMR11/CROT/PIP5K1B/ITPKA/LPCAT3/CPT1A/IP6K2/ACADS/AUH/EHHADH/BPNT1/PHYH/ACADM/ACAT1/ETFDH/PLPP1/ABCB11/MTM1/EPHX2/PDK4/PPARG/B4GALNT2/ACOX1/PPARGC1A/APPL2

GOBP_MULTICELLULAR_ORGANISMAL_HOMEOSTASIS GOBP_MULTICELLULAR_ORGANISMAL_HOMEOSTASIS GOBP_MULTICELLULAR_ORGANISMAL_HOMEOSTASIS497 0.354604655 1.354361418 0.005649718 0.051442165 0.040712073 2241 tags=20%, list=13%, signal=18% LCN2/LPCAT1/GJA1/SERPINA3/SPP1/AQP3/IL1B/PECAM1/CLDN1/CD38/TFF1/CAV1/CDH3/ZG16B/LAMC1/TFF2/CTSH/CDH5/CCR2/SCD/LPIN1/AZGP1/RAC2/IRF4/MRAP2/GRB10/TNFRSF11B/MFSD2A/LDB2/LAMA4/F2R/TCIRG1/PTGS2/PRKAR2B/MUC4/HOMER1/UCP2/GPR137B/VSIG1/JAK2/CEBPB/CXCR4/ADGRF5/SASH3/G0S2/SCNN1G/HYAL2/MET/SNX10/ABCA12/CORO1A/IL1A/CRTC3/LTF/JAM2/S1PR1/STAT3/WWTR1/NMU/LYZ/FFAR4/CLDN18/MFAP2/AKR1B1/CSF1R/CLDN12/AMPD2/LIPA/IL18R1/BARD1/JAM3/TRPV2/WFS1/ADAMTS5/EXT2/TFE3/ACP5/VCL/ACACA/AKT3/IL6/ADAM8/ELOVL6/ADCY4/MUC2/RAB3D/DEF8/TMEM119/EIF4G1/RBP4/TMEM64/TNFSF11/P2RX7/NOD2/INPP5D/ESAM/NRDC/STRAP/PPP1R13L/PDGFC

GOBP_MITOCHONDRIAL_ELECTRON_TRANSPORT_NADH_TO_UBIQUINONE

GOBP_MITOCHONDRIAL_ELECTRON_TRANSPORT_NADH_TO_UBIQUINONE
GOBP_MITOCHONDRIAL_ELECTRON_TRANSPORT_NADH_TO_UBIQUINONE 43
-0.543022094 -1.701732468 0.005665722 0.051494437 0.040753442 4439
tags=58%, list=26%, signal=43%
NDUFB3/NDUFB11/NDUFA6/NDUFB2/NDUFA3/NDUFS1/SNCA/NDUFS3/NDUFS4/NDUFA13/NDUFS7/NDUFS2/NDUFB10/NDUFB5/NDUFB9/NDUFA1/NDUFB1/DLD/NDUFV1/COQ9/NDUFA10/PINK1/NDUFA2/NDUFB7/NDUFA5
GOBP_POSITIVE_REGULATION_OF_PROTEIN_LOCALIZATION_TO_NUCLEUS
GOBP_POSITIVE_REGULATION_OF_PROTEIN_LOCALIZATION_TO_NUCLEUS
GOBP_POSITIVE_REGULATION_OF_PROTEIN_LOCALIZATION_TO_NUCLEUS 82
0.510429245 1.600021425 0.005797101 0.05235695 0.041436047 2977
tags=43%, list=18%, signal=35%
FERMT2/HCLS1/TGFB1/BAG3/PARP9/FYN/JAK2/SHH/HYAL2/FLNA/WWTR1/CDK1/ECT2/PLK1/RAN/LIF/CCT3/ZPR1/PARP1/CCT8/IFNG/CCT5/CCT2/CCT6A/CCT4/CCT7/PINX1/HSP90AB1/TRIM28/CDK5RAP3/GLIS2/AKT1/PARK7/TARDBP/TRIM8
GOBP_RETROGRADE_VESICLE_MEDIATED_TRANSPORT_GOLGI_TO_ENDOPLASMIC_RETICULUM
GOBP_RETROGRADE_VESICLE_MEDIATED_TRANSPORT_GOLGI_TO_ENDOPLASMIC_RETICULUM
GOBP_RETROGRADE_VESICLE_MEDIATED_TRANSPORT_GOLGI_TO_ENDOPLASMIC_RETICULUM 82 0.517149096 1.621085861 0.005797101 0.05235695 0.041436047 3923
tags=45%, list=23%, signal=35%
KDELR3/ARFGAP3/PITPNB/TMED3/KLC2/CENPE/COPB2/SURF4/KIF23/BET1L/TMED9/KIF2C/SCYL1/KIF11/ARF4/KDELR2/SEC22B/COPA/KIF4A/COPZ2/ATP9B/TAPBP/COPB1/ARFGAP1/KDELR1/SCFD1/RACGAP1/RINT1/KIF15/KIF18A/KLC1/ARCN1/COPE/STX18/COPG1/TMED2/COPZ1
GOBP_MYELOID_CELL_DEVELOPMENT GOBP_MYELOID_CELL_DEVELOPMENT
GOBP_MYELOID_CELL_DEVELOPMENT 65 0.534390164 1.624609934 0.005856515
0.05235695 0.041436047 2102 tags=35%, list=12%, signal=31%
FBN1/EVI2B/KIT/ADGRF5/TLR2/TYROBP/LYAR/BCL6/FLI1/KLF2/LTF/TSPAN2/MEIS1/CLDN18/PIP4K2A/NRROS/SLC9B2/ANXA2/LILRB1/PABPC4/JMJD6/NCKAP1L/TNFSF11
GOBP_ASTROCYTE_DIFFERENTIATION GOBP_ASTROCYTE_DIFFERENTIATION
GOBP_ASTROCYTE_DIFFERENTIATION 67 0.530163054 1.618833997 0.005865103
0.05235695 0.041436047 1898 tags=25%, list=11%, signal=23%
S100A8/S100A9/BACE2/SERPINE2/C5AR1/VIM/SHH/C1QA/STAT3/TSPAN2/IL6ST/LAMB2/LIF/IL6/MAP2K1/IFNG/FPR2
GOBP_REGULATION_OF_CHROMOSOME_SEPARATION
GOBP_REGULATION_OF_CHROMOSOME_SEPARATION
GOBP_REGULATION_OF_CHROMOSOME_SEPARATION 68 0.522723668 1.602158018
0.005865103 0.05235695 0.041436047 5156 tags=65%, list=30%, signal=45%
TRIP13/CCNB1/MAD2L2/BUB1/CDC20/NDC80/NEK6/TACC3/PTTG1/CDC6/CENPE/AURKB/BUB1B/PLK1/UBE2C/DLGAP5/CDC27/ZWINT/HECW2/CDC26/CDT1/SPDL1/KLHL22/KNTC1/ANAPC7/PLSCR1/FBXO5/ZNF207/CENPF/BUB3/TTK/CHFR/MAD2L1/DYNC1LI1/CSNK2A2/ATM/ZW10/GEN1/CDK5RAP2/MAD1L1/ANAPC5/CDC16/RIOK2/TPR
GOBP_POSITIVE_REGULATION_OF_PHAGOCYTOSIS
GOBP_POSITIVE_REGULATION_OF_PHAGOCYTOSIS

GOBP_POSITIVE_REGULATION_OF_PHAGOCYTOSIS 61 0.548633153 1.653762535 0.005882353 0.05235695 0.041436047 2123 tags=38%, list=13%, signal=33% IL1B/RAB31/C3/ITGA2/C2/PTPRC/FCER1G/CLEC7A/FCGR2B/CCL2/LYAR/SIRPA/CALR/ANO6/DOCK2/IL15RA/MFGE8/CD47/CD300LF/IFNG/FPR2/NCKAP1L/NOD2

GOBP_NATURAL_KILLER_CELL_MEDIATED_IMMUNITY GOBP_NATURAL_KILLER_CELL_MEDIATED_IMMUNITY GOBP_NATURAL_KILLER_CELL_MEDIATED_IMMUNITY60 0.550781704 1.654408782 0.005899705 0.05235695 0.041436047 3688 tags=42%, list=22%, signal=33% SERPINB9/CADM1/SLAMF7/GZMB/SERPINB4/CORO1A/VAV1/LYST/TUBB/LAG3/LILRB1/HAVCR2/SLAMF6/RNF19B/RASGRP1/IL18/PVR/ARRB2/AP1G1/STAT5B/IL18RAP/RAB27A/UNC13D/NECTIN2/HLA-E

GOBP_METAPHASE_ANAPHASE_TRANSITION_OF_CELL_CYCLE GOBP_METAPHASE_ANAPHASE_TRANSITION_OF_CELL_CYCLE GOBP_METAPHASE_ANAPHASE_TRANSITION_OF_CELL_CYCLE 62 0.538162627 1.620401471 0.005908419 0.05235695 0.041436047 3908 tags=52%, list=23%, signal=40% TRIP13/CCNB1/MAD2L2/BUB1/CDC20/NDC80/NEK6/TACC3/CDC6/CENPE/AURKB/BUB1B/PLK1/UBE2C/DLGAP5/CDC27/ZWINT/HECW2/CDC26/CDT1/SPDL1/KLHL22/KNTC1/ANAPC7/FBXO5/ZNF207/CENPF/BUB3/TTK/CHFR/MAD2L1/DYNC1LI1

GOBP_POSITIVE_REGULATION_OF_INTERFERON_GAMMA_PRODUCTION GOBP_POSITIVE_REGULATION_OF_INTERFERON_GAMMA_PRODUCTION GOBP_POSITIVE_REGULATION_OF_INTERFERON_GAMMA_PRODUCTION 62 0.543255445 1.635735889 0.005908419 0.05235695 0.041436047 2027 tags=32%, list=12%, signal=29% IL1B/WNT5A/SLC7A5/CCR2/PDE4B/IL27RA/RIPK2/CLEC7A/IL1R1/TLR8/SASH3/HLA-DPA1/CD276/IL12RB1/IL18R1/EBI3/CD2/IL23A/HAVCR2/SLAMF6

GOBP_CELLULAR_RESPONSE_TO_ABIOTIC_STIMULUS GOBP_CELLULAR_RESPONSE_TO_ABIOTIC_STIMULUS GOBP_CELLULAR_RESPONSE_TO_ABIOTIC_STIMULUS 317 0.388414313 1.442330772 0.00591716 0.05235695 0.041436047 3824 tags=32%, list=23%, signal=26% MMP1/MMP3/TIMP1/GJA1/IL1B/MMP7/COL1A1/MMP9/CASP1/MMP2/HYAL1/ITGA2/ENG/IFI16/BAG3/BNIP3/TLR8/CARD16/HSPA5/PTAFR/POLD3/PIEZO1/HYAL2/CD40/SERPINB6/H2AX/TNFRSF10A/CDC25A/MAP3K20/CASP5/BLM/EIF2S1/IRF1/LRRC8E/TNFRSF10B/MME/AKR1B1/RIPOR2/ECT2/AURKB/METTL3/PBK/PKD2/ATR/NMT1/TMEM109/LRRC8C/CASP8/TNKS1BP1/CRYAB/NEDD4/ARHGEF2/SFRP1/PARP1/RCSD1/FIGNL1/CNN2/SFRP2/MFAP4/NOC2L/AQP1/CHEK1/RAD1/RUVBL2/ATF4/BMP6/AKT1/NPM1/FBXO4/TANK/DDB2/TSPYL5/COPS9/TRIAP1/CRY1/SLC2A1/PTPN11/YBX3/HRAS/EEF1D/ERRFI1/TMEM161A/HYAL3/DDB1/NOX1/ACTR5/USP47/XRCC5/BAK1/RAD51AP1/GRK1/MAP3K14/SLC38A2/MDM2/ZFP36L1/METAP2/RHBDD1/AQP5/SOX9/FADD/HABP4/SIPA1/GPR68

GOBP_CHROMOSOME_SEGREGATION GOBP_CHROMOSOME_SEGREGATION GOBP_CHROMOSOME_SEGREGATION 317 0.385935905 1.433127494 0.00591716 0.05235695 0.041436047 5068 tags=45%, list=30%, signal=32% CENPW/GEM/FAM83D/FEN1/TRIP13/CCNB1/BIRC5/MAD2L2/TUBG1/TTL/BUB1/CDC20/PRC1/NCAPG/NDC80/NEK6/RRS1/CDCA5/TACC3/PTTG1/KIF14/CDC6/MIS12/MKI67/KNSTRN/TENT4A

/ECT2/NUP62/CENPE/AURKB/BUB1B/NUSAP1/FANCD2/PLK1/UBE2C/RAN/CDCA2/SMC4/DLGAP5/HJURP/KIF23/KNL1/TOP1/CDC27/RCC2/ZWINT/SKA2/NUDC/KPNB1/TRAPPC12/MLH1/KIF2C/CENPN/SKA3/PSRC1/NTMT1/HECW2/CDCA8/KIF4A/CDC26/PINX1/RAD51C/SPAG5/NUP37/NCAPH/NCAPG2/CDT1/FBXW7/SPDL1/NDE1/KLHL22/ACTR3/EML3/UBE2I/KNTC1/ANAPC7/EME1/PLSCR1/FBXO5/SGO2/RACGAP1/SFPQ/SMC2/TUBG2/KLHDC8B/VPS4A/NUF2/BRCA1/CENPX/DSCC1/KIF18A/SPC25/REC8/NAA10/CENPT/KIF18B/ZNF207/DDX11/CENPF/RCC1/BUB3/TTK/NEK11/CHFR/TOP2A/MAD2L1/DYNC1LI1/ARL8A/MRE11/CSNK2A2/BANF1/NEK2/CIAO2B/PHF13/SKA1/NDC1/NCAPD3/CCNE1/CENPK/P3H4/SMC1A/ATM/HNRNPU/MMS19/CHMP7/RGS14/HASPIN/ZW10/MLH3/GEN1/CDK5RAP2/CORT/NAA50/MUS81/SIRT2/MAD1L1/ANAPC5/OIP5/SMARCAD1/TOP3A/BAG6/CDC16/RIOK2

GOBP_ENDODERM_DEVELOPMENT GOBP_ENDODERM_DEVELOPMENT

GOBP_ENDODERM_DEVELOPMENT 74 0.526667735 1.624847768 0.00591716

0.05235695 0.041436047 1602 tags=27%, list=9%, signal=25%

COL12A1/MMP9/COL5A2/MMP2/LAMC1/COL6A1/LAMA3/COL4A2/INHBA/COL5A1/FN1/ITGA5/DUSP4/ITGB2/COL8A1/PELO/DKK1/LAMB1/COL7A1/ITGAV

GOBP_REGULATION_OF_LYMPHOCYTE_MIGRATION

GOBP_REGULATION_OF_LYMPHOCYTE_MIGRATION

GOBP_REGULATION_OF_LYMPHOCYTE_MIGRATION 59 0.539253419 1.61352061

0.00591716 0.05235695 0.041436047 2688 tags=46%, list=16%, signal=39%

CXCL13/CXCL10/WNT5A/MSN/CCR2/IL27RA/ECM1/CCL20/S100A7/MADCAM1/CCL4/CCL2/JAM2/CCR6/LRCH1/RIPOR2/CD200/CXCL14/ADAM8/STK10/AIF1/APOD/SELENOK/CCL7/AKT1/ITGA4/DOCK8

GOBP_REGULATION_OF_MORPHOGENESIS_OF_AN_EPITHELIUM

GOBP_REGULATION_OF_MORPHOGENESIS_OF_AN_EPITHELIUM

GOBP_REGULATION_OF_MORPHOGENESIS_OF_AN_EPITHELIUM 59 0.550170819

1.646186977 0.00591716 0.05235695 0.041436047 2506 tags=27%, list=15%, signal=23%

GREM1/GJA1/CXCL10/AGT/WNT5A/SULF1/SHH/TACSTD2/PIK3CD/LIF/ITGAX/SFRP1/FGF7/WNT4/WNT2/MDK

GOBP_SOMATIC_DIVERSIFICATION_OF_IMMUNOGLOBULINS

GOBP_SOMATIC_DIVERSIFICATION_OF_IMMUNOGLOBULINS

GOBP_SOMATIC_DIVERSIFICATION_OF_IMMUNOGLOBULINS 59 0.535937714

1.603599565 0.00591716 0.05235695 0.041436047 3508 tags=41%, list=21%, signal=32%

TGFB1/IL27RA/PTPRC/TNFSF13/MAD2L2/SAMHD1/CD40/BCL6/CCR6/TCF3/EXOSC3/EXO1/MSH6/MLH1/BATF/NBN/MCM3AP/SWAP70/THOC1/POLB/RNF8/HSPD1/SLC15A4/CTNNBL1

GOBP_IMMUNOGLOBULIN_PRODUCTION_INVOLVED_IN_IMMUNOGLOBULIN_MEDIATED_IMMUNE_RESPONSE

GOBP_IMMUNOGLOBULIN_PRODUCTION_INVOLVED_IN_IMMUNOGLOBULIN_MEDIATED_IMMUNE_RESPONSE

GOBP_IMMUNOGLOBULIN_PRODUCTION_INVOLVED_IN_IMMUNOGLOBULIN_MEDIATED_IMMUNE_RESPONSE 52 0.55522398 1.638578255 0.005943536 0.052483471

0.041536178 3272 tags=40%, list=19%, signal=33%

TGFB1/HLA-DQB1/IL27RA/PTPRC/TNFSF13/MAD2L2/CD40/BCL6/CCR6/EXOSC3/BTK/EXO1/

MSH6/MLH1/BATF/NBN/SWAP70/THOC1/RNF8/HSPD1/SLC15A4
GOBP_INTERLEUKIN_10_PRODUCTION GOBP_INTERLEUKIN_10_PRODUCTION GOBP_INTERLEUKIN_10_PRODUCTION 55 0.550930783 1.638816195 0.005952381 0.052483471 0.041536178 2623 tags=36%, list=15%, signal=31% CD274/IDO1/TRIB2/JAK3/IRF4/CLEC7A/FCGR2B/SASH3/TLR2/TYROBP/STAT3/CD47/IL23A/IL6/TSLP/LILRB4/LILRB1/NOD2/SYK/DLL1
GOBP_REACTIVE_OXYGEN_SPECIES_METABOLIC_PROCESS GOBP_REACTIVE_OXYGEN_SPECIES_METABOLIC_PROCESS GOBP_REACTIVE_OXYGEN_SPECIES_METABOLIC_PROCESS 254 0.408655113 1.489217396 0.006082725 0.053509986 0.042348577 1898 tags=22%, list=11%, signal=20% MMP3/DUOX2/DUOXA2/IL1B/AGT/NCF2/CAV1/PXDN/NOS2/ALOX5/CCN1/ASS1/IFI6/TGFB1/HBB/TIGAR/PRDX4/RAC2/CLU/BNIP3/HIF1A/PTGS2/NQO2/CLEC7A/TGFBR2/FYN/ITGB2/JAK2/SH3PXD2B/DDIT4/TLR2/GNAI2/ICAM1/PDGFRB/SOD3/TYROBP/FOXM1/SIRPA/ITGAM/KLF2/FBLN5/XDH/VAV1/PKD2/HK2/RORA/NRROS/CFLAR/CD47/CRYAB/GLA/PTGIS/NCF4/ATP2B4/IFNG/FPR2
GOBP_I_KAPPAB_KINASE_NF_KAPPAB_SIGNALING GOBP_I_KAPPAB_KINASE_NF_KAPPAB_SIGNALING GOBP_I_KAPPAB_KINASE_NF_KAPPAB_SIGNALING 261 0.413188092 1.506402134 0.006090134 0.053509986 0.042348577 3811 tags=39%, list=23%, signal=30% TNIP3/GJA1/IL1B/S100A12/WNT5A/TGM2/CASP1/PIM2/TRIM22/UNC5CL/CD74/PRDX4/LY96/BIRC3/LGALS1/ECM1/F2R/RIPK2/APOL3/TLR8/CARD16/TIFA/CCR7/BST2/FYN/DDX21/STAT1/TRIM27/TLR2/IRAK1/S100A4/CD40/RHOH/SIRPA/FLNA/LTF/MTDH/NEK6/GPRC5B/CASP10/TNFRSF10B/TRAF5/ECT2/NUP62/CCL19/PRKCB/S100A13/IRAK2/RORA/BTK/CFLAR/S100B/AJUBA/CASP8/LILRB4/LURAP1L/RELA/PLK2/TNFSF11/NOD2/TRAF1/GOLT1B/SHISA5/REL/MALT1/HACD3/CD4/TRIM59/TRAF3/AKT1/TANK/LIME1/UBE2N/TLE1/IRF3/TRIM5/TRIM21/ROCK1/TBK1/ATP2C1/TRIM8/GSTP1/EEF1D/RHOC/PRKD1/ERC1/CARD11/CX3CL1/MAPKBP1/NKIRAS1/MAP3K14/DEFB124/MID2/FASLG/DDRGK1/TERF2IP/FADD/TFG/TIFAB/IKBKG/CCDC22
GOBP_DETECTION_OF_LIGHT_STIMULUS GOBP_DETECTION_OF_LIGHT_STIMULUS GOBP_DETECTION_OF_LIGHT_STIMULUS 72 -0.446870166 -1.545351205 0.006116208 0.053643411 0.042454171 742 tags=8%, list=4%, signal=8% CACNA2D4/GNA11/CNGA1/NMT2/OPN3/PDE6A
GOBP_NEGATIVE_REGULATION_OF_ENDOTHELIAL_CELL_PROLIFERATION GOBP_NEGATIVE_REGULATION_OF_ENDOTHELIAL_CELL_PROLIFERATION GOBP_NEGATIVE_REGULATION_OF_ENDOTHELIAL_CELL_PROLIFERATION 39 0.584931874 1.644523219 0.006153846 0.053643411 0.042454171 1072 tags=28%, list=6%, signal=26% GJA1/SPARC/RGCC/CAV1/ALOX5/SULF1/PTPRM/STAT1/CAV2/CCL2/XDH
GOBP_COLLAGEN_BIOSYNTHETIC_PROCESS GOBP_COLLAGEN_BIOSYNTHETIC_PROCESS GOBP_COLLAGEN_BIOSYNTHETIC_PROCESS 43 0.581700406 1.665379451 0.006163328 0.053643411 0.042454171 2826 tags=47%, list=17%, signal=39% COL1A1/RGCC/ITGA2/ENG/SERPINB7/TGFB1/COL5A1/F2R/VIM/PDGFRB/TRAM2/CREB3L1/EMILIN1/SERPINH1/IL6/WNT4/SERPINF2/TGFB3/ARRB2/SUCO
GOBP_MYOBLAST_FUSION GOBP_MYOBLAST_FUSION GOBP_MYOBLAST_FUSION 40 0.585695625 1.653248547 0.00617284 0.053643411 0.042454171 1720 tags=25%, list=10%, signal=23% CXCL9/CXCL10/CD53/CD81/JAM2/PLEKHO1/RIPOR2/MYOF/CFLAR/EHD2

GOBP_NUCLEOSIDE_MONOPHOSPHATE_BIOSYNTHETIC_PROCESS
GOBP_NUCLEOSIDE_MONOPHOSPHATE_BIOSYNTHETIC_PROCESS
GOBP_NUCLEOSIDE_MONOPHOSPHATE_BIOSYNTHETIC_PROCESS 40 0.584871679
1.650922786 0.00617284 0.053643411 0.042454171 3655 tags=55%, list=22%, signal=43%
ADA/PPAT/GART/AMPD3/PRPS1/UPP1/AMPD2/TK1/DCTD/TYMS/UMPS/IMPDH1/GMPS/CAD/PAICS/HPRT1/PFAS/APRT/ADSL/TK2/ATIC/NUDT2
GOBP_EXPORT_ACROSS_PLASMA_MEMBRANE GOBP_EXPORT_ACROSS_PLASMA_MEMBRANE
GOBP_EXPORT_ACROSS_PLASMA_MEMBRANE 56 -0.528484061 -1.735320139
0.00619195 0.053643411 0.042454171 952 tags=16%, list=6%, signal=15%
ATP1B3/ATP2B1/KCNK5/PDZK1/SLC4A4/SLC22A5/SLC35G1/ABCB1/ABCG2
GOBP_ORGANIC_HYDROXY_COMPOUND_CATABOLIC_PROCESS
GOBP_ORGANIC_HYDROXY_COMPOUND_CATABOLIC_PROCESS
GOBP_ORGANIC_HYDROXY_COMPOUND_CATABOLIC_PROCESS 71 -0.460514248
-1.586031851 0.00619195 0.053643411 0.042454171 2447 tags=37%, list=14%, signal=31%
FAH/INPP5A/SYNJ1/GK5/ALDH1B1/ADH4/CYP4F3/SCARB1/INPP5K/MT3/DIO3/AKR1C3/IMPA2/INPP5J/IMPA1/OCRL/AKR1B10/CYP4F12/LDHD/SULT1A1/CYP4F2/SULT1A2/SULT1B1/BPNT1/MAOA/CYP27A1
GOBP_ESTABLISHMENT_OF_ENDOTHELIAL_BARRIER
GOBP_ESTABLISHMENT_OF_ENDOTHELIAL_BARRIER
GOBP_ESTABLISHMENT_OF_ENDOTHELIAL_BARRIER 48 0.554258205 1.608696223
0.00620155 0.053643411 0.042454171 2098 tags=31%, list=12%, signal=27%
IL1B/PECAM1/CLDN1/MSN/ENG/CDH5/S1PR3/ICAM1/FASN/ROBO4/PLCB1/PPP1R16B/VCL/RDX/PTPRS
GOBP_NCRNA_EXPORT_FROM_NUCLEUS GOBP_NCRNA_EXPORT_FROM_NUCLEUS
GOBP_NCRNA_EXPORT_FROM_NUCLEUS 37 0.594693148 1.651810345 0.00620155
0.053643411 0.042454171 4612 tags=68%, list=27%, signal=49%
SEC13/XPOT/NUP62/RAN/NUP98/NUP85/NUP93/NUP210/RAE1/NUP37/NPM1/NUP58/NUP205/AAAS/NUP50/NUP35/NUP155/NUP107/RANBP2/NOL6/NDC1/NUP188/NUP88/NUP54/NUP214
GOBP_BONE_CELL_DEVELOPMENT GOBP_BONE_CELL_DEVELOPMENT
GOBP_BONE_CELL_DEVELOPMENT 32 0.601565961 1.643294192 0.00625
0.053882794 0.042643622 3254 tags=47%, list=19%, signal=38%
FBN1/KIT/TYROBP/FLI1/LTF/MEIS1/CLDN18/PIP4K2A/SLC9B2/ANXA2/LILRB1/TNFSF11/FBXW7/PTPN11/WASF2
GOBP_TRNA_TRANSPORT GOBP_TRNA_TRANSPORT GOBP_TRNA_TRANSPORT 35
0.601845142 1.655398079 0.006259781 0.053882794 0.042643622 5156
tags=77%, list=30%, signal=54%
SEC13/XPOT/NUP62/RAN/NUP98/NUP85/NUP93/NUP210/RAE1/NUP37/NUP58/NUP205/AAAS/NUP50/NUP35/NUP155/NUP107/RANBP2/NOL6/NDC1/NUP188/NUP88/NUP54/NUP214/SSB/YBX1/TPR
GOBP_BASEMENT_MEMBRANE_ORGANIZATION
GOBP_BASEMENT_MEMBRANE_ORGANIZATION

GOBP_BASEMENT_MEMBRANE_ORGANIZATION 28 0.619557922 1.643767109 0.006279435 0.053882794 0.042643622 2465 tags=43%, list=15%, signal=37% COL4A1/NID1/CAV1/PXDN/CAV2/LAMB1/LAMB2/NID2/PLOD3/PHLDB2/RAMP2/FERMT1
GOBP_CHAPERONE_COFACTOR_DEPENDENT_PROTEIN_REFOLDING GOBP_CHAPERONE_COFACTOR_DEPENDENT_PROTEIN_REFOLDING GOBP_CHAPERONE_COFACTOR_DEPENDENT_PROTEIN_REFOLDING 28 0.619135479 1.642646314 0.006279435 0.053882794 0.042643622 1107 tags=25%, list=7%, signal=23% SDF2L1/CD74/HSPH1/HSPA5/HSPA6/HSPA13/ERO1A
GOBP_STEROID_METABOLIC_PROCESS GOBP_STEROID_METABOLIC_PROCESS GOBP_STEROID_METABOLIC_PROCESS 299 -0.355932309 -1.486105885 0.006289308 0.053882794 0.042643622 1955 tags=22%, list=12%, signal=20% PLEKHA1/SCARB1/OSBPL7/FDX1/SREBF2/SCP2/APOBR/HINT2/PBX1/LGMN/MT3/SCAP/CEBPA/RXRA/PLPP6/PRKAG2/LIMA1/YWHAH/AKR1C2/CYP1A1/SIRT1/PRKAA1/ERLIN2/ARV1/AKR1C3/SOAT1/HSD3B1/DGKQ/CFTR/SRD5A1/CAT/ABCG1/PPARD/ACAA1/ACOT8/ACOX2/VDR/BMP2/ATP8B1/HSD11B2/ACAA2/NR1I2/SULT1A1/NR1H4/UGT2B28/HSD17B11/SULT1A2/APP/CLCN2/LPCAT3/SULT1B1/NR5A2/PRLR/CYP27A1/ACAT1/ABCB11/EPHX2/OSBPL1A/FMO5/PPARGC1A/CYP2B6/HSD3B2/HSD17B2/DHRS11/VLDLR/HMGCS2
GOBP_DETECTION_OF_MECHANICAL_STIMULUS_INVOLVED_IN_SENSORY_PERCEPTION GOBP_DETECTION_OF_MECHANICAL_STIMULUS_INVOLVED_IN_SENSORY_PERCEPTION GOBP_DETECTION_OF_MECHANICAL_STIMULUS_INVOLVED_IN_SENSORY_PERCEPTION 31 0.605923592 1.639238666 0.006299213 0.053882794 0.042643622 657 tags=19%, list=4%, signal=19% SERPINE2/ITGA2/TRPA1/KIT/FYN/CXCR4
GOBP_GLYCEROPHOSPHOLIPID_METABOLIC_PROCESS GOBP_GLYCEROPHOSPHOLIPID_METABOLIC_PROCESS GOBP_GLYCEROPHOSPHOLIPID_METABOLIC_PROCESS325 -0.308343426 -1.301289357 0.006329114 0.053882794 0.042643622 2107 tags=26%, list=12%, signal=23% SLC44A4/FAR1/ITPKC/PIGN/SH3YL1/SERINC3/INPP5A/SACM1L/SYNJ1/AGPAT2/PNPLA7/BMX/MPPE1/PLEKHA3/CRLS1/PLEKHA1/SCARB1/PIGL/GPD1/PCTP/CDS1/INPP5K/ABHD4/PLA2G4C/SLC44A3/PAFAH1B1/SLC44A5/CAPN2/SLC44A1/SERINC2/ARF3/SOCS2/SERINC1/PLAAT4/PIK3C2B/PLA2G12A/ABHD5/HADHB/DDHD2/LIPH/CDIPT/PIGV/NR1H3/LPIN2/CHPT1/FAM126B/IMPA2/INPP5J/INPP4B/DGKQ/IMPA1/CHKA/ABHD12/OCRL/SOCS6/PGAP3/PLD1/GDE1/HADHA/SERINC5/ALPI/PNLIPRP2/GPD1L/ABHD3/PGAP1/LIPC/MTMR11/NR1H4/PLCD1/NAPEPLD/PIP5K1B/ITPKA/LPCAT3/IP6K2/PRDX6/BPNT1/GDPD1/MTM1/ETNK1/PLAAT2/PIGZ/NAAA/CWH43
GOBP_ANGIOGENESIS_INVOLVED_IN_WOUND_HEALING GOBP_ANGIOGENESIS_INVOLVED_IN_WOUND_HEALING GOBP_ANGIOGENESIS_INVOLVED_IN_WOUND_HEALING 25 0.645156215 1.673857998 0.006369427 0.053882794 0.042643622 3086 tags=52%, list=18%, signal=43% MCAM/ALOX5/SERPINE1/XBP1/SMOC2/CXCR4/KDR/HPSE/GPR4/GPX1/ETS1/CD34/PRCP
GOBP_LIPID_CATABOLIC_PROCESSGOBP_LIPID_CATABOLIC_PROCESS GOBP_LIPID_CATABOLIC_PROCESS315 -0.471090974 -1.991713602 0.006369427 0.053882794 0.042643622 2058 tags=32%, list=12%, signal=28% ADTRP/IRS2/ACAD10/PLCB4/PNPLA4/PECR/CYP4F3/SCARB1/RAB7A/CRAT/FAAH/SCP2/HINT2/FABP2/ABHD4/PLA2G4C/PAFAH1B1/NEU4/DECR1/THRA/MT3/ECHDC2/PLAAT4/PDE3B/PLA2G12A/PLCD3/YWHAH/ABHD5/ETFB/PEX13/HADHB/DDHD2/LIPH/PRKAA1/ACBD5/LPIN2/ECI1/PPA

RA/FMC1/PLCXD2/AKR1C3/PCCA/ETFA/PCK2/PAFAH2/SRD5A1/ECH1/ABHD12/FUCA1/AKR1B10/PPARD/ACAA1/ACOT8/ACOX2/ABCD3/PLCH1/PLD1/BDH2/HADH/GDE1/HADHA/ACAA2/PNLIPRP2/ABHD3/CES3/CYP4F12/CPT2/LIPC/FABP1/MGLL/PLCD1/NEU1/CROT/NAPEPLD/HSD17B11/CYP4F2/CES2/CPT1A/ACADS/AUH/SORL1/PLCE1/EHHADH/PRDX6/BCO1/GDPD1/PHYH/NCEH1/NUDT7/ACADM/CYP27A1/ACAT1/ETFDH/ABCB11/PLAAT2/ACOX1/GBA3/PLA2G12B/NAAA/PCK1

GOBP_HYALURONAN_CATABOLIC_PROCESS GOBP_HYALURONAN_CATABOLIC_PROCESS GOBP_HYALURONAN_CATABOLIC_PROCESS 18 0.689170781 1.671571841 0.006379585 0.053882794 0.042643622 780 tags=28%, list=5%, signal=27% CEMIP/HYAL1/CD44/TGFB1/HYAL2

GOBP_INFLAMMATORY_CELL_APOPTOTIC_PROCESS GOBP_INFLAMMATORY_CELL_APOPTOTIC_PROCESS GOBP_INFLAMMATORY_CELL_APOPTOTIC_PROCESS 18 0.68340553 1.657588326 0.006379585 0.053882794 0.042643622 3204 tags=61%, list=19%, signal=50% ANXA1/SLC7A11/PIK3CD/PLEKHO2/ITPKB/IL6/CTSL/NOD2/IRF7/IRF3/SELENOS

GOBP_POSITIVE_REGULATION_OF_INTERLEUKIN_17_PRODUCTION GOBP_POSITIVE_REGULATION_OF_INTERLEUKIN_17_PRODUCTION GOBP_POSITIVE_REGULATION_OF_INTERLEUKIN_17_PRODUCTION 18 0.685321149 1.662234625 0.006379585 0.053882794 0.042643622 2677 tags=50%, list=16%, signal=42% SLC7A5/TGFB1/SPHK1/IL23A/IL6/SLAMF6/NOD2/LY9/IL18

GOBP_RESPONSE_TO_PROTOZOAN GOBP_RESPONSE_TO_PROTOZOAN GOBP_RESPONSE_TO_PROTOZOAN 20 0.700449451 1.741200884 0.006379585 0.053882794 0.042643622 3030 tags=50%, list=18%, signal=41% PF4/IRF4/CLEC7A/CD40/GBP2/LYST/BATF2/BATF/GBP4/HRAS

GOBP_STORE_OPERATED_CALCIUM_ENTRY GOBP_STORE_OPERATED_CALCIUM_ENTRY GOBP_STORE_OPERATED_CALCIUM_ENTRY 18 0.683975084 1.658969768 0.006379585 0.053882794 0.042643622 2456 tags=44%, list=15%, signal=38% MS4A1/HOMER1/GRAMD2A/CD84/SPINK1/ORAI2/ORAI1/STIM2

GOBP_THYROID_HORMONE_METABOLIC_PROCESS GOBP_THYROID_HORMONE_METABOLIC_PROCESS GOBP_THYROID_HORMONE_METABOLIC_PROCESS 26 0.639247132 1.668418673 0.006420546 0.05413761 0.042845287 107 tags=12%, list=1%, signal=11% DUOX2/DUOXA2/CTSK

GOBP_POSITIVE_REGULATION_OF_I_KAPPAB_KINASE_NF_KAPPAB_SIGNALING GOBP_POSITIVE_REGULATION_OF_I_KAPPAB_KINASE_NF_KAPPAB_SIGNALING GOBP_POSITIVE_REGULATION_OF_I_KAPPAB_KINASE_NF_KAPPAB_SIGNALING 172 0.435147687 1.518420746 0.006485084 0.054569384 0.043186999 2378 tags=30%, list=14%, signal=26% GJA1/S100A12/TGM2/CASP1/PIM2/TRIM22/UNC5CL/CD74/BIRC3/LGALS1/ECM1/F2R/RIPK2/APOL3/CARD16/TIFA/CCR7/BST2/FYN/DDX21/TRIM27/IRAK1/S100A4/CD40/FLNA/LTF/MTDH/NEK6/GPRC5B/CASP10/TNFRSF10B/TRAF5/ECT2/NUP62/CCL19/PRKCB/S100A13/CFLAR/S100B/AJUBA/CASP8/LURAP1L/RELA/PLK2/TNFSF11/NOD2/GOLT1B/SHISA5/REL/MALT1/CD4

GOBP_FATTY_ACID_METABOLIC_PROCESS GOBP_FATTY_ACID_METABOLIC_PROCESS GOBP_FATTY_ACID_METABOLIC_PROCESS 362 -0.418628542 -1.791233442 0.006493506 0.054569384 0.043186999 2232 tags=30%, list=13%, signal=26%

ECHS1/ETFBKMT/AKR1C1/GSTA1/ACSS1/ADTRP/IRS2/ACAD10/PRKAB1/HACD2/PECR/ADH4/CYP4F3/CRAT/GSTM1/FAAH/CBR4/SCP2/GPX4/PLA2G4C/DLD/ACOT11/DECR1/ECHDC2/GSTM2/SCAP/PRKAG2/CYP2J2/HPGDS/AKR1C2/ABHD5/ETFB/CYP1A1/PEX13/HADHB/SIRT1/LIPH/PRKAA1/ACBD5/ERLIN2/NR1H3/LPIN2/ECI1/PRKAB2/PPARA/EDN2/PTGR1/AKR1C3/PCCA/ETFA/PCK2/PER2/PDK3/ACSL5/ECH1/PPARD/PTGR2/ACAA1/ALKBH7/ACOT8/ACOX2/ABCD3/ACSS2/ZADH2/BDH2/EPHX1/ACOT4/HADH/CRYL1/HADHA/ACSM3/ACAA2/ABHD3/CYP4F12/CPT2/ACADSB/LIPC/FABP1/MGLL/CROT/CYP4F2/GSTM4/LIAS/CES2/CPT1A/ACADS/AUH/EHHADH/PHYH/NUDT7/ACADM/ACAT1/ETFDH/ABCB11/PDK2/EPHX2/PDK4/PPARG/CYP2S1/ACOX1/PPARGC1A/HPGD/CYP2B6/APPL2/ACSF2/EDN1/NAAA/PCK1

GOBP_ORGANIC_ACID_TRANSPORT GOBP_ORGANIC_ACID_TRANSPORT GOBP_ORGANIC_ACID_TRANSPORT 308 -0.365397561 -1.532938331 0.006535948 0.054737279 0.043319873 1476 tags=16%, list=9%, signal=15% RXRA/PLA2G12A/PLA2R1/PRKAG2/MAP2K6/PRKAA1/GLS/PRKAB2/PPARA/BDKRB2/PER2/ACSL5/PPARD/LLGL2/PLIN2/ABCD3/NTRK2/ATP8B1/NCOA2/CPT2/FABP1/NR1H4/CROT/CYP4F2/XK/SLC25A20/SLC46A1/CPT1A/SLC9A3R1/P2RX4/SFXN1/SLC26A3/SLC22A4/ABCB11/SLC6A19/SLC23A1/PPARG/SLC36A1/SLC51B/THBS1/SLC1A1/PLA2G12B/EDN1/SLC16A1/SLC3A1/ABCB1/SLC38A4/SLC26A2/SLC51A/ABCG2

GOBP_HYALURONAN_BIOSYNTHETIC_PROCESS GOBP_HYALURONAN_BIOSYNTHETIC_PROCESS GOBP_HYALURONAN_BIOSYNTHETIC_PROCESS 14 0.7263683 1.662254479 0.006578947 0.054737279 0.043319873 333 tags=29%, list=2%, signal=28% CEMIP/IL1B/HYAL1/TGFB1

GOBP_INFLAMMATORY_RESPONSE_TO_WOUNDING GOBP_INFLAMMATORY_RESPONSE_TO_WOUNDING GOBP_INFLAMMATORY_RESPONSE_TO_WOUNDING 14 0.731115481 1.673118145 0.006578947 0.054737279 0.043319873 941 tags=50%, list=6%, signal=47% TIMP1/ALOX5/CCR2/TGFB1/F2R/HIF1A/IL1A

GOBP_MAINTENANCE_OF_PROTEIN_LOCALIZATION_IN_ENDOPLASMIC_RETICULUM GOBP_MAINTENANCE_OF_PROTEIN_LOCALIZATION_IN_ENDOPLASMIC_RETICULUM GOBP_MAINTENANCE_OF_PROTEIN_LOCALIZATION_IN_ENDOPLASMIC_RETICULUM 14 0.725011091 1.659148578 0.006578947 0.054737279 0.043319873 2793 tags=43%, list=16%, signal=36% GJA1/KDELR3/HSPA5/INSIG1/KDELR2/KDELR1

GOBP_POSITIVE_REGULATION_OF_T_CELL_RECEPTOR_SIGNALING_PATHWAY GOBP_POSITIVE_REGULATION_OF_T_CELL_RECEPTOR_SIGNALING_PATHWAY GOBP_POSITIVE_REGULATION_OF_T_CELL_RECEPTOR_SIGNALING_PATHWAY 14 0.733436074 1.678428697 0.006578947 0.054737279 0.043319873 3615 tags=71%, list=21%, signal=56% ADA/KCNN4/CCR7/CD81/RAB29/LCK/RELA/CARD11/PRKD2/NECTIN2

GOBP_SEQUESTERING_OF_METAL_ION GOBP_SEQUESTERING_OF_METAL_ION GOBP_SEQUESTERING_OF_METAL_ION 14 0.72733989 1.664477909 0.006578947 0.054737279 0.043319873 486 tags=29%, list=3%, signal=28% S100A8/LCN2/S100A9/S100A7

GOBP_AROMATIC_AMINO_ACID_TRANSPORT GOBP_AROMATIC_AMINO_ACID_TRANSPORT GOBP_AROMATIC_AMINO_ACID_TRANSPORT 13 0.724618366 1.61684465 0.006666667 0.055192519 0.043680157 3272 tags=54%, list=19%, signal=43%

SLC7A5/SLC7A1/SLC38A5/SLC36A4/SLC25A29/SLC7A8/SLC15A4
GOBP_MEMBRANE_REPOLARIZATION_DURING_VENTRICULAR_CARDIAC_MUSCLE_CELL_ACTION_POTENTIAL
GOBP_MEMBRANE_REPOLARIZATION_DURING_VENTRICULAR_CARDIAC_MUSCLE_CELL_ACTION_POTENTIAL
GOBP_MEMBRANE_REPOLARIZATION_DURING_VENTRICULAR_CARDIAC_MUSCLE_CELL_ACTION_POTENTIAL 13 0.725083602 1.617882734 0.006666667 0.055192519 0.043680157 1343 tags=23%, list=8%, signal=21% KCND3/KCNE3/KCNJ8
GOBP_REGULATION_OF_ACUTE_INFLAMMATORY_RESPONSE_TO_ANTIGENIC_STIMULUS
GOBP_REGULATION_OF_ACUTE_INFLAMMATORY_RESPONSE_TO_ANTIGENIC_STIMULUS
GOBP_REGULATION_OF_ACUTE_INFLAMMATORY_RESPONSE_TO_ANTIGENIC_STIMULUS 13 0.725279877 1.618320683 0.006666667 0.055192519 0.043680157 3688 tags=62%, list=22%, signal=48% C3/CCR7/FCGR2B/BTK/PARK7/SELENOS/PLA2G2D/HLA-E
GOBP_CELL_RECOGNITION GOBP_CELL_RECOGNITION GOBP_CELL_RECOGNITION 145 0.437794798 1.498418454 0.006711409 0.055380166 0.043828664 2715 tags=23%, list=16%, signal=19% C4BPB/C4BPA/PECAM1/CADM1/ROBO1/CATSPERB/MSN/VCAN/EMB/CLEC7A/FCN1/CCR7/CXCR4/CD81/FCN3/PALLD/ROBO4/DOCK2/CCL19/MFGE8/NEXN/CCT3/PEAR1/CCT8/HAVCR2/JMJD6/CCT5/CCT2/CCT4/CCT7/CD6/DOCK8/NRCAM
GOBP_REGULATION_OF_EXTRINSIC_APOPTOTIC_SIGNALING_PATHWAY
GOBP_REGULATION_OF_EXTRINSIC_APOPTOTIC_SIGNALING_PATHWAY
GOBP_REGULATION_OF_EXTRINSIC_APOPTOTIC_SIGNALING_PATHWAY 145 0.452285203 1.54801404 0.006711409 0.055380166 0.043828664 2630 tags=32%, list=16%, signal=27%
IL1B/AGT/CAV1/PEA15/SERPINE1/IFI6/PF4/INHBA/PTPRC/FYN/TNFRSF12A/ICAM1/G0S2/HYAL2/SKIL/TNFRSF10A/SNAI2/IL1A/TNFRSF10B/TMC8/FGFR1/EYA3/BID/ITGA6/PDIA3/CFLAR/ACVR1/ITGAV/ITPRIP/PML/CASP8/STK3/ARHGEF2/SFRP1/BCL2L12/SRPX/RELA/TGFBR1/TRAF1/SFRP2/GPX1/TIMP3/PSME3/CYLD/AKT1/PARK7
GOBP_CONNECTIVE_TISSUE_REPLACEMENTGOBP_CONNECTIVE_TISSUE_REPLACEMENT
GOBP_CONNECTIVE_TISSUE_REPLACEMENT10 0.789381221 1.6798885 0.006779661 0.05566083 0.044050785 941 tags=50%, list=6%, signal=47% TIMP1/TGFB1/F2R/HIF1A/IL1A
GOBP_ALCOHOL_METABOLIC_PROCESS GOBP_ALCOHOL_METABOLIC_PROCESS
GOBP_ALCOHOL_METABOLIC_PROCESS 350 -0.388864381 -1.646527837 0.006802721 0.05566083 0.044050785 1979 tags=24%, list=12%, signal=21% DHRS3/PLCB4/PNPLA4/PECR/ADH4/SCARB1/FDX1/DEGS2/GPD1/SREBF2/CBR4/APOBR/INPP5K/MT3/SCAP/CEBPA/RXRA/PLPP6/LIMA1/PLCD3/ALDH1A1/SPR/AKR1C2/CYP1A1/PRKAA1/ERLIN2/ARV1/SPHK2/AKR1C3/PCK2/SOAT1/IMPA2/INPP5J/SGPP2/INPP4B/DGKQ/PPIP5K2/CFTR/SPTLC3/IMPA1/CAT/MINPP1/CHKA/OCRL/AKR1B10/ABCG1/PPARD/PLCH1/ACSS2/PCBD2/ADH1A/BMP2/GDE1/ACAA2/SULT1A1/NR1H4/PLCD1/NAPEPLD/MOGAT2/ADH6/SULT1A2/APP/ITPKA/CLCN2/LPCAT3/IP6K2/PLCE1/SULT1B1/BCO1/BPNT1/GDPD1/RETSAT/CYP27A1/PLPP1/EPHX2/P2RY1/OSBPL1A/FMO5/NAAA/VLDLR/ADH1C/HMGCS2/PCK1
GOBP_GUANOSINE_CONTAINING_COMPOUND_BIOSYNTHETIC_PROCESS
GOBP_GUANOSINE_CONTAINING_COMPOUND_BIOSYNTHETIC_PROCESS

GOBP_GUANOSINE_CONTAINING_COMPOUND_BIOSYNTHETIC_PROCESS 11 0.77741286 1.669786358 0.006802721 0.05566083 0.044050785 2694 tags=64%, list=16%, signal=54% NME1/NME5/NME4/IMPDH1/GMPS/NME7/HPRT1

GOBP_ORGANIC_ANION_TRANSPORT GOBP_ORGANIC_ANION_TRANSPORT GOBP_ORGANIC_ANION_TRANSPORT 350 -0.315971587 -1.33788549 0.006802721 0.05566083 0.044050785 1476 tags=16%, list=9%, signal=15% PPFIA3/GLTP/SLC25A12/SLC25A5/APBA1/SLC25A25/ABCC3/CA7/RXRA/PLA2G12A/PLA2R1/MAP2K6/SLC52A3/PRKAA1/ABCC6/GLS/BDKRB2/PER2/SLC35A3/SLC35D2/SLC25A24/CFTR/SLC16A5/LLGL2/NTRK2/ATP8B1/SLC25A23/NCOA2/NR1H4/CA12/CYP4F2/SLCO4C1/SLC46A1/SLC9A3R1/CA2/P2RX4/SFXN1/SLC26A3/ABCB11/SLC23A1/SLC36A1/SLC4A4/SLC51B/SLC1A1/PLA2G12B/CA4/EDN1/SLC16A9/SLC17A4/SLC16A1/CA1/SLC3A1/SLC26A2/SLC51A/ABCG2

GOBP_POSITIVE_REGULATION_OF_TOLERANCE_INDUCTION GOBP_POSITIVE_REGULATION_OF_TOLERANCE_INDUCTION GOBP_POSITIVE_REGULATION_OF_TOLERANCE_INDUCTION 11 0.766593415 1.646547532 0.006802721 0.05566083 0.044050785 1769 tags=45%, list=10%, signal=41% CD274/IDO1/TGFBR2/LILRB2/LILRB4

GOBP_REGULATION_OF_REPRODUCTIVE_PROCESS GOBP_REGULATION_OF_REPRODUCTIVE_PROCESS GOBP_REGULATION_OF_REPRODUCTIVE_PROCESS 142 0.441842148 1.509310964 0.006811989 0.05566083 0.044050785 1860 tags=23%, list=11%, signal=20% TIMP1/GJA1/WNT5A/CDC25B/ADA/PRDX4/SULF1/INHBA/STXBP1/TRIP13/AGO2/SHH/ARHGDIB/PTAFR/CDC20/SERPINF1/VIP/CDC25A/ZFPM2/CALR/SEMG1/WDR77/CCR6/PLCB1/AURKA/METTL3/SPINK1/PHLDA2/LIF/STK3/SFRP1/INTS13

GOBP_POSITIVE_REGULATION_OF_CYSTEINE_TYPE_ENDOPEPTIDASE_ACTIVITY GOBP_POSITIVE_REGULATION_OF_CYSTEINE_TYPE_ENDOPEPTIDASE_ACTIVITY GOBP_POSITIVE_REGULATION_OF_CYSTEINE_TYPE_ENDOPEPTIDASE_ACTIVITY 141 0.442771653 1.511335042 0.006830601 0.055722156 0.044099319 1843 tags=23%, list=11%, signal=21% S100A8/S100A9/ROBO1/CASP1/CTSH/F3/CCN1/IFI16/AIM2/F2R/RIPK2/BOK/CLEC7A/FYN/JAK2/DLC1/MAP3K5/TNFRSF10A/LAPTM5/ASPH/VCP/XDH/CASP10/ATP2A3/TNFSF15/TNFRSF10B/LCK/BID/DAP/CFLAR/PML/CASP8/NLRP2

GOBP_T_CELL_ACTIVATION_INVOLVED_IN_IMMUNE_RESPONSE GOBP_T_CELL_ACTIVATION_INVOLVED_IN_IMMUNE_RESPONSE GOBP_T_CELL_ACTIVATION_INVOLVED_IN_IMMUNE_RESPONSE 105 0.482911645 1.585425952 0.006849315 0.055784113 0.044148353 2728 tags=36%, list=16%, signal=31% ANXA1/CD74/JAK3/IRF4/HLA-DRA/NFKBIZ/ENTPD7/HLA-DMB/RIPK2/FCER1G/FCGR2B/CD86/ICAM1/CD81/BCL6/APBB1IP/LEF1/STAT3/LCP1/IL12RB1/GPR183/CCL19/IL18R1/RORA/IL23A/IL6/LILRB1/IFNG/HAVCR2/BATF/SLAMF6/HLX/SEMA4A/MALT1/LY9/MDK/IL18/ATP7A

GOBP_DNA_GEOMETRIC_CHANGE GOBP_DNA_GEOMETRIC_CHANGE GOBP_DNA_GEOMETRIC_CHANGE 110 0.482220241 1.587002675 0.006925208 0.056297663 0.044554784 5176 tags=52%, list=31%, signal=36% ANXA1/ASCC3/RTEL1/HMGA1/BLM/MCM6/RUVBL1/TWNK/RFC3/PARP1/CETN2/NBN/CHTF18/SETX/MCM3/MCM5/SSBP1/ERCC6L/RFC4/RUVBL2/MCM7/RECQL/DDB2/CHD7/PIF1/CHD1L/C

HD3/CHD8/G3BP1/RAD54L/DDB1/HMGB1/CHD4/FBH1/XRCC5/MCM2/DSCC1/DDX11/TOP2A/GTF2H3/DHX36/SMARCA1/CHD1/MRE11/CUL4A/RAD54L2/ERCC3/RAD54B/SMARCAL1/GTF2H5/WRN/RFC5/ZRANB3/SMARCAD1/RAD51/DDX1/HNRNPA2B1
GOBP_RECEPTOR_INTERNALIZATION GOBP_RECEPTOR_INTERNALIZATION GOBP_RECEPTOR_INTERNALIZATION 107 0.48390359 1.5848914 0.006934813 0.056297663 0.044554784 1911 tags=21%, list=11%, signal=18% GREM1/CXCL8/RAB31/CXCR2/CAV1/RAMP3/FCER1G/ITGB2/CALCRL/SELE/PLCG2/CD81/PCSK9/MX2/DKK1/WDR54/SDCBP/AHI1/LILRB4/NEDD4/LILRB1/MX1
GOBP_NUCLEOSIDE_BISPHOSPHATE_METABOLIC_PROCESS GOBP_NUCLEOSIDE_BISPHOSPHATE_METABOLIC_PROCESS GOBP_NUCLEOSIDE_BISPHOSPHATE_METABOLIC_PROCESS 125 -0.422055601 -1.604143729 0.007067138 0.05725426 0.045311849 2644 tags=34%, list=16%, signal=29% PDP2/FAR1/ACACB/HSD17B8/PDHA1/FAR2/FITM2/MPC2/HMGCR/ACSS1/SLC35B3/GLYAT/HACD2/CBR4/DLAT/DLD/ACOT11/SUCLA2/HMGCL/PDK3/PAPSS2/ACSL5/ACOT8/ACOT13/ACSS2/ACOT4/ACSM3/SUCLG2/PANK3/SULT1A1/CROT/SULT1A2/SULT1B1/BPNT1/NUDT7/ACAT1/ENPP1/PDK2/PDK4/PANK1/ACSF2/SLC26A2/HMGCS2
GOBP_VIRAL_LIFE_CYCLE GOBP_VIRAL_LIFE_CYCLE GOBP_VIRAL_LIFE_CYCLE 328 0.374391858 1.394711458 0.007075472 0.05725426 0.045311849 3799 tags=35%, list=22%, signal=28% CD55/CXCL8/CLDN1/LAMP3/IFITM2/CAV1/SERPINB3/IFITM3/ISG20/STOM/HYAL1/TRIM22/ITGA2/CD74/IFI16/IFITM1/SLPI/LGALS1/OAS2/ITGA5/FCN1/BST2/CXCR4/CD86/CAV2/TRIM27/NPC1/ICAM1/CCL2/KPNA2/SEC13/HYAL2/CD81/APOBEC3G/FCN3/P4HB/LTF/TRIM15/VCP/RAB29/MRC1/OAS3/CDK1/LY6E/SELPLG/NUP62/TMEM39A/NRP1/RAN/CR2/PPIB/NUP98/ITGAV/LARP1/DDX6/PML/CTSL/AXL/TNFRSF4/NEDD4/NUP85/MX1/KPNB1/CCR5/SHFL/CLEC5A/TMEM39B/FURIN/NUP93/NUP210/HACD3/RAE1/CD4/KPNA6/HSP90AB1/TRIM28/SLC1A5/ADAR/NUP37/NECTIN4/TYRO3/SLC52A2/PVR/CTBP2/TRIM5/TRIM21/EFNB2/APOE/TRIM8/SPCS1/IDE/NUP58/PLSCR1/NUP205/RAD23A/HYAL3/RESF1/VPS4A/DDB1/CR1/TARBP2/AAAS/CD209/FBXL2/NUP50/LDLR/TRIM26/NECTIN2/MID2/PPIH/PARP10/NUP35/KPNA1/NUP155/NUP107
GOBP_LONG_CHAIN_FATTY_ACID_METABOLIC_PROCESS GOBP_LONG_CHAIN_FATTY_ACID_METABOLIC_PROCESS GOBP_LONG_CHAIN_FATTY_ACID_METABOLIC_PROCESS 107 -0.396388989 -1.480209237 0.007117438 0.057501103 0.045507204 2150 tags=28%, list=13%, signal=25% GSTA1/ADTRP/CYP4F3/GSTM1/FAAH/SCP2/GPX4/PLA2G4C/GSTM2/CYP2J2/HPGDS/CYP1A1/AKR1C3/ACSL5/ACAA1/ACOT8/EPHX1/ACOT4/ACSM3/CYP4F12/CPT2/MGLL/CYP4F2/GSTM4/CPT1A/EPHX2/CYP2S1/ACOX1/HPGD/CYP2B6
GOBP_CHEMOKINE_PRODUCTIONGOBP_CHEMOKINE_PRODUCTION GOBP_CHEMOKINE_PRODUCTION89 0.499497801 1.587787879 0.007132668 0.057531501 0.045531261 2123 tags=31%, list=13%, signal=28% S100A8/CXCL6/S100A9/IL1B/WNT5A/IL33/CD74/LPL/RIPK2/HIF1A/CLEC7A/TWIST1/ACKR1/POSTN/TLR2/FFAR2/SIRPA/SNAI2/CSF1R/IL6/TSLP/LILRB4/IFNG/AIF1/APOD/HAVCR2/SELENOK/NOD2
GOBP_CELLULAR_HORMONE_METABOLIC_PROCESS

GOBP_CELLULAR_HORMONE_METABOLIC_PROCESS
GOBP_CELLULAR_HORMONE_METABOLIC_PROCESS 122 -0.388375212 -1.467313018
0.007194245 0.057900665 0.045823423 2279 tags=25%, list=13%, signal=22%
TIPARP/TTR/AKR1C1/DHRS3/PNPLA4/PLEKHA1/ADH4/SCARB1/FDX1/ALDH1A1/AKR1C2/CYP1A1/AKR1C3/HSD3B1/DGKQ/SRD5A1/AKR1B10/ADH1A/BMP2/SULT1A1/ADH6/HSD17B11/CLCN2/BCO1/RETSAT/CYP2S1/PPARGC1A/HSD3B2/HSD17B2/DHRS11/ADH1C
GOBP_GLIOGENESIS GOBP_GLIOGENESIS GOBP_GLIOGENESIS 269 0.396614835
1.455241949 0.007202881 0.057900665 0.045823423 3620 tags=30%, list=21%, signal=24%
S100A8/S100A9/ANXA1/ADGRG6/IL1B/BACE2/IL33/SERPINE2/LYN/LRP8/CCR2/TGFB1/CLU/BNIP3/BOK/C5AR1/VIM/SHH/CXCR4/TLR2/CCL2/IDH2/LEF1/C1QA/STAT3/TSPAN2/IL6ST/EZH2/CDK1/CSF1R/GPR183/LAMB1/METTL3/DUSP10/TNFRSF1B/LAMB2/NRROS/PRKCH/MXRA8/PHGDH/LIF/CDKN2C/IL6/EIF2B3/MAP2K1/IFNG/PRMT5/FPR2/CREB1/DICER1/ITGB4/RELA/NAB1/EIF2B2/B4GALT6/ETV5/MDK/NAB2/AKT1/DLL1/SLC45A3/GPC1/EIF2B5/ARHGEF10/PTPN11/EIF2B4/FA2H/GSTP1/NFIB/NF2/BIN1/HDAC2/CX3CL1/SRGAP2C/EPHA4/CNTNAP1/LDLR/KCNJ10/MMP14/RHEB
GOBP_ACTIVATION_OF_CYSTEINE_TYPE_ENDOPEPTIDASE_ACTIVITY_INVOLVED_IN_APOPTOTIC_PROCESS
GOBP_ACTIVATION_OF_CYSTEINE_TYPE_ENDOPEPTIDASE_ACTIVITY_INVOLVED_IN_APOPTOTIC_PROCESS
GOBP_ACTIVATION_OF_CYSTEINE_TYPE_ENDOPEPTIDASE_ACTIVITY_INVOLVED_IN_APOPTOTIC_PROCESS 82 0.505323353 1.584016195 0.007246377 0.057900665 0.045823423
1714 tags=28%, list=10%, signal=25%
S100A8/S100A9/ROBO1/CTSH/F3/F2R/BOK/JAK2/DLC1/TNFRSF10A/LAPTM5/VCP/XDH/CASP10/ATP2A3/TNFSF15/TNFRSF10B/LCK/BID/DAP/CFLAR/PML/CASP8
GOBP_CELLULAR_TRANSITION_METAL_ION_HOMEOSTASIS
GOBP_CELLULAR_TRANSITION_METAL_ION_HOMEOSTASIS
GOBP_CELLULAR_TRANSITION_METAL_ION_HOMEOSTASIS 109 -0.411475012
-1.539051514 0.007272727 0.057900665 0.045823423 1051 tags=20%, list=6%, signal=19%
CCDC115/FTH1/MT2A/SMAD4/SLC30A1/MT1HL1/SRI/SLC30A4/MT1E/MT1X/MT1H/SLC39A5/APP/SLC46A1/MT1G/FLVCR1/MT1F/HMOX1/SLC1A1/MT1M/SLC30A10/ABCG2
GOBP_RESPONSE_TO_XENOBIOTIC_STIMULUS GOBP_RESPONSE_TO_XENOBIOTIC_STIMULUS
GOBP_RESPONSE_TO_XENOBIOTIC_STIMULUS 109 -0.420989591 -1.57463916
0.007272727 0.057900665 0.045823423 2165 tags=28%, list=13%, signal=25%
AKR1C1/GSTA1/GLYAT/AKR7A3/GSTM1/MGST3/ARNT/GSTM2/NQO1/CYP2J2/BPHL/POR/CYP1A1/GSTM3/AHRR/CYB5B/ACAA1/EPHX1/NAT2/CES3/NR1I2/SULT1A1/UGT2B28/AOC1/SULT1A2/CMBL/GSTM4/CES2/SULT1B1/CYP2S1/CYP2B6
GOBP_CELLULAR_RESPONSE_TO_CAFFEINE GOBP_CELLULAR_RESPONSE_TO_CAFFEINE
GOBP_CELLULAR_RESPONSE_TO_CAFFEINE 10 -0.774276637 -1.734467144 0.007281553
0.057900665 0.045823423 1535 tags=40%, list=9%, signal=36%
CACNA1S/GSTM2/TMEM38B/PPARGC1A
GOBP_FATTY_ACID_BETA_OXIDATION_USING_ACYL_COA_DEHYDROGENASE
GOBP_FATTY_ACID_BETA_OXIDATION_USING_ACYL_COA_DEHYDROGENASE

GOBP_FATTY_ACID_BETA_OXIDATION_USING_ACYL_COA_DEHYDROGENASE 10 -0.824263745 -1.846443914 0.007281553 0.057900665 0.045823423 2302 tags=70%, list=14%, signal=61% IVD/ETFBKMT/ETFB/ETFA/ACADS/ACADM/ETFDH

GOBP_SOMITE_ROSTRAL_CAUDAL_AXIS_SPECIFICATION GOBP_SOMITE_ROSTRAL_CAUDAL_AXIS_SPECIFICATION GOBP_SOMITE_ROSTRAL_CAUDAL_AXIS_SPECIFICATION 10 -0.785093897 -1.758699029 0.007281553 0.057900665 0.045823423 1000 tags=40%, list=6%, signal=38% EPB41L5/SMAD4/NRARP/MESP1

GOBP_DETOXIFICATION GOBP_DETOXIFICATION GOBP_DETOXIFICATION 121 -0.374027907 -1.407724939 0.00729927 0.057900665 0.045823423 2061 tags=30%, list=12%, signal=26% GSTA1/GSR/AKR7A3/ADH4/TP53INP1/GSTM1/MGST3/GPX4/MT3/GSTM2/PRDX5/NQO1/GSTM3/SESN1/PIM1/MT2A/IYD/NNT/CAT/AKR1B10/APOM/SLC30A1/MT1HL1/MT1E/FABP1/MT1X/MT1H/PRXL2A/PRDX6/MT1G/MT1F/PDZK1/SLC22A5/MT1M/SLC30A10/ABCG2

GOBP_SOMATIC_DIVERSIFICATION_OF_IMMUNE_RECEPTORS GOBP_SOMATIC_DIVERSIFICATION_OF_IMMUNE_RECEPTORS GOBP_SOMATIC_DIVERSIFICATION_OF_IMMUNE_RECEPTORS 70 0.524662739 1.613929477 0.007309942 0.057900665 0.045823423 4782 tags=51%, list=28%, signal=37% TGFB1/IL27RA/PTPRC/TNFSF13/MAD2L2/SAMHD1/CD40/BCL6/LEF1/CCR6/TCF3/EXOSC3/EXO1/MSH6/MLH1/BATF/NBN/MCM3AP/SWAP70/ADAR/THOC1/POLB/RNF8/HSPD1/SLC15A4/HMGB1/CTNNBL1/TCF7/SUPT6H/IL4/CLCF1/ATM/PRKDC/DCLRE1C/TP53BP1/DCAF1

GOBP_INORGANIC_ANION_TRANSMEMBRANE_TRANSPORT GOBP_INORGANIC_ANION_TRANSMEMBRANE_TRANSPORT GOBP_INORGANIC_ANION_TRANSMEMBRANE_TRANSPORT 116 -0.419189569 -1.573074726 0.007326007 0.057900665 0.045823423 1240 tags=16%, list=7%, signal=14% ANO10/ANO5/CFTR/SLC20A1/BEST4/FXYD3/CLDN4/ANKH/CLIC5/SLC37A2/SLC20A2/CLCN2/BEST2/SLC26A3/CLCA4/GABRA2/SLC1A1/SLC26A2

GOBP_CELLULAR_RESPONSE_TO_ACID_CHEMICAL GOBP_CELLULAR_RESPONSE_TO_ACID_CHEMICAL GOBP_CELLULAR_RESPONSE_TO_ACID_CHEMICAL 68 0.516330698 1.582563441 0.007331378 0.057900665 0.045823423 1411 tags=24%, list=8%, signal=22% COL4A1/COL1A2/COL1A1/COL5A2/MMP2/COL6A1/ASS1/XBP1/PDGFRA/FYN/CEBPB/CPEB4/KLF2/COL16A1/HNRNPD/PKD2

GOBP_PHOSPHOLIPID_METABOLIC_PROCESS GOBP_PHOSPHOLIPID_METABOLIC_PROCESS GOBP_PHOSPHOLIPID_METABOLIC_PROCESS 393 -0.298930168 -1.28761594 0.007352941 0.057900665 0.045823423 2107 tags=21%, list=12%, signal=19% BMX/MPPE1/PLEKHA3/DOLPP1/CRLS1/PLEKHA1/SCARB1/PIGL/GPD1/PCTP/CDS1/GPX4/INPP5K/ABHD4/PLA2G4C/SLC44A3/PAFAH1B1/SLC44A5/CAPN2/SLC44A1/SERINC2/ARF3/SOCS2/SERINC1/PLAAT4/PIK3C2B/PLA2G12A/ABHD5/HADHB/DDHD2/LIPH/CDIPT/PIGV/NR1H3/LPIN2/CHPT1/SPHK2/FAM126B/IMPA2/INPP5J/INPP4B/DGKQ/IMPA1/CHKA/ABHD12/OCRL/PPARD/SOCS6/PGAP3/PLD1/GDE1/HADHA/SERINC5/ALPI/PNLIPRP2/GPD1L/ABHD3/PGAP1/LIPC/MTMR11/NR1H4/GATA6/PLCD1/NAPEPLD/PIP5K1B/ITPKA/LPCAT3/IP6K2/PRDX6/BPNT1/GDPD1/ABCA8/PLPP1/

MTM1/ETNK1/PLAAT2/TMEM38B/PIGZ/PLA2G12B/NAAA/CWH43/HMGCS2
GOBP_POSITIVE_REGULATION_OF_PROTEIN_CONTAINING_COMPLEX_ASSEMBLY GOBP_POSITIVE_REGULATION_OF_PROTEIN_CONTAINING_COMPLEX_ASSEMBLY GOBP_POSITIVE_REGULATION_OF_PROTEIN_CONTAINING_COMPLEX_ASSEMBLY 243 0.401667178 1.455032852 0.007352941 0.057900665 0.045823423 3324 tags=29%, list=20%, signal=24% MMP1/MMP3/CCL11/CXCL13/WARS1/FERMT2/MSN/CDH5/TGFB1/CLU/ASAP1/CLEC7A/ARPC1B/CCR7/PLEK/GMFG/TRIM27/HCK/ICAM1/BAIAP2L1/PIEZO1/MET/CCL24/CORO1A/GBP5/LCP1/VCP/CDC42EP1/FCHSD2/PSMC2/ARPC5L/BID/ATR/FSCN1/AJUBA/MAP1B/PARP1/IFNG/PSMC4/CREB1/SPIRE1/ARFIP2/EIF4G1/NCKAP1L/TCL1A/PSRC1/FERMT1/SYK/CDT1/PARK7/DDB2/PSMC5/CDC42EP2/WAS/CLIP1/ACTR3/PPP2R5B/CHD1L/FES/BIK/HSP90AA1/EVL/CTTN/RASIP1/RHOC/PIH1D1/ABI2/WASF2/DHX33/DDB1/BIN1
GOBP_ANIMAL_ORGAN_REGENERATION GOBP_ANIMAL_ORGAN_REGENERATION GOBP_ANIMAL_ORGAN_REGENERATION 71 0.516112243 1.587350924 0.00736377 0.057900665 0.045823423 2060 tags=31%, list=12%, signal=27% REG1A/NNMT/CLDN1/PKM/LPIN1/ANXA3/UCP2/C5AR1/TGFBR2/CEBPB/PPAT/SULF2/LCP1/EZH2/AURKA/CCND1/TYMS/FPGS/IL6/AXL/PRMT5/CAD
GOBP_MEMBRANE_PROTEIN_PROTEOLYSIS GOBP_MEMBRANE_PROTEIN_PROTEOLYSIS GOBP_MEMBRANE_PROTEIN_PROTEOLYSIS 60 0.537404392 1.614226722 0.007374631 0.057900665 0.045823423 4011 tags=48%, list=24%, signal=37% TIMP1/IL1B/MMP7/BACE2/CTSH/TGFB1/HM13/ADAM19/ADAM9/TNFRSF1B/ADAM8/APH1B/IFNG/TIMP2/RELA/NRDC/FURIN/TIMP3/BACE1/APOE/ROCK1/CLPP/MBTPS1/RHBDD1/ADAM17/PSENEN/TIMP4/ERAP1/PACSIN3
GOBP_NEGATIVE_REGULATION_OF_INNATE_IMMUNE_RESPONSE GOBP_NEGATIVE_REGULATION_OF_INNATE_IMMUNE_RESPONSE GOBP_NEGATIVE_REGULATION_OF_INNATE_IMMUNE_RESPONSE 60 0.540750586 1.624277841 0.007374631 0.057900665 0.045823423 3688 tags=42%, list=22%, signal=33% MMP12/SERPINB9/SERPING1/IFI16/SLAMF8/SERPINB4/NLRC5/SAMHD1/LYAR/PARP14/A2M/METTL3/DUSP10/LILRB1/HAVCR2/NMI/ADAR/TYRO3/ARRB2/TRIM21/IRAK3/TRAFD1/CR1/DHX58/HLA-E
GOBP_NEGATIVE_REGULATION_OF_INTRACELLULAR_TRANSPORT GOBP_NEGATIVE_REGULATION_OF_INTRACELLULAR_TRANSPORT GOBP_NEGATIVE_REGULATION_OF_INTRACELLULAR_TRANSPORT 54 0.546805204 1.626274471 0.007407407 0.05806256 0.045951549 3555 tags=44%, list=21%, signal=35% DERL3/BAG3/PLN/PCSK9/ERLEC1/LMAN1/RAB23/UBE2J1/INSIG1/ITGB1BP1/BARD1/RANGAP1/CRYAB/MAP1B/PKIA/APOD/DERL2/LRRK2/FERMT1/PARK7/UFM1/UBE2G2/GDI1/PKIG
GOBP_RUFFLE_ORGANIZATION GOBP_RUFFLE_ORGANIZATION GOBP_RUFFLE_ORGANIZATION 53 0.553350998 1.63944078 0.007418398 0.05806256 0.045951549 2045 tags=34%, list=12%, signal=30% CAV1/EPS8L1/TCIRG1/CCR7/PLEK/ICAM1/FAM98A/SNX10/CSF1R/TACSTD2/CORO1C/RCC2/RHOG/ARHGAP24/AIF1/RDX/DEF8/ARFIP2
GOBP_CELL_CELL_ADHESION_VIA_PLASMA_MEMBRANE_ADHESION_MOLECULES

GOBP_CELL_CELL_ADHESION_VIA_PLASMA_MEMBRANE_ADHESION_MOLECULES
GOBP_CELL_CELL_ADHESION_VIA_PLASMA_MEMBRANE_ADHESION_MOLECULES 210
0.402417176 1.438039129 0.007462687 0.058318222 0.046153884 1677
tags=18%, list=10%, signal=17%
REG3A/IL1RN/PECAM1/CLDN1/SELL/CDH3/CADM1/ROBO1/CDH11/SPARCL1/SELP/CDH5/DSG3/ALCAM/PCDH17/ITGA5/EMB/VCAM1/TGFBR2/FXYD5/CLDN2/ITGB2/PTPRM/SELE/ICAM1/PCDH18/ITGAM/PALLD/ROBO4/CLDN18/CD84/FLRT3/CLDN12/GPC6/NEXN/DCHS1/ACVR1/AJUBA
GOBP_SMALL_MOLECULE_CATABOLIC_PROCESS
GOBP_SMALL_MOLECULE_CATABOLIC_PROCESS
GOBP_SMALL_MOLECULE_CATABOLIC_PROCESS 408 -0.371289846 -1.602585029
0.007518797 0.058665326 0.046428587 2591 tags=32%, list=15%, signal=28%
TYSND1/PFKL/ACACB/NUDT18/ADAL/CSAD/FAH/ADH5/INPP5A/GLYCTK/OAT/SYNJ1/TAT/IVD/GK5/ENPP4/ECHS1/BAD/DAO/ETFBKMT/ALDH1B1/ALDH6A1/OTC/SDSL/NUDT16/RIDA/NUDT15/ADTRP/IRS2/ACAD10/PECR/ADH4/DDO/CYP4F3/SCARB1/CRAT/HAGH/FAAH/SCP2/SLC25A12/INPP5K/GLUD1/DLD/GPT/DECR1/MT3/ECHDC2/HMGCL/NT5E/ALDH1A1/ETFB/CYP1A1/PEX13/HADHB/ACBD5/LPIN2/HIBCH/GLS/ECI1/PPARA/PNKD/AKR1C3/PCCA/GALM/ETFA/PCK2/IMPA2/INPP5J/PGM1/ALDOC/GLUD2/PHYKPL/IMPA1/DERA/QPRT/AMT/ECH1/OCRL/AKR1B10/PPARD/ACAA1/BDH1/ACOT8/ACOX2/ABCD3/BDH2/ACOT4/HADH/CRYL1/HADHA/BCKDHB/TST/MPST/ACAA2/ABHD3/BCAT2/RBKS/CYP4F12/CPT2/ACADSB/LDHD/FABP1/SULT1A1/CROT/ASPA/CYP4F2/SULT1A2/CBS/CTH/PFKFB2/ALDH5A1/HNMT/CPT1A/ACADS/AUH/EHHADH/SULT1B1/BPNT1/PHYH/NUDT7/ACADM/CYP27A1/ACAT1/ETFDH/DDAH2/ABCB11/ABAT/ACOX1/ENTPD5/APOBEC3B/PCK1
GOBP_COENZYME_A_METABOLIC_PROCESS GOBP_COENZYME_A_METABOLIC_PROCESS
GOBP_COENZYME_A_METABOLIC_PROCESS 15 -0.726432744 -1.82331515 0.007556675
0.058869315 0.046590027 535 tags=40%, list=3%, signal=39%
HMGCR/PANK3/CROT/NUDT7/ACAT1/PANK1
GOBP_RIBONUCLEOPROTEIN_COMPLEX_SUBUNIT_ORGANIZATION
GOBP_RIBONUCLEOPROTEIN_COMPLEX_SUBUNIT_ORGANIZATION
GOBP_RIBONUCLEOPROTEIN_COMPLEX_SUBUNIT_ORGANIZATION 180 0.412767371
1.449888384 0.007643312 0.059372781 0.046988477 5317 tags=47%, list=31%, signal=32%
NOP2/AGO2/VCP/RRS1/RUVBL1/WDR77/EIF2S2/EIF3B/RRP7A/MRTO4/ATR/EIF3J/AGO3/SNRPG/SNRPD1/PRMT5/SF1/EIF3I/CELF2/POLR2D/MRPL20/DICER1/SETX/STRAP/MCTS1/SNRPF/HSP90AB1/ADAR/RUVBL2/PRPF31/SNRPB/LUC7L2/GEMIN6/GEMIN8/EIF3G/HSP90AA1/GEMIN5/LUC7L/DHX30/PIH1D1/RPF2/DHX29/BRIX1/TXNL4A/TARBP2/XRCC5/SNRPD2/SNRPC/SRSF1/DDX23/DENR/KLC1/SART3/ZRSR2/SFSWAP/SART1/DDX20/CD2BP2/SNRPE/SF3A2/SRSF6/PRPF3/CPSF7/KHDC4/LUC7L3/XAB2/GEMIN4/EIF3M/PTBP2/ATM/PUF60/NLE1/CPSF6/PRKDC/CELF5/ZNHIT6/PRPF8/PRPF19/DDX1/MDN1/TFIP11/FASTKD2/DHX8/LSM2
GOBP_CELLULAR_RESPONSE_TO_RADIATION GOBP_CELLULAR_RESPONSE_TO_RADIATION
GOBP_CELLULAR_RESPONSE_TO_RADIATION 182 0.413057115 1.454393778
0.007653061 0.059372781 0.046988477 3650 tags=33%, list=22%, signal=26%
MMP1/MMP3/TIMP1/MMP9/MMP2/HYAL1/IFI16/CARD16/HSPA5/POLD3/HYAL2/H2AX/CDC25A/MAP3K20/BLM/EIF2S1/MME/ECT2/AURKB/METTL3/PBK/ATR/NMT1/TMEM109/TNKS1BP1/CRYAB/NEDD4/SFRP1/PARP1/FIGNL1/SFRP2/MFAP4/NOC2L/AQP1/CHEK1/RAD1/RUVBL2/ATF4/NPM1/FBXO4/TANK/DDB2/TSPYL5/COPS9/TRIAP1/CRY1/HRAS/EEF1D/TMEM161A/HYAL3/DDB1/

ACTR5/USP47/XRCC5/BAK1/RAD51AP1/GRK1/MDM2/METAP2/RHBDD1
GOBP_AMINO_ACID_BETAINE_METABOLIC_PROCESS
GOBP_AMINO_ACID_BETAINE_METABOLIC_PROCESS
GOBP_AMINO_ACID_BETAINE_METABOLIC_PROCESS 16 -0.751323409 -1.919559103
0.007672634 0.059372781 0.046988477 1901 tags=44%, list=11%, signal=39%
CRAT/TMLHE/CPT2/CROT/CPT1A/ACADM/SLC22A4
GOBP_BLOOD_VESSEL_REMODELING GOBP_BLOOD_VESSEL_REMODELING
GOBP_BLOOD_VESSEL_REMODELING 39 0.577984147 1.624989838 0.007692308
0.059372781 0.046988477 2751 tags=33%, list=16%, signal=28%
AGT/TGM2/CCR2/TGFB1/BGN/RSPO3/FLNA/LIF/AXL/JAG1/ACE/ATP7A/CHD7
GOBP_GLYCEROLIPID_METABOLIC_PROCESS GOBP_GLYCEROLIPID_METABOLIC_PROCESS
GOBP_GLYCEROLIPID_METABOLIC_PROCESS 402 -0.316265836 -1.36036553 0.007692308
0.059372781 0.046988477 1986 tags=26%, list=12%, signal=24%
MTMR3/AGPAT3/MOGAT3/SLC44A4/FAR1/ITPKC/PIGN/SH3YL1/SERINC3/FITM2/AVIL/INPP5A/SACM1L/SYNJ1/AGPAT2/GK5/PNPLA7/BMX/MPPE1/PLEKHA3/CRLS1/PNPLA4/PLEKHA1/SCARB1/PIGL/FAAH/GPD1/PCTP/CDS1/APOBR/FABP2/INPP5K/ABHD4/PLA2G4C/SLC44A3/PAFAH1B1/SLC44A5/CAPN2/SLC44A1/SERINC2/ARF3/SOCS2/SERINC1/PLAAT4/PIK3C2B/PLA2G12A/ABHD5/HADHB/SIRT1/DDHD2/LIPH/CDIPT/PIGV/NR1H3/LPIN2/CHPT1/FAM126B/PCK2/IMPA2/INPP5J/INPP4B/DGKQ/IMPA1/CAT/CHKA/ABHD12/OCRL/SOCS6/PGAP3/ATG14/PLD1/GAL3ST1/GDE1/HADHA/SERINC5/ALPI/PNLIPRP2/GPD1L/ABHD3/PGAP1/LIPC/FABP1/MTMR11/NR1H4/MGLL/PLCD1/NAPEPLD/PIP5K1B/MOGAT2/ITPKA/LPCAT3/CPT1A/IP6K2/SORL1/PLCE1/PRDX6/BPNT1/GDPD1/SLC22A4/MTM1/ETNK1/PLAAT2/PIGZ/NAAA/CWH43/PCK1
GOBP_REGULATION_OF_KERATINOCYTE_DIFFERENTIATION
GOBP_REGULATION_OF_KERATINOCYTE_DIFFERENTIATION
GOBP_REGULATION_OF_KERATINOCYTE_DIFFERENTIATION 39 0.577637345
1.624014811 0.007692308 0.059372781 0.046988477 1791 tags=26%, list=11%, signal=23% REG3A/AQP3/CTSK/CBFB/TRIM16/CYP27B1/PRKCH/CD109/CTSL/ZBED2
GOBP_MATURATION_OF_SSU_RRNA GOBP_MATURATION_OF_SSU_RRNA
GOBP_MATURATION_OF_SSU_RRNA 49 0.549190972 1.597830743 0.007727975
0.059556455 0.047133839 5122 tags=61%, list=30%, signal=43%
UTP4/RRP36/TSR1/RRS1/BYSL/NAT10/UTP6/RPP40/NOP14/DCAF13/RIOK1/TBL3/HEATR1/BMS1/UTP3/SRFBP1/LSM6/NOB1/NOL10/WDR46/WDR3/UTP25/NOP9/SNU13/NOL11/GTF2H5/TSR2/TSR3/RIOK2/UTP20
GOBP_FC_RECEPTOR_SIGNALING_PATHWAY GOBP_FC_RECEPTOR_SIGNALING_PATHWAY
GOBP_FC_RECEPTOR_SIGNALING_PATHWAY 172 0.420985734 1.469003493 0.007782101
0.059744967 0.047283031 3961 tags=42%, list=23%, signal=32%
FCGR2A/LYN/LCP2/FCGR1B/PTPRC/FCER1G/PSME4/PSMB9/KIT/ARPC1B/FYN/FCGR2B/FGR/HCK/PLCG2/WIPF1/PSMD14/VAV1/PSMD12/PSMC2/PSMB2/PSMA3/PSMD1/BTK/ITK/CD47/ELMO1/PSMA5/PSMD6/PSMB5/PSMC4/RELA/NCKAP1L/PPP3CA/PSMB1/PSMA1/MALT1/PPP3R1/LAT2/HSP90AB1/PSME3/SYK/CUL1/PSMA7/PSMB8/PSMC5/WAS/ACTR3/LIMK1/NR4A3/PSMB10/HSP90AA1/NFATC3/PSMB6/PSMA4/LAT/PSMD13/SHC1/WASF2/PSME1/MYO1G/CD247/CARD11/PSMC1/NFATC2/PSMB4/PSMB3/PSMD2/IKBKG/PSMC6/PSMD7/PSMA2
GOBP_REGULATION_OF_ANIMAL_ORGAN_MORPHOGENESIS
GOBP_REGULATION_OF_ANIMAL_ORGAN_MORPHOGENESIS

GOBP_REGULATION_OF_ANIMAL_ORGAN_MORPHOGENESIS 172 0.413896475 1.444265966 0.007782101 0.059744967 0.047283031 4036 tags=42%, list=24%, signal=32%
CTHRC1/GREM1/AGT/WNT5A/ROBO1/SULF1/TNFRSF11B/RSPO3/PSME4/PSMB9/SHH/DACT1/DAB2/PSMD14/PSMD12/PSMC2/TACSTD2/DKK1/PSMB2/PSMA3/PSMD1/GPC6/PSMA5/AHI1/SFRP1/FGF7/PSMD6/CELSR1/PSMB5/PSMC4/VANGL1/FZD4/WNT4/SFRP2/PSMB1/PSMA1/WNT2/PSME3/PLEKHA4/PSMA7/PSMB8/PSMC5/ARRB2/DVL1/SMURF1/CD34/PSMB10/HOXA11/SPRY1/PSMB6/PSMA4/AP2B1/PSMD13/PSME1/PRICKLE1/SAPCD2/PSMC1/SMURF2/WNT2B/DVL3/PSMB4/ARHGEF19/AP2S1/SOX9/PSMB3/PSMD2/PSMC6/PSMD7/PSMA2/SP6/ANKRD6/PSMD4
GOBP_REGULATION_OF_PHOSPHOLIPASE_C_ACTIVITY GOBP_REGULATION_OF_PHOSPHOLIPASE_C_ACTIVITY GOBP_REGULATION_OF_PHOSPHOLIPASE_C_ACTIVITY 45 0.574234378 1.650760939 0.007788162 0.059744967 0.047283031 1973 tags=27%, list=12%, signal=24% GNA15/EDNRA/C5AR1/KIT/PDGFRA/CD86/SELE/PTAFR/PDGFRB/FGFR1/ITK/ANG
GOBP_INTERLEUKIN_17_PRODUCTION GOBP_INTERLEUKIN_17_PRODUCTION GOBP_INTERLEUKIN_17_PRODUCTION 35 0.591897377 1.628036372 0.007824726 0.059933818 0.04743249 2677 tags=40%, list=16%, signal=34% SLC7A5/RFTN1/TGFB1/IL27RA/SPHK1/PRNP/IL23A/IL6/IFNG/SLAMF6/NCKAP1L/NOD2/LY9/IL18
GOBP_CYTOLYSIS GOBP_CYTOLYSIS GOBP_CYTOLYSIS 30 0.605397115 1.625219243 0.007849294 0.060030344 0.047508882 1433 tags=27%, list=8%, signal=24% PLA2G2A/TGFB1/GZMB/APOL1/MICB/KRT6A/LYZ/MMD
GOBP_RESPONSE_TO_DIETARY_EXCESSGOBP_RESPONSE_TO_DIETARY_EXCESS GOBP_RESPONSE_TO_DIETARY_EXCESS22 -0.668856273 -1.80827504 0.007936508 0.060526436 0.047901497 358 tags=41%, list=2%, signal=40% PCSK1N/PRLH/OMA1/SLC25A25/ADRB1/SORL1/ACVR1C/PPARGC1A/APPL2
GOBP_ESTABLISHMENT_OF_PROTEIN_LOCALIZATION_TO_CHROMOSOME GOBP_ESTABLISHMENT_OF_PROTEIN_LOCALIZATION_TO_CHROMOSOME GOBP_ESTABLISHMENT_OF_PROTEIN_LOCALIZATION_TO_CHROMOSOME 24 0.644871476 1.655601191 0.007949126 0.060526436 0.047901497 3120 tags=54%, list=18%, signal=44% ACD/ATR/CCT3/CCT8/CCT5/CCT2/CCT6A/CCT4/CCT7/RUVBL2/NABP2/MACROH2A2/PIH1D1
GOBP_DETECTION_OF_OTHER_ORGANISM GOBP_DETECTION_OF_OTHER_ORGANISM GOBP_DETECTION_OF_OTHER_ORGANISM 18 0.68021278 1.649844364 0.007974482 0.060526436 0.047901497 2123 tags=28%, list=13%, signal=24% DMBT1/CLEC7A/TLR2/TLR1/NOD2
GOBP_NEGATIVE_REGULATION_OF_LEUKOCYTE_MEDIATED_CYTOTOXICITY GOBP_NEGATIVE_REGULATION_OF_LEUKOCYTE_MEDIATED_CYTOTOXICITY GOBP_NEGATIVE_REGULATION_OF_LEUKOCYTE_MEDIATED_CYTOTOXICITY 21 0.675811533 1.692324546 0.007974482 0.060526436 0.047901497 1976 tags=33%, list=12%, signal=29% SERPINB9/IL7R/PTPRC/SERPINB4/FCGR2B/LILRB1/HAVCR2
GOBP_POSITIVE_REGULATION_OF_ALPHA_BETA_T_CELL_PROLIFERATION GOBP_POSITIVE_REGULATION_OF_ALPHA_BETA_T_CELL_PROLIFERATION

GOBP_POSITIVE_REGULATION_OF_ALPHA_BETA_T_CELL_PROLIFERATION 21 0.674466212 1.688955679 0.007974482 0.060526436 0.047901497 2677 tags=52%, list=16%, signal=44% CD55/CCR2/PTPRC/RIPK2/TGFBR2/CD81/EBI3/IL23A/SYK/RASAL3/IL18
GOBP_MATURATION_OF_5_8S_RRNA_FROM_TRICISTRONIC_RRNA_TRANSCRIPT_SSU_RRNA_5_8S_RRNA_LSU_RRNA GOBP_MATURATION_OF_5_8S_RRNA_FROM_TRICISTRONIC_RRNA_TRANSCRIPT_SSU_RRNA_5_8S_RRNA_LSU_RRNA GOBP_MATURATION_OF_5_8S_RRNA_FROM_TRICISTRONIC_RRNA_TRANSCRIPT_SSU_RRNA_5_8S_RRNA_LSU_RRNA 23 0.646813614 1.649825988 0.008025682 0.060548643 0.047919071 3721 tags=65%, list=22%, signal=51% ERI1/RRS1/EXOSC3/RPP40/NOP14/EXOSC7/FTSJ3/EXOSC9/EXOSC8/URB1/NOL9/WDR12/PES1/EXOSC10/NOP9
GOBP_OLFACTORY_LOBE_DEVELOPMENT GOBP_OLFACTORY_LOBE_DEVELOPMENT GOBP_OLFACTORY_LOBE_DEVELOPMENT 26 0.625061445 1.631394393 0.008025682 0.060548643 0.047919071 2751 tags=35%, list=16%, signal=29% WNT5A/ROBO1/EFNA2/KIF14/CSF1R/ATF5/SALL1/LRRK2/CHD7
GOBP_POSITIVE_REGULATION_OF_SMOOTH_MUSCLE_CONTRACTION GOBP_POSITIVE_REGULATION_OF_SMOOTH_MUSCLE_CONTRACTION GOBP_POSITIVE_REGULATION_OF_SMOOTH_MUSCLE_CONTRACTION 26 0.625364893 1.632186385 0.008025682 0.060548643 0.047919071 1354 tags=38%, list=8%, signal=35% PROK2/ITGA2/ADA/F2R/KIT/PTAFR/MYOCD/NMU/SPHK1/CHRM3
GOBP_PYRIMIDINE_RIBONUCLEOTIDE_METABOLIC_PROCESS GOBP_PYRIMIDINE_RIBONUCLEOTIDE_METABOLIC_PROCESS GOBP_PYRIMIDINE_RIBONUCLEOTIDE_METABOLIC_PROCESS 26 0.621176106 1.621253756 0.008025682 0.060548643 0.047919071 2060 tags=35%, list=12%, signal=30% CTPS1/NME1/ENTPD7/NME5/UPP1/UMPS/NME4/NME7/CAD
GOBP_REGULATION_OF_CELL_SHAPE GOBP_REGULATION_OF_CELL_SHAPE GOBP_REGULATION_OF_CELL_SHAPE 145 0.433145297 1.482504838 0.008053691 0.060577765 0.047942119 2508 tags=32%, list=15%, signal=27% CCL11/ANXA1/RHOQ/FERMT2/MSN/RAC2/PLXNA1/PDPN/FN1/KIT/FYN/ITGB2/STRIP2/DLC1/PALMD/FGR/HCK/ICAM1/CCL2/SEMA4D/CCL24/PLXNC1/LST1/DIAPH1/RHOH/CORO1A/KDR/CDC42EP1/PLEKHO1/CSF1R/S100A13/PARVB/MYH10/PLXND1/RHOG/FMNL2/FGD2/RDX/FMNL1/CDC42SE1/SEMA4A/PLXNA3/PARVA/RHOJ/FGD3/CCL7
GOBP_TOLL_LIKE_RECEPTOR_SIGNALING_PATHWAY GOBP_TOLL_LIKE_RECEPTOR_SIGNALING_PATHWAY GOBP_TOLL_LIKE_RECEPTOR_SIGNALING_PATHWAY 145 0.434121787 1.485847023 0.008053691 0.060577765 0.047942119 2924 tags=30%, list=17%, signal=25% S100A8/TNIP3/S100A9/CTSK/CAV1/LYN/RFTN1/LY96/BIRC3/PIK3AP1/IRF4/RIPK2/TLR8/ITGB2/TLR2/CD300A/IRAK1/CD40/ITGAM/LTF/IRF1/CTSB/FCRL3/IRAK2/HSP90B1/TLR1/BTK/CASP8/CD300LF/CTSL/HAVCR2/CNPY3/PTPRS/NOD2/IRF7/MAPKAPK2/NMI/TRAF3/TYRO3/TANK/IRF3/ARRB2/TBK1/IRAK3
GOBP_EPITHELIAL_TO_MESENCHYMAL_TRANSITION GOBP_EPITHELIAL_TO_MESENCHYMAL_TRANSITION GOBP_EPITHELIAL_TO_MESENCHYMAL_TRANSITION 144 0.436949408 1.495999286

0.008086253 0.060731636 0.048063895 2506 tags=33%, list=15%, signal=29%
GREM1/LOXL2/IL1B/WNT5A/COL1A1/RGCC/SERPINB3/FERMT2/ENG/TGFB1/FAM83D/PDPN/HIF1A/TWIST1/TGFBR2/MAD2L2/TGFB1I1/S100A4/FLNA/SNAI2/LEF1/DAB2/WWTR1/RFLNB/EZH2/SPRED1/FGFR1/OLFM1/NOLC1/ACVR1/ADAM15/SDCBP/IL6/SFRP1/JAG1/PHLDB2/WNT4/TGFBR1/SFRP2/MCRIP1/STRAP/WNT2/VASN/FOXF2/FOXA2/TRIM28/TGFB3/MDK
GOBP_PHOSPHATIDYLGLYCEROL_ACYL_CHAIN_REMODELING
GOBP_PHOSPHATIDYLGLYCEROL_ACYL_CHAIN_REMODELING
GOBP_PHOSPHATIDYLGLYCEROL_ACYL_CHAIN_REMODELING 16 0.690302275
1.625745668 0.008183306 0.061368679 0.04856806 293 tags=19%, list=2%, signal=18% LPCAT1/PLA2G2A/LPGAT1
GOBP_NEGATIVE_REGULATION_OF_CELL_CYCLE_PROCESS
GOBP_NEGATIVE_REGULATION_OF_CELL_CYCLE_PROCESS
GOBP_NEGATIVE_REGULATION_OF_CELL_CYCLE_PROCESS 322 0.378132031 1.40818211
0.008206331 0.061449493 0.048632017 4774 tags=48%, list=28%, signal=35%
RRM2/RGCC/EVI2B/TRIP13/PSME4/PSMB9/CCNB1/NABP1/MAD2L2/SLFN11/CCL2/BUB1/CDC20/DACT1/DONSON/NDC80/BCL6/BLM/PSMD14/CALR/E2F6/CBX5/PTTG1/CDC6/EZH2/PSMD12/CDK1/PSMC2/MUC1/PSMB2/AURKB/PSMA3/AURKA/PSMD1/BUB1B/CCND1/PLK1/PKD2/MIF/PRMT1/LIF/PSMA5/CDKN2C/AVEN/PML/FHL1/TNKS1BP1/CDC7/CDK2/ZWINT/PSMD6/CNOT9/GPNMB/PSMB5/PSMC4/PLK2/JADE1/NBN/ATF5/RBBP8/ARID3A/PSMB1/PSMA1/CDK4/PINX1/CHEK1/UIMC1/CCNF/RAD1/HSP90AB1/PSME3/CLSPN/CDT1/CDK5RAP3/NABP2/FAM107A/SPDL1/NPM1/CNOT6L/CUL1/PSMA7/PSMB8/PSMC5/KLHL22/PRKAR1A/TRIAP1/PPP2R5B/NACC2/KNTC1/PSMB10/PSMB6/PSMA4/FBXO5/INTS3/PCBP4/PSMD13/PSME1/VPS4A/BRCA1/USP47/RINT1/PSMC1/DTL/ZNF830/NAA10/FBXL7/MDM2/ZFP36L1/ZNF207/PSMB4/CENPF/NUBP1/PSMB3/PSMD2/BUB3/TTK/KANK2/CHFR/PSMC6/MAD2L1/PSMD7/DYNC1LI1/PSMA2/L3MBTL2/MRE11/PSMD4/NEK2/KAT2A/RBM14/MDM4/PSMD9/CDK2AP2/FZD9/MRNIP/CNOT11/GTSE1/TFDP1/CBX3/ATM/YAF2/RBBP7/GML/BABAM2/E2F8/PSMB7/ZNF268/PRKDC/EED/CNOT3/ZW10/BRD7/GEN1/CDK5RAP2
GOBP_BILE_ACID_AND_BILE_SALT_TRANSPORT GOBP_BILE_ACID_AND_BILE_SALT_TRANSPORT
GOBP_BILE_ACID_AND_BILE_SALT_TRANSPORT 30 -0.600943038 -1.73494186
0.008219178 0.061453974 0.048635563 1732 tags=33%, list=10%, signal=30%
AKR1C1/ABCC3/RXRA/PRKAA1/ATP8B1/NCOA2/NR1H4/ABCB11/SLC51B/SLC51A
GOBP_GLYCOPROTEIN_BIOSYNTHETIC_PROCESS
GOBP_GLYCOPROTEIN_BIOSYNTHETIC_PROCESS
GOBP_GLYCOPROTEIN_BIOSYNTHETIC_PROCESS 320 0.384471842 1.429868764
0.008254717 0.06162785 0.048773171 2824 tags=30%, list=17%, signal=25%
SRD5A3/DERL3/BACE2/SDF2L1/CSGALNACT1/CHST15/PHLDA1/IL33/DPY19L1/MUC5B/BGN/JAK3/PGM3/CHST11/CHSY1/VCAN/FUT8/MUC4/ST3GAL4/DSE/AGO2/CCR7/TMEM165/DCN/GALNT2/TMTC1/TUSC3/NPC1/CHPF/RPN2/PMM2/ST6GALNAC4/LMAN1/B3GALT6/HS3ST3B1/CSGALNACT2/UBE2J1/ST3GAL5/GALNT6/PLCB1/MUC1/DPAGT1/B3GALNT2/CCL19/MCFD2/OSTC/GFPT2/MGAT1/GALNT10/GALNT18/ALG5/EXT2/ST6GAL2/UGGT2/ALG2/SLC35C1/FUT11/MUC5AC/CHST12/TMEM258/ST3GAL2/ITM2A/DPY19L3/FUT4/GALNT5/MUC2/PLOD3/B3GNT7/NUS1/B3GNT6/SERP1/DOLK/ALG8/RPN1/ALG9/MAN1A2/B4GALT2/KRTCAP2/B4GALT6/HS6ST2/OGT/RFT1/STT3A/POGLUT1/EOGT/UGGT1/CHPF2/MVD/CHST3/ST6GALNAC2/ST6GAL1/ATP7A/DDOST/EXTL2/GALNT8

GOBP_POSITIVE_REGULATION_OF_PROTEIN_SERINE_THREONINE_KINASE_ACTIVITY
GOBP_POSITIVE_REGULATION_OF_PROTEIN_SERINE_THREONINE_KINASE_ACTIVITY
GOBP_POSITIVE_REGULATION_OF_PROTEIN_SERINE_THREONINE_KINASE_ACTIVITY 295 0.396773745 1.465264322 0.008363202 0.062344998 0.049340733 2523 tags=26%, list=15%, signal=22% CEMIP/PROK2/IL1B/S100A12/AGT/WNT5A/RGCC/MLKL/ROBO1/FERMT2/FPR1/PEA15/CD74/TGFB1/TCIM/PTPRC/RIPK2/CCNB1/C5AR1/KIT/CXCR4/MAP3K5/DUSP6/DUSP7/PDGFRB/IRAK1/CD81/CD40/IGFBP6/MAP3K20/MAP3K6/LTF/MST1R/MAP4K1/CDC6/PIK3CG/EZH2/CDK1/CSF1R/FGFR1/ALS2/DKK1/CCL19/CCND1/IRAK2/PBK/PKD2/PIK3R5/CKS2/TPD52L1/AJUBA/ADAM8/ADORA2B/STK3/MAP2K1/ATP2B4/IFNG/ERN1/FGD2/FZD4/DBF4/TNFSF11/KSR1/NOD2/TCL1A/PSRC1/SHC2/PDGFC/MAPKAPK2/HACD3/LRRK2/PTPN1/RASGRP1/HSP90AB1/TGFB3/SYK
GOBP_NEGATIVE_REGULATION_OF_LOCOMOTION
GOBP_NEGATIVE_REGULATION_OF_LOCOMOTION
GOBP_NEGATIVE_REGULATION_OF_LOCOMOTION 294 0.396743843 1.464175585 0.008403361 0.062441326 0.049416968 2193 tags=26%, list=13%, signal=23% TIMP1/IGFBP5/GREM1/GJA1/CXCL13/WNT5A/ADAMTS9/RGCC/ROBO1/IL33/COL3A1/ADA/ENG/SERPINE1/CD74/TGFB1/IFITM1/MCTP1/SULF1/STC1/IL27RA/SLAMF8/BST2/SHH/DLC1/THY1/PTPRM/DCN/TIE1/ARHGDIB/CCL2/CD300A/DPYSL3/HYAL2/SEMA4D/IDH2/MYOCD/OSBPL8/SERPINF1/MMRN2/EMILIN1/CALR/SEMG1/LIMCH1/LRCH1/SPRED1/RIPOR2/PLCB1/TACSTD2/MCC/SEMA4B/ITGB1BP1/DUSP10/NRP1/MIF/CD200/CORO1C/DUSP22/ADAM15/VCL/RAP2A/GTPBP4/DACH1/SFRP1/ABHD2/ATP2B4/AIF1/JAG1/VASH1/APOD/PHLDB2/SEMA3G/WNT4/SFRP2/STRAP/SEMA4A
GOBP_REGULATION_OF_CELL_MORPHOGENESIS
GOBP_REGULATION_OF_CELL_MORPHOGENESIS
GOBP_REGULATION_OF_CELL_MORPHOGENESIS 294 0.391878314 1.446219441 0.008403361 0.062441326 0.049416968 2508 tags=23%, list=15%, signal=20% REG1A/OLFM4/CCL11/ANXA1/RHOQ/SPARC/WNT5A/GBP1/FERMT2/MSN/LRP8/CD44/RAC2/PLXNA1/PDPN/MFSD2A/FN1/ENPP2/KIT/FYN/ITGB2/STRIP2/DLC1/CXCR4/PALMD/POSTN/FGR/HCK/ICAM1/CCL2/SEMA4D/CCL24/PLXNC1/LST1/DIAPH1/RHOH/CORO1A/FLNA/KDR/P4HB/CALR/CDC42EP1/RASAL1/PLEKHO1/CSF1R/TACSTD2/S100A13/ITGB1BP1/NRP1/MACF1/PARVB/MYH10/CORO1C/PLXND1/RCC2/RHOG/FMNL2/FGD2/RDX/FMNL1/FZD4/CDC42SE1/SEMA4A/PLXNA3/PARVA/RHOJ/FGD3/MDK/CCL7
GOBP_REGULATION_OF_MAP_KINASE_ACTIVITY
GOBP_REGULATION_OF_MAP_KINASE_ACTIVITY
GOBP_REGULATION_OF_MAP_KINASE_ACTIVITY 293 0.384364685 1.417824219 0.008413462 0.062441326 0.049416968 3071 tags=30%, list=18%, signal=25% PROK2/IL1B/S100A12/AGT/WNT5A/TRIB2/CAV1/SERPINB3/MLKL/ROBO1/FPR1/LYN/PEA15/LAX1/CD74/TGFB1/PTPRC/RIPK2/DUSP4/DUSP14/C5AR1/KIT/CXCR4/MAP3K5/DUSP6/DUSP7/PDGFRB/CD300A/HYAL2/IRAK1/CD81/CD40/IGFBP6/RGS2/MAP3K20/MAP3K6/MST1R/DNAJA1/MAP4K1/PIK3CG/EZH2/SPRED1/CDK1/FGFR1/DKK1/RGS4/NUP62/CCL19/DUSP10/IRAK2/PBK/PIK3R5/TPD52L1/AJUBA/ADAM8/ADORA2B/SFRP1/MAP2K1/ERN1/FGD2/FZD4/TNFSF11/KSR1/NOD2/SFRP2/SHC2/PDGFC/MAPKAPK2/HACD3/LRRK2/PTPN1/RASGRP1/TGFB3/SYK/UCHL1/CDK5RAP3/RGS3/MAP3K11/LIME1/UBE2N/APOE/FGF2/IRAK3/PTPN11/SPRY1/HRAS/GSTP1
GOBP_PURINE_NUCLEOBASE_BIOSYNTHETIC_PROCESS

GOBP_PURINE_NUCLEOBASE_BIOSYNTHETIC_PROCESS
GOBP_PURINE_NUCLEOBASE_BIOSYNTHETIC_PROCESS 10 0.771258818 1.641322069 0.008474576 0.062801993 0.049702405 3294 tags=90%, list=19%, signal=73% ADA/PPAT/SHMT2/GART/PRPS1/GMPS/PAICS/HPRT1/APRT
GOBP_HAIR_FOLLICLE_MATURATION GOBP_HAIR_FOLLICLE_MATURATION GOBP_HAIR_FOLLICLE_MATURATION 12 0.739941522 1.621140142 0.008488964 0.062815832 0.049713357 479 tags=33%, list=3%, signal=32% SPINK5/WNT5A/CDH3/GAL
GOBP_NEGATIVE_REGULATION_OF_CYTOKINE_PRODUCTION
GOBP_NEGATIVE_REGULATION_OF_CYTOKINE_PRODUCTION
GOBP_NEGATIVE_REGULATION_OF_CYTOKINE_PRODUCTION 265 0.393484424 1.43817678 0.008505468 0.062845261 0.049736648 3393 tags=32%, list=20%, signal=26%
ANXA1/CD274/IDO1/TRIB2/RGCC/GBP1/CDH3/IL33/UBE2L6/TGFB1/SRGN/JAK3/INHBA/IL27RA/PTPRC/FN1/TLR8/CARD16/CLEC4A/TWIST1/CCR7/BST2/FCGR2B/TRIM27/NLRC5/TYROBP/SIRPA/BCL6/KLF2/LEF1/LAPTM5/LTF/BANK1/FFAR4/CD84/TWSG1/NLRP7/PRNP/HDAC7/BTK/CD200/LAG3/IL23A/PML/IL6/LILRB4/SERPINB1/AXL/LILRB1/IFNG/GPNMB/APOD/HAVCR2/DICER1/NCKAP1L/PTPRS/NOD2/INPP5D/SYT11/FURIN/REL/INHBB/CMKLR1/NMI/HSP90AB1/TGFB3/CYLD/LRRC32/DLL1/IRF3/ADCY7/ARRB2/BTN2A2/TBK1/IRAK3/CD34/GSTP1/ERRFI1/UFD1/SELENOS/CUEDC2/IL1RL1/HMGB1/CX3CL1/CR1/MAPKBP1
GOBP_NUCLEAR_CHROMOSOME_SEGREGATION
GOBP_NUCLEAR_CHROMOSOME_SEGREGATION
GOBP_NUCLEAR_CHROMOSOME_SEGREGATION 255 0.395583362 1.441106995 0.008526188 0.06290571 0.049784488 4917 tags=44%, list=29%, signal=31%
GEM/FAM83D/FEN1/TRIP13/CCNB1/MAD2L2/TUBG1/TTL/BUB1/CDC20/PRC1/NCAPG/NDC80/NEK6/RRS1/CDCA5/TACC3/PTTG1/KIF14/CDC6/MIS12/KNSTRN/TENT4A/ECT2/NUP62/CENPE/AURKB/BUB1B/NUSAP1/FANCD2/PLK1/UBE2C/RAN/SMC4/DLGAP5/KIF23/KNL1/CDC27/RCC2/ZWINT/NUDC/KPNB1/TRAPPC12/MLH1/KIF2C/PSRC1/HECW2/CDCA8/KIF4A/CDC26/PINX1/RAD51C/SPAG5/NCAPH/NCAPG2/CDT1/FBXW7/SPDL1/KLHL22/ACTR3/EML3/KNTC1/ANAPC7/EME1/FBXO5/SGO2/RACGAP1/SFPQ/SMC2/TUBG2/KLHDC8B/VPS4A/NUF2/CENPX/DSCC1/KIF18A/REC8/NAA10/KIF18B/ZNF207/DDX11/CENPF/BUB3/TTK/CHFR/TOP2A/MAD2L1/DYNC1LI1/MRE11/NEK2/PHF13/NDC1/NCAPD3/CCNE1/CENPK/P3H4/SMC1A/ATM/HNRNPU/CHMP7/HASPIN/ZW10/MLH3/GEN1/CDK5RAP2/CORT/NAA50/MUS81/SIRT2/MAD1L1/ANAPC5
GOBP_BONE_REMODELING GOBP_BONE_REMODELING GOBP_BONE_REMODELING 88 0.496596593 1.576464737 0.008595989 0.063327569 0.050118353 2912 tags=38%, list=17%, signal=31%
CTHRC1/GREM1/GJA1/SPP1/CD38/TGFB1/RAC2/TNFRSF11B/TCIRG1/GPR137B/SNX10/RASSF2/EFNA2/S1PR1/CLDN18/CSF1R/ACP5/IL6/ADAM8/SFRP1/RAB3D/DEF8/TMEM119/TMEM64/TNFSF11/P2RX7/INPP5D/TGFB3/MDK/SYK/SUCO/ADRB2/MITF
GOBP_VASCULOGENESIS GOBP_VASCULOGENESIS GOBP_VASCULOGENESIS 76 0.502699035 1.55649347 0.008708273 0.064060711 0.050698573 3097 tags=36%, list=18%, signal=29%
CAV1/ENG/HEG1/TGFB1/APLNR/QKI/TGFBR2/SHH/TIE1/FOXF1/PDGFRB/MYOCD/KDR/ZFPM2/XDH/SPRED1/ITGAV/EGFL7/FZD4/TEAD2/RAMP2/GLMN/FBXW7/CD34/EMP2/CCM2/RASIP1

GOBP_REGULATION_OF_TISSUE_REMODELING GOBP_REGULATION_OF_TISSUE_REMODELING GOBP_REGULATION_OF_TISSUE_REMODELING 70 0.511277558 1.572754952 0.00877193 0.064434512 0.050994404 2843 tags=39%, list=17%, signal=32% GREM1/SPP1/CD38/TGFB1/TNFRSF11B/GPR137B/DDR2/S1PR1/CLDN18/CSF1R/IL23A/IL6/ADAM8/SFRP1/GPNMB/DEF8/TMEM119/TMEM64/TNFSF11/PPP3CA/P2RX7/INPP5D/MDK/SYK/IL18/SUCO/ROCK1

GOBP_CELLULAR_RESPONSE_TO_MECHANICAL_STIMULUS GOBP_CELLULAR_RESPONSE_TO_MECHANICAL_STIMULUS GOBP_CELLULAR_RESPONSE_TO_MECHANICAL_STIMULUS 73 0.502038031 1.548047771 0.008888889 0.065198181 0.051598783 1204 tags=23%, list=7%, signal=22% GJA1/IL1B/MMP7/COL1A1/CASP1/ITGA2/ENG/BAG3/BNIP3/TLR8/PIEZO1/CD40/TNFRSF10A/CASP5/IRF1/TNFRSF10B/RIPOR2

GOBP_CARBOHYDRATE_DERIVATIVE_CATABOLIC_PROCESS GOBP_CARBOHYDRATE_DERIVATIVE_CATABOLIC_PROCESS GOBP_CARBOHYDRATE_DERIVATIVE_CATABOLIC_PROCESS 185 0.409122952 1.44207818 0.008905852 0.065227243 0.051621783 2117 tags=22%, list=13%, signal=19% CHI3L1/MMP12/CEMIP/ADAMTS9/HYAL1/ADA/CD44/FBXO6/TGFB1/PDE4B/BGN/CHI3L2/LUM/PNP/ENTPD7/VCAN/ADA2/APOBEC1/DCN/TYMP/HYAL2/SAMHD1/SDC2/APOBEC3G/AMPD3/XDH/UPP1/MGAT1/GM2A/GPC6/FMOD/HPSE/HGSNAT/CTSL/GLA/SDC3/AGRN/LYVE1/PDE7A/EDEM1

GOBP_REGULATION_OF_REACTIVE_OXYGEN_SPECIES_METABOLIC_PROCESS GOBP_REGULATION_OF_REACTIVE_OXYGEN_SPECIES_METABOLIC_PROCESS GOBP_REGULATION_OF_REACTIVE_OXYGEN_SPECIES_METABOLIC_PROCESS 175 0.412144698 1.441074336 0.009043928 0.066141961 0.052345704 1898 tags=23%, list=11%, signal=21% MMP3/DUOXA2/IL1B/AGT/CAV1/ALOX5/ASS1/TGFB1/HBB/TIGAR/RAC2/CLU/BNIP3/HIF1A/PTGS2/NQO2/CLEC7A/TGFBR2/FYN/ITGB2/JAK2/GNAI2/ICAM1/PDGFRB/TYROBP/FOXM1/SIRPA/ITGAM/KLF2/FBLN5/XDH/PKD2/HK2/CFLAR/CD47/CRYAB/GLA/PTGIS/ATP2B4/IFNG/FPR2

GOBP_RESPONSE_TO_RADIATION GOBP_RESPONSE_TO_RADIATION GOBP_RESPONSE_TO_RADIATION 429 0.358579672 1.35415849 0.009163803 0.066900469 0.052945998 3468 tags=27%, list=20%, signal=22% MMP1/MMP3/TIMP1/CCL11/ANXA1/CXCL10/MMP9/SLC7A11/THBD/MMP2/COL3A1/HYAL1/IKBIP/IFI16/TIGAR/FEN1/PTPRC/HIF1A/VCAM1/CARD16/KIT/HSPA5/NABP1/ICAM1/POLD3/HYAL2/ASNS/H2AX/CDC25A/MAP3K20/BLM/EIF2S1/PCLAF/NMU/MME/ECT2/AURKB/EYA3/PLEKHB1/METTL3/FANCD2/CCND1/PBK/AEN/ATR/BHLHE40/NMT1/UVSSA/TMEM109/TIPIN/COPS3/PML/TNKS1BP1/MSH6/CRYAB/NEDD4/SFRP1/PARP1/SCARA3/CREB1/HMGCS1/RELA/FIGNL1/SFRP2/MFAP4/RRM1/B4GALT2/NOC2L/SOD2/FANCG/AQP1/CHEK1/UIMC1/RAD1/GPX1/CCL7/BACE1/RUVBL2/ANGPT2/ATF4/NABP2/AKT1/NPM1/FBXO4/TANK/POLB/DDB2/TSPYL5/COPS9/ABCG5/TRIAP1/PITPNM1/CRY1/CCAR2/MTA1/PNKP/RO60/RNF8/HRAS/EEF1D/INTS3/TMEM161A/HYAL3/RAD54L/DDB1/BRCA1/USF1/ACTR5/USP47/POLG/XRCC5/BAK1/RAD51AP1/CLK2/GRK1

GOBP_REGULATION_OF_ENDOTHELIAL_CELL_MIGRATION GOBP_REGULATION_OF_ENDOTHELIAL_CELL_MIGRATION GOBP_REGULATION_OF_ENDOTHELIAL_CELL_MIGRATION 162 0.410875341 1.423007465 0.009174312 0.066900469 0.052945998 3086 tags=33%, list=18%, signal=27%

ANXA1/CXCL13/AGT/SPARC/WNT5A/ADAMTS9/RGCC/TGFB1/STC1/ANXA3/HIF1A/PTGS2/SMOC2/PTPRM/DCN/SRPX2/MET/CD40/SERPINF1/MMRN2/VEGFC/KDR/TEK/CALR/PIK3CG/ADGRA2/SPRED1/FGFR1/PIK3CD/ITGB1BP1/NRP1/HDAC7/AKT3/FGFBP1/ATP2B4/VASH1/NUS1/PLK2/SEMA4A/RHOJ/GLUL/FBXW7/ETS1/ANGPT2/AKT1/JCAD/APOE/FGF2/GPI/EMP2/STAT5A/NR2F2/PRCP
GOBP_GLAND_DEVELOPMENT GOBP_GLAND_DEVELOPMENT
GOBP_GLAND_DEVELOPMENT 395 0.356817593 1.341193342 0.00921659
0.067111221 0.05311279 2676 tags=24%, list=16%, signal=20%
REG1A/SERPINB5/IGFBP5/TNC/CCL11/ANXA1/WNT5A/CLDN1/TGM2/CAV1/ROBO1/MSN/SERPINE2/ITGA2/ADA/PKM/ASS1/DKK3/TGFB1/SULF1/PLXNA1/NME1/OAS2/HIF1A/XBP1/UCP2/TGFBR2/PDGFRA/JAK2/SHH/CEBPB/PPAT/FOXF1/GNPNAT1/SULF2/MET/MAFB/SERPINF1/PCSK9/ASNS/ALDH1A3/LEF1/FASN/XDH/GPAT4/PAM/WDR77/EZH2/CSF1R/HNRNPD/AURKA/CCND1/TYMS/PKD2/TWSG1/HK2/UMPS/NKX2-3/ORAI1/CFLAR/PLXND1/SRP54/FPGS/PML/IL6/SFRP1/LAMA5/FGF7/PITX1/MAP2K1/PRMT5/CREB1/CAD/HMGCS1/RELA/WNT4/HOXB13/TNFSF11/TGFBR1/HLX/SALL1/WNT2/GPX1/FSTL3/TGFB3/MDK/CDK5RAP3/FBXW7/ETS1/ARID5B/AKT1/LMO4/POLB
GOBP_EPITHELIAL_CELL_DIFFERENTIATION_INVOLVED_IN_KIDNEY_DEVELOPMENT
GOBP_EPITHELIAL_CELL_DIFFERENTIATION_INVOLVED_IN_KIDNEY_DEVELOPMENT
GOBP_EPITHELIAL_CELL_DIFFERENTIATION_INVOLVED_IN_KIDNEY_DEVELOPMENT 48
0.544118337 1.579265957 0.009302326 0.067389319 0.053332881 2333
tags=25%, list=14%, signal=22%
GREM1/MMP9/BASP1/PODXL/STAT1/ACTA2/WWTR1/LAMB2/LIF/JAG1/GPR4/SALL1
GOBP_MESENCHYME_MORPHOGENESIS GOBP_MESENCHYME_MORPHOGENESIS
GOBP_MESENCHYME_MORPHOGENESIS 48 0.541446618 1.571511476 0.009302326
0.067389319 0.053332881 1582 tags=25%, list=9%, signal=23%
WNT5A/ROBO1/ENG/ACTG2/TWIST1/TGFBR2/FOXF1/ACTA2/SNAI2/LEF1/DCHS1/ACVR1
GOBP_T_CELL_SELECTION GOBP_T_CELL_SELECTION GOBP_T_CELL_SELECTION 48
0.539384862 1.565527374 0.009302326 0.067389319 0.053332881 2523
tags=38%, list=15%, signal=32%
CD74/IRF4/PTPRC/CCR7/SHH/STAT3/IL12RB1/DOCK2/ITPKB/IL23A/IL6/CTSL/BATF/SLAMF6/CD4/LY9/CD3D/SYK
GOBP_RECEPTOR_SIGNALING_PATHWAY_VIA_STAT
GOBP_RECEPTOR_SIGNALING_PATHWAY_VIA_STAT
GOBP_RECEPTOR_SIGNALING_PATHWAY_VIA_STAT 156 0.426145343 1.467779027
0.009308511 0.067389319 0.053332881 1968 tags=21%, list=12%, signal=19%
AGT/CAV1/IL7R/LYN/CCR2/HCLS1/JAK3/PTPRC/F2R/SOCS3/PARP9/KIT/FYN/JAK2/STAT1/CCL2/CD300A/IL10RA/CD40/PARP14/STAT4/STAT3/IL6ST/AKR1B1/CSF1R/IFNAR2/PKD2/LIF/IL23A/IL6/TSLP/IFNG/STAT2
GOBP_DENDRITIC_CELL_DIFFERENTIATION GOBP_DENDRITIC_CELL_DIFFERENTIATION
GOBP_DENDRITIC_CELL_DIFFERENTIATION 44 0.555272253 1.590318401 0.009360374
0.067569782 0.053475702 4530 tags=50%, list=27%, signal=37%
LYN/IRF4/TGFBR2/CCR7/FCGR2B/CEBPB/LILRB2/CCL19/BATF2/AXL/LILRB1/BATF3/BATF/BLK/SPI1/TRPM2/HMGB1/ITGB8/IL4/DCSTAMP/LTBR/NOTCH2
GOBP_NEGATIVE_REGULATION_OF_LYMPHOCYTE_MEDIATED_IMMUNITY
GOBP_NEGATIVE_REGULATION_OF_LYMPHOCYTE_MEDIATED_IMMUNITY

GOBP_NEGATIVE_REGULATION_OF_LYMPHOCYTE_MEDIATED_IMMUNITY 44 0.564061875 1.615492173 0.009360374 0.067569782 0.053475702 2123 tags=27%, list=13%, signal=24% C4BPB/C4BPA/SERPINB9/IL7R/PTPRC/SERPINB4/FCGR2B/BCL6/LILRB4/LILRB1/HAVCR2/NOD2

GOBP_NON_CANONICAL_WNT_SIGNALING_PATHWAY GOBP_NON_CANONICAL_WNT_SIGNALING_PATHWAY GOBP_NON_CANONICAL_WNT_SIGNALING_PATHWAY 148 0.427770194 1.462478699 0.009395973 0.067729306 0.053601952 4308 tags=45%, list=25%, signal=34% CTHRC1/WNT5A/RSPO3/PSME4/PSMB9/AGO2/DACT1/RNF213/LEF1/DAB2/PSMD14/CSNK1E/PSMD12/PSMC2/PLCB1/DKK1/PSMB2/PSMA3/PSMD1/GPC6/PSMA5/AGO3/SFRP1/PSMD6/CELSR1/PSMB5/PSMC4/VANGL1/FZD4/WNT4/PPP3CA/SFRP2/PSMB1/PSMA1/PPP3R1/PSME3/PLEKHA4/PSMA7/PSMB8/PSMC5/ARRB2/DVL1/SMURF1/PSMB10/PSMB6/PSMA4/AP2B1/PSMD13/PSME1/PRICKLE1/PSMC1/SMURF2/DVL3/PSMB4/ARHGEF19/AP2S1/PSMB3/PSMD2/PSMC6/PSMD7/PSMA2/ANKRD6/PSMD4/AP2A2/PSMD9/TIAM1/FZD9

GOBP_RRNA_TRANSCRIPTION GOBP_RRNA_TRANSCRIPTION GOBP_RRNA_TRANSCRIPTION 31 0.595144073 1.610076236 0.009448819 0.068012517 0.053826089 4359 tags=58%, list=26%, signal=43% CAVIN1/GTF3A/NIFK/ANG/NCL/POLR1B/MACROH2A2/PWP1/GTF3C6/PIH1D1/DDX11/TCOF1/BRF1/TAF1B/NOL11/SMARCB1/NPM3/SMARCA4

GOBP_CELLULAR_CARBOHYDRATE_BIOSYNTHETIC_PROCESS GOBP_CELLULAR_CARBOHYDRATE_BIOSYNTHETIC_PROCESS GOBP_CELLULAR_CARBOHYDRATE_BIOSYNTHETIC_PROCESS 81 -0.424767143 -1.504474114 0.00952381 0.068219521 0.053989915 2079 tags=26%, list=12%, signal=23% HAS2/IRS2/B4GALT5/INPP5K/PPP1CB/B3GNT2/PCK2/IMPA2/PGM1/PER2/B3GALT5/IMPA1/AGL/INSR/DYRK2/UGP2/HAS3/B3GNT8/ENPP1/PPP1R3B/PCK1

GOBP_MORPHOGENESIS_OF_A_POLARIZED_EPITHELIUM GOBP_MORPHOGENESIS_OF_A_POLARIZED_EPITHELIUM GOBP_MORPHOGENESIS_OF_A_POLARIZED_EPITHELIUM 140 0.435327193 1.484136022 0.009536785 0.068219521 0.053989915 4282 tags=48%, list=25%, signal=36% CTHRC1/WNT5A/MSN/LAMA3/RSPO3/PSME4/PSMB9/FOXF1/DACT1/DAB2/PSMD14/PSMD12/PSMC2/DKK1/PSMB2/PSMA3/PSMD1/GPC6/PSMA5/AHI1/SFRP1/LAMA5/PSMD6/CELSR1/PSMB5/PSMC4/VANGL1/FZD4/SFRP2/PSMB1/PSMA1/FOXF2/PSME3/PLEKHA4/PSMA7/PSMB8/PSMC5/ARRB2/DLG5/DVL1/SMURF1/PSMB10/PSMB6/PSMA4/AP2B1/PSMD13/PSME1/PRICKLE1/SAPCD2/PSMC1/SMURF2/DVL3/PSMB4/ARHGEF19/AP2S1/PSMB3/PSMD2/TCF15/PSMC6/PSMD7/PSMA2/ANKRD6/PSMD4/AP2A2/PSMD9/IFT20/TIAM1

GOBP_MEMBRANE_REPOLARIZATION_DURING_ACTION_POTENTIAL GOBP_MEMBRANE_REPOLARIZATION_DURING_ACTION_POTENTIAL GOBP_MEMBRANE_REPOLARIZATION_DURING_ACTION_POTENTIAL 24 0.629284034 1.615583004 0.009538951 0.068219521 0.053989915 1343 tags=21%, list=8%, signal=19% KCND3/CAV1/KCNE3/FLNA/KCNJ8

GOBP_POSITIVE_REGULATION_OF_MONOCYTE_CHEMOTAXIS GOBP_POSITIVE_REGULATION_OF_MONOCYTE_CHEMOTAXIS

GOBP_POSITIVE_REGULATION_OF_MONOCYTE_CHEMOTAXIS 20 0.676575371 1.681853889 0.009569378 0.068219521 0.053989915 1898 tags=50%, list=11%, signal=44% CXCL10/SERPINE1/CCR2/PLA2G7/S100A7/CCR1/ANO6/CREB3/AIF1/FPR2
GOBP_POSITIVE_REGULATION_OF_EXTRACELLULAR_MATRIX_ORGANIZATION GOBP_POSITIVE_REGULATION_OF_EXTRACELLULAR_MATRIX_ORGANIZATION GOBP_POSITIVE_REGULATION_OF_EXTRACELLULAR_MATRIX_ORGANIZATION 22 0.645540886 1.634696824 0.009615385 0.068219521 0.053989915 2478 tags=50%, list=15%, signal=43% RGCC/TGFB1/PDPN/EFEMP2/DDR2/EMILIN1/FSCN1/CFLAR/IL6/PHLDB2/COLGALT1
GOBP_REGULATION_OF_MYELOID_LEUKOCYTE_DIFFERENTIATION GOBP_REGULATION_OF_MYELOID_LEUKOCYTE_DIFFERENTIATION GOBP_REGULATION_OF_MYELOID_LEUKOCYTE_DIFFERENTIATION 109 0.474399742 1.562225898 0.009628611 0.068219521 0.053989915 2912 tags=39%, list=17%, signal=32% LYN/FBN1/HCLS1/CD74/TGFB1/PF4/EVI2B/INHBA/NME1/CCR1/GPR137B/CEBPB/PIAS3/MAFB/TYROBP/RASSF2/LEF1/LTF/CLDN18/TESC/SLC9B2/TFE3/LIF/IL23A/CASP8/LILRB4/SFRP1/LILRB1/IFNG/LILRB3/CREB1/TMEM64/TNFSF11/INPP5D/IRF7/CD4/HAX1/FSTL3/FBXW7/PLA2G3/FES/MITF
GOBP_REGULATION_OF_MEMBRANE_PROTEIN_ECTODOMAIN_PROTEOLYSIS GOBP_REGULATION_OF_MEMBRANE_PROTEIN_ECTODOMAIN_PROTEOLYSIS GOBP_REGULATION_OF_MEMBRANE_PROTEIN_ECTODOMAIN_PROTEOLYSIS 23 0.636672555 1.623959212 0.009630819 0.068219521 0.053989915 2843 tags=52%, list=17%, signal=43% TIMP1/IL1B/ADAM9/TNFRSF1B/ADAM8/IFNG/TIMP2/NRDC/FURIN/TIMP3/APOE/ROCK1
GOBP_POSITIVE_REGULATION_OF_MITOTIC_CELL_CYCLE GOBP_POSITIVE_REGULATION_OF_MITOTIC_CELL_CYCLE GOBP_POSITIVE_REGULATION_OF_MITOTIC_CELL_CYCLE 106 0.475022179 1.557954658 0.009641873 0.068219521 0.053989915 3742 tags=39%, list=22%, signal=30% ANXA1/ADAMTS1/RGCC/CDC25B/HYAL1/CCNB1/SMOC2/TTL/ASNS/CDC25A/CDCA5/CDC6/CDK1/PLCB1/SPHK1/CCND1/UBE2C/LSM10/DLGAP5/CDC27/RCC2/CDC7/AIF1/RDX/EIF4G1/CDK4/RAD51C/UBE2E2/CDT1/AKT1/STAT5B/PTPN11/ANAPC7/FBXO5/KLHL18/DTL/CCND3/MDM2/DUSP3/ADAM17/CCND2
GOBP_REGULATION_OF_STEM_CELL_DIFFERENTIATION GOBP_REGULATION_OF_STEM_CELL_DIFFERENTIATION GOBP_REGULATION_OF_STEM_CELL_DIFFERENTIATION 106 0.46899584 1.538189764 0.009641873 0.068219521 0.053989915 4252 tags=47%, list=25%, signal=36% CBFB/PSME4/PSMB9/PDGFRA/LMO2/PSMD14/PSMD12/PSMC2/TACSTD2/PSMB2/PSMA3/METTL3/PSMD1/TCF3/PSMA5/PSMD6/JAG1/PSMB5/PSMC4/PUS7/TEAD2/PSMB1/PSMA1/PSME3/NSUN2/PSMA7/PSMB8/PSMC5/PWP1/PSMB10/PSMB6/PSMA4/LDB1/PSMD13/PSME1/PRICKLE1/PSMC1/PSMB4/SOX9/PSMB3/PSMD2/PSMC6/PSMD7/DHX36/PSMA2/PSMD4/TGFB2/HOXB4/GATA2/PSMD9
GOBP_ESTROGEN_BIOSYNTHETIC_PROCESS GOBP_ESTROGEN_BIOSYNTHETIC_PROCESS GOBP_ESTROGEN_BIOSYNTHETIC_PROCESS 12 -0.712066696 -1.70034966 0.00968523 0.068219521 0.053989915 1007 tags=33%, list=6%, signal=31%

HSD3B1/HSD17B11/HSD17B2/DHRS11
GOBP_REGULATION_OF_LEUKOCYTE_CHEMOTAXIS
GOBP_REGULATION_OF_LEUKOCYTE_CHEMOTAXIS
GOBP_REGULATION_OF_LEUKOCYTE_CHEMOTAXIS 110 0.470674696 1.549005907
0.009695291 0.068219521 0.053989915 2508 tags=37%, list=15%, signal=32%
GREM1/CXCL13/CXCL8/CXCL10/WNT5A/LYN/SERPINE1/CCR2/CD74/PLA2G7/RAC2/SLAMF8/S100A7/CCR1/C5AR1/CCR7/CCL4/CCL2/VEGFC/CALR/CCR6/ANO6/C3AR1/RIPOR2/CSF1R/GPSM3/CCL19/JAM3/MIF/CXCL14/IL23A/IL6/CREB3/AIF1/FPR2/NCKAP1L/NOD2/SWAP70/CMKLR1/MDK/CCL7
GOBP_COENZYME_A_BIOSYNTHETIC_PROCESS GOBP_COENZYME_A_BIOSYNTHETIC_PROCESS
GOBP_COENZYME_A_BIOSYNTHETIC_PROCESS 10 -0.745639277 -1.670316223
0.009708738 0.068219521 0.053989915 535 tags=30%, list=3%, signal=29%
PANK3/ACAT1/PANK1
GOBP_DIET_INDUCED_THERMOGENESIS GOBP_DIET_INDUCED_THERMOGENESIS
GOBP_DIET_INDUCED_THERMOGENESIS 10 -0.748184641 -1.676018125 0.009708738
0.068219521 0.053989915 358 tags=40%, list=2%, signal=39%
OMA1/ADRB1/SORL1/APPL2
GOBP_DRUG_TRANSMEMBRANE_TRANSPORT GOBP_DRUG_TRANSMEMBRANE_TRANSPORT
GOBP_DRUG_TRANSMEMBRANE_TRANSPORT 10 -0.736631197 -1.650137105
0.009708738 0.068219521 0.053989915 1732 tags=40%, list=10%, signal=36%
ABCC3/ABCC6/ATP8B1/ABCB11
GOBP_MITOCHONDRIAL_ELECTRON_TRANSPORT_UBIQUINOL_TO_CYTOCHROME_C
GOBP_MITOCHONDRIAL_ELECTRON_TRANSPORT_UBIQUINOL_TO_CYTOCHROME_C
GOBP_MITOCHONDRIAL_ELECTRON_TRANSPORT_UBIQUINOL_TO_CYTOCHROME_C 10
-0.74881311 -1.677425967 0.009708738 0.068219521 0.053989915 3713
tags=80%, list=22%, signal=62%
UQCRQ/UQCR11/UQCR10/CYC1/UQCRFS1/UQCRC2/CYCS/UQCRC1
GOBP_RECEPTOR_GUANYLYL_CYCLASE_SIGNALING_PATHWAY
GOBP_RECEPTOR_GUANYLYL_CYCLASE_SIGNALING_PATHWAY
GOBP_RECEPTOR_GUANYLYL_CYCLASE_SIGNALING_PATHWAY 10 -0.736848183
-1.650623179 0.009708738 0.068219521 0.053989915 972 tags=20%, list=6%, signal=19% GUCY2C/PDZD3
GOBP_REGULATION_OF_CELL_PROJECTION_SIZE
GOBP_REGULATION_OF_CELL_PROJECTION_SIZE
GOBP_REGULATION_OF_CELL_PROJECTION_SIZE 10 -0.743789336 -1.666172144
0.009708738 0.068219521 0.053989915 3062 tags=60%, list=18%, signal=49%
TWF2/USH1C/PLS1/CDHR2/VIL1/CDHR5
GOBP_NEGATIVE_REGULATION_OF_ION_TRANSPORT
GOBP_NEGATIVE_REGULATION_OF_ION_TRANSPORT
GOBP_NEGATIVE_REGULATION_OF_ION_TRANSPORT 278 0.38042465 1.394830583
0.00973236 0.068289861 0.054045583 2846 tags=26%, list=17%, signal=22%
ANXA1/IL1B/DERL3/MMP9/CAV1/KCNE3/SERPINE2/ADA/PEA15/CD74/GEM/STC1/BAG3/GRB10/F2R/PIM3/PLN/UCP2/TWIST1/VSNL1/SHH/LILRB2/SESTD1/TRIM27/ICAM1/RHBDF2/IDH2/VIP/PCSK9/RGS2/ERLEC1/LMAN1/GNB5/IRS1/RAB23/UBE2J1/SEMG1/CLIC2/APOC1/INSIG1/FFAR4/

RGS4/PRKCB/ITGB1BP1/GSTO1/BARD1/SPINK1/PKD2/CD200/ITGAV/RANGAP1/OAZ3/NEDD4/SFRP1/LILRB1/PKIA/RHBDF1/APOD/DERL2/PPP3CA/SYT11/HECW2/INHBB/LRRK2/FERMT1/ATF4/AKT1/PARK7/ATP7A/ABCG5/CRY1/APOE/PPM1F

GOBP_TELOMERASE_RNA_LOCALIZATION GOBP_TELOMERASE_RNA_LOCALIZATION GOBP_TELOMERASE_RNA_LOCALIZATION 17 0.679301803 1.613222179 0.009756098 0.068360812 0.054101735 3991 tags=71%, list=24%, signal=54% RUVBL1/CCT3/CCT8/CCT5/CCT2/CCT6A/CCT4/CCT7/NHP2/RUVBL2/EXOSC10/NOP10

GOBP_NEGATIVE_REGULATION_OF_KINASE_ACTIVITY GOBP_NEGATIVE_REGULATION_OF_KINASE_ACTIVITY GOBP_NEGATIVE_REGULATION_OF_KINASE_ACTIVITY 241 0.389142989 1.408073783 0.009803922 0.068600104 0.054291114 3449 tags=34%, list=20%, signal=28% WARS1/IL1B/AGT/TRIB2/CAV1/SERPINB3/LYN/HEG1/LAX1/SH3BP5/PTPRC/PRKAR2B/SOCS3/DNAJC3/DUSP4/DUSP14/THY1/GMFG/DUSP6/TRIM27/DUSP7/LATS2/CD300A/HHEX/HYAL2/MYOCD/RHOH/RGS2/DNAJA1/WWTR1/SPRED1/RGS4/NUP62/TESC/ITGB1BP1/DUSP10/PLK1/PIP4K2A/GPRC5A/IBTK/CORO1C/DUSP22/ITPRIP/CDKN2C/AJUBA/LILRB4/SFRP1/PKIA/IFNG/CDKN1C/CHORDC1/RUBCN/SFRP2/PARVA/PTPN1/FOXA2/ADAR/UCHL1/CDK5RAP3/AKT1/RGS3/PARK7/NPM1/MLLT1/PRKAR1A/DVL1/APOE/PPM1F/SOCS4/IRAK3/SPRY1/GSTP1/NR2F2/RASIP1/ERRFI1/CDK5RAP1/NF2/YWHAG/AIDA/PKN1/TARBP2/IPO7/EPHA1

GOBP_CELL_CYCLE_G1_S_PHASE_TRANSITION GOBP_CELL_CYCLE_G1_S_PHASE_TRANSITION GOBP_CELL_CYCLE_G1_S_PHASE_TRANSITION 258 0.393866332 1.432958118 0.00982801 0.068672876 0.054348707 2888 tags=30%, list=17%, signal=25% ANXA1/RRM2/ADAMTS1/RGCC/HYAL1/PIM2/FAM83D/INHBA/TCIM/CCNB1/SLFN11/PPAT/BCAT1/CCL2/LATS2/DACT1/CDC25A/MCM6/CDC45/E2F6/KIF14/CDC6/EZH2/CDK1/PLCB1/MUC1/MCM10/AURKA/CCND1/TYMS/PKD2/BID/LSM10/PRMT1/CDKN3/E2F3/ACVR1/CDKN2C/PML/FHL1/TNKS1BP1/GSPT1/ZPR1/CDC7/CDK2/CNOT9/ATP2B4/AIF1/GPNMB/RDX/POLE2/ORC6/EIF4G1/DBF4/PLK2/JADE1/CCNH/PPP3CA/MCM3/ARID3A/MCM5/CDK4/PAF1/CRLF3/UBE2E2/PSME3/CDT1/FBXW7/AKT1/FAM107A/CNOT6L/MAP3K11/MCM7/CUL1/TRIAP1/TAF10/CCNA2/NACC2

GOBP_CELLULAR_RESPONSE_TO_PURINE_CONTAINING_COMPOUND GOBP_CELLULAR_RESPONSE_TO_PURINE_CONTAINING_COMPOUND GOBP_CELLULAR_RESPONSE_TO_PURINE_CONTAINING_COMPOUND 13 -0.736778033 -1.793769356 0.009950249 0.069333886 0.05487184 146 tags=31%, list=1%, signal=31% GSTM2/TMEM38B/P2RY1/PPARGC1A

GOBP_REGULATION_OF_EPITHELIAL_CELL_MIGRATION GOBP_REGULATION_OF_EPITHELIAL_CELL_MIGRATION GOBP_REGULATION_OF_EPITHELIAL_CELL_MIGRATION 223 0.393392246 1.411777277 0.009950249 0.069333886 0.05487184 3086 tags=33%, list=18%, signal=27% ANXA1/CXCL13/AGT/SPARC/WNT5A/ADAMTS9/MMP9/RGCC/HYAL1/ITGA2/CTSH/TGFB1/STC1/ANXA3/HIF1A/PTGS2/ENPP2/TGFBR2/SMOC2/PTPRM/DCN/MAP4K4/SRPX2/MET/PLCG2/CD40/SERPINF1/MMRN2/VEGFC/KDR/TEK/CALR/CCR6/PIK3CG/ADGRA2/SPRED1/TACSTD2/FGFR1/MCC/ADAM9/PIK3CD/ITGB1BP1/DUSP10/NRP1/HDAC7/MACF1/CORO1C/AKT3/FGFBP1/FGF7/ATP2B4/IFNG/VASH1/NUS1/PLK2/STRAP/SEMA4A/RHOJ/GLUL/FBXW7/ETS1/ANGPT2/AKT1/JCAD/APOE/PPM1F/FGF2/EVL/GPI/EMP2/ITGA3/STAT5A/NR2F2/PRCP

GOBP_NEGATIVE_REGULATION_OF_TYPE_I_INTERFERON_MEDIATED_SIGNALING_PATHWAY GOBP_NEGATIVE_REGULATION_OF_TYPE_I_INTERFERON_MEDIATED_SIGNALING_PATHWAY

GOBP_NEGATIVE_REGULATION_OF_TYPE_I_INTERFERON_MEDIATED_SIGNALING_PATHWAY 13 0.71558813 1.596695439 0.01 0.069583911 0.055069713 1348 tags=31%, list=8%, signal=28% MMP12/NLRC5/SAMHD1/METTL3
GOBP_CALCIUM_ION_IMPORT GOBP_CALCIUM_ION_IMPORT GOBP_CALCIUM_ION_IMPORT 77 0.494441473 1.539175681 0.010086455 0.070088291 0.055468887 1989 tags=25%, list=12%, signal=22% MS4A1/STC1/MCUB/RAMP3/HOMER1/PLN/FYN/TRIM27/CCL2/PDGFRB/SEMG1/SPINK1/FCRL3/PKD2/PRNP/TRPV2/ORAI1/ATP2B4/ATP2A2
GOBP_REGULATION_OF_BIOLOGICAL_PROCESS_INVOLVED_IN_SYMBIOTIC_INTERACTION GOBP_REGULATION_OF_BIOLOGICAL_PROCESS_INVOLVED_IN_SYMBIOTIC_INTERACTION GOBP_REGULATION_OF_BIOLOGICAL_PROCESS_INVOLVED_IN_SYMBIOTIC_INTERACTION 186 0.404180623 1.425761079 0.010152284 0.070448147 0.055753682 4116 tags=38%, list=24%, signal=29% CXCL6/CXCL8/LAMP3/IFITM2/CAV1/IFITM3/ISG20/TRIM22/CD74/IFI16/IFITM1/SLPI/LGALS1/OAS2/FCN1/BST2/CXCR4/STAT1/CAV2/TRIM27/KPNA2/APOBEC3G/FCN3/P4HB/LTF/TRIM15/KRT6A/OAS3/LY6E/TMEM39A/ITGAV/LARP1/PML/AXL/MX1/POLR2D/SHFL/TMEM39B/HACD3/CD4/KPNA6/TRIM28/ADAR/TYRO3/FUCA2/TRIM5/TRIM21/SUPT5H/TRIM8/POLR2L/PLSCR1/RAD23A/RESF1/VPS4A/DDB1/TARBP2/CD209/TRIM26/NECTIN2/MID2/PPIH/POLR2A/PARP10/NELFCD/TOP2A/CCNT1/ZNF502/FAM111A/BANF1/PPID/EXOC7
GOBP_RIBOSOMAL_SMALL_SUBUNIT_BIOGENESIS GOBP_RIBOSOMAL_SMALL_SUBUNIT_BIOGENESIS GOBP_RIBOSOMAL_SMALL_SUBUNIT_BIOGENESIS 71 0.498646958 1.533634825 0.010309278 0.071438742 0.056537653 3721 tags=39%, list=22%, signal=31% UTP4/RRP36/TSR1/RRS1/RRP7A/BYSL/NAT10/UTP6/RPP40/NOP14/DCAF13/RIOK1/TBL3/HEATR1/LTV1/NPM1/BMS1/UTP3/SRFBP1/LSM6/NOB1/NOL10/XRCC5/WDR46/WDR3/EMG1/UTP25/NOP9
GOBP_MITOCHONDRIAL_ELECTRON_TRANSPORT_CYTOCHROME_C_TO_OXYGEN GOBP_MITOCHONDRIAL_ELECTRON_TRANSPORT_CYTOCHROME_C_TO_OXYGEN GOBP_MITOCHONDRIAL_ELECTRON_TRANSPORT_CYTOCHROME_C_TO_OXYGEN 17 -0.681200531 -1.755586304 0.010335917 0.071524548 0.056605561 4265 tags=76%, list=25%, signal=57% COX7C/COX6B1/AFG1L/COX10/COX7B/COX8A/COX6A1/COX4I1/CYCS/COX5A/COX6C/COX5B/COX15
GOBP_SOMATIC_RECOMBINATION_OF_IMMUNOGLOBULIN_GENE_SEGMENTS GOBP_SOMATIC_RECOMBINATION_OF_IMMUNOGLOBULIN_GENE_SEGMENTS GOBP_SOMATIC_RECOMBINATION_OF_IMMUNOGLOBULIN_GENE_SEGMENTS 53 0.539038203 1.597035543 0.010385757 0.071770442 0.056800166 3272 tags=40%, list=19%, signal=32% TGFB1/IL27RA/PTPRC/TNFSF13/MAD2L2/CD40/BCL6/CCR6/TCF3/EXOSC3/EXO1/MSH6/MLH1/BATF/NBN/SWAP70/THOC1/POLB/RNF8/HSPD1/SLC15A4
GOBP_POSITIVE_REGULATION_OF_PROTEIN_KINASE_B_SIGNALING GOBP_POSITIVE_REGULATION_OF_PROTEIN_KINASE_B_SIGNALING GOBP_POSITIVE_REGULATION_OF_PROTEIN_KINASE_B_SIGNALING 160 0.413965136 1.429277989 0.01061008 0.073202405 0.057933441 3027 tags=34%, list=18%,

signal=28%

CHI3L1/IGFBP5/FERMT2/ENG/F3/HCLS1/TGFB1/PIK3AP1/RAC2/MYDGF/RAMP3/KIT/PDGFRA/CCR7/CD19/FYN/CD86/PDGFRB/MET/OSBPL8/IRS1/MST1R/TEK/MTDH/TPBG/VAV1/PIK3CG/LCK/FGFR1/CCL19/PIK3CD/ITGB1BP1/HPSE/PIK3R5/ADAM8/RHOG/AXL/STK3/FGF7/TNFSF11/TGFBR1/HAX1/GPX1/HSP90AB1/PARK7/HIP1/IL18/TSPYL5/C1QTNF1/ARRB2/FGF2/HSP90AA1/PTPN11/RASD2

GOBP_NEGATIVE_REGULATION_OF_ADAPTIVE_IMMUNE_RESPONSE

GOBP_NEGATIVE_REGULATION_OF_ADAPTIVE_IMMUNE_RESPONSE

GOBP_NEGATIVE_REGULATION_OF_ADAPTIVE_IMMUNE_RESPONSE 50 0.534148223 1.564010694 0.010622155 0.073202405 0.057933441 2123 tags=30%, list=13%, signal=26%

C4BPB/C4BPA/IL7R/IL33/JAK3/IL27RA/PTPRC/SAMSN1/FCGR2B/TRIM27/BCL6/LILRB4/LILRB1/HAVCR2/NOD2

GOBP_REGULATION_OF_COLLAGEN_METABOLIC_PROCESS

GOBP_REGULATION_OF_COLLAGEN_METABOLIC_PROCESS

GOBP_REGULATION_OF_COLLAGEN_METABOLIC_PROCESS39 0.556018717 1.563234507 0.010769231 0.074114171 0.058655026 2826 tags=46%, list=17%, signal=39%

RGCC/ITGA2/ENG/SERPINB7/TGFB1/F2R/VIM/FAP/PDGFRB/CREB3L1/EMILIN1/IL6/WNT4/MFAP4/SERPINF2/TGFB3/ARRB2/SUCO

GOBP_REGULATION_OF_T_CELL_MIGRATION GOBP_REGULATION_OF_T_CELL_MIGRATION

GOBP_REGULATION_OF_T_CELL_MIGRATION 42 0.567583597 1.617436384 0.010785824 0.074126686 0.058664931 2039 tags=38%, list=12%, signal=34%

CXCL13/CXCL10/WNT5A/CCR2/IL27RA/ECM1/CCL20/S100A7/CCR6/LRCH1/RIPOR2/CD200/ADAM8/AIF1/APOD/SELENOK

GOBP_POSITIVE_REGULATION_OF_EPITHELIAL_CELL_MIGRATION

GOBP_POSITIVE_REGULATION_OF_EPITHELIAL_CELL_MIGRATION

GOBP_POSITIVE_REGULATION_OF_EPITHELIAL_CELL_MIGRATION 143 0.431399535 1.475186988 0.010810811 0.074139935 0.058675416 1956 tags=27%, list=12%, signal=24%

ANXA1/AGT/SPARC/WNT5A/MMP9/HYAL1/ITGA2/CTSH/TGFB1/ANXA3/HIF1A/PTGS2/ENPP2/TGFBR2/SMOC2/MAP4K4/SRPX2/MET/PLCG2/CD40/VEGFC/KDR/TEK/CALR/CCR6/PIK3CG/ADGRA2/FGFR1/ADAM9/PIK3CD/ITGB1BP1/NRP1/HDAC7/AKT3/FGFBP1/FGF7/IFNG/NUS1

GOBP_ERK1_AND_ERK2_CASCADE GOBP_ERK1_AND_ERK2_CASCADE

GOBP_ERK1_AND_ERK2_CASCADE 283 0.37788995 1.390226966 0.010817308 0.074139935 0.058675416 2549 tags=26%, list=15%, signal=22%

CHI3L1/CCL11/PLA2G2A/CCL18/IL1B/GBP1/FERMT2/LYN/APIP/CTSH/CCN1/CD44/CD74/TGFB1/FAM83D/PTPRC/CCL20/F2R/S100A7/RIPK2/CCR1/RAMP3/FN1/NQO2/DUSP4/C5AR1/PDGFRA/CCR7/CCL4/DUSP6/GNAI2/ICAM1/CCL2/PDGFRB/CCL24/SIRPA/KDR/CCL22/EMILIN1/TEK/TPBG/FFAR4/SPRED1/CSF1R/GPR183/CCL19/ITGB1BP1/DUSP10/NRP1/AKAP12/MIF/CFLAR/ITGAV/LIF/DENND2B/MAP2K1/PRMT5/GPNMB/FPR2/HAVCR2/TNFSF11/NOD2/PDGFC/SERPINF2/NPNT/PRXL2C/PTPN1/CD4/RASGRP1/TIMP3/CCL7/SYK/FBXW7

GOBP_TRANSITION_METAL_ION_HOMEOSTASIS

GOBP_TRANSITION_METAL_ION_HOMEOSTASIS

GOBP_TRANSITION_METAL_ION_HOMEOSTASIS 130 -0.378208991 -1.443299112

0.010869565 0.07436984 0.058857366 1761 tags=25%, list=10%, signal=22%
ABCB7/NCOA4/IREB2/MT3/SLC39A14/STEAP3/STEAP2/NEO1/XIAP/CCDC115/FTH1/MT2A/SMAD4/BDH2/SLC30A1/MT1HL1/SRI/SLC30A4/MT1E/MT1X/MT1H/SLC39A5/APP/SLC46A1/MT1G/FLVCR1/MT1F/HMOX1/SLC1A1/MT1M/SLC30A10/ABCG2

GOBP_HETEROPHILIC_CELL_CELL_ADHESION_VIA_PLASMA_MEMBRANE_CELL_ADHESION_MOLECULES
GOBP_HETEROPHILIC_CELL_CELL_ADHESION_VIA_PLASMA_MEMBRANE_CELL_ADHESION_MOLECULES
GOBP_HETEROPHILIC_CELL_CELL_ADHESION_VIA_PLASMA_MEMBRANE_CELL_ADHESION_MOLECULES 45 0.560792175 1.61211842 0.010903427 0.07436984 0.058857366 739
tags=18%, list=4%, signal=17% REG3A/CADM1/SELP/ALCAM/ITGA5/VCAM1/SELE/ICAM1

GOBP_ICOSANOID_METABOLIC_PROCESS GOBP_ICOSANOID_METABOLIC_PROCESS
GOBP_ICOSANOID_METABOLIC_PROCESS 109 -0.389280908 -1.456038284 0.010909091
0.07436984 0.058857366 2165 tags=29%, list=13%, signal=26%
AKR1C1/GSTA1/GGT7/CYP4F3/GSTM1/MGST3/FAAH/GPX4/PLA2G4C/CYP2J2/GGT6/HPGDS/AKR1C2/GGTLC1/CYP1A1/SIRT1/EDN2/PTGR1/AKR1C3/PTGR2/ZADH2/EPHX1/CYP4F12/MGLL/CYP4F2/CES2/EPHX2/CYP2S1/ACOX1/HPGD/CYP2B6/EDN1

GOBP_MULTIVESICULAR_BODY_ORGANIZATION GOBP_MULTIVESICULAR_BODY_ORGANIZATION
GOBP_MULTIVESICULAR_BODY_ORGANIZATION 29 -0.579298532 -1.663638209
0.010928962 0.07436984 0.058857366 2554 tags=41%, list=15%, signal=35%
IST1/STAM2/VPS36/VPS28/CHMP2A/CHMP4C/VPS4B/VTA1/PDCD6IP/CHMP5/CHMP1B/CHMP4B

GOBP_REGULATORY_T_CELL_DIFFERENTIATION GOBP_REGULATORY_T_CELL_DIFFERENTIATION
GOBP_REGULATORY_T_CELL_DIFFERENTIATION 32 0.592603845 1.618812432
0.0109375 0.07436984 0.058857366 3527 tags=53%, list=21%, signal=42%
HLA-DRA/CBFB/LILRB2/BCL6/IRF1/DUSP10/FANCD2/LAG3/LILRB4/IFNG/MDK/TNFRSF18/CTLA4/IL2RA/PLA2G2D/CR1/NFATC2

GOBP_MITOTIC_NUCLEAR_DIVISION GOBP_MITOTIC_NUCLEAR_DIVISION
GOBP_MITOTIC_NUCLEAR_DIVISION 286 0.377056368 1.38463828 0.010948905
0.07436984 0.058857366 4111 tags=38%, list=24%, signal=30%
IL1B/RGCC/LPIN1/TRIP13/CCNB1/MAD2L2/CAV2/ANLN/TUBG1/PDGFRB/BUB1/CDC20/PRC1/NCAPG/NDC80/FLNA/IL1A/NEK6/PPP2R2A/RRS1/CDCA5/TACC3/PTTG1/KIF14/CDC6/CDK1/MIS12/MKI67/KNSTRN/TENT4A/NUP62/SPHK1/CENPE/PRKCB/AURKB/AURKA/BUB1B/NUSAP1/PLK1/UBE2C/RAN/SMC4/DLGAP5/KIF23/CDC27/UBE2S/TPX2/VRK1/RANBP1/MZT1/ZWINT/PRMT5/NUDC/KPNB1/MYBL2/KIF2C/BCCIP/KIF11/PSRC1/HECW2/CDCA8/KIF4A/CDC26/CCSAP/PINX1/CHEK1/SPAG5/NCAPH/NCAPG2/CDT1/SPDL1/MAP9/NDE1/KLHL22/EML3/KNTC1/PPP1R9B/ARHGEF10/ANAPC7/LMNA/FBXO5/SGO2/RACGAP1/SMC2/TUBG2/EMD/KLHDC8B/VPS4A/NUF2/CCNB2/AAAS/DSCC1/KIF18A/REC8/NAA10/ANKLE2/KIF18B/ZNF207/CENPF/CTDNEP1/RCC1/BUB3/TTK/MAD2L1/DYNC1LI1/GOLGA2/CCDC8/BANF1/NEK2/CNEP1R1

GOBP_POSITIVE_REGULATION_OF_LYMPHOCYTE_MIGRATION
GOBP_POSITIVE_REGULATION_OF_LYMPHOCYTE_MIGRATION
GOBP_POSITIVE_REGULATION_OF_LYMPHOCYTE_MIGRATION 35 0.577315882
1.587929413 0.010954617 0.07436984 0.058857366 2688 tags=46%, list=16%, signal=39%

CXCL13/CXCL10/WNT5A/CCR2/CCL20/S100A7/MADCAM1/CCL4/JAM2/CXCL14/ADAM8/AIF1/SELENOK/CCL7/ITGA4/DOCK8

GOBP_FC_EPSILON_RECEPTOR_SIGNALING_PATHWAY

GOBP_FC_EPSILON_RECEPTOR_SIGNALING_PATHWAY

GOBP_FC_EPSILON_RECEPTOR_SIGNALING_PATHWAY 108 0.459851019 1.513690585 0.010973937 0.074400326 0.058881494 4036 tags=48%, list=24%, signal=37%

LYN/LCP2/FCER1G/PSME4/PSMB9/PLCG2/PSMD14/VAV1/PSMD12/PSMC2/PSMB2/PSMA3/PSMD1/BTK/ITK/PSMA5/PSMD6/PSMB5/PSMC4/RELA/PPP3CA/PSMB1/PSMA1/MALT1/PPP3R1/LAT2/PSME3/SYK/CUL1/PSMA7/PSMB8/PSMC5/NR4A3/PSMB10/NFATC3/PSMB6/PSMA4/LAT/PSMD13/SHC1/PSME1/CARD11/PSMC1/NFATC2/PSMB4/PSMB3/PSMD2/IKBKG/PSMC6/PSMD7/PSMA2/PSMD4

GOBP_INTRACELLULAR_RECEPTOR_SIGNALING_PATHWAY

GOBP_INTRACELLULAR_RECEPTOR_SIGNALING_PATHWAY

GOBP_INTRACELLULAR_RECEPTOR_SIGNALING_PATHWAY 256 -0.323406441 -1.340468911 0.010989011 0.074401981 0.058882803 2285 tags=25%, list=13%, signal=22%

WBP2/PUM2/UFL1/RNF6/TAB3/TAF7/CALCOCO1/SCGB2A1/EP300/DHRS3/ESRRA/NFKBIA/NR1D2/NR2F6/NCOA4/ARNT/NR4A2/FAM120B/TNFAIP3/LATS1/NR2C2/NR3C1/THRA/TRIM24/RXRA/BIRC2/TSPAN6/ARNTL/MAP2K6/YWHAH/PAK1/SIRT1/RNF14/NR1H3/KDM3A/PPARA/TAB2/AHRR/AKR1C3/XIAP/RNF135/PIM1/PDK3/LACC1/ZMIZ1/NR4A1/PPARD/INAVA/VDR/RIOK3/NR1I2/HNF4G/NR1H4/ERBIN/NR3C2/PPARGC1B/NR5A2/CITED2/DDX60/PLPP1/RNF125/PPARG/PADI2/THRB

GOBP_POSITIVE_REGULATION_OF_LEUKOCYTE_MEDIATED_IMMUNITY

GOBP_POSITIVE_REGULATION_OF_LEUKOCYTE_MEDIATED_IMMUNITY

GOBP_POSITIVE_REGULATION_OF_LEUKOCYTE_MEDIATED_IMMUNITY 114 0.45318852 1.500056555 0.011034483 0.074609164 0.059046771 3111 tags=35%, list=18%, signal=29%

IL1B/CADM1/NOS2/C3/TGFB1/AZGP1/HLA-DRA/PTPRC/TNFSF13/CLEC7A/IL1R1/MAD2L2/ITGB2/DDX21/SASH3/PTAFR/TYROBP/CD81/CD40/ITGAM/VAV1/IL12RB1/IL18R1/EXOSC3/BTK/LAG3/IL23A/IL6/MR1/MLH1/SLAMF6/NOD2/MALT1/RASGRP1/IL18/PVR/AP1G1/STAT5B/IL18RAP/HSPD1

GOBP_REGULATION_OF_FATTY_ACID_OXIDATION

GOBP_REGULATION_OF_FATTY_ACID_OXIDATION

GOBP_REGULATION_OF_FATTY_ACID_OXIDATION 32 -0.5666283 -1.657549415 0.011049724 0.074611661 0.059048747 2591 tags=44%, list=15%, signal=37%

TYSND1/ACACB/ETFBKMT/IRS2/PRKAG2/PPARA/PPARD/FABP1/CPT1A/ABCB11/PDK4/PPARG/PPARGC1A/APPL2

GOBP_REGULATION_OF_SYNCYTIUM_FORMATION_BY_PLASMA_MEMBRANE_FUSION

GOBP_REGULATION_OF_SYNCYTIUM_FORMATION_BY_PLASMA_MEMBRANE_FUSION

GOBP_REGULATION_OF_SYNCYTIUM_FORMATION_BY_PLASMA_MEMBRANE_FUSION 25 0.627188031 1.62723954 0.011146497 0.075161049 0.05948354 1720 tags=32%, list=10%, signal=29%

CXCL9/CXCL10/CD53/TYROBP/RIPOR2/ADAM9/CFLAR/EHD2

GOBP_CELLULAR_RESPONSE_TO_LIGHT_STIMULUS

GOBP_CELLULAR_RESPONSE_TO_LIGHT_STIMULUS
GOBP_CELLULAR_RESPONSE_TO_LIGHT_STIMULUS 126 0.434662633 1.459392809 0.011173184 0.075161049 0.05948354 3650 tags=31%, list=22%, signal=24% MMP1/MMP3/TIMP1/MMP9/MMP2/HYAL1/CARD16/POLD3/HYAL2/CDC25A/EIF2S1/MME/AURKB/METTL3/PBK/ATR/NMT1/NEDD4/PARP1/MFAP4/NOC2L/AQP1/CHEK1/RUVBL2/ATF4/NPM1/DDB2/COPS9/TRIAP1/CRY1/TMEM161A/HYAL3/DDB1/ACTR5/USP47/BAK1/GRK1/METAP2/RHBDD1
GOBP_ADENYLATE_CYCLASE_MODULATING_G_PROTEIN_COUPLED_RECEPTOR_SIGNALING_PATHWAY
GOBP_ADENYLATE_CYCLASE_MODULATING_G_PROTEIN_COUPLED_RECEPTOR_SIGNALING_PATHWAY
GOBP_ADENYLATE_CYCLASE_MODULATING_G_PROTEIN_COUPLED_RECEPTOR_SIGNALING_PATHWAY 222 0.392369395 1.407936283 0.011180124 0.075161049 0.05948354 1898 tags=16%, list=11%, signal=14% CXCL9/CXCL11/ADGRG6/CXCL10/ADGRL4/GRK5/GNA15/FPR1/EDNRA/PDE4B/PF4/S1PR3/MRAP2/RAMP3/ADGRE2/ADGRL2/PLN/GNA14/CALCRL/GNAI2/VIP/RGS2/FLNA/S1PR1/GPR176/ADGRE5/CHRM3/AKAP12/ADGRF1/PTGFR/GPR65/ADCY4/ATP2B4/PRMT5/FPR2
GOBP_KERATINOCYTE_DIFFERENTIATION GOBP_KERATINOCYTE_DIFFERENTIATION
GOBP_KERATINOCYTE_DIFFERENTIATION 209 0.394460817 1.407990503 0.01120797 0.075161049 0.05948354 1892 tags=14%, list=11%, signal=12% REG3A/PI3/ANXA1/AQP3/SPINK5/CTSK/WNT5A/CDH3/DSG3/CBFB/S100A7/CSTA/TRIM16/BCR/ABCA12/PALLD/KRT6A/SPRR1B/ADAM9/CYP27B1/PRKCH/SCEL/KAZN/CD109/KLK12/CTSL/ZBED2/MAP2K1/JAG1
GOBP_DETECTION_OF_EXTERNAL_BIOTIC_STIMULUS
GOBP_DETECTION_OF_EXTERNAL_BIOTIC_STIMULUS
GOBP_DETECTION_OF_EXTERNAL_BIOTIC_STIMULUS 22 0.63686802 1.612734611 0.011217949 0.075161049 0.05948354 2123 tags=27%, list=13%, signal=24% DMBT1/LY96/CLEC7A/TLR2/TLR1/NOD2
GOBP_CARBOXYLIC_ACID_TRANSPORT GOBP_CARBOXYLIC_ACID_TRANSPORT
GOBP_CARBOXYLIC_ACID_TRANSPORT 266 -0.316644693 -1.318232292 0.011235955 0.075161049 0.05948354 1476 tags=14%, list=9%, signal=13% RXRA/PLA2G12A/PLA2R1/MAP2K6/PRKAA1/ABCC6/GLS/BDKRB2/PER2/SLC35D2/SLC16A5/LLGL2/NTRK2/ATP8B1/NCOA2/NR1H4/CYP4F2/SLC46A1/SLC9A3R1/P2RX4/SFXN1/SLC26A3/ABCB11/SLC23A1/SLC36A1/SLC51B/SLC1A1/PLA2G12B/EDN1/SLC16A9/SLC17A4/SLC16A1/SLC3A1/SLC26A2/SLC51A/ABCG2
GOBP_NEGATIVE_REGULATION_OF_VIRAL_LIFE_CYCLE
GOBP_NEGATIVE_REGULATION_OF_VIRAL_LIFE_CYCLE
GOBP_NEGATIVE_REGULATION_OF_VIRAL_LIFE_CYCLE 23 0.629400265 1.6054098 0.011235955 0.075161049 0.05948354 1218 tags=35%, list=7%, signal=32% IFITM2/IFITM3/IFITM1/FCN1/BST2/FCN3/TRIM15/LY6E
GOBP_CELLULAR_CARBOHYDRATE_METABOLIC_PROCESS
GOBP_CELLULAR_CARBOHYDRATE_METABOLIC_PROCESS
GOBP_CELLULAR_CARBOHYDRATE_METABOLIC_PROCESS 273 -0.31953514 -1.335120295 0.011363636 0.075913933 0.060079384 2489 tags=23%, list=15%,

signal=20%
KAT2B/BRAF/INPP5A/PPP1CC/PPP4R3B/SYNJ1/GK5/SLC5A3/BAD/EP300/PHKB/HAS2/PYGB/IRS2/B4GALT5/SLC25A12/RB1CC1/INPP5K/PMAIP1/PPP1CB/GPT/PHKA2/PRKAG2/C1QTNF12/SCARB2/TREH/B3GNT2/SIRT1/PPARA/PCK2/IMPA2/INPP5J/PGM1/PER2/B3GALT5/PDK3/DGKQ/PPIP5K2/IMPA1/GNPTAB/OCRL/MST1/AGL/LCMT1/INSR/DYRK2/UGP2/NCOA2/RBKS/MOGAT2/ITPKA/PFKFB2/HAS3/BPNT1/B3GNT8/ENPP1/PPP1R3B/PDK2/PDK4/P2RY1/PPARGC1A/GBA3/PCK1
GOBP_POSITIVE_REGULATION_OF_LEUKOCYTE_CHEMOTAXIS
GOBP_POSITIVE_REGULATION_OF_LEUKOCYTE_CHEMOTAXIS
GOBP_POSITIVE_REGULATION_OF_LEUKOCYTE_CHEMOTAXIS 87 0.491079515
1.558981823 0.011444921 0.07635528 0.060428672 2508 tags=39%, list=15%, signal=33%
CXCL13/CXCL8/CXCL10/WNT5A/SERPINE1/CCR2/CD74/PLA2G7/RAC2/S100A7/CCR1/C5AR1/CCR7/CCL4/VEGFC/CALR/CCR6/ANO6/C3AR1/RIPOR2/CSF1R/GPSM3/CCL19/CXCL14/IL23A/IL6/CREB3/AIF1/FPR2/NCKAP1L/SWAP70/CMKLR1/MDK/CCL7
GOBP_REGULATION_OF_COAGULATION GOBP_REGULATION_OF_COAGULATION
GOBP_REGULATION_OF_COAGULATION 69 0.497440172 1.526985987 0.011713031
0.078040206 0.061762147 3008 tags=38%, list=18%, signal=31%
PLAU/CAV1/THBD/ANXA5/SERPINE2/SERPING1/F3/SERPINE1/TFPI/F2R/ST3GAL4/PDGFRA/PLAT/FAP/PROCR/ANO6/HPSE/VKORC1/SERPINF2/F12/FOXA2/C1QTNF1/APOE/PLAUR/CD34/PROS1
GOBP_REGULATION_OF_CATION_TRANSMEMBRANE_TRANSPORT
GOBP_REGULATION_OF_CATION_TRANSMEMBRANE_TRANSPORT
GOBP_REGULATION_OF_CATION_TRANSMEMBRANE_TRANSPORT 323 0.366731681
1.365656405 0.011764706 0.078280543 0.061952353 1872 tags=16%, list=11%, signal=15%
CEMIP/CXCL9/CXCL11/CXCL10/MMP9/CAV1/KCNE3/STOM/LYN/CCR2/PDE4B/GEM/JPH1/APLNR/F2R/GAL/TCIRG1/RAMP3/HOMER1/PLN/TWIST1/FXYD5/CD19/FYN/THY1/SESTD1/CCL2/GLRX/PLCG2/DIAPH1/PCSK9/CORO1A/RGS2/FLNA/GNB5/CLIC2/RASGRF2/SELENON/ANO6/PIK3CG/RGS4/TESC/GSTO1/PKD2/PRNP/FHL1/RANGRF/NEDD4/KCNMB1/ATP2B4/KCNAB2/IFNG
GOBP_REGULATION_OF_OXIDATIVE_STRESS_INDUCED_CELL_DEATH
GOBP_REGULATION_OF_OXIDATIVE_STRESS_INDUCED_CELL_DEATH
GOBP_REGULATION_OF_OXIDATIVE_STRESS_INDUCED_CELL_DEATH 58 0.527177219
1.580594363 0.01179941 0.07840747 0.062052805 2859 tags=31%, list=17%, signal=26%
MMP3/VNN1/SLC7A11/HIF1A/FYN/MET/P4HB/NME5/PARP1/LRRK2/SOD2/GPX1/FBXW7/ATF4/AKT1/PYCR1/PARK7/NR4A3
GOBP_PLATELET_AGGREGATION GOBP_PLATELET_AGGREGATION
GOBP_PLATELET_AGGREGATION 59 0.521982764 1.561844428 0.01183432
0.078444249 0.062081912 2759 tags=32%, list=16%, signal=27%
SLC7A11/SERPINE2/LYN/HBB/STXBP1/PDPN/FN1/PDGFRA/PLEK/MYL9/FLNA/PIK3CG/VCL/PEAR1/TLN1/BLK/SYK/TYRO3/C1QTNF1
GOBP_NEGATIVE_REGULATION_OF_MAP_KINASE_ACTIVITY
GOBP_NEGATIVE_REGULATION_OF_MAP_KINASE_ACTIVITY
GOBP_NEGATIVE_REGULATION_OF_MAP_KINASE_ACTIVITY 72 0.49240193

1.514181094 0.011851852 0.078444249 0.062081912 3071 tags=39%, list=18%, signal=32%

IL1B/AGT/CAV1/SERPINB3/LYN/LAX1/DUSP4/DUSP14/DUSP6/DUSP7/CD300A/HYAL2/RGS2/DNAJA1/SPRED1/RGS4/NUP62/DUSP10/SFRP1/SFRP2/PTPN1/UCHL1/CDK5RAP3/RGS3/APOE/IRAK3/SPRY1/GSTP1

GOBP_NEGATIVE_REGULATION_OF_TUMOR_NECROSIS_FACTOR_SUPERFAMILY_CYTOKINE_PRODUCTION

GOBP_NEGATIVE_REGULATION_OF_TUMOR_NECROSIS_FACTOR_SUPERFAMILY_CYTOKINE_PRODUCTION

GOBP_NEGATIVE_REGULATION_OF_TUMOR_NECROSIS_FACTOR_SUPERFAMILY_CYTOKINE_PRODUCTION 54 0.535055405 1.591328939 0.011851852 0.078444249 0.062081912

4292 tags=46%, list=25%, signal=35%

CD274/IL27RA/CLEC4A/TWIST1/TRIM27/SIRPA/LTF/AXL/LILRB1/HAVCR2/DICER1/NOD2/SYT11/ARRB2/IRAK3/CD34/GSTP1/SELENOS/CX3CL1/ILRUN/LBP/POMC/IL4/ARG2/CD33

GOBP_MESENCHYME_DEVELOPMENT GOBP_MESENCHYME_DEVELOPMENT

GOBP_MESENCHYME_DEVELOPMENT 268 0.37750857 1.381763093 0.012077295

0.079726036 0.063096337 2560 tags=28%, list=15%, signal=24%

GREM1/LOXL2/IL1B/WNT5A/COL1A1/RGCC/SERPINB3/ROBO1/FERMT2/BASP1/ENG/EDNRA/TGFB1/FAM83D/MEOX1/PDPN/ACTG2/HIF1A/FN1/TWIST1/TGFBR2/MAD2L2/ANXA6/SHH/STAT1/TGFB1I1/FOXF1/ACTA2/PDGFRB/SEMA4D/S100A4/FLNA/SNAI2/LEF1/DAB2/ZFPM2/WWTR1/RFLNB/EZH2/SPRED1/FGFR1/SEMA4B/OLFM1/NRP1/PKD2/NOLC1/DCHS1/ACVR1/CORO1C/ADAM15/SDCBP/IL6/SFRP1/LAMA5/JAG1/BNC2/PHLDB2/SEMA3G/WNT4/TGFBR1/SFRP2/MCRIP1/STRAP/SEMA4A/TEAD2/WNT2/VASN/POGLUT1/FOXF2/FOXA2/TRIM28/TGFB3/MDK/SEMA3F

GOBP_MONOACYLGLYCEROL_METABOLIC_PROCESS

GOBP_MONOACYLGLYCEROL_METABOLIC_PROCESS

GOBP_MONOACYLGLYCEROL_METABOLIC_PROCESS 11 -0.735151285 -1.694922056

0.012077295 0.079726036 0.063096337 3322 tags=64%, list=20%, signal=51%

PLA2G4A/ABHD16B/MOGAT3/FAAH/ABHD12/MGLL/MOGAT2

GOBP_CELL_CYCLE_G2_M_PHASE_TRANSITION GOBP_CELL_CYCLE_G2_M_PHASE_TRANSITION

GOBP_CELL_CYCLE_G2_M_PHASE_TRANSITION 265 0.375635031 1.372937648

0.012150668 0.080104997 0.063396253 4110 tags=42%, list=24%, signal=33%

CDC25B/PHLDA1/PPP1R12B/PRKAR2B/PSME4/PSMB9/CCNB1/NABP1/TUBG1/DONSON/FOXM1/CDC25A/BLM/CEP78/PPP2R2A/PSMD14/MELK/CEP164/CSNK1E/KIF14/CDC6/PSMD12/CDK1/PSMC2/PLCB1/TUBA4A/PSMB2/AURKB/NES/PSMA3/AURKA/ODF2/PSMD1/TUBB/CCND1/PLK1/TPD52L1/PSMA5/AJUBA/TPX2/AVEN/FHL1/RCC2/CDC7/CDK2/PSMD6/PKIA/PSMB5/PSMC4/CETN2/CCNH/NBN/CEP135/ATF5/PSMB1/PSMA1/ARPP19/CDK4/PINX1/CHEK1/UIMC1/RAD51C/PSME3/CLSPN/CDK5RAP3/NABP2/NPM1/CUL1/PSMA7/PSMB8/NDE1/PSMC5/TUBA1A/CCNA2/PSMB10/HSP90AA1/PSMB6/PSMA4/FBXO5/INTS3/PSMD13/PSME1/VPS4A/YWHAG/BRCA1/CCNB2/USP47/RINT1/CALM3/PSMC1/DTL/PPP1R12A/ZNF830/NEDD1/FBXL7/SKP2/PSMB4/PLK4/CENPF/PPME1/PSMB3/PSMD2/TAF2/HAUS7/PSMC6/PSMD7/PSMA2/HAUS1/MRE11/PSMD4/NEK2/HAUS2

GOBP_REGULATION_OF_T_CELL_RECEPTOR_SIGNALING_PATHWAY

GOBP_REGULATION_OF_T_CELL_RECEPTOR_SIGNALING_PATHWAY

GOBP_REGULATION_OF_T_CELL_RECEPTOR_SIGNALING_PATHWAY 40 0.564124667

1.592360001 0.012345679 0.081177289 0.06424488 2319 tags=38%, list=14%,

signal=32%

GBP1/ADA/KCNN4/CCR7/THY1/CD300A/CD81/LAPTM5/DGKZ/RAB29/LCK/PRNP/LILRB4/RELA/MALT1

GOBP_SUBSTRATE_ADHESION_DEPENDENT_CELL_SPREADING

GOBP_SUBSTRATE_ADHESION_DEPENDENT_CELL_SPREADING

GOBP_SUBSTRATE_ADHESION_DEPENDENT_CELL_SPREADING 108 0.450677321

1.483493542 0.012345679 0.081177289 0.06424488 2645 tags=31%, list=16%, signal=26%

OLFM4/GBP1/FERMT2/LAMC1/PDPN/FN1/ANTXR1/POSTN/FLNA/ITGA8/P4HB/TEK/CALR/KIF14/TACSTD2/LAMB1/ITGB1BP1/NRP1/LAMB2/PARVB/LPXN/CORO1C/ITGAV/RCC2/AXL/LAMA5/MICALL2/FZD4/PARVA/PARVG/MDK/TYRO3/ITGA4

GOBP_MEMBRANE_PROTEIN_ECTODOMAIN_PROTEOLYSIS

GOBP_MEMBRANE_PROTEIN_ECTODOMAIN_PROTEOLYSIS

GOBP_MEMBRANE_PROTEIN_ECTODOMAIN_PROTEOLYSIS41 0.569261751 1.614335087

0.01236476 0.081196339 0.064259956 2843 tags=39%, list=17%, signal=33%

TIMP1/IL1B/MMP7/BACE2/ADAM19/ADAM9/TNFRSF1B/ADAM8/IFNG/TIMP2/NRDC/FURIN/TIMP3/BACE1/APOE/ROCK1

GOBP_POSITIVE_REGULATION_OF_LIPID_KINASE_ACTIVITY

GOBP_POSITIVE_REGULATION_OF_LIPID_KINASE_ACTIVITY

GOBP_POSITIVE_REGULATION_OF_LIPID_KINASE_ACTIVITY 37 0.569793767

1.582650215 0.012403101 0.081341643 0.064374952 2123 tags=41%, list=13%, signal=36%

LYN/TGFB1/KIT/PDGFRA/CCR7/CD19/FGR/PDGFRB/CD81/IRS1/TEK/DGKZ/CCL19/FPR2/NOD2

GOBP_RESPONSE_TO_LEUCINE GOBP_RESPONSE_TO_LEUCINE

GOBP_RESPONSE_TO_LEUCINE 13 -0.715188932 -1.74120825 0.012437811

0.08146279 0.06447083 1813 tags=38%, list=11%, signal=34%

UBR2/LARS1/SESN1/SESN3/PPARGC1A

GOBP_ACTIN_CYTOSKELETON_REORGANIZATION

GOBP_ACTIN_CYTOSKELETON_REORGANIZATION

GOBP_ACTIN_CYTOSKELETON_REORGANIZATION103 0.460217385 1.503050093 0.0125

0.081763364 0.064708708 3438 tags=39%, list=20%, signal=31%

ANXA1/HCLS1/PLEK2/ANTXR1/KIT/PDGFRA/PLEK/GMFG/HCK/ARHGDIB/BAIAP2L1/FLNA/S1PR1/TEK/CSF1R/NRP1/PARVB/GPR65/RAP2A/ARHGDIA/FGF7/PHACTR1/MICALL2/ESAM/PARVA/PARVG/PTPN1/HAX1/MDK/CDC42BPB/FES/ATP2C1/CTTN/HRAS/SYDE1/RAPGEF3/SHC1/TRPM2/ABL2/S1PR2

GOBP_NEGATIVE_REGULATION_OF_G_PROTEIN_COUPLED_RECEPTOR_SIGNALING_PATHWAY

GOBP_NEGATIVE_REGULATION_OF_G_PROTEIN_COUPLED_RECEPTOR_SIGNALING_PATHWAY

GOBP_NEGATIVE_REGULATION_OF_G_PROTEIN_COUPLED_RECEPTOR_SIGNALING_PATHWAY 47 0.55072648 1.587654665 0.012539185 0.081912879 0.064827036 2879

tags=32%, list=17%, signal=27%

CXCL8/ADA/PDE4B/MRAP2/APLNR/PLEK/GNAI2/MET/RGS2/RGS4/ATP2B4/ARRB2/CRY1/ARRDC3/ADRB2

GOBP_REGULATION_OF_ENDOCRINE_PROCESS GOBP_REGULATION_OF_ENDOCRINE_PROCESS GOBP_REGULATION_OF_ENDOCRINE_PROCESS 34 0.573263491 1.572302539 0.01255887 0.081934785 0.064844373 2994 tags=32%, list=18%, signal=27% GJA1/AGT/INHBA/F2R/GAL/RAB8B/INHBB/BMP6/C1QTNF1/CRY1/PTPN11

GOBP_ENDOPLASMIC_RETICULUM_TO_GOLGI_VESICLE_MEDIATED_TRANSPORT GOBP_ENDOPLASMIC_RETICULUM_TO_GOLGI_VESICLE_MEDIATED_TRANSPORT GOBP_ENDOPLASMIC_RETICULUM_TO_GOLGI_VESICLE_MEDIATED_TRANSPORT 192 0.388188995 1.374109166 0.012594458 0.081936156 0.064845458 4225 tags=36%, list=25%, signal=28% CD55/KDELR3/CREB3L2/ARFGAP3/SERPINA1/GAS1/HYOU1/SEC24D/SEC22C/PREB/SEC13/LMAN1/VCP/CTSC/TMED3/INSIG1/USO1/MCFD2/COL7A1/COPB2/SEC24C/SAR1A/SEC23B/PPP6R1/BET1/BET1L/SEC24A/SEC16A/RANGRF/TRAPPC12/YIF1A/TMED9/ARF4/KDELR2/SEC22B/COPA/CD59/YIF1B/COPZ2/LRRK2/SEC31A/TRAPPC2L/YIPF5/COPB1/ARFGAP1/KDELR1/STX5/STX17/SCFD1/YKT6/PROS1/PEF1/COG5/DCTN6/TEX261/RINT1/ARCN1/TFG/COPE/STX18/ARF1/DYNC1LI1/COPG1/TMED2/COPZ1/GOLGA2/FOLR1/GBF1/TRAPPC10/SEC23IP

GOBP_NEGATIVE_REGULATION_OF_RESPONSE_TO_BIOTIC_STIMULUS GOBP_NEGATIVE_REGULATION_OF_RESPONSE_TO_BIOTIC_STIMULUS GOBP_NEGATIVE_REGULATION_OF_RESPONSE_TO_BIOTIC_STIMULUS 94 0.475630141 1.527605636 0.012676056 0.081936156 0.064845458 3688 tags=38%, list=22%, signal=30% MMP12/SPINK5/SERPINB9/SERPING1/HTRA1/IFI16/SLAMF8/SEC14L1/CARD16/SERPINB4/NLRC5/SAMHD1/MICB/LYAR/PARP14/LTF/A2M/METTL3/DUSP10/LILRB1/HAVCR2/NMI/ADAR/TYRO3/ARRB2/TRIM21/IRAK3/UFD1/TRAFD1/IL2RA/CR1/MAPKBP1/TARBP2/DHX58/ILRUN/HLA-E

GOBP_ANATOMICAL_STRUCTURE_HOMEOSTASIS GOBP_ANATOMICAL_STRUCTURE_HOMEOSTASIS GOBP_ANATOMICAL_STRUCTURE_HOMEOSTASIS 437 0.349465697 1.321420774 0.012687428 0.081936156 0.064845458 2599 tags=22%, list=15%, signal=19% LPCAT1/GJA1/SERPINA3/SPP1/PECAM1/CLDN1/CD38/TFF1/CDH3/ZG16B/LAMC1/TFF2/CTSH/CDH5/AZGP1/FEN1/RAC2/BAG3/TNFRSF11B/MFSD2A/LDB2/F2R/TCIRG1/HIF1A/MUC4/HOMER1/GPR137B/VSIG1/ADGRF5/SASH3/LOX/POLD3/SNX10/ABCA12/RTEL1/CORO1A/BLM/LTF/JAM2/S1PR1/WWTR1/LYZ/CLDN18/AKR1B1/CSF1R/CFL2/CLDN12/LIPA/AURKB/HNRNPD/NAT10/BARD1/ACD/JAM3/ATR/ACP5/VCL/EXO1/ACACA/AKT3/PML/CCT3/IL6/TNKS1BP1/RFC3/ADAM8/PARP1/MUC2/CCT8/POLE2/RAB3D/DEF8/TMEM119/CCT5/MAP1A/CCT2/RBP4/TMEM64/NBN/TNFSF11/P2RX7/NOD2/INPP5D/ESAM/STRAP/CCT6A/CCT4/CCT7/PINX1/RAD51C/NHP2/RFC4/HSP90AB1/SYK/POLD2/NABP2/TYRO3

GOBP_CELLULAR_ANION_HOMEOSTASIS GOBP_CELLULAR_ANION_HOMEOSTASIS GOBP_CELLULAR_ANION_HOMEOSTASIS 14 -0.692770342 -1.709534775 0.012690355 0.081936156 0.064845458 301 tags=36%, list=2%, signal=35% SLC34A1/SLC12A2/SLC9A3R1/ENPP1/CKB

GOBP_PRIMARY_AMINO_COMPOUND_METABOLIC_PROCESS GOBP_PRIMARY_AMINO_COMPOUND_METABOLIC_PROCESS GOBP_PRIMARY_AMINO_COMPOUND_METABOLIC_PROCESS 14 -0.694454882 -1.713691678 0.012690355 0.081936156 0.064845458 981 tags=57%, list=6%, signal=54% BTBD9/AZIN2/HTR1A/SRD5A1/CHKA/GDE1/TPH1/DDC

GOBP_ANTIGEN_PROCESSING_AND_PRESENTATION_OF_ENDOGENOUS_ANTIGEN GOBP_ANTIGEN_PROCESSING_AND_PRESENTATION_OF_ENDOGENOUS_ANTIGEN GOBP_ANTIGEN_PROCESSING_AND_PRESENTATION_OF_ENDOGENOUS_ANTIGEN 24 0.625858169 1.60678766 0.012718601 0.081936156 0.064845458 746 tags=21%, list=4%, signal=20% CD74/AZGP1/HLA-DRA/TAP2/TAP1

GOBP_REGULATION_OF_B_CELL_RECEPTOR_SIGNALING_PATHWAY GOBP_REGULATION_OF_B_CELL_RECEPTOR_SIGNALING_PATHWAY GOBP_REGULATION_OF_B_CELL_RECEPTOR_SIGNALING_PATHWAY 24 0.622337925 1.597750015 0.012718601 0.081936156 0.064845458 2366 tags=50%, list=14%, signal=43% LYN/CBFB/CD19/FCGR2B/CD300A/CD81/PRKCB/FCRL3/PRKCH/LPXN/SLC39A10/BLK

GOBP_GLUCOSAMINE_CONTAINING_COMPOUND_METABOLIC_PROCESS GOBP_GLUCOSAMINE_CONTAINING_COMPOUND_METABOLIC_PROCESS GOBP_GLUCOSAMINE_CONTAINING_COMPOUND_METABOLIC_PROCESS 25 0.606421096 1.57335972 0.012738854 0.081936156 0.064845458 737 tags=20%, list=4%, signal=19% CHI3L1/CHI3L2/PGM3/CHST2/GNPNAT1

GOBP_LYMPH_VESSEL_DEVELOPMENT GOBP_LYMPH_VESSEL_DEVELOPMENT GOBP_LYMPH_VESSEL_DEVELOPMENT 25 0.613583515 1.59194262 0.012738854 0.081936156 0.064845458 3079 tags=40%, list=18%, signal=33% HEG1/PDPN/TMEM204/TIE1/PTPN14/VEGFC/CLEC14A/VASH1/SYK/NR2F2

GOBP_PROTEIN_IMPORT GOBP_PROTEIN_IMPORT GOBP_PROTEIN_IMPORT 178 0.399463741 1.401001523 0.012755102 0.081936156 0.064845458 3984 tags=40%, list=24%, signal=31% MMP12/HCLS1/TGFB1/BAG3/CLU/JAK2/SHH/IPO4/KPNA2/SEC13/RAB8B/HYAL2/FLNA/STAT3/RAB23/TNPO1/ECT2/NUP62/TNPO2/RAN/NOLC1/E2F3/NUP98/IPO9/HIKESHI/PML/ZPR1/PKIA/NUP85/IFNG/DNLZ/NXT1/KPNB1/APOD/TIMM17A/PPP3CA/CSE1L/NUP93/LRRK2/KPNA6/FERMT1/HSP90AB1/TRIM28/SYK/AKT1/TOMM40/PAM16/APOE/TARDBP/NUP62CL/GRPEL2/HSP90AA1/HSPA4/HEATR3/NUP58/HSPD1/ELAVL1/PRICKLE1/UFM1/MBTPS1/IPO7/NUP50/GRPEL1/PKIG/TIMM50/NUP35/KPNA1/NUP155/NUP107/IPO13/POM121L12/RANBP2

GOBP_POSITIVE_REGULATION_OF_T_HELPER_CELL_DIFFERENTIATION GOBP_POSITIVE_REGULATION_OF_T_HELPER_CELL_DIFFERENTIATION GOBP_POSITIVE_REGULATION_OF_T_HELPER_CELL_DIFFERENTIATION 21 0.656330331 1.64354095 0.012759171 0.081936156 0.064845458 2677 tags=48%, list=16%, signal=40% ANXA1/NFKBIZ/RIPK2/CD86/IL12RB1/CCL19/IL23A/HLX/MALT1/IL18

GOBP_PYRIMIDINE_NUCLEOSIDE_TRIPHOSPHATE_METABOLIC_PROCESS GOBP_PYRIMIDINE_NUCLEOSIDE_TRIPHOSPHATE_METABOLIC_PROCESS GOBP_PYRIMIDINE_NUCLEOSIDE_TRIPHOSPHATE_METABOLIC_PROCESS 21 0.649241599 1.625789795 0.012759171 0.081936156 0.064845458 2060 tags=43%, list=12%, signal=38% CTPS1/NME1/ENTPD7/NME5/DTYMK/TYMS/NME4/NME7/CAD

GOBP_REGULATION_OF_EPITHELIAL_TO_MESENCHYMAL_TRANSITION GOBP_REGULATION_OF_EPITHELIAL_TO_MESENCHYMAL_TRANSITION GOBP_REGULATION_OF_EPITHELIAL_TO_MESENCHYMAL_TRANSITION 92 0.475595089 1.520563441 0.012784091 0.081936156 0.064845458 2506 tags=37%, list=15%, signal=32%

GREM1/LOXL2/IL1B/COL1A1/RGCC/SERPINB3/FERMT2/ENG/TGFB1/PDPN/TWIST1/TGFBR2/MAD2L2/TGFB1I1/LEF1/DAB2/WWTR1/EZH2/SPRED1/OLFM1/ACVR1/SDCBP/IL6/SFRP1/JAG1/PHLDB2/TGFBR1/SFRP2/MCRIP1/STRAP/VASN/FOXA2/TGFB3/MDK
GOBP_RESPONSE_TO_CAFFEINE GOBP_RESPONSE_TO_CAFFEINE GOBP_RESPONSE_TO_CAFFEINE 16 -0.683414986 -1.746059609 0.012787724 0.081936156 0.064845458 1535 tags=31%, list=9%, signal=28% GSTM2/PRKAA1/TMEM38B/PPARG/PPARGC1A
GOBP_VESICLE_MEDIATED_TRANSPORT_TO_THE_PLASMA_MEMBRANE GOBP_VESICLE_MEDIATED_TRANSPORT_TO_THE_PLASMA_MEMBRANE GOBP_VESICLE_MEDIATED_TRANSPORT_TO_THE_PLASMA_MEMBRANE 86 -0.412886797 -1.479863269 0.012903226 0.08257077 0.065347701 2985 tags=31%, list=18%, signal=26% BBS1/EXOC1/OPTN/GOPC/CLN3/DNM2/LYPLA1/EXOC8/CLSTN1/PREPL/AKAP5/CNST/RAB7A/GRIP1/EXOC6B/BBS2/GOLPH3L/STEAP2/VPS26C/ANKRD27/WASHC2C/PKDCC/STX3/ARHGAP44/SLC1A1/AMN/ANK3
GOBP_REGULATION_OF_RESPONSE_TO_OXIDATIVE_STRESS GOBP_REGULATION_OF_RESPONSE_TO_OXIDATIVE_STRESS GOBP_REGULATION_OF_RESPONSE_TO_OXIDATIVE_STRESS 79 0.487515548 1.526172469 0.01300578 0.083030265 0.065711352 2630 tags=29%, list=16%, signal=25% MMP3/VNN1/SLC7A11/ALOX5/MCTP1/FUT8/HIF1A/FYN/MET/P4HB/FBLN5/NME5/SELENON/NCOA7/PARP1/LRRK2/SOD2/GPX1/FBXW7/ATF4/AKT1/PYCR1/PARK7
GOBP_LYMPHANGIOGENESIS GOBP_LYMPHANGIOGENESIS GOBP_LYMPHANGIOGENESIS 17 0.677253917 1.608358811 0.01300813 0.083030265 0.065711352 1938 tags=35%, list=11%, signal=31% PDPN/TIE1/PTPN14/VEGFC/CLEC14A/VASH1
GOBP_REGULATION_OF_CHROMOSOME_SEGREGATION GOBP_REGULATION_OF_CHROMOSOME_SEGREGATION GOBP_REGULATION_OF_CHROMOSOME_SEGREGATION 83 0.492297968 1.541796527 0.013138686 0.083757037 0.06628653 5156 tags=59%, list=30%, signal=41% TRIP13/CCNB1/MAD2L2/BUB1/CDC20/NDC80/NEK6/TACC3/PTTG1/CDC6/MKI67/CENPE/AURKB/BUB1B/PLK1/UBE2C/DLGAP5/CDC27/RCC2/ZWINT/KIF2C/HECW2/CDC26/CDT1/SPDL1/KLHL22/KNTC1/ANAPC7/PLSCR1/FBXO5/ZNF207/CENPF/BUB3/TTK/CHFR/MAD2L1/DYNC1LI1/CSNK2A2/ATM/HNRNPU/ZW10/GEN1/CDK5RAP2/SIRT2/MAD1L1/ANAPC5/CDC16/RIOK2/TPR
GOBP_LYMPHOCYTE_APOPTOTIC_PROCESS GOBP_LYMPHOCYTE_APOPTOTIC_PROCESS GOBP_LYMPHOCYTE_APOPTOTIC_PROCESS 69 0.495917578 1.522312099 0.01317716 0.083895698 0.066396268 2688 tags=30%, list=16%, signal=26% CD274/IDO1/WNT5A/IL7R/LYN/ADA/CD74/JAK3/HIF1A/GIMAP8/CD27/BCL6/AURKB/BTK/ADAM8/SLC39A10/TSC22D3/BLK/NOC2L/AKT1/DOCK8
GOBP_PYRIMIDINE_RIBONUCLEOSIDE_TRIPHOSPHATE_METABOLIC_PROCESS GOBP_PYRIMIDINE_RIBONUCLEOSIDE_TRIPHOSPHATE_METABOLIC_PROCESS GOBP_PYRIMIDINE_RIBONUCLEOSIDE_TRIPHOSPHATE_METABOLIC_PROCESS 15 0.692356228 1.615891072 0.01322314 0.084081744 0.066543507 2060 tags=47%, list=12%, signal=41% CTPS1/NME1/ENTPD7/NME5/NME4/NME7/CAD
GOBP_NEGATIVE_REGULATION_OF_TRANSFERASE_ACTIVITY

GOBP_NEGATIVE_REGULATION_OF_TRANSFERASE_ACTIVITY
GOBP_NEGATIVE_REGULATION_OF_TRANSFERASE_ACTIVITY 266 0.375010839
1.371405579 0.013349515 0.084777867 0.067094429 3449 tags=33%, list=20%, signal=27%
WARS1/IL1B/AGT/TRIB2/CAV1/SERPINB3/LYN/HEG1/LAX1/BAG2/SH3BP5/PTPRC/PRKAR2B/SOCS3/DNAJC3/DUSP4/DUSP14/MAD2L2/THY1/GMFG/DUSP6/TRIM27/DUSP7/LATS2/CD300A/HHEX/HYAL2/MYOCD/RHOH/RGS2/DNAJA1/WWTR1/SPRED1/RGS4/NUP62/TESC/ITGB1BP1/DUSP10/PLK1/PIP4K2A/GPRC5A/IBTK/CORO1C/DUSP22/ITPRIP/CDKN2C/AJUBA/LILRB4/SFRP1/PKIA/IFNG/CDKN1C/CHORDC1/RUBCN/SFRP2/PARVA/PTPN1/PINX1/FOXA2/ADAR/UCHL1/CDK5RAP3/AKT1/RGS3/PARK7/NPM1/MLLT1/PRKAR1A/PIF1/LIMK1/DVL1/APOE/PPM1F/SOCS4/IRAK3/SPRY1/GSTP1/NR2F2/RASIP1/FBXO5/ERRFI1/CDK5RAP1/NF2/YWHAG/AIDA/PKN1/TARBP2/IPO7/EPHA1
GOBP_AMMONIUM_ION_METABOLIC_PROCESS
GOBP_AMMONIUM_ION_METABOLIC_PROCESS
GOBP_AMMONIUM_ION_METABOLIC_PROCESS 25 -0.592133575 -1.649366061
0.013368984 0.084794175 0.067107336 1656 tags=44%, list=10%, signal=40%
PRG3/BTBD9/ENPP6/HTR1A/SLC44A1/SRD5A1/CHKA/BCHE/TPH1/DDC/HNMT
GOBP_VIRAL_BUDDING GOBP_VIRAL_BUDDING GOBP_VIRAL_BUDDING 24 -0.600297233
-1.65491293 0.013404826 0.084914155 0.06720229 2110 tags=42%, list=12%, signal=37%
VPS28/CHMP2A/MVB12B/CHMP4C/VPS4B/VTA1/PDCD6IP/CHMP5/CHMP1B/CHMP4B
GOBP_NEGATIVE_REGULATION_OF_CELL_CYCLE_PHASE_TRANSITION
GOBP_NEGATIVE_REGULATION_OF_CELL_CYCLE_PHASE_TRANSITION
GOBP_NEGATIVE_REGULATION_OF_CELL_CYCLE_PHASE_TRANSITION 238 0.386770814
1.399337458 0.013431013 0.084972755 0.067248666 4774 tags=52%, list=28%, signal=38%
RGCC/TRIP13/PSME4/PSMB9/CCNB1/NABP1/MAD2L2/SLFN11/CCL2/BUB1/CDC20/DACT1/DONSON/NDC80/BLM/PSMD14/CDC6/EZH2/PSMD12/CDK1/PSMC2/MUC1/PSMB2/AURKB/PSMA3/AURKA/PSMD1/BUB1B/CCND1/PLK1/PKD2/PRMT1/PSMA5/CDKN2C/AVEN/PML/FHL1/TNKS1BP1/CDK2/ZWINT/PSMD6/CNOT9/GPNMB/PSMB5/PSMC4/PLK2/JADE1/NBN/ATF5/ARID3A/PSMB1/PSMA1/CDK4/PINX1/CHEK1/UIMC1/PSME3/CLSPN/CDT1/CDK5RAP3/NABP2/FAM107A/SPDL1/CNOT6L/CUL1/PSMA7/PSMB8/PSMC5/KLHL22/TRIAP1/NACC2/KNTC1/PSMB10/PSMB6/PSMA4/FBXO5/INTS3/PCBP4/PSMD13/PSME1/VPS4A/BRCA1/USP47/RINT1/PSMC1/DTL/ZNF830/FBXL7/MDM2/ZFP36L1/ZNF207/PSMB4/CENPF/PSMB3/PSMD2/BUB3/TTK/KANK2/CHFR/PSMC6/MAD2L1/PSMD7/DYNC1LI1/PSMA2/MRE11/PSMD4/MDM4/PSMD9/CDK2AP2/MRNIP/CNOT11/GTSE1/TFDP1/ATM/GML/BABAM2/E2F8/PSMB7/PRKDC/CNOT3/ZW10/BRD7/GEN1/CDK5RAP2
GOBP_POSITIVE_REGULATION_OF_LIPID_METABOLIC_PROCESS
GOBP_POSITIVE_REGULATION_OF_LIPID_METABOLIC_PROCESS
GOBP_POSITIVE_REGULATION_OF_LIPID_METABOLIC_PROCESS 143 0.428261166
1.464455216 0.013513514 0.085387024 0.067576525 2859 tags=29%, list=17%, signal=25%
ANXA1/ELOVL5/IL1B/AGT/LYN/CCN1/LPGAT1/CD74/TGFB1/MFSD2A/STARD4/PTGS2/TWIST1/KIT/PDGFRA/CCR7/CD19/ADGRF5/FGR/PDGFRB/CD81/IRS1/TEK/DGKZ/APOC1/MID1IP1/CCL19/CCDC3/IFNG/FPR2/CREB1/WNT4/NOD2/BMP6/AKT1/ACSL3/RAB38/PLA2G3/SLC45A3/APOE/FGF2/NR4A3

GOBP_AMINO_SUGAR_CATABOLIC_PROCESS GOBP_AMINO_SUGAR_CATABOLIC_PROCESS GOBP_AMINO_SUGAR_CATABOLIC_PROCESS 12 0.726848952 1.59245559 0.013582343 0.085713981 0.067835283 1335 tags=25%, list=8%, signal=23% CHI3L1/CHI3L2/MGAT1

GOBP_NEGATIVE_REGULATION_OF_MUSCLE_TISSUE_DEVELOPMENT GOBP_NEGATIVE_REGULATION_OF_MUSCLE_TISSUE_DEVELOPMENT GOBP_NEGATIVE_REGULATION_OF_MUSCLE_TISSUE_DEVELOPMENT 11 0.750197279 1.611330667 0.013605442 0.085751889 0.067865284 947 tags=36%, list=6%, signal=34% IGFBP5/TGFB1/TWIST1/LEF1

GOBP_GPI_ANCHOR_METABOLIC_PROCESS GOBP_GPI_ANCHOR_METABOLIC_PROCESS GOBP_GPI_ANCHOR_METABOLIC_PROCESS 31 -0.553136981 -1.609891054 0.013623978 0.085760977 0.067872477 3064 tags=35%, list=18%, signal=29% PGAP2/PIGU/PIGS/PIGN/MPPE1/PIGL/PIGV/PGAP3/PGAP1/PIGZ/CWH43

GOBP_NEPHRON_DEVELOPMENT GOBP_NEPHRON_DEVELOPMENT GOBP_NEPHRON_DEVELOPMENT 136 0.422457717 1.432465188 0.013812155 0.086558067 0.068503305 3043 tags=28%, list=18%, signal=23% GREM1/PECAM1/AGT/NID1/BASP1/SERPINB7/SULF1/PODXL/PDGFRA/SHH/STAT1/ACTA2/PDGFRB/SULF2/TEK/SEC61A1/WWTR1/TACSTD2/PKD2/LAMB2/CFLAR/DCHS1/LIF/AHI1/LAMA5/NUP85/JAG1/MPV17/GPR4/WNT4/NPNT/SALL1/ANGPT2/DLL1/FGF2/CD34/HOXA11/ITGA3

GOBP_REGULATION_OF_T_HELPER_CELL_DIFFERENTIATION GOBP_REGULATION_OF_T_HELPER_CELL_DIFFERENTIATION GOBP_REGULATION_OF_T_HELPER_CELL_DIFFERENTIATION 36 0.576824965 1.595980243 0.013824885 0.086558067 0.068503305 2677 tags=39%, list=16%, signal=33% ANXA1/JAK3/IRF4/HLA-DRA/NFKBIZ/RIPK2/CD86/BCL6/IL12RB1/CCL19/IL23A/HLX/MALT1/IL18

GOBP_DNA_REPLICATION_INITIATION GOBP_DNA_REPLICATION_INITIATION GOBP_DNA_REPLICATION_INITIATION 39 0.553353858 1.555742316 0.013846154 0.086558067 0.068503305 3444 tags=41%, list=20%, signal=33% NOC3L/MCM6/CDC45/CDC6/MCM10/GINS3/POLE2/ORC6/NBN/MCM3/MCM5/CDT1/MCM7/ORC5/POLA2/MCM2

GOBP_NEGATIVE_REGULATION_OF_LEUKOCYTE_CELL_CELL_ADHESION GOBP_NEGATIVE_REGULATION_OF_LEUKOCYTE_CELL_CELL_ADHESION GOBP_NEGATIVE_REGULATION_OF_LEUKOCYTE_CELL_CELL_ADHESION 119 0.439372407 1.462485377 0.013850416 0.086558067 0.068503305 3388 tags=34%, list=20%, signal=28% ANXA1/CD274/IDO1/ASS1/LAX1/CD74/JAK3/CBFB/FCGR2B/SHH/CEBPB/LILRB2/CD86/CD300A/BCL6/LAPTM5/IRF1/RIPOR2/RUNX3/TWSG1/PRNP/LAG3/LILRB4/TMEM131L/LILRB1/GPNMB/HAVCR2/HLX/GLMN/MDK/LRRC32/AKT1/PRKAR1A/DLG5/BTN2A2/CTLA4/RC3H2/IL2RA/PLA2G2D/HMGB1/CR1

GOBP_LACTATION GOBP_LACTATION GOBP_LACTATION 43 0.543673787 1.556511124 0.013867488 0.086558067 0.068503305 3294 tags=42%, list=19%, signal=34% CAV1/NME1/OAS2/HIF1A/XBP1/PPAT/XDH/GPAT4/PAM/CCND1/HK2/UMPS/CREB1/CAD/ATP7A/STAT5B/STAT5A/APRT

GOBP_MACROPHAGE_DIFFERENTIATION GOBP_MACROPHAGE_DIFFERENTIATION GOBP_MACROPHAGE_DIFFERENTIATION 43 0.546962293 1.565925955 0.013867488 0.086558067 0.068503305 1872 tags=33%, list=11%, signal=29% MMP9/HCLS1/TGFB1/PF4/INHBA/TLR2/TSPAN2/CSF1R/NKX2-3/NRROS/LIF/CASP8/PARP1/IFNG

GOBP_PATTERN_RECOGNITION_RECEPTOR_SIGNALING_PATHWAY GOBP_PATTERN_RECOGNITION_RECEPTOR_SIGNALING_PATHWAY GOBP_PATTERN_RECOGNITION_RECEPTOR_SIGNALING_PATHWAY 191 0.38782625 1.371883323 0.013871375 0.086558067 0.068503305 3272 tags=31%, list=19%, signal=26% S100A8/TNIP3/S100A9/CTSK/CAV1/LYN/RFTN1/LY96/BIRC3/PIK3AP1/IRF4/SEC14L1/RIPK2/TLR8/TIFA/FCN1/ITGB2/TLR2/SLC15A3/CD300A/FFAR2/IRAK1/CD40/ITGAM/LTF/TRIM15/CLEC4E/IRF1/CTSB/FCRL3/IRAK2/HSP90B1/TLR1/BTK/CASP8/CD300LF/CTSL/HAVCR2/CNPY3/RELA/PTPRS/NOD2/IRF7/MAPKAPK2/NMI/TRAF3/CYLD/TYRO3/TANK/UBE2N/IRF3/ARRB2/TBK1/IRAK3/TLR10/HSPD1/UFD1/OTULIN/IFI35/SLC15A4

GOBP_PROSTATE_GLAND_DEVELOPMENT GOBP_PROSTATE_GLAND_DEVELOPMENT GOBP_PROSTATE_GLAND_DEVELOPMENT 41 0.567700361 1.609907237 0.013910355 0.086693483 0.068610475 1117 tags=20%, list=7%, signal=18% SERPINB5/TNC/ANXA1/WNT5A/SULF1/SHH/SERPINF1/WDR77

GOBP_UNSATURATED_FATTY_ACID_BIOSYNTHETIC_PROCESS GOBP_UNSATURATED_FATTY_ACID_BIOSYNTHETIC_PROCESS GOBP_UNSATURATED_FATTY_ACID_BIOSYNTHETIC_PROCESS 45 0.552093206 1.587111351 0.014018692 0.087260268 0.069059037 1805 tags=29%, list=11%, signal=26% ANXA1/ELOVL5/IL1B/PTGDS/CD74/SCD/TBXAS1/PTGS2/DEGS1/PTGS1/MIF/ELOVL6/PTGIS

GOBP_POSITIVE_REGULATION_OF_FIBROBLAST_PROLIFERATION GOBP_POSITIVE_REGULATION_OF_FIBROBLAST_PROLIFERATION GOBP_POSITIVE_REGULATION_OF_FIBROBLAST_PROLIFERATION 44 0.542154387 1.55274839 0.014040562 0.087288101 0.069081065 2420 tags=36%, list=14%, signal=31% AGT/WNT5A/CD74/FN1/CCNB1/PDGFRA/PDGFRB/DDR2/CDC6/SPHK1/MIF/PML/PDGFC/CDK4/WNT2/AQP1

GOBP_XENOBIOTIC_TRANSPORT GOBP_XENOBIOTIC_TRANSPORT GOBP_XENOBIOTIC_TRANSPORT 40 -0.530619967 -1.631010635 0.014124294 0.087699978 0.06940703 518 tags=22%, list=3%, signal=22% NR1I2/ABCA8/SLC22A4/ABCB11/SLC36A1/PDZK1/SLC22A5/ABCB1/ABCG2

GOBP_ACTIVATION_OF_PROTEIN_KINASE_ACTIVITY GOBP_ACTIVATION_OF_PROTEIN_KINASE_ACTIVITY GOBP_ACTIVATION_OF_PROTEIN_KINASE_ACTIVITY 313 0.361762192 1.341033178 0.014251781 0.088357021 0.069927023 2879 tags=25%, list=17%, signal=21% CHI3L1/GREM1/PROK2/IL1B/AGT/WNT5A/RGCC/MLKL/FPR1/PEA15/CD74/PTPRC/F2R/RIPK2/PRKAR2B/C5AR1/KIT/TGFBR2/JAK2/CXCR4/CD86/MAP3K5/MAP4K4/DUSP6/DUSP7/CD300A/IRAK1/CD81/OSBPL8/IGFBP6/TNFRSF10A/MAP3K20/MAP3K6/GPRC5B/MAP4K1/TNFSF15/TNFRSF10B/KIF14/CDK1/ECT2/CCL19/ADAM9/ITGB1BP1/IRAK2/PBK/GPRC5A/IL23A/TPX2/ADORA2B/ST

K3/ADCY4/MAP2K1/ERN1/ANG/NCKAP1L/TNFSF11/KSR1/NOD2/TGFBR1/SHC2/PDGFC/MAPKAPK2/MALT1/HACD3/LRRK2/PTPN1/TGFB3/SYK/CLSPN/AKT1/PARK7/MAP3K11/IL18/UBE2N/PRKAR1A/ADCY7/FGF2/COPS8/ADRB2

GOBP_OSTEOCLAST_DIFFERENTIATION GOBP_OSTEOCLAST_DIFFERENTIATION GOBP_OSTEOCLAST_DIFFERENTIATION 89 0.473099622 1.50387418 0.014265335 0.088357021 0.069927023 2126 tags=35%, list=13%, signal=31% FBN1/TCIRG1/CCR1/SBNO2/GPR137B/CEBPB/PIAS3/MAFB/TYROBP/CD81/SNX10/RASSF2/EFNA2/LTF/CLDN18/CSF1R/GPR183/SLC9B2/ANXA2/TFE3/IL23A/CD109/LILRB4/SFRP1/LILRB1/IFNG/LILRB3/CREB1/TMEM64/TNFSF11/INPP5D

GOBP_REGULATION_OF_HEMATOPOIETIC_PROGENITOR_CELL_DIFFERENTIATION GOBP_REGULATION_OF_HEMATOPOIETIC_PROGENITOR_CELL_DIFFERENTIATION GOBP_REGULATION_OF_HEMATOPOIETIC_PROGENITOR_CELL_DIFFERENTIATION 85 0.492186176 1.554519269 0.014306152 0.088500571 0.070040631 4710 tags=52%, list=28%, signal=38% CBFB/PSME4/PSMB9/LMO2/PSMD14/PSMD12/PSMC2/PSMB2/PSMA3/METTL3/PSMD1/TCF3/PSMA5/PSMD6/PSMB5/PSMC4/PUS7/PSMB1/PSMA1/PSME3/PSMA7/PSMB8/PSMC5/PSMB10/PSMB6/PSMA4/LDB1/PSMD13/PSME1/PSMC1/PSMB4/PSMB3/PSMD2/PSMC6/PSMD7/DHX36/PSMA2/PSMD4/GATA2/PSMD9/EIF2AK2/PSMB7/PRKDC/PDCD2

GOBP_PROTEIN_LOCALIZATION_TO_CHROMOSOME GOBP_PROTEIN_LOCALIZATION_TO_CHROMOSOME GOBP_PROTEIN_LOCALIZATION_TO_CHROMOSOME 88 0.472215952 1.499067467 0.014326648 0.088518215 0.070054595 5300 tags=57%, list=31%, signal=39% NDC80/VCP/CDCA5/EZH2/CDK1/MIS12/AURKB/BUB1B/ZWILCH/ACD/PLK1/ATR/KNL1/CCT3/RCC2/CCT8/TRAPPC12/CCT5/CCT2/CCT6A/CCT4/CCT7/PINX1/CDT1/RUVBL2/NABP2/SPDL1/MACROH2A2/PIH1D1/TNKS2/XRCC5/TINF2/IFFO1/CENPT/TERF2IP/BUB3/TTK/MRNIP/ATM/CHMP7/HASPIN/ZW10/CENPA/PPHLN1/TPP1/PARP3/GNL3/MCM8/BOD1/RPA2

GOBP_CELL_JUNCTION_DISASSEMBLY GOBP_CELL_JUNCTION_DISASSEMBLY GOBP_CELL_JUNCTION_DISASSEMBLY 20 0.647171426 1.60876057 0.014354067 0.088578541 0.070102338 3610 tags=60%, list=21%, signal=47% C3/MAP4K4/C1QB/SNAI2/ITGAM/C1QA/DKK1/TGFBR1/TGFB3/CX3CL1/EPHA4/DUSP3

GOBP_POSITIVE_REGULATION_OF_ENDOTHELIAL_CELL_PROLIFERATION GOBP_POSITIVE_REGULATION_OF_ENDOTHELIAL_CELL_PROLIFERATION GOBP_POSITIVE_REGULATION_OF_ENDOTHELIAL_CELL_PROLIFERATION 86 0.488264954 1.542488479 0.014450867 0.089066339 0.070488388 3362 tags=41%, list=20%, signal=33%

CCL11/WNT5A/F3/MYDGF/APLNR/ECM1/HIF1A/CAV2/CCL24/VIP/VEGFC/KDR/TEK/FGFR1/LRG1/PIK3CD/NRP1/PPP1R16B/EGR3/AKT3/FGFBP1/ANG/EGFL7/TGFBR1/WNT2/MDK/BMP6/AKT1/ITGA4/JCAD/FGF2/STAT5A/CDH13/PRKD1/HMGB1

GOBP_INOSITOL_METABOLIC_PROCESS GOBP_INOSITOL_METABOLIC_PROCESS GOBP_INOSITOL_METABOLIC_PROCESS 11 -0.717750432 -1.65480366 0.014492754 0.089214902 0.070605963 2261 tags=55%, list=13%, signal=47% ISYNA1/SLC5A3/IMPA2/PPIP5K2/IMPA1/ITPKA

GOBP_CELLULAR_MONOVALENT_INORGANIC_CATION_HOMEOSTASIS GOBP_CELLULAR_MONOVALENT_INORGANIC_CATION_HOMEOSTASIS

GOBP_CELLULAR_MONOVALENT_INORGANIC_CATION_HOMEOSTASIS 108 -0.370475673 -1.382670829 0.014652015 0.090040453 0.071259316 3407 tags=31%, list=20%, signal=25% ATP1A2/ATP6V1D/ATP1B1/ATP6V0D1/SLC9A1/ATP1A1/ATP12A/SLC12A2/DMXL2/CHP1/RAB20/CLN3/MAPK3/CLN5/MAPK1/SLC9A8/ATP5F1B/RAB7A/ATP6V0D2/CA7/BCL2/NEDD4L/CCDC115/CFTR/LACC1/ATP1B3/KCNJ2/TTPA/CA2/SLC26A3/SLC9A2/AQP11/SLC9A3/SLC4A4

GOBP_DEFENSE_RESPONSE_TO_GRAM_NEGATIVE_BACTERIUM GOBP_DEFENSE_RESPONSE_TO_GRAM_NEGATIVE_BACTERIUM GOBP_DEFENSE_RESPONSE_TO_GRAM_NEGATIVE_BACTERIUM 68 0.49498889 1.517150395 0.014662757 0.090040453 0.071259316 1908 tags=22%, list=11%, signal=20% DEFA5/DEFA6/DMBT1/CXCL6/NOS2/SERPINE1/SELP/S100A7/RNASE6/LTF/LYZ/IL23A/IL6/MR1/CTSG

GOBP_INTERLEUKIN_12_PRODUCTION GOBP_INTERLEUKIN_12_PRODUCTION GOBP_INTERLEUKIN_12_PRODUCTION 58 0.510970274 1.532002344 0.014749263 0.090460942 0.071592097 3111 tags=45%, list=18%, signal=37% IDO1/JAK3/RIPK2/CLEC7A/TLR8/CCR7/TLR2/CD40/LAPTM5/IRF1/PLCB1/CCL19/CD47/IL23A/LILRB1/IFNG/RELA/LTB/NOD2/IL16/CMKLR1/MDK/SYK/ARRB2/IRAK3/HSPD1

GOBP_RESPONSE_TO_PEPTIDE_HORMONE GOBP_RESPONSE_TO_PEPTIDE_HORMONE GOBP_RESPONSE_TO_PEPTIDE_HORMONE 417 0.350266048 1.32146528 0.01489118 0.09121984 0.0721927 1183 tags=13%, list=7%, signal=13% REG1A/REG1B/REG3A/TIMP1/IGFBP5/GJA1/ANXA1/IL1B/RHOQ/MZB1/SPARC/COL1A1/RAB31/TFF1/CAV1/LYN/PKM/ASS1/FBN1/PIK3R3/LPIN1/JAK3/LPL/GRB10/RAB12/GAL/TCIRG1/PRKAR2B/SOCS3/XBP1/PLN/UCP2/APOBEC1/FYN/JAK2/PPAT/STAT1/CAV2/TRIM16/TLR2/GNAI2/ICAM1/HHEX/BAIAP2L1/RAB8B/OSBPL8/PCSK9/RAB13/STAT4/IRS1/AGTRAP/TEK/STAT3/CDC6/INSIG1/USO1

GOBP_CELL_GROWTH GOBP_CELL_GROWTH GOBP_CELL_GROWTH 440 0.345033761 1.306280393 0.014942529 0.091422764 0.072353297 3019 tags=22%, list=18%, signal=19% REG1A/S100A8/IGFBP5/GREM1/GJA1/S100A9/SPP1/AGT/WNT5A/CD38/IGFBP7/SERPINE2/HYAL1/TGFB1/PLXNA1/INHBA/ALCAM/MFSD2A/FN1/TRIM40/TGFBR2/BST2/MAD2L2/CXCR4/DCUN1D5/POSTN/TTL/HYAL2/SEMA4D/PPT1/MYOCD/BCL6/RGS2/ENO1/WFDC1/RASAL1/KIF14/TMC8/FLRT3/RGS4/SEMA4B/SPHK1/OLFM1/AURKA/IGFBP4/CYP27B1/NRP1/LAMB2/MACF1/TRPV2/SPOCK1/ADAM15/SDCBP/VCL/CDKN2C/PML/FHL1/CRYAB/CCDC85B/SFRP1/MAP1B/SEMA3G/EIF4G1/KDM2B/DERL2/SH3BP4/JADE1/PTPRS/TGFBR1/SFRP2/SEMA4A/PSRC1/CXCL16/EXOSC9/PLXNA3/PARP2/FXN/HSP90AB1/CRLF3/SEMA3F/AKT1/LLPH/FAM107A/DCLK1/ITGA4/DPYSL2/NRCAM/KLHL22/LIMK1/DVL1/CLSTN3/APOE/RASGRP2/SMURF1/HSP90AA1/PPP1R9B/CCAR2/SEMA4F/CTTN

GOBP_NEGATIVE_REGULATION_OF_PHOSPHORYLATION GOBP_NEGATIVE_REGULATION_OF_PHOSPHORYLATION GOBP_NEGATIVE_REGULATION_OF_PHOSPHORYLATION 368 0.352837518 1.32360899 0.015116279 0.092373169 0.073105461 3610 tags=34%, list=21%, signal=27% GREM1/WARS1/IL1B/AGT/TRIB2/CAV1/SERPINB3/LYN/ENG/HEG1/LAX1/TGFB1/TIGAR/NIBAN1/SH3BP5/INHBA/PTPRC/PRKAR2B/SOCS3/PMEPA1/DNAJC10/CCNB1/DNAJC3/DUSP4/DUSP14

/TWIST1/SAMSN1/THY1/GMFG/DUSP6/TRIM27/DDIT4/DUSP7/LATS2/CD300A/HHEX/HYAL2/SEMA4D/MYOCD/RHOH/SIRPA/RASSF2/RGS2/PARP14/EMILIN1/DNAJA1/XDH/WWTR1/SPRED1/DKK1/RGS4/NUP62/TESC/ITGB1BP1/SPINK1/DUSP10/PLK1/PBK/PRNP/PIP4K2A/GPRC5A/IBTK/CORO1C/DUSP22/ITPRIP/CDKN2C/AJUBA/CD109/LILRB4/SFRP1/PKIA/IFNG/CDKN1C/CHORDC1/EIF4G1/RUBCN/SFRP2/STRAP/PARVA/LRRK2/PTPN1/FOXA2/ADAR/UCHL1/MICAL1/CDK5RAP3/AKT1/RGS3/PARK7/NPM1/MLLT1/PRKAR1A/ARRB2/DVL1/FBLN1/PWP1/APOE/ROCK1/PPM1F/TARDBP/CBFA2T3/SOCS4/IRAK3/SPRY1/GSTP1/NR2F2/RASIP1/ERRFI1/CDK5RAP1/NF2/YWHAG/AIDA/PKN1/FKBP8/TARBP2/IPO7/EPHA1/BAK1/CALM3/PKIG/PPIF/ANKLE2/PTPN13/DUSP3

GOBP_HISTONE_H3_K14_ACETYLATION GOBP_HISTONE_H3_K14_ACETYLATION GOBP_HISTONE_H3_K14_ACETYLATION 14 -0.681878727 -1.682657766 0.015228426 0.092875198 0.073502774 2285 tags=43%, list=13%, signal=37% WBP2/KAT7/SIRT1/BRPF3/NAP1L2/PIWIL2

GOBP_PROTEIN_LOCALIZATION_TO_ENDOPLASMIC_RETICULUM GOBP_PROTEIN_LOCALIZATION_TO_ENDOPLASMIC_RETICULUM GOBP_PROTEIN_LOCALIZATION_TO_ENDOPLASMIC_RETICULUM 123 0.428822184 1.435441022 0.015235457 0.092875198 0.073502774 3290 tags=23%, list=19%, signal=18% GJA1/KDELR3/SRPRB/HSPA5/SSR3/SPCS3/TRAM2/SRPRA/SRP19/SEC61A1/SRP68/INSIG1/SRP72/SEC61B/SEC16A/SRP54/SGTB/SEC61G/SPCS2/EDEM1/KDELR2/LRRK2/TRAM1/KDELR1/SPCS1/SEC62/SEC63/HERPUD1

GOBP_NEGATIVE_REGULATION_OF_SYNAPSE_ORGANIZATION GOBP_NEGATIVE_REGULATION_OF_SYNAPSE_ORGANIZATION GOBP_NEGATIVE_REGULATION_OF_SYNAPSE_ORGANIZATION 10 0.761977853 1.621571174 0.015254237 0.092876831 0.073504066 717 tags=20%, list=4%, signal=19% WNT5A/TLR2

GOBP_PHOTOTRANSDUCTION GOBP_PHOTOTRANSDUCTION GOBP_PHOTOTRANSDUCTION 54 -0.481797115 -1.568649374 0.01529052 0.092891132 0.073515384 430 tags=9%, list=3%, signal=9% GNA11/CNGA1/NMT2/OPN3/PDE6A

GOBP_REGULATION_OF_GAMMA_DELTA_T_CELL_ACTIVATION GOBP_REGULATION_OF_GAMMA_DELTA_T_CELL_ACTIVATION GOBP_REGULATION_OF_GAMMA_DELTA_T_CELL_ACTIVATION 11 0.739563124 1.588489822 0.015306122 0.092891132 0.073515384 3787 tags=82%, list=22%, signal=64% PTPRC/LEF1/EGR3/LILRB1/NCKAP1L/NOD2/SYK/STAT5B/TCF7

GOBP_MUSCLE_CONTRACTION GOBP_MUSCLE_CONTRACTION GOBP_MUSCLE_CONTRACTION 334 0.362821532 1.352414871 0.015312132 0.092891132 0.073515384 2030 tags=17%, list=12%, signal=15% GJA1/KCND3/PROK2/CD38/CAV1/KCNE3/ITGA2/ADA/GUCY1A1/EDNRA/TPM4/PDE4B/SULF1/STC1/ACTG2/F2R/PPP1R12B/HOMER1/PLN/TPM2/VIM/KIT/ANXA6/CXCR4/CALCRL/ACTA2/CALD1/MYL9/PTAFR/SULF2/MYOCD/DOCK4/RGS2/FLNA/ASPH/CLIC2/ENO1/NMU/PIK3CG/PABPN1/SPHK1/CNN1/KCNJ8/GSTO1/CHRM3/MYOF/VCL/RAP1GDS1/RANGRF/CRYAB/ATP2B4/TLN1/SMTN/DES/ATP2A2/SMAD5

GOBP_FATTY_ACID_DERIVATIVE_CATABOLIC_PROCESS GOBP_FATTY_ACID_DERIVATIVE_CATABOLIC_PROCESS

GOBP_FATTY_ACID_DERIVATIVE_CATABOLIC_PROCESS16 -0.661121688 -1.689102373 0.015345269 0.092979725 0.073585498 829 tags=50%, list=5%, signal=48% FITM2/CYP4F3/BDH1/CYP4F12/CYP4F2/NUDT7/ACAT1/HPGD
GOBP_RAS_PROTEIN_SIGNAL_TRANSDUCTION GOBP_RAS_PROTEIN_SIGNAL_TRANSDUCTION GOBP_RAS_PROTEIN_SIGNAL_TRANSDUCTION 325 0.361026875 1.342666238 0.015402844 0.093216003 0.073772492 3287 tags=31%, list=19%, signal=26% COL1A2/ROBO1/F2RL2/COL3A1/LYN/HEG1/EPS8L1/RAC2/PDPN/RAB12/F2R/JAK2/DLC1/ARHGEF3/ARHGAP29/MAP4K4/ARHGDIB/PDGFRB/MET/P2RY8/BCR/STMN3/RHOH/FOXM1/BCL6/CTNNAL1/CDC42EP1/VAV1/RASGRF2/RASGRP3/RASAL1/KIF14/PIK3CG/CYTH4/RIPOR2/ALS2/NUP62/LRRC59/NRP1/RALGPS2/ITPKB/ELMO1/GPR65/RAP2A/ARHGDIA/RANGRF/RHOG/RASSF1/CDK2/ARHGEF2/ARHGAP24/DENND4B/AIF1/CELSR1/KPNB1/RDX/TIMP2/GPR4/PLK2/KSR1/CDC42SE1/SHC2/RGL2/HACD3/OGT/GNA12/RIPOR1/DNMT1/RASGRP1/RHOJ/TRIM28/RASAL3/PARK7/CDC42EP2/WAS/ARFGAP1/SGSM3/LIMK1/SSX2IP/RHOD/APOE/ROCK1/FGF2/CCNA2/RASGRP2/ARHGEF10/SPRY1/HRAS/ITGA3/RASIP1/LZTR1/RAPGEF3/G3BP1/ABI2/TNFAIP1/LAT/CDH13/RASA3/SHC1/WASF2/PRKD1/RIT1
GOBP_ENDOSOME_ORGANIZATION GOBP_ENDOSOME_ORGANIZATION GOBP_ENDOSOME_ORGANIZATION 71 -0.429136951 -1.477967021 0.015479876 0.093569324 0.074052115 2567 tags=34%, list=15%, signal=29% EXOC8/IST1/SYNJ1/STAM2/VPS36/USP8/VPS28/SQSTM1/RAB7A/CHMP2A/CHMP4C/HOOK2/RILP/SCARB2/VPS4B/TMEM127/VTA1/PDCD6IP/DNAJC13/HOOK1/CHMP5/CHMP1B/CHMP4B/AQP11
GOBP_LEUKOCYTE_TETHERING_OR_ROLLING GOBP_LEUKOCYTE_TETHERING_OR_ROLLING GOBP_LEUKOCYTE_TETHERING_OR_ROLLING 30 0.596557071 1.601487697 0.015698587 0.09477715 0.075008006 1779 tags=33%, list=11%, signal=30% SELL/SELP/ST3GAL4/VCAM1/MADCAM1/SELE/PTAFR/JAM2/SELPLG/FUT4
GOBP_POSITIVE_REGULATION_OF_PHOSPHATIDYLINOSITOL_3_KINASE_SIGNALING GOBP_POSITIVE_REGULATION_OF_PHOSPHATIDYLINOSITOL_3_KINASE_SIGNALING GOBP_POSITIVE_REGULATION_OF_PHOSPHATIDYLINOSITOL_3_KINASE_SIGNALING 87 0.474156652 1.505258472 0.015736767 0.09489346 0.075100056 1518 tags=25%, list=9%, signal=23% AGT/LYN/SELP/HCLS1/PIK3AP1/MYDGF/F2R/FN1/KIT/PDGFRA/FYN/JAK2/DCN/FGR/PDGFRB/SEMA4D/KDR/TEK/PIK3CG/FGFR1/PIK3CD/PIK3R5
GOBP_DNA_RECOMBINATION GOBP_DNA_RECOMBINATION GOBP_DNA_RECOMBINATION 278 0.365390413 1.33970741 0.015815085 0.095161837 0.075312453 4820 tags=39%, list=28%, signal=28% IL7R/TGFB1/FEN1/IL27RA/PTPRC/TRIP13/TNFSF13/NABP1/MAD2L2/KPNA2/SAMHD1/CD40/RTEL1/H2AX/BCL6/LEF1/BLM/MCM6/PSMD14/CDC45/RUVBL1/CCR6/TCF3/FANCD2/EXOSC3/EXO1/MSH6/CDC7/PARP1/SFR1/TIMELESS/AUNIP/MLH1/BATF/NBN/SETX/MCM3/RBBP8/FIGNL1/MCM5/SWAP70/CHEK1/RAD51C/RUVBL2/POLM/PARPBP/NABP2/THOC1/ARID2/CGAS/POLB/MCM7/RECQL/WAS/UBE2N/PIF1/RNF8/RAD51D/EME1/HSPD1/GINS2/SFPQ/ALYREF/SLC15A4/RAD54L/BRCA1/CENPX/HMGB1/INO80E/C11orf80/ACTR5/FBH1/XRCC5/MCM2/RAD51AP1/FUS/UBQLN4/REC8/PGBD5/TERF2IP/KPNA1/TCF7/PAGR1/PALB2/TOP2A/SUPT6H/MRE11/IL4/CLCF1/RAD54B/MRNIP/MAGEF1/ATM/AP5S1/XRCC1/RPA3/NONO/MMS19/TFPT/PRKDC/MLH3/DCLRE1C/TP53BP1/GEN1/DCAF1/CORT/WRN/MUS81

GOBP_REGULATION_OF_VESICLE_MEDIATED_TRANSPORT
GOBP_REGULATION_OF_VESICLE_MEDIATED_TRANSPORT
GOBP_REGULATION_OF_VESICLE_MEDIATED_TRANSPORT 484 0.337764187 1.287304724 0.015819209 0.095161837 0.075312453 3470 tags=28%, list=20%, signal=23% GREM1/IL13RA2/ANXA1/IL1B/WNT5A/RAB31/CAV1/MSN/C3/LYN/ITGA2/SERPINE1/C2/CCR2/GAS1/MCTP1/RAC2/STXBP1/RAB12/PTPRC/CLU/FCER1G/ADGRE2/CLEC7A/ITGB2/FCGR2B/VSNL1/PREB/FOXF1/FGR/SELE/TLR2/GNAI2/RIMS3/HCK/PTAFR/CCL2/CD300A/RAB8B/PPT1/PLCG2/PCSK9/LYAR/RAB13/CORO1A/SIRPA/STXBP6/ITGAM/DAB2/CALR/RAB29/APOC1/ANO6/CD84/DKK1/DOCK2/CCL19/SPHK1/PRKCB/WDR54/NRP1/ATP13A2/IL15RA/SAR1A/MFGE8/ANXA2/CADPS2/CD47/ITGAV/SDCBP/STON1/BET1L/EHD2/AHI1/DNAJC6/CD300LF/AXL/LILRB1/MAP2K1/IFNG/SH3GL1/FPR2/RDX/ATP2A2/RAB3D/NCKAP1L/PPP3CA/RUBCN/NOD2/SYT11/LRRK2/DYSF/BLK/PTPN1/CLIP3/SYT12/SIRPG/SYK/MICAL1/SEPTIN1/CHRNA5/BACE1/DLL1/HIP1/ACSL3/YIPF5/ARFGAP1/PLA2G3/ARRB2/DVL1/APOE/ROCK1/FES/SCFD1/AP1G1/PIP4P2/GRIN3A/CPLX1/RAB27B/CADPS/PLSCR1/CDH13/AP2B1/RAB27A/HYAL3/PRKD1/VPS4A/BIN1/MBTPS1/HMGB1/DRD4/ABL2/RINT1/PTPN23/LRPAP1/CALM3
GOBP_CELL_DEATH_IN_RESPONSE_TO_OXIDATIVE_STRESS
GOBP_CELL_DEATH_IN_RESPONSE_TO_OXIDATIVE_STRESS
GOBP_CELL_DEATH_IN_RESPONSE_TO_OXIDATIVE_STRESS 78 0.4836234 1.5101879 0.015895954 0.095508982 0.075587189 2630 tags=28%, list=16%, signal=24% MMP3/VNN1/SLC7A11/HIF1A/FYN/JAK2/MAP3K5/MET/P4HB/NME5/MELK/PDK1/PML/PARP1/LRRK2/SOD2/GPX1/FBXW7/ATF4/AKT1/PYCR1/PARK7
GOBP_REGULATION_OF_HETEROTYPIC_CELL_CELL_ADHESION
GOBP_REGULATION_OF_HETEROTYPIC_CELL_CELL_ADHESION
GOBP_REGULATION_OF_HETEROTYPIC_CELL_CELL_ADHESION 25 0.598015808 1.551552198 0.015923567 0.095560449 0.07562792 1216 tags=20%, list=7%, signal=19% IL1RN/IL1B/CD44/THY1/LCK
GOBP_REGULATION_OF_CELL_CELL_ADHESION_MEDIATED_BY_CADHERIN
GOBP_REGULATION_OF_CELL_CELL_ADHESION_MEDIATED_BY_CADHERIN
GOBP_REGULATION_OF_CELL_CELL_ADHESION_MEDIATED_BY_CADHERIN 20 0.64425417 1.601508757 0.015948963 0.095598505 0.075658039 2846 tags=40%, list=17%, signal=33% WNT5A/RGCC/MAD2L2/ADAM19/SERPINF2/FOXA2/BMP6/PPM1F
GOBP_LATE_ENDOSOME_TO_VACUOLE_TRANSPORT
GOBP_LATE_ENDOSOME_TO_VACUOLE_TRANSPORT
GOBP_LATE_ENDOSOME_TO_VACUOLE_TRANSPORT 21 -0.622152408 -1.658774827 0.016 0.095675805 0.075719215 3050 tags=52%, list=18%, signal=43% LEPROT/BECN1/TMEM50A/VPS36/VPS28/CHMP2A/CHMP4C/VTA1/CHMP5/CHMP1B/CHMP4B
GOBP_RESPONSE_TO_CORTICOSTERONE GOBP_RESPONSE_TO_CORTICOSTERONE
GOBP_RESPONSE_TO_CORTICOSTERONE 20 -0.636657798 -1.68296947 0.016 0.095675805 0.075719215 1007 tags=20%, list=6%, signal=19% HSD3B1/ATP2B1/HSD3B2/FOSB
GOBP_FOCAL_ADHESION_ASSEMBLY GOBP_FOCAL_ADHESION_ASSEMBLY
GOBP_FOCAL_ADHESION_ASSEMBLY 83 0.4739255 1.484256971 0.016058394

0.095910671	0.075905091	3227	tags=39%,	list=19%,	signal=31%
GREM1/FERMT2/ITGA2/DLC1/THY1/MAP4K4/ACTN1/BCR/TRIP6/KDR/COL16A1/TEK/LIMCH1/ITGB1BP1/NRP1/MACF1/CORO1C/DUSP22/VCL/AJUBA/RCC2/SFRP1/APOD/PHLDB2/WNT4/FAM107A/RHOD/ROCK1/PPM1F/CTTN/GPM6B/LDB1
GOBP_REGULATION_OF_GTPASE_ACTIVITY	GOBP_REGULATION_OF_GTPASE_ACTIVITY
GOBP_REGULATION_OF_GTPASE_ACTIVITY	440	0.34183567	1.294172582	0.016091954
0.095996829	0.075973278	3603	tags=30%,	list=21%,	signal=25%
CCL11/CXCL13/RGS5/CCL18/WNT5A/ARFGAP3/FERMT2/TAGAP/PLXNA1/CCL20/F2R/TBC1D8/ASAP1/CHN1/FICD/RGS18/CCR7/GPR137B/CCL4/DLC1/THY1/ARHGAP29/CAV2/PREB/MAP4K4/ARHGDIB/ICAM1/CCL2/SEMA4D/MET/CCL24/PLXNC1/BCR/TBC1D16/DOCK4/STMN3/CD40/RHOH/BCL6/RGS2/SMAP2/GNB5/EIF2S1/CCL22/S1PR1/ARHGAP9/VAV1/RASGRP3/RASAL1/LRCH1/EZH2/RIPOR2/PLCB1/DOCK11/ALS2/DOCK2/TBC1D2B/RGS4/ECT2/CCL19/ITGB1BP1/NRP1/ITGA6/SEC23B/ARHGAP25/ARHGAP31/ARAP3/CORO1C/ARHGAP23/RANGAP1/SBF1/GPR65/AJUBA/PLXND1/RAP1GDS1/ARHGDIA/RANBP1/RCC2/RHOG/SFRP1/ARHGAP24/FGD2/AGRN/RDX/ARHGEF6/SH3BP4/WNT4/NCKAP1L/CDC42SE1/RRP1B/PLXNA3/DOCK9/HACD3/LRRK2/ACAP3/RASGRP1/FGD3/CCL7/RGS3/RASAL3/ARHGAP26/DOCK8/PKP4/CDC42EP2/ARFGAP1/SGSM3/ARRB2/GPSM1/RASGRP2/ARHGEF10/SPRY1/HRAS/SYDE1/TBC1D10C/GMIP/RASIP1/RAPGEF3/RACGAP1/ERRFI1/RASA3/TBCD/AGAP3/DEPDC1/CX3CL1/SGSM2/ACAP1/EPHA4/EPHA1/PLXNB2/GDI1/ADPRH/RGS17/DVL3/BNIP2
GOBP_NEGATIVE_REGULATION_OF_RESPONSE_TO_CYTOKINE_STIMULUS
GOBP_NEGATIVE_REGULATION_OF_RESPONSE_TO_CYTOKINE_STIMULUS
GOBP_NEGATIVE_REGULATION_OF_RESPONSE_TO_CYTOKINE_STIMULUS	61	0.501365764
1.511282925	0.016176471	0.096386405	0.076281594	1721	tags=23%,	list=10%,	signal=21%
IL1RN/MMP12/CAV1/ROBO1/PXDN/ECM1/PTPRC/CARD16/NLRC5/SAMHD1/PARP14/METTL3/CCDC3/IL6
GOBP_MICROTUBULE_CYTOSKELETON_ORGANIZATION_INVOLVED_IN_MITOSIS
GOBP_MICROTUBULE_CYTOSKELETON_ORGANIZATION_INVOLVED_IN_MITOSIS
GOBP_MICROTUBULE_CYTOSKELETON_ORGANIZATION_INVOLVED_IN_MITOSIS	138
0.417085529	1.422126289	0.016304348	0.097033112	0.076793407	3888
tags=40%,	list=23%,	signal=31%
GJA1/INSC/EML1/CCNB1/TUBG1/CDC20/PRC1/NDC80/FLNA/VCP/TACC3/NUP62/CENPE/AURKB/AURKA/NUSAP1/EFHC1/PLK1/RAN/DLGAP5/KIF23/TPX2/MZT1/INTS13/NUDC/KPNB1/MYBL2/BCCIP/PLK2/KIF11/PSRC1/KIF4A/RAE1/CCSAP/STIL/SPDL1/MAP9/NDE1/EML3/ARHGEF10/SPRY1/TACC1/RACGAP1/TUBG2/DCTN6/NUF2/SAPCD2/AAAS/SPC25/MAP4/ZNF207/RCC1/TTK/PTPA/MAD2L1
GOBP_CELL_MIGRATION_INVOLVED_IN_SPROUTING_ANGIOGENESIS
GOBP_CELL_MIGRATION_INVOLVED_IN_SPROUTING_ANGIOGENESIS
GOBP_CELL_MIGRATION_INVOLVED_IN_SPROUTING_ANGIOGENESIS	52	0.530507718
1.565635568	0.016344725	0.097043179	0.076801374	2852	tags=46%,	list=17%,	signal=38%
GREM1/ANXA1/ROBO1/PIK3R3/PTGS2/SRPX2/MMRN2/KDR/SPRED1/ITGB1BP1/CLEC14A/NRP1/HDAC7/EGR3/EPHB4/AKT3/FGFBP1/PLK2/RHOJ/FBXW7/AKT1/JCAD/EFNB2/FGF2
GOBP_REGULATION_OF_MYELOID_LEUKOCYTE_MEDIATED_IMMUNITY

GOBP_REGULATION_OF_MYELOID_LEUKOCYTE_MEDIATED_IMMUNITY
GOBP_REGULATION_OF_MYELOID_LEUKOCYTE_MEDIATED_IMMUNITY 52 0.523724359 1.545616505 0.016344725 0.097043179 0.076801374 1489 tags=37%, list=9%, signal=33%
CXCL6/IL13RA2/C3/LYN/CCR2/RAC2/STXBP1/ADGRE2/ITGB2/FCGR2B/DDX21/FOXF1/FGR/PTAFR/CD300A/TYROBP/ITGAM/CD84/BTK
GOBP_POST_ANAL_TAIL_MORPHOGENESIS GOBP_POST_ANAL_TAIL_MORPHOGENESIS GOBP_POST_ANAL_TAIL_MORPHOGENESIS 14 0.702404111 1.60741373 0.016447368 0.097537172 0.077192328 3920 tags=57%, list=23%, signal=44% WNT5A/CHST11/SP5/DCHS1/SFRP2/TCF15/PALB2/TMED2
GOBP_REGULATION_OF_OSTEOBLAST_DIFFERENTIATION
GOBP_REGULATION_OF_OSTEOBLAST_DIFFERENTIATION
GOBP_REGULATION_OF_OSTEOBLAST_DIFFERENTIATION 113 0.441332753 1.460135725 0.016551724 0.098040142 0.077590385 2289 tags=28%, list=14%, signal=25% CTHRC1/IGFBP5/GREM1/FERMT2/CCN1/IFITM1/TCIRG1/RUNX2/TWIST1/CEBPB/SEMA4D/RASSF2/SNAI2/DDR2/VEGFC/LTF/WWTR1/IL6ST/FFAR4/TWSG1/HDAC7/ACVR1/IL6/SFRP1/JAG1/TMEM119/SMAD5/WNT4/TMEM64/RIOX1/SFRP2/NPNT
GOBP_ACID_SECRETION GOBP_ACID_SECRETION GOBP_ACID_SECRETION 37 -0.530282275 -1.604882778 0.016806723 0.099433169 0.078692848 1764 tags=22%, list=10%, signal=19% APBA1/SLC22A16/PRKAA1/SGK1/SLC9A3R1/ABCB11/SLC51B/SLC51A
GOBP_NEGATIVE_REGULATION_OF_HEMOPOIESIS
GOBP_NEGATIVE_REGULATION_OF_HEMOPOIESIS
GOBP_NEGATIVE_REGULATION_OF_HEMOPOIESIS 96 0.464990918 1.499474713 0.016853933 0.099595029 0.078820946 2549 tags=30%, list=15%, signal=26% ANXA1/LYN/FBN1/CD74/JAK3/INHBA/NME1/CBFB/FCGR2B/GPR137B/SHH/PIAS3/MAFB/BCL6/LTF/IRF1/CLDN18/RUNX3/LAG3/LILRB4/TMEM131L/SFRP1/LILRB1/LILRB3/INPP5D/HLX/FSTL3/MDK/FBXW7
GOBP_NEGATIVE_REGULATION_OF_MITOTIC_CELL_CYCLE
GOBP_NEGATIVE_REGULATION_OF_MITOTIC_CELL_CYCLE
GOBP_NEGATIVE_REGULATION_OF_MITOTIC_CELL_CYCLE 291 0.365678532 1.346737466 0.016949153 0.099922325 0.079079972 3908 tags=40%, list=23%, signal=31% RGCC/GAS1/TGFB1/TRIP13/PSME4/PSMB9/CCNB1/NABP1/MAD2L2/SLFN11/FAP/CCL2/BUB1/CDC20/DACT1/DONSON/NDC80/BCL6/MAP3K20/BLM/PSMD14/CDC6/EZH2/PSMD12/CDK1/PSMC2/MUC1/PSMB2/AURKB/DDIAS/PSMA3/AURKA/PSMD1/BUB1B/FANCD2/ZWILCH/CCND1/PLK1/PKD2/PRMT1/TIPIN/PSMA5/CDKN2C/AVEN/PML/FHL1/TNKS1BP1/CDK2/ZWINT/PSMD6/CNOT9/CDKN1C/GPNMB/PSMB5/PSMC4/TIMP2/PLK2/JADE1/NBN/ARID3A/PSMB1/PSMA1/CDK4/PINX1/CHEK1/PSME3/CLSPN/CDT1/CDK5RAP3/NABP2/FAM107A/SPDL1/CNOT6L/CUL1/PSMA7/PSMB8/PSMC5/MDC1/KLHL22/TRIAP1/NACC2/KNTC1/PSMB10/PSMB6/EME1/PSMA4/FBXO5/INTS3/PCBP4/PNPT1/PSMD13/PSME1/VPS4A/BRCA1/USP47/RINT1/PSMC1/ZNF830/FBXL7/MDM2/ZFP36L1/ZNF207/PSMB4/CENPF/PSMB3/PSMD2/BUB3/TTK/KANK2/NEK11/CHFR/PSMC6/MAD2L1/PSMD7/DYNC1LI1
GOBP_T_CELL_MEDIATED_CYTOTOXICITY GOBP_T_CELL_MEDIATED_CYTOTOXICITY GOBP_T_CELL_MEDIATED_CYTOTOXICITY 42 0.542422064 1.545733855 0.016949153 0.099922325 0.079079972 3745 tags=43%, list=22%, signal=33%

IL7R/CTSH/AZGP1/HLA-DRA/PTPRC/FCGR2B/CTSC/IL12RB1/IL23A/MR1/LILRB1/HPRT1/PVR/EMP2/RAB27A/NECTIN2/HLA-E/FADD
KEGG_CYTOKINE_CYTOKINE_RECEPTOR_INTERACTION
KEGG_CYTOKINE_CYTOKINE_RECEPTOR_INTERACTION
KEGG_CYTOKINE_CYTOKINE_RECEPTOR_INTERACTION 250 0.581330124 2.126127811
0.001219512 0.015220949 0.011025928 2250 tags=32%, list=13%, signal=28%
CXCL5/CXCL1/CXCL3/CXCL6/CCL11/CXCL9/CXCL11/CXCL13/CXCL8/CCL18/CXCL10/IL1B/CXCL2/CXCR2/CSF2RB/IL7R/TNFRSF17/CCR2/CCR10/TGFB1/PF4/INHBA/TNFRSF11B/CCL20/CCR1/TNFSF13/IL1R1/TNFSF13B/KIT/TGFBR2/PDGFRA/CCR7/CSF3R/CCL4/TNFRSF9/CXCR4/TNFRSF12A/CD27/CCL2/PDGFRB/MET/CCL24/IL10RA/CD40/TNFRSF10A/VEGFC/IL1A/KDR/CCL22/IL6ST/CCR6/TNFSF15/TNFRSF10B/CSF1R/IFNAR2/IL12RB1/PPBP/CCL19/IL18R1/PLEKHO2/TNFRSF1B/IL15RA/INHBC/ACVR1/CXCL14/CSF2RA/LIF/IL23A/IL6/TSLP/TNFRSF4/IL3RA/IFNG/CCR5/LTB/TNFSF11/TGFBR1/CXCL16/PDGFC/INHBB
KEGG_FOCAL_ADHESION KEGG_FOCAL_ADHESION KEGG_FOCAL_ADHESION 195
0.5016776 1.785911825 0.00128041 0.015220949 0.011025928 1874
tags=29%, list=11%, signal=26%
TNC/COL6A3/COL4A1/SPP1/VWF/COL1A2/COL1A1/CAV1/COL5A2/LAMC1/COL3A1/COL6A2/COL6A1/ITGA2/LAMC2/LAMA3/COL4A2/PIK3R3/BIRC3/RAC2/COL5A1/LAMA4/ZYX/FN1/ITGA5/PDGFRA/FYN/CAV2/MYL9/PDGFRB/MET/ACTN1/DIAPH1/FLNA/VEGFC/KDR/ITGA8/VAV1/PIK3CG/PRKCB/LAMB1/PIK3CD/ITGA9/CCND1/ITGA6/LAMB2/FLNC/PIK3R5/PARVB/ITGAV/VCL/AKT3/LAMA5/THBS2/MAP2K1/TLN1
KEGG_CHEMOKINE_SIGNALING_PATHWAY KEGG_CHEMOKINE_SIGNALING_PATHWAY
KEGG_CHEMOKINE_SIGNALING_PATHWAY 179 0.579948363 2.039076242 0.001285347
0.015220949 0.011025928 2199 tags=34%, list=13%, signal=29%
CXCL5/CXCL1/CXCL3/CXCL6/CCL11/CXCL9/CXCL11/CXCL13/CXCL8/CCL18/CXCL10/CXCL2/CXCR2/GRK5/LYN/CCR2/GNG11/PIK3R3/CCR10/PF4/JAK3/RAC2/CCL20/CCR1/CCR7/JAK2/CCL4/CXCR4/STAT1/FGR/GNAI2/HCK/CCL2/CCL24/GNB5/CCL22/STAT3/VAV1/GNB4/CCR6/PIK3CG/PLCB1/PPBP/DOCK2/CCL19/PRKCB/PIK3CD/PIK3R5/GNB2/ITK/CXCL14/ELMO1/AKT3/ADCY4/MAP2K1/STAT2/CCR5/RELA/CXCL16/SHC2
KEGG_CELL_ADHESION_MOLECULES_CAMS KEGG_CELL_ADHESION_MOLECULES_CAMS
KEGG_CELL_ADHESION_MOLECULES_CAMS 127 0.540533826 1.831412244 0.001345895
0.015220949 0.011025928 1602 tags=31%, list=9%, signal=29%
CD274/PECAM1/CLDN1/SELL/CDH3/CADM1/HLA-DMA/SELP/CDH5/HLA-DQB1/HLA-DRA/ALCAM/PTPRC/HLA-DMB/VCAN/VCAM1/MADCAM1/CLDN2/ITGB2/CD86/PTPRM/SELE/ICAM1/HLA-DQA1/CD40/HLA-DPA1/ICAM2/SDC2/ITGAM/ITGA8/JAM2/HLA-DOB/CD276/CLDN18/SELPLG/ITGA9/ITGA6/CD2/JAM3/ITGAV
KEGG_TOLL_LIKE_RECEPTOR_SIGNALING_PATHWAY
KEGG_TOLL_LIKE_RECEPTOR_SIGNALING_PATHWAY
KEGG_TOLL_LIKE_RECEPTOR_SIGNALING_PATHWAY 97 0.563113257 1.836811856
0.001422475 0.015220949 0.011025928 1861 tags=26%, list=11%, signal=23%
CXCL9/CXCL11/CXCL8/SPP1/CXCL10/IL1B/CTSK/PIK3R3/LY96/TLR8/CCL4/CD86/STAT1/TLR2/IRAK1/CD40/PIK3CG/IFNAR2/PIK3CD/TLR1/PIK3R5/AKT3/CASP8/IL6/MAP2K1
KEGG_SYSTEMIC_LUPUS_ERYTHEMATOSUS KEGG_SYSTEMIC_LUPUS_ERYTHEMATOSUS
KEGG_SYSTEMIC_LUPUS_ERYTHEMATOSUS 95 0.546894285 1.77301014 0.001428571

0.015220949 0.011025928 1063 tags=22%, list=6%, signal=21%
FCGR3B/FCGR2A/C1S/C3/HLA-DMA/C2/C1R/HLA-DQB1/HLA-DRA/HLA-DMB/FCGR2C/FCGR2B/CD86/C1QB/ACTN1/HLA-DQA1/CD40/HLA-DPA1/H2AX/C1QA/HLA-DOB
KEGG_ECM_RECEPTOR_INTERACTION KEGG_ECM_RECEPTOR_INTERACTION
KEGG_ECM_RECEPTOR_INTERACTION 81 0.652955629 2.08097622 0.001440922
0.015220949 0.011025928 2049 tags=42%, list=12%, signal=37%
TNC/COL6A3/COL4A1/SPP1/VWF/COL1A2/COL1A1/COL5A2/LAMC1/COL3A1/COL6A2/COL6A1/ITGA2/LAMC2/LAMA3/CD44/COL4A2/COL5A1/LAMA4/FN1/ITGA5/SDC2/ITGA8/LAMB1/ITGA9/ITGA6/LAMB2/CD47/ITGAV/LAMA5/THBS2/SDC3/AGRN/ITGB4
KEGG_LEISHMANIA_INFECTION KEGG_LEISHMANIA_INFECTION
KEGG_LEISHMANIA_INFECTION 65 0.672141757 2.084882437 0.001459854
0.015220949 0.011025928 1306 tags=37%, list=8%, signal=34%
FCGR3B/IL1B/NCF2/FCGR2A/NOS2/C3/HLA-DMA/TGFB1/HLA-DQB1/HLA-DRA/HLA-DMB/PTGS2/FCGR2C/ITGB2/JAK2/STAT1/TLR2/IRAK1/HLA-DQA1/HLA-DPA1/ITGAM/IL1A/HLA-DOB/PRKCB
KEGG_VIRAL_MYOCARDITIS KEGG_VIRAL_MYOCARDITIS KEGG_VIRAL_MYOCARDITIS 64
0.635095045 1.962093628 0.001461988 0.015220949 0.011025928 1714
tags=30%, list=10%, signal=27%
CD55/CAV1/HLA-DMA/HLA-DQB1/RAC2/HLA-DRA/HLA-DMB/FYN/ITGB2/CD86/ICAM1/HLA-DQA1/CD40/HLA-DPA1/HLA-DOB/CCND1/BID/MYH10/CASP8
KEGG_ANTIGEN_PROCESSING_AND_PRESENTATION
KEGG_ANTIGEN_PROCESSING_AND_PRESENTATION
KEGG_ANTIGEN_PROCESSING_AND_PRESENTATION 68 0.57785831 1.797271995
0.00147929 0.015220949 0.011025928 3013 tags=38%, list=18%, signal=32%
HLA-DMA/CD74/HLA-DQB1/HLA-DRA/HLA-DMB/TAP2/HSPA5/HSPA6/TAP1/HLA-DQA1/HLA-DPA1/HLA-DOB/CALR/CTSB/PDIA3/CTSL/CREB1/RFX5/CANX/CD4/HSP90AB1/PSME3/TAPBP/HSP90AA1/HLA-DPB1/HSPA4
KEGG_COMPLEMENT_AND_COAGULATION_CASCADES
KEGG_COMPLEMENT_AND_COAGULATION_CASCADES
KEGG_COMPLEMENT_AND_COAGULATION_CASCADES 67 0.714846639 2.224801265
0.00147929 0.015220949 0.011025928 1186 tags=36%, list=7%, signal=33%
CD55/C4BPB/C4BPA/CFB/CFI/PLAU/VWF/THBD/C1S/C3/SERPING1/F3/SERPINE1/SERPINA1/C2/C1R/TFPI/F2R/C5AR1/C1QB/PLAT/C1QA/A2M/C3AR1
KEGG_CYTOSOLIC_DNA_SENSING_PATHWAY KEGG_CYTOSOLIC_DNA_SENSING_PATHWAY
KEGG_CYTOSOLIC_DNA_SENSING_PATHWAY 51 0.59333669 1.777183894
0.00149925 0.015220949 0.011025928 2886 tags=35%, list=17%, signal=29%
CXCL10/IL1B/CASP1/IL33/AIM2/STING1/CCL4/IL6/ZBP1/RELA/IRF7/POLR3H/ADAR/POLR3G/IL18/IRF3/TBK1/POLR1C
KEGG_AUTOIMMUNE_THYROID_DISEASE KEGG_AUTOIMMUNE_THYROID_DISEASE
KEGG_AUTOIMMUNE_THYROID_DISEASE 45 0.650365118 1.900697952 0.001503759
0.015220949 0.011025928 1063 tags=22%, list=6%, signal=21%
HLA-DMA/HLA-DQB1/HLA-DRA/GZMB/HLA-DMB/CD86/HLA-DQA1/CD40/HLA-DPA1/HLA-DOB
KEGG_INTESTINAL_IMMUNE_NETWORK_FOR_IGA_PRODUCTION

KEGG_INTESTINAL_IMMUNE_NETWORK_FOR_IGA_PRODUCTION
KEGG_INTESTINAL_IMMUNE_NETWORK_FOR_IGA_PRODUCTION 43 0.723093335 2.092317622 0.00152207 0.015220949 0.011025928 1721 tags=42%, list=10%, signal=38%
TNFRSF17/HLA-DMA/CCR10/TGFB1/HLA-DQB1/HLA-DRA/HLA-DMB/TNFSF13/TNFSF13B/MADCAM1/CXCR4/CD86/HLA-DQA1/CD40/HLA-DPA1/HLA-DOB/IL15RA/IL6

KEGG_PROTEASOME KEGG_PROTEASOME KEGG_PROTEASOME 41 0.694392252 1.985979501 0.001538462 0.015220949 0.011025928 4036 tags=80%, list=24%, signal=61%
PSME4/PSMB9/PSMD14/PSMD12/PSMC2/PSMB2/PSMA3/PSMD1/PSMA5/PSMD6/IFNG/PSMB5/PSMC4/PSMB1/PSMA1/PSME3/POMP/PSMA7/PSMB8/PSMC5/PSMB10/PSMB6/PSMA4/PSMD13/PSME1/PSMC1/PSMB4/PSMB3/PSMD2/PSMC6/PSMD7/PSMA2/PSMD4

KEGG_ALLOGRAFT_REJECTION KEGG_ALLOGRAFT_REJECTION KEGG_ALLOGRAFT_REJECTION 32 0.690167752 1.906280432 0.001540832 0.015220949 0.011025928 1063 tags=31%, list=6%, signal=29%
HLA-DMA/HLA-DQB1/HLA-DRA/GZMB/HLA-DMB/CD86/HLA-DQA1/CD40/HLA-DPA1/HLA-DOB

KEGG_GRAFT_VERSUS_HOST_DISEASE KEGG_GRAFT_VERSUS_HOST_DISEASE KEGG_GRAFT_VERSUS_HOST_DISEASE 32 0.718102717 1.983438308 0.001540832 0.015220949 0.011025928 1872 tags=41%, list=11%, signal=36%
IL1B/HLA-DMA/HLA-DQB1/HLA-DRA/GZMB/HLA-DMB/CD86/HLA-DQA1/HLA-DPA1/IL1A/HLA-DOB/IL6/IFNG

KEGG_TYPE_I_DIABETES_MELLITUS KEGG_TYPE_I_DIABETES_MELLITUS KEGG_TYPE_I_DIABETES_MELLITUS 38 0.680539296 1.92082392 0.001569859 0.015220949 0.011025928 1063 tags=29%, list=6%, signal=27%
IL1B/HLA-DMA/HLA-DQB1/HLA-DRA/GZMB/HLA-DMB/CD86/HLA-DQA1/HLA-DPA1/IL1A/HLA-DOB

KEGG_PROTEIN_EXPORT KEGG_PROTEIN_EXPORT KEGG_PROTEIN_EXPORT 23 0.72606304 1.86226285 0.001631321 0.015220949 0.011025928 3275 tags=70%, list=19%, signal=56%
SRPRB/SEC11C/HSPA5/SPCS3/SRPRA/SRP19/SEC61A1/SRP68/SRP72/SEC61B/SRP54/SEC61G/SPCS2/SPCS1/SEC62/SEC63

KEGG_ASTHMA KEGG_ASTHMA KEGG_ASTHMA 25 0.768166668 1.988242338 0.001636661 0.015220949 0.011025928 1063 tags=40%, list=6%, signal=38%
CCL11/HLA-DMA/HLA-DQB1/HLA-DRA/HLA-DMB/FCER1G/HLA-DQA1/CD40/HLA-DPA1/HLA-DOB

KEGG_PROXIMAL_TUBULE_BICARBONATE_RECLAMATION
KEGG_PROXIMAL_TUBULE_BICARBONATE_RECLAMATION
KEGG_PROXIMAL_TUBULE_BICARBONATE_RECLAMATION 23 -0.756855837 -2.112053472 0.002570694 0.018719807 0.013560471 1174 tags=43%, list=7%, signal=41% GLUD1/GLS/PCK2/GLUD2/ATP1B3/CA2/SLC9A3/SLC4A4/CA4/PCK1

KEGG_FATTY_ACID_METABOLISM KEGG_FATTY_ACID_METABOLISM KEGG_FATTY_ACID_METABOLISM 40 -0.702639274 -2.206988763 0.002816901 0.018719807 0.013560471 1292 tags=55%, list=8%, signal=51%

ADH5/ECHS1/ALDH1B1/ADH4/HADHB/ECI1/ACSL5/ACAA1/ADH1A/HADH/HADHA/ACAA2/CPT2/ACADSB/ADH6/CPT1A/ACADS/EHHADH/ACADM/ACAT1/ACOX1/ADH1C
KEGG_PROPANOATE_METABOLISM KEGG_PROPANOATE_METABOLISM
KEGG_PROPANOATE_METABOLISM 32 -0.6679178 -2.003757531 0.002832861 0.018719807 0.013560471 2752 tags=56%, list=16%, signal=47% ACAT2/MMUT/ACACB/ECHS1/ALDH1B1/ALDH6A1/ACSS1/SUCLG1/SUCLA2/HIBCH/PCCA/ACSS2/HADHA/SUCLG2/EHHADH/ACADM/ACAT1/ABAT
KEGG_BUTANOATE_METABOLISM KEGG_BUTANOATE_METABOLISM
KEGG_BUTANOATE_METABOLISM 33 -0.701276238 -2.121282207 0.002849003 0.018719807 0.013560471 1510 tags=55%, list=9%, signal=50% ACAT2/PDHA1/ECHS1/ALDH1B1/L2HGDH/HMGCL/AKR1B10/BDH1/BDH2/HADH/HADHA/ACSM3/ALDH5A1/ACADS/EHHADH/ACAT1/ABAT/HMGCS2
KEGG_HEMATOPOIETIC_CELL_LINEAGE KEGG_HEMATOPOIETIC_CELL_LINEAGE
KEGG_HEMATOPOIETIC_CELL_LINEAGE 82 0.549985475 1.760863093 0.002853067 0.018719807 0.013560471 1851 tags=29%, list=11%, signal=26% CD55/IL1B/CD38/MS4A1/IL7R/ITGA2/CD44/HLA-DRA/ITGA5/IL1R1/KIT/CD19/CSF3R/CD37/ITGAM/IL1A/MME/CSF1R/ITGA6/CD2/CR2/CSF2RA/IL6/IL3RA
KEGG_RETINOL_METABOLISM KEGG_RETINOL_METABOLISM
KEGG_RETINOL_METABOLISM 45 -0.6249201 -1.995187582 0.002967359 0.018719807 0.013560471 1979 tags=29%, list=12%, signal=26% DHRS3/PNPLA4/ADH4/ALDH1A1/CYP1A1/ADH1A/UGT2B28/ADH6/BCO1/RETSAT/CYP2B6/ADH1C/UGT2A3
KEGG_VALINE_LEUCINE_AND_ISOLEUCINE_DEGRADATION
KEGG_VALINE_LEUCINE_AND_ISOLEUCINE_DEGRADATION
KEGG_VALINE_LEUCINE_AND_ISOLEUCINE_DEGRADATION 44 -0.674019983 -2.144618212 0.002967359 0.018719807 0.013560471 2302 tags=52%, list=14%, signal=45%
IVD/ECHS1/ALDH1B1/ALDH6A1/DLD/HMGCL/HADHB/HIBCH/PCCA/ACAA1/HADH/HADHA/BCKDHB/ACAA2/BCAT2/ACADSB/ACADS/AUH/EHHADH/ACADM/ACAT1/ABAT/HMGCS2
KEGG_DRUG_METABOLISM_CYTOCHROME_P450
KEGG_DRUG_METABOLISM_CYTOCHROME_P450
KEGG_DRUG_METABOLISM_CYTOCHROME_P450 55 -0.574548002 -1.922878303 0.00297619 0.018719807 0.013560471 2150 tags=29%, list=13%, signal=25% GSTA1/ADH4/GSTM1/MGST3/GSTM2/GSTM3/ADH1A/UGT2B28/ADH6/GSTM4/MAOA/FMO4/FMO5/CYP2B6/ADH1C/UGT2A3
KEGG_METABOLISM_OF_XENOBIOTICS_BY_CYTOCHROME_P450
KEGG_METABOLISM_OF_XENOBIOTICS_BY_CYTOCHROME_P450
KEGG_METABOLISM_OF_XENOBIOTICS_BY_CYTOCHROME_P450 53 -0.593953605 -1.955204391 0.003012048 0.018719807 0.013560471 2165 tags=36%, list=13%, signal=31%
AKR1C1/GSTA1/ADH4/GSTM1/MGST3/GSTM2/AKR1C2/CYP1A1/GSTM3/AKR1C3/ADH1A/EPHX1/UGT2B28/ADH6/GSTM4/CYP2S1/CYP2B6/ADH1C/UGT2A3
KEGG_PRIMARY_IMMUNODEFICIENCY KEGG_PRIMARY_IMMUNODEFICIENCY
KEGG_PRIMARY_IMMUNODEFICIENCY 35 0.630709032 1.757782636 0.003115265

0.018719807 0.013560471 2521 tags=40%, list=15%, signal=34%
IL7R/ADA/JAK3/PTPRC/TAP2/CD19/TAP1/CD40/CD79A/LCK/BTK/RFX5/CD4/CD3D
KEGG_PEROXISOME KEGG_PEROXISOME KEGG_PEROXISOME 77 -0.513110028 -1.829130389 0.003125 0.018719807 0.013560471 3537 tags=51%, list=21%, signal=40%
ECI2/MLYCD/SLC27A2/PEX2/PEX1/PEX14/IDH1/ACSL6/AGXT/FAR1/MPV17L/FAR2/DAO/PECR/DDO/CRAT/SCP2/AGPS/PXMP4/PRDX5/HMGCL/PEX13/PEX19/ACSL5/CAT/ECH1/ACAA1/ACOT8/ACOX2/ABCD3/NUDT12/CROT/PEX11A/EHHADH/PXMP2/PHYH/EPHX2/PEX26/ACOX1
KEGG_GLYCOSAMINOGLYCAN_BIOSYNTHESIS_CHONDROITIN_SULFATE KEGG_GLYCOSAMINOGLYCAN_BIOSYNTHESIS_CHONDROITIN_SULFATE KEGG_GLYCOSAMINOGLYCAN_BIOSYNTHESIS_CHONDROITIN_SULFATE 22 0.677458724 1.729149628 0.003220612 0.018719807 0.013560471 2576 tags=50%, list=15%, signal=42%
CSGALNACT1/CHST15/CHST11/CHSY1/DSE/CHPF/B3GALT6/CSGALNACT2/CHST12/CHPF2/CHST3
KEGG_PATHWAYS_IN_CANCER KEGG_PATHWAYS_IN_CANCER KEGG_PATHWAYS_IN_CANCER 320 0.386256846 1.434147831 0.003550296 0.020010758 0.014495626 3071 tags=28%, list=18%, signal=24%
MMP1/COL4A1/CXCL8/WNT5A/MMP9/MMP2/NOS2/LAMC1/ITGA2/LAMC2/LAMA3/COL4A2/PIK3R3/TGFB1/BIRC3/RAC2/LAMA4/HIF1A/PTGS2/FN1/BIRC5/KIT/TGFBR2/RASSF5/PDGFRA/CSF3R/SHH/STAT1/PIAS3/PDGFRB/MET/PLCG2/BCR/VEGFC/LEF1/STAT3/PIK3CG/CSF1R/FGFR1/TRAF5/PRKCB/LAMB1/PIK3CD/CCND1/BID/ITGA6/HSP90B1/LAMB2/PIK3R5/E2F3/ITGAV/CSF2RA/AKT3/PML/CASP8/IL6/MSH6/RASSF1/CDK2/LAMA5/FGF7/MAP2K1/MLH1/FZD4/RELA/WNT4/TGFBR1/TRAF1/NFKB2/CDK4/WNT2/ZBTB16/HSP90AB1/TGFB3/TRAF3/ELOC/ETS1/AKT1/RUNX1T1/CTBP2/DVL1/SLC2A1/FGF2/JAK1/MITF/HSP90AA1/STAT5B/HRAS/ITGA3/STAT5A/GSTP1
KEGG_LIMONENE_AND_PINENE_DEGRADATION KEGG_LIMONENE_AND_PINENE_DEGRADATION KEGG_LIMONENE_AND_PINENE_DEGRADATION 10 -0.771177851 -1.759072492 0.004705882 0.025743945 0.018648698 2232 tags=50%, list=13%, signal=43%
ECHS1/ALDH1B1/YOD1/HADHA/EHHADH
KEGG_NITROGEN_METABOLISM KEGG_NITROGEN_METABOLISM KEGG_NITROGEN_METABOLISM 22 -0.697394992 -1.935996028 0.005249344 0.027659021 0.020035963 1700 tags=45%, list=10%, signal=41%
GLUD1/CA7/GLS/GLUD2/AMT/CA12/CTH/CA2/CA4/CA1
KEGG_CITRATE_CYCLE_TCA_CYCLEKEGG_CITRATE_CYCLE_TCA_CYCLE KEGG_CITRATE_CYCLE_TCA_CYCLE29 -0.610484425 -1.793262125 0.005390836 0.027659021 0.020035963 3540 tags=62%, list=21%, signal=49%
OGDH/OGDHL/IDH1/SDHA/PDHA1/IDH3B/IDH3A/MDH1/DLAT/SUCLG1/DLD/SUCLA2/SDHB/ACO2/PCK2/SDHD/SUCLG2/PCK1
KEGG_LEUKOCYTE_TRANSENDOTHELIAL_MIGRATION KEGG_LEUKOCYTE_TRANSENDOTHELIAL_MIGRATION KEGG_LEUKOCYTE_TRANSENDOTHELIAL_MIGRATION 112 0.473659541 1.575565727 0.005502063 0.027659021 0.020035963 1649 tags=29%, list=10%, signal=26%
PECAM1/CLDN1/NCF2/MMP9/MMP2/MSN/CDH5/PIK3R3/RAC2/VCAM1/RASSF5/CLDN2/IT

GB2/THY1/CXCR4/GNAI2/MYL9/ICAM1/PLCG2/ACTN1/RHOH/ITGAM/JAM2/VAV1/PIK3CG/CLDN18/PRKCB/PIK3CD/JAM3/PIK3R5/ITK/VCL
KEGG_NOD_LIKE_RECEPTOR_SIGNALING_PATHWAY KEGG_NOD_LIKE_RECEPTOR_SIGNALING_PATHWAY KEGG_NOD_LIKE_RECEPTOR_SIGNALING_PATHWAY 61 0.545095298 1.66725067 0.005899705 0.028877503 0.020918622 2677 tags=33%, list=16%, signal=28% CXCL1/CCL11/CXCL8/IL1B/CXCL2/CARD6/CASP1/BIRC3/RIPK2/CCL2/TRIP6/CASP5/HSP90B1/CASP8/IL6/RELA/NOD2/HSP90AB1/CCL7/IL18
KEGG_PPAR_SIGNALING_PATHWAY KEGG_PPAR_SIGNALING_PATHWAY KEGG_PPAR_SIGNALING_PATHWAY 66 -0.466874922 -1.615013269 0.00621118 0.029622551 0.021458328 1235 tags=32%, list=7%, signal=30% SCP2/FABP2/RXRA/PDPK1/NR1H3/PPARA/PCK2/ACSL5/PPARD/ACAA1/ACOX2/CPT2/FABP1/CPT1A/EHHADH/ACADM/CYP27A1/PPARG/ACOX1/HMGCS2/PCK1
KEGG_OXIDATIVE_PHOSPHORYLATION KEGG_OXIDATIVE_PHOSPHORYLATION KEGG_OXIDATIVE_PHOSPHORYLATION 111 -0.432746117 -1.602166506 0.007246377 0.033695652 0.024408848 4265 tags=52%, list=25%, signal=39% COX7C/ATP6V1C1/COX6B1/ATP5F1A/NDUFA6/COX7A2/ATP6V0E1/NDUFB2/ATP5MC1/UQCRQ/ATP5PO/ATP5F1E/NDUFA3/NDUFS1/ATP6V1D/UQCR11/NDUFS3/ATP6V0D1/COX10/COX7B/NDUFS4/ATP12A/SDHA/UQCR10/COX11/NDUFS7/CYC1/ATP5F1D/ATP5PB/UQCRFS1/NDUFS2/COX8A/LHPP/COX6A1/COX4I1/UQCRC2/NDUFB10/ATP5MC2/NDUFB5/ATP5PF/ATP5F1B/NDUFB9/UQCRC1/NDUFA1/ATP5MC3/NDUFB1/COX5A/COX6C/ATP6V0D2/NDUFV1/COX5B/SDHB/COX15/NDUFA10/NDUFA2/NDUFB7/SDHD/NDUFA5
KEGG_SMALL_CELL_LUNG_CANCER KEGG_SMALL_CELL_LUNG_CANCER KEGG_SMALL_CELL_LUNG_CANCER 84 0.509181147 1.642646925 0.008403361 0.038122566 0.027615668 2602 tags=37%, list=15%, signal=31% COL4A1/NOS2/LAMC1/ITGA2/LAMC2/LAMA3/COL4A2/PIK3R3/BIRC3/LAMA4/PTGS2/FN1/PIAS3/PIK3CG/TRAF5/LAMB1/PIK3CD/CCND1/ITGA6/LAMB2/PIK3R5/E2F3/ITGAV/AKT3/CDK2/LAMA5/RELA/TRAF1/CDK4/TRAF3/AKT1
KEGG_ABC_TRANSPORTERS KEGG_ABC_TRANSPORTERS KEGG_ABC_TRANSPORTERS 43 -0.56169239 -1.779853088 0.008695652 0.038509317 0.027895827 2035 tags=28%, list=12%, signal=25% ABCB10/ABCB7/ABCC3/ABCC6/CFTR/ABCG1/ABCD3/ABCA5/ABCA8/ABCB11/ABCB1/ABCG2
KEGG_NATURAL_KILLER_CELL_MEDIATED_CYTOTOXICITY KEGG_NATURAL_KILLER_CELL_MEDIATED_CYTOTOXICITY KEGG_NATURAL_KILLER_CELL_MEDIATED_CYTOTOXICITY 123 0.42377553 1.426963744 0.010869565 0.045948617 0.033284793 3308 tags=30%, list=20%, signal=24% FCGR3B/PIK3R3/LCP2/RAC2/GZMB/FCER1G/FYN/ITGB2/ICAM1/MICB/PLCG2/TYROBP/ICAM2/TNFRSF10A/VAV1/TNFRSF10B/PIK3CG/LCK/IFNAR2/PRKCB/PIK3CD/BID/PIK3R5/MAP2K1/IFNG/PPP3CA/SHC2/PPP3R1/SYK/TNFRSF10C/CD48/PTPN11/NFATC3/HRAS/LAT/SHC1/CD247
KEGG_PRION_DISEASES KEGG_PRION_DISEASES KEGG_PRION_DISEASES 34 0.600703251 1.667113913 0.010869565 0.045948617 0.033284793 1861 tags=32%, list=11%, signal=29% IL1B/LAMC1/HSPA5/FYN/C1QB/IL1A/C1QA/STIP1/PRNP/IL6/MAP2K1
KEGG_STARCH_AND_SUCROSE_METABOLISM KEGG_STARCH_AND_SUCROSE_METABOLISM KEGG_STARCH_AND_SUCROSE_METABOLISM 33 -0.573298154 -1.734162813

0.011396011 0.047103514 0.034121391 1364 tags=30%, list=8%, signal=28% TREH/ENPP3/PGM1/AGL/UGP2/UGDH/UGT2B28/ENPP1/GBA3/UGT2A3
KEGG_DNA_REPLICATION KEGG_DNA_REPLICATION KEGG_DNA_REPLICATION 36 0.564290199 1.581852402 0.01399689 0.056078148 0.04062254 3444 tags=42%, list=20%, signal=33% FEN1/POLD3/MCM6/RFC3/POLE2/MCM3/MCM5/SSBP1/RFC4/POLD2/MCM7/RNASEH2A/RNASEH1/POLA2/MCM2
KEGG_JAK_STAT_SIGNALING_PATHWAY KEGG_JAK_STAT_SIGNALING_PATHWAY KEGG_JAK_STAT_SIGNALING_PATHWAY 152 0.401062364 1.394659062 0.014248705 0.056078148 0.04062254 3052 tags=27%, list=18%, signal=22% IL13RA2/CSF2RB/IL7R/PIK3R3/JAK3/SOCS3/CSF3R/JAK2/STAT1/PIAS3/IL10RA/STAT4/STAT3/IL6ST/PIK3CG/SPRED1/IFNAR2/IL12RB1/PIK3CD/CCND1/IL15RA/PIK3R5/CSF2RA/LIF/IL23A/AKT3/IL6/TSLP/IRF9/IL3RA/IFNG/STAT2/IL24/AKT1/TYK2/JAK1/SOCS4/STAT5B/PTPN11/SPRY1/STAT5A
KEGG_B_CELL_RECEPTOR_SIGNALING_PATHWAY KEGG_B_CELL_RECEPTOR_SIGNALING_PATHWAY KEGG_B_CELL_RECEPTOR_SIGNALING_PATHWAY 75 0.492329972 1.550504494 0.01447178 0.056078148 0.04062254 2602 tags=41%, list=15%, signal=35% DAPP1/LYN/PIK3R3/IFITM1/PIK3AP1/RAC2/CD19/FCGR2B/PLCG2/CD81/VAV1/CD79B/CD79A/RASGRP3/PIK3CG/PRKCB/PIK3CD/CD72/CR2/BTK/PIK3R5/AKT3/MAP2K1/LILRB3/RELA/PPP3CA/INPP5D/MALT1/PPP3R1/SYK/AKT1
KEGG_SPLICEOSOME KEGG_SPLICEOSOME KEGG_SPLICEOSOME 115 0.43641104 1.456642933 0.01504788 0.057120523 0.041377628 5325 tags=55%, list=31%, signal=38% PPIL1/HSPA6/MAGOHB/SNRNP70/SNRPG/SNRPD1/SRSF7/PRPF4/SNRPA1/SNRPF/SNRPB2/PRPF31/THOC1/SNRPB/TRA2B/SF3B3/SNRPA/LSM6/TCERG1/HNRNPC/ALYREF/TXNL4A/PRPF40A/EIF4A3/SNRPD2/RBM8A/CTNNBL1/SNRPC/SRSF1/DDX23/LSM7/SART1/RBM17/PPIH/SF3B2/SRSF3/PCBP1/SNRPE/SF3A2/SRSF4/SRSF6/EFTUD2/SNU13/PRPF3/XAB2/SMNDC1/U2AF1/HNRNPU/PUF60/ACIN1/DDX46/SF3B5/PRPF8/HNRNPM/HNRNPA3/PRPF19/DDX42/SF3B4/BCAS2/DHX15/DHX8/LSM2/CCDC12
KEGG_PENTOSE_AND_GLUCURONATE_INTERCONVERSIONS KEGG_PENTOSE_AND_GLUCURONATE_INTERCONVERSIONS KEGG_PENTOSE_AND_GLUCURONATE_INTERCONVERSIONS 14 -0.680000574 -1.668111192 0.020460358 0.076112532 0.055135281 683 tags=36%, list=4%, signal=34% CRYL1/UGP2/UGDH/UGT2B28/UGT2A3
KEGG_ASCORBATE_AND_ALDARATE_METABOLISM KEGG_ASCORBATE_AND_ALDARATE_METABOLISM KEGG_ASCORBATE_AND_ALDARATE_METABOLISM 12 -0.681030355 -1.614519605 0.022900763 0.083520431 0.060501501 615 tags=33%, list=4%, signal=32% ALDH1B1/UGDH/UGT2B28/UGT2A3
